# TTC33 forms a complex with WDR61 and PHF5A to recruit factors relevant to genomic integrity

**DOI:** 10.1038/s41467-026-76928-z

**Published:** 2026-08-21

**Authors:** Rafał Tomecki, Małgorzata Drabko, Małgorzata Siek, Maria Matykiewicz, Miłosz Ludwinek, Łukasz S. Borowski, Aneta Jurkiewicz, Kamil Kobyłecki, Agnieszka Jabłońska, Dominik Cysewski, Agata Malinowska, Magdalena Bakun, Rafał Płoski, Roman J. Szczęsny, Agnieszka Tudek

**Affiliations:** 1 Laboratory of RNA Processing and Decay, Institute of Biochemistry and Biophysics, Polish Academy of Sciences, Warsaw, Poland; 2 Laboratory of Epitranscriptomics, Institute of Bioengineering, Faculty of Biology, Biological and Chemical Research Centre, University of Warsaw, Warsaw, Poland; 3 Doctoral School of Molecular Biology and Biological Chemistry at IBB PAS, Warsaw, Poland; 4 Laboratory of RNA Biology, Institute of Biochemistry and Biophysics, Polish Academy of Sciences, Warsaw, Poland; 5 Faculty of Biology, Institute of Genetics and Biotechnology, University of Warsaw, Warsaw, Poland; 6 Mass Spectrometry Laboratory, Institute of Biochemistry and Biophysics, Polish Academy of Sciences, Warsaw, Poland; 7 Department of Medical Genetics, Medical University of Warsaw, Warsaw, Poland; 8 Present Address: Chair and Department of Biochemistry, Medical University of Warsaw, Warsaw, Poland; 9 Present Address: Centre for Regenerative Medicine, Medical University of Białystok, Białystok, Poland

**Keywords:** Protein-protein interaction networks, Double-strand DNA breaks

## Abstract

The functions of many human proteins remain unknown, highlighting major gaps in our understanding of cellular biology. TTC33 is an evolutionarily conserved tetratricopeptide repeat (TPR) protein expressed across human tissues, whose molecular role is not defined. Here we identify the TTC33 partners using comparative label-free mass spectrometry. The TTC33-associated network (TAN) comprises WDR61, CCDC97, UNG1/2, PP2A-B55α, PHF5A, and components of the SF3B U2 spliceosomal complex. Using a combination of biochemical assays, structural modeling and molecular dynamics we show that TTC33 directly recruits WDR61 and PHF5A to assemble into a trimeric core complex (TANC), which then forms distinct interactions with either UNG1/2 or SF3B–CCDC97. Somatic TTC33 mutations at key TANC interface residues reduce complex stability and weaken the interaction network. We further show that WDR61 stabilizes TTC33 by protecting it from proteolytic degradation. Loss of network components induces genomic instability and activates DNA damage markers, including γH2AX and p53 phosphorylation.

## Abstract (web summary)

Authors identify the TANC complex, comprising TTC33, WDR61 and PHF5A, and show that it nucleates a protein interaction network containing factors implicated in splicing and DNA repair. Its disruption promotes genomic instability.

## Introduction

Carcinogenesis is induced by external genotoxicity and heritable genetics. Few single mutations are associated with high disease risk. The overwhelming majority of cases are polygenic. Genome-wide association studies advance the identification of risk variants, which may influence protein expression patterns in cancer and affect growth, migration, and DNA repair. However, functional understanding of associations is insufficient, partly because 9% of the human proteome is largely uncharacterized^1^.

DNA lesions, such as damaged bases, DNA crosslinks, and single- or double-stranded DNA breaks (SSBs or DSBs), are the main drivers of cancer. Depending on the lesion, the cell has many, at times redundant, repair mechanisms. Base Excision Repair (BER) targets bases damaged by alkylating or oxidizing agents, and is initiated by a collection of lesion-dedicated glycosylases^2,3^. Base excision creates an apurinic/apyrimidinic (AP) site, which is endonucleolytically cleaved by APE1. Hereby, the double-strand helix is opened, granting access to the DNA polymerase. After complementary strand synthesis, the displaced oligonucleotide is cleaved off to enable ligation. Uracil, which is a major driver of G/A mutations, arises from spontaneous cytosine deamination or is incorporated during replication. Primarily, it is targeted by uracil DNA glycosylase 1/2 (UNG1/2), or with lesser efficiency by SMUG1^4^, but can also be a substrate for Nucleotide Incision Repair^5^, highlighting pathway redundancies. Essentially, BER induces DNA SSBs, however, if multiple lesions occur in proximity, it results in DSBs. The latter also occur during natural processes (meiosis, antibody class switch recombination promoted by AID cytosine deaminase and UNG1/2), and are induced following exposure to external stimuli (e.g., reactive oxygen species: ROS, ionizing radiation, chemotherapeutics – doxorubicin). DSBs can lead to genomic rearrangements, which, if not lethal, accelerate carcinogenesis. They are repaired by error-free homologous recombination (HR) and error-prone non-homologous end joining (NHEJ) or alternative end joining (a-EJ)^6,7^.

DNA lesions, such as DSBs and SSBs, activate the phosphoinositide-3-kinase-related protein (PIKK) kinases, which phosphorylate the H2AX variant of histone H2A at serine 139, leading to the formation of γH2AX. Amplification of H2AX phosphorylation along the chromatin is promoted by the formation of repair foci, that channel further recruitment of DNA DSB repair enzymes^6,8^. After repair, γH2AX modification is removed, and here a role for the PP2A-B55α or Wip1 phosphatases was proposed^9,10^. PIKK kinases also modify p53 at various sites. p53 is short-lived due to MDM2-mediated ubiquitylation. p53 phosphorylation stabilizes the protein, activating it as a transcription factor. Phosphorylated p53 promotes transcription of p21 cyclin inhibitor, causing cell cycle arrest, and if DNA damage persists, it stimulates expression of pro-apoptotic genes^11^. The combination of p53 modifications determines which genes are transcribed, ultimately influencing cell survival or apoptosis. In particular, p53-serine15 phosphorylation (p53-S15P) induces DNA damage-related cell cycle arrest^12^. DNA repair enzymes are expressed in all cell types^13^. However, due to tissue-specific gene expression patterns or metabolic particularities, some cells may rely more heavily on certain repair pathways to maintain genomic integrity^14^.

Here, we investigated Tetratricopeptide Repeat (TPR) Domain 33 (TTC33), a predicted TPR-containing protein. TTC33 is encoded adjacent to *PRKAA1* within a genomic locus reported to undergo rearrangements in several cancers^15,16^. Apart from its annotation in large-scale interaction and cancer transcriptomic datasets, no direct functional characterization of TTC33 has been reported. Using comparative label-free mass spectrometry analysis, we showed that the TTC33-associated network (TAN) of interactors comprises WDR61, PP2A-B55α phosphatase, uncharacterized CCDC97, UNG1/2, as well as PHF5A and other subunits of the SF3A/B complex. A trimeric core complex formed by TTC33:WDR61:PHF5A (TANC) is a platform that recruits UNG1/2 or CCDC97-SF3A/B in a mutually exclusive manner. Structural TANC analyses revealed that WDR61 and PHF5A are recruited independently to the TTC33 core TPR domain. Importantly, somatic cancer mutations disrupted not only the TANC assembly, but also UNG1/2 binding. Essentially, WDR61 maintains protein levels of TTC33, which is otherwise subjected to proteolytic regulation. TAN is required for genomic integrity. Loss of TTC33 resulted in DNA fragmentation and increased nuclear accumulation of γH2AX in HEK293 cells. The latter phenotype was shared with WDR61, CCDC97, and PP2A-B55α depletions. Supporting phenotype specificity, γH2AX accumulation in WDR61-depleted cells could be rescued by expression of a wild-type protein, but not by a mutant incapable of binding to TTC33. TTC33 loss also led to nuclear re-distribution of p53-S15P, proliferative decrease due to accumulation of cells in G2 phase, and sensitized cells to ROS and doxorubicin. Analysis of TCGA clinical studies showed that TAN levels are associated with cancer survival rates in Kidney Renal Clear Cell Carcinoma (KIRC).

## Results

### TTC33 is composed of conserved TPR repeats

**Fig. 1:**
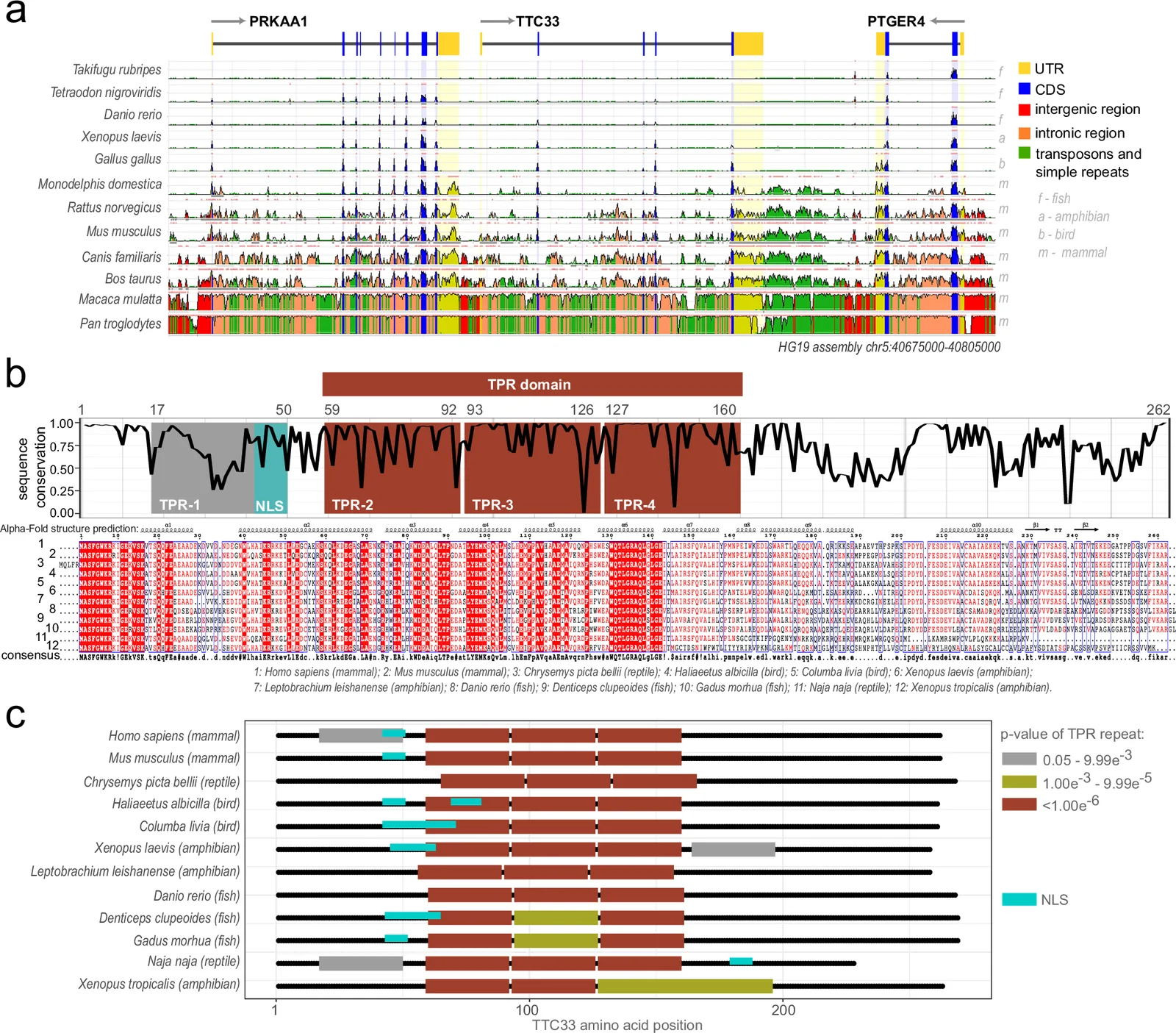
TTC33 is conserved in bony vertebrates. **a** Genomic alignment of the *TTC33* and surrounding *PRKAA1* and *PTGER4* loci derived from various vertebrate species (displayed using the ECR Browser suite^59^). In the human genome the gene is located on chromosome 5 (the genome assembly and coordinates are indicated on the figure). **b** Assessment of evolutionary conservation of the human TTC33 protein sequence. At the bottom is shown an example of multiple sequence alignment of TTC33 proteins from twelve species representing various vertebrate classes. The top graph displays the evolutionary conservation of each amino acid in the human TTC33 protein sequence based on the analysis of 300 vertebrate sequences. The number of identical residues was calculated and expressed as a fraction of the total. The position of the putative NLS and structural TPR1-2-3-4 motifs is shown. **c** Schematic representation of 12 vertebrate TTC33 proteins shown in (**b**). TPR motifs were detected using TPRpred^62–64^, and are color-coded according to the *p*-value range, which indicates the likelihood of adopting a TPR fold. Nuclear localization signal (NLS) sequences were detected using NLS mapper^60^. Source data are provided as a Source Data file.

TTC33 orthologs are encoded in bony vertebrate genomes (*Euteleostomi* clade) in a locus located between the *PTGER4* and *PRKAA1* genes. Typically, the *TTC33* mRNA is spliced from five exons: four coding, which show significant evolutionary conservation, and one containing the 5’UTR (Fig. 1a).

The human protein, similarly to its orthologs, is 262 residues long and weighs 29.4 kDa. Multiple protein sequence alignment of TTC33 orthologs from various classes of vertebrates revealed strong conservation within the protein N-terminal and middle parts (Fig. 1b). TTC33 proteins contain TPR motifs (Fig. 1c; Supplementary Fig. 1a). TPRs are typically 34 residues long and fold into two anti-parallel helices. TPRs scarcely display strong sequence conservation, but rather contain ordered repetitions of small and large hydrophobic amino acids. The former are usually located at positions 8, 20, and 27, whereas the latter are at positions 4, 7, 11, and 24. A proline is often situated at position 32, breaking the second alpha-helix. A cluster of three adjacent TPRs located in the TTC33 middle part was identified in various bony vertebrate sequences. Occasionally, additional TPRs were identified in the N- or C-terminal regions, albeit with lower confidence (Fig. 1c; Supplementary Fig. 1a; Supplementary Dataset 1). Since three TPRs are sufficient to produce a TPR fold, we predict that the central TTC33 domain adopts this fold^17,18^. Many TTC33 orthologues contain a putative nuclear localization signal located mostly in the N-terminal part (Fig. 1c; Supplementary Dataset 1). In humans, it overlaps with the degenerate TPR1 (42-HAIKRRKEI-50). In sum, we conclude that TTC33 is a conserved vertebrate-specific structural protein.

### Comparative label-free mass spectrometry (cLF-MS) identifies TTC33 interaction network (TAN)

**Fig. 2:**
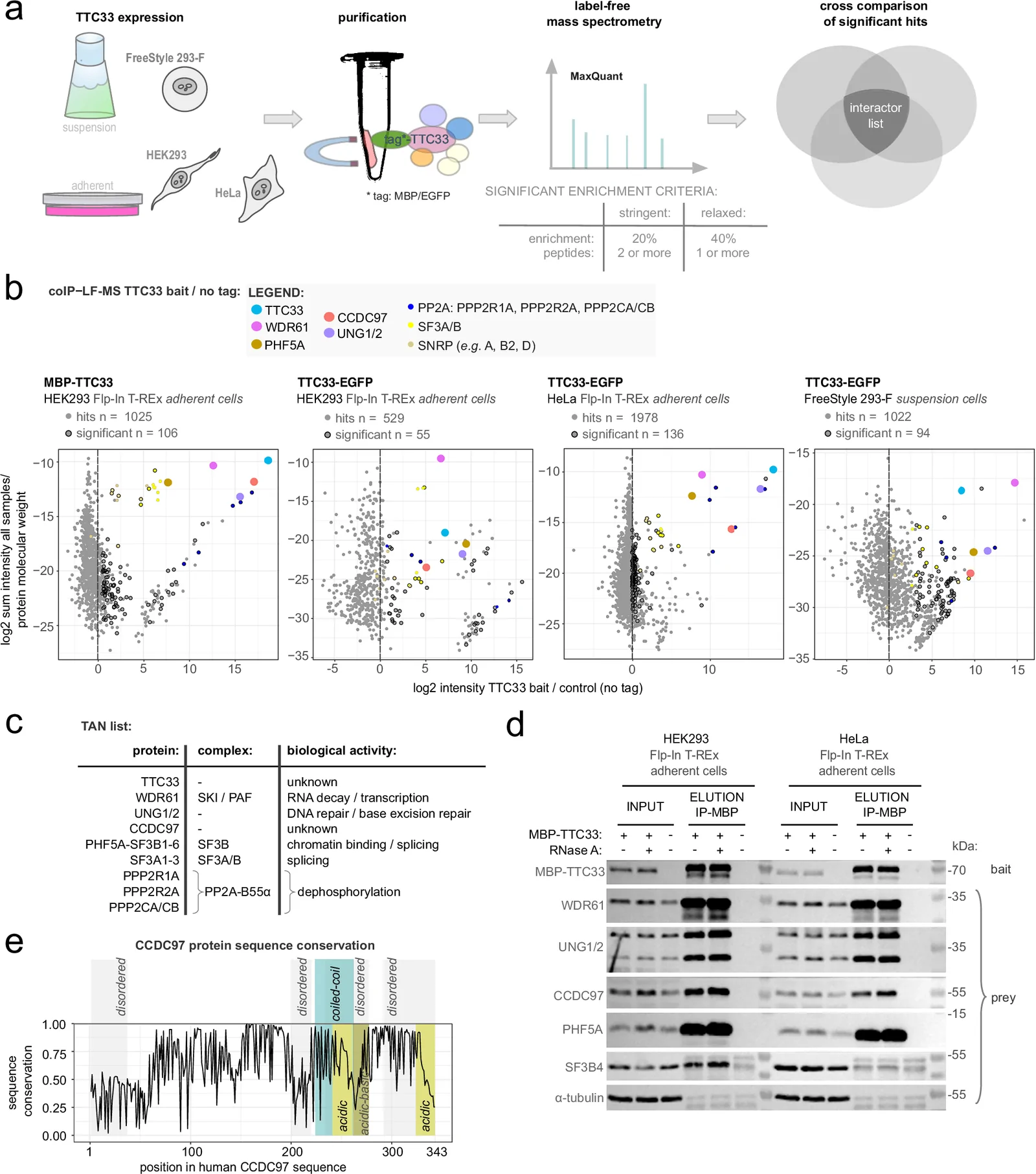
TTC33-associated network (TAN) was identified using comparative label-free mass spectrometry. **a** A scheme illustrating the cLF-MS approach leading to the identification of TTC33-interacting proteins. **b** Results of LF-MS analysis of proteins co-purifying with MBP-TTC33 bait from adherent HEK293 or with TTC33-EGFP fusion from either FreeStyle 293-F suspension cells, HEK293, or HeLa adherent cells. All identified proteins are shown as full gray dots, whereas the significantly enriched ones are highlighted as black circles. Amongst the latter, proteins of interest were additionally color-coded. **c** Table showing list of significant TTC33 interactors selected from experiments illustrated in (**a**, **b**). The details regarding the analysis are presented in the “Methods” section, in Supplementary Fig. 1f, and Supplementary Dataset 2. The latter presents all pre-filtered hits identified in each dataset, with log2-fold change values, and the complete cross-comparison of all TTC33 interactor datasets. **d** CoIP-WB analysis of proteins co-purifying with MBP-TTC33 from HEK293 or HeLa cells. Extracts were either treated or not with RNase A to assess if interactions were mediated by RNA. This was relevant mainly to SF3B, which is an RNA-binding complex^27^. Interaction with α-tubulin was assessed as a negative control. **e** Graph showing the conservation of the amino acid sequence of CCDC97 (as in (**b**), *top*; Supplementary Dataset 1). Colored boxes indicate the position of structural motifs, and regions of composition bias in the human protein. Source data are provided as a Source Data file.

To identify TTC33 protein interactors by mass spectrometry, we generated stable HEK293 and HeLa Flp-In T-REx cell lines (hereafter HEK293 and HeLa, respectively) expressing human TTC33 fused to C-terminal EGFP or N-terminal MBP under a tetracycline-inducible promoter^19^. In addition, TTC33-EGFP was transiently expressed in FreeStyle 293-F suspension cells. We purified TTC33 with its interactors at non-stringent low salt (100 mM NaCl) concentration and subjected the purified proteins to label-free mass spectrometry analysis (LF-MS; MaxQuant algorithm^20^), in total producing four datasets (TTC33-EGFP purified from HEK293, HeLa, and FreeStyle 293-F, and MBP-TTC33 purified from HEK293; Fig. 2a, b; Supplementary Fig. 1b–e). Being a sensitive method, MS is capable of identifying even transient interactions, but is known to produce many false positives in the process. Since we were interested in identifying the stable TTC33 interactors, we set several stringent and relaxed criteria to shortlist enriched proteins (Fig. 2a). The relaxed criteria gave a list of 1334 proteins identified in either of the four datasets, including 311 also passing the stringent threshold (Fig. 2a and “Methods”). Next, we determined in how many datasets each protein appeared using strict or relaxed criteria. As stable TTC33 interactors, we considered only proteins consistently identified in all four datasets using either criterion. However, if one or more such partners were known to partake in other complexes, subunits of which were also enriched but identified with lower consistency, we aggregated such partners (Supplementary Fig. 1f; Supplementary Dataset 2).We thus defined the TTC33-associated network (TAN) (Fig. 2c), and validated most interactions by co-immunoprecipitation coupled with western blot (coIP-WB) in HEK293 and HeLa (Fig. 2d).

The most promising TAN factor was WDR61, a 33.5 kDa structural protein composed of WD-repeats folding into a β-barrel^21^. WDR61 is a shared subunit of the *superkiller* complex (SKIc) and human Polymerase-Associated Factor complex (PAFc), partaking in cytoplasmic and nuclear RNA metabolism respectively. Both complexes are assembled around large, ~130 kDa TPR-containing scaffolding proteins, to which WDR61 binds directly (TTC37 in SKIc, CTR9 in PAFc^22–25^). Relaxed criteria identified the Splicing Factor 3 A and 3B (SF3A/B) complex subunits. Among ten SF3A/B proteins, the 14 kDa PHF5A, composed of a PHD-finger-like domain, was identified in the stringent screening^26,27^. PP2A subunits were also identified. This heterotrimeric enzyme exists in multiple isoforms with one of sixteen regulatory subunits plus scaffolding and catalytic proteins, each present in two variants^28^. We identified the scaffolding PPP2R1A, both catalytic PPP2CA/CB, and regulatory PPP2R2A subunits, which form the B55α-PP2A phosphatase. PPP2R2A is involved in cell cycle progression surveillance, Tau dephosphorylation, and response to DNA damage^9,29,30^. Using the stringent criteria, the UNG1/2 BER uracil DNA glycosylase was also consistently identified^3,4^. The final interactor, CCDC97, is encoded by yet another bony vertebrates uncharacterized gene (Supplementary Fig. 1g). CCDC97 protein sequence is evolutionarily conserved only at its C-terminus containing one structural coiled-coil motif, and several patches of positively or negatively charged residues (Fig. 2e).

**Fig. 3:**
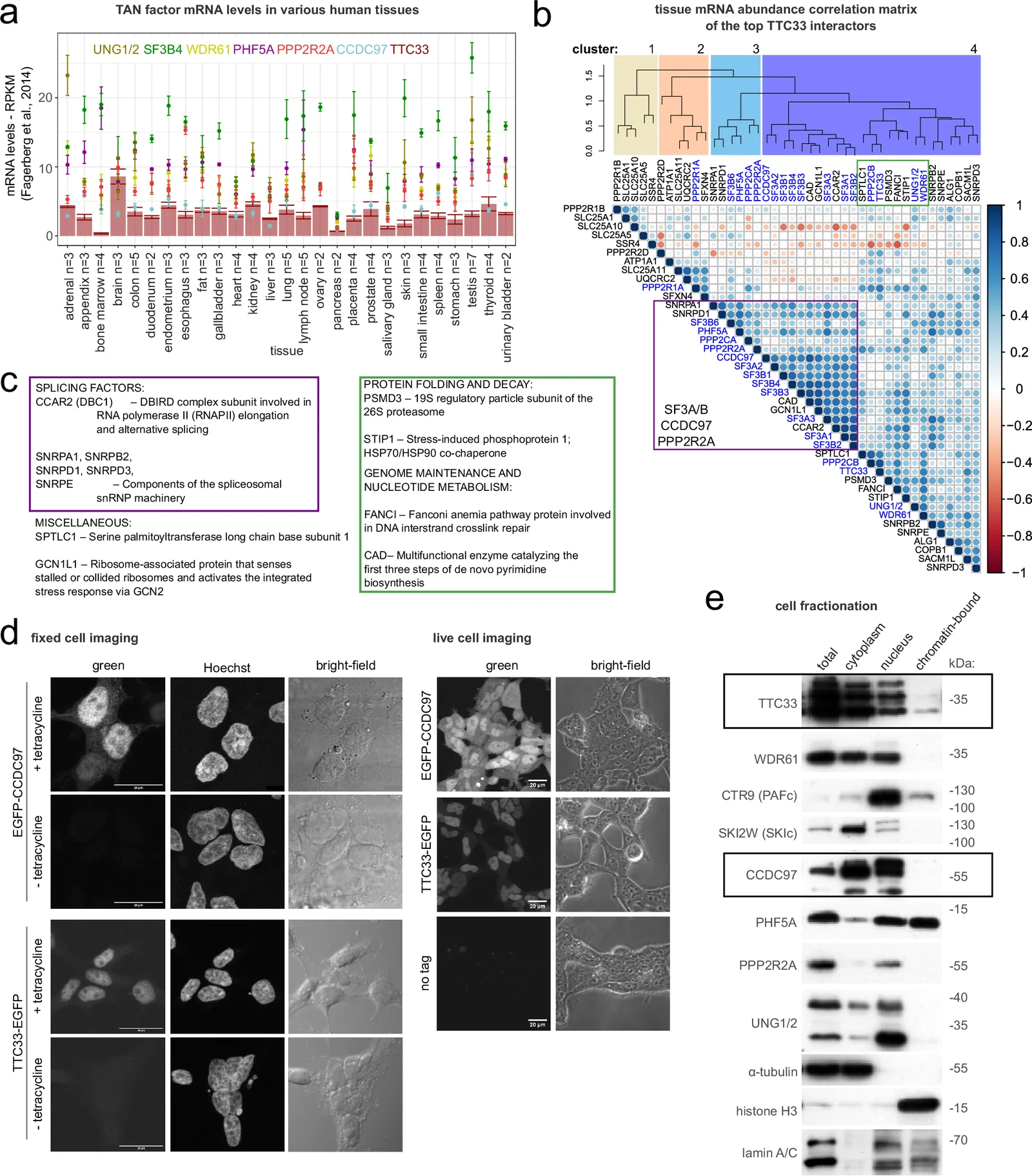
TTC33 and CCDC97 are ubiquitously expressed in various human tissues with other TAN factors and display a mixed cytoplasmic-nuclear localization. **a** The graph shows the mRNA levels of TTC33 and other TAN factors in various human tissues, as defined by Fagerberg et al.^31^. Mean expression values and corresponding standard deviations extracted from the original dataset are provided in the Source Data/Mendeley Data. The number of reported biological replicates is given (n). **b** Correlation matrix evaluating human tissue expression^31^ of TAN factors and an extended list of potential TTC33 interactors derived from LF-MS datasets (listed in Supplementary Fig. 1f and Supplementary Dataset 2). The matrix is clustered by expression similarity into four groups. Expression analysis limited to TAN factors is shown in Supplementary Fig. 2a. **c** Summarizes the roles of putative TTC33 interactors other than TAN, which closely clustered with either TAN subunit. **d** Images show sub-cellular localization of TTC33-EGFP or EGFP-CCDC97 in live and fixed HEK293 cells. Cells not expressing GFP were included as a negative control. **e** Western blot analysis of sub-cellular fractionation of HEK293 cells. Proteins were identified using specific antibodies. Source data are provided as a Source Data file.

TAN components are expressed in humans with no pronounced tissue specificity (Fig. 3a^31^). However, when compared with the extended list of TTC33 interactors, expression of TAN factors markedly correlated (Fig. 3b; Supplementary Fig. 1f, 2a). The TAN cluster was split into highly correlated SF3B-CCDC97 and more loosely associated TTC33-WDR61-UNG1/2. Within the extended interactor list, only a few additional factors showed correlated expression, primarily involved in splicing, protein folding and degradation, and DNA damage response or repair (Fig. 3c).

TTC33 contains a putative NLS (Fig. 1b, c), prompting us to examine its localization. Microscopy revealed that large fractions of EGFP-tagged TTC33 or CCDC97 were localized to the nucleus in living and fixed cells alike, though a cytoplasmic presence was also evident, especially for CCDC97 (Fig. 3d). These intracellular localization patterns were evidenced by cell fractionation coupled to western blot (Fig. 3e). Thus, TTC33 and CCDC97, similarly to WDR61, localize to both the nucleus and cytoplasm. Other TAN factors (SF3A/SF3B, UNG2, and PP2A-B55α) are predominantly nuclear (Fig. 3e; Supplementary Fig. 2b)^4,9,23,27,28,32^.

### TTC33:WDR61:PHF5A engage in mutually exclusive interactions with SF3A/B-CCDC97 and UNG1/2

**Fig. 4:**
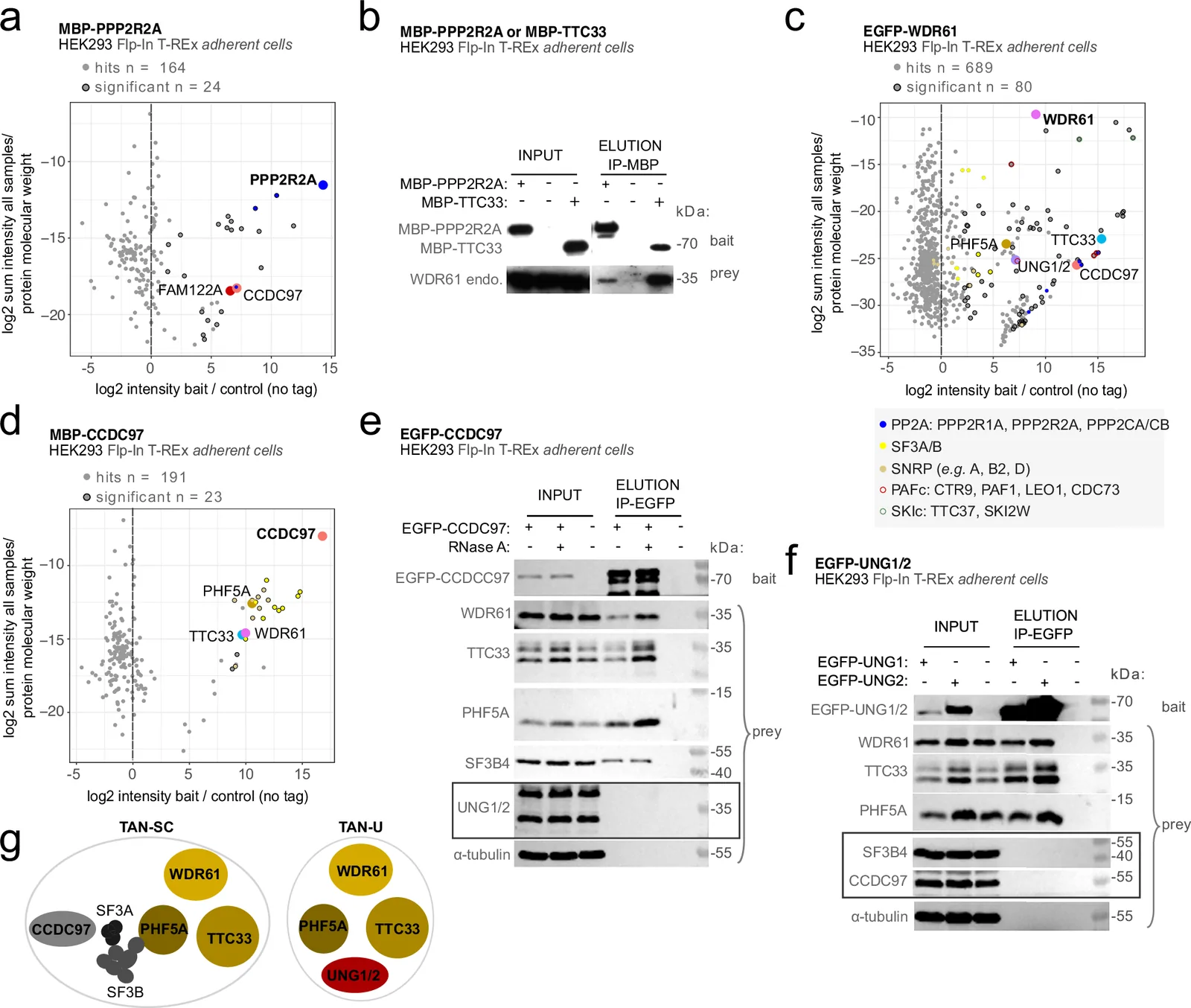
TTC33, WDR61, and PHF5A form mutually exclusive interactions with either UNG1/2 or CCDC97-SF3B. **a** Results of LF-MS analysis of proteins co-purifying with MBP-PPP2R2A from HEK293 cells. All hits are shown as gray dots, whereas the significant ones are outlined with black circles. Additionally, proteins of interest are color-coded and labeled. **b** CoIP-WB analysis of WDR61 association with MBP-tagged PPP2R2A or TTC33. **c** Results of LF-MS analysis of proteins co-purifying with EGFP-WDR61 from HEK293 cells. **d** Results of LF-MS analysis of proteins co-purifying with MBP-CCDC97 from HEK293 cells. **e** CoIP-WB analysis of TAN factors co-purifying with EGFP-CCDC97 from HeLa cells. Protein extracts were prepared with or without RNase A treatment to assess if CCDC97 co-purification with other TAN factors, particularly SF3B4, is mediated by nucleic acids. Association with α-tubulin was used as a negative control. The experiment confirms that UNG1/2 does not associate with CCDC97, whereas other TAN subunits, including TTC33, co-purify with the bait. The use of a different cell line and tag compared to the LF-MS data presented in (**d**) was deliberate to validate and strengthen the findings. **f** CoIP-WB analysis of TAN factors co-purifying with EGFP-UNG1 or EGFP-UNG2 from HEK293 cells. Complementing the analysis shown in (**e**), SF3B4 and CCDC97 do not co-purify with UNG1/2, whereas other TAN subunits associate with the baits. **g** A model proposing that the TAN is composed of two distinct networks, in which TTC33, PHF5A, and WDR61 associate either with CCDC97 and SF3B4, forming TAN-SC, or with UNG1/2, producing TAN-U. Source data are provided as a Source Data file.

To cross-validate the TAN list, we performed additional coIP-LF-MS analyses using tagged WDR61, PPP2R2A, or CCDC97, as baits. CoIP-LF-MS of PPP2R2A confirmed its preferential association with the PPP2R1A scaffold, and both PPP2CA and PPP2CB catalytic isoforms, but only CCDC97 was identified among TAN subunits (Fig. 4a). Instead, PPP2R2A co-purified with a known PP2A inhibitor, FAM122A^33,34^ (Fig. 4a), which was absent in all our previous TTC33 coIP-LF-MS analyses, suggesting that this time an inactive PP2A was largely purified. Further coIP-WB analyses, in which PP2A subunits were strongly overexpressed, showed that they could co-purify with low amounts of WDR61, co-expressed MBP-TTC33 (Fig. 4b; Supplementary Fig. 2c), and co-expressed EGFP-UNG2 (Supplementary Fig. 2d), which confirmed a PP2A-B55α physical link to TAN.

CoIP-LF-MS analyses of WDR61 interactors revealed SKIc and PAFc subunits^23,24^, and also identified TTC33 and the entire set of TAN factors, fully replicating previous TTC33 coIP-LF-MS analyses (Fig. 4c; Supplementary Fig. 2e). This time again, PHF5A was identified more robustly than other SF3A/B subunits. Curiously, coIP-LF-MS of CCDC97 confirmed association with TTC33, WDR61, SF3A/B, but not with UNG1/2 and PP2A (Fig. 4d; Supplementary Fig. 2f), which we verified in a different cell line by coIP-WB (Fig. 4e). Intrigued, we purified UNG1/2 and found that TTC33, WDR61, and PHF5A associated with each UNG isoform, but CCDC97 and SF3B4 did not (Fig. 4f). Altogether, this suggested that PHF5A could be extracted out of the context of the SF3B complex to independently interact with TTC33 and WDR61. We conclude that TTC33:WDR61:PHF5A engage in mutually exclusive interactions with either SF3A/B-CCDC97 or UNG1/2 (Fig. 4g).

### At the heart of TAN is a trimeric TTC33-associated network core (TANC) complex composed of TTC33, WDR61, and PHF5A

**Fig. 5:**
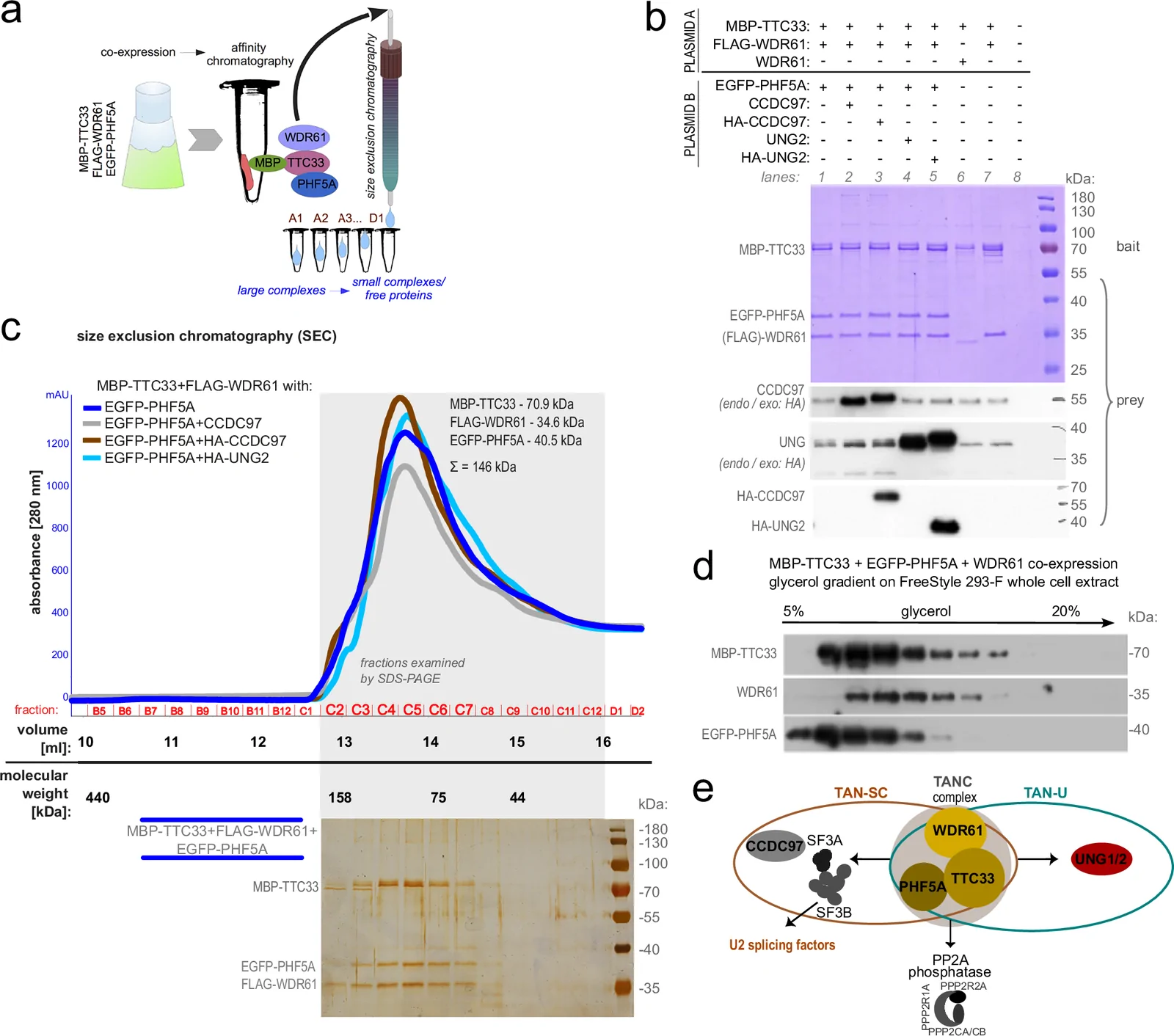
TTC33, WDR61, and PHF5A co-purify in stoichiometric levels and co-migrate in size exclusion chromatography. **a** A scheme illustrating the series of experiments presented in (**b**, **c**). MBP-TTC33, FLAG-WDR61, and EGFP-PHF5A were co-expressed in FreeStyle 293-F cells (in the presence or absence of HA-tagged or non-tagged CCDC97 or UNG2). In each case, the MBP-TTC33 was used as bait to purify the other components by affinity chromatography and the obtained samples were subjected to size exclusion chromatography. **b** Results of coIPs performed using MBP-TTC33 as bait, co-expressed with FLAG-WDR61 and either EGFP-PHF5A alone or in combination with either HA-tagged or non-tagged CCDC97 or UNG2. The purified proteins were resolved in SDS-PAGE gels and analyzed either by Coomassie staining (*top*) or western blot (*bottom*) with antibodies directed against CCDC97, UNG2, or HA-tag. **c** The proteins co-purified with MBP-TTC33 in affinity chromatography (selected samples from the experiment shown in (**b**)—see legend) were subjected to SEC analysis. The graph shows the traces of the 280 nm absorbance in relation to the elution buffer volume of the Superdex™ 200 Increase 10/300 GL column. The column molecular size calibration is also shown in relation to elution volume. The peaks highlighted by the gray box designate the volume range at which proteins were eluted at the highest concentration. Superimposed with the chromatogram, a silver-stained SDS-PAGE analysis of fractions from one of the purifications is shown. **d** Western blot analysis of whole-cell extract of FreeStyle 293-F cells co-expressing MBP-TTC33, EGFP-PHF5A, and WDR61 separated in 5–20% glycerol gradients. **e** The scheme summarizes the main findings of this work regarding TTC33 and its interaction partners. TTC33 forms a trimeric complex with WDR61 and PHF5A, coined TANC. This stable core complex recruits, in a mutually exclusive manner, either UNG1/2, forming TAN-U, or CCDC97 together with the SF3A/B U2-splicing subcomplex, resulting in TAN-SC. The PP2A-B55α phosphatase represents an additional potential interactor of TANC; however, it remains unclear whether it associates with the TAN-U or TAN-SC assemblies, or constitutes a distinct TANC complex. Source data are provided as a Source Data file.

Preliminary experiments showed that WDR61, PHF5A, and CCDC97 co-purified with TTC33 in high (300 mM NaCl) salt conditions, whereas UNG1/2 could only be purified using a buffer containing 100 mM salt (Supplementary Fig. 2g, h). This suggested that the interaction between TTC33, WDR61, and PHF5A was likely strong, allowing us to hypothesize complex formation. To test this, we performed purification from FreeStyle 293-F cells co-expressing MBP-TTC33, FLAG-WDR61, and EGFP-PHF5A (Fig. 5a), and obtained all three proteins in stoichiometric levels (Fig. 5b, *lane 1*). In contrast, CCDC97 and UNG, although successfully overexpressed along with the TTC33:WDR61:PHF5A trimer (Supplementary Fig. 3a), always co-purified in sub-stoichiometric levels and could only be detected by western blot analysis (Fig. 5b, *lanes 2–5*). Similarly, when CCDC97 was used as bait, it never co-purified in stoichiometric levels with co-expressed TTC33, WDR61, and PHF5A (Supplementary Fig. 3b). Therefore, we subjected the purified TTC33:WDR61:PHF5A trimer to size exclusion chromatography (SEC), and observed co-migration of all three proteins in stoichiometric levels (Fig. 5c; Supplementary Fig. 3c).

To confirm TANC formation in vivo, whole-cell extracts from non-transfected FreeStyle 293-F cells were separated on a 5–20% glycerol gradient, showing TTC33 and CCDC97 co-migration (Supplementary Fig. 3d). Not surprisingly, the gradient distribution of WDR61 and PHF5A was consistent with the formation of multiple high- and low-molecular weight complexes, reflecting the many well-studied interactions of both proteins^23,24,27^ (Supplementary Fig. 3d). However, the same analysis on extracts from FreeStyle 293-F cells overexpressing all TANC subunits simultaneously and observed much better co-migration (Fig. 5d). Altogether, these in vitro data showed formation of a TTC33:WDR61:PHF5A complex, which we coined the TTC33-associated network core (TANC; Fig. 5e).

### WDR61 and PHF5A are independently recruited to the TTC33 TPR2-3-4 domain to form the TANC complex

**Fig. 6:**
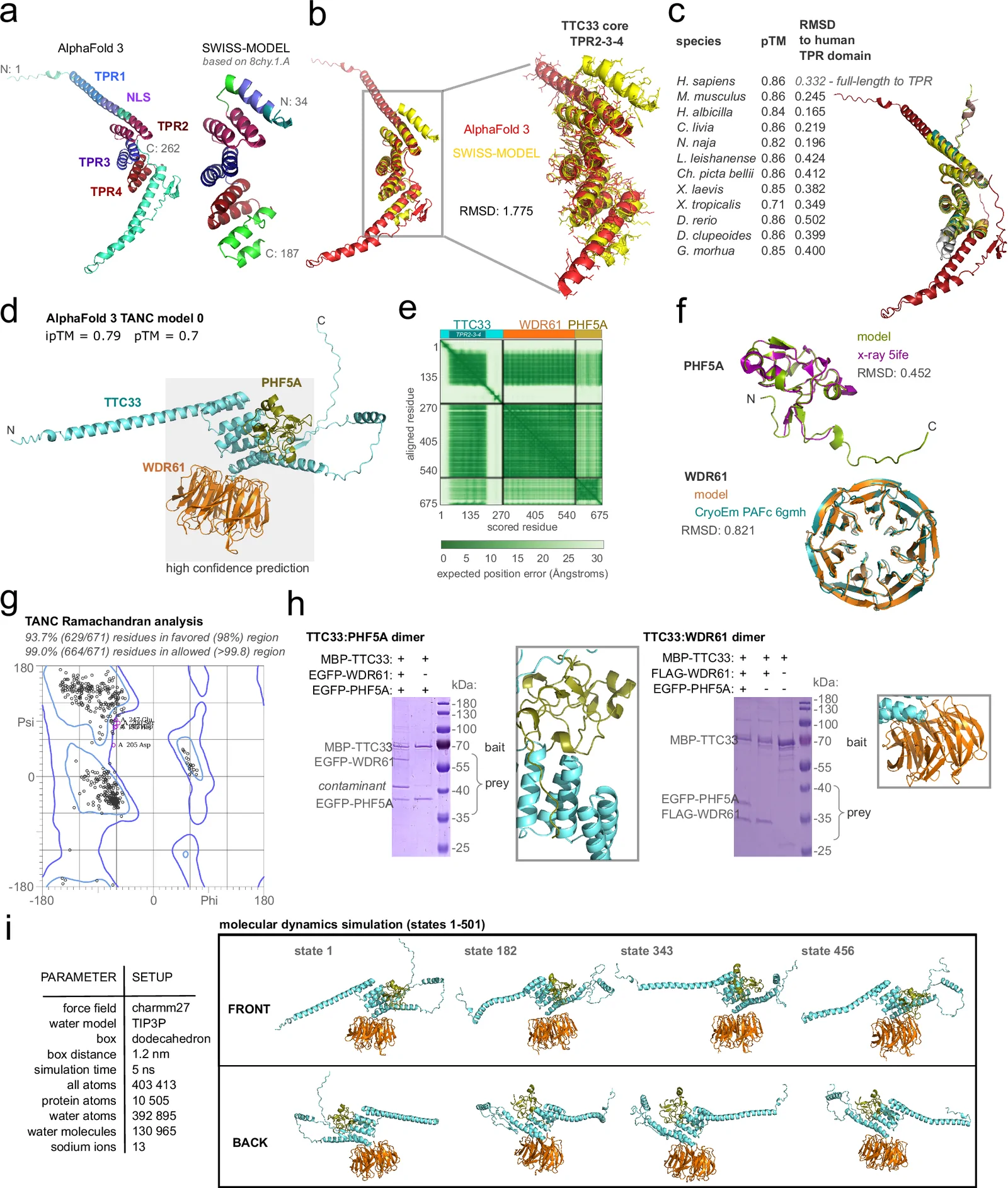
WDR61 and PHF5A are independently recruited to opposite sides of the TTC33 core TPR domain to form the TANC complex. **a** Two TTC33 structural models with color-coded TPR motifs, generated using the AlphaFold 3 algorithm^35^ or the homology-based SWISS-MODEL. The latter is based on the consensus TPR protein as the template (PDB: 8chy^37^; Supplementary Table 1). **b** Structural alignment, with side chains, of the TTC33 models shown in (**a**) (RMSD = 1.775). **c** Alignment of AlphaFold 3 models of TTC33 core TPR domains from 12 vertebrate species analyzed in Fig. 1b, c. The domains correspond to human residues 42–168 (Supplementary Table 2). The table lists pTM and RMSD values relative to the human TPR model. **d** View of AlphaFold 3 TANC model 0 (pTM/ipTM = 0.70/0.79; other angles in Supplementary Fig. 4a). The gray box marks the high-confidence PHF5A/WDR61/TTC33_TPR region (**e** and Supplementary Fig. 4b). Four additional TANC models are compared with model 0 in Supplementary Fig. 4c, d. **e** Expected position error for model in (**d**). **f** Alignment of PHF5A structures modeled by AlphaFold 3 or determined by crystallography (PDB: 5ife^27^). WDR61 structures modeled by AlphaFold 3 or determined by cryo-EM (PDB: 6gmh^23^) are shown below. **g** Ramachandran analysis of AlphaFold 3 TANC model 0 refined using the MolProbity suite (for alignment of models see Supplementary Fig. 4f; RMSD ≈ 0), with the full validation report in Supplementary Fig. 4g. The model contains 93.7% of residues in favored and 99% in allowed conformations. **h** Coomassie-stained SDS–PAGE gels showing proteins co-expressed in FreeStyle 293-F cells and co-purifying with MBP-TTC33. MBP-TTC33 forms stoichiometric trimers with EGFP/FLAG-WDR61 and EGFP-PHF5A, and stoichiometric dimers with either PHF5A (left) or WDR61 (right). Insets show the corresponding TTC33:PHF5A and TTC33:WDR61 interaction surfaces. **i** Table lists molecular dynamics simulations conditions for the TANC model. Adjacent are shown four representative states at different simulation times for replicate 1. Additional structural metrics for replicates 1–3 (RMSD, RMSF, radius of gyration, SASA, energies) are shown in Supplementary Fig. 4h–m. Source data are provided as a Source Data file.

To gain insight into TTC33 architecture, we analyzed an AlphaFold 3^35^ model of the full-length protein (Fig. 6a, *left*; Supplementary Fig. 4a, b, c, d), and a Swiss-Model structure issued from an automated database^36^ that uses homology-based modeling. The latter proposed a model limited to the TTC33 TPR2-3-4 domain, using a consensus TPR protein as reference^37^ (Fig. 6a, *right*; Supplementary Table 1). The amino acid backbone of both models aligned well and displayed differences only around amino acid side-chains (Fig. 6b). Both models proposed a characteristic TPR domain fold, in which 3 pairs of antiparallel helices stacked into a twisted superhelix forming a concave-convex surface^17,18^. AlphaFold 3 confidently modeled such a structure for most TTC33 bony vertebrate orthologs (Fig. 6c, Supplementary Fig. 4e).

Next, we examined a TANC structural model generated by AlphaFold 3, which displayed a reasonable overall quality (Fig. 6d, e; Supplementary Fig. 4a, b). Five models obtained using the algorithm aligned well with one another within the regions containing well-defined motifs, such as the core TPR2-3-4 of TTC33, and entire PHF5A and WDR61 subunits (Supplementary Fig. 4c, d). Indeed, experimentally solved PHF5A and WDR61 structures aligned well with the model^23,24,27^ (Fig. 6f). We further tested the structure quality using the MolProbity suite^38^, which proposed a slight conformation optimization of the second helix of TPR4, and a narrow region of the C-terminus (Supplementary Fig. 4f, g). Ramachandran analysis of this improved model revealed that 93.7% and 99% of residues were in favored and allowed conformations, respectively (Fig. 6g), confirming reasonable quality. The model showed that WDR61 and PHF5A were recruited independently of one another to opposite sides of the TPR2-3-4 domain. This was confirmed experimentally, when we independently purified TTC33:WDR61 or TTC33:PHF5A as stoichiometric dimers (Fig. 6h; see also Fig. 5b, *lanes 6–7*). Finally, to test the stability of the TANC structure, we performed three molecular dynamics simulations, with independent velocity assignments generated during system equilibration (Fig. 6i; Supplementary Fig. 4h–m). PHF5A and WDR61 remained stably associated with their docking sites at TTC33, which aligned with our SEC results (Fig. 5c). The TTC33 C-terminus was most conformationally flexible, both in the initial AlphaFold 3 model and during the simulations, which indicates that this region is likely unstructured. Altogether, we obtained a reasonable structural model of the TTC33:WDR61:PHF5A trimer for further validation, which supported our biochemical results.

### The TTC33:WDR61 and TTC33:PHF5A interaction surfaces are dynamically remodeled and can be disrupted by somatic cancer mutations

**Fig. 7:**
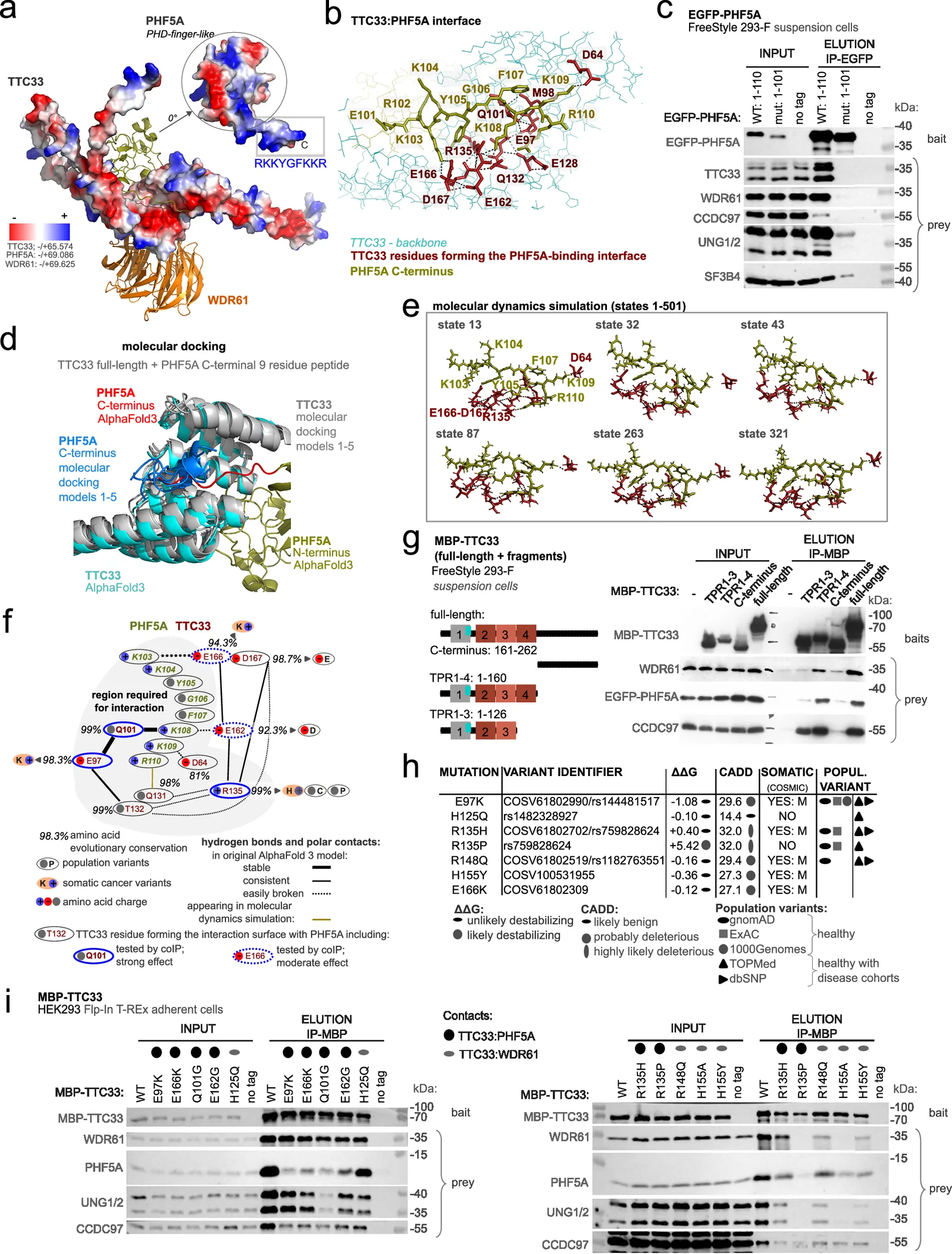
PHF5A positively charged C-terminus binds the negatively charged TTC33 TPR concave. **a** Surface charge representation of TTC33 with PHF5A and WDR61 shown as cartoons. The PHF5A surface charge is shown separately at the same angle. The globular PHD finger-like domain is followed by a positively charged, lysine-rich C-terminus that inserts into the negatively charged TTC33 TPR2-4 concave. **b** A stick model of the TTC33:PHF5A interface. TTC33 is colored cyan with PHF5A-binding residues highlighted in red, whereas gold sticks represent the PHF5A C-terminus. Interacting residues are labeled and polar contacts indicated by black dashed lines. **c** CoIP-WB analysis of TAN factors co-purifying with EGFP-PHF5A wild-type or a C-terminal 9 residue truncation mutant from FreeStyle 293-F cells. **d** The TTC33:PHF5A (cyan:olive) interface from AlphaFold 3 model 0 aligned against five GalaxyWeb docking models^39^. The PHF5A C-terminal peptide (RKKYGFKKR) was docked onto TANC-derived TTC33. **e** Six molecular dynamics (Fig. 6i) states of the PHF5A:TTC33 interaction surface. **f** Schematic depicting polar bonds formed by the PHF5A C-terminus and TTC33 TPR concave in AlphaFold 3 and molecular dynamics. Evolutionary conservation (Fig. 1b) and population/cancer variants are indicated (Supplementary Dataset 1, **h**, and Supplementary Fig. 5b). **g** CoIP-WB analysis of proteins co-purifying with MBP-TTC33 full-length or fragments corresponding to the TPR1-2-3, TPR1-2-3-4, or C-terminus. **h** Table shows TTC33 population and somatic cancer variants affecting residues relevant to WDR61 or PHF5A binding. ΔΔG predicts stability effects (stabilizing <0 <destabilizing), while CADD predicts functional impact. The “SOMATIC (COSMIC)” column indicates COSMIC-listed variants (“YES: M”—predicted moderate impact. The final column reports presence in gnomAD, 1000Genomes, ExAC, TOPMed, or dbSNP; these include population and disease-associated datasets as indicated. All TTC33 mutations are displayed in Supplementary Fig. 5b along with combined SIFT-PolyPhen scores predicting functional impact. **i** CoIP-WB analysis of TAN factors co-purifying with MBP-TTC33 WT or mutants (**h**) from HEK293 cells (HeLa validation in Supplementary Fig. 5c). For H155 and variant-free residues (Q101 and E162), alanine or glycine substitutions were introduced. Potential residue involvement in TTC33:PHF5A or TTC33:WDR61 interaction is marked. Source data are provided as a Source Data file.

According to the TANC model, the TPR2-3-4 concave formed a negatively charged surface, composed of aspartic and glutamic acids, which recruited the electropositive lysine-rich PHF5A C-terminus by means of multiple entangled interactions (Fig. 7a, b). PHF5A is a direct TTC33 interactor, and as such, the most likely factor recruiting the SF3A/B complex. We confronted the SF3B experimental structure^27^ with the TANC model to verify if the assembly of both was possible. Though the globular core of PHF5A is tightly embedded within the SF3B complex, its N- and C-termini are positioned towards the solvent (Supplementary Fig. 5a). Of those, the C-terminus is the longest, and thus the most plausible region, which could enable PHF5A to concomitantly bind to SF3B and TTC33, explaining our LF-MS and coIP-WB results. To inquire about the relevance of PHF5A C-terminus, we produced a truncated mutant, devoid of the terminal 9 smino acids, analogous to the somatic Y105Ifs*64 allele (TCGA-F7-A624-01), lacking the last 6 residues. We found that the mutation strongly decreased PHF5A association with TAN factors. It also reduced the interaction with SF3B4 (Fig. 7c). Molecular docking^39^ of a 9 amino acid peptide corresponding to PHF5A C-terminus into the TTC33 structure invariably positioned it at the TPR domain concave, similarly to the AlphaFold 3 model (Fig. 7d). To further validate the model, we mapped the TTC33 residues engaging into polar contacts with PHF5A in the starting model and during our molecular dynamics simulation (Fig. 7e), and observed that they were scattered mainly around TPR3-4 (aa 93-126 and 127-160) and a narrow region adjacent to TPR4 (aa 161-167). Interactions were formed and broken dynamically during the simulation (Fig. 7f). To verify those predictions, we first fragmented the TTC33 protein (Fig. 7g). An N-terminal fragment containing TPR1-2-3-4, and devoid of the C-terminal 161-262 residues associated with WDR61 and PHF5A similarly to the full-length, but a shortened TPR1-2-3 fragment did not. This narrowed the WDR61/PHF5A-binding region to the TPR domain and identified TPR4 as an essential one. We also designed TTC33 point mutants based on rare population variants and cancer-associated somatic mutations (Fig. 7h; Supplementary Fig. 5b). If no natural variants were reported at a given residue, we substituted glycine or alanine. Those mutants were subjected to coIP-WB analysis to verify TTC33 interfaces predicted by the model in two cell lines (Fig. 7i; Supplementary Fig. 5c). TTC33_Q101G and TTC33_E97K substitutions greatly affected the interaction. Both residues interacted with one another and throughout the simulation consistently formed polar contacts with PHF5A_K108 (Fig. 7f). Mutations of E162 and E166 had a less consistent effect, which showed some contribution from those auxiliary residues, despite negative indications from the fragmentation assay. In either case, the PHF5A:TTC33 interaction was never fully abrogated, which was to be expected, given the width of the interaction surface and the number of potentially compensatory residues. Curiously, we observed that while replacement of R135 with histidine decreased TTC33 binding to PHF5A, mutation to proline completely abolished the association with both PHF5A and WDR61. The R135H mutation has a ΔΔG factor of 0.4, indicating a low overall effect on TTC33 stability. In contrast, R135P has a ΔΔG of 5.42, and is likely destabilizing (Fig. 7h). R135 is located near the center of the TPR4 helix, and given that prolines are helix-breakers, it likely disrupts the TPR fold. Combined with our fragmentation assay, this result indicated that the TTC33 core TPR domain fold is key for recruiting PHF5A^18^.

**Fig. 8:**
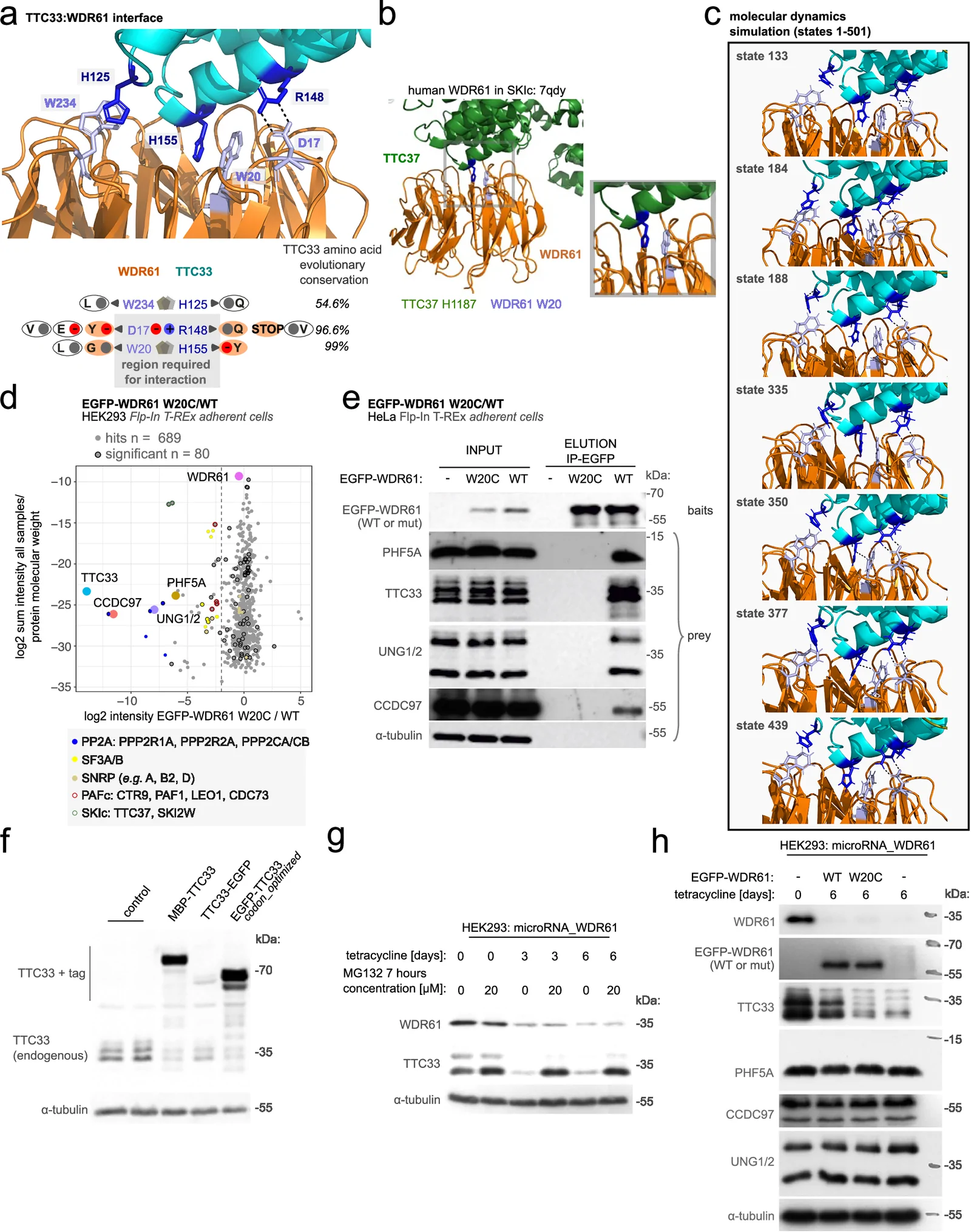
The TTC33:WDR61 interface has two contacts and prevents TTC33 decay. **a** Overview of the TTC33:WDR61 interface. Polar bonds are highlighted using black dashed lines. A schematic representation of the interface is shown below. Evolutionary conservation and population/somatic variants are indicated. **b** Zoom-in on the WDR61:TTC37 interaction surface in the human SKI complex (PDB: 7qdy^24^). **c** Molecular dynamics simulation snapshots of the TTC33:WDR61 interface, with polar bonds marked as dashed black lines. **d** Results of LF-MS analysis of EGFP-WDR61 WT or W20C mutant purified from HEK293 Flp-In T-REx cells. All hits are gray dots; significantly enriched hits above control (Fig. 4c) are circled. SKIc, PAFc subunits, and TAN list factors are color-coded (see legend). **e** CoIP-WB analysis of TAN factors co-purifying with EGFP-WDR61 wild-type and W20C mutant in HeLa cells. As in LF-MS in (**d**), the mutated WDR61 loses interaction with TTC33, PHF5A, UNG1/2, and CCDC97. α-tubulin was used as a negative control. **f** Western blot analysis of TTC33 levels in control HEK293 cells compared to cell lines expressing MBP-TTC33 (used for TANC complex purification and SEC), TTC33-EGFP (used for LF-MS analyses), or EGFP-TTC33_*codon_optimized* (recoded for increased expression). Tagged and endogenous TTC33 levels were assessed, revealing reduced endogenous TTC33 upon tagged protein expression. α-tubulin was used as a loading control. **g** Western blot analysis of WDR61 and TTC33 levels in whole cell extracts. Control (tetracycline-untreated) cells were compared to conditions of 3 or 6 days microRNA WDR61 knock-down, which also led to TTC33 down-regulation. MG132 proteasomal inhibitor at a final concentration of 20 μM was optionally added for 7 h to each condition, resulting in partial rescue of TTC33, but not WDR61, protein levels. **h** Western blot analysis of WDR61, TTC33, and other TAN factors in whole cell extracts. Control (tetracycline-untreated) cells were compared with 6-day WDR61 knock-down conditions, with or without expression of EGFP-WDR61 WT or W20C mutant from constructs insensitive to endogenous WDR61-targeting microRNAs. Rescue of TTC33 levels was abrogated by the W20C mutant compared to wild-type EGFP-WDR61. α-tubulin was used as a loading control. Source data are provided as a Source Data file.

The TTC33:WDR61 interaction surface was less complex. WDR61 docked to the end of the second TPR4 helix in a conformation previously observed for the PAF and SKI complexes (Fig. 8a, b; Supplementary Fig. 6a). In our model, WDR61_D17 formed a polar contact with TTC33_R148. In addition, two pairs of aromatic residues were positioned face-to-face: WDR61_W20 and WDR61_W234 against TTC33_H155 and TTC33_H125, respectively (Fig. 8a), likely mediating non-covalent molecular interactions^40^. Importantly, in the human SKIc structure, the WDR61_W20 residue was similarly positioned against TTC37_H1187^24^ (Fig. 8b). Fragmentation assays confirmed that TTC33 TPR4 was required for association with WDR61 (Fig. 7g; Supplementary Fig. 6b). During the molecular dynamics simulation, the TTC33_R148:D17_WDR61 pair remained stable (Fig. 8c). The conformation of TTC33_H155:W20_WDR61 was also mostly preserved, though occasional flips occurred, which could produce polar contacts between the edges of the aromatic side-chains. Similarly to residues binding to PHF5A, TTC33_R148 and TTC33_H155 are highly evolutionarily conserved (96-99% identity). In contrast, TTC33_H125 is only conserved in 54% of homologs (Fig. 8a), and its conformation in relation to W234_WDR61 displayed great variability (Fig. 8c), indicating that H125 might not significantly contribute to the interaction. To test those in silico predictions, we replaced W20 with cysteine, and observed that WDR61 had lost not only its association with SKIc and PAFc subunits, but also its ability to bind TTC33 and all other TAN factors, which was shown by LF-MS and coIP-WB in three cell lines (Fig. 8d, e; Supplementary Fig. 6c, d). Reciprocal mutation of TTC33_H155 also affected interaction with WDR61, with alanine substitution being more effective compared to the somatic tyrosine variant. The TTC33:WDR61 interaction was also weakened by a somatic TTC33_R148Q variant, but not altered in a TTC33_H125Q mutant (Fig. 7i; Supplementary Fig. 5c). Altogether, those results suggest that the TTC33:WDR61 residue pairs H155:W20 and R148:D17 are most relevant to the interaction.

Aside from the R135P mutations, all somatic and population variants assessed had a low ΔΔG score (Fig. 7h), suggesting a minor impact on overall TTC33 protein structure. Nonetheless, all variants, which strongly impacted TTC33 binding to either PHF5A or WDR61, also decreased its association with UNG1/2 and CCDC97 (Fig. 7i; Supplementary Fig. 5c). Our data suggested that TTC33 TPR domain, and in particular TPR2-3, were sufficient for recruitment of CCDC97 and PPP2R2A (Fig. 7g; Supplementary Fig. 6b). Thus, a strong impact from point mutations located in TPR4 suggests that even slight alterations to the TTC33 core TPR domain structure have a significant impact on the TAN network dynamics. The results of TTC33 fragmentation and point substitutions were summarized in Supplementary Fig. 6e.

### TTC33 is stabilized by WDR61, and its level is regulated by proteolytic degradation

We observed that the expression of tagged TTC33 proteins resulted in down-regulation of endogenous TTC33 (Fig. 8f). The levels of the mRNA arising from the *TTC33* locus were unaltered upon expression of tagged TTC33, excluding regulation by transcription (Supplementary Fig. 6f). In contrast, endogenous TTC33 down-regulation could be partly rescued by addition of a proteasome inhibitor MG132 (Supplementary Fig. 6g). Pursuing the mechanism, we also observed that knock-down of WDR61 concomitantly down-regulated TTC33, and that this could be efficiently rescued by MG132 addition (Fig. 8g). Furthermore, a similar rescue of TTC33 levels in conditions of WDR61 knock-down was also obtained by expression of a wild-type EGFP-WDR61 protein, but not with the W20C mutant version, which does not interact with TTC33 (Fig. 8h). Taken together, we conclude that TTC33 levels are regulated in vivo by proteolytic decay, and WDR61 is key to safeguarding TTC33 from decay, by direct interaction.

Three tagged TTC33 variants were expressed from identical strong CMV promoters yielding identical mRNA levels (Supplementary Fig. 6f). Yet, TTC33-EGFP levels were close to the endogenous TTC33 amount, while MBP-TTC33 and EGFP-TTC33_*codon_optimized* were expressed at much higher levels (Fig. 8f). We compared the interaction profiles of TTC33-EGFP and EGFP-TTC33_*codon_optimized* by coIP-WB (Supplementary Fig. 6h), and observed that TTC33-EGFP precipitated more efficiently WDR61, UNG1/2, and PPP2R2A when compared to bait level. We conclude that the N-terminal tagging impairs TTC33 abundance regulation, possibly due to altered interactions with TAN factors.

### TTC33 is relevant to genomic stability

**Fig. 9:**
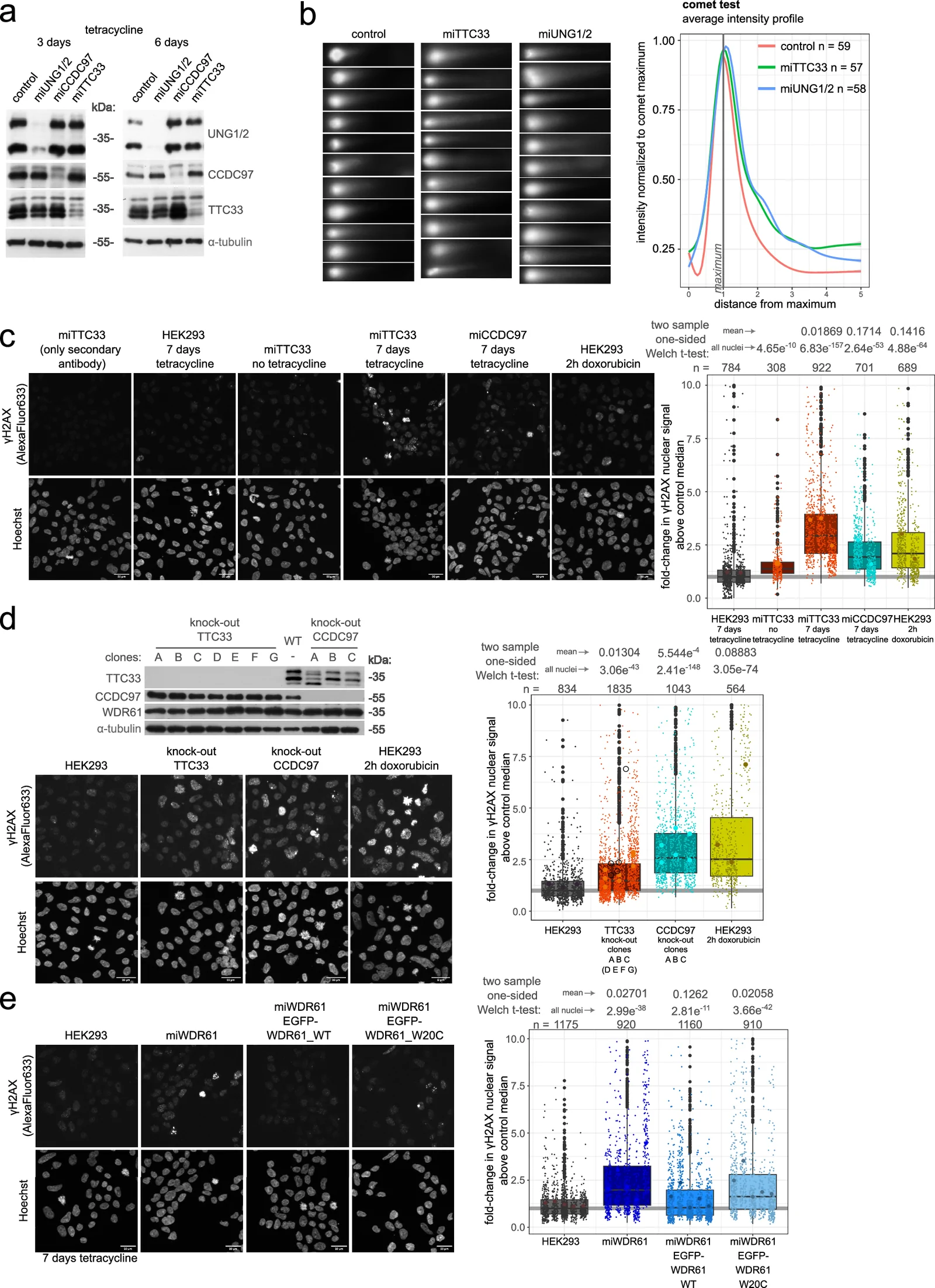
TAN factor loss leads to increased DNA DSBs and nuclear accumulation of γH2AX. **a** Western blot analysis of whole-cell extracts from control HEK293 cells and cells expressing microRNAs against TTC33, CCDC97, or UNG1/2 for 3 or 6 days. **b** The left panel shows representative comet assay images from control HEK293 cells and cells depleted of TTC33 or UNG1/2. The right panel shows DNA intensity profiles normalized to the maximum comet head signal. **c** Analysis of nuclear γH2AX levels by immunofluorescence in cells knocked-down for TTC33 or CCDC97 using microRNAs. Doxorubicin-treated cells (2 h) served as a positive control. Representative images are shown on the left. The graph shows quantitative analysis of the IF data. Small dots represent individual nuclei grouped by biological replicate; larger dots indicate replicate means. Signal intensity was normalized to the control median. Box plots combine data from all replicates (box: median, 25th and 75th percentiles, whiskers: 5th and 95th percentile). “n” indicates the total number of nuclei analyzed across all biological replicates. Two sample one-tailed Welch’s *t*-test results are shown above the graph. **d** Analysis of γH2AX accumulation in TTC33 and CCDC97 knock-out cells by immunofluorescence. Top left western blots show TTC33, CCDC97, and WDR61 levels in whole cell extracts from control HEK293 cells and stable TTC33 or CCDC97 knock-out lines. α-tubulin was used as a loading control. Seven TTC33 and three CCDC97 CRISPR-Cas9 knockout clones (A–G and A–C, respectively) were obtained. Clones A–C were validated by genomic sequencing (Methods; Supplementary Information Appendix). The bottom left part shows representative images. The graph shows the quantitative analysis as in (**c**), with modification. Each nucleus group combines two biological replicates from one clone. The larger dots show the replicate mean. Means for TTC33 clones D-G are shown as black rings. **e** IF analysis of nuclear γH2AX levels in WDR61 microRNA knocked-down cells. Functional rescue was performed with EGFP-WDR61 WT or W20C (characterized in Fig. 8d, e; validated in Fig. 8h). Representative images (left) and quantitative analysis of five biological replicates (right), as in (**c**). Source data are provided as a Source Data file.

To uncover the TTC33 function, we adopted the strategy of testing if its depletion replicated any of the known phenotypes of its interacting partners. The PP2A-B55α, PHF5A, and UNG1/2 factors are directly linked to DNA repair^4,9,41,42^. We initiated our analysis with a comet test that revealed that chromatin isolated from either TTC33- or UNG1/2-knocked-down cell line was markedly more fragmented compared to control (Fig. 9a,b). This indicated that loss of either protein leads to an increase in DNA DSBs, which was previously shown for UNG knock-down^43^.

The H2AX histone variant phosphorylated at serine 139 (γH2AX) is an in vivo marker of DNA DSBs. We analyzed the nuclear levels of γH2AX by immunofluorescence in control compared to cells depleted of TTC33, CCDC97, or PPP2R2A. As a positive control, we assessed cells treated with doxorubicin, which is known to induce DNA DSBs^44^. Resonating with previous reports, siRNA-mediated depletion of PPP2R2A increased the nuclear γH2AX levels^9^ (Supplementary Fig. 7a). Similarly, knock-down of TTC33 or CCDC97 using microRNAs also led to an increase in nuclear γH2AX (Fig. 9c). Notably, TTC33 overexpression also resulted in γH2AX accumulation, indicating that both reduced and elevated TTC33 levels perturb DNA damage homeostasis (Supplementary Fig. 7b). Importantly, this phenotype was confirmed independently in HEK293 cell lines knocked-out of TTC33 or CCDC97 genes using CRISPR-Cas9 (Fig. 9d), and in cells depleted of TTC33 using siRNAs (Supplementary Fig. 7c). Evaluation of this phenotype using multiple protein-reduction methods confirmed its specificity. In WDR61 knock-down, which concomitantly down-regulates TTC33 (Fig. 8g, h), γH2AX was similarly increased (Fig. 9e). Importantly, this phenotype was markedly reduced to control levels by ectopic expression of wild-type EGFP-WDR61, but could not be rescued by the EGFP-WDR61_W20C mutant (Fig. 9e). Altogether, an increase in γH2AX levels is a phenotype associated with the loss of TAN factors in general and is likely a consequence of increased DNA DSB.

Depletion of UNG1/2 leads to accumulation of DNA DSBs through activation of alternative, less efficient, uracil excision pathways^5^. In the in vitro glycosylase assays, we observed only a minor impact of TTC33 or CCDC97 down-regulation on uracil excision, while UNG1/2 depletion or up-regulation strongly impacted the reaction rate, as expected (Supplementary Fig. 7d, e). This indicated that the mechanism of DNA DSB formation in TTC33-/CCDC97-deleted cells is not strongly linked to UNG1/2 catalysis.

**Fig. 10:**
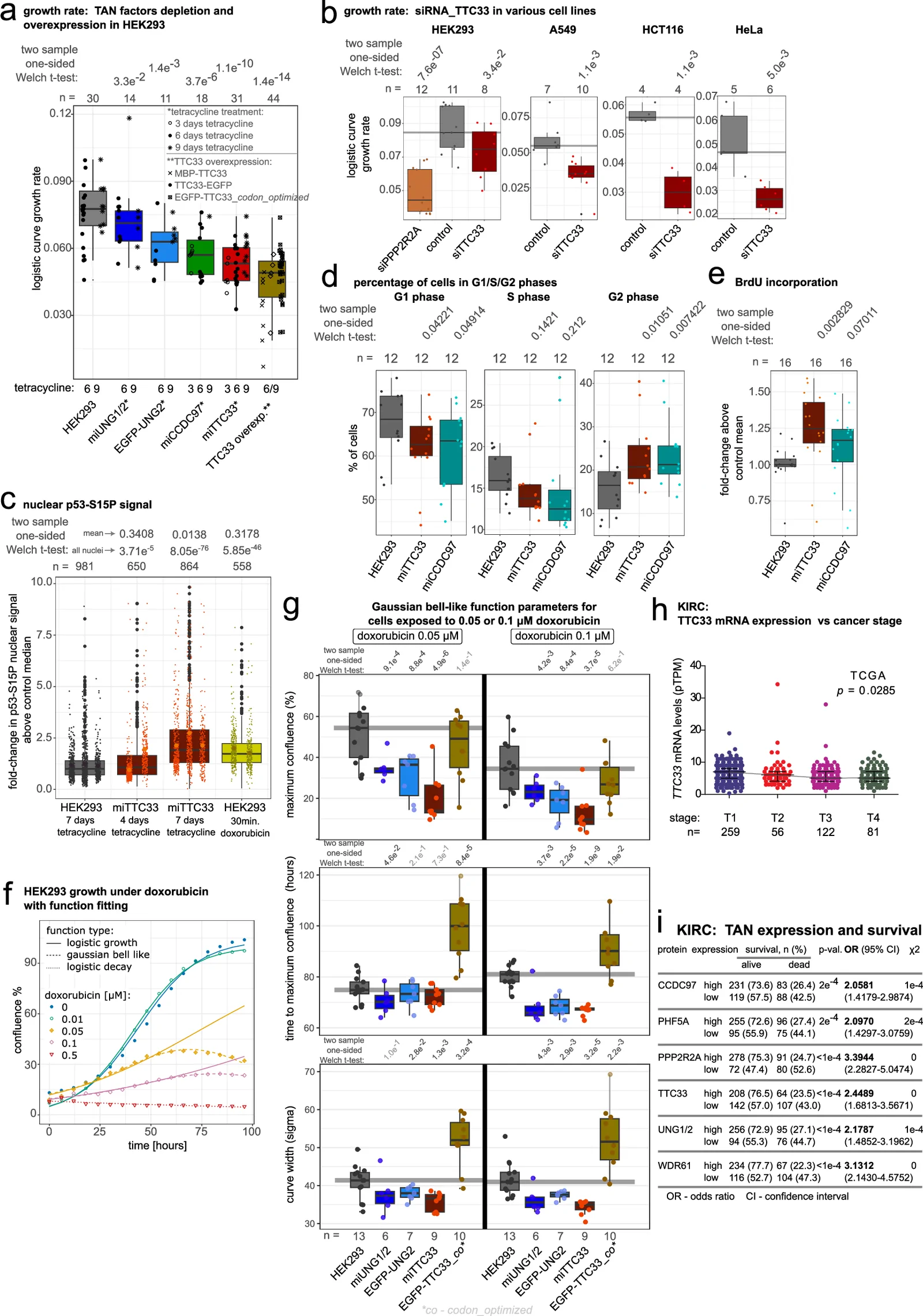
TTC33 loss causes cell cycle arrest and sensitizes to genotoxic agents. **a** IncuCyte S3 growth analysis of cells with altered TAN factor levels (see legend). Logistic growth rates are compared (method shown in Supplementary Fig. 8a). Box plots show median, 25th/75th percentiles, and whiskers indicate 5th/95th percentiles. Biological replicates (n) and one-tailed Welch *t*-test results are indicated. **b** Logistic growth rate analysis as in (**a**) for TTC33 siRNA-depleted HEK293, A549, HeLa, and HCT116 cells or HEK293 knocked-down for PPP2R2A. **c** Quantification of nuclear p53-S15P levels in control and TTC33-microRNA-depleted HEK293 cells (as in Fig. 9c; images: Supplementary Fig. 8b). **d** Box plots show G1, S, and G2 phase distributions in control and TTC33/CCDC97-depleted HEK293 cells (statistics as in (**a**); two biological replicates comprising six technical replicates each are shown). **e** Box plot showing BrdU incorporation in TTC33/CCDC97-depleted cells compared to control (two biological replicates comprising of eight technical replicates). Statistics as in (**a**). **f** Experimental HEK293 confluence for cells exposed to 0, 0.01, 0.05, 0.1, or 0.5 μM doxorubicin is shown as dots. Lines show fitted logistic growth/decay or Gaussian functions. Control and low-dose conditions followed logistic growth, 0.5 μM logistic decay, and intermediate doses Gaussian behavior (Supplementary Fig. 9c) after initial logistic growth (≤65 h). More cell lines in Supplementary Fig. 9d. **g** Box plot showing Gaussian bell-like parameters describing cell growth under 0.1 and 0.05 μM doxorubicin. Statistics as in (**a**). **h** TTC33 protein transcripts per million (pTM) in KIRC tumor samples according to cancer stage. Dots represent patient samples; boxes indicate median, connected by gray lines, and quartiles. Statistical testing using Fisher exact test. Data were obtained from the TCGA Research Network^49^ (https://www.cancer.gov/tcga) via the Human Protein Atlas. **i** Associations between TTC33, CCDC97, WDR61, PHF5A, UNG1/2, and PPP2R2A mRNA expression and overall survival in KIRC patients. Stratification by expression level and survival status was assessed using Fisher’s exact test. Odds ratios (OR) with 95% confidence intervals are indicated (other metrics in Supplementary Fig. 9e, data in Supplementary Dataset 4). Source data are provided as a Source Data file.

Increased genotoxic stress often impacts proliferation. We evaluated growth rates using logistic modeling of experimental confluence data (Supplementary Fig. 8a; Supplementary Dataset 3). Proliferation of HEK293 cells overexpressing TTC33 or depleted of TTC33 using si- or microRNAs alike was decreased. A similar, but more pronounced, effect was observed in cancer-derived HeLa, A549, and HCT116 cells. Similarly, but to a lower extent growth of cells depleted of PPP2R2A or UNG1/2 and overexpressing UNG2 was also affected (Fig. 10a, b). This showed that TAN factors are required for efficient cell proliferation. To further validate this, we examined nuclear p53 phosphorylation at serine 15 (p53-S15P), which marks a cell cycle arrest in anticipation of DNA repair^12^. Accordingly, si-/microRNA-mediated TTC33 knock-down resulted in nuclear accumulation of p53-S15P in HEK293 and A549 cells (Fig. 10c, Supplementary Fig. 8b–d). This phenotype was shared with depletion of UNG1/2, and with TTC33 or UNG1/2 overexpressions (Supplementary Fig. 8e). Thus, the patterns of nuclear accumulation of p53-S15P supported the proliferation rate results. We further inquired about the reason, and observed that in HEK293 cells depleted of TTC33 or CCDC97, the number of cells in G1 phase was decreased with a concomitant increase in the proportion of cells in G2 (Fig. 10d). This suggested that TTC33 loss results in a delay in exit from G2 to mitosis.

Additional observations supported the notion of increased genotoxic stress in TTC33-/CCDC97-depleted cells. First, both cell lines displayed elevated BrdU incorporation (Fig. 10e). This assay primarily measures DNA replication, and is usually positively correlated with proliferation levels. However, in quiescent cells or in conditions of slowed proliferation, such as observed for TTC33 or CCDC97 depletions, increased BrdU incorporation rather indicates ongoing DNA repair^45,46^. Second, impaired DNA repair is associated with increased sensitivity to ROS, which was previously shown in UNG1/2-depleted cells^47,48^. We briefly exposed cells to hydrogen peroxide and evaluated their growth rate during the recovery phase. While the proliferative phase of control cells was extended by 11 h due to ROS, it was twice as long as for cells knocked-down or overexpressing either TTC33 or UNG1/2 (Supplementary Fig. 9a, b). Thus, either condition reduced tolerance to ROS, likely because it exacerbated the genomic instability already induced by alterations in TTC33 or UNG1/2 abundance. To test for tolerance of DNA DSBs, we evaluated sensitivity to doxorubicin. The drug impaired control cell line growth in a dose-dependent manner (Fig. 10f; Supplementary Fig. 9c, d). At low concentration, which did not affect control cell growth, proliferation of cells depleted of or overexpressing TTC33 or UNG2 was slightly inhibited (Supplementary Fig. 9e). At higher concentrations of doxorubicin, cell confluence exhibited a growth–plateau–decline profile (Fig. 10f; Supplementary Fig. 9c, d). TTC33-depleted cells displayed reduced growth capacity, reaching a lower maximal confluence and doing so earlier and over a shorter growth period compared with control cells. This phenotype was shared by both UNG1/2-depleted and UNG2 overexpressing cells. TTC33 overexpressing cells reached a similar maximal confluence as control cells, but did so later and over a more prolonged growth period (Fig. 10g), suggesting enhanced tolerance rather than resistance to doxorubicin. Collectively, these findings suggest that appropriate TTC33 levels are required for an efficient cellular response to genotoxic stress.

TAN depletion in HEK293 cells increased genomic instability, suggesting that its loss may enhance mutagenesis and accelerate carcinogenesis in vivo. Analysis of RNA-sequencing datasets from The Cancer Genome Atlas^49^, revealed that *TTC33* expression decreased with cancer stage in patients with KIRC (Fig.10h). Regression analysis further revealed that higher expression of all TAN factors was positively correlated with roughly 2-times longer overall survival and, for most TAN factors, improved 5-year survival rate among patients with KIRC (Fig. 10i; Supplementary Fig. 9f; Supplementary Dataset 4). This suggests that TAN factor expression may collectively serve as a potential prognostic indicator in KIRC.

## Discussion

Roughly 9% of human proteins are poorly characterized^1^, which evidences the significant gap in our understanding of cell physiology. Here, we delineated the physical links and phenotypic traits of two interacting uncharacterized human proteins: TTC33 and CCDC97.

To define the list of TTC33 interactors, we developed the comparative label-free mass spectrometry (cLF-MS) pipeline, rooted in the quantitative evaluation of protein enrichment using the MaxQuant algorithm^20^. Mass spectrometry is highly sensitive, which is useful for the identification of weak interactions, but also generates many false-positives that obscure the identification of stable, functionally relevant interactions. We showed that the excessive sensitivity of MS can be minimized by simply comparing TTC33 interaction profiles issued from several cell lines and applying various filtering conditions. We used HEK293, FreeStyle 293-F, and HeLa, which differ by growth conditions (suspension-adherent), background (embryonic-cancer), and bait protein expression system (genomic integration under inducible promoter-transient expression). This approach shortlisted several possible stable TTC33 partners: WDR61, uncharacterized CCDC97, UNG1/2, PP2A-B55α phosphatase, and SF3B-PHF5A, which were validated by coIP-WB. All TAN proteins have a broad tissue expression pattern, and share the nucleus as a possible common sub-cellular compartment. Co-purification in various cell lines shows that the TAN network is likely functionally relevant to many human cell types.

Reciprocal purifications coupled to LF-MS and western blot provided further insight into the TAN network architecture. Among WDR61 interactors we found all the TTC33 partners. In contrast, UNG1/2 and CCDC97 purifications hinted towards a possible mutually exclusive partnership of both proteins with TTC33, WDR61, and PHF5A. It also showed that the remainder of the SF3B complex is linked exclusively to CCDC97, suggesting that PHF5A can interact with TTC33 and WDR61 outside the context of SF3B. This led us to hypothesize the formation of a trimeric complex by TTC33, WDR61, and PHF5A. We successfully purified this trimer in stoichiometric levels, and showed its stable co-migration in size exclusion chromatography. Altogether, we propose that TTC33:WDR61:PHF5A (TAN core/TANC) is a platform that mostly recruits either UNG1/2 or CCDC97 and SF3B. It is unclear whether PP2A-B55α is yet another minor, mutually exclusive interactor. Likely so, as TTC33 fragmentation showed that both CCDC97 and PPP2R2A bind to the TPR domain. However, when overexpressed, PPP2R2A can co-precipitate with UNG1/2, suggesting that the phosphatase might be linked to the glycosylase.

We modeled the TANC structure, confronted it with known WDR61- and PHF5A-containing structures, and validated by fragmentation and mutational analyses. High-confidence TTC33 modeling was only possible in the TPR2-3-4 region, which was responsible for PHF5A, WDR61, CCDC97, and PP2A-B55α binding. In light of future follow-up structural studies, removal of the TTC33 N-terminal helical region and the unstructured C-terminus is warranted to produce a more globular and easily purifiable protein. While the TTC33:WDR61 interaction surface was seamlessly validated by reciprocal mutations, the TTC33:PHF5A interface proved to be more complex. Though the PHF5A C-terminus, which mediated the interaction with TTC33, was unstructured, its removal decreased the protein association not only with TTC33 and TAN factors, but also with SF3B4. This was surprising as only the globular PHF5A core interacts with the SF3B complex^27^. Combining analysis of the original AlphaFold 3 model and its evolution in the molecular dynamics simulation, we identified residues forming the TTC33:PHF5A interaction surface, and successfully validated their relevance. Regardless of whether the mutations affected WDR61 or PHF5A binding, they also impacted TTC33 association with UNG and CCDC97. This suggested that neither UNG nor CCDC97 is recruited to a short linear motif, but to a complex surface, which is easily distorted by small structural changes. Many of the variants we tested, previously reported as somatic cancer SNPs of uncertain significance, were shown here to disrupt normal cellular protein networks.

Functionally, our main goal was to associate TTC33 with a biological process, by describing phenotypes arising after its loss. We hypothesized that the TTC33 function should be shared with at least one of its partners. Though both WDR61 and PHF5A impact transcription and RNA metabolism, we downplayed this immediate hypothesis. First, we defined the WDR61 role in regard to TTC33 as being limited to stabilizing the TPR-containing protein. Second, PHF5A is an emerging oncoprotein, which impacts carcinogenesis in ways extending beyond alternative splicing^50^. PHF5A together with p400 chaperone mediates deposition of γH2AX to promote NHEJ at the antibody locus during class switch recombination^42^ (CSR). UNG1/2 and PP2A-B55α participate in DNA repair, with the former initiating BER^4^, and the latter dephosphorylating γH2AX following DNA damage repair^9,41^. Considering that many TAN subunits function in DNA repair, we investigated genomic stability in TTC33. Chromatin in nuclei isolated from TTC33-depleted cells was clearly more fragmented, and nuclear γH2AX levels, which represent the most direct marker of DNA DSBs, were increased in TTC33, CCDC97, PPP2R2A, and WDR61 knocked-down cells. Thus, in general, TAN-depleted cells display genomic instability, but the source of the damage is unclear.

TTC33 and CCDC97 knock-downs produced other phenotypes associated with genotoxic stress. The primary effect was a growth delay observed in four cell lines, particularly in cancer-derived cells, accompanied by increased p53-S15P levels. Rather than apoptosis, the most likely reason for slower growth is an increased duration of the G2 cell cycle phase. A cell cycle arrest in anticipation of DNA damage repair is a primary mechanism preventing propagation of mutations in the cell population. Increased BrdU incorporation likely indicates ongoing DNA synthesis associated with repair. Finally, TTC33 levels affected the cellular response to ROS and doxorubicin. Increased sensitivity to ROS was a phenotype shared with UNG1/2 depletions^47,48^. ROS oxidizes DNA, lipids, and proteins, which is poorly tolerated by cells already experiencing genomic instability, regardless of its source. In contrast, doxorubicin specifically induces DNA DSBs^44^, and the altered response to this drug more directly implicates TTC33 in cellular adaptation to DNA damage. At cytotoxic levels, commonly used in cancer therapies, TTC33 loss increased sensitivity, while its overexpression enabled longer cell survival and proliferation, albeit at a lower rate, suggesting a degree of cellular adaptation.

DNA DSBs accumulate spontaneously in TAN-deficient cells, though the mechanism is unclear. Neither TTC33 nor CCDC97 impacts UNG1/2 catalysis; however, the glycosylase also performs scaffolding roles. UNG1 associates with PRDX3 to physically coat the mitochondrial DNA, protecting it from ROS^48^. Though UNG2 glycosylase activity is crucial for initiation of immunoglobulin locus CSR and somatic hypermutation, the enzyme’s scaffolding role was also proposed^51–53^. Interestingly, in the CSR context, PHF5A was suggested to facilitate NHEJ^42^. Altogether, TAN might directly support repair of DNA DSBs in steps downstream or independent of UNG1/2 catalysis. This was a function ascribed to PP2A-B55α in the context of response to irradiation^9^. In TAN-deficient cells, DNA DSBs may arise from co-transcriptional processes, which might put the TTC33 association with SF3A/B in the right context. More specifically, helix unwinding during transcription initiation and elongation causes tension relaxed by DNA-cleaving topoisomerases. In addition, DNA:RNA hybrids also increase the rate of homologous recombination^54^. In sum, future efforts to define the TAN function will aim to narrow down these hypotheses.

## Methods

### Cell lines and general growth conditions

All adherent cell lines used in this study, HeLa Flp-In T-REx^55^, HEK293 Flp-In T-REx cells (R78007; Thermo Fisher Scientific), A549 (ATCC CCL-185), and HCT116 (ATCC CCL-247), were grown in Dulbecco’s Modified Eagle Medium (DMEM) with high glucose (392-0415; VWR) supplemented with 10% of Fetal Bovine Serum (FBS) (A5256701 or 10270106; LifeTechnologies) at 37 °C with 5% CO_2_. The FreeStyle™ 293-F suspension cells (R79007; Thermo Fisher Scientific) were grown in FreeStyle™ 293 Expression Medium (12338001; Thermo Fisher Scientific) at 37 °C with 8% CO_2_ and continuous shaking (120 rpm).

### Cloning and tagging of cDNA encoding proteins of interest for expression in adherent HEK293 or HeLa Flp-In T-REx cells or FreeStyle 293-F cells

Reverse transcription was performed on RNA extracted from HEK293 Flp-In T-REx cells using SuperScript IV (18090050; Life Technologies), following the manufacturer’s instructions (see below for the protocol of RNA extraction and reverse transcription). The resulting cDNA was used as a template for amplification of open reading frames (ORFs) of interest using Phusion™ High-Fidelity DNA Polymerase (F530L; Life Technologies) and gene-specific oligonucleotides bearing a 21 nt-long overhangs complementary to the acceptor plasmid. For the list of oligonucleotides, see Supplementary Dataset 5. The primer overhangs were designed for cloning using the SLiC method^19^ into the pKK vector series bearing N-terminal or C-terminal MBP or EGFP tags separated from the ORF with a TEV cleavage site. To construct vectors expressing one protein, pKK acceptor plasmids were digested with *Bsh*TI (*Age*I; A/CCGGT) and *Nhe*I (G/CTAGC). Plasmid and insert were then mixed and treated with T4 DNA polymerase (M0203L; New England Biolabs) for 2 min. at room temperature, and the reaction mixture was immediately used to transform chemically competent *Escherichia coli* MH1 bacteria (*araD lacX74 galU hsdR hsdM rpsL*).

In case of the constructs for the expression of EGFP-WDR61 WT and W20C insensitive to microRNAs directed against endogenous WDR61, and EGFP-TTC33_*codon_optimized*, the inserts encompassing ORFs were ordered from Thermo Fisher Scientific GeneArt service. The ORFs were flanked by sequences corresponding to the desired pKK or pKK-BI16 vectors. The inserts were excised using *Bsh*TI and *Nhe*I restriction enzymes, and purified following separation in the agarose gel, using the Gel-Out kit (023-250; A&A Biotechnology), and then ligated with equally processed acceptor plasmid using T4 DNA ligase (EL0012; Thermo Fisher Scientific). The sequences of synthetic inserts with overhangs, as ordered from GeneArt, are given in Supplementary Dataset 5.

Derivatives of the pKK-BI16 vector with bi-directional promoter allowing for the simultaneous expression of two different proteins (for TANC purification and SEC analyses) were constructed using traditional restriction enzyme and T4 DNA ligase (EL0012; Thermo Fisher Scientific)-dependent cloning, wherein the two ORF-coding inserts were introduced between *Bsh*TI and *Nhe*I (insert no. 1) or *Mun*I/*Mlu*I (insert no. 2) cleavage sites.

Irrespective of the cloning method, selected clones were screened for the presence of the insert by digestion with appropriate restriction enzymes and agarose gel electrophoresis of the products. Plasmids with correct digestion patterns were then subjected to Sanger sequencing to ensure a perfect match with the reference sequence. The constructs obtained in this study are listed in Supplementary Dataset 5.

### Obtaining constructs for microRNA-mediated protein knock-down in adherent HEK293 Flp-In T-REx cells

We established several HEK293 Flp-In T-REx cell lines with conditional knock-down of proteins of interest using microRNAs (miRNAs), which were designed using the BLOCK-iT™ RNAi Designer (https://rnaidesigner.thermofisher.com/rnaiexpress/). The criteria used were 35-55% GC content and miRNA mapping to an open reading frame. To increase knock-down efficiency, one construct contained three miRNAs mapping to various mRNA sequence regions. The miRNA cassette sequences are listed in Supplementary Dataset 5. The constructs were ordered from Thermo Fisher Scientific GeneArt service as inserts in *E. coli* plasmids, which were then re-cloned into pKK-RNAi-nuc-CHERRYmiR-EGFP-TEV plasmid (a derivative of pcDNA5/FRT/TO vector^19^). The biologically relevant region is preceded by nucCherry ORF and flanked by *Bsp*TI (*Afl*II; C/TTAAG) and *Not*I (GC/GGCCGC) restriction sites, which were used for cloning. The derivatives of pKK-RNAi-nuc-CHERRYmiR-EGFP-TEV containing miRNAs against proteins of interest were then transfected into HEK293 Flp-In T-REx cells to produce stable cell lines (refer to the subsequent section for the protocol of cell line generation).

### Generation of stable HEK293/HeLa Flp-In T-REx cell lines for microRNA or protein expression

Stable HEK293 or HeLa Flp-In T-REx cell lines were generated as described previously by Szczęsny et al.^19^. Briefly, the day before transfection, HEK293 cells were plated on a 6-well plate to around 50% confluence (1 × 10^6^ cells), whereas HeLa cells were plated to approximately 40% confluence (8 × 10^5^ cells) using DMEM supplemented with 10% FBS at 37 °C with 5% CO_2_. The next day, 300 ng of plasmid bearing either microRNA or cloned cDNA was mixed with 1 μg of pOG44 helper plasmid and 250 μl of Opti-MEM™ I Reduced Serum Medium, GlutaMAX™ Supplement (51985-026; Gibco), supplemented with 2 µl of TransIT-2020 Transfection Reagent (MIR 5400; Mirus Bio), and incubated at room temperature for 20 min. prior to adding the mixture dropwise to the cells. A negative control (non-transfected cells) was included in parallel to ensure the specific selection of the cell lines. Following transfection, the cells were grown for 24 h before transfer to a Ø60 mm dish, and then cultivated in DMEM + FBS with hygromycin B (10687010; LifeTechnologies) and blasticidin S (J67216.XF;LifeTechnologies). The final concentration of blasticidin S was 8 µg/ml, whereas hygromycin B was added to the final concentration of 40 µg/ml for HEK293 and 140 µg/ml for HeLa. After 6–7 days of initial selection of antibiotic-resistant cells, the medium was replaced with a fresh one, containing both antibiotics at increased concentration, that is 10 µg/ml blasticidin S and 50 or 175 µg/ml hygromycin B for HEK293 and HeLa, respectively. Cells were further grown until single antibiotic-resistant colonies appeared, which usually occurred approximately 4 weeks later. Expanded into larger plate formats, the obtained polyclonal cell lines were tested for protein expression or knock-down efficiency using western blot analysis. Established stable cell lines were stored in liquid nitrogen after freezing in growth media containing 10% DMSO using Mr. Frosty container. The list of plasmids in Supplementary Dataset 5 details whether a respective stable cell line was generated using each particular plasmid.

### Generation of HEK293 Flp-In T-REx TTC33- and CCDC97-knock-out (KO) cell lines using

#### CRISPR-Cas9

The protocol described by Ran et al.^56^ was generally used, with minor modifications, to establish stable HEK293 cell lines knocked-out for TTC33 or CCDC97. Three sgRNAs were used in either case (TTC33/CCDC97 sgRNA 1–3). For each sgRNA, the respective construct was prepared by cloning annealed and T4 PNK (M0201S; New England Biolabs)-pre-phosphorylated top-strand (ts) and bottom-strand (bs) synthetic DNA oligonucleotides (for sequences refer to the Supplementary Dataset 5) into *Bbs*I (*Bpi*I) restriction sites of pSpCas9(BB)-2A-Puro (PX459) V2.0 plasmid (Addgene #62988) using T4 DNA ligase. The ligation mixture was transformed into *E. coli* MH1 strain. Inserts in recombinant vectors were verified by Sanger sequencing using the standard U6_fwd primer.

5 × 10^5^ cells per well of a 6-well plate were transfected with an equimolar (200 ng of each) mixture of respective three pSpCas9(BB)-2A-Puro plasmids using Lipofectamine 3000 (L3000001; Thermo Fisher Scientific). Briefly, 600 ng plasmid DNA (total) and 1.2 μl P3000 reagent in 100 μl of Opti-MEM were combined with 1.5 μl Lipofectamine 3000 in 100 μl of Opti-MEM, incubated for 10 min. at room temperature, and added dropwise to the cells. 48 h following transfection, the culture medium was replaced with fresh DMEM + 10% FBS containing puromycin (1.5 μg/ml; ant-pr-1; InvivoGen). Selection of puromycin-resistant cell pools in the antibiotic-containing medium was continued for 6 days, with a change of the medium to a fresh one after 72 h. ~90% confluent cells were detached by trypsinization, diluted with fresh medium, and half of the cell suspension was used for the preparation of protein samples, which were then analyzed by western blot using anti-TTC33 and anti-CCDC97 antibodies to confirm successful CRISPR-Cas9-mediated gene knock-outs in the obtained cell pools (polyclonal KO cell lines). The remainder of the cell suspension was passed through the cell strainer (732-2758; VWR) to thoroughly dissociate the potential cell clumps. Following cell counting using the Thoma chamber, the suspension was serially diluted in the medium containing puromycin aiming at 12 ml of medium containing 60 cells (final concentration of 0.5 cell/100 μl), which were transferred to an Ø100 mm dish and then plated onto 96-well plate using a multichannel pipette (100 μl per single well) for clonal selection. The entire clonal selection in 96-well plate format lasted for 14–17 days, and during this time the medium was changed to a fresh one every 3 days. After the first 3 days, the wells were inspected every day for the presence of cells. During the next 4 days, formation of single colonies, representing monoclonal KO cell lines, began. The wells appearing to contain only one colony were marked, and the growth of cells in the 96-well plate was continued until reaching approximately 90% confluence. Monoclonal cell lines thus obtained were then propagated by sequential expansion into bigger plate formats (96-well plate → 24-well plate → 12-well plate → 6-well plate → Ø60 mm dish → Ø100 mm dish). When two Ø100 mm dishes were available for the given monoclonal KO cell line, one of them was used for the preparation of cell aliquots in medium supplemented with 10% DMSO, which were frozen using a Mr. Frosty container and long-term stored in liquid nitrogen until needed in subsequent experiments. In turn, the cells from the second dish were utilized for the preparation of protein and DNA samples. First, the whole cell protein extracts were separated in SDS-PAGE and analyzed by western blot to validate the absence of proteins of interest (TTC33 or CCDC97) resulting from gene knock-out. Second, genomic DNA (isolated using Genomic Mini kit from A&A Biotechnology, cat. no. 116-250, according to the manufacturer’s instructions) served as a template in control PCR amplifications performed using various primer pairs flanking sites targeted by sgRNAs (see Supplementary Dataset 5 for a full list of primers used in PCR-based validations). For selected clones, the obtained amplicons were cloned into pCR^TM^ -II-Topo^®^ vector using Zero Blunt^®^ TOPO^®^ PCR Cloning Kit (450245; Invitrogen), and the inserts were subjected to Sanger sequencing using M13 (-20) universal primer. Analysis of the sequencing results provided ultimate proof of successful gene knock-out. The detailed overview of such verification of TTC33 or CCDC97 CRISPR-Cas9-mediated KOs, at both the protein and the genomic DNA levels, in several monoclonal cell lines obtained for each target gene is presented in the Appendix deposited at Mendeley data (doi: 10.17632/p822vhnbdj.1).

### Protein or miRNA expression in stable HEK293 or HeLa Flp-In T-REx cell lines

HEK293 or HeLa Flp-In T-REx stable cell lines expressing tagged protein and/or microRNAs placed under the control of a tetracycline-inducible promoter were grown to near-confluence at conditions described above. Knock-down of the protein of interest was performed by incubation of the established cell lines with tetracycline added to a final concentration of 100 ng/ml for a minimum of 3 and a maximum of 9 days, depending on the experiment (see relevant figures and caption for details). Down-regulation of gene expression was evaluated by western blot analysis. Expression of the protein of interest was performed by incubation of the established cell lines with tetracycline added to a final concentration of 100 ng/ml for a minimum of 3 and a maximum of 7 days (see relevant figure and caption for details), and protein production was similarly tested by western blot.

### Knock-down of TTC33 or PPP2R2A using siRNAs

HEK293 Flp-In T-REx, HeLa Flp-In T-REx, A549, or HCT116 cells were plated on a Ø60 mm dish in DMEM high glucose with 10% FBS, and at the time of transfection cells were at 40–50% confluence in 3.3 ml of media. For one transfection, 5 μl of 20 μM siRNA stock was diluted in 350 μl of Opti-MEM, and mixed. Next, 7 μl of Lipofectamine™ RNAiMAX Transfection Reagent (13778100 or 13778150; LifeTechnologies) was diluted in 350 μl of Opti-MEM, and mixed. The Opti-MEM-diluted siRNAs and Lipofectamine™ RNAiMAX dilutions were then combined and left to settle at room temperature for 15 min. The transfection mixture was dispensed evenly on the culture by gentle pipetting. Cells were cultured for 3 days, and if need be were transfected again. The catalog numbers of siRNAs against PPP2R2A and Stealth siRNAs against TTC33, which were used in this study, are given in Supplementary Dataset 5.

### FreeStyle 293-F transfection for protein overexpression

To express proteins in FreeStyle™ 293-F, the cells were grown in 20/30 ml volumes in dedicated flasks to 1–2 million cells per ml and transfected with a total of 20/30 μg of plasmid (1:1 ratio μg of plasmid to ml of culture). The transfection mix components were prepared by combining 0.5/0.75 ml of FreeStyle™ 293-F Expression Medium with the plasmid and another 0.5/0.75 ml of media with 80/120 μl of PEI transfection reagent (25 mM HEPES pH 7.5, 150 mM NaCl, 1 mg/ml Polyethylenimine [PEI], Linear, MW 25000, Transfection Grade [PEI 25 K™] CAS Number: 9002-98-6, 26913-06-4, cat. no. 23966-100 from Polysciences). The two 0.5/0.75 ml aliquots were then combined, vortexed, and incubated for 15 min. before applying to cells. Protein expression was induced for 3 days before cell harvesting.

### RNA extraction

The hot acid phenol method was used to extract total RNA. Medium was discarded from the culture dish and the cells were washed with PBS. Cells were scraped and resuspended in PBS. After centrifugation, the supernatant was removed and the cells were resuspended in 400 μl of Aqua-Rotiphenol (ROTH-A980.1), and thoroughly mixed. Next, 400 μl of TES buffer (10 mM Tris-HCl, pH 7.5, 5 mM EDTA, 1% SDS) was added. Samples were incubated with shaking for 45 min. at 65 °C. After centrifugation at 20,000 × *g*, 4 °C for 10 min., the supernatant was transferred into a fresh tube and 400 μl of Aqua-Rotiphenol solution. The samples were again incubated with shaking at 65 °C for 20 min. and then centrifuged as above. The supernatant was transferred into a fresh tube and 400 μl of chloroform (C2432; Sigma) was added to remove the traces of phenol. The samples were briefly vortexed at room temperature and centrifuged as above. The supernatant was transferred into a fresh tube and RNA was precipitated using 45 μl of 2 M LiCl and 1 ml of 95% ethanol for 24 h at −80 °C. Precipitated RNA was centrifuged for 25 min. at 4 °C, washed with 400 μl of 80% ethanol, and, after removing the supernatant, dried at 37 °C. Pellets were resuspended in nuclease-free water, and the concentration was measured using a Nanodrop spectrophotometer.

### Reverse transcription coupled to qPCR

10 μg of total RNA was subjected to DNase I (M0303S; New England BioLabs) treatment in dedicated buffer (B0303S; New England BioLabs) for 30 min. at 37 °C. RNA was next extracted briefly with acid phenol, cleared with chloroform, and precipitated (see “RNA extraction” section above). SuperScript IV reverse transcriptase (18090050; LifeTechnologies) was used to prepare cDNA. The cDNA synthesis was primed using oligo-dT_(18)_ (final concentration 5 μM), random hexamers (final concentration 2.5 ng/μl), 0.5 mM dNTPs, and 1 μg of total DNase I-treated RNA. Briefly, the RNA was denatured in the presence of oligo-dT_(18)_, random hexamers, and dNTP mix for 5 min. at 65 °C and then placed on ice for 2 min. Thus denatured and primed RNA was mixed with SuperScript IV reaction buffer mix containing DTT, RiboLock RNase inhibitor (EO 0382; LifeTechnologies), and enzyme, according to the manufacturer’s instructions. The reaction mix was kept at room temperature (20–21 °C) for 20 min. and then shifted for 40 min. to 50 °C. The reaction was stopped by incubating the samples for 10 min. at 80 °C. qPCR reactions were prepared using SYBR qPCR mix (2008–1000; A&A Biotechnology) and run on the LightCycler 480 instrument (Roche). Ct values were extracted using the 2nd derivative maximum method. Oligonucleotides used are listed in the Supplementary Dataset 5.

### Whole-cell extract preparation for SDS-PAGE analysis

Cell pellets derived from 6-well plates or Ø60 mm dishes were washed with PBS. Next, they were resuspended in (depending on cell number) 40–80 μl of urea buffer (50 mM Tris-HCl pH 7.5, 9 M urea, 150 mM 2-mercaptoethanol). Samples were then incubated on ice for 20 min. and centrifuged for 10 min. at 10,000 × *g* at 4 °C. The supernatant was then diluted with 20–40 μl of Laemmli buffer (150 mM Tris-HCl pH 6.8, 300 mM DTT, 6% SDS, 0.3% bromophenol blue, 30% glycerol) and stored at −20 °C for SDS-PAGE analysis. Alternatively, cell pellets were resuspended in 40–80 μl of RIPA buffer (50 mM Tris-HCl pH 8.0, 150 mM NaCl, 1% NP-40, 0.5% sodium deoxycholate, 0.1% SDS, supplemented with 1 mM PMSF and protease inhibitors cocktail—2 μM pepstatin A, 0.6 μM leupeptin, 2 mM benzamidine, 2 μg/ml chymostatin). Then, samples were sonicated using a Bioruptor XL sonicator (Diagenode) at a high power setting, by applying 20 cycles consisting of 15 s pulse:15 s break. Lysed samples were centrifuged for 15 min. at 20,000 × *g* at 4 °C. Supernatants were mixed with Laemmli buffer and analyzed by SDS-PAGE as above.

### Immunoprecipitation of proteins and protein complexes

Each sample for protein immunoprecipitation (IP) experiments, which was later subjected to western blot analysis, was obtained from one Ø100 mm dish with confluent HEK293 or HeLa Flp-In T-REx cells (8–10 million cells). For samples that were later subjected to mass spectrometry analyses, one Ø145 mm dish was used (up to approximately 20 million cells). For IP experiments performed using transiently transfected FreeStyle 293-F, cell pellets obtained from 20 to 30 ml suspension culture at 1–2 million cells per ml, were utilized. Cells were collected into 15 ml tubes and the pellets were washed with 4 ml of ice-cold sterile PBS. Cell pellets were frozen in liquid nitrogen and stored at −80 °C until use.

To prepare a whole-cell protein extract, frozen pellets were resuspended in 1.7 ml of IP buffer (20 mM HEPES-KOH pH 7.1, 100 mM NaCl, 3 mM MgCl_2_, 10% glycerol, 0.5% NP-40, 0.1 mM DTT supplemented with 1 mM PMSF and protease inhibitors cocktail; see above). Samples were optionally treated with RNase A (0.2 mg/ml; 10109142001; Sigma). When needed, the NaCl concentration in the IP buffer was increased to 300 mM, which is specifically indicated for each experiment (Supplementary Fig. 2g, h). Samples were incubated on ice for 30 min. and sonicated using a Bioruptor XL sonicator (Diagenode) at high power setting, by applying 30 cycles consisting of 30 s pulse:30 s break. Lysed samples were centrifuged for 15 min. at 20,000 × *g* at 4 °C, the supernatant was collected, and protein concentration was quantified using Bradford Assay (5000006; BioRad). Typically, concentrations from 2–5 μg/μl were achieved and if protein levels between samples were significantly different, the concentrations were appropriately adjusted.

Prior to the IP experiment, beads were pre-washed 3 times with 1 ml of the IP buffer on the rotating wheel at 4 °C for 5 min. Typically, 200 μl of amylose resin (E8021L; New England Biolabs), 20 μl of anti-MBP magnetic beads (SAE0218; Sigma), or 50 μl of home-made GFP-trap magnetic beads (prepared by coupling of recombinant GFP nanobody to the CNBr-activated SepFast^TM^ MAG 4HF [310201-10G; BioToolomics] via the recommended protocol), or 20 μl of GFP-Trap^®^ Magnetic Agarose (gtma-10; Chromotek) were used per sample. Whole-cell extracts were applied to the washed resin and incubated for 90 min. on a rotating wheel at 4 °C. Past this time, the supernatants were removed, either by applying samples with anti-MBP/GFP-trap magnetic beads to a magnetic stand or by centrifuging amylose resin for 3 min. at 300 × *g*. Beads were then washed 3 times with the IP buffer for 5 min. on a rotating wheel at 4 °C.

To elute proteins directly for western blot, the supernatant was carefully removed, and the beads were incubated with 50–70 μl of Laemmli buffer for 7 min. at 95 °C with occasional vortexing. Beads were discarded and the supernatant (eluate) was collected to be loaded onto SDS-PAGE gels. Typically, 0.6% of input (whole-cell extract) and 15% or 5% of IP elution respectively derived from anti-MBP/GFP-trap or amylose resin, were utilized. The input concentrations typically ranged from 3 to 6 μg/μl.

Beads with bound proteins intended for use in the analyses employing mass spectrometry were divided. 10% of the sample was subjected to elution with Laemmli buffer as described above and then to western blot analysis or Coomassie-/silver-stained after electrophoresis in SDS-PAGE gel. The remaining 90% was processed in a way that facilitated subsequent MS analyses. In the case of anti-MBP or GFP-trap resin, the IP buffer was simply removed and the dried beads were frozen in liquid nitrogen and stored at −80 °C prior to further processing (see below). If a sepharose-based amylose resin was used, the elution was performed by adding 100–200 μl of the IP buffer containing 0.1 M maltose (M9171; Sigma) and incubating the samples at 4 °C for 10 min. The supernatant was collected, brought to 1 ml volume, and precipitated by adding 330 μl of PRM reagent (0.05 mM pyrogallol red, 0.16 mM sodium molybdate dihydrate, 1.0 mM sodium oxalate, 50 mM succinic acid, pH 2.5 set using HCl). The samples were thoroughly vortexed and incubated for 20 min. at room temperature. The tubes were centrifuged at 20,000 × *g* for 20 min., the supernatant was discarded, and the protein pellets were air-dried.

### Processing of samples for mass spectrometry analysis after immunoprecipitation

Mass spectrometry analysis was performed at the Mass Spectrometry Laboratory at the Institute of Biochemistry and Biophysics PAS. Several datasets, represented in Fig. 2b, Fig. 4a, c, d, and Fig. 8d were generated, and below we specify the protocols used for each dataset presented in the manuscript. Raw datasets are deposited at PRIDE (accession numbers: PXD059336, PXD059337, PXD066861, PXD043138 and PXD043098; see Supplementary Dataset 2 and “Data availability” section). The analysis criteria are specified below and the lists of significantly enriched proteins are given in Supplementary Dataset 2.

To generate datasets for the analysis of TTC33-EGFP, EGFP-WDR61_WT, and EGFP-WDR61_W20C purified from adherent HEK293 Flp-In T-REx cells, dried anti-GFP magnetic resin beads were suspended in 50 μl of 100 mM triethylammonium bicarbonate (TEAB). Proteins were reduced with tris(2-carboxyethyl)phosphine (TCEP), cysteine residues were blocked with methyl methanethiosulfonate (MMTS), and proteins were digested overnight with sequencing-grade trypsin (Promega, cat. no. V5111), as described previously by Kloska et al.^57^, Peptide digests were purified using Oasis HLB 96-well plates (Waters), vacuum-dried, dissolved in 2% acetonitrile/0.1% trifluoroacetic acid, and analyzed by online nanoLC-MS/MS.

Peptides were analyzed using a nanoACQUITY UPLC system (Waters) coupled online to a Q Exactive Orbitrap mass spectrometer (Thermo Fisher Scientific). Peptides were loaded onto a nanoACQUITY UPLC trapping column using 0.1% formic acid in water as the loading solvent and subsequently separated on a nanoACQUITY UPLC BEH C18 analytical column, 75 μm inner diameter × 250 mm length (Waters). Solvent A was 0.1% formic acid in water and solvent B was 0.1% formic acid in acetonitrile. The analytical column was maintained at 35 °C and the sample compartment at 4 °C. Peptides were trapped for 6 min at 6 μl/min in 100% solvent A and separated at a flow rate of 250 nl/min using the following gradient: 1% B initially, 5% B at 1 min, 35% B at 160 min, 90% B from 163 to 167 min, followed by re-equilibration to 1% B. The total LC-MS/MS method duration was 182 min. Blank/washing runs were used to monitor and minimize carry-over between sample injections. Followed by a high-organic wash and column re-equilibration. Blank or washing runs were included between sample injections to monitor and minimize carry-over.

The Q Exactive instrument was operated in positive ion mode using a data-dependent acquisition method with a full MS/dd-MS2 TopN scheme. Full MS scans were acquired in the Orbitrap over the m/z range of 300–2000 at a resolution of 70,000, with an AGC target of 1 × 10^6^ and a maximum injection time of 60 ms. MS/MS spectra were acquired at a resolution of 17,500, with an AGC target of 5 × 10^5^ and a maximum injection time of 300 ms. The 12 most intense precursor ions were selected for fragmentation in each acquisition cycle using an isolation window of 3.0 m/z. Fragmentation was performed by higher-energy collisional dissociation with a normalized collision energy of 27. The fixed first mass was set to 100 m/z. The minimum AGC target was 6.5 × 10^3^, the precursor intensity threshold was 2.2 × 10^4^, and dynamic exclusion was set to 30 s. Unassigned, singly charged, and >8 charged precursor ions were excluded from fragmentation, and isotope exclusion was enabled.

Raw data were searched using MaxQuant 2.0.3.0^20^ with the Andromeda search engine against a human UniProt reference proteome FASTA file downloaded in April 2022. Trypsin/P was selected as the digestion enzyme and up to two missed cleavages were allowed. The minimum peptide length was set to seven amino acids. Cysteine methylthiolation was set as a fixed modification, whereas methionine oxidation and protein N-terminal acetylation were set as variable modifications and were included in protein quantification. The main search precursor mass tolerance was 4.5 ppm and the fragment ion tolerance for FTMS MS/MS spectra was 20 ppm. The maximum peptide mass was set to 4600 Da. Decoy sequences were generated by sequence reversal and common contaminants were included in the search.

Peptide-spectrum match and protein-level false discovery rates were controlled at 1%. Label-free quantification was performed in MaxQuant using razor peptides. Match between runs was enabled with a matching time window of 0.7 min and an alignment time window of 20 min. Second peptides were enabled, whereas dependent peptide search and iBAQ quantification were disabled. LFQ comparisons required MS/MS evidence. Reverse hits, potential contaminants, and proteins identified only by site were removed before downstream analysis.

The IP-eluate after purification of MBP-CCDC97, MBP-PPP2R2A, or MBP-TTC33 derived from adherent HEK293 Flp-In T-REx cells was precipitated with pyrogallol and dried. Buffer was removed from the magnetic beads used to purify TTC33-EGFP from suspension FreeStyle 293-F or HeLa Flp-In T-REx cells. The dried protein pellet or magnetic beads were resuspended in 50 or 100 µl of 100 mM ammonium bicarbonate buffer, pH 8.5, with 5 mM TCEP, respectively. To block reduced cysteines, 200 mM MMTS at a final concentration of 10 mM was added, and the samples were incubated at room temperature for 10 min. The digestion of proteins was performed overnight at 37 °C with 1 or 2 µg of trypsin (Promega), respectively. After digestion, peptide solutions were acidified by adding formic acid to 0.1% concentration and dried. Peptide pellets obtained from MBP-CCDC97 derived from adherent HEK293 Flp-In T-REx cells and TTC33-EGFP purified from suspension FreeStyle 293-F cells were re-suspended in 20 µl extraction buffer (0.1% trifluoroacetic acid and 2% acetonitrile), and peptide samples were processed using the single-pot solid-phase-enhanced sample preparation (SP3) method with some modifications^58^. The magnetic beads mix was prepared by combining equal parts of Sera-Mag Carboxyl hydrophilic and hydrophobic particles (65152105050250 and 45152105050250, Cytiva). After triple washing with MS-grade water, the beads mix was resuspended to a working concentration of 10 µg/µl. 36 µl of the prepared bead mix was added to each sample, along with 100% acetonitrile to a final concentration of 80%. Peptides bound to beads were washed three times with 1 ml of 100% acetonitrile. Beads were dried, and clean peptides were eluted with 200 µl of 2% acetonitrile. Samples were dried again and resuspended in 50 µl of 0.1% formic acid. Half of the samples were analyzed using an LC-MS system consisting of an Evosep One (Evosep Biosystems) coupled with an Orbitrap Exploris 480 mass spectrometer (Thermo Fisher Scientific). MaxQuant/Andromeda suite (version 2.5) was used for protein identification and quantification with parameters similar to those described above. A reference human database derived from Uniprot was used for protein identification (one sequence per gene, versions 2025_02 and 2025_03, 20,659 and 20,675 sequences, respectively).

Peptides were analyzed using an LC-MS system consisting of an Evosep One (Evosep Biosystems) coupled with an Orbitrap Exploris 480 mass spectrometer (Thermo Fisher Scientific). For each sample, half of the peptides were loaded onto disposable Evotip Pure C18 trap columns (Evosep Biosystems) according to the manufacturer’s instructions. The bound peptides were washed three times with 100 µl and then covered with 300 µl of solvent A (0.1% formic acid in water). Chromatographic separation was achieved using a preformed 88-min gradient on an analytical column (ReproSil Saphir C18, 1.5 µm beads, 150 µm ID, 15 cm length, Bruker Daltonics) at a flow rate of 220 nl/min. Data acquisition was conducted in positive ion mode using a data-dependent acquisition method with the following settings: MS1 scans were obtained at a resolution of 60,000, with a normalized AGC target of 300%, an automatic maximum injection time, and a scan range of 300–1600 m/z. MS2 scans were carried out at a resolution of 15,000, with a standard normalized AGC target and automatic maximum injection time. The top 40 precursor ions were selected for MS/MS analysis within an isolation window of 1.6 m/z. A dynamic exclusion period of 20 seconds was applied, with a mass tolerance of ±10 ppm and a precursor intensity threshold of 5e3. Fragmentation was performed using higher-energy collisional dissociation (HCD) with a normalized collision energy of 30%. The source parameters included a spray voltage of 2.1 kV, a funnel RF level of 40, and a heated capillary temperature set at 275 °C.

### Numerical analysis of MaxQuant mass spectrometry data

The raw MaxQuant protein intensity data were further analyzed using RStudio (Version 1.2.5033 or Version 2024.04.2 + 764). Data was pre-filtered to remove hits identified mainly in the negative control, as those were clearly contaminants. To perform this filtering, a hit had to be identified in the bait-containing sample in 100–50% of samples. A higher threshold was set for datasets containing 3 or fewer such samples, whereas relaxed criteria applied to larger datasets. The missing values were replaced by randomized data, which was at least one order of magnitude lower than the weakest hit in each sample. Next, the biological replicate samples were normalized to the sum of protein intensities in the given replicate, thus allowing for quantitative comparison between biological replicates in each experiment. To identify significantly enriched proteins, the average intensity across tagged samples and the average intensity across non-tagged control samples were calculated, and enrichment was expressed as the log2 ratio of these mean intensities:

1 $$log 2\left(\frac{\frac{\left(\sum {INTENSIT}{Y}_{{TaggedSample}}\right)}{{numbe}{r}_{{TaggedSamples}}}}{\frac{\left(\sum {INTENSIT}{Y}_{{NonTaggedSample}}\right)}{{numbe}{r}_{{NonTaggedSamples}}}}\right)$$

As significantly enriched proteins were selected those, which had a log2 fold-change higher than the dataset 0.6 or 0.8 quantile, giving two sets of enriched factors. The obtained list was additionally curated to ensure that more than one peptide for the q0.8 set or at least one peptide for the q0.6 set was identified for each significantly enriched protein. The number of peptides identified in the control was limited to 10 on average. To produce a list of potential TTC33 interactors, the proteins identified as significantly enriched using the q0.6 threshold were merged, giving 1334 hits. Of those, 311 were also identified using the relaxed criteria. This shorter list was subjected to gene ontology analysis using the https://geneontology.org/ suite with ‘biological process’ setting. For each of the 1334 hits a score is assigned, which indicates in how many datasets it was identified using the q0.6 or q0.8 criteria. Given that four datasets were produced for the assessment of TTC33 interactions, the maximum score for each criterion is 4. A sum of both scores (max = 8) is also given as an ultimate tool to explore this dataset.

The code written for the analysis is deposited as text files at Mendeley (doi: 10.17632/p822vhnbdj.1). Pre-filtered raw high-throughput mass spectrometry datasets, along with imputation statistics, comparative analysis of TTC33 interactors, and gene ontology analysis are listed in Supplementary Dataset 2.

### Western blotting

Proteins were separated in 10-15% SDS-PAGE gels in Tris-glycine-SDS buffer (25 mM Tris-HCl, 192 mM glycine, 0.1% SDS, pH 8.3) and then transferred to Protran nitrocellulose western blotting membranes (GE10600001; Amersham) using the semi-dry transfer (17 V, 1 h 25 min.) in semi-dry transfer buffer (48 mM Tris base, 39 mM glycine, 20% methanol, 0.0375% SDS) and Semi-Dry Blot Apparatus (1703940; Bio-Rad). To verify protein transfer efficiency, the blots were stained with Ponceau stain (0.1% Ponceau S in 5% acetic acid) for 5 min. and washed 5 times with deionized water. Afterwards, the blots were blocked in 5% skimmed milk in TBS-T (20 mM Tris-HCl pH 7.5, 150 mM NaCl, 0.05% Tween-20) and probed overnight with relevant primary antibodies in the analogous milk solution on a rocking platform at 4 °C. After 4 times washing in TBS-T (10 min. for each wash), blots were incubated with appropriate secondary antibodies conjugated with HRP in 5% milk in TBS-T for 1–3 h, washed as above, and developed with ECL substrate (1705061; Clarity Western ECL, BioRad) using X-ray film (Fuji Medical X-ray film Super RX) or an iBright apparatus (Thermo Fisher Scientific). All antibodies used in experiments presented in this work are listed in the Supplementary Dataset 5.

To analyze protein levels in whole cell extracts equal amounts of protein were loaded on the gel to ensure reproducibility. To analyze coIP experiments typically 0.5% of input sample was compared to 10 or 20 % of elution. Multiple TAN factors are close in molecular weight (TTC33, WDR61, UNG1/2, and CCDC97), necessitating running multiple gels to analyze all factors. All measures were taken to prepare each blot in equal conditions, the efficiency of protein transfer was assessed each time by Ponceau S staining and by evaluation of loading control levels (α-tubulin or GAPDH). Blots not meeting transfer standards by Ponceau S staining were discarded. Blots in Figures are structured to show the sample processing control at the top (e.g., bait for coIP or blot directly testing the knock-down or overexpression in the whole cell extract), followed by assessment of other factors, and typically ending with the loading control (α-tubulin or GAPDH). Uncropped and unprocessed blots are deposited in Mendeley Data (doi: 10.17632/p822vhnbdj.1), and deposited as Source Data files.

### Coomassie and silver-staining of SDS-PAGE gels

To visualize proteins separated in SDS-PAGE with Coomassie, the gel was pre-boiled in distilled water in the microwave for 1 min., soaked in Coomassie stain (0.025% Coomassie Brilliant Blue R-250, 7.5% acetic acid, 40% methanol), boiled in the microwave for 10 s, and then incubated with gentle shaking for 15 min. The gel was destained by incubation with the destaining solution (10% ethanol and 4% acetic acid).

To silver-stain proteins, the gel was first fixed in 50% methanol, 12% glacial acetic acid for a minimum of 1 h. Then, the gel was washed with 50% methanol for 20 min. and twice with water for 20 min. The gel was subsequently immersed in a sensitizing solution (0.02% sodium thiosulfate) for 1 min. and rinsed twice in water for 1 min. The gel was incubated for 20–30 min. in a staining solution (0.1% silver nitrate stored at 4 °C), washed twice in water for 1 min., and eventually immersed in a developing solution (0.04% formalin and 2% sodium carbonate). The reaction was stopped by transferring the gel to 5% acetic acid when the desired intensity was reached.

### Analysis of protein migration in glycerol gradients

Pellets of FreeStyle™ 293-F suspension cells (either non-transfected or transfected with combination of plasmids encoding proteins of interest; 100 ml of culture was used for each experiment) were resuspended in 0.5 ml of lysis buffer (20 mM HEPES-KOH pH 7.1, 100 mM NaCl, 3 mM MgCl_2_, 5% glycerol, 0.5% NP-40, supplemented with 1 mM PMSF and protease inhibitors cocktail), and incubated for 30 min. at 4 °C in the presence of 0.1 mg/ml RNase A. Lysates were then sonicated at 4 °C in a BioRuptor sonicator (Diagenode) using 30 cycles of 30 s pulse:30 s break at a high setting, and centrifuged at 15,000 × *g* for 15 min. at 4 °C. Cleared extracts were loaded onto 13 ml continuous 5–20% glycerol gradients containing 20 mM HEPES-KOH pH 7.1, 100 mM NaCl, 3 mM MgCl_2_, 1 mM PMSF, and protease inhibitors, and centrifuged at 170,000 × *g* (max speed or 33,000 rpm) in an SW 41 Ti Swinging-Bucket Rotor (Beckman Coulter) for 16 h at 4 °C. 1 ml fractions were collected manually from the top to the bottom of the gradient and analyzed by western blot as described above. An equal volume (12 μl out of 1 ml) of each fraction mixed with Laemmli buffer was analyzed in SDS-PAGE.

### Size exclusion chromatography (SEC)

Pellets of FreeStyle™ 293-F suspension cells transfected with single plasmids encoding proteins of interest or their combinations (200 ml of culture was used for each experiment) were resuspended in 13 ml of lysis buffer (20 mM HEPES-KOH pH 7.1, 100 mM NaCl, 3 mM MgCl_2_, 10% glycerol, 0.5% NP-40, 1 mM DTT, supplemented with 1 mM PMSF and protease inhibitors cocktail), and incubated for 30 min. on ice, and then for 30 min. at 4 °C on a rotating wheel. Lysates were centrifuged at 15,000 × *g* for 15 min. at 4 °C. Cleared extracts were subjected to affinity purification on amylose resin, performed essentially as described above for IP experiments, but using 500 μl of beads. Following protein binding to the resin, the supernatant was removed and the beads were washed three times with the lysis buffer. Native elution was performed using 1 ml of the lysis buffer supplemented with 20 mM maltose. Eluates were filtered from the resin using Poly-Prep® Chromatography Columns (7311550; BioRad) and then separated in the SEC buffer (20 mM HEPES-KOH pH 7.1, 100 mM NaCl, 3 mM MgCl_2_) by gel filtration on Superdex^TM^ 200 Increase 10/300 GL column (28-9909-44; GE Healthcare) using ÄKTA purifier FPLC system (GE Healthcare). 300 μl fractions were automatically collected during the run and analyzed by silver-stained SDS-PAGE or western blot.

### Fluorescence microscopy analysis

To perform immunolocalization of proteins of interest, HEK293 Flp-In T-REx cell lines were grown on coverslips in 6-well plates. Glass coverslips were sterilized and coated under the laminar hood with 0.01% (w/v) water solution of poly-L-lysine hydrobromide (P1274; Sigma) for 30 min. at room temperature. The solution was removed and the coverslips were dried before seeding cell cultures.

For immunolocalization of EGFP-tagged proteins, cells were grown with tetracycline (100 ng/ml) for 24 h to allow the expression of proteins of interest. At 50% confluence, the cells were washed three times with 1 ml of PBS and fixed with 4% (v/v) formaldehyde solution in PBS for 20 min. at room temperature under the fume hood. Cells were then washed 3 times with 1 ml of PBS and permeabilized with 0.25% (v/v) Triton X-100 in PBS for 15 min. at room temperature. Then, the supernatant was discarded and the coverslips were washed three times with 1 ml of PBS and incubated with 1 ml PBS containing 3% (w/v) BSA at room temperature for 30 min. BSA was removed by washing cells three times with 1 ml PBS. Hoechst dye (H3570; Invitrogen) in PBS was added (1:5 000) and incubated for 5 min. After 3 washes with 1 ml PBS, the coverslips were dried and mounted with Abberior Mount Solid Antifade (MM-2013-2X15ML; Abberior) overnight in the dark at room temperature.

For the immunolocalization of endogenous proteins, the cells were permeabilized and blocked with BSA as described above. Then, 50 μl of the primary antibody (anti-PPP2R2A, anti-γH2AX, or anti-p53-S15P; Supplementary Dataset 5) in 3% (w/v) BSA in PBS was added and topped with another coverslip for 1 h at room temperature or overnight at 4 °C. The top coverslip was removed by three washes with 1 ml PBS. The coverslips were then incubated with secondary antibodies conjugated with Alexa Fluor (Supplementary Dataset 5) in the same manner as above. To remove the top coverslip, a Hoechst dye in PBS was added (1:5 000) and incubated for 5 min. After 3 washes with 1 ml PBS, the coverslips were dried and mounted as above.

Microscope slides used to test subcellular localization of TTC33, CCDC97, or PP2A subunits were imaged on a FluoView 1000 confocal microscope (Olympus) using a PlanApo N 60×, NA 1.4 oil objective (Olympus). Subcellular localization of γH2AX or p53-S15P was performed using Fluoview FV10i (FV10C-W3; Olympus) using a UPlanSApo 60×, NA 1.2 water objective (Olympus). Images were acquired in Z-stacks composed of 9-13 images, and merged into one using Fiji ImageJ (Version 2.14.0/1.54f, build c89e8500e4) software with a maximum intensity setting.

### Live imaging of EGFP-tagged TTC33 or CCDC97

EGFP-CCDC97 or TTC33-EGFP expression was induced for 3 days by the addition of tetracycline to a final concentration of 100 ng/ml. The day before imaging, roughly 2 × 10^4^ cells were seeded per well in a Lab-Tek II Chambered Coverglass 1.5 Borosilicate Glass (155409PK, LifeTechnologies). Imaging was performed using a Fluoview FV10i (FV10C-W3; Olympus) with a UPlanSApo 60×, NA 1.2 water objective (Olympus). Imaging was performed with the microscope function of a chamber heating system set to 36 °C. Images were acquired in Z-stacks composed of 12-20 images, and merged into one using Fiji ImageJ (Version 2.14.0/1.54f, build c89e8500e4) software with a maximum intensity setting.

### Analysis of γH2AX or p53-S15P nuclear intensity

The p53-S15P or γH2AX nuclear signal was quantified using ImageJ (Version 2.14.0/1.54f, build c89e8500e4). The DAPI channel maximum intensity pictures were converted to binary images, which were smoothed, if need be, watersheded, and used to create RoiSets covering the nuclear signal (function Analyze Particles setting 10-Infinity).

To quantify total γH2AX or p53-S15P nuclear intensity, the RoiSets were directly applied to max intensity images depicting the γH2AX or p53-S15P signal. The average background intensity, obtained by measuring the signal from 5-10 areas outside cells, was subtracted from the nuclear signal. The median of the control image’s nuclear γH2AX or p53-S15P intensity was calculated and used to divide the intensities in the control and test sample images. Those values could be compared between replicates and are depicted on the graphs.

Graphs were prepared using RStudio (Version 1.2.5033 or Version 2024.04.2 + 764) and the ggplot2 package. To test for significance, a two-sample unpaired one-tailed Welch *t*-test with unequal variance was performed, using values merged from all replicates.

### Cell fractionation

Subcellular fractionation of HEK293 Flp-In T-REx cells was performed using a Subcellular Protein Fractionation Kit for Cultured Cells (78840; Thermo Fisher Scientific), according to the manufacturer’s instructions.

### Genomic alignments and bioinformatics analyses of protein motifs

Genomic alignments were prepared using the ECR Browser suite^59^ (https://ecrbrowser.dcode.org/). Detection of nuclear localization signals (NLS) was performed using the online NLS mapper^60^ (http://nls-mapper.iab.keio.ac.jp). Detection of TPR motifs in TTC33 was done using both the Prosite online tool^61^ (https://prosite.expasy.org) and the TPRpred^62–64^ (https://toolkit.tuebingen.mpg.de/tools/tprpred). Detection of the coiled-coil motif in CCDC97 was performed using an online PFAM resource^65^ (http://pfam.xfam.org). Protein alignments were prepared using the online MultAlin tool with the Blosum62-12-2 matrix^66^ (http://multalin.toulouse.inra.fr) or NCBI BLAST. To prepare graphs showing TTC33 or CCDC97 protein sequence conservation, 300 sequences were downloaded from NCBI and aligned. Next, the fraction of identical amino acids in reference to the human protein was calculated, omitting gaps. Lists of mutations in TTC33 were integrated from UniProt and cBioportal, both of which rely on The Cancer Genome Atlas^49^ data (https://www.cancer.gov/ccg/research/genome-sequencing/tcga) and COSMIC database (https://cancer.sanger.ac.uk/cosmic).

The entire analysis of TTC33 and CCDC97 protein sequence conservation, along with TPR and NLS motif, and mutation distribution can be found in Supplementary Dataset 1.

### Modeling of TTC33 and TANC structure and molecular dynamics simulation

To examine the putative TTC33 structure, we used models taken from the Swiss-Model database Repository^36^ (https://swissmodel.expasy.org/repository/uniprot/Q6PID6), which proposes protein structural models based on sequence homology to experimentally solved structures. The list of the highest-ranking templates proposed by Swiss-Model is presented in Supplementary Table 1. The TTC33 model obtained in the top-ranking 8chy template was presented in the main text and Fig. 6. However, all top 4 ranking templates also produced models of the TTC33 TPR domain strongly resembling the AlphaFold 3 generated structure (see below).

We also examined the TTC33 structure modeled in the context of the TANC complex, which was generated with the AlphaFold 3 algorithm^35^ (https://alphafoldserver.com) on the 10^th^ may 2024, using the full-length TTC33, PHF5A, and WDR61 amino acid sequences downloaded from UniProt (https://uniprot.org; Q6PID6: TTC33; Q9GZS3: WDR61; Q7RTV0: PHF5A). The quality of the TANC AlphaFold 3 model was tested using the MolProbity suite^38^ (http://molprobity.biochem.duke.edu). Additionally, models corresponding to human TTC33 residues 42–168 were generated on the 7^th^ November 2025 for a collection of vertebrate species listed in Fig. 1b, c. The list of full length sequences with the modeled region highlighted is provided in Supplementary Table 2.

The models were visualized using PyMol (PyMOL v1.7 Enhanced for Mac OS X; http://www.pymol.org; or Version 3.1.6.1).

The molecular dynamics simulations were prepared using GROMACS 2025.1 through the VisualDynamics suite (https://visualdynamics.fiocruz.br/)^67^. The MolProbity-corrected TANC model 0 was used as the starting structure. Preliminary simulations, which all showed TANC trimer stability, were performed using different box geometries, and force fields (CHARMM27, OPLS-AA and AMBER94). Those simulations were performed to assess and optimize the periodic boundary effects. As a result, an optimal system setup for production simulations was selected and is described below. Results of all simulations are deposited at Mendeley Data (doi: 10.17632/p822vhnbdj.1). The trajectories were visualized using PyMol (Version 3.1.6.1).

For the final production simulations, the protein topology was generated using the GROMACS pdb2gmx utility with the CHARMM27 force field and the TIP3P water model. Protonation states were assigned during topology generation. Histidine residues were assigned as HISE, HISD, or HISH according to their local environment. The histidine residues relevant to this study (chain A; corresponding to TTC33 His155 and H125) were assigned as HISE. The N- and C-termini were modeled in their charged forms (NH₃⁺ and COO⁻, respectively).

The protein was placed in a dodecahedral periodic box with a minimum distance of 1.2 nm between the solute and the box boundary and solvated with explicit TIP3P water molecules. Prior to ion addition, the system carried a net charge of −13 e and was neutralized by the addition of 13 Na⁺ counterions using the GROMACS genion utility. The final solvated system contained 10,505 protein atoms, 130,965 TIP3P water molecules (392,895 atoms), and 13 Na⁺ ions, corresponding to a total of 403,413 atoms.

Energy minimization was performed prior to equilibration. An initial steepest-descent minimization was carried out for a maximum of 5000 steps with a force convergence criterion of 1000 kJ mol⁻¹ nm⁻¹. All three systems converged within this criterion, requiring 2018, 1605, and 2061 steps for replicates 1, 2, and 3, respectively.

Following minimization, the system was equilibrated in the NVT ensemble at 300 K for 10 ps, followed by equilibration in the NPT ensemble for 10 ps to stabilize system density and pressure. Temperature and pressure coupling were applied during equilibration according to the parameters specified in the GROMACS input files. Three independent molecular dynamics replicas were performed using distinct initial velocity assignments generated during equilibration and carried forward into the production simulations. The equilibrated systems were subsequently used as starting points for 5 ns production molecular dynamics simulations under periodic boundary conditions. Long-range electrostatic interactions were calculated using the particle-mesh Ewald (PME) method.

### Correlating and clustering of TTC33 interactors by human tissue expression

Raw tissue mRNA abundance for the top 42 TTC33 interactors listed in Supplementary Fig. 1f and Supplementary Dataset 2 was obtained from the Fagerberg et al.^31^ dataset. A Spearman ρ matrix of correlation coefficients was obtained using the R cor() function. A correlation distance matrix (1 − ρ) was generated and hierarchical clustering was performed using the hclust() function with complete linkage. The resulting dendrogram was cut into four clusters using cutree() (*k* = 4). A separate ρ matrix was generated and visualized using corrplot() with hierarchical clustering based on the same distance metric.

### Assessment of TTC33 sensitivity to proteasomal decay

Various HEK293 cell lines were grown on 6-well plates in DMEM, if need be supplemented with tetracycline to induce microRNA expression or production of constructs of interest. MG132 was added to a final concentration of 20 μM for 7 h using concentrated DMSO stock. Control cells were treated with DMSO alone. Cells were harvested, proteins were extracted using urea buffer, run in a SDS-PAGE gel, and analyzed by western blotting.

### Cell line growth rate estimation

To evaluate cell line growth rate, we used the parental HEK293 Flp-In T-REx cell line and its derivatives containing genome-integrated plasmids coding for microRNAs directed against mRNAs coding for proteins of interest or/and expressing proteins of interest.

Protein expression was induced with tetracycline (100 ng/ml final concentration) for a total of 6 days. Technically, the cells were grown first on a Ø60 mm dish in the presence of tetracycline for 3 days, and then transferred to a 96-well plate for another 3-day growth period in the IncuCyte S3 live-cell imaging system (4647; Essen BioScience).

Effects of microRNA-mediated protein depletions using a 100 ng/ml tetracycline were tested for a total of 3, 6, or 9 days as indicated in the Fig. 10a. To this end, the cells were either directly seeded in a 96-well plate with tetracycline, or pre-treated with tetracycline for 3 or 6 days prior to transfer to 96-well plate and growth evaluation using IncuCyte S3 apparatus. In addition, we also used HEK293 Flp-In T-REx, HeLa Flp-In T-REx, HCT116, and A549 cell lines transfected with siRNAs against TTC33 or PPP2R2A. For siRNA-mediated knock-downs, two consecutive transfections were performed, separated by 4 days. A day after the second transfection cells were seeded in 96-well plates to evaluate growth in the IncuCyte S3 apparatus.

Generally, to determine cell culture growth rate using the IncuCyte S3 live-cell imaging system, the cells were plated in a 96-well plate at a starting confluence typically below 20% (5 × 10^3^ cells of either type per well on a 96-well plate). Each cell line was seeded in at least 4 to 5 wells. This number of technical replicates allowed to discard results from unevenly seeded wells. Growth was typically evaluated during 72 up to 144 h in 4 hour intervals by taking one bright field picture of each well. To control for miRNA expression, pictures were also taken using the red light channel, as the microRNAs were expressed along with mCherry. To control for protein expression, pictures were taken using the green channel, as proteins of interest fused to EGFP were mostly expressed.

To test for the recovery capacity of HEK293 cells (control or depleted of UNG1/2, TTC33, CCDC97 or overexpressing TTC33 or UNG2) after hydrogen peroxide treatment, expression of microRNAs and/or protein of interest was induced for 3 days prior to exposure to 0.01% H_2_O_2_. Hydrogen peroxide was added to 50-60% confluent cells for 30 min. and then removed by replacing the growth media with a fresh batch (containing tetracycline). The cells were left to recover for 24 h and then seeded onto a 96-well plate to assess growth for another 72 h in an IncuCyte S3 live-cell imaging system.

To test for survival in the presence of doxorubicin, HEK293 control or cells depleted of or overexpressing TTC33 were exposed to 100 ng/ml tetracycline for 3 or 6 days prior to the experiment to ensure TTC33 knock-down or overexpression. Cells were plated under tetracycline on 96-well plates at a minimum of 5 and a maximum of 25% initial confluence (replicates with higher initial confluence were rejected). 24 h after seeding, when cells attached to the growth surface, the media was complemented with doxorubicin to final concentration of 0, 0.5, 0.1, 0.05, 0.025, or 0.01 μM. Thus, prepared plates were grown in the IncuCyte S3 live-cell imaging system for a minimum of 4 and a maximum of 5 days.

### Calculation of logistic growth rates under non-stress conditions

To calculate the growth rate, the bright field pictures were converted to a confluence mask using IncuCyte 2019B Rev2 software. The increase in the percentage of surface occupied by the cells was calculated. The resulting raw confluence data is deposited at Supplementary Dataset 3 and Mendeley (doi: 10.17632/p822vhnbdj.1). Next, the raw confluence numerical data were subjected to logistic curve fitting following the equation:

2 $$y=\frac{100}{1+\exp (\frac{(x_{{MidPoint}}-x)}{k})}$$

where *x* is time (hours), *y* is confluence (% surface area occupied in the bright-field image), and *k* is the logistic scale parameter obtained from nonlinear least-squares fitting in R (nls). The parameter *k* corresponds to the characteristic time scale of the curve and is the reciprocal of the classical logistic growth rate.

To derive the fitted equation coefficients the nls() protocol in RStudio (Version 1.2.5033 or Version 2024.04.2 + 764) was used. The curve plateau values were fixed at a minimum of 0 and a maximum of 100 (-/ + 3%). The resulting logistic functions were cross-compared with the experimental data to ensure correct fitting.

The template code is deposited at Mendeley (doi: 10.17632/p822vhnbdj.1), and in Supplementary Dataset 3. The resulting logistic growth rates were summed up in Supplementary Dataset 3 and directly compared. For the readers’ ease, the reciprocals of the R scaling factor (i.e., the actual logistic growth rate factors) are shown in the Figures (both values are given in the Supplementary Dataset 3). In this setting, the higher the growth rate values, the faster the cell growth. To enable drawing average growth profiles, the normalized midpoint values were calculated so that at time-point 0 min the logistic curve confluence was equal to ~5%. The relationship between the raw growth rate and the normalized mid-point was estimated using linear fitting at:

3 $${normalize}{d}_{{MidPoint}}=2.955\cdot k+0.0007186187$$

### Calculation of growth rates under doxorubicin treatment

Depending on doxorubicin concentration, different curve fitting strategies were used. The choice of the fitted function was motivated by visual inspection of raw growth curves.

At low doxorubicin concentrations of 0.01 μM and 0.05 μM, the growth curves retained the usual appearance characteristic of non-treated cells. For those, logistic growth rates were calculated as described above.

At 0.5 μM doxorubicin, a reduction of the initial population was observed. We chose to represent this with a logistic decay. To this end, the nls() protocol in RStudio was used. The curve plateau values were fixed at a maximum of 0 and a minimum of 100 (±3%). The resulting R scaling factors and logistic growth rates have negative values.

At intermediate doxorubicin concentrations of 0.1 μM and 0.05 μM, the curves displayed initial growth up till around 65–80 h, followed by a peak of varied width and gradual population decline. Two fitting strategies were used. First, the distribution was limited to the area of active growth (~65 h) and logistic growth fitting was performed as described in the previous section. This allowed to obtain a collection of coefficients directly comparable with control and extreme doxorubicin exposures. To represent the entire curve, a Gaussian bell-like distribution was fitted using the nls() protocol in RStudio (Version 1.2.5033 or Version 2024.04.2 + 764) and the following equation:

4 $$y=A\cdot \exp \left(\frac{-{\left(x-{mu}\right)}^{2}}{(2\cdot {sigm}{a}^{2})}\right)$$

where *x* is time (hours), *A* is the amplitude corresponding to the maximum confluence, *mu* is the position of the peak (i.e., the time at which maximum confluence is reached), and *sigma* is the standard deviation parameter controlling the width of the distribution.

To normalize for differences in cell seeding at the beginning of the experiment, the starting confluence was subtracted from all points of the distribution. Multiplication by this value repositions the distribution at the raw experimental level. The three resulting Gaussian bell-like factors are listed in Supplementary Dataset 3 for relevant samples. The code for fitting Gaussian bell-like functions are given in Supplementary Dataset 3.

### Comet assay

Comet assay was performed using Comet Assay Kit (3 well slides; ab238544; Abcam) according to the manufacturer’s recommendations with some modifications. Before first use, Comet agarose was heated to 95 °C in a water bath for 20 min. until liquified, mixed thoroughly, and aliquoted. 1 ml aliquots were then stored at room temperature. Before each experiment, an aliquot of agarose was melted in a thermoblock at 95 °C for 20 min. and then placed at 37 °Cfor 20 min. to cool down. 65 μl of agarose solution was then spread over each well of the Comet slide with the pipette tip to create an even base layer. The slide was then transferred to 4 °C for 15 min., being kept in a horizontal position. HEK293 Flp-In T-REx cells (non-modified, miTTC33, or miUNG) grown in Ø100 mm dishes to 90% confluence were washed with PBS, detached with trypsin-EDTA, resuspended in 5 ml of ice-cold PBS, and centrifuged at 700 × *g* for 2 min. Following one additional wash with ice-cold PBS, the cell pellet was resuspended in PBS at 1 × 10^6^ cells/ml. 30 μl of the cell suspension was then mixed with 120 μl of the Comet agarose (1:4 ratio), and 65 μl of the solution was transferred onto the top of the initial base layer pre-solidified on the Comet slide well. The slide was again moved to 4 °C for 15 min. while being maintained in a horizontal position. The slide was subsequently immersed in 20 ml of the pre-chilled lysis buffer (1× Lysis solution; 2.5 M NaCl; 100 mM EDTA; 10% DMSO; pH adjusted to 10.0 using 10 M NaOH) and incubated for 60 min. at 4 °C in the dark. Following careful removal of the lysis buffer with an automatic pipette to prevent agarose detachment, the slide was incubated with 20 ml of pre-chilled alkaline solution (0.3 M NaOH and 1 mM EDTA) for 30 min. at 4 °C in the dark, and then washed twice for 5 min. with cold 1× TBE buffer (89 mM Tris-base; 89 mM boric acid; 2 mM EDTA). The slide was subsequently transferred to the electrophoresis tank filled with pre-chilled 1× TBE and exposed to 54 V (3 V/cm) for 40 min. Following electrophoresis, the slide was rinsed three times with 20 ml of cold water for 2 min. and once with 20 ml of pre-chilled 70% ethanol. The slide was air-dried and eventually 100 μl of Vista Green DNA Staining Dye diluted 1:10 000 in TE buffer (10 mM Tris-HCl pH 7.5; 1 mM EDTA) was added to each well. Staining was performed for 15 min. at room temperature in the dark.

To quantify the data, a minimum of 50 images of cell nuclei were taken using an Olympus IX81 fluorescent microscope under a FITC filter and 40× magnification. The exact number of captured nuclei is indicated in Fig. 9b. Pictures were quantified in ImageJ (Version 2.14.0/1.54 f, build c89e8500e4) by drawing a thick line (linewidth: 100) spanning the entire nuclei from its beginning until well behind the end of the comet. The pixel intensity was listed and analyzed in RStudio. To draw the average profile in Fig. 9b, the intensity data were internally normalized to the brightest pixel for each comet and this given maximum was defined at position 0. The ggplot2 package geom_smooth protocol was used to draw an average profile.

### UNG glycosylase assay

UNG glycosylase assay in whole-cell extracts was performed as previously described by Weeks et al.^68^ on a double-stranded DNA substrate prepared by annealing of two synthetic strands labeled with different fluorophores: (1) 5′[HEX]-GTAAAACGACGGCCAGTG**U**CTTCGAGCTCGGTACCCGGGG-3′ and (2) 3′-CATTTTGCTGCCGGTCACAGAAGCTCGAGCCATGGGCCCC[Cy5]-5′ in a buffer containing 10 mM Tris-HCl pH 8.0 and 20 mM KCl. Prior to the preparation of the cell extract, proteins of interest were knocked-down using miRNAs for 3 or 6 days. Cells were washed once with ice-cold PBS, detached with trypsin-EDTA, and collected in PBS by centrifugation at 300 × *g* for 5 min. Cell pellets were washed three times in ice-cold PBS containing 1 mM PMSF and protease inhibitors cocktail, and then resuspended in three volumes of the lysis buffer (50 mM Tris-HCl pH 7.5, 60 mM NaCl, 1 mM EDTA, 0.1% (v/v) NP40, 1 mM DTT, protease inhibitors), and disrupted on ice using Dounce homogenizer (10 up-and-down strokes of the tight-fitting pestle). Lysates were centrifuged at 12,000 × *g*, 4 °C for 20 min. Supernatants (whole-cell extracts) were collected and protein concentration was measured using the Bradford assay. UNG activity assay was performed in a 20 μl volume using freshly prepared, 0.22 μm filter-sterilized reaction buffer (20 mM Tris-HCl pH 8.0, 100 mM NaCl, 1 mM EDTA, 1 mM DTT), 5 or 15 μg of protein extract, and 5 μl of 1 μM dsDNA substrate. The reactions were performed at 37 °C and stopped at different time points within 90 min. after the start of the incubation by collecting an aliquot of the mixture and adding it to an equal volume of formamide gel loading dye (90% formamide, 20 mM EDTA, 0.03% bromophenol blue, and 0.03% xylene cyanol in 1× TBE), followed by snap freezing in liquid nitrogen. After thermal denaturation at 90 °C for 5 min., reaction products were resolved in denaturing 20% polyacrylamide/8 M urea/1× TBE gel. A Typhoon FLA 9000 laser scanner (GE Healthcare) was used to visualize the conversion of fluorescently labeled substrate to the cleaved product.

### BrdU incorporation and cell cycle analysis

The day before labeling, 6 × 10^3^ HEK293 cells were seeded into each well of a 384-well plate (781946, Greiner). Once the cells adhered, BrdU (B5002; Sigma) was added to a final concentration of 20 μM, and the cells were incubated for 18 h. Following incubation, the cells were washed twice with 100 μl of PBS using a 405 Select LS plate washer (BioTek).

Next, the cells were fixed in 4% formaldehyde with 0.25% Triton X-100 in PBS for 30 min. at room temperature. This was followed by two washes with 100 μl of PBS and 100 μl of distilled water. Subsequently, HCl was added at a concentration of 0.25 M, and the cells were incubated for 20 min. The cells were then washed four times with 100 μl of PBS and incubated for 30 min. in 3% BSA in PBS as a blocking solution.

After removing the blocking solution, anti-BrdU antibodies were added at a concentration of 0.5 μg/ml in 3% BSA and incubated overnight at 4 °C. The following day, the cells were washed four times with 100 μl of PBS. Alexa Fluor 647-conjugated anti-mouse IgG1 secondary antibodies were then added at a concentration of 2 μg/ml in 3% BSA and incubated for 1 h at room temperature. Afterward, the cells were washed twice with 100 μl of PBS and incubated for 10 min. with 2 μg/ml Hoechst in PBS. The cells were washed again twice with 100 μl of PBS and then left in 50 μl of PBS.

The plate was covered with adhesive sealing film (04-095-0060; Nerbe) and imaged using a ScanR automated wide-field microscope (Olympus) with an UPlanSApo 20×, NA 0.75 objective (Olympus). Images were collected for a single plane in two fluorescence channels: Hoechst and Alexa Fluor 647 (representing BrdU). Image analysis was conducted using dedicated ScanR software.

Image segmentation was performed based on the fluorescence signal from the Hoechst channel to identify cell nuclei. Integrated fluorescence intensity from both Hoechst and incorporated BrdU was measured within the nuclei. The Hoechst fluorescence intensity was used to determine the cell cycle phase of individual cells. The results of the fluorescence intensity quantification were then analyzed using the R environment. Typically 300 cells were analyzed to determine cell cycle parameters in one technical replicate.

### Calculation of TAN factors’ clinical significance for survival, 5-year survival, and cancer stage

The data used to produce graphs and tables presented in Fig. 10h, i and Supplementary Fig. 9e are in whole based upon data generated by the TCGA Research Network^49^ (https://www.cancer.gov/tcga), and acquired via the Human Protein Atlas (proteinatlas.org). The downloaded raw data are presented in Supplementary Dataset 4. Expression cut offs (high vs low) were applied as suggested by TCGA, and essentially correspond to the distribution mean value. Significance was tested using the chi-square test, and logistic regression analysis, using SPSS (v29 for Windows).

### Statistics and reproducibility

Co-immunoprecipitation coupled to label-free mass spectrometry (LF-MS) was performed using between two and five independent biological replicates, depending on the experiment (HEK293 TTC33-EGFP, n_bait=2/n_control=1; HeLa TTC33-EGFP, n_bait=4/n_control=4; FreeStyle 293-F TTC33-EGFP, n_bait=4/n_control=3; HEK293 MBP-TTC33, n_bait=3/n_control=2; HEK293 EGFP-CCDC97, n_bait=5/n=control=4; HEK293 EGFP-PPP2R2A, *n* = 2/n_control_4; HEK293 EGFP-WDR61 WT and W20C, n_bait=2 each/n_control=1). All protein interactions identified by LF-MS were subsequently validated using independently prepared samples by co-immunoprecipitation followed by western blotting, for which at least two independent biological replicates were performed, if possible using different tags. Whenever possible, and particularly for key interactions, co-immunoprecipitation-western blot analyses were additionally reproduced in two or three independent cell lines (HEK293, HeLa, or FreeStyle 293-F). LF-MS and co-immunoprecipitation-western blotting represent orthogonal experimental approaches that independently support the identified protein interactions.

The size-exclusion chromatography (SEC) analysis shown in this study comprises four independent purifications of the TANC trimer performed either alone or together with tagged or untagged CCDC97 or HA-UNG2. Because these samples were generated from independently grown and transfected cell cultures, and each purification yielded independently isolated TANC complexes, the four SEC experiments represent four biological replicates. Protein purification and SEC conditions were independently optimized in preliminary experiments that are not shown.

The analyses of protein levels in whole-cell extracts presented in Fig. 8 were independently reproduced in at least three biological replicates. Where possible, two representative biological replicates are shown in the figure (for example, Fig. 8g).

Immunofluorescence experiments were performed using a minimum of two and a maximum of five independent biological replicates, all of which were included in the statistical analyses. In the accompanying graphs, small dots represent individual nuclei and are grouped according to biological replicate. Larger dots indicate the mean value of each biological replicate. Box plots combine measurements from all biological replicates. The total number of analyzed nuclei is indicated by n in each graph.

Growth assays and cell-cycle analyses were performed using between three and forty-four independent biological replicates, depending on the experiment. Growth assays were initially established to quantify proliferation rates and were subsequently extended to assess sensitivity to genotoxic agents. Control samples acquired during these experiments were also included in the analyses presented in Fig. 10a. Cell-cycle analyses were performed using biological replicates composed of multiple technical replicates, as described in the corresponding figure legends.

DNA glycosylase activity assays were performed using between two and four independent biological replicates. The comet assay was independently repeated three times and consistently demonstrated increased chromatin fragmentation following TTC33 or UNG1/2 depletion. The most representative experiment is shown in the figure.

Unless stated otherwise in the figure legends, statistical analyses were performed using one-tailed Welch’s two-sample *t*-tests. Fisher’s exact test was used for contingency table analyses. Box plots display the median, with the box representing the 25th and 75th percentiles and whiskers indicating the 5th and 95th percentiles. Error bars, if applicable, are defined in figure captions.

### Inclusion and ethics statement

The study was conducted entirely at the institutions listed in the author affiliations. The data presented in the publication falls into the category of basic research, and was conducted in line with the Institution’s internal code of ethics. No ethics committee approval was required to conduct the research, including the clinical data, which is fully based on publicly available data acquired via the Human Protein Atlas (proteinatlas.org). The project was conducted mainly in the frame of the OPUS (2022/45/B/NZ1/02654) and also SONATA-BIS (2017/26/E/NZ1/00724) grants awarded by the National Science Centre to AT and RT respectively, who were the principal investigators.

### Reporting summary

Further information on research design is available in the Nature Portfolio Reporting Summary linked to this article.

## Supplementary information

Supplementary Information

Description of Additional Supplementary Files

Supplementary Data 1

Supplementary Data 2

Supplementary Data 3

Supplementary Data 4

Supplementary Data 5

Reporting Summary

Transparent Peer Review file

## Source data

Source Data 1

Source Data 2

## Data Availability

All data supporting the results of this study can be found in the article, supplementary, and source data files. The mass spectrometry proteomics data have been deposited to the ProteomeXchange Consortium via the PRIDE repository under codes PXD059336, PXD059337, PXD066861, PXD043138, and PXD043098. Quantitatively analyzed mass spectrometry data are provided in Supplementary Dataset 2 and Mendeley Data (https://doi.org/10.17632/p822vhnbdj.1)^69^. Source data, underlying all other analyses presented in this work, are deposited in the Mendeley Data repository [https://doi.org/10.17632/p822vhnbdj.1]^69^, and in relevant Supplementary Datasets. Source Data are provided with this paper as Source Data files.The clinical data presented here are in whole based upon data generated by the TCGA Research Network: https://www.cancer.gov/tcga, and acquired via the Human Protein Atlas (proteinatlas.org), and were publicly available under a Creative Commons Attribution-ShareAlike 4.0 International License (https://www.proteinatlas.org/about/licence). The relevant source publication was cited. The obtained data is presented in Supplementary Dataset 4. Source data are provided with this paper.

## Code availability

The custom code written to derive logistic growth coefficients from confluence data collected using the IncuCyte S3 live-cell imaging system (4647; Essen BioScience) and the IncuCyte 2019B Rev2 software is described in the “Methods” section and given in detail in Supplementary Dataset 3. Script written to determine protein enrichment in LF-MS dataset is deposited at Mendeley data (https://doi.org/10.17632/p822vhnbdj.1) in relevant figure folder. Molecular dynamics simulations were performed using the publicly available VisualDynamics web server^67^. No custom code was developed. Simulation command files and associated output data are available from Mendeley Data (https://doi.org/10.17632/p822vhnbdj.1).
