# Supplementary material for: TTC33 forms a complex with WDR61 and PHF5A to recruit factors relevant to genomic integrity
Source: Nat Commun. 2026 Aug 21;17:9984. doi: 10.1038/s41467-026-76928-z (PMC13590505; doi:10.1038/s41467-026-76928-z)
Supplement: Supplementary file 1 [file 41467_2026_76928_MOESM1_ESM.pdf]

SUPPLEMENTARY INFORMATION

TTC33 forms a complex with WDR61 and PHF5A to recruit factors relevant to genomic integrity

Rafał Tomecki[1,2]#, Małgorzata Drabko[1,3]#, Małgorzata Siek[1,3], Maria Matykiewicz[1], Miłosz Ludwinek[1,‡], Łukasz S. Borowski[4,5], Aneta Jurkiewicz[4], Kamil Kobyłecki[1], Agnieszka Jabłońska[1], Dominik Cysewski[6,§], Agata Malinowska[6], Magdalena Bakun[6], Rafał Płoski[7], Roman J. Szczęsny[4], and Agnieszka Tudek[1,*]

[1] Laboratory of RNA Processing and Decay, Institute of Biochemistry and Biophysics, Polish Academy of Sciences, A. Pawińskiego 5A, 02-106 Warsaw, Poland

[2] Laboratory of Epitranscriptomics, Institute of Bioengineering, Faculty of Biology, Biological and Chemical Research Centre, University of Warsaw, S. Banacha 2c, 02-097 Warsaw, Poland

[3] Doctoral School of Molecular Biology and Biological Chemistry at IBB PAS, A. Pawińskiego 5a, 02-106 Warsaw, Poland

[4] Laboratory of RNA Biology, Institute of Biochemistry and Biophysics, Polish Academy of Sciences, A. Pawińskiego 5A, 02-106 Warsaw, Poland

[5] Institute of Genetics and Biotechnology, Faculty of Biology, University of Warsaw, A. Pawińskiego 5A, 02-106 Warsaw, Poland

[6] Mass Spectrometry Laboratory, Institute of Biochemistry and Biophysics, Polish Academy of Sciences, A. Pawińskiego 5A, 02-106 Warsaw, Poland

[7] Department of Medical Genetics, Medical University of Warsaw, A. Pawińskiego 3c, 02-106 Warsaw, Poland

[‡] Present address: Chair and Department of Biochemistry, Medical University of Warsaw, S. Banacha 1, 02-097 Warsaw, Poland

[§] Present address: Medical University of Białystok, Centre for Regenerative Medicine, Waszyngtona 15 B,. 15-269 Białystok

# These authors contributed equally to this work

***** Correspondence: atudek@ibb.waw.pl

***

CONTENTS

Supplementary Figures 1–9

Supplementary Tables 1–2

Appendix

Source Data for Supplementary Blots

Supplementary References

a prediction of TPR motifs with TPRpred

START	1	2	3	4	5	6	7	8	9	10	11	12	13	14	15	16	17	18	19	20	21	22	23	24	25	26	27	28	29	30	31	32	33	34	END	p-value
17	V	T	S	Q	Q	F	E	A	E	A	A	D	E	K	D	V	V	D	N	D	E	G	N	W	L	H	A	I	K	R	R	K	E	I	50	7.7e-02
59	S	K	Q	L	K	D	E	G	A	S	L	A	E	N	K	R	Y	R	E	A	I	Q	K	W	D	E	A	L	Q	L	T	**P**	N	D	92	**4.4e-08**
93	A	T	L	Y	E	M	K	S	Q	V	L	M	S	L	H	E	M	F	P	A	V	H	A	A	E	M	A	V	Q	Q	N	**P**	H	S	126	**3.0e-06**
127	W	E	S	W	Q	T	L	G	R	A	Q	L	G	L	G	E	I	I	L	A	I	R	S	F	Q	V	A	L	H	I	Y	**P**	M	N	160	**7.6e-10**

proline

large hydrophobic : F, W, Y, L, I, M

small hydrophobic: G, V, A, P

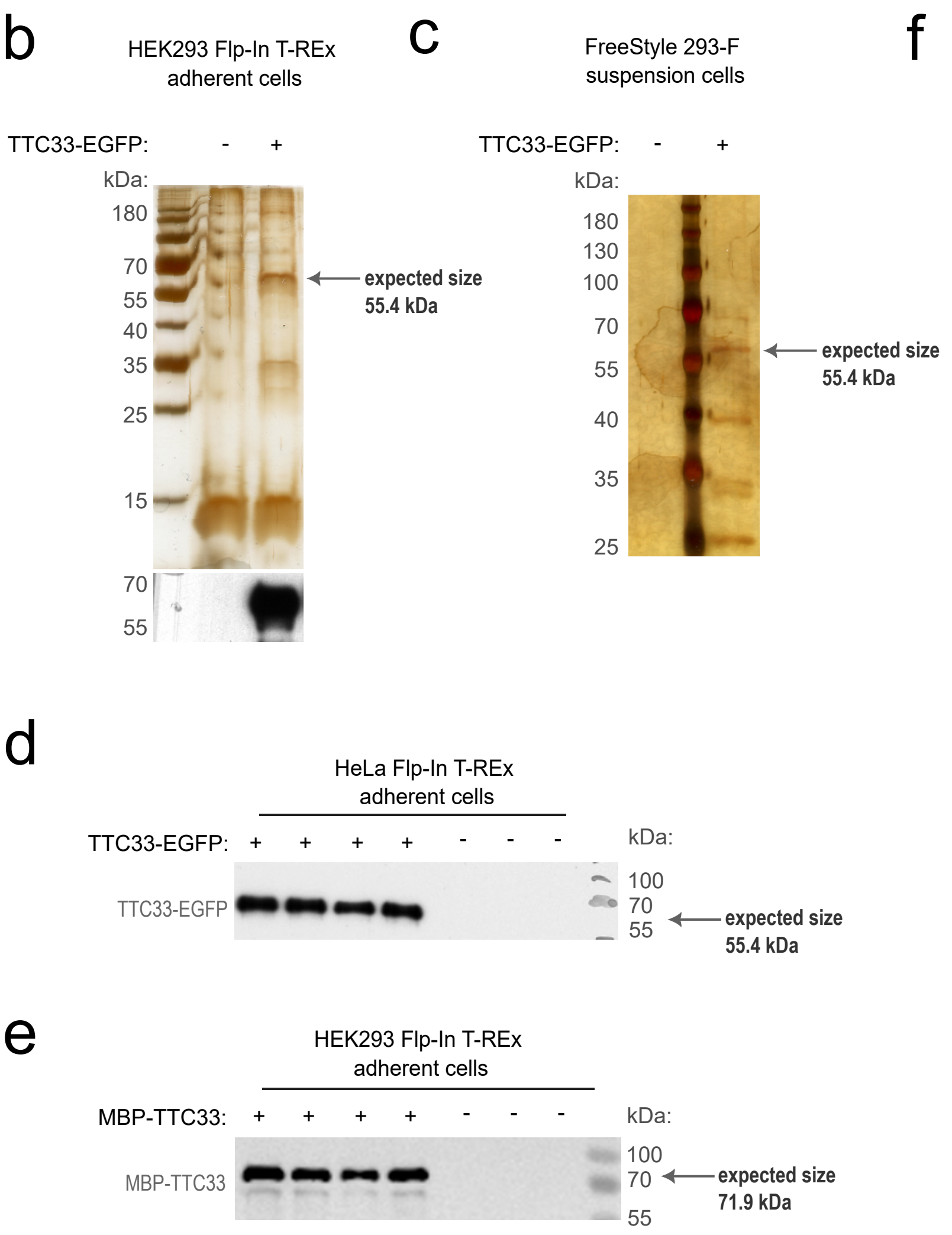

f

Gene.names	score_q60+q80	score_q60 (relaxed criteria)	score_q80 (stringent criteria)	
ALG1	5	3	2	
ATP1A1	6	4	2	
CAD	5	3	2	
CCAR2	5	3	2	
**CCDC97**	8	4	4	
COPB1	5	4	1	
FANCI	5	3	2	
GCN1L1	6	4	2	
**PPP2CA**	8	4	4	PP2A-B55α
**PPP2CB**	5	3	2	PP2A-B55α
**PPP2R1A**	7	4	3	PP2A-B55α
**PPP2R1B**	5	3	2	PP2A-B55δ
**PPP2R2A**	8	4	4	PP2A-B55α
**PPP2R2D**	8	4	4	PP2A-B55δ
PSMD3	5	3	2	
SACM1L	5	3	2	
**SF3A1**	5	4	1	SF3A
**SF3A2**	7	4	3	SF3A
**SF3A3**	7	4	3	SF3A
**SF3B1**	5	4	1	SF3B
**SF3B2**	5	4	1	SF3B
**SF3B3**	6	4	2	SF3B
**SF3B4**	7	4	3	SF3B
**SF3B5**	8	4	4	SF3B
**SF3B6**	8	4	4	SF3B
**PHF5A**	8	4	4	SF3B
SNRPA1	5	4	1	U2
SNRPB2	8	4	4	U2
SNRPD1	5	3	2	U2
SNRPD3	5	3	2	U2
SNRPE	5	3	2	U2
SFXN4	6	3	3	
SLC25A1	6	4	2	
SLC25A10	5	4	1	
SLC25A11	6	4	2	
SLC25A5	5	4	1	
SPTLC1	5	3	2	
SSR4	5	4	1	
STIP1	7	4	3	
**TTC33**	8	4	4	
**UNG**	8	4	4	
UQCRC2	5	3	2	
**WDR61**	8	4	4	

TAN factors (bold). Red circle: best score. complexes: PP2A-B55α, PP2A-B55δ, SF3A, SF3B, U2

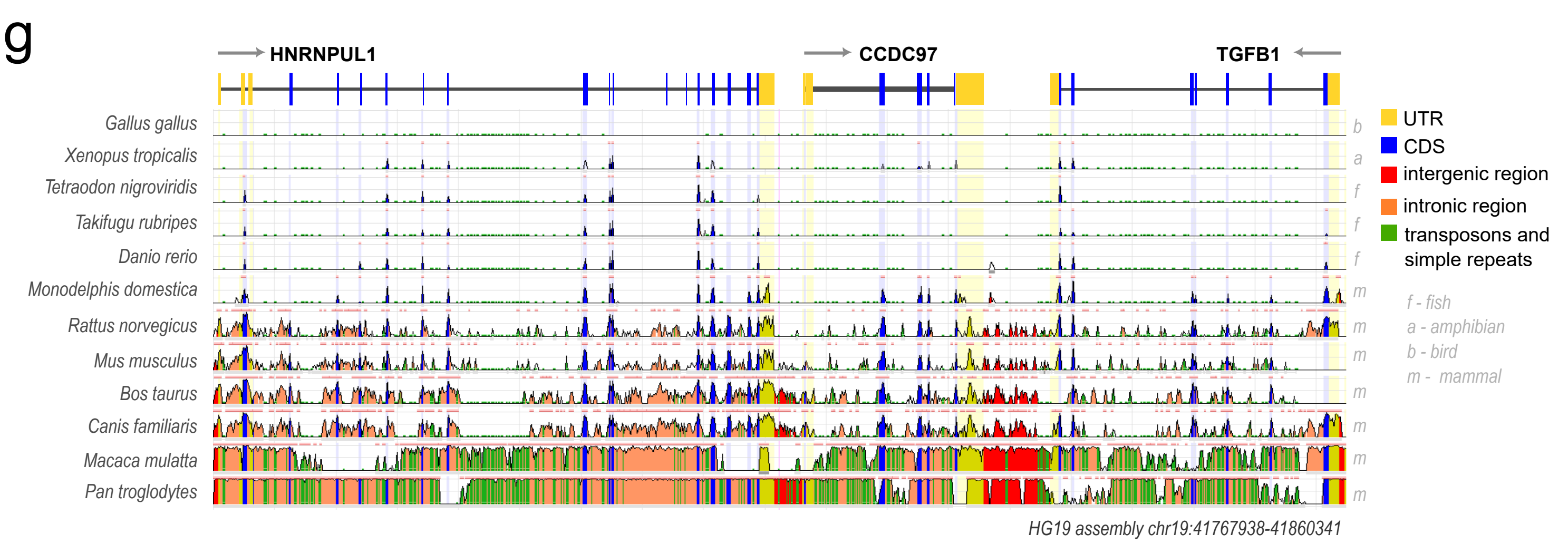

Supplementary Figure 1. Delineation of TTC33 TPR motifs and identification of TTC33 interactors by label-free mass spectrometry. a Alignment of four putative TPR motifs identified in the human TTC33 protein using the TPRpred suite[1,2,3]. The p-value, as given by the TPRpred suite, for each motif is indicated on the right. Blue and brown boxes on the alignment indicate the usual location of large and small hydrophobic residues, respectively, in a TPR motif. Below the alignment, the amino acids classified as large and small hydrophobic residues are listed. The position typically occupied by a proline residue in a TPR motif is highlighted in using a yellow box. **b** Evaluation of samples subjected to LF-MS analysis by SDS-PAGE silver-staining and western blot. TTC33-EGFP was purified from HEK293 adherent cells. **c** Evaluation of samples subjected to LF-MS analysis by SDS-PAGE silver-staining. TTC33-EGFP was purified from FreeStyle 293-F suspension cells. **d** Evaluation of samples subjected to LF-MS analysis by western blot. TTC33-EGFP was purified from HeLa adherent cells. **e** Evaluation of samples subjected to LF-MS analysis by SDS-PAGE silver-staining and western blot. MBP-TTC33 was purified from HEK293 adherent cells. **f** The table shows an extract from Supplementary Dataset 2, where a global comparison of all LF-MS datasets produced to identify TTC33 interactors is presented. The list shown in the figure presents the top 43 most enriched proteins. To produce this list, a cross comparison of all datasets was performed. For each dataset, we produced two lists of significantly enriched proteins: *stringent* and *relaxed*. For the stringent criteria, the interactor had to be among the 20% most enriched proteins and represented by at least 2 peptides. For the relaxed criteria, proteins was required to be among the 40% most enriched proteins and represented by at least 1 peptide. We merged the list of interactors identified using the relaxed criteria obtaining a total of 1334 proteins listed in Supplementary Dataset 2. Of those, 311 were identified in at least one dataset as enriched using the stringent criteria. For those 311 proteins, a gene ontology analysis was performed and is presented in the Supplementary Dataset 2. The most significant gene ontology terms indicated functions in splicing, which was mostly due to the presence of U2 SF3A/B sub-complexes, and other U2 associated factors. Cell cycle regulation, DNA repair, including double-strand break, nucleotide metabolism, and other functional groups were also mentioned with lower confidence. Next, we assigned 3 scores to each of the 1334 proteins obtained with the relaxed criteria. This was done to rank the interactors in order to define the most stable partners. *Score_q80* assigned 1 point if the protein was detected in a given dataset using the stringent criteria; thus the maximum score value was 4. *Score_q60* assigned 1 point if the protein was detected in a given dataset using the relaxed criteria; thus the maximum score value was also 4. *Score_q60+q80* is the sum of *score_q60* and *score_q80*, producing a maximum value of 8. The proteins most stably associated with TTC33 are highlighted in blue, and their highest score of 8 is marked by a red dot. Those interactors were confirmed by western blot analysis in Fig. 2d and in subsequent analyses shown in Fig. 4. To facilitate identification of multi-protein assemblies among the TTC33 interactor list, the sets of proteins known to form complexes are aggregated and marked with color-coded boxes. **g** Genomic alignment of the *CCDC97* and surrounding *HNRNPUL1* and *TGFB1* loci derived from various vertebrate species (displayed using the ECR Browser suite[4]). In the human genome, the gene is located on chromosome 19 (the genome assembly and coordinates are indicated on the figure). Source data are provided as a Source Data file (or appended to this document).

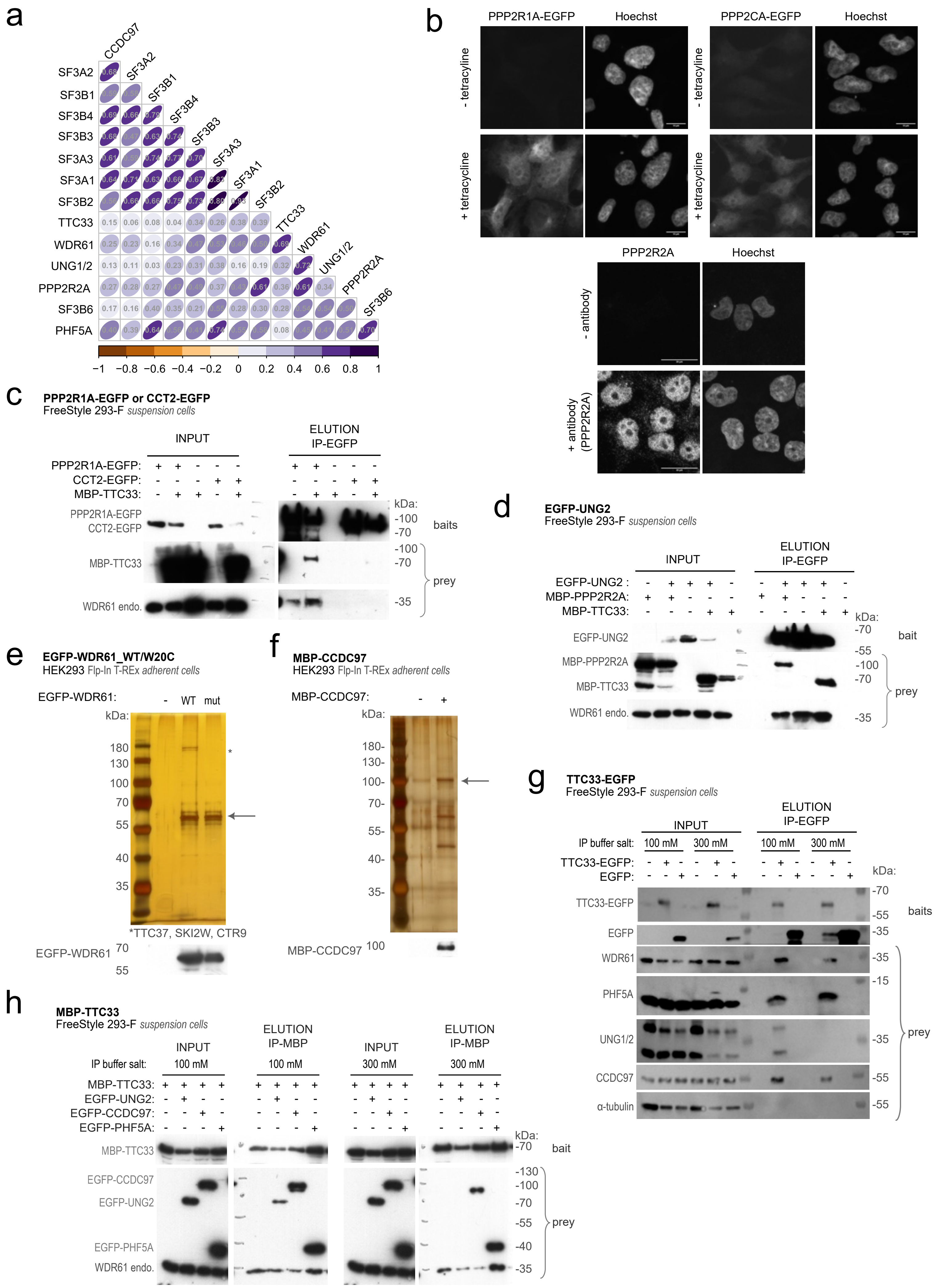

a
b
PPP2R1A-EGFP
Hoechst
PPP2CA-EGFP
- tetracycline
+ tetracycline
PPP2R2A
- antibody
+ antibody (PPP2R2A)
c
PPP2R1A-EGFP or CCT2-EGFP
FreeStyle 293-F suspension cells
INPUT
ELUTION IP-EGFP
PPP2R1A-EGFP:
CCT2-EGFP:
MBP-TTC33:
kDa:
baits
prey
MBP-TTC33
WDR61 endo.
d
EGFP-UNG2
FreeStyle 293-F suspension cells
EGFP-UNG2 :
MBP-PPP2R2A:
bait
e
EGFP-WDR61_WT/W20C
HEK293 Flp-In T-REx adherent cells
EGFP-WDR61:
WT
mut
*TTC37, SKI2W, CTR9
f
MBP-CCDC97
HEK293 Flp-In T-REx adherent cells
MBP-CCDC97:
g
TTC33-EGFP
FreeStyle 293-F suspension cells
IP buffer salt:
100 mM
300 mM
TTC33-EGFP:
EGFP:
WDR61
PHF5A
UNG1/2
CCDC97
α-tubulin
h
MBP-TTC33
FreeStyle 293-F suspension cells
ELUTION IP-MBP
EGFP-UNG2:
EGFP-CCDC97:
EGFP-PHF5A:

Supplementary Figure 2. Subcellular localization and interaction analysis of TTC33 interactors. a The graph presents a clustered correlation plot evaluating human tissue expression of TAN subunits. Data were derived from Fagerberg et al. (2014)[5]. Raw abundance of TAN factors is shown in Fig. 3a, while a broader correlation matrix including a larger TTC33 interactor list is shown in Fig. 3b. **b** The top panel shows microscopy images of subcellular localization of exogenously expressed EGFP-fused PPP2R1A or PPP2CA. Cells not expressing EGFP were used as a negative control. Bottom panel shows microscopy images of sub-cellular localization of endogenous PPP2R2A detected using a protein-specific antibody. **c** CoIP-WB analysis of proteins associating with PPP2R1A-EGFP or CCT2-EGFP. Endogenously expressed WDR61 was tested along with co-transfected MBP-TTC33. Since interaction between proteins was assessed by overexpressing both factors of interest, we used the CCT2-EGFP protein as a negative control. Because MBP-TTC33 did not interact with CCT2-EGFP, but did co-purify with PPP2R1A-EGFP, we conclude that the assay can be used to assess protein interactions *in vivo*. This approach was justified, as we have observed that PP2A co-purification with WDR61 was weak and we therefore wished to increase the detectability of the interaction. This also serves as a control for the experiment shown in Supplementary Fig. 2d. **d** CoIP-WB analysis of proteins associating with EGFP-UNG2. Endogenous WDR61 was tested along with co-transfected MBP-tagged PPP2R2A or TTC33. **e** Evaluation of samples subjected to LF-MS analysis by SDS-PAGE silver-staining and western blot. EGFP-WDR61 wild-type (WT) and W20C mutant (mut) were purified from HEK293 adherent cells. **f** Evaluation of samples subjected to LF-MS analysis by SDS-PAGE silver-staining and western blot. MBP-CCDC97 was purified from HEK293 adherent cells. **g** CoIP-WB analysis of TAN factors co-purifying with TTC33-EGFP from FreeStyle 293-F cells under 100 versus 300 mM NaCl conditions. TAN factors were detected using protein-specific antibodies. EGFP was also expressed as an additional negative control to show that the tag alone does not interact with endogenous TAN factors. **h** CoIP-WB analysis of proteins co-purifying with MBP-TTC33 from FreeStyle 293-F cells under 100 vs 300 mM NaCl concentration. EGFP-tagged CCDC97, UNG2, or PHF5A were co-transfected with the MBP-TTC33 bait. Endogenous WDR61 was detected using a protein-specific antibody. A different tag was used for the TTC33 bait to cross-verify the result presented in panel g. Source data are provided as a Source Data file (or appended to this document).

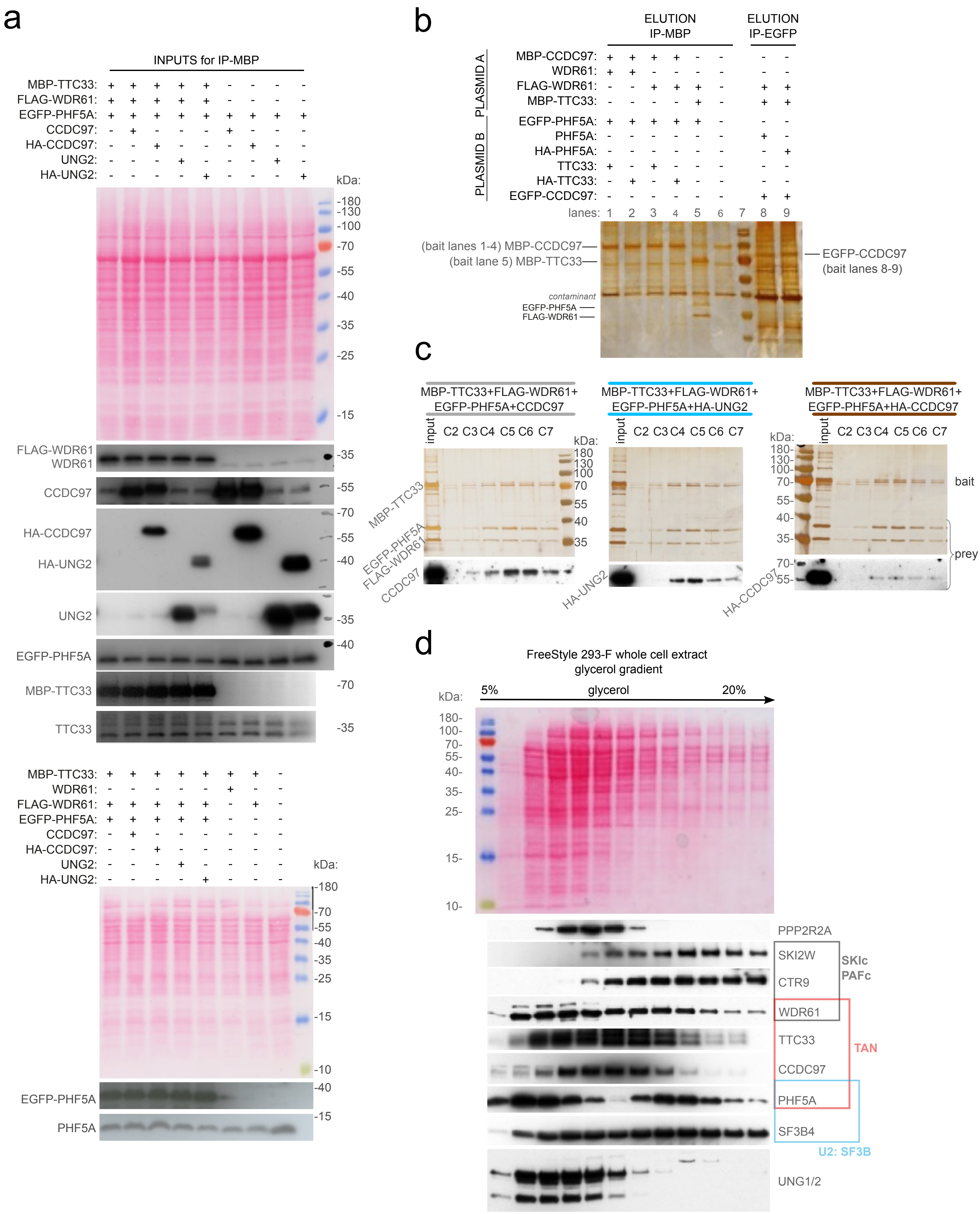

a
INPUTS for IP-MBP
MBP-TTC33:
FLAG-WDR61:
EGFP-PHF5A:
CCDC97:
HA-CCDC97:
UNG2:
HA-UNG2:
kDa:
-180
-130
-100
-70
-55
-40
-35
-25
-15
FLAG-WDR61
WDR61
CCDC97
HA-CCDC97
HA-UNG2
UNG2
EGFP-PHF5A
MBP-TTC33
TTC33
MBP-TTC33:
WDR61:
FLAG-WDR61:
EGFP-PHF5A:
CCDC97:
HA-CCDC97:
UNG2:
HA-UNG2:
kDa:
-10
PHF5A
b
ELUTION IP-MBP
ELUTION IP-EGFP
PLASMID A
PLASMID B
MBP-CCDC97:
WDR61:
FLAG-WDR61:
MBP-TTC33:
EGFP-PHF5A:
PHF5A:
HA-PHF5A:
TTC33:
HA-TTC33:
EGFP-CCDC97:
lanes: 1 2 3 4 5 6 7 8 9
(bait lanes 1-4) MBP-CCDC97
(bait lane 5) MBP-TTC33
contaminant
EGFP-PHF5A
FLAG-WDR61
EGFP-CCDC97 (bait lanes 8-9)
c
MBP-TTC33+FLAG-WDR61+ EGFP-PHF5A+CCDC97
MBP-TTC33+FLAG-WDR61+ EGFP-PHF5A+HA-UNG2
MBP-TTC33+FLAG-WDR61+ EGFP-PHF5A+HA-CCDC97
input C2 C3 C4 C5 C6 C7
MBP-TTC33
EGFP-PHF5A
FLAG-WDR61
CCDC97
HA-UNG2
HA-CCDC97
bait
prey
d
FreeStyle 293-F whole cell extract glycerol gradient
5% glycerol 20%
kDa:
180- 100- 70- 55- 40- 35- 25- 15- 10-
PPP2R2A
SKI2W
CTR9
WDR61
TTC33
CCDC97
PHF5A
SF3B4
UNG1/2
SKIc
PAFc
TAN
U2: SF3B

Supplementary Figure 3. Large-scale purification of TTC33 and associated proteins for size-exclusion chromatography. a Western blot analysis of whole cell extracts overexpressing proteins used for the purifications shown in Fig. 5a-c. **b** Results of various purifications using MBP- (*lanes 1-4*) or EGFP-tagged (*lanes 8-9*) CCDC97 as bait, co-expressed with TANC subunits. CCDC97 could not be co-purified at stoichiometric levels with TANC. For comparison, a purification of TANC using MBP-TTC33 co-expressed with FLAG-WDR61 and EGFP-PHF5A (*lane 5*) is shown. **c** Selected SEC fractions from Fig. 5c were separated by SDS-PAGE and analyzed by silver staining or western blot. **d** Western blot analysis of whole-cell extract of non-transfected FreeStyle 293-F separated in 5-20% glycerol gradients. Proteins were detected using the indicated antibodies. Source data are provided as a Source Data file (or appended to this document).

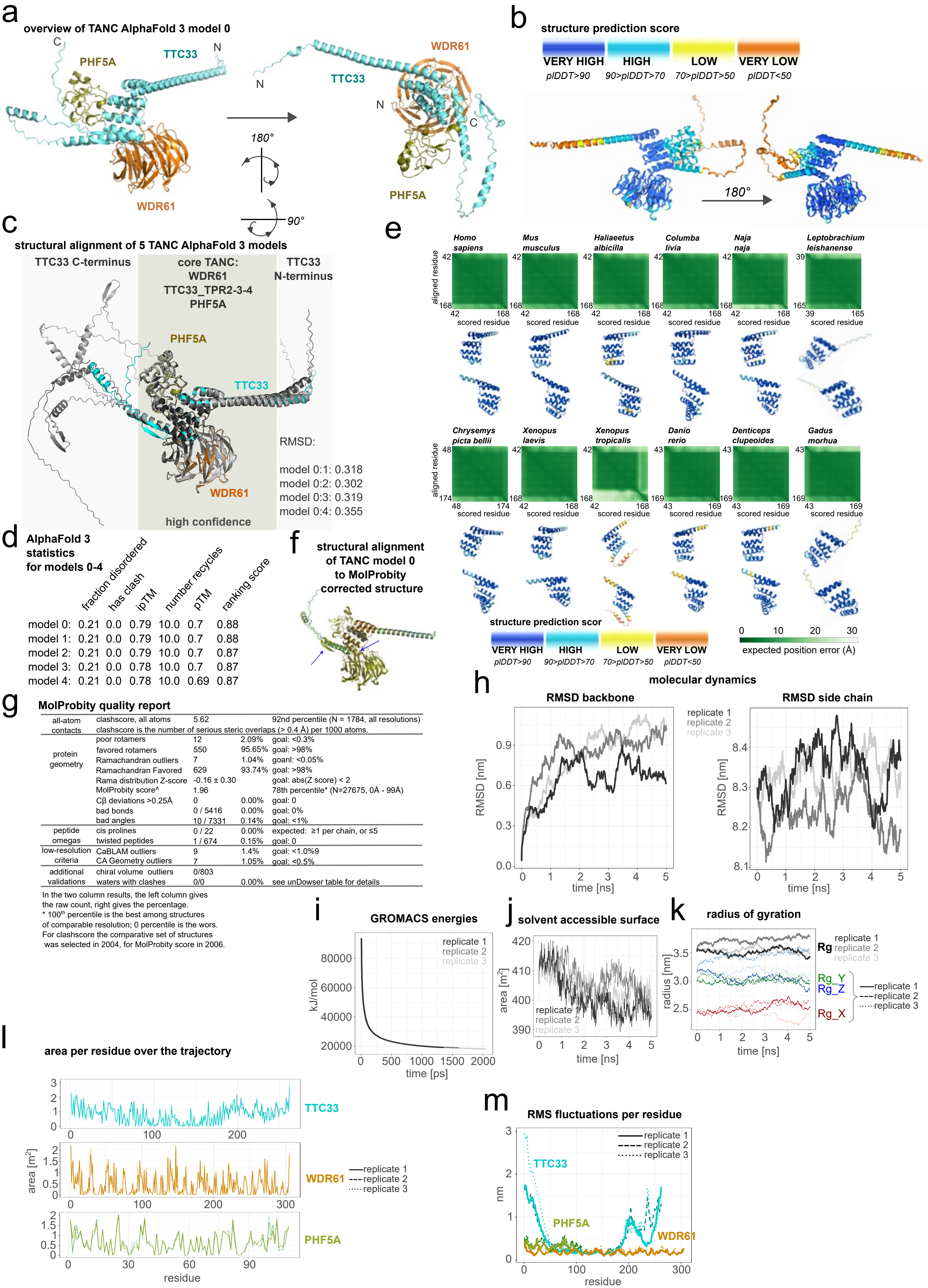
a
overview of TANC AlphaFold 3 model 0
C
N
PHF5A
TTC33
WDR61
180°
90°
b
structure prediction score
VERY HIGH
HIGH
LOW
VERY LOW
pIDDT>90
90>pIDDT>70
70>pIDDT>50
pIDDT<50
180°
c
structural alignment of 5 TANC AlphaFold 3 models
TTC33 C-terminus
core TANC: WDR61 TTC33_TPR2-3-4 PHF5A
TTC33 N-terminus
high confidence
RMSD:
model 0:1: 0.318
model 0:2: 0.302
model 0:3: 0.319
model 0:4: 0.355
d
AlphaFold 3 statistics for models 0-4
fraction disordered
has clash
ipTM
number recycles
pTM
ranking score
model 0: 0.21 0.0 0.79 10.0 0.7 0.88
model 1: 0.21 0.0 0.79 10.0 0.7 0.88
model 2: 0.21 0.0 0.79 10.0 0.7 0.87
model 3: 0.21 0.0 0.78 10.0 0.7 0.87
model 4: 0.21 0.0 0.78 10.0 0.69 0.87
e
Homo sapiens
Mus musculus
Haliaeetus albicilla
Columba livia
Naja naja
Leptobrachium leishanense
Chrysemys picta bellii
Xenopus laevis
Xenopus tropicalis
Danio rerio
Denticeps clupeoides
Gadus morhua
aligned residue
scored residue
structure prediction scor
VERY HIGH
HIGH
LOW
VERY LOW
pIDDT>90
90>pIDDT>70
70>pIDDT>50
pIDDT<50
0 10 20 30
expected position error (Å)
f
structural alignment of TANC model 0 to MolProbity corrected structure
g
MolProbity quality report
all-atom contacts | clashscore, all atoms | 5.62 | | 92nd percentile (N = 1784, all resolutions)
clashscore is the number of serious steric overlaps (> 0.4 Å) per 1000 atoms.
protein geometry | poor rotamers | 12 | 2.09% | goal: <0.3%
favored rotamers | 550 | 95.65% | goal: >98%
Ramachandran outliers | 7 | 1.04% | goanl: <0.05%
Ramachandran Favored | 629 | 93.74% | goal: >98%
Rama distribution Z-score | -0.16 ± 0.30 | | goal: abs(Z score) < 2
MolProbity score^ | 1.96 | | 78th percentile* (N=27675, 0Å - 99Å)
Cβ deviations >0.25Å | 0 | 0.00% | goal: 0
bad bonds | 0 / 5416 | 0.00% | goal: 0%
bad angles | 10 / 7331 | 0.14% | goal: <1%
peptide omegas | cis prolines | 0 / 22 | 0.00% | expected: ≥1 per chain, or ≤5
twisted peptides | 1 / 674 | 0.15% | goal: 0
low-resolution criteria | CaBLAM outliers | 9 | 1.4% | goal: <1.0%9
CA Geometry outliers | 7 | 1.05% | goal: <0.5%
additional validations | chiral volume outliers | 0/803
waters with clashes | 0/0 | 0.00% | see unDowser table for details
In the two column results, the left column gives the raw count, right gives the percentage.
* 100th percentile is the best among structures of comparable resolution; 0 percentile is the wors.
For clashscore the comparative set of structures was selected in 2004, for MolProbity score in 2006.
h
molecular dynamics
RMSD backbone
RMSD side chain
replicate 1
replicate 2
replicate 3
RMSD [nm]
time [ns]
i
GROMACS energies
kJ/mol
time [ps]
j
solvent accessible surface
area [m²]
time [ns]
k
radius of gyration
Rg
Rg_Y
Rg_Z
Rg_X
radius [nm]
time [ns]
l
area per residue over the trajectory
TTC33
WDR61
PHF5A
area [m²]
residue
m
RMS fluctuations per residue
TTC33
PHF5A
WDR61
nm
residue

Supplementary Figure 4. AlphaFold 3 modeling, MolProbity validation, and molecular dynamics simulations of TANC. **a** Different views of TANC model 0 generated by AlphaFold 3. **b** Two views of the model of the TANC complex proposed by AlphaFold 3. Complex elements are colored by predicted Local Distance Difference Test (pLDDT) score[6]). **c** Structural alignment of five TANC complex models proposed by AlphaFold 3. Model 0 is color-coded as in panel a to facilitate identification of the individual subunits, whereas models 1–4 are shown in gray. The core TTC33 TPR2-3-4 domain, WDR61, and PHF5A are predicted similarly in each model, whereas the N- and especially the C-terminus of TTC33 display the highest variability. RMSD values with respect to model 0 are indicated in the bottom right corner. **d** The table shows the quality report of five TANC complex models generated by AlphaFold 3. **e** The chart shows Predicted Aligned Error (PAE) plots and two structural views colored according to the pLDDT score for a collection of TTC33 TPR domains derived from various vertebrate species. The sequences used for AlphaFold 3 modeling are listed in Supplementary Table 2. The coordinates on the PAE chart denote the amino acid ranges used for modeling. **f** Structural alignment of the TANC AlphaFold 3 model with the structure corrected by the MolProbity suite. The RMSD is <0.001 Å. The original AlphaFold 3 model 0 was color-coded as in panel a and in main Figure 6d, whereas the MolProbity corrected model is shown in dark olive. Blue arrows indicate regions most divergent in both models. **g** MolProbity quality report on the TANC model. **h** Time evolution of RMSD values for the TANC backbone and side chains in a 5 ns molecular dynamics simulation. **i** Energy terms calculated by GROMACS during the TANC molecular dynamics simulation. **j** Solvent accessible surface of the TANC complex throughout the molecular dynamics simulation. **k** TANC complex radius of gyration: overall (Rg) and components (Rg_X, Rg_Y, Rg_Z) during the molecular dynamics simulation. **l** Changes in area per residue over the trajectory for TANC subunits during the molecular dynamics simulation. **m** Residue root mean square (RMS) fluctuations of TANC complex subunits during the molecular dynamics simulation. Source data are provided as a Source Data file or deposited at Mendeley Data (DOI: 10.17632/p822vhnbdj.1).

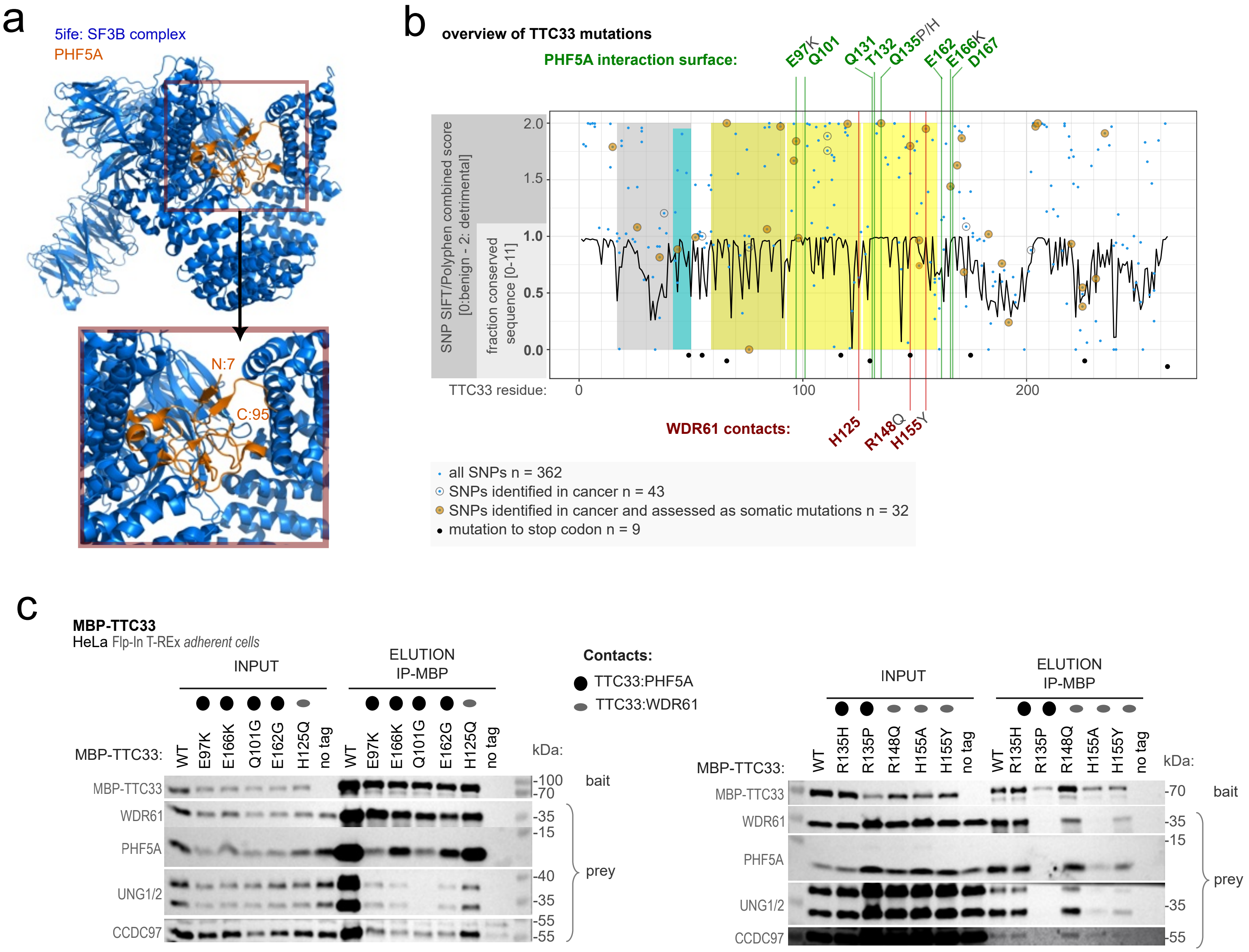
a
5ife: SF3B complex
PHF5A
N:7
C:95
b
overview of TTC33 mutations
PHF5A interaction surface:
E97K
Q101
Q131
T132
Q135P/H
E162
E166K
D167
SNP SIFT/Polyphen combined score [0:benign - 2: detrimental]
fraction conserved sequence [0-11]
2.0
1.5
1.0
0.5
0.0
TTC33 residue:
0
100
200
WDR61 contacts:
H125
R148Q
H155Y
all SNPs n = 362
SNPs identified in cancer n = 43
SNPs identified in cancer and assessed as somatic mutations n = 32
mutation to stop codon n = 9
c
MBP-TTC33
HeLa Flp-In T-REx adherent cells
INPUT
ELUTION IP-MBP
Contacts:
TTC33:PHF5A
TTC33:WDR61
MBP-TTC33:
WT
E97K
E166K
Q101G
E162G
H125Q
no tag
R135H
R135P
R148Q
H155A
H155Y
kDa:
MBP-TTC33
WDR61
PHF5A
UNG1/2
CCDC97
-100
-70
-35
-15
-40
-55
bait
prey

Supplementary Figure 5. Characterization of TTC33 mutations and their effects on TAN integrity. a The top part shows a view of the SF3B subcomplex of U2 splicing factor determined by Cretu et al. 2016 (PDB code: 5ife[7]). All SF3B complex subunits except PHF5A are shown in blue, whereas PHF5A is highlighted in orange. The bottom part shows a close-up of the PHF5A subunit, with its N- and C-termini highlighted. The numbers indicate the first or last PHF5A residue visible in the structure. **b** The graph shows the TTC33 residue evolutionary conservation (as in Fig. 1b; Supplementary Dataset 1) superimposed with the distribution of population variants and somatic cancer mutations (see legend for details). To score the mutations, a combined SIFT-Polyphen score was applied (Polyphen + [1-SIFT]), where 2 indicates a likely detrimental variant, whereas 0 designates a likely benign. **c** CoIP-WB analysis of TAN factors co-purifying with MBP-TTC33 wild-type (WT) or point mutants from HeLa cells (for an equivalent assay in HEK293 cells see Fig. 7i). Population or somatic variants used in this experiment are listed in Fig. 7h. For H155, and for Q101 and E162 residues, for which no population or somatic variants have been reported, substitutions to alanine or glycine were used. For ease of interpretation, variants are color-coded to indicate whether they potentially participate in the TTC33:PHF5A or TTC33:WDR61 interaction interface. Source data are provided as a Source Data file (or appended to this document).

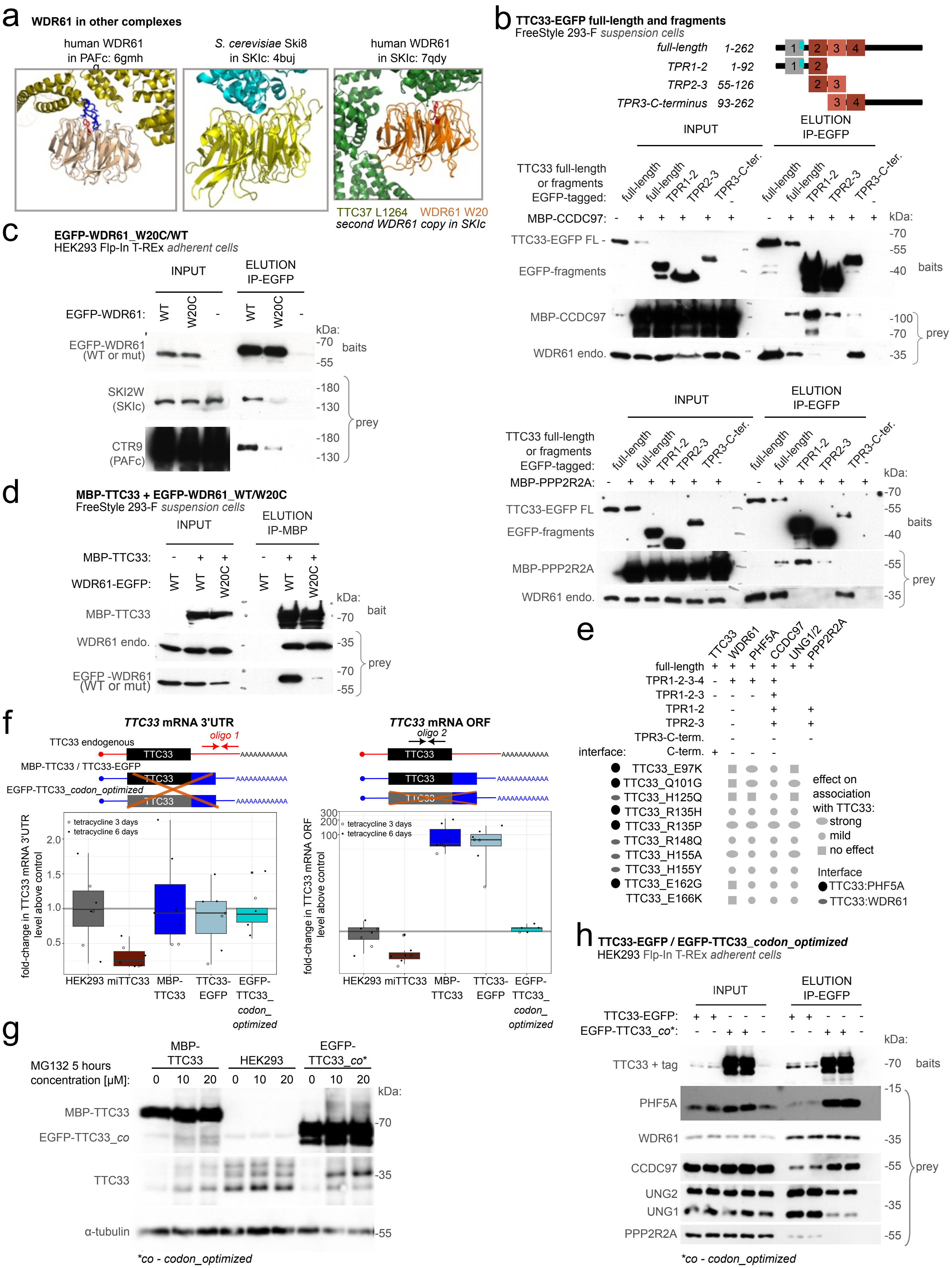
a
WDR61 in other complexes
human WDR61 in PAFc: 6gmh
S. cerevisiae Ski8 in SKIc: 4buj
human WDR61 in SKIc: 7qdy
TTC37 L1264
WDR61 W20
second WDR61 copy in SKIc
b
TTC33-EGFP full-length and fragments
FreeStyle 293-F suspension cells
full-length 1-262
TPR1-2 1-92
TRP2-3 55-126
TPR3-C-terminus 93-262
INPUT
ELUTION IP-EGFP
TTC33 full-length or fragments EGFP-tagged:
full-length full-length TPR1-2 TPR2-3 TPR3-C-ter. -
MBP-CCDC97:
kDa:
TTC33-EGFP FL
EGFP-fragments
MBP-CCDC97
WDR61 endo.
baits
prey
MBP-PPP2R2A:
c
EGFP-WDR61_W20C/WT
HEK293 Flp-In T-REx adherent cells
INPUT
ELUTION IP-EGFP
EGFP-WDR61:
WT W20C -
EGFP-WDR61 (WT or mut)
SKI2W (SKIc)
CTR9 (PAFc)
baits
prey
d
MBP-TTC33 + EGFP-WDR61_WT/W20C
FreeStyle 293-F suspension cells
INPUT
ELUTION IP-MBP
MBP-TTC33:
WDR61-EGFP:
MBP-TTC33
WDR61 endo.
EGFP -WDR61 (WT or mut)
bait
prey
e
TTC33 WDR61 PHF5A CCDC97 UNG1/2 PPP2R2A
full-length
TPR1-2-3-4
TPR1-2-3
TPR1-2
TPR2-3
TPR3-C-term.
interface: C-term.
TTC33_E97K
TTC33_Q101G
TTC33_H125Q
TTC33_R135H
TTC33_R135P
TTC33_R148Q
TTC33_H155A
TTC33_H155Y
TTC33_E162G
TTC33_E166K
effect on association with TTC33:
strong
mild
no effect
Interface
TTC33:PHF5A
TTC33:WDR61
f
TTC33 mRNA 3'UTR
TTC33 endogenous
oligo 1
MBP-TTC33 / TTC33-EGFP
EGFP-TTC33_codon_optimized
TTC33 mRNA ORF
oligo 2
tetracycline 3 days
tetracycline 6 days
fold-change in TTC33 mRNA 3'UTR level above control
fold-change in TTC33 mRNA ORF level above control
HEK293 miTTC33 MBP-TTC33 TTC33-EGFP EGFP-TTC33_codon_optimized
g
MG132 5 hours concentration [μM]:
MBP-TTC33
HEK293
EGFP-TTC33_co*
0 10 20
MBP-TTC33
EGFP-TTC33_co
TTC33
α-tubulin
*co - codon_optimized
h
TTC33-EGFP / EGFP-TTC33_codon_optimized
HEK293 Flp-In T-REx adherent cells
INPUT
ELUTION IP-EGFP
TTC33-EGFP:
EGFP-TTC33_co*:
TTC33 + tag
PHF5A
WDR61
CCDC97
UNG2
UNG1
PPP2R2A
baits
prey
*co - codon_optimized

Supplementary Figure 6. Structural and functional characterization of TTC33 interactions with TAN-associated factors. a Close-up views of the experimental structures of human WDR61 in complex with the PAFc scaffold CTR9 (*left*; 6gmh[8]), yeast Ski8 binding to SKIc scaffold Ski3 (*middle*; 4buj[9]), and TTC37 scaffold of human SKIc in complex with its second WDR61 subunit (*right*; 7qdy[10]; for the other WDR61 subunit see Fig. 8b). **b** CoIP-WB analysis of proteins co-purifying with EGFP-tagged TTC33 full-length or its fragments: TPR1-2, TPR2-3, and TPR3-C-terminus. Endogenous WDR61 was assessed along with co-transfected MBP-CCDC97 (*top*) or MBP-PPP2R2A (*bottom*). **c** CoIP-WB analysis of proteins co-purifying with EGFP-WDR61 WT or W20C. Blots were probed for SKI2W (helicase subunit of SKIc) or CTR9 (scaffolding subunit of PAFc). **d** CoIP-WB analysis of proteins co-purifying with MBP-TTC33. Blots were probed for endogenously expressed WDR61 or co-transfected EGFP-WDR61 WT or W20C mutant. **e** The table summarizes TTC33 fragmentation and mutation assays presented in Fig. 7g,i, Supplementary Fig. 5c, and Supplementary Fig. 6b. **f** Graphs showing results of RT-qPCR performed to evaluate the levels of endogenous *TTC33* mRNA in cells overexpressing MBP-TTC33, TTC33-EGFP, or EGFP-TTC33_*codon_optimized*. Two oligonucleotide pairs were used. The left panel shows amplification using the 3'UTR, which is specific only to the endogenous *TTC33* mRNA transcript. The right panel shows amplification using primers recognizing the TTC33 open reading frame, which is shared by the endogenous mRNA, and by MBP-TTC33 and TTC33-EGFP constructs, but not by the EGFP-TTC33_*codon_optimized*. **g** Western blot analysis of whole-cell extracts for control HEK293 and cells expressing either MBP-TTC33 or EGFP-TTC33_*codon_optimized*. Additionally, cells were exposed to 10 or 20 μM of the proteasome inhibitor MG132 for 5 hours to test if the levels of endogenous TTC33 downregulated as a result of MBP-/EGFP-TTC33 overexpression could be rescued. **h** Western blot analysis of co-IP experiments performed using either TTC33-EGFP (previously used for cLF-MS) or EGFP-TTC33_*codon_optimized* as bait. As expected, EGFP-TTC33_*codon_optimized* was expressed at much higher levels than TTC33-EGFP, and consequently was more abundant in the elution. Given this, as expected it precipitated more PHF5A and CCDC97. However, compared to TTC33-EGFP, the more efficiently produced EGFP-TTC33 version associated with lower amounts of UNG1/2 and PPP2R2A. WDR61 levels are equal in the eluates, but compared to (or otherwise – normalized to) the bait amount, the EGFP-TTC33_*codon_optimized* precipitated also less WDR61 than TTC33-EGFP. We thus conclude that transferring the EGFP tag from the C- to the N-terminus of TTC33 resulted in altered TAN factor binding efficiency. Source data are provided as a Source Data file (or appended to this document).

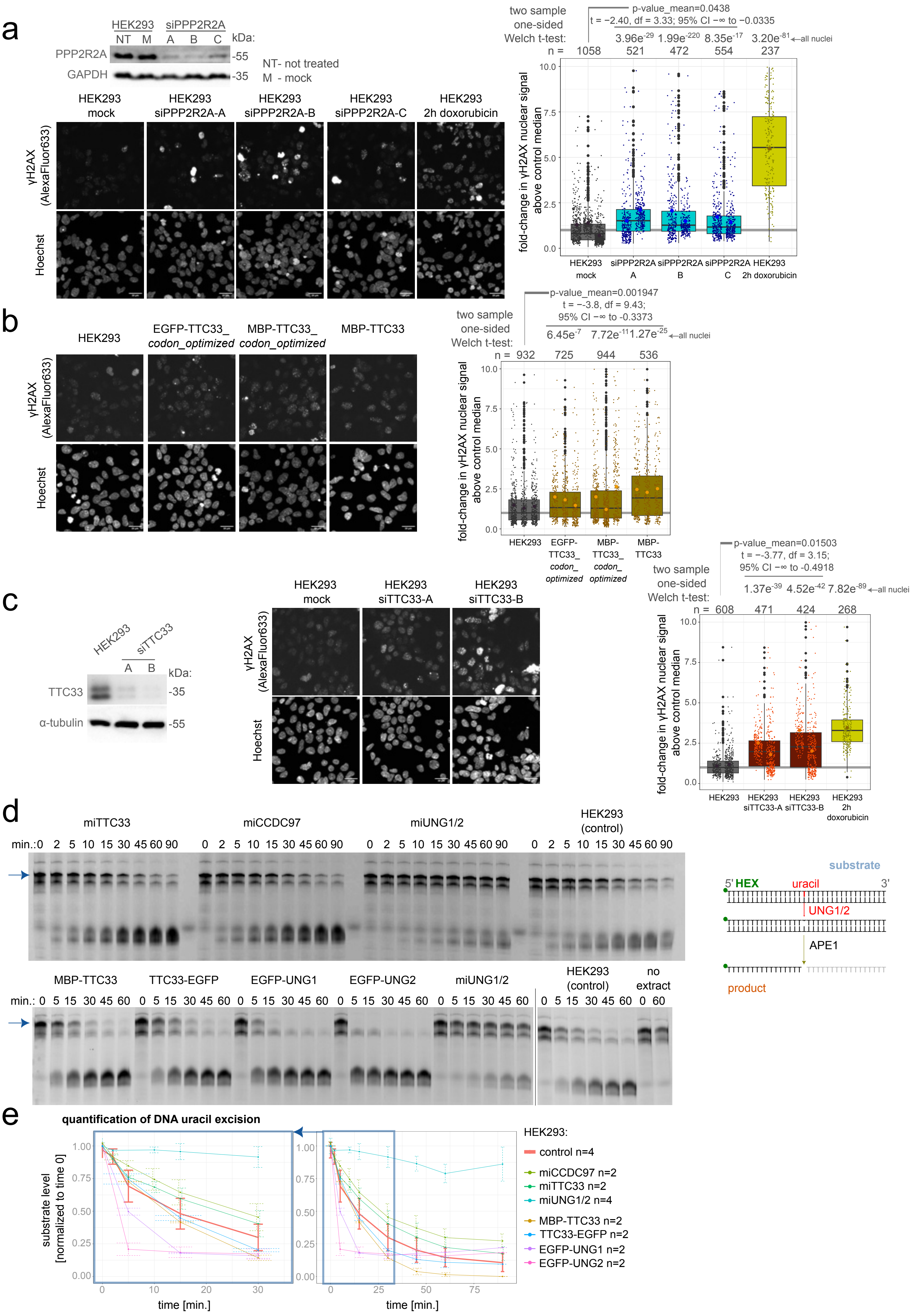
a
HEK293
NT M
siPPP2R2A
A B C
kDa:
PPP2R2A -55
GAPDH -35
NT- not treated
M - mock
HEK293 mock
HEK293 siPPP2R2A-A
HEK293 siPPP2R2A-B
HEK293 siPPP2R2A-C
HEK293 2h doxorubicin
γH2AX (AlexaFluor633)
Hoechst
two sample one-sided Welch t-test:
p-value_mean=0.0438
t = −2.40, df = 3.33; 95% CI −∞ to −0.0335
3.96e-29 1.99e-220 8.35e-17 3.20e-81 ←all nuclei
n = 1058 521 472 554 237
fold-change in γH2AX nuclear signal above control median
HEK293 mock
siPPP2R2A A
siPPP2R2A B
siPPP2R2A C
HEK293 2h doxorubicin
b
HEK293
EGFP-TTC33_ codon_optimized
MBP-TTC33_ codon_optimized
MBP-TTC33
γH2AX (AlexaFluor633)
Hoechst
two sample one-sided Welch t-test:
p-value_mean=0.001947
t = −-3.8, df = 9.43;
95% CI −∞ to -0.3373
6.45e-7 7.72e-11 1.27e-25 ←all nuclei
n = 932 725 944 536
fold-change in γH2AX nuclear signal above control median
HEK293
EGFP-TTC33_ codon_ optimized
MBP-TTC33_ codon_ optimized
MBP-TTC33
c
HEK293
siTTC33
A B
kDa:
TTC33 -35
α-tubulin -55
HEK293 mock
HEK293 siTTC33-A
HEK293 siTTC33-B
γH2AX (AlexaFluor633)
Hoechst
two sample one-sided Welch t-test:
p-value_mean=0.01503
t = −-3.77, df = 3.15;
95% CI −∞ to -0.4918
1.37e-39 4.52e-42 7.82e-89 ←all nuclei
n = 608 471 424 268
fold-change in γH2AX nuclear signal above control median
HEK293
HEK293 siTTC33-A
HEK293 siTTC33-B
HEK293 2h doxorubicin
d
miTTC33
miCCDC97
miUNG1/2
HEK293 (control)
min.: 0 2 5 10 15 30 45 60 90
MBP-TTC33
TTC33-EGFP
EGFP-UNG1
EGFP-UNG2
miUNG1/2
HEK293 (control)
no extract
min.: 0 5 15 30 45 60
substrate
5' HEX uracil 3'
UNG1/2
APE1
product
e
quantification of DNA uracil excision
substrate level [normalized to time 0]
time [min.]
HEK293:
control n=4
miCCDC97 n=2
miTTC33 n=2
miUNG1/2 n=4
MBP-TTC33 n=2
TTC33-EGFP n=2
EGFP-UNG1 n=2
EGFP-UNG2 n=2

Supplementary Figure 7. Effects of TTC33 and TAN-associated factors on DNA damage response and glycosylase activity. a The top left panel shows western blot analysis of PPP2R2A knock-down in HEK293 cells using three different siRNAs. The bottom left panel shows immunofluorescence (IF) images of γH2AX (*top row*) in nuclei (*bottom row*) of control HEK293 and PPP2R2A-depleted cells using three different siRNAs. The graph on the right shows the quantitative analysis of the IF data. Box plots combine data from all replicates and display the median along with the 25th and 75th percentiles; whiskers indicate the 5th and the 95th percentile. Small dots show the level of signal normalized to the control median in individual nuclei. Individual nuclei are grouped by biological replicate and the mean of each replicate is indicated as a large dot. The number of observations (nuclei) and the result of a one-tailed Welch two-sample test for all nuclei combined or for the replicate means are indicated above the graph. **b** The left panel shows IF images of γH2AX (*top row*) in nuclei (*bottom row*) of control HEK293 and cells overexpressing TTC33. N-terminal EGFP or MBP tags were used because they provided higher expression. The protein was expressed from an original open reading frame or a codon-optimized one, as indicated on the panel. The graph on the right shows the quantitative analysis of the IF data as in panel a. **c** The left panel shows western blot analysis of TTC33 knock-down in HEK293 cells using two different siRNAs. The middle panel shows IF images of γH2AX (*top row*) in nuclei (*bottom row*) of control HEK293 and cells knocked-down for TTC33. The graph on the right shows quantitative analysis of nuclear γH2AX as in panel a. **d** Results of UNG glycosylase assays performed on whole cell extracts obtained from control HEK293 or cells depleted of or overexpressing TTC33, CCDC97, or UNG1/2. The scheme placed next to the top gel image explains the principle of the assay. A fluorescently labelled double-stranded oligo containing uracil facing adenosine in the middle is incubated with the whole cell extract. Removal of the uracil by a DNA glycosylase, such as UNG1/2, leads to the formation of an abasic site, which recruits the APE1 endonuclease. This leads to the cleavage of one strand and the formation of a short single-stranded oligo as the final product. **e** Quantification of substrate levels during glycosylase assays shown in panel c, along with other biological replicates. Error bars represent deviation from the mean, while 'n' indicates the number of biological replicates analyzed quantitatively. A complete time-course (0-90 min.) is shown on the right, along with a close-up of the beginning of the reaction (0-30 min.) on the left. Source data are provided as a Source Data file (or appended to this document).

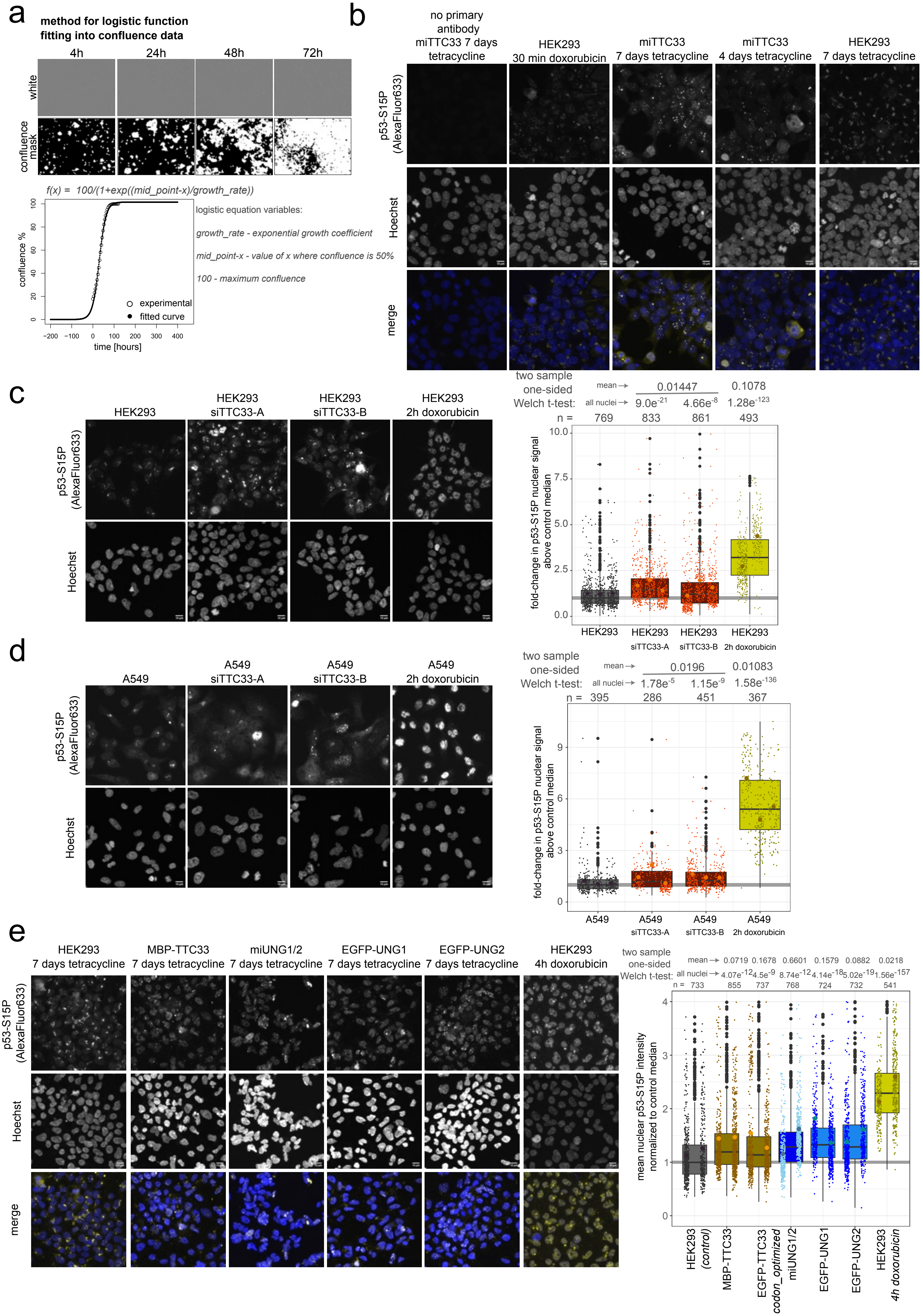

a
method for logistic function fitting into confluence data
4h
24h
48h
72h
white
confluence mask
f(x) = 100/(1+exp((mid_point-x)/growth_rate))
logistic equation variables:
growth_rate - exponential growth coefficient
mid_point-x - value of x where confluence is 50%
100 - maximum confluence
experimental
fitted curve
confluence %
time [hours]
b
no primary antibody miTTC33 7 days tetracycline
HEK293 30 min doxorubicin
miTTC33 7 days tetracycline
miTTC33 4 days tetracycline
HEK293 7 days tetracycline
p53-S15P (AlexaFluor633)
Hoechst
merge
c
HEK293
HEK293 siTTC33-A
HEK293 siTTC33-B
HEK293 2h doxorubicin
two sample one-sided Welch t-test:
mean → 0.01447 0.1078
all nuclei → 9.0e-21 4.66e-8 1.28e-123
n = 769 833 861 493
fold-change in p53-S15P nuclear signal above control median
d
A549
A549 siTTC33-A
A549 siTTC33-B
A549 2h doxorubicin
two sample one-sided Welch t-test:
mean → 0.0196 0.01083
all nuclei → 1.78e-5 1.15e-9 1.58e-136
n = 395 286 451 367
e
HEK293 7 days tetracycline
MBP-TTC33 7 days tetracycline
miUNG1/2 7 days tetracycline
EGFP-UNG1 7 days tetracycline
EGFP-UNG2 7 days tetracycline
HEK293 4h doxorubicin
two sample one-sided Welch t-test:
mean → 0.0719 0.1678 0.6601 0.1579 0.0882 0.0218
all nuclei → 4.07e-12 4.5e-9 8.74e-12 4.14e-18 5.02e-19 1.56e-157
n = 733 855 737 768 724 732 541
mean nuclear p53-S15P intensity normalized to control median
HEK293 (control)
MBP-TTC33
EGFP-TTC33 codon_optimized
miUNG1/2
EGFP-UNG1
EGFP-UNG2
HEK293 4h doxorubicin

Supplementary Figure 8. Characterization of TTC33 and TAN factor effects on p53-S15 phosphorylation and DNA damage signaling. a The top panel shows selected images of cells acquired using the IncuCyte S3 imaging system (*top row*) converted into a confluence mask (*bottom row*). The bottom graph shows an example of the outcome of logistic curve fitting into the experimental percentage confluence data obtained for one of the control HEK293 cell line experiments. Empty circles indicate experimental data, whereas solid dots show the fitted logistic line. The raw confluence data and the exact script to fit a logistic function into experimental data is given in Supplementary Dataset 3. **b** Images of immunolocalization of p53-S15P in control HEK293 cells compared to cells knocked down for TTC33 for 4 or 7 days using microRNAs, or HEK293 cells treated with doxorubicin for 30 min. To ensure the specificity of the signal, miTTC33 cells were probed with only the secondary antibody, which revealed no significant signal. Cells were stained with Hoechst to visualize the nuclei, and merged signals were shown for comparison. Assay quantification is shown in Fig. 10c. **c** Images on the left panel show the subcellular distribution of p53-S15P (*top row*) in Hoechst-stained nuclei (*bottom row*) of HEK293 control, and cells knocked-down for TTC33 using two siRNAs. Additionally, control cells were treated with doxorubicin to induce DNA DSBs and reveal the phenotype of p53-S15P localization in response to DNA damage. The graph on the right shows quantification of the result. Box plots combine data from all replicates and display the median along with the 25th and 75th percentiles; whiskers indicate the 5th and 95th percentile. Small dots show the level of signal normalized to the control median in individual nuclei. These are grouped by biological replicate, and the mean of each replicate is indicated as a large dot of matching color. The number of nuclei and the result of a one-tailed Welch two-sample test, for all nuclei combined and for biological replicates, are indicated above the graph. **d** Images on the left show the subcellular distribution of p53-S15P (*top row*) in Hoechst-stained nuclei (*bottom row*) of A549 control, and cells knocked-down for TTC33 using two siRNAs. Additionally, control cells were treated with doxorubicin to induce DNA DSBs and reveal the phenotype of p53-S15P localization in response to DNA damage. The graph on the right shows quantification of the result over three biological replicates as described for panel c. **e** The left panel shows images of immunolocalization of p53-S15P in control HEK293 cells compared to cells knocked-down for UNG1/2 or overexpressing MBP-TTC33, EGFP-UNG1, or EGFP-UNG2. As indicated, cells were treated with tetracycline for 7 days or with doxorubicin for 4 hours. Cells were stained with Hoechst to visualize the nuclei, and merged signals were shown for comparison. The graph on the right shows quantification from two biological replicates, quantified as described in panel c. Source data are provided as a Source Data file (or appended to this document).

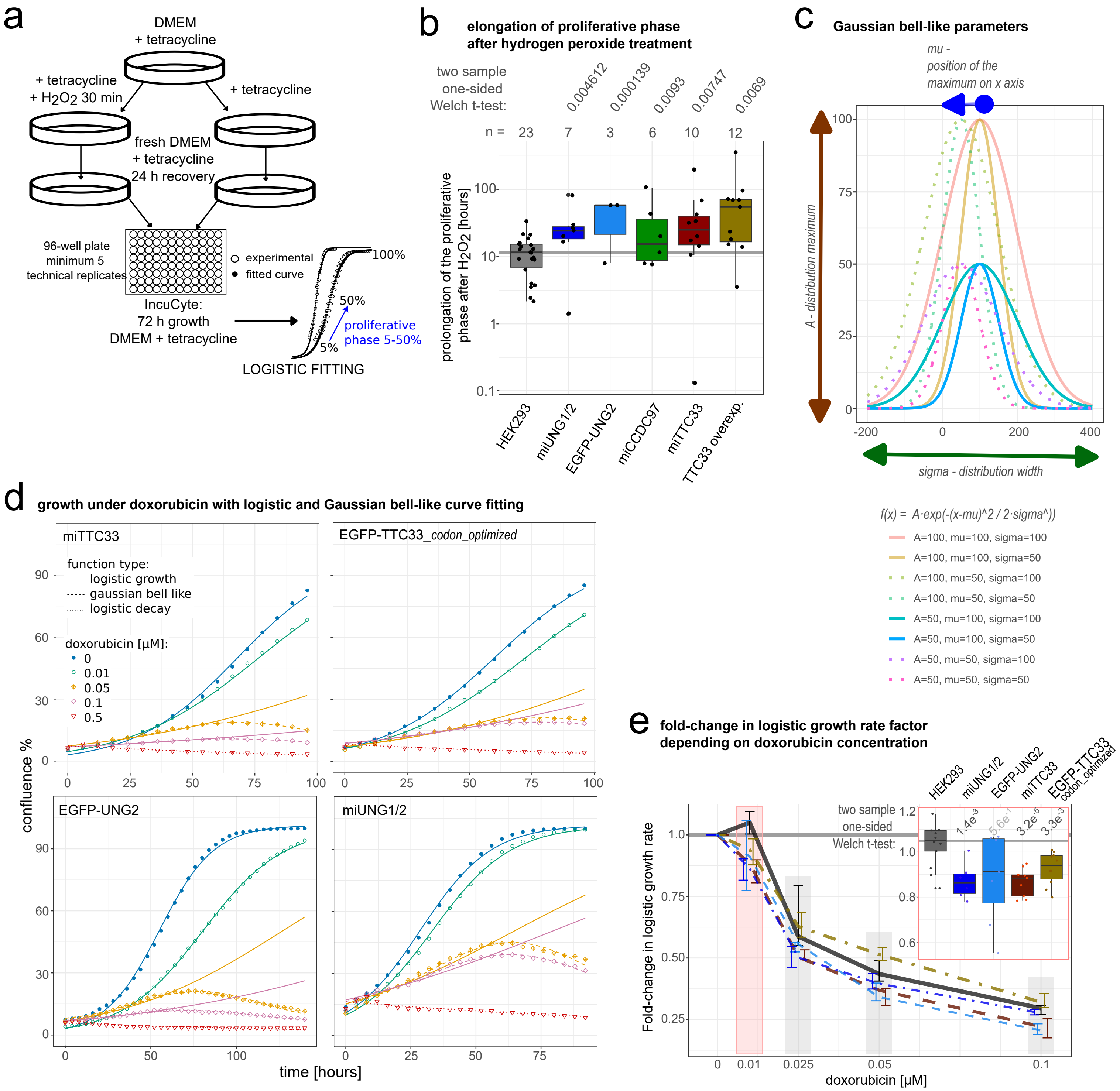

f

KIRC: TAN expression and stage or 5-year survival

protein	expression	n (%)	stage, n (%) early (I-II)	stage, n (%) late (III-IV)	no data	p-value	OR (95% CI)	χ2	5-years survival, n (%) < 5	5-years survival, n (%) ≥ 5	p-value	OR (95% CI)	χ2
CCDC97	high<br>low	314 (60.3)<br>207 (39.7)	205 (65.3)<br>110 (53.1)	106 (33.8)<br>97 (46.9)	3 (0.9)<br>0 (0.0)	0.0044	1.7054<br>(1.1900-2.4441)	0.0036	228 (72.6)<br>145 (70.0)	86 (27.4)<br>62 (30.0)	0.5521	0.8821<br>(0.5989-1.2993)	0.5263
PHF5A	high<br>low	351 (67.4)<br>170 (32.6)	228 (64.9)<br>87 (51.2)	121 (34.5)<br>82 (48.2)	2 (0.6)<br>1 (0.6)	0.0029	1.7760<br>(1.2223-2.5805)	0.0026	240 (68.4)<br>133 (78.2)	111 (31.6)<br>37 (21.8)	0.0225	1.6625<br>(1.0835-2.5510)	0.0176
PPP2R2A	high<br>low	369 (70.8)<br>152 (29.2)	239 (64.8)<br>76 (50.0)	129 (34.9)<br>74 (48.7)	1 (0.3)<br>2 (1.3)	0.0029	1.8040<br>(1.2274-2.6514)	0.0027	255 (69.1)<br>118 (77.6)	114 (30.9)<br>34 (22.4)	0.0546	1.5516<br>(0.9985-2.4110)	0.0465
TTC33	high<br>low	272 (52.2)<br>249 (47.8)	188 (69.1)<br>127 (51.0)	83 (30.5)<br>120 (48.2)	1 (0.4)<br>2 (0.8)	< 0.0001	2.1402<br>(1.4945-3.0650)	0.0000	175 (64.3)<br>198 (79.5)	97 (35.7)<br>51 (20.5)	0.0001	2.1519<br>(1.4493-3.1953)	0.0001
UNG1/2	high<br>low	351 (67.4)<br>170 (32.6)	233 (66.4)<br>82 (48.2)	117 (33.3)<br>86 (50.6)	1 (0.3)<br>2 (1.2)	0.0001	2.0886<br>(1.4351-3.0397)	0.0001	246 (70.1)<br>127 (74.7)	105 (29.9)<br>43 (25.3)	0.3008	1.2606<br>(0.8329-1.9081)	0.2699
WDR61	high<br>low	301 (57.8)<br>220 (42.2)	211 (70.1)<br>104 (47.3)	88 (29.2)<br>115 (52.3)	2 (0.7)<br>1 (0.4)	< 0.0001	2.6513<br>(1.8431-3.8139)	0.0000	206 (68.4)<br>167 (75.9)	95 (31.6)<br>53 (24.1)	0.0627	1.4531<br>(0.9807-2.1530)	0.0604

OR - odds ratio CI - confidence interval

Supplementary Figure 9. TTC33-dependent regulation of cellular recovery following oxidative and genotoxic stress. a Scheme of the experiment designed to assess the efficiency of cell line recovery after hydrogen peroxide treatment. Cells were treated with tetracycline for 3 or 6 days to ensure microRNA or protein expression. Cells were exposed to H_2O_2 for 30 min. After that time, hydrogen peroxide was removed and replaced with fresh medium containing tetracycline. Cells were left to recover for 24 hours, after which they were seeded in a 96-well plate to assess growth for another 3 days. Logistic fitting was used to obtain a collection of growth rate coefficients. The time required for cells to proliferate from 5% to 50% confluence was calculated for control and hydrogen peroxide-treated cells, and the difference is shown in Supplementary Fig. 9b. **b** The graph shows the sensitivity of HEK293 control and cells depleted of or overexpressing TTC33, CCDC97, or UNG1/2 to reactive oxygen species (ROS) induced by hydrogen peroxide treatment. Individual dots indicate the additional time required for each cell line to proliferate from a confluence of 5% to 50% as a result of exposure to ROS. The growth of control cells was prolonged by 11 hours on average due to ROS, while for all other cell lines this value was two-fold higher, indicating increased sensitivity. **c** The graph illustrates the properties of the Gaussian bell-like function parameters. In short, A controls the maximum of the distribution, sigma regulates the width of the curve, and mu designates the position of the maximum. Importantly, none of these parameters directly reflects the logistic growth rate. **d** Exemplary data assessing the impact of TAN factors on the proliferative response of HEK293 cells to doxorubicin. The graphs complement the HEK293 control data shown in main Fig. 10f. Points depict experimental confluence data for cells knocked-down or overexpressing TTC33, depleted of UNG1/2, or overexpressing EGFP-UNG2. Each cell line was grown in control conditions or under 0.01, 0.05, 0.1, or 0.5 μM doxorubicin concentrations. Various lines represent the logistic growth/decay or Gaussian bell-like functions fitted to the raw data. Briefly, control cells and those exposed to 0.01 or 0.025 μM doxorubicin were modeled using logistic growth. Those at 0.5 μM more accurately followed logistic decay. The intermediate concentrations of 0.1 and 0.05 μM produced curves characteristic of initial growth and then population death. The initial growth phase (until the 65th hour) was modeled with logistic growth, while the full experimental growth was described by a Gaussian bell-like distribution (properties of this distribution are explained in panel c). **e** The graph shows, as lines, the changes in the median fold change of the logistic growth rates as a function of doxorubicin concentration for HEK293 control, and cells depleted of or overexpressing TTC33 or UNG2. Error bars indicate the the 25th and 75th percentiles. The raw values are listed in Supplementary Dataset 3. The bar plot in the top right corner shows an enlarged view of the fold-change distribution at 0.01 μM doxorubicin concentration. Individual points represent biological replicates. The fold-change was calculated separately for each biological replicate. **f** The table summarizes the associations between TAN factor mRNA expression (CCDC97, PHF5A, PPP2R2A, TTC33, UNG1/2, and WDR61) and cancer stage or 5-year survival in KIRC patients. Patients were stratified into high- and low-expression groups. Associations between expression status and clinical outcome were assessed using Fisher's exact test (two-sided). Odds ratios (OR) with 95% confidence intervals (CI) are reported. P values are shown for each comparison. Expression group counts (n) are indicated in the table. OR, odds ratio; CI, confidence interval. Source data are provided as a Source Data file (or appended to this document).

Supplementary Table 1. List of the highest-ranking Swiss-Model templates for structural modeling of TTC33. The 'Template PDB code' column indicates the structure used as template, while the codes in parentheses indicate alternative models of the same protein. Those most often differ in ligand content. Only the highest-ranking model of the collection was analyzed. Alternative models are listed in order proposed by Swiss-Model.

RANK	Template PDB code<br><br>(variants)	Coverage (graphical: TTC33 region covered by model; numerical: identity coverage)	I (S)[1]	GMQE [template search (model)]	QMEAN	Method (Resol. Å)	Oligo-meric state<br><br>(ligand)	RMSD to TTC33 Alpha Fold3 model	Reference<br><br>DOI<br><br>(description)
1	8chy.1.A<br><br>(8ot7.1.A)	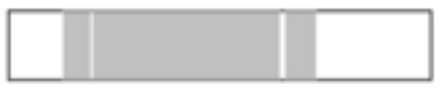<br>0.59	23.33 (0.32)	0.39 (0.41)	0.73±0.07	X-ray (2)	monomer<br><br>(13xZn)	1.78	Liutkus et al., 2024[11]<br><br>https://doi.org/10.1002/pro.4971<br><br>(synthetic model 8 repeat TPR protein with zinc)
2	9qhc.1.A<br><br>(9qhc.2.A 9qhb.1.A)	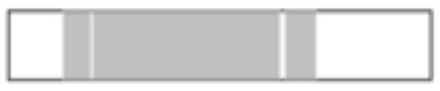<br>0.58	22.88 (0.32)	0.39 (0.40)	0.70±0.07	X-ray (2.88)	monomer<br><br>(1xCa)	1.7	Liutkus et al., 2025[12]<br><br>https://doi.org/10.1002/anie.202516635<br><br>(natural: 8-repeat CTPR protein; *Escherichia coli*)
3	5npr.1.A	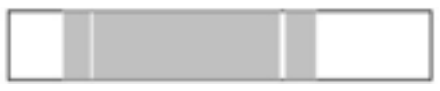<br>0.57	22 (0.31)	0.37 (0.39)	0.68±0.07	X-ray (1.8)	monomer<br><br>1xLig.[2]	1.91	Rafie et al., 2018[13]<br><br>https://doi.org/10.1021/acs.bioconjchem.8b00194<br><br>(natural: O-GlcNAc transferase; *Homo sapiens*)
4	4j8d.1.A	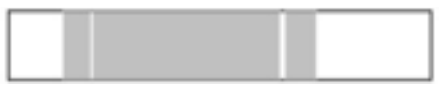<br>0.58	21.19 (0.31)	0.36 (0.39)	0.67±0.07	X-ray (2.8)	monomer<br><br>(none)	2.01	Li et al., 2013[14]<br><br>https://doi.org/10.1038/nsmb.2608<br><br>(natural: Hsc70-interacting protein; *Rattus norvegicus*)
5	4n5c.4.A<br><br>(4n5c.2.A 4n5c.3.A 4n5c.1.A 4n5c.7.A 4n5c.5.A 4n5c.6.A 4n5c.8.A)	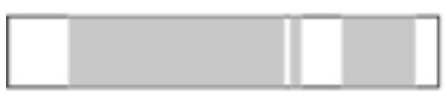<br>0.76	11.50 (0.25)	0.38 (0.35)	0.48±0.06	X-ray, (3.3)	monomer<br><br>(none)	18.35	Wu et al., 2014[15]<br><br>https://doi.org/10.1016/j.devcel.2013.11.012<br><br>(natural: Ypp1; *Saccharomyces cerevisiae* S288C)

[1]I – Identity, S- Similarity

[2]Lig. - [[(2~{R},3~{S},4~{R},5~{R})-5-[2,4-bis(oxidanylidene)pyrimidin-1-yl]-3,4-bis(oxidanyl)oxolan-2-yl]methoxy-oxidanyl-phosphoryl] propyl hydrogen phosphate

Supplementary Table 2. List of vertebrate TTC33 sequences, with the core TPR domain used for structural modeling in Fig. 6c highlighted. The full-length sequences are divided into three fragments for visualization; the middle fragment corresponds to the core TPR domain used for modeling.

IDENTIFIER	SPECIES	Sequence	Used for modeling in Fig. 6c
Q6PID6	*Homo sapiens*	MASFGWKRKIGEKVSKVTSQQFEAEAADEKDVVDNDEGNWL<br>HAIKRRKEILLEGCAEKSKQLKDEGASLAENKRYREAIQKWDEALQLTPNDATLYEMKSQVLMSLHE<br>MFPAVHAAEMAVQQNPHSWESWQTLGRAQLGLGEIILAIRSFQVALHIYPMNPEIWKEDL<br>SWARTLQEQQKVAQRIKKSEAPAEVTHFSPKSIPDYDFESDEIVAVCAAIAEKEKTVSANKTMVIVSAS<br>GAIETVTEKEDGATPPDGSVFIKAR	NO<br>YES<br>YES<br>NO<br>NO
Q9D6K7	*Mus musculus*	MASFGWKRRIGEKVSKATSQQFEAEAADEKDAAENEDGNWL<br>QASKRRKETLQEGCKQRSQQLKDEGAQLAENKRYKEAIQKWDEALQLTPGDATLYEMKSQVLLSLH<br>EMFPAVHAAEMAVKRNPHSWEAWQTLGRAQLGLGEIVLAIRSFQIALHIYPMNPELWKEDL<br>SWARKLQEQQKVAQRIENKEMPPEGPDLSPGSIPDYDFESDEIVAVCAAVAEKQKSVSANKTMVIVSA<br>SGTVEIVNEKEEGSSTPDGSVFIKAR	NO<br>YES<br>YES<br>NO<br>NO
0A7K7NDE6	*Haliaeetus albicilla*	MASFGWKRKIGEKVSKATSQQFEAEAADDKDLADDDAANWV<br>HATKRRKKILVEDCVKKSKELKDEGANLAEKRRYREAIQKWDKALQLTPEDATLYEMKSQVLMSLH<br>EMFPAVHAAEMAVQRNPRSWDAWQTLGRAQLGLGEIALAIRSFQVSLHIFPMNPEVWKEDL<br>SWARKLHEQQKATKTEAVQALAKEKELSQESIPDYDFESDEIVAVCAAIAEKEKALSAAKTVVIVSAS<br>GTVETVTERENCPTAPDETLFIRAR	NO<br>YES<br>YES<br>NO<br>NO
A0A2I0LTW0	*Columba livia*	MASFGWKRKIGEKVSKATSQQFEAEAADDKDVADDDDGNWV<br>HATKRRKEALLEDCVKKSKQLKDEGASLAENRRYREAIHKWDKALQLTPEDATLYEMKSQVLMSLH<br>EMFPAVHAAEMAVQRNPRSWDAWQTLGRAQLGLGEIALAIRSFQVSLHIFPMNPEVWKEDL<br>SWARKLQEQQKATKTEEVQALAKGKALAQESIPDYDFESDEIVAVCAAIAEKEKALSAAKTVVIVSAS<br>GAVETVIEKENCPSTPDETLFIRAR	NO<br>YES<br>YES<br>NO<br>NO
A0A8C5P822	*Leptobrachium leishanense*	MASFGWKRKIGEKVSKVTSQQFEEEAADVVKKEDIDWI<br>HATKRKKEVLLEDNITKSKRLREEGIALAQNARHLEAIKVWDSAIHLTPEDASLYEMKSQALMCLDEK<br>FHAVQTAETAVQRDPHFVEAWQTLGRAQLGLGEITMAIRSFQVGLHLCPTNSELREEDL<br>TWALRLFQQKKGAVKTEERKKDCRGINEELEIIPDYDFESDEVVAACAAISQRQVASANNTGIVVSASG<br>LVTNAEQKKNDSSDSNTEMFIKAR	NO<br>YES<br>YES<br>NO<br>NO
A0A8C4C7J9	*Denticeps clupeoides*	MASFGWKRKIGEKVSKTKVQQFEDEAERLDENNPEAEGVDWL<br>HAIKRRREVLLEDCAAKSKRLKEEGAELAEHGRPWEAIKRWDEAIQLTPEEAPLYEMKSQVLNTLHEG<br>FPAVQAAETAVKLCPLWWEAWQTLGRAQLSLGEVDLAVRAFQVAIHLCPTDRTLWEEDL<br>AWALRLRDQRRSVDAKAKEEVEAQHLLDNAPELQRDYDDFESDEVIAACTAVADRQKKFEELRRTA<br>VIVDVESVTVETLQRDSDRPSAASQSQFVKARGL	NO<br>YES<br>YES<br>NO<br>NO
A0A8C7DYV4	*Naja naja*	MASFGWKRKIGEKVSKVTSQQFEAQAADEQGLVDNSEVDWL<br>QEAKRKKGLLLEDCVAQSKHLKDEGAILAENGRYQEALHKWDEALQLTPEDATLYEMKSQVLMSLH<br>EMFPAVQAAEMAVQRDPHSWEAWQTLGRAQLGLGEITLAIRSFQVALHIYPLNSGCGTKIF<br>PGQENYNNRKRQPKRRQEHREMRQKLWRGQSQIMILKAMRSWPCVLPLQKKKKALRPLKV	NO<br>YES<br>YES<br>NO
A0A8C3FLN0	*Chrysemys picta bellii*	MQLFRMASFGWKRKIGEKVSKATSQQFEAEAADDKGLVDNDDDVDWL<br>HATKRRKEILLEDCMEKSKQLKDEGARLAENRRYWEAVHKWDEALQLTPEDATLYEMKSQVLMAL<br>HEMFPAVHAAEMAIQRNPHSWEAWQTLGRAQLGLGEIGLAIRSFQVALHICPMSPELWKEDL<br>TWARKLHDQQKKATKTKAMQTDAKEADVAHESIPDYDFESDEIVAVCAAIAEKEKTVSATKTVVIV<br>SASGTVETITEKEECTTTPDDAVFIRAR	NO<br>YES<br>YES<br>NO<br>NO
Q5M990	*Xenopus laevis*	MASFGWKRKIGEKVSKEVSQHFEEEAADESVVLDSHDVDWL<br>HAIKRKKDVLLEDNVAKSKRLKEEGGLLAEDGRQKEALTKWDEAIQLTPGDAALYEMKAQVLMGVH<br>EIFPAVQAAETAVQRNPHFVEAWQTLGRAQLSLGEITMAIRSFQIGLHICPANTELWEQDL<br>NWARQLLLQKMDTESAERKRRDVNITREQIPDYDFESDEVVAACDAISQKQKMAAANKTVVVSASGS<br>ENLSDRKEDKVETNDSKEFIKAR	NO<br>YES<br>YES<br>NO<br>NO
A0A803J3U9	*Xenopus tropicalis*	MASFGWKRKIGEKVSKAVSQHFEEEAADESVVLDSHEVDWL<br>HAIKRKKGILLEDNTARSKRLKEEGSLLAEDGRHKEALTKWDEAIQLTPEDAALYEMKAQVLMGVHE<br>IFPAVQAAETAVQRNPRFVEAWQTLGRAQLGLGEITMGCTDSNIWFWTLITYYRIRVPKN<br>LGRLKMTIQRKMIGLMYIRKCSHNPHTFLLVSCTLNHLHYRYLHQNALRALSYGCALCCAVLPAFYSD<br>TATSGPACSSRVPSVPYIICRSSVVMK	NO<br>YES<br>YES<br>NO<br>NO
A0A8C5C0W3	*Gadus morhua*	MASFGWKRKVGERVSKSTLQQFESEAEKAGDRPRKEDGVDWM<br>HAIKRRREVLLEDCETKSERLKGEGALLAEEGRHWEAIKKWDEAIQLTPENHLLYEMKSQVLSILQELF<br>PAVKAAEMAVKLRPMWWEGWQTLGRAQLSLGEVDLAVRSFQVAVHLSPSDPALREQDL<br>AWAQKLQRQQRAAQERTLQEDEAQRLVLDAPELRRDYDDFESDEVLEACTAVAERQRRLEELRRTTV<br>VLDADGNVRSIVAPAGAGGAETSQAPLVKARGL	NO<br>YES<br>YES<br>NO<br>NO
Q6DI40	*Danio rerio*	MASFGWKRKVGERVSKQTVQQFEQDSEQADEDEVEREGVDWL<br>HVIKRKREVLLEDCAAKSKRLKEEGALLAEQDRNWEALKKWDEAVQLTPEDAVLYEMKSQVLITLGE<br>VFLAVQSAEMATRLRPIWWEAWQTLGRAQLSLGEVELAVRSFQVALHLHPSERPLWEEDL<br>NWALKLRENQQSLREKASQVDEARRLLVEAPELKGDYDFESDEVIEACSAMADRQKQYEDLKKKAV<br>VVDAHGEAKEMLVEGDDNPTSSSQSVLIKARGL	NO<br>YES<br>YES<br>NO<br>NO

Appendix

Generation and validation of TTC33 and CCDC97 HEK293 knockout cell lines

Verification of TTC33 or CCDC97 Crispr-Cas9 knock-outs at the protein level using western blot

membranes after transfer stained with Ponceau S red

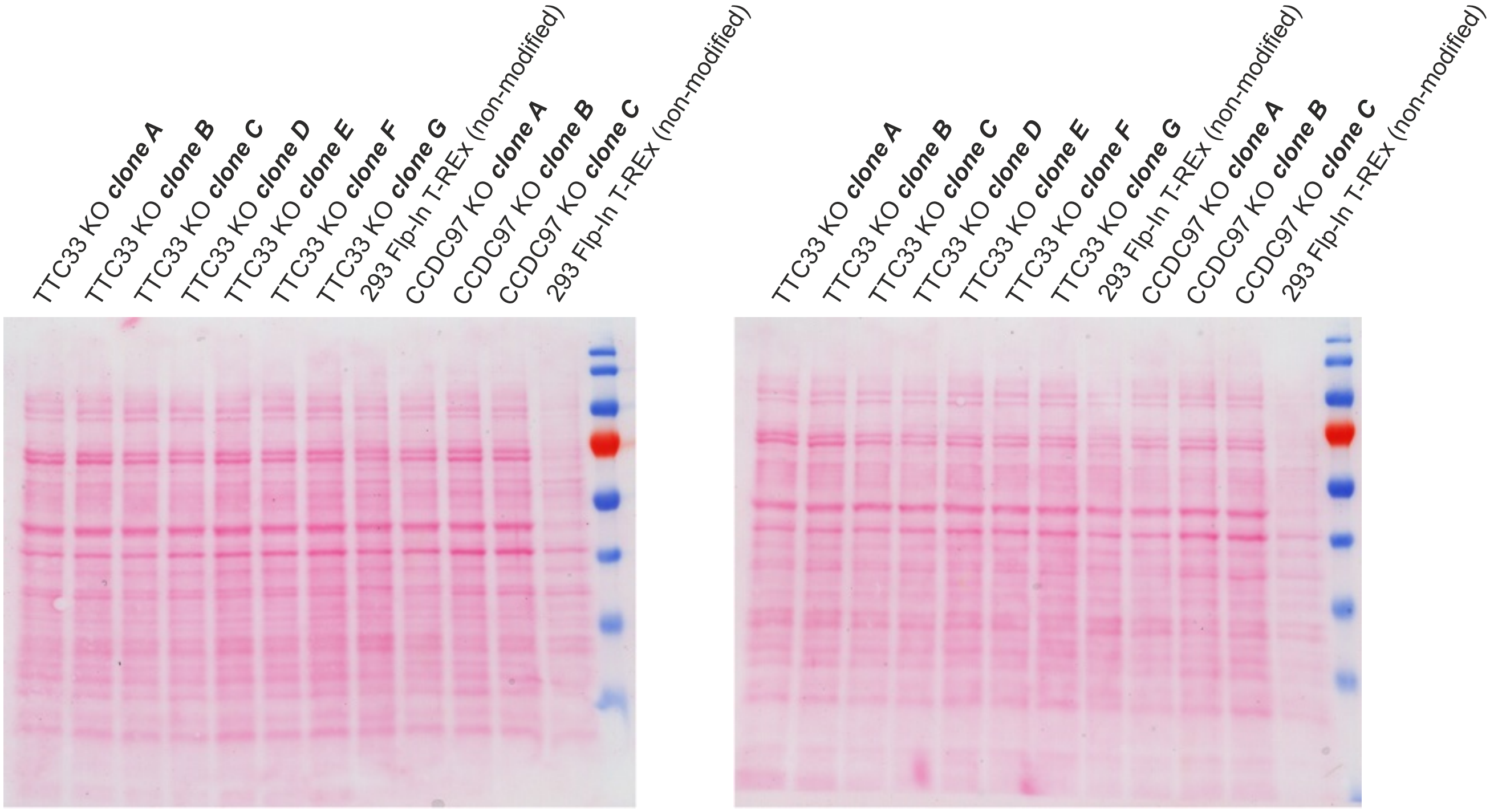

western-blot results

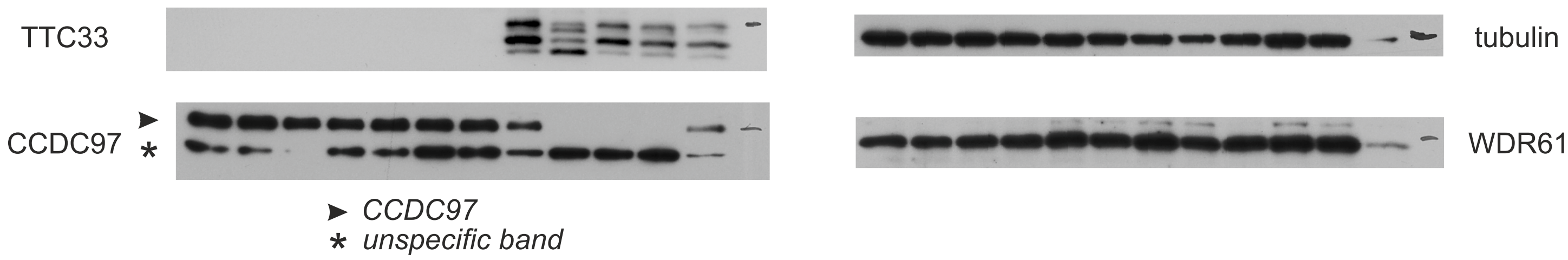

Verification of TTC33 Crispr-Cas9 knock-outs at the genomic DNA level

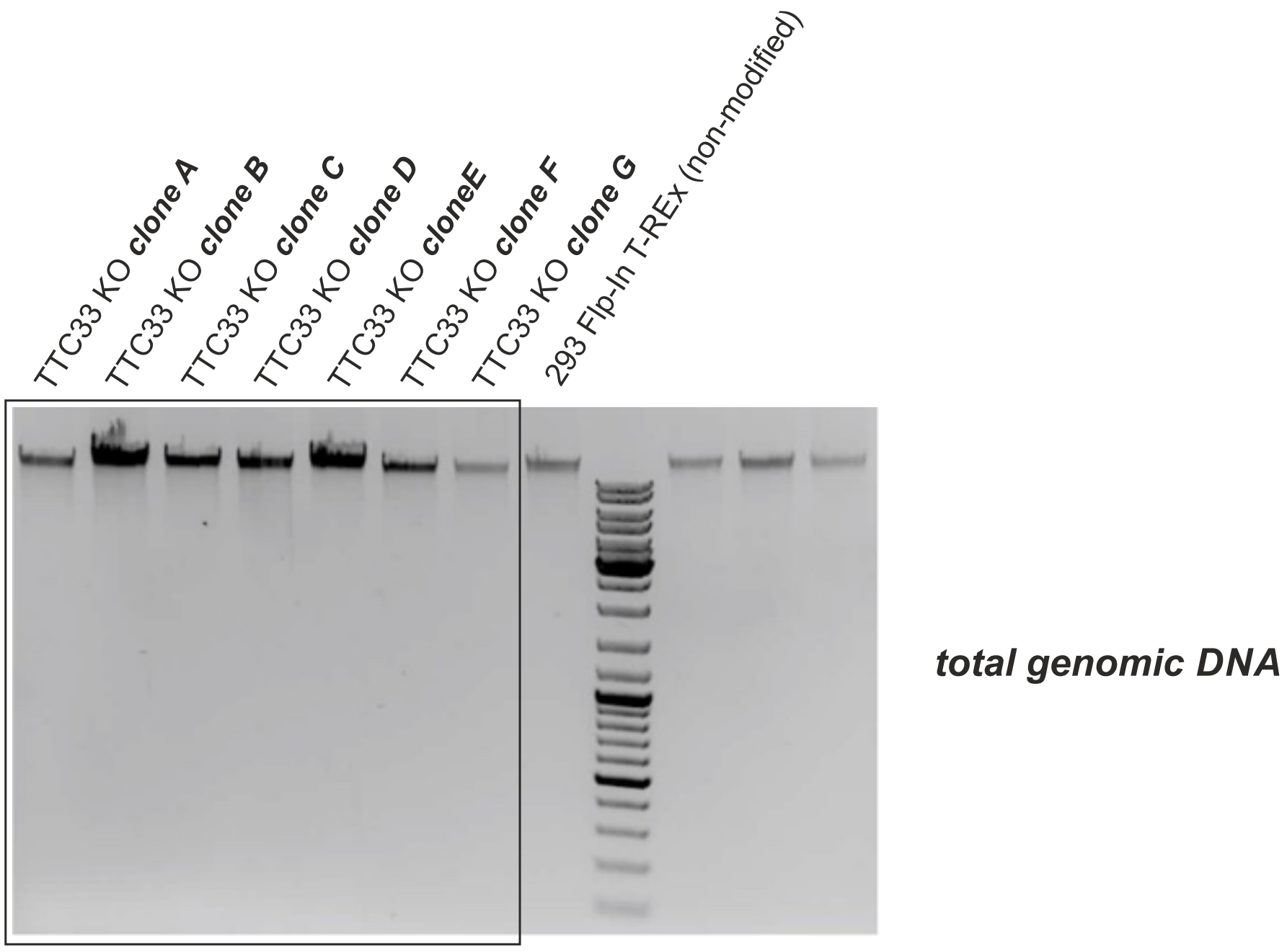

Schematic genomic organization of *TTC33* locus and other relevant information

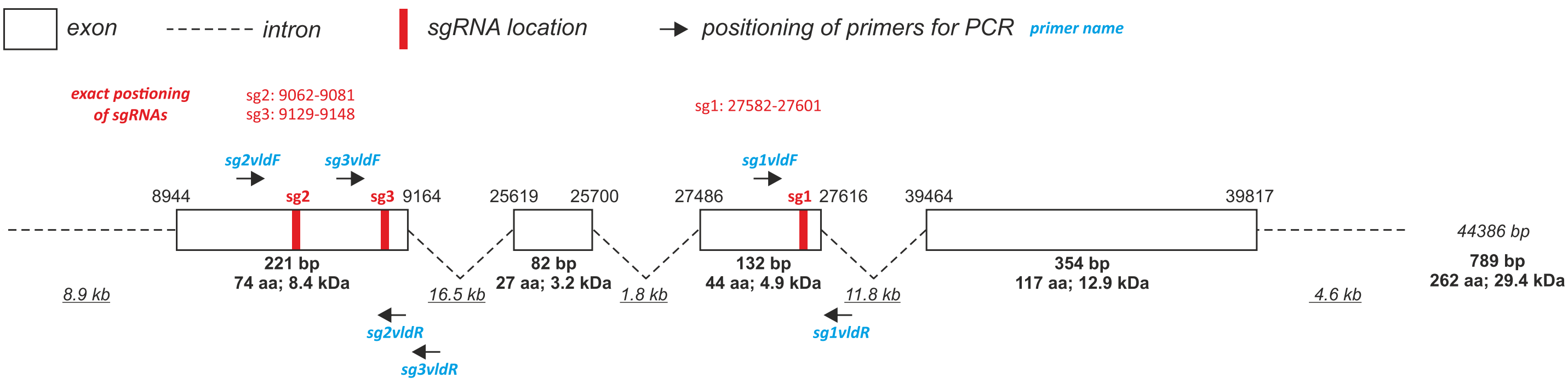

exemplary results of PCR-based validation

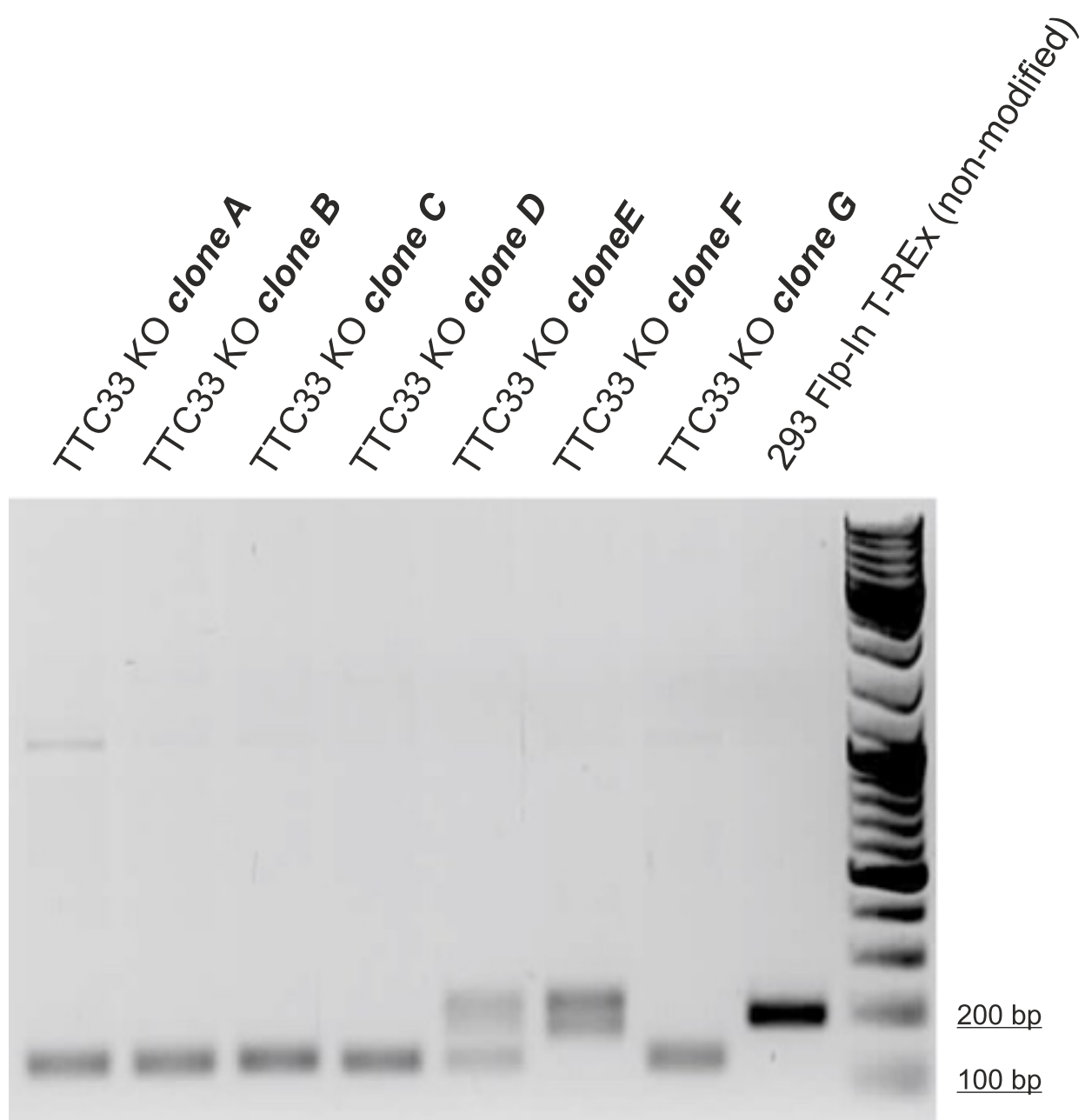

primer pair: sg3vldF-sg3vldR

predicted product size for control: 195 bp

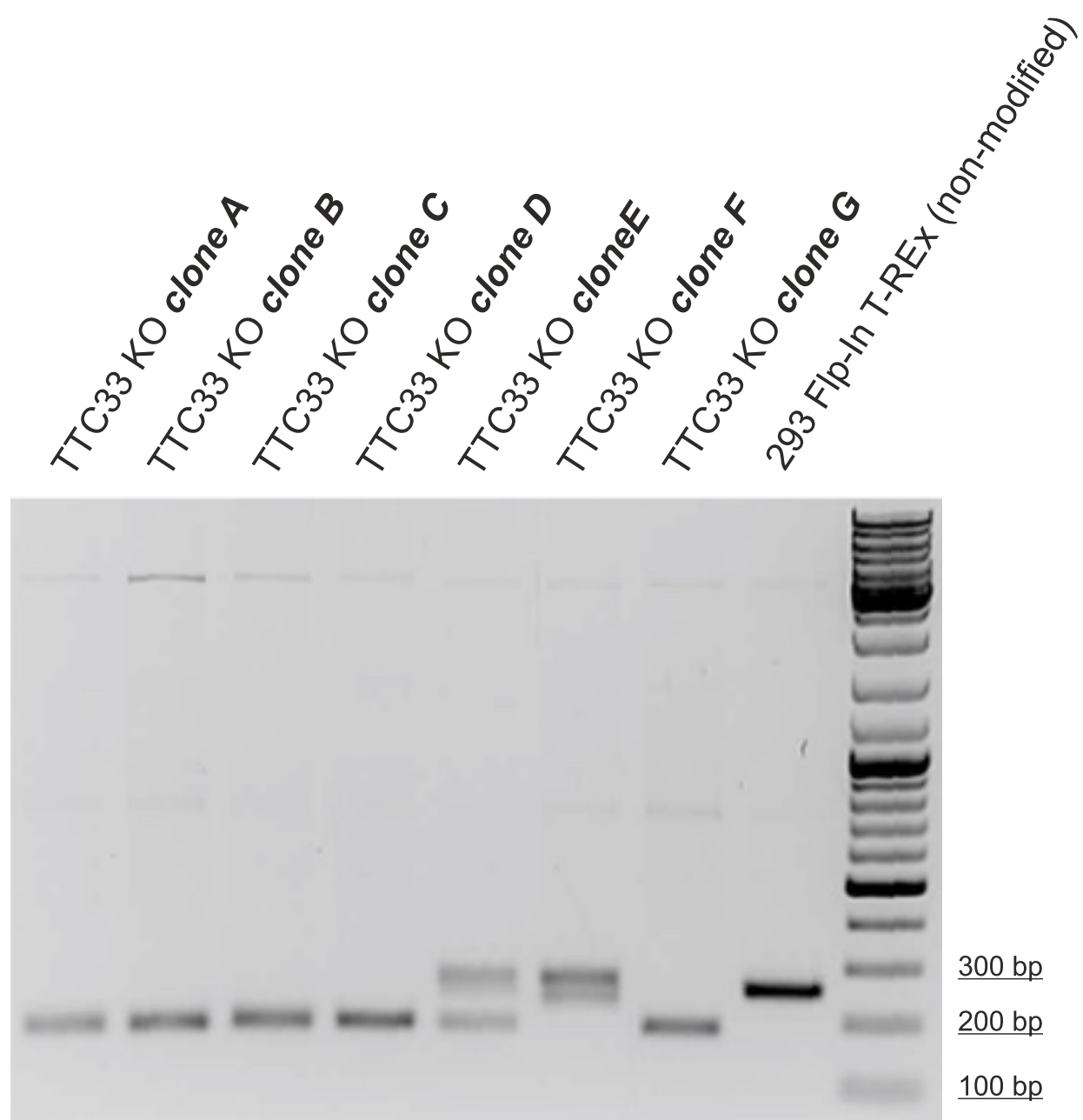

primer pair sg2vldF-sg3vldR

predicted product size for control: 260 bp

PCR products obtained using sg2vldF-sg3vldR primer pair for TTC33 KO clones A, B, C, and negative control (non-modified 293 Flp-In T-REx) were gel-purified, cloned into pCR™-Blunt II-TOPO® vector, and subjected to Sanger sequencing using M13 (-20) universal primer

Sanger sequencing of the wild-type *TTC33* locus fragment

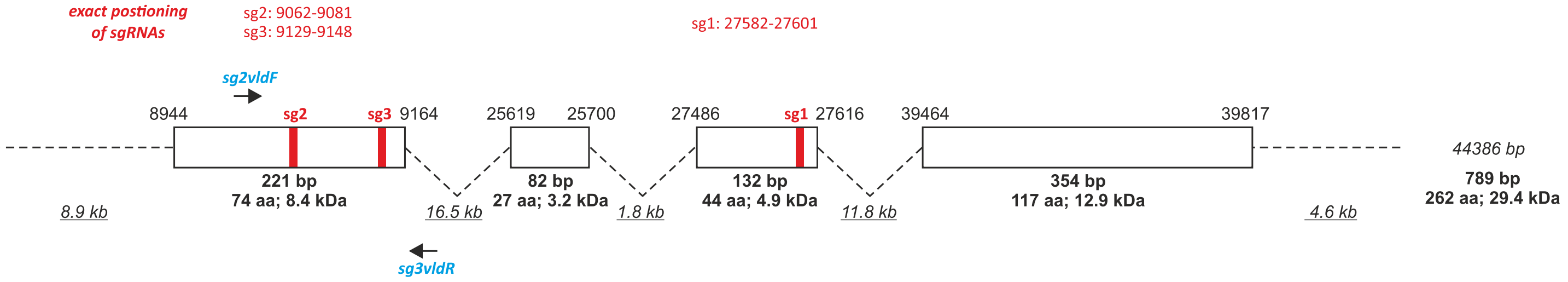

exon 1 + *intron 1-2* sequence (***reference; wild-type***); **8972-9231 (*260 bp*)**

ATGGCTTCCTTTGGGTGGAAGAGGAAAATTGGTGAGAAGGTCTCAAAGGTCACTTCCCAG sg2vldF
CAGTTTGAAGCTGAAGCTGCTGATGAGAAGGATGTAGTTGACAACGATGAAGGGAACTGG
CTTCATGCCATTAAACGTAGGAAAGAAATTCTTCTTGAAGGCTGTGCTGAGAAAAGTAAA **sg2**
CAGCTGAAGGATGAAGGAGCCAGTTTGGCTGAAAATAAAAG*gtatgtatttaaagttttt* **sg3**
tttttatggagaagagcttacattttctagctcactgtccgtaatggg sg3vldR

Topo+PCR for non-modified 293 Flp-In T-REx (***sequence read***)

260 bp

ATGGCTTCCTTTGGGTGGAAGAGGAAAATTGGTGAGAAGGTCTCAAAGGTCACTTCCCAG
CAGTTTGAAGCTGAAGCTGCTGATGAGAAGGATGTAGTTGACAACGATGAAGGGAACTGG
CTTCATGCCATTAAACGTAGGAAAGAAATTCTTCTTGAAGGCTGTGCTGAGAAAAGTAAA
CAGCTGAAGGATGAAGGAGCCAGTTTGGCTGAAAATAAAAG*gtatgtatttaaagttttt*
tttttatggagaagagcttacattttctagctcactgtccgtaatggg

8972-9231

(sequence identical to the reference one)

resulting protein amino acid sequence:
MASFGWKRKIGEKVSKVTSQQFEAEAADEKDVVDNDEGNWLHAIKRRKEILLEGCAEKSKQLKDEGASLAENK...
(**262 aa protein; 29.4 kDa**)

- source sequencing chromatogram (sequencing in forward orientation)

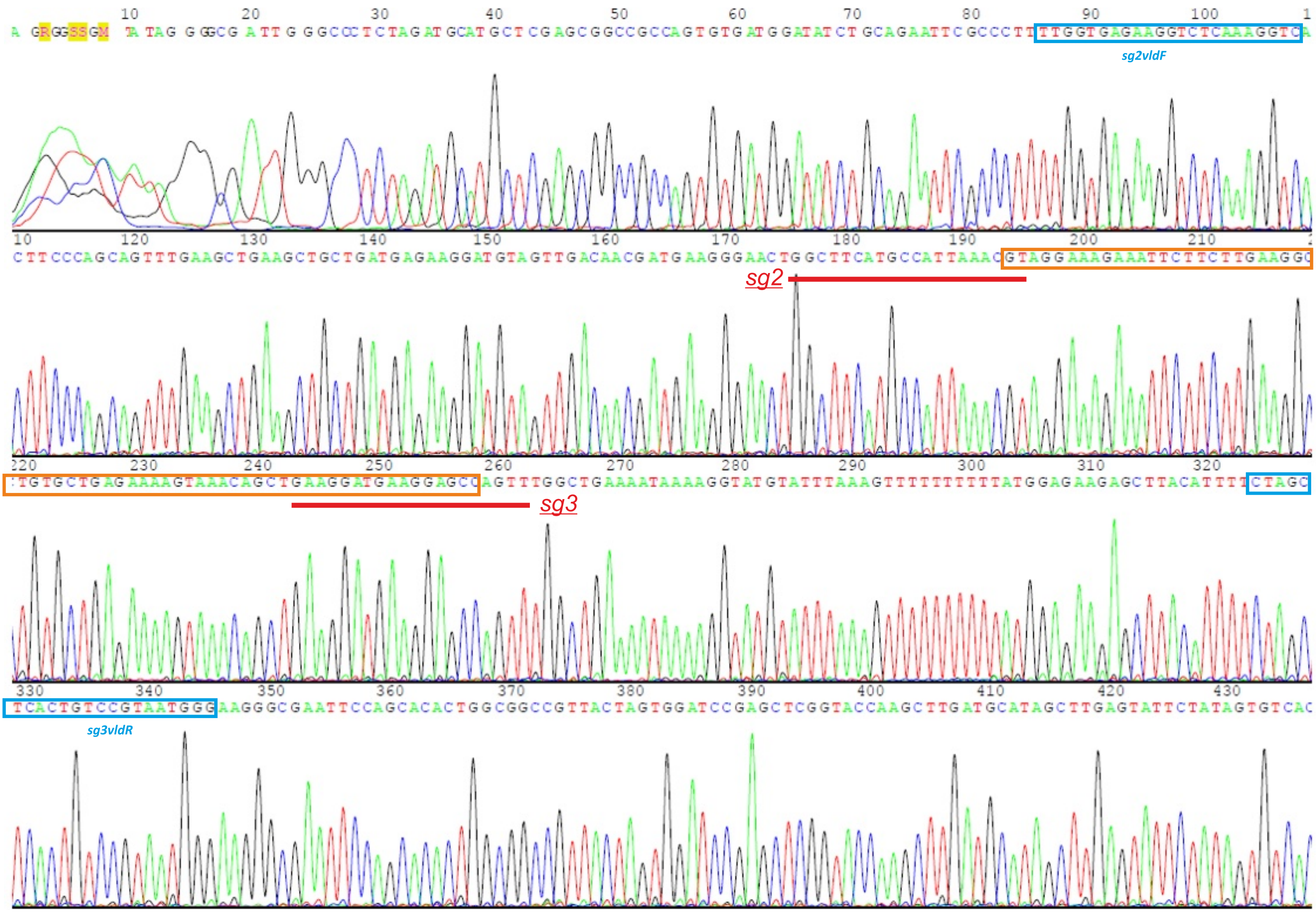

Sanger sequencing of the *TTC33* locus fragment in TTC33 KO clones A-C

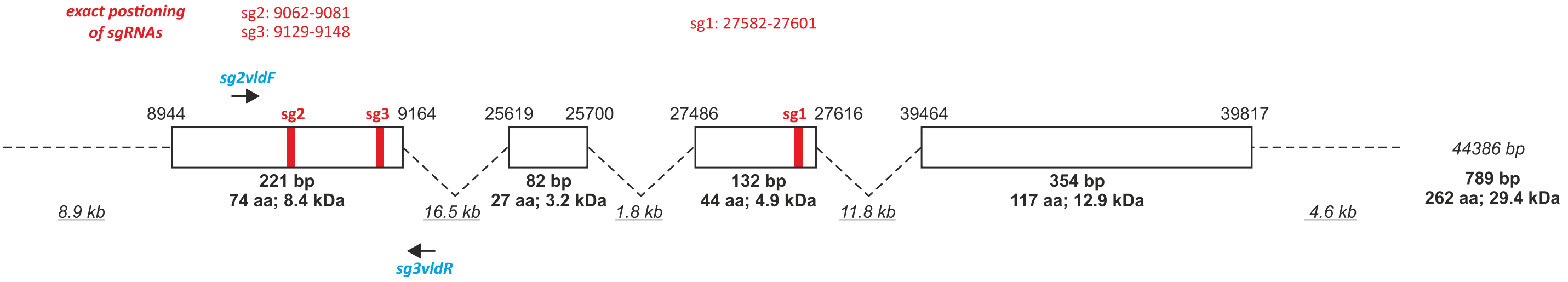

exon 1 + *intron 1-2* sequence (***reference; wild-type***)

ATGGCTTCCTTTGGGTGGAAGAGGAAAATTGGTGAGAAGGTCTCAAAGGTCACTTCCCAG — sg2vldF
CAGTTTGAAGCTGAAGCTGCTGATGAGAAGGATGTAGTTGACAACGATGAAGGGAACTGG
CTTCATGCCATTAAACGTAGGAAAGAAATTCTTCTTGAAGGCTGTGCTGAGAAAAGTAAA — **sg2**
CAGCTGAAGGATGAAGGAGCCAGTTTGGCTGAAAATAAAAG*gtatgtatttaaagttttt* — **sg3**
tttttatggagaagagcttacattttctagctcactgtccgtaatggg — sg3vldR

Topo+PCR for TTC33 KO A, B, or C (***sequence read***)

195 bp

ATGGCTTCCTTTGGGTGGAAGAGGAAAATTGGTGAGAAGGTCTCAAAGGTCACTTCCCAG
CAGTTTGAAGCTGAAGCTGCTGATGAGAAGGATGTAGTTGACAACGATGAAGGGAACTGG — **8972-9079**
CTTCATGCCATTAAAC~~GTAGGAAAGAAATTCTTCTTGAAGGCTGTGCTGAGAAAAGTAAA~~ — **Δ9080-9144 (65 bp)**
~~CAGCTGAAGGATGAAGGAGCC~~AGTTTGGCTGAAAATAAAAG*gtatgtatttaaagttttt*
tttttatggagaagagcttacattttctagctcactgtccgtaatggg — **9145-9231**

resulting protein amino acid sequence:
MASFGWKRKIGEKVSKVTSQQFEAEAADEKDVVDNDEGNWLHAIKQFG* (**48 aa polypeptide; 5.43 kDa**) - containing almost entire TPR1 (aa 17-50). but missing TPRs 2-4

- exemplary source sequencing chromatogram (sequencing in forward orientation)

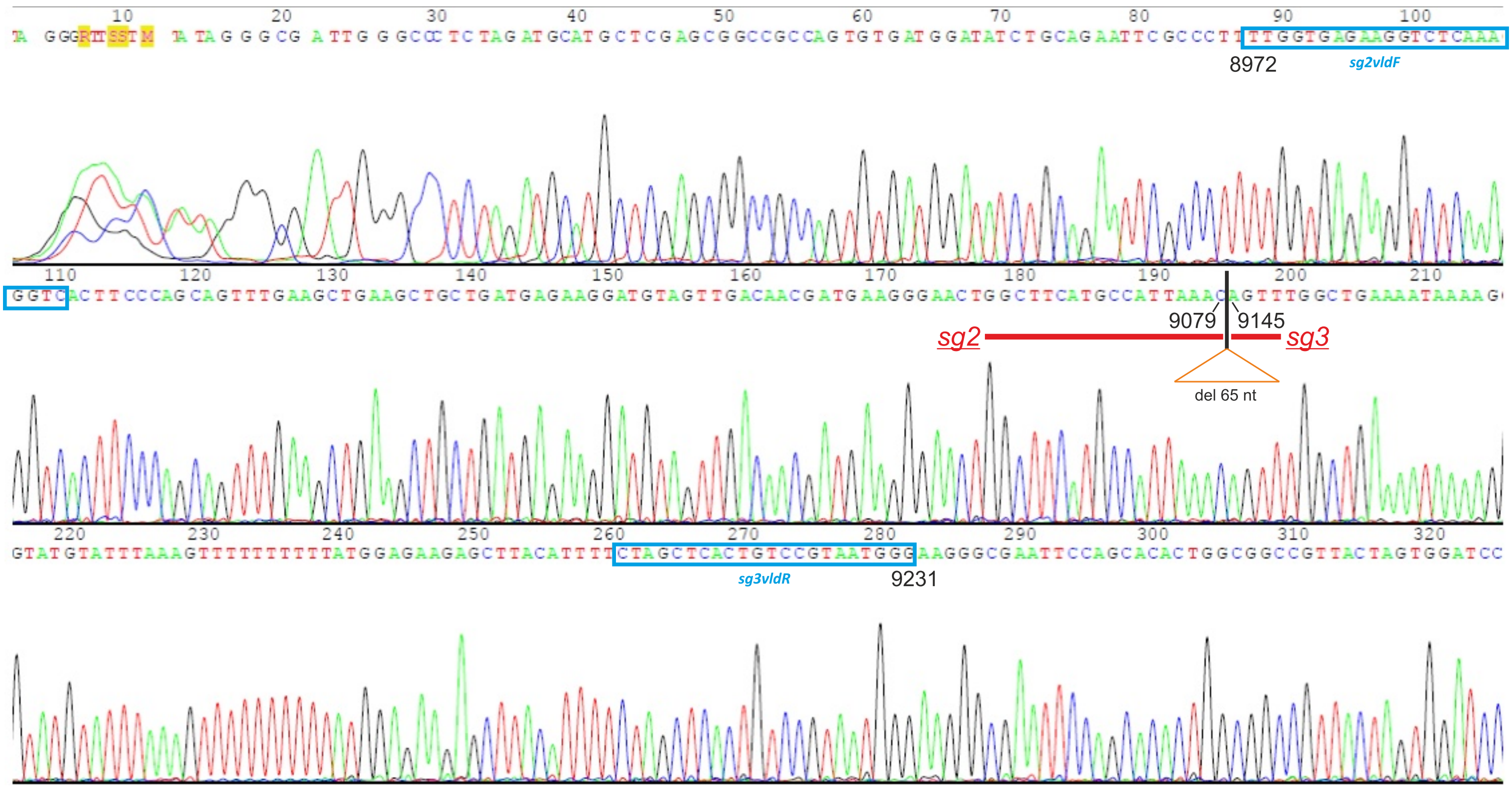

Verification of CCDC97 Crispr-Cas9 knock-outs at the genomic DNA level

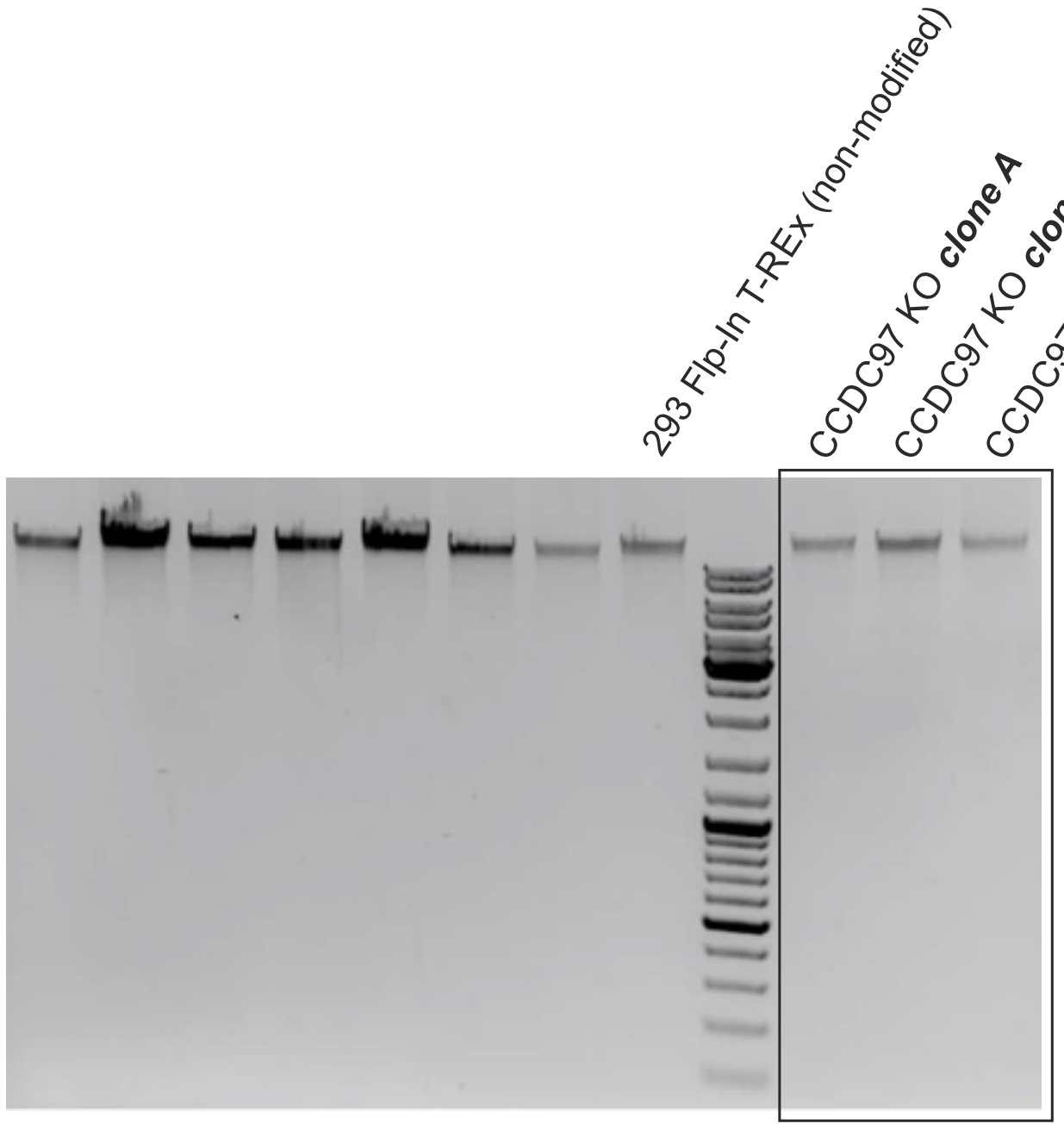

total genomic DNA

Schematic genomic organization of *CCDC97* locus and other relevant information

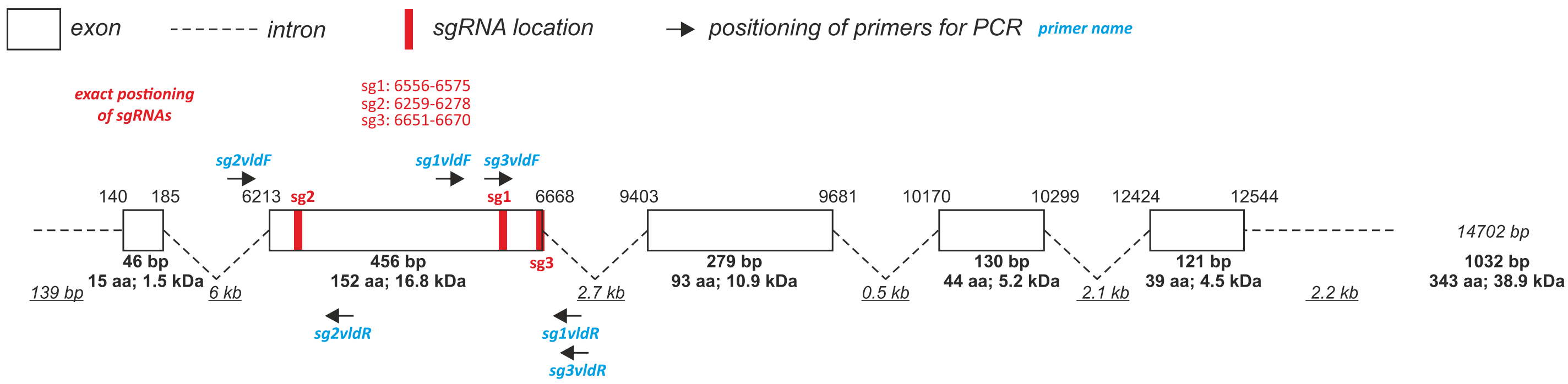

exemplary results of PCR-based validation

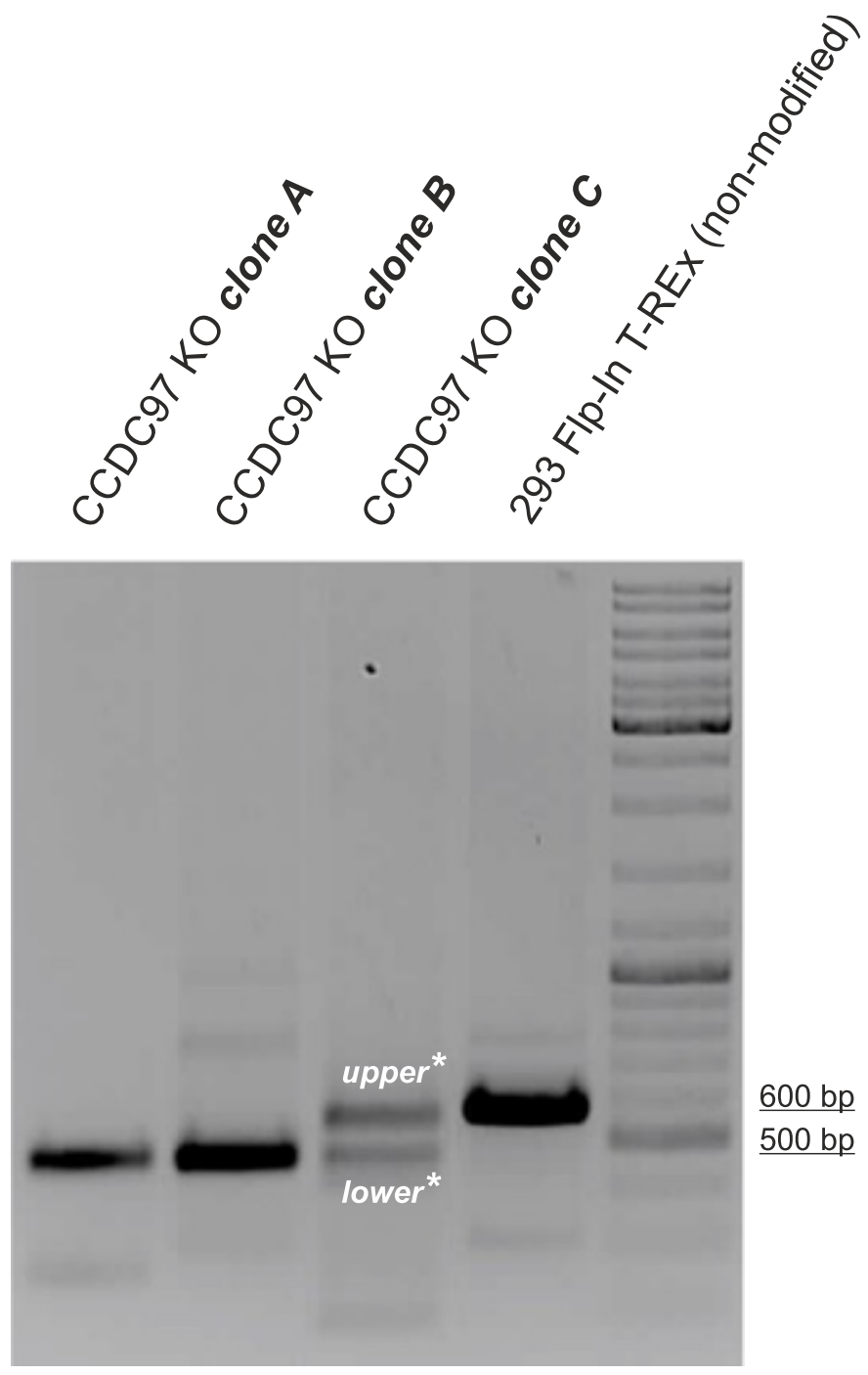

upper/lower* - two distinct PCR products were obtained using total genomic DNA from CCDC97 KO clone C, most likely corresponding to two variably modified alleles of the gene locus; both products were cloned and subjected to Sanger sequencing independently of each other*

primer pair sg2vldF-sg3vldR

predicted product size for control: 567 bp

PCR products obtained using sg2vldF-sg3vldR primer pair for CCDC97 KO clones A, B, C, and negative control (non-modified 293 Flp-In T-REx) were gel-purified, cloned into pCR™-Blunt II-TOPO® vector, and subjected to Sanger sequencing using M13 (-20) universal primer

Sanger sequencing of the wild-type *CCDC97* locus fragment

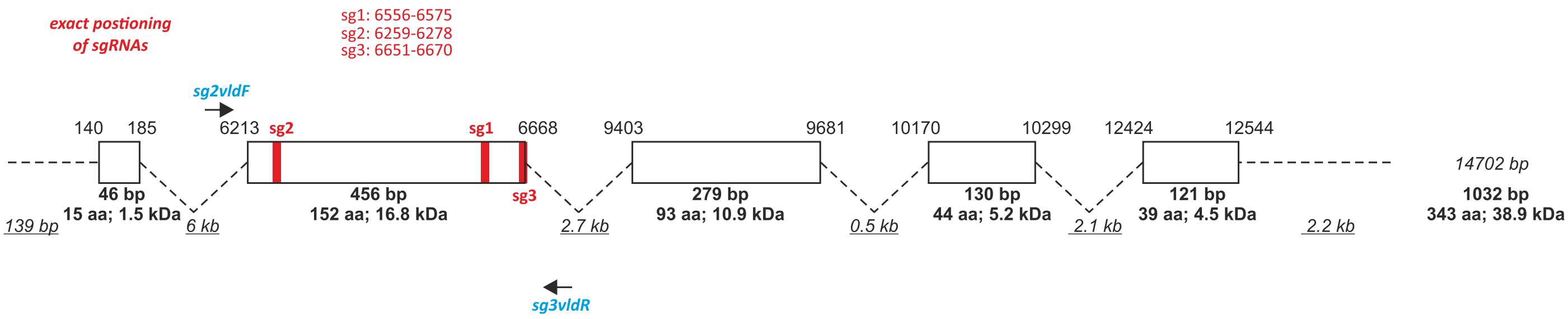

intron 1-2+exon 2+*intron 2-3* sequence (***reference; wild-type***); **6175-6741 (*567 bp*)**

```
ctcccatctctgaactaaccaatctctcctttcctcagGCTGCATAGAGCCTGGACCTGG      sg2vldF
GCACTGGGGTGAGCTGAGCCGGACACCAGTCCCATCTAAACCCCAGGACAAAGTGGAAGC      sg2
AGCTGAGGCAACACCAGTGGCCCTGGACAGTGACACCTCCGGGGCTGAAAATGCAGCAGT
GAGTGCTATGCTGCACGCTGTAGCCGCCAGCCGCCTGCCTGTTTGCAGCCAGCAGCAGGG
TGAACCCGACTTGACAGAGCATGAGAAAGTGGCCATCCTGGCCCAGCTGTACCACGAGAA
GCCACTGGTGTTCCTGGAGCGCTTCCGCACAGGCCTCCGTGAGGAGCATCTGGCCTGCTT
TGGCCACGTGCGTGGCGACCACCGTGCAGACTTCTACTGTGCTGAGGTGGCCCGGCAGGG      sg1
CACTGCCCGGCCCCGCACCCTGCGTACCCGCCTGCGTAACCGGCGCTATGCTGCCCTGCG
AGAGCTGATCCAAGgtgtgggggccagatgggcgacagtgggcacatatggggagggagg      sg3
ggagactgaggcagagggaattagaga                                       sg3vldR
```

Topo+PCR for non-modified 293 Flp-In T-REx (***sequence read***)

6175-6741

567 bp

```
ctcccatctctgaactaaccaatctctcctttcctcagGCTGCATAGAGCCTGGACCTGG
GCACTGGGGTGAGCTGAGCCGGACACCAGTCCCATCTAAACCCCAGGACAAAGTGGAAGC
AGCTGAGGCAACACCAGTGGCCCTGGACAGTGACACCTCCGGGGCTGAAAATGCAGCAGT
GAGTGCTATGCTGCACGCTGTAGCCGCCAGCCGCCTGCCTGTTTGCAGCCAGCAGCAGGG
TGAACCCGACTTGACAGAGCATGAGAAAGTGGCCATCCTGGCCCAGCTGTACCACGAGAA
GCCACTGGTGTTCCTGGAGCGCTTCCGCACAGGCCTCCGTGAGGAGCATCTGGCCTGCTT
TGGCCACGTGCGTGGCGACCACCGTGCAGACTTCTACTGTGCTGAGGTGGCCCGGCAGGG
CACTGCCCGGCCCCGCACCCTGCGTACCCGCCTGCGTAACCGGCGCTATGCTGCCCTGCG
AGAGCTGATCCAAGgtgtgggggccagatgggcgacagtgggcacatatggggagggagg
ggagactgaggcagagggaattagaga
```

(sequence identical to the reference one)

resulting protein amino acid sequence:

```
MEAVATATAAKEPDKGCIEPGPGHWGELSRTPVPSKPQDKVEAAEATPVALDSDTSGAEN
AAVSAMLHAVAASRLPVCSQQQGEPDLTEHEKVAILAQLYHEKPLVFLERFRTGLREEHL
ACFGHVRGDHRADFYCAEVARQGTARPRTLRTRLRNRRYAALRELIQ...
```

(**343 aa protein; 38.9 kDa**)

- *source sequencing chromatogram:* ***see next page***

Sanger sequencing of the wild-type *CCDC97* locus fragment

- source sequencing chromatogram (sequencing in forward orientation)

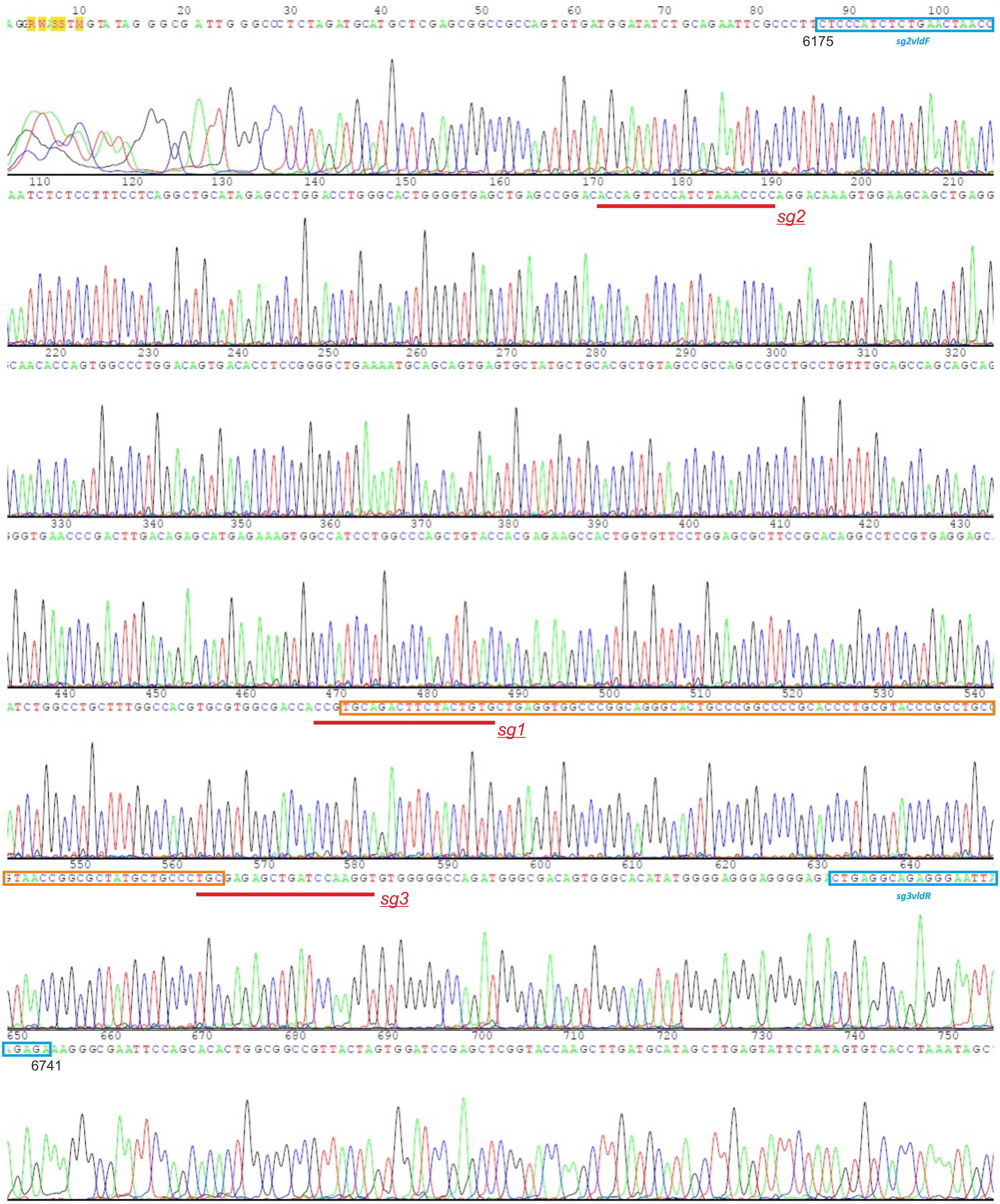

Sanger sequencing of the *CCDC97* locus fragment in CCDC97 KO clone A

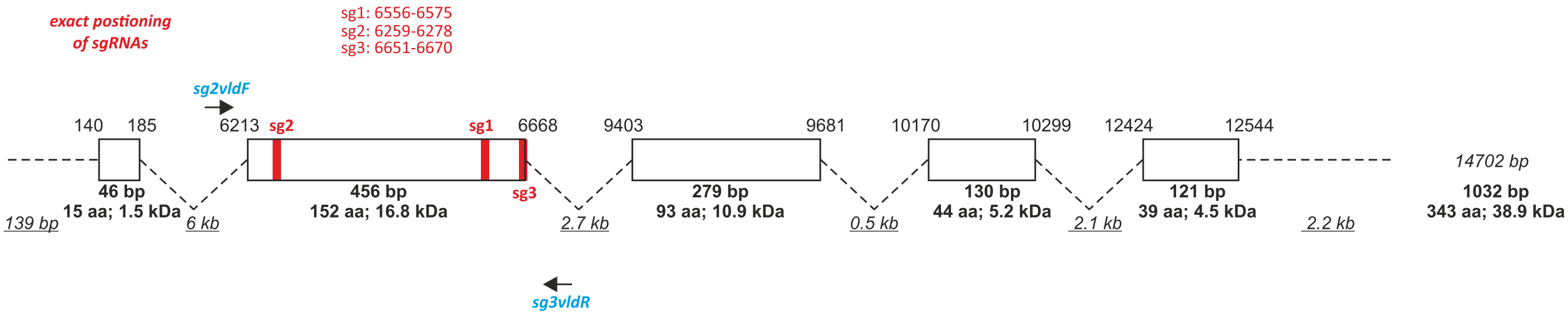

intron 1-2+exon 2+*intron 2-3* sequence (***reference; wild-type***); **6175-6741 (*567 bp*)**

*ctcccatctctgaactaacc**aatctctcctttcctcag*GCTGCATAGAGCCTGGACCTGG — sg2vldF
GCACTGGGGTGAGCTGAGCCGGACACCAGTCCCATCTAAACCCCAGGACAAAGTGGAAGC — **sg2**
AGCTGAGGCAACACCAGTGGCCCTGGACAGTGACACCTCCGGGGCTGAAAATGCAGCAGT
GAGTGCTATGCTGCACGCTGTAGCCGCCAGCCGCCTGCCTGTTTGCAGCCAGCAGCAGGG
TGAACCCGACTTGACAGAGCATGAGAAAGTGGCCATCCTGGCCCAGCTGTACCACGAGAA
GCCACTGGTGTTCCTGGAGCGCTTCCGCACAGGCCTCCGTGAGGAGCATCTGGCCTGCTT
TGGCCACGTGCGTGGCGACCACCGTGCAGACTTCTACTGTGCTGAGGTGGCCCGGCAGGG — **sg1**
CACTGCCCGGCCCCGCACCCTGCGTACCCGCCTGCGTAACCGGCGCTATGCTGCCCTGCG
AGAGCTGATCCAAG*gtgtgggggccagatgggcgacagtgggcacatatggggagggagg* — **sg3**
ggagactgaggcagagggaattagaga — sg3vldR

Topo+PCR for CCDC97 KO A (***sequence read***)

472 bp

*ctcccatctctgaactaaccaatctctcctttcctcag*GCTGCATAGAGCCTGGACCTGG — **6175-6558**
GCACTGGGGTGAGCTGAGCCGGACACCAGTCCCATCTAAACCCCAGGACAAAGTGGAAGC
AGCTGAGGCAACACCAGTGGCCCTGGACAGTGACACCTCCGGGGCTGAAAATGCAGCAGT
GAGTGCTATGCTGCACGCTGTAGCCGCCAGCCGCCTGCCTGTTTGCAGCCAGCAGCAGGG
TGAACCCGACTTGACAGAGCATGAGAAAGTGGCCATCCTGGCCCAGCTGTACCACGAGAA
GCCACTGGTGTTCCTGGAGCGCTTCCGCACAGGCCTCCGTGAGGAGCATCTGGCCTGCTT
TGGCCACGTGCGTGGCGACCACCG~~TGCAGACTTCTACTGTGCTGAGGTGGCCCGGCAGGG~~ — **∆6559-6653 (95 bp)**
~~CACTGCCCGGCCCCGCACCCTGCGTACCCGCCTGCGTAACCGGCGCTATGCTGCCCTGC~~G
AGAGCTGATCCAAG*gtgtgggggccagatgggcgacagtgggcacatatggggagggagg* — **6654-6741**
ggagactgaggcagagggaattagaga

resulting protein amino acid sequence:
MEAVATATAAKEPDKGCIEPGPGHWGELSRTPVPSKPQDKVEAAEATPVALDSDTSGAEN
AAVSAMLHAVAASRLPVCSQQQGEPDLTEHEKVAILAQLYHEKPLVFLERFRTGLREEHL
ACFGHVRGDHRRADPRCGGQMGDSGHIWGGRGD*
(**153 aa protein; 16.3 kDa**) - containing almost entire PHF-binding site, but lacking coiled-coil domain

- *source sequencing chromatogram:* ***see next page***

Sanger sequencing of the *CCDC97* locus fragment in CCDC97 KO clone A

- *source sequencing chromatogram (sequencing in reverse orientation)*

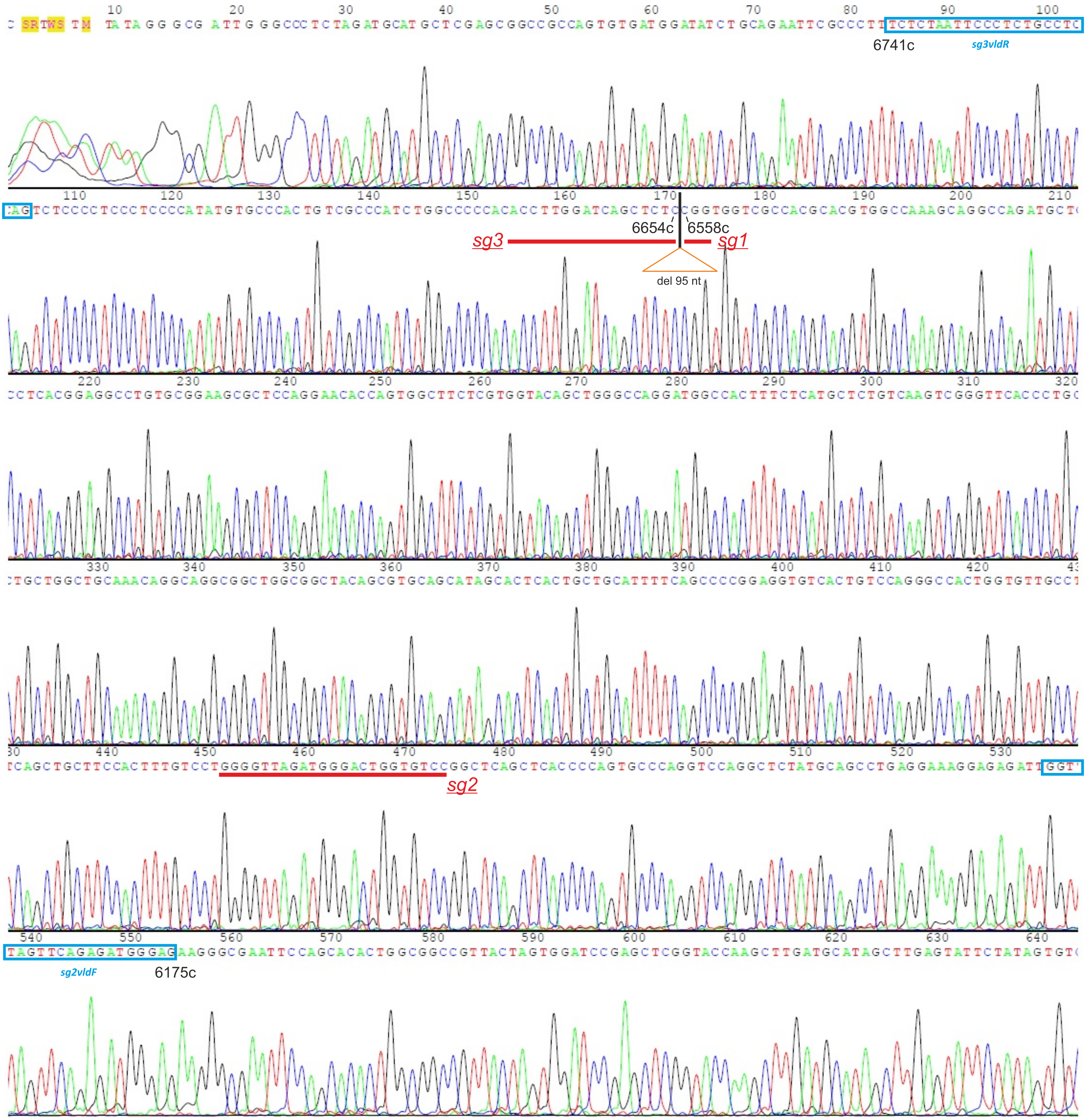

Sanger sequencing of the *CCDC97* locus fragment in CCDC97 KO clone B

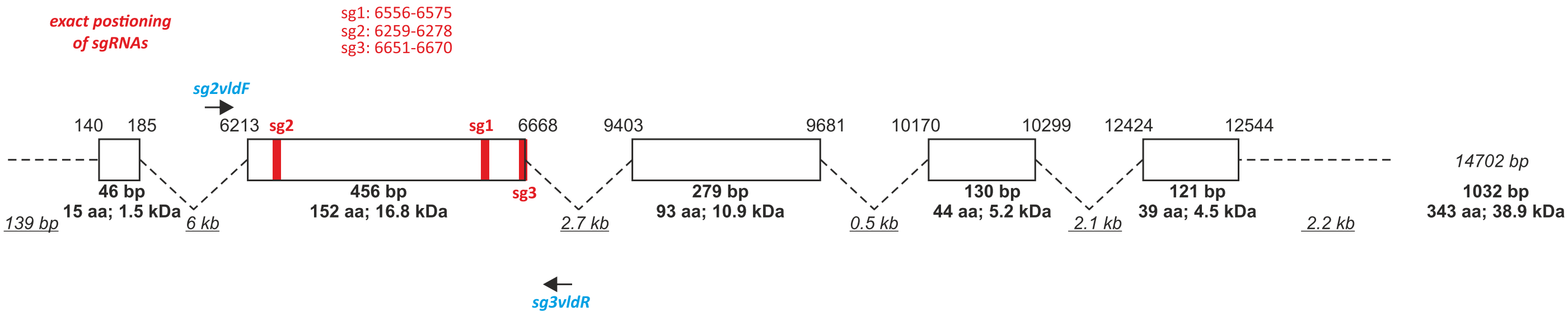

intron 1-2+exon 2+*intron 2-3* sequence (***reference; wild-type***); **6175-6741 (*567 bp*)**

*ctcccatctctgaactaaccaatctctcctttcctcag*GCTGCATAGAGCCTGGACCTGG sg2vldF
GCACTGGGGTGAGCTGAGCCGGACACCAGTCCCATCTAAACCCCAGGACAAAGTGGAAGC **sg2**
AGCTGAGGCAACACCAGTGGCCCTGGACAGTGACACCTCCGGGGCTGAAAATGCAGCAGT
GAGTGCTATGCTGCACGCTGTAGCCGCCAGCCGCCTGCCTGTTTGCAGCCAGCAGCAGGG
TGAACCCGACTTGACAGAGCATGAGAAAGTGGCCATCCTGGCCCAGCTGTACCACGAGAA
GCCACTGGTGTTCCTGGAGCGCTTCCGCACAGGCCTCCGTGAGGAGCATCTGGCCTGCTT
TGGCCACGTGCGTGGCGACCACCGTGCAGACTTCTACTGTGCTGAGGTGGCCCGGCAGGG **sg1**
CACTGCCCGGCCCCGCACCCTGCGTACCCGCCTGCGTAACCGGCGCTATGCTGCCCTGCG
AGAGCTGATCCAAG*gtgtgggggccagatgggcgacagtgggcacatatggggagggagg* **sg3**
ggagactgaggcagagggaattagaga sg3vldR

Topo+PCR for CCDC97 KO B (***sequence read***)

*ctcccatctctgaactaaccaatctctcctttcctcag*GCTGCATAGAGCCTGGACCTGG **6175-6275**
GCACTGGGGTGAGCTGAGCCGGACACCAGTCCCATCTAAACCGGTGGTCGCCACGCACGT
GGCCAAAGCAGGCCAGATGCTCCTCACGGAGGCCTGTGCGGAAGCGCTCCAGGAACACCA
GTGGCTTCTCGTGGTACAGCTGGGCCAGGATGGCCACTTTCTCATGCTCTGTCAAGTCGG **6558-6276 opposite strand**
472 bp GTTCACCCTGCTGCTGGCTGCAAACAGGCAGGCGGCTGGCGGCTACAGCGTGCAGCATAG
CACTCACTGCTGCATTTTCAGCCCCGGAGGTGTCACTGTCCAGGGCCACTGGTGTTGCCT
CAGCTGCTTCCACTTTGTCCTGGG~~TGCAGACTTCTACTGTGCTGAGGTGGCCCGGCAGGG~~ **∆6559-6653 (95 bp)**
~~CACTGCCCGGCCCCGCACCCTGCGTACCCGCCTGCGTAACCGGCGCTATGCTGCCCTGC~~G
AGAGCTGATCCAAG*gtgtgggggccagatgggcgacagtgggcacatatggggagggagg* **6654-6741**
ggagactgaggcagagggaattagaga

resulting protein amino acid sequence:
MEAVATATAAKEPDKGCIEPGPGHWGELSRTPVPSKPVVATHVAKAGQMLLTEACAEALQ
EHQWLLVVQLGQDGHFLMLCQVGFTLLLAANRQAAGGYSVQHSTHCCIFSPGGVTVQGHW
CCLSCFHFVLGRADPRCGGQMGDSGHIWGGRGD*
(**153 aa protein; 16.2 kDa**) - lacking most of the potential structural elements

- source sequencing chromatogram: ***see next page***

Sanger sequencing of the *CCDC97* locus fragment in CCDC97 KO clone B

- source sequencing chromatogram (sequencing in reverse orientation)

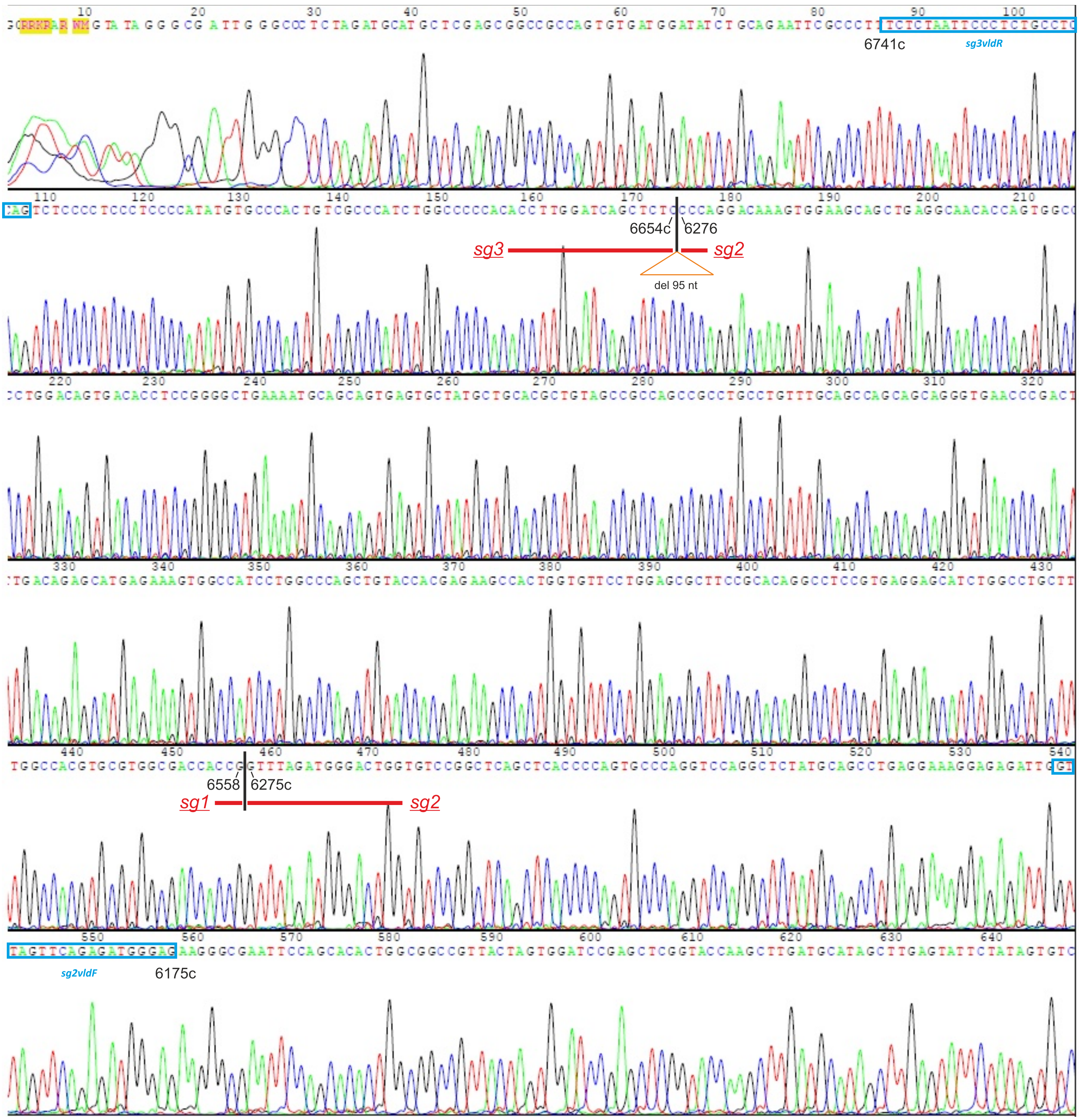

Sanger sequencing of the *CCDC97* locus fragment in CCDC97 KO clone C

upper band (see PCR result on page 5)

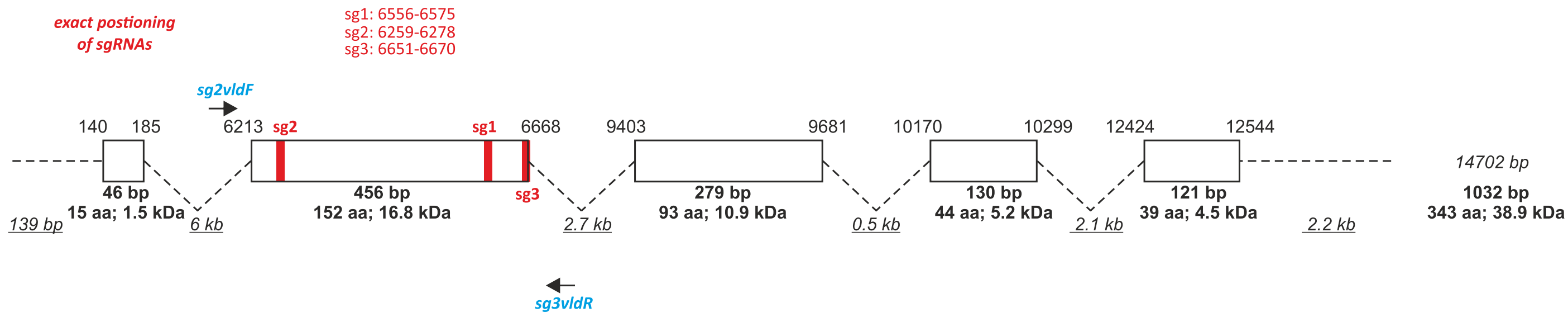

intron 1-2+exon 2+*intron 2-3* sequence (***reference; wild-type***); **6175-6741 (*567 bp*)**

ctcccatctctgaactaaccaatctctcctttcctcagGCTGCATAGAGCCTGGACCTGG — sg2vldF
GCACTGGGGTGAGCTGAGCCGGACACCAGTCCCATCTAAACCCCAGGACAAAGTGGAAGC — sg2
AGCTGAGGCAACACCAGTGGCCCTGGACAGTGACACCTCCGGGGCTGAAAATGCAGCAGT
GAGTGCTATGCTGCACGCTGTAGCCGCCAGCCGCCTGCCTGTTTGCAGCCAGCAGCAGGG
TGAACCCGACTTGACAGAGCATGAGAAAGTGGCCATCCTGGCCCAGCTGTACCACGAGAA
GCCACTGGTGTTCCTGGAGCGCTTCCGCACAGGCCTCCGTGAGGAGCATCTGGCCTGCTT
TGGCCACGTGCGTGGCGACCACCGTGCAGACTTCTACTGTGCTGAGGTGGCCCGGCAGGG — sg1
CACTGCCCGGCCCCGCACCCTGCGTACCCGCCTGCGTAACCGGCGCTATGCTGCCCTGCG
AGAGCTGATCCAAGgtgtgggggccagatgggcgacagtgggcacatatggggagggagg — sg3
ggagactgaggcagagggaattagaga — sg3vldR

Topo+PCR for CCDC97 KO C, *upper band* (***sequence read***)

567 bp

ctcccatctctgaactaaccaatctctcctttcctcagGCTGCATAGAGCCTGGACCTGG — 6175-6275
GCACTGGGGTGAGCTGAGCCGGACACCAGTCCCATCTAAACCGGTGGTCGCCACGCACGT
GGCCAAAGCAGGCCAGATGCTCCTCACGGAGGCCTGTGCGGAAGCGCTCCAGGAACACCA
GTGGCTTCTCGTGGTACAGCTGGGCCAGGATGGCCACTTTCTCATGCTCTGTCAAGTCGG — 6558-6276 opposite strand
GTTCACCCTGCTGCTGGCTGCAAACAGGCAGGCGGCTGGCGGCTACAGCGTGCAGCATAG
CACTCACTGCTGCATTTTCAGCCCCGGAGGTGTCACTGTCCAGGGCCACTGGTGTTGCCT
CAGCTGCTTCCACTTTGTCCTGGGTCGCAGGGCAGCATAGCGCCGGTTACGCAGGCGGGT — 6655-6559 opposite strand
ACGCAGGGTGCGGGGCCGGGCAGTGCCCTGCCGGGCCACCTCAGCACAGTAGAAGTCTGC — *6654-6655 duplicated*
AGAGAGCTGATCCAAGgtgtgggggccagatgggcgacagtgggcacatatggggaggga
ggggagactgaggcagagggaattagaga — 6654-6739

resulting protein amino acid sequence:

MEAVATATAAKEPDKGCIEPGPGHWGELSRTPVPSKPVVATHVAKAGQMLLTEACAEALQ
EHQWLLVVQLGQDGHFLMLCQVGFTLLLAANRQAAGGYSVQHSTHCCIFSPGGVTVQGHW
CCLSCFHFVLGRRAA*

(**135 aa protein; 14.3 kDa**) - lacking most of the potential structural elements

- *source sequencing chromatogram:* ***see next page***

Sanger sequencing of the *CCDC97* locus fragment in CCDC97 KO clone C

upper band (see PCR result on page 5)

- *source sequencing chromatogram (sequencing in reverse orientation)*

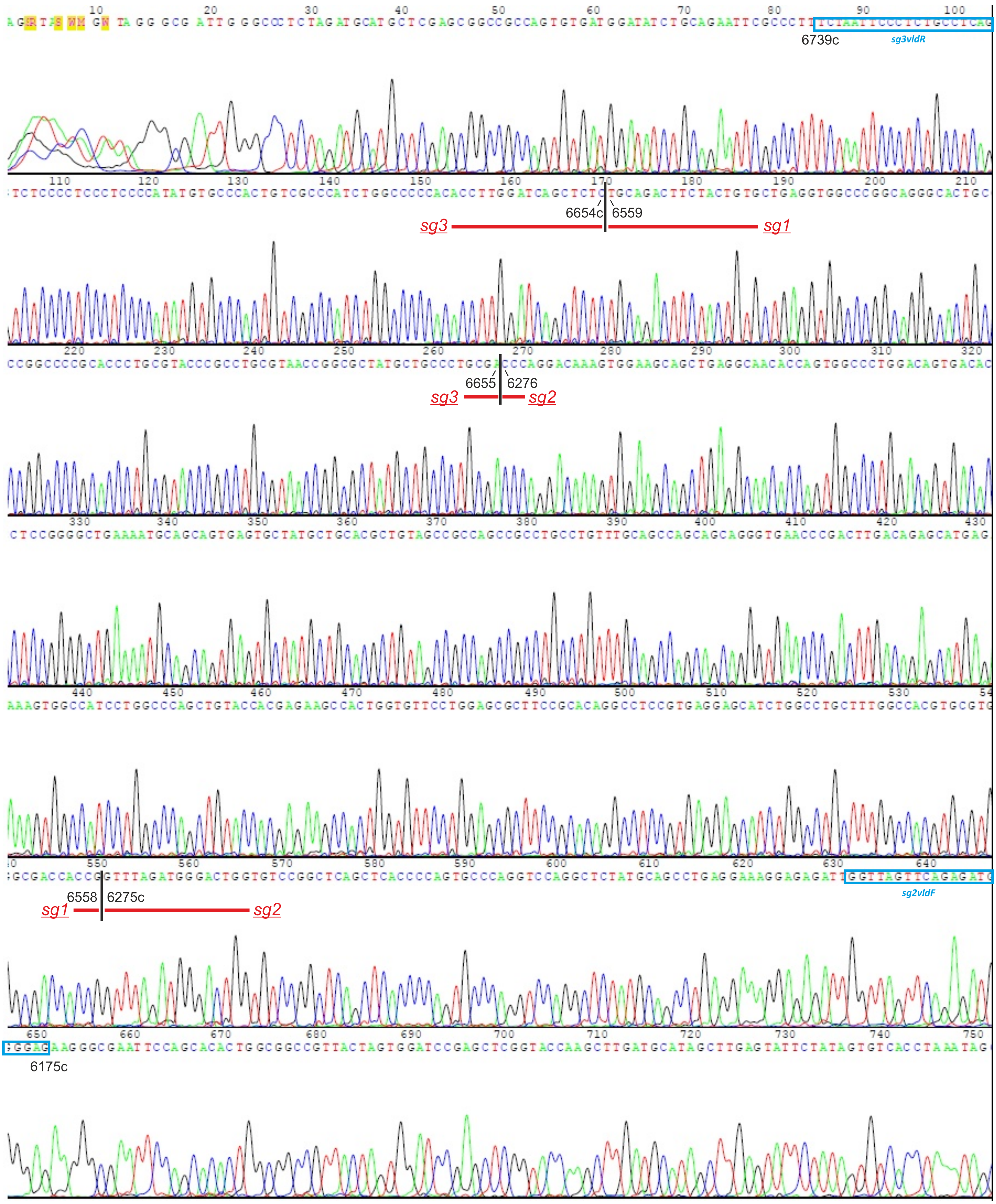

Sanger sequencing of the *CCDC97* locus fragment in CCDC97 KO clone C

lower band (see PCR result on page 5)

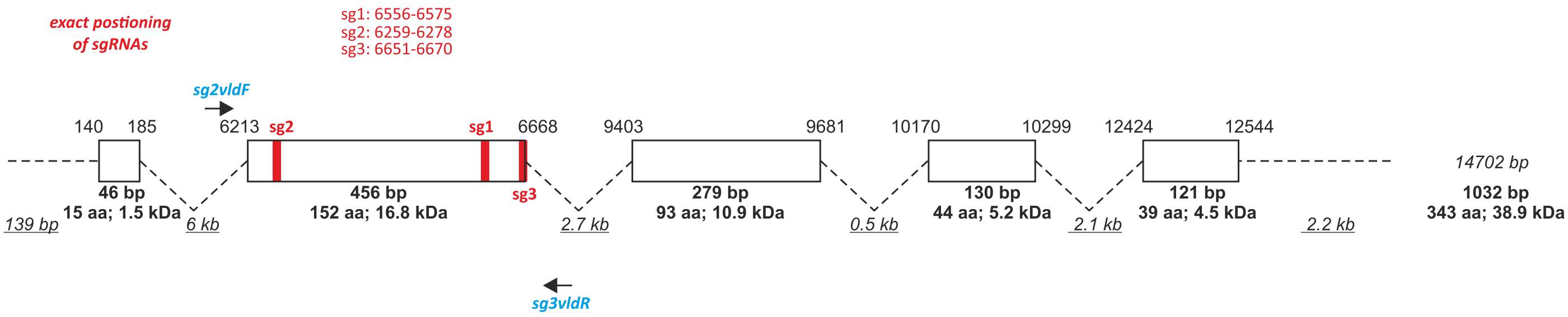

intron 1-2+exon 2+*intron 2-3* sequence (***reference; wild-type***); **6175-6741 (*567 bp*)**

*ctcccatctctgaactaaccaatctctcctttcctcag*GCTGCATAGAGCCTGGACCTGG — sg2vldF
GCACTGGGGTGAGCTGAGCCGGACACCAGTCCCATCTAAACCCCAGGACAAAGTGGAAGC — **sg2**
AGCTGAGGCAACACCAGTGGCCCTGGACAGTGACACCTCCGGGGCTGAAAATGCAGCAGT
GAGTGCTATGCTGCACGCTGTAGCCGCCAGCCGCCTGCCTGTTTGCAGCCAGCAGCAGGG
TGAACCCGACTTGACAGAGCATGAGAAAGTGGCCATCCTGGCCCAGCTGTACCACGAGAA
GCCACTGGTGTTCCTGGAGCGCTTCCGCACAGGCCTCCGTGAGGAGCATCTGGCCTGCTT
TGGCCACGTGCGTGGCGACCACCGTGCAGACTTCTACTGTGCTGAGGTGGCCCGGCAGGG — **sg1**
CACTGCCCGGCCCCGCACCCTGCGTACCCGCCTGCGTAACCGGCGCTATGCTGCCCTGCG
AGAGCTGATCCAAG*gtgtgggggccagatgggcgacagtgggcacatatggggagggagg* — **sg3**
ggagactgaggcagagggaattagaga — sg3vldR

Topo+PCR for CCDC97 KO C, *lower band* (***sequence read***)

473 bp

*ctcccatctctgaactaaccaatctctcctttcctcag*GCTGCATAGAGCCTGGACCTGG — **6175-6275**
GCACTGGGGTGAGCTGAGCCGGACACCAGTCCCATCTAAA*C*CGGTGGTCGCCACGCACGT
GGCCAAAGCAGGCCAGATGCTCCTCACGGAGGCCTGTGCGGAAGCGCTCCAGGAACACCA — **6558-6275 opposite strand; *6275 duplicated***
GTGGCTTCTCGTGGTACAGCTGGGCCAGGATGGCCACTTTCTCATGCTCTGTCAAGTCGG
GTTCACCCTGCTGCTGGCTGCAAACAGGCAGGCGGCTGGCGGCTACAGCGTGCAGCATAG
CACTCACTGCTGCATTTTCAGCCCCGGAGGTGTCACTGTCCAGGGCCACTGGTGTTGCCT
CAGCTGCTTCCACTTTGTCCTGGG*G*~~TGCAGACTTCTACTGTGCTGAGGTGGCCCGGCAGG~~
~~GCACTGCCCGGCCCCGCACCCTGCGTACCCGCCTGCGTAACCGGCGCTATGCTGCCCTGC~~ — **∆6559-6653 (95 bp)**
GAGAGCTGATCCAAG*gtgtgggggccagatgggcgacagtgggcacatatggggagggag* — **6654-6741**
gggagactgaggcagagggaattagaga

resulting protein amino acid sequence:

MEAVATATAAKEPDKGCIEPGPGHWGELSRTPVPSKPVVATHVAKAGQMLLTEACAEALQ
EHQWLLVVQLGQDGHFLMLCQVGFTLLLAANRQAAGGYSVQHSTHCCIFSPGGVTVQGHW
CCLSCFHFVLGES*

(**133 aa protein; 14.1 kDa**) - lacking most of the potential structural elements

- *source sequencing chromatogram:* ***see next page***

Sanger sequencing of the *CCDC97* locus fragment in CCDC97 KO clone C

lower band (see PCR result on page 5)

- source sequencing chromatogram (sequencing in forward orientation)

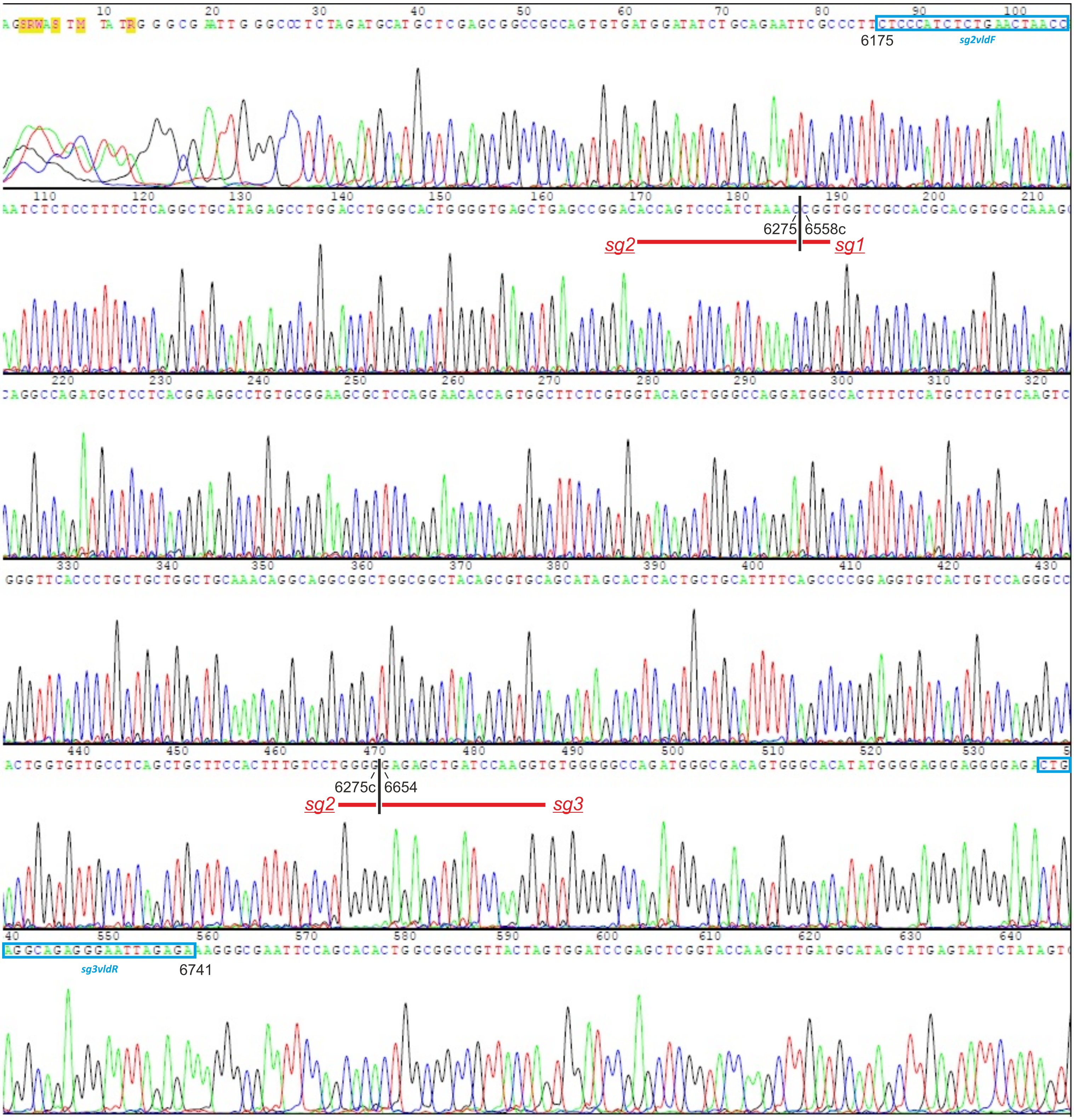

Source Data for Supplementary Blots

Supplementary Figure 1 b. Validation of the bait presence in sample from HEK293 Flp-In T-REx stable cell line producing TTC33-EGFP subjected to co-IP-LF-MS analysis along with negative control

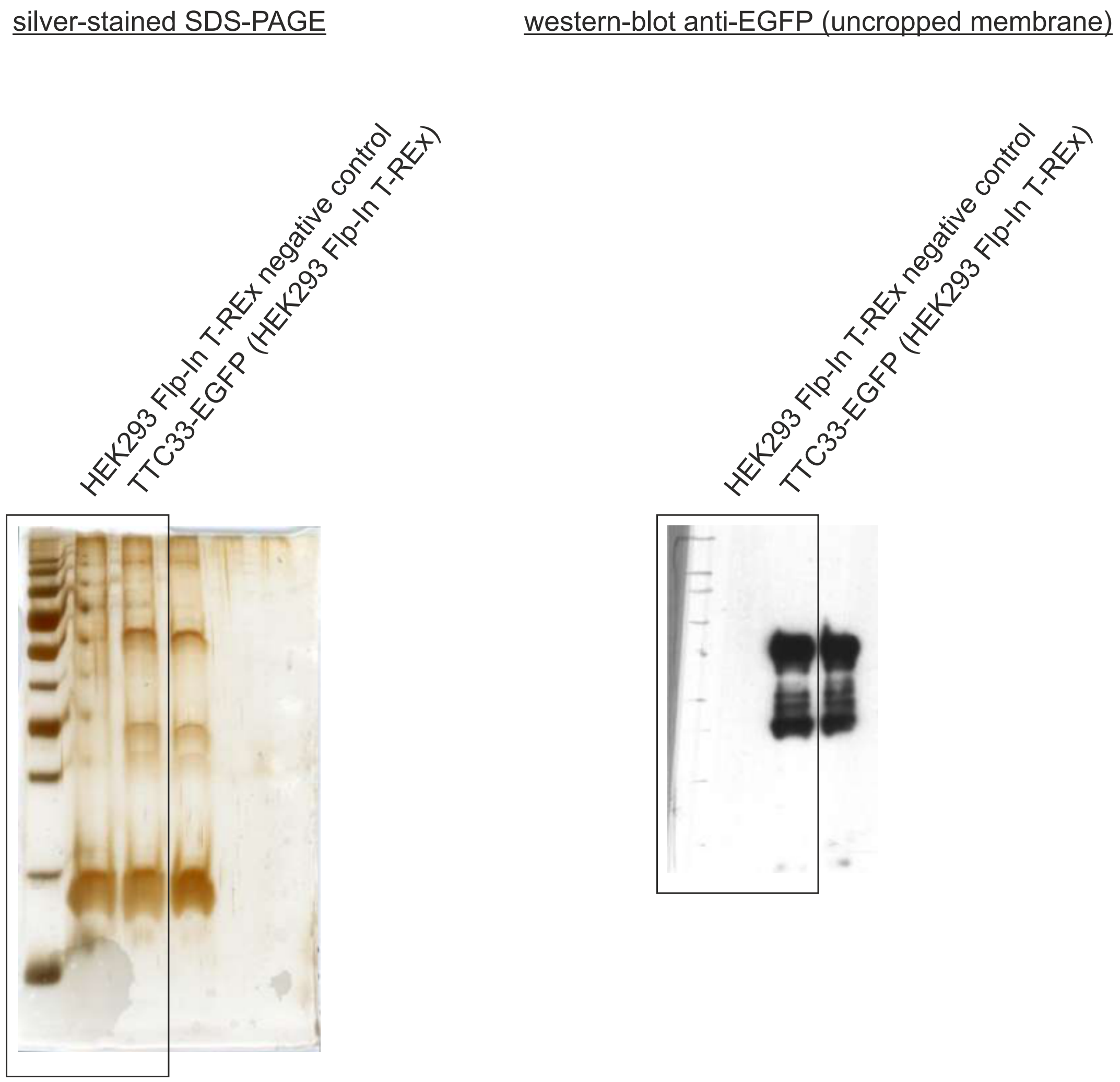

Supplementary Figure 1 c. Validation of the bait presence in sample from transiently transfected FreeStyle 293-F producing TTC33-EGFP subjected to co-IP-LF-MS analysis along with negative control

silver-stained SDS-PAGE

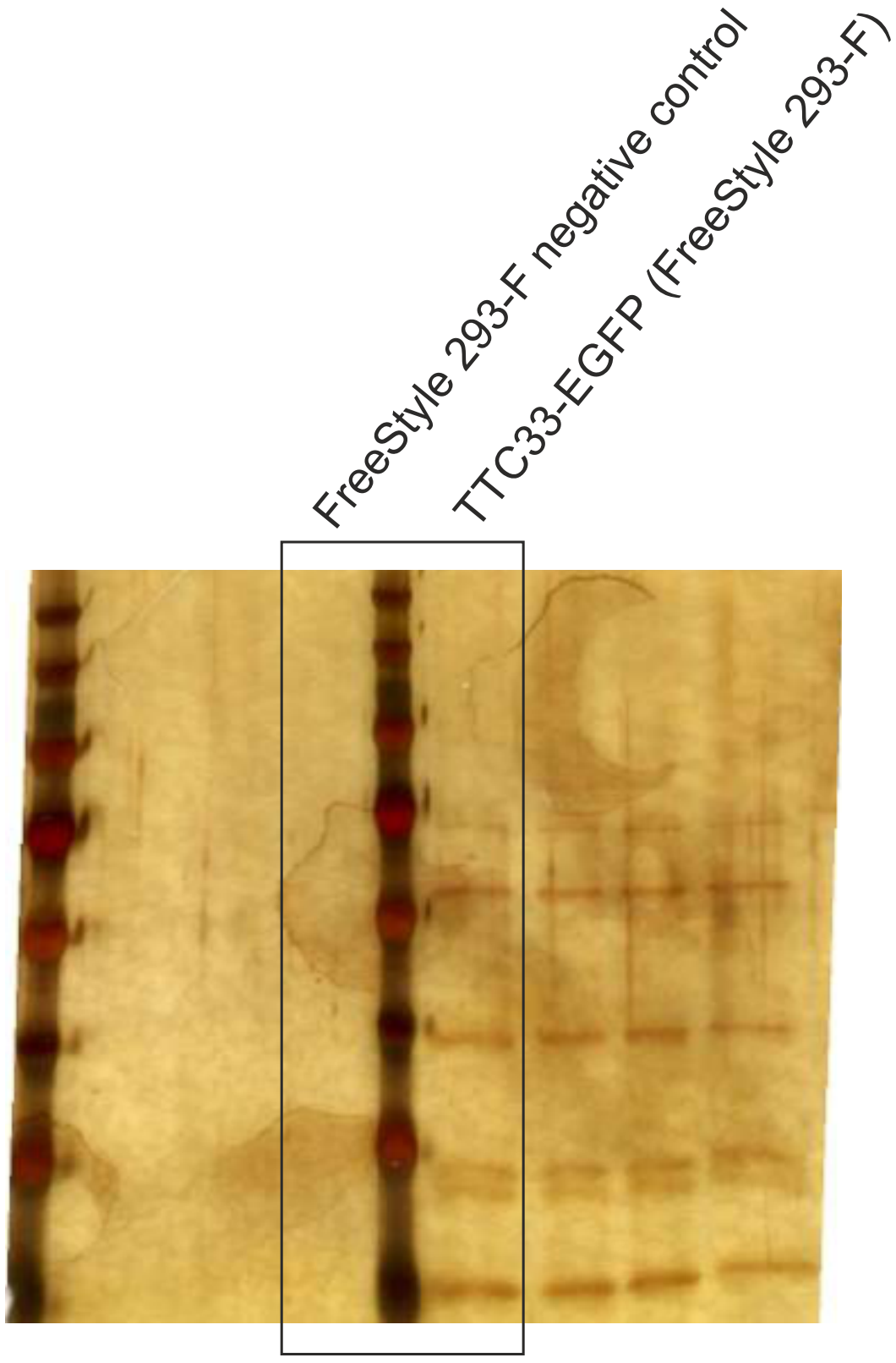

Supplementary Figure 1 d. Western-blot validation of the bait presence in samples from HeLa Flp-In T-REx stable cell line producing TTC33-EGFP subjected to co-IP-LF-MS analysis along with negative controls

membrane after transfer stained with Ponceau S red

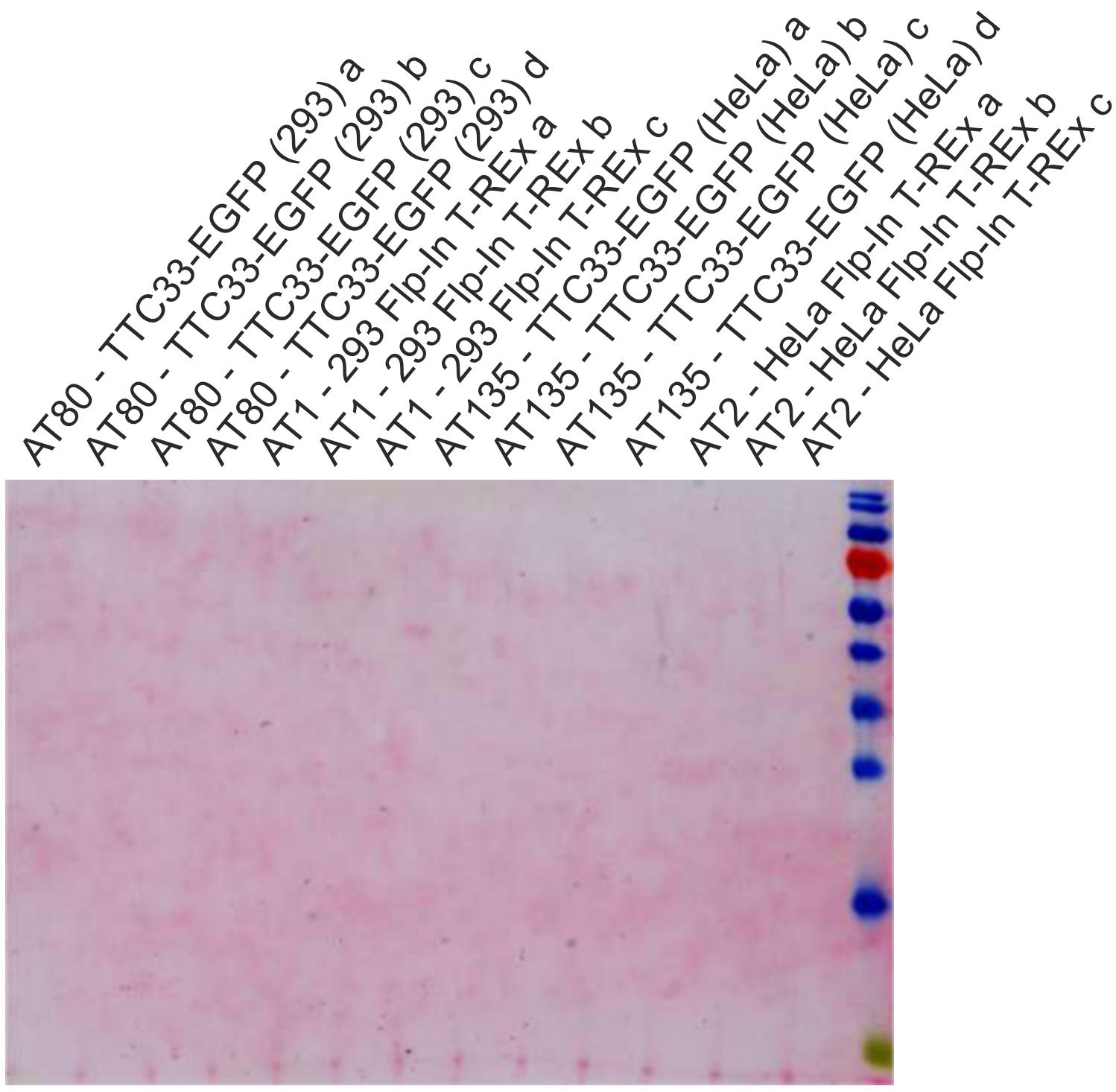

western-blot results (uncropped membrane)

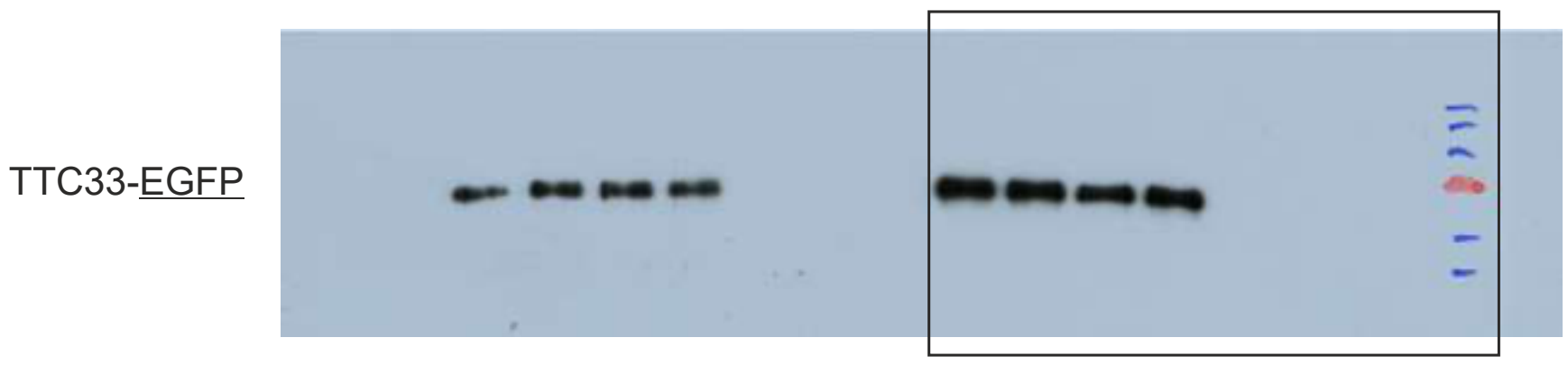

Supplementary Figure 1 e. Western-blot validation of the bait presence in samples from 293 Flp-In T-REx stable cell line producing MBP-TTC33 subjected to co-IP-LF-MS analysis along with negative controls

membrane after transfer stained with Ponceau S red

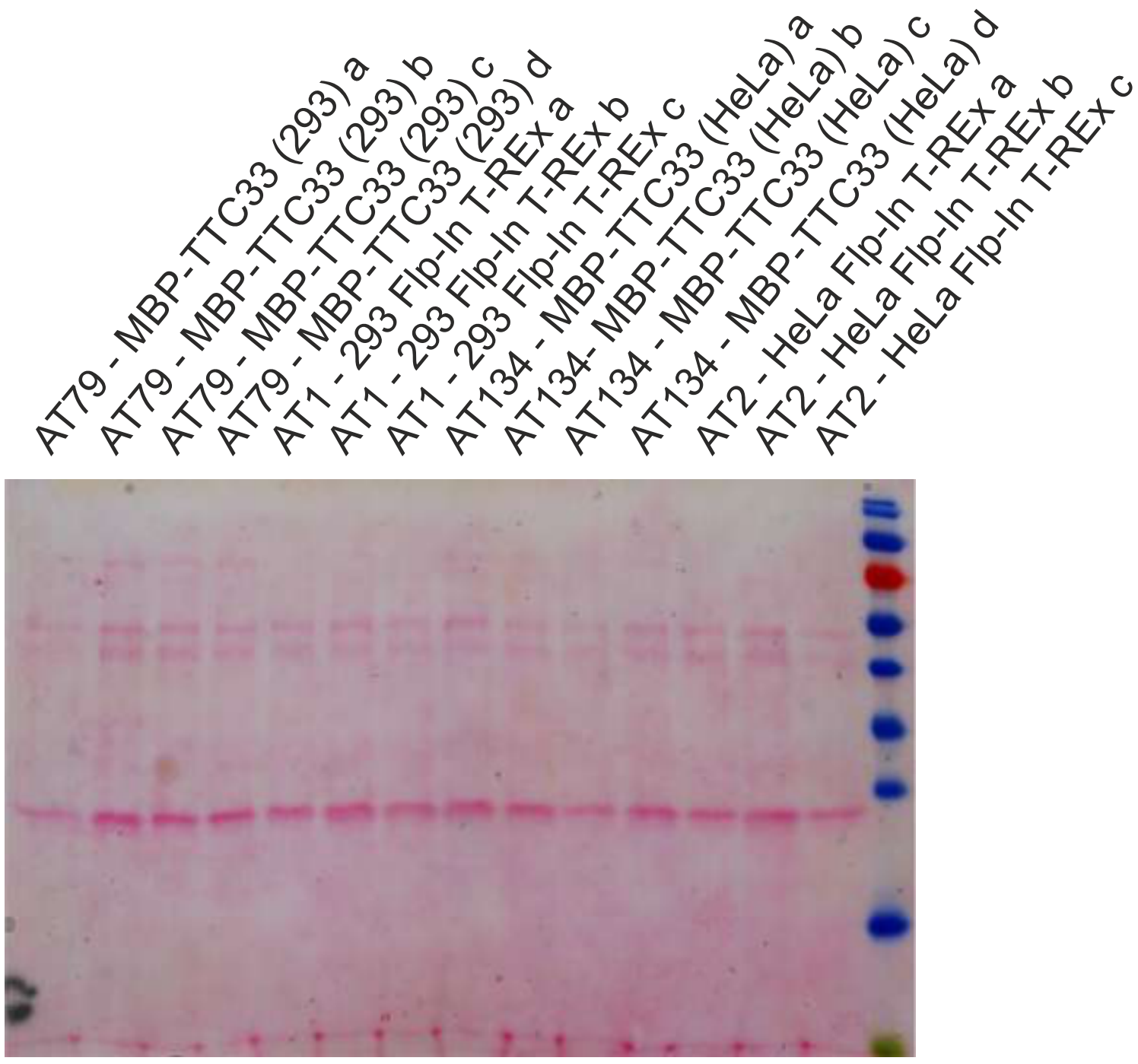

western-blot results (uncropped membrane)

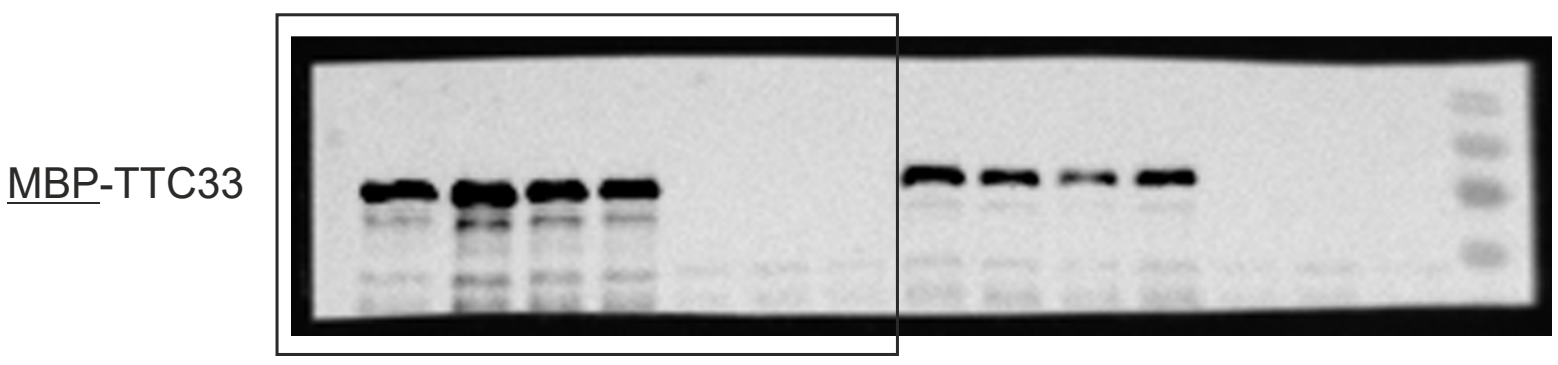

Supplementary Figure 2 c. coIP-WB from FreeStyle 293-F using PPP2R1A-EGFP or CCT-EGFP as baits

western-blot results (uncropped membranes)

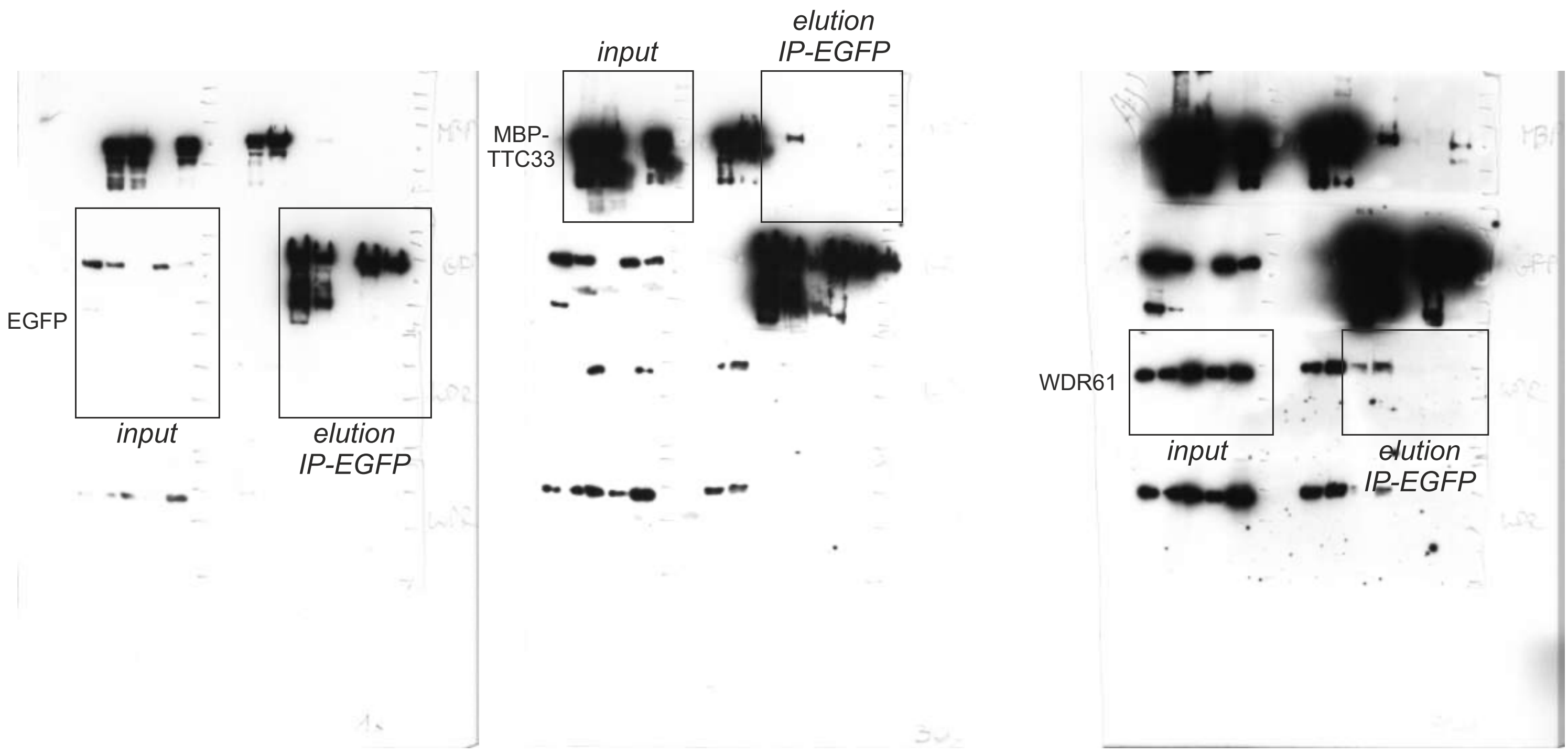

Supplementary Figure 2 d. coIP-WB from FreeStyle 293-F using EGFP-UNG2 as bait

western-blot results (uncropped membranes)

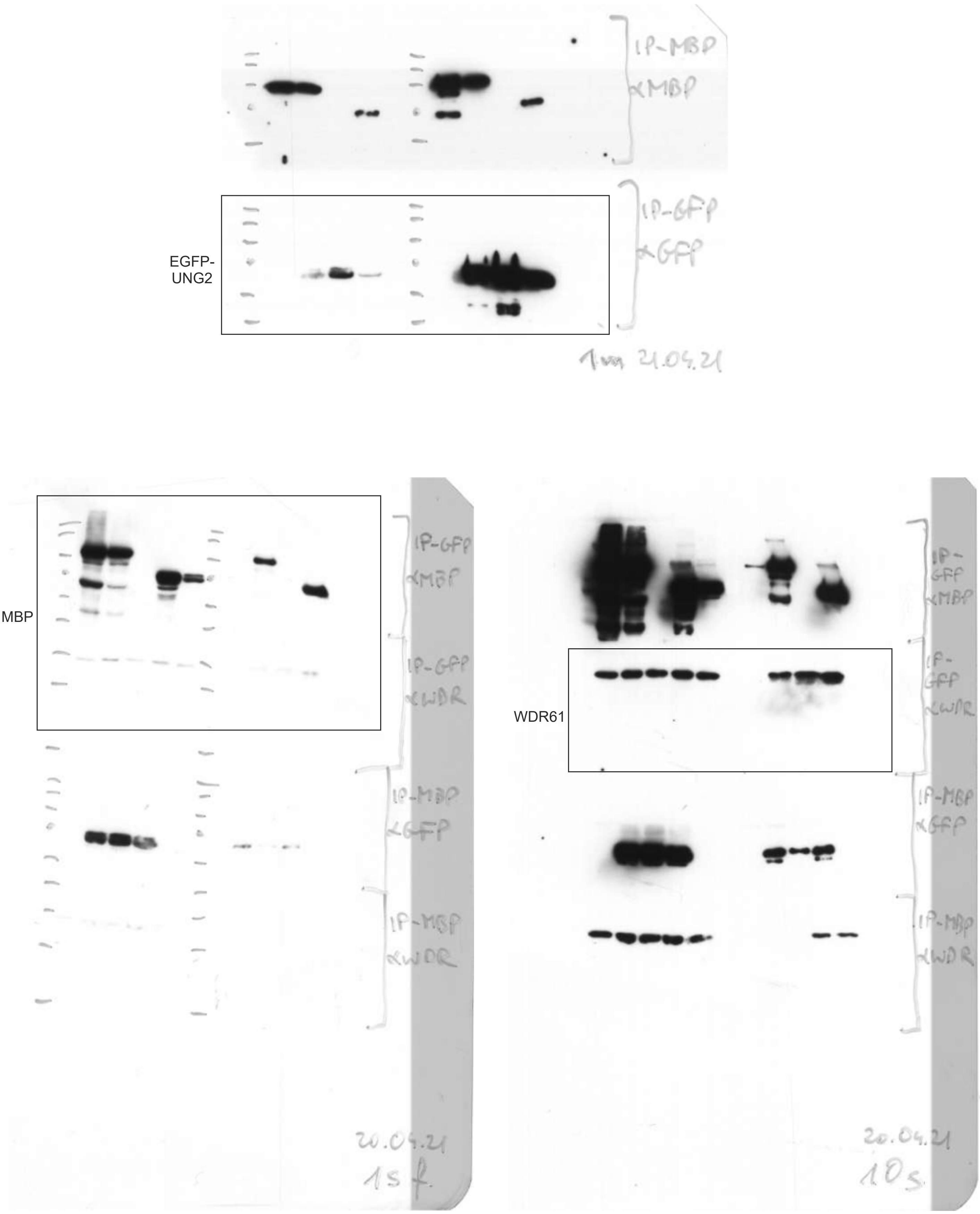

Supplementary Figure 2 e. coIP silver and WB of LF-MS EGFP-WDR61 WT vs W20C samples

silver-stained SDS-PAGE

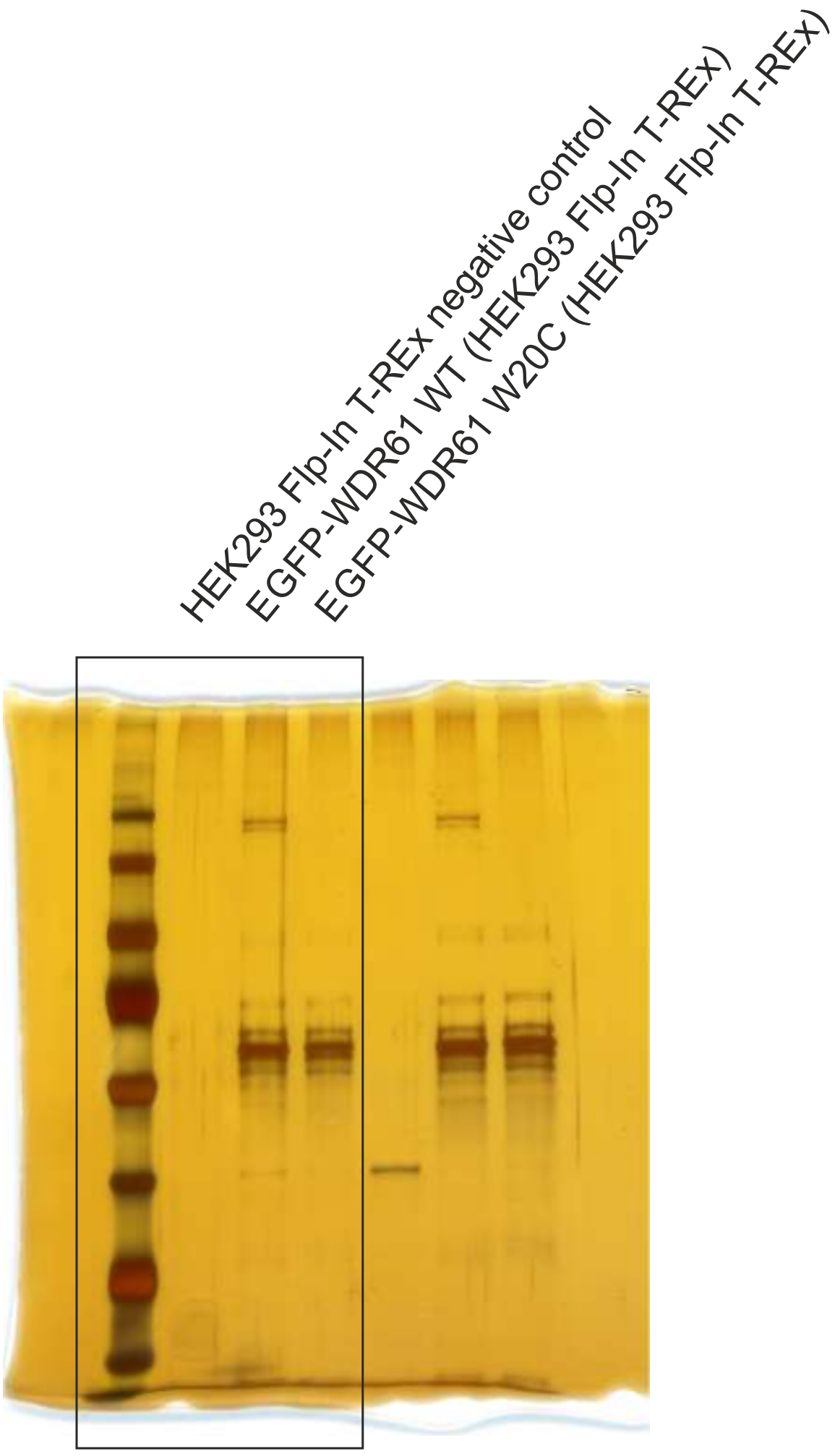

western-blot anti-EGFP (uncropped membrane)

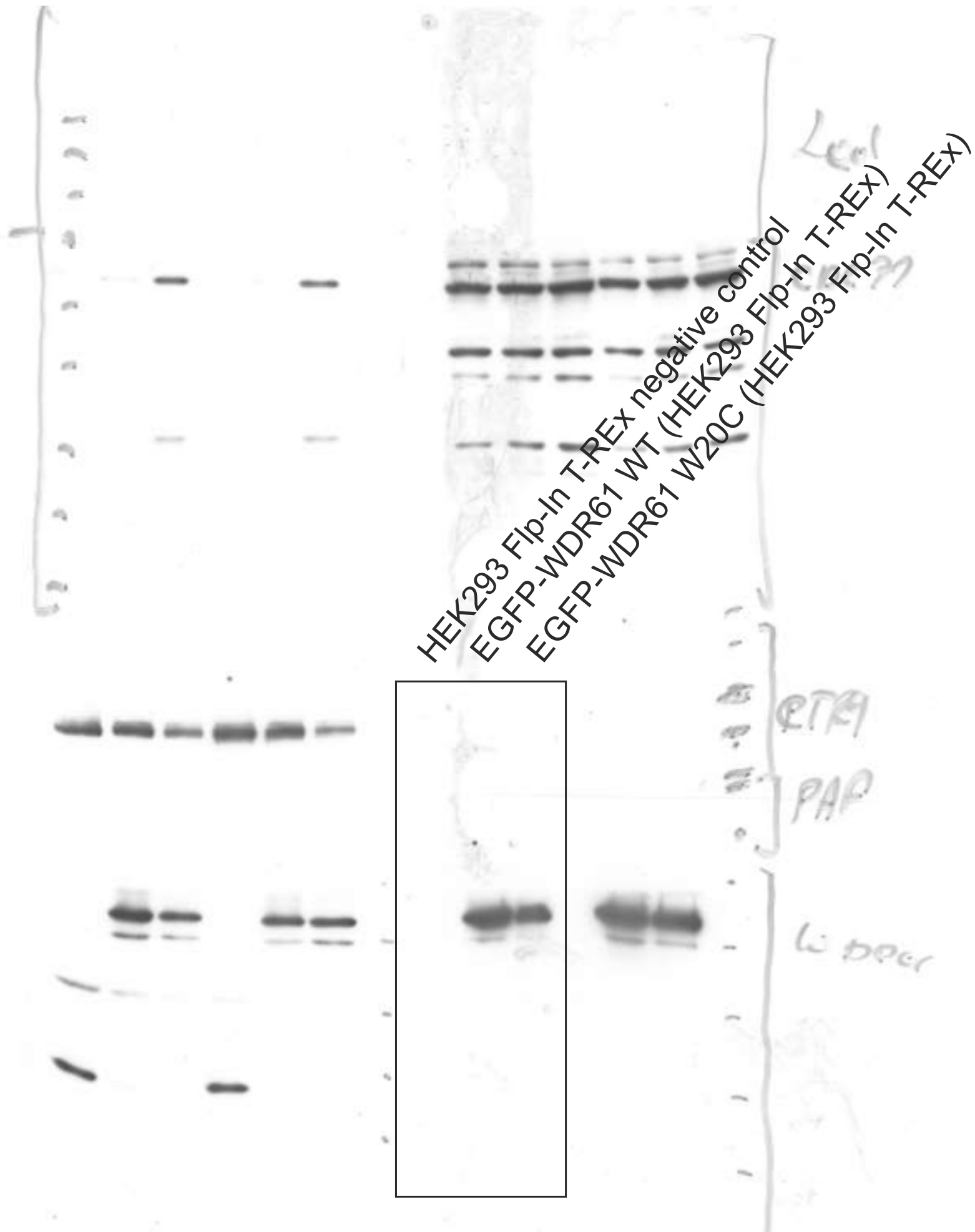

Supplementary Figure 2 f. coIP silver and WB of LF-MS MBP-CCDC97 samples

silver-stained SDS-PAGE

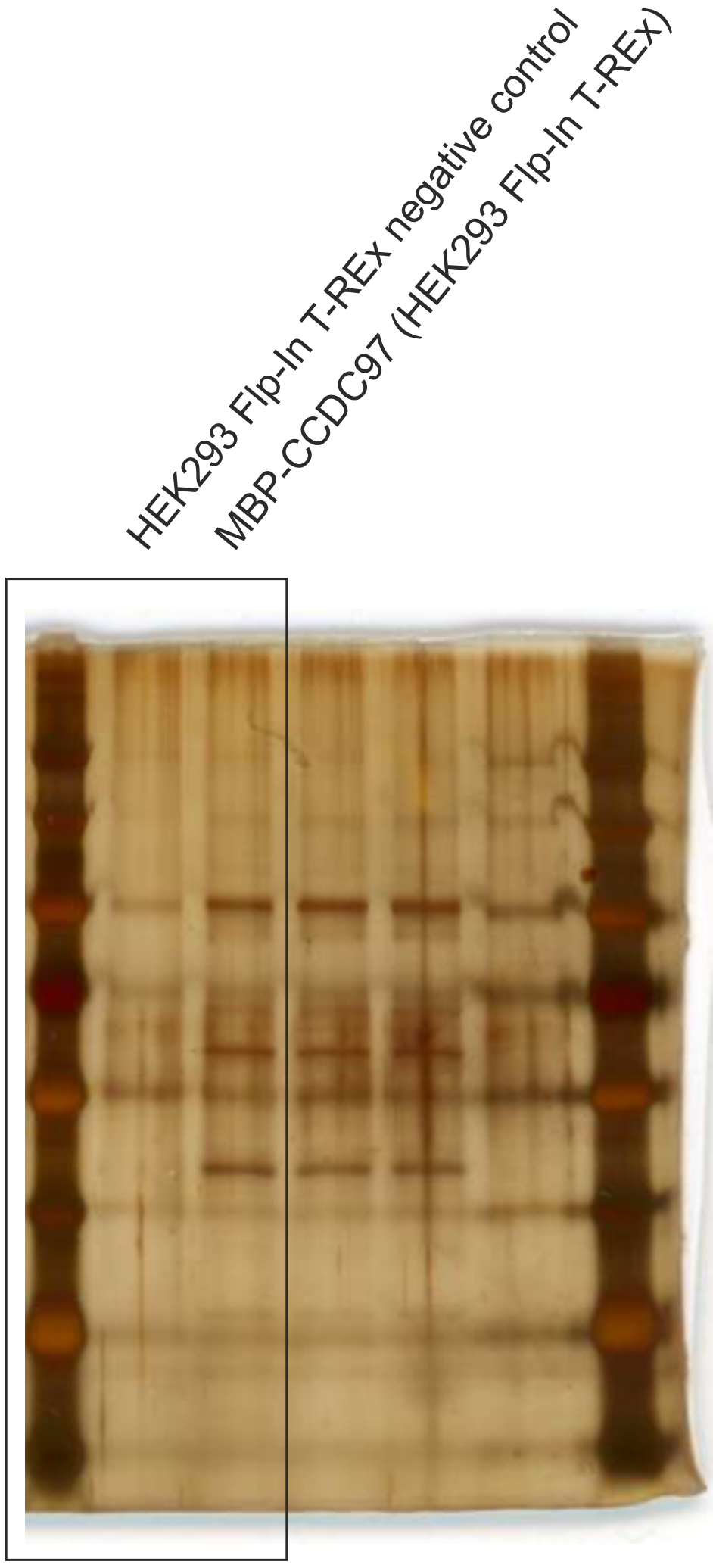

western-blot anti-MBP (uncropped membrane)

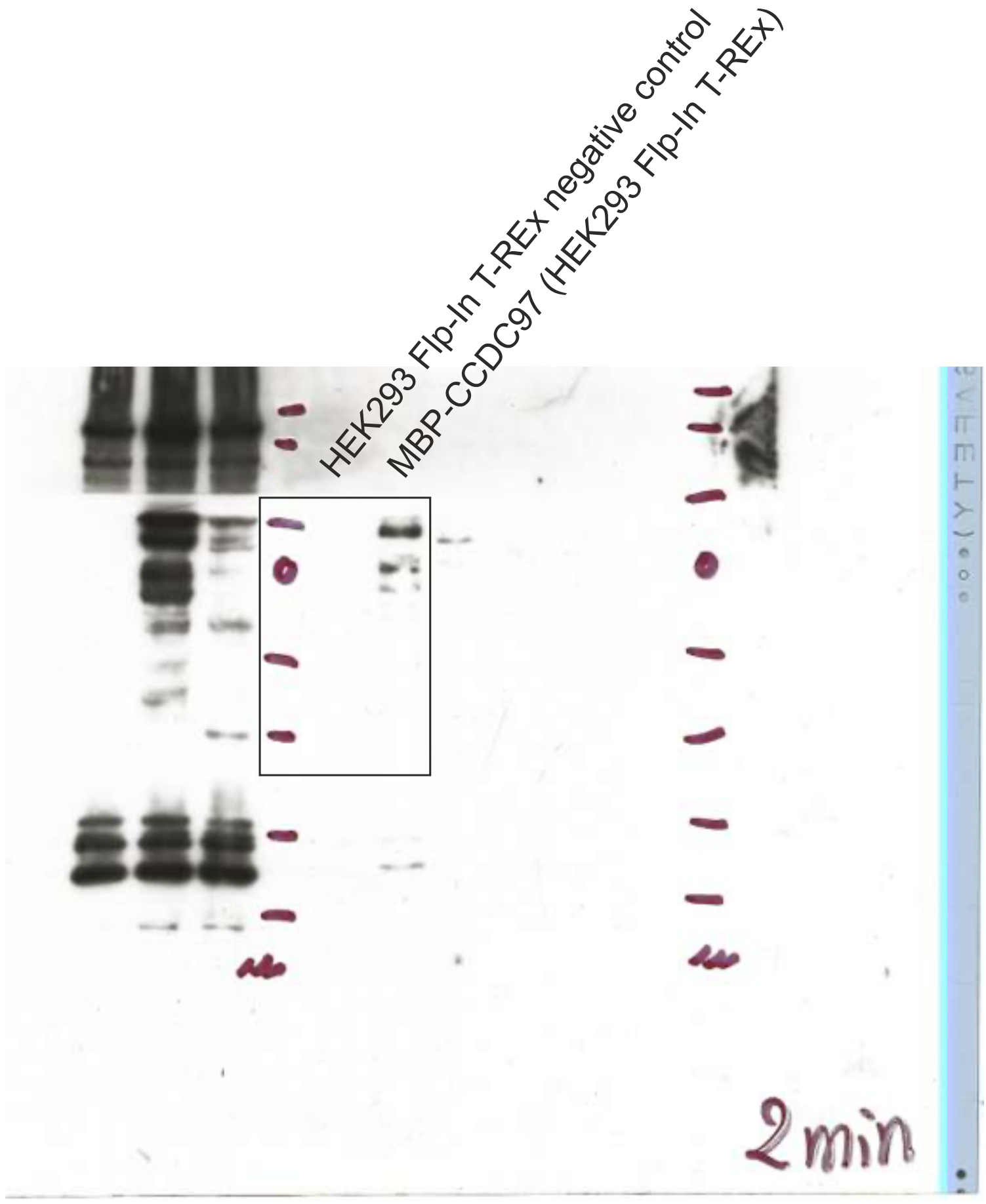

Supplementary Figure 2 g. coIP-WB TTC33-EGFP in 100 and 300 mM salt

western-blot results (uncropped membranes)

TTC33-EGFP

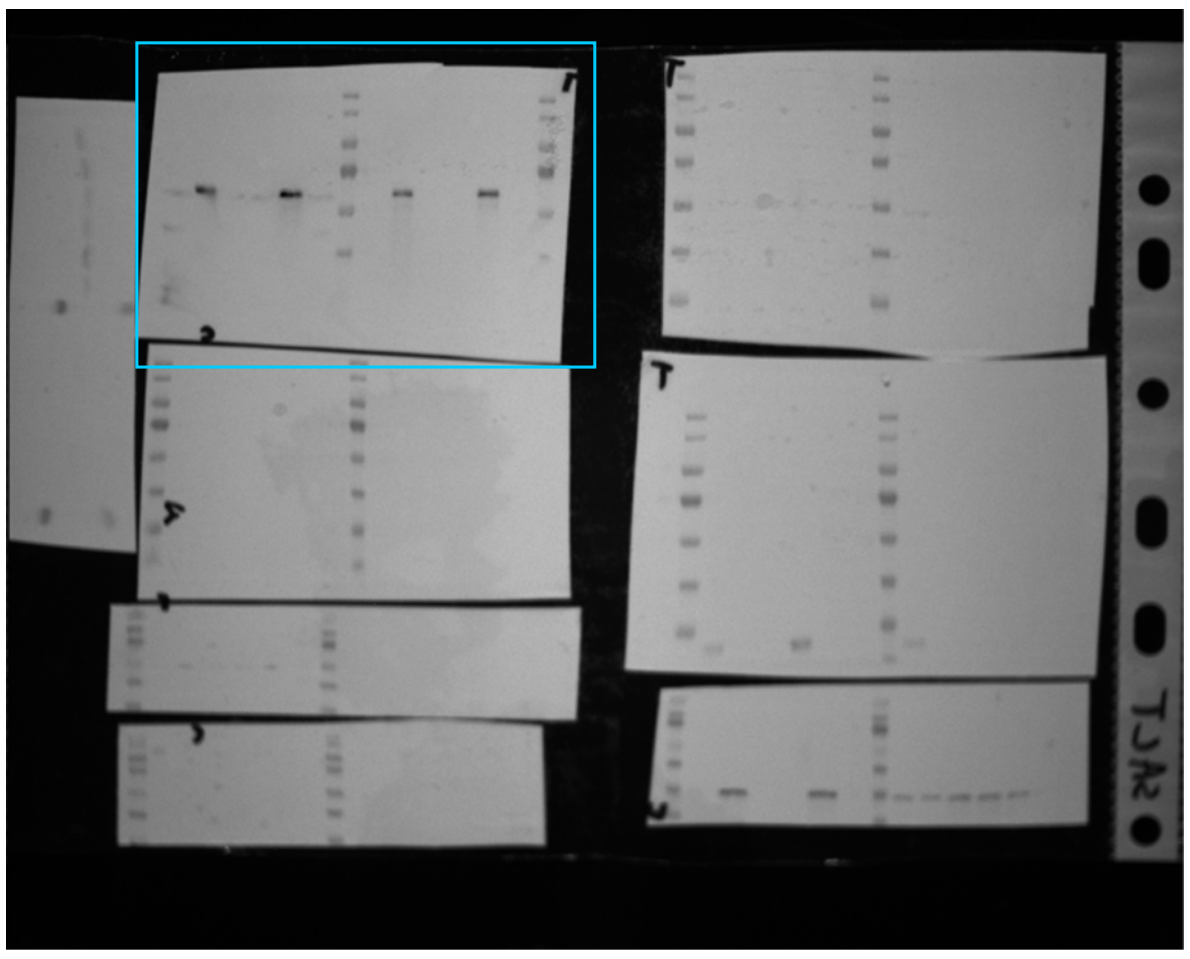

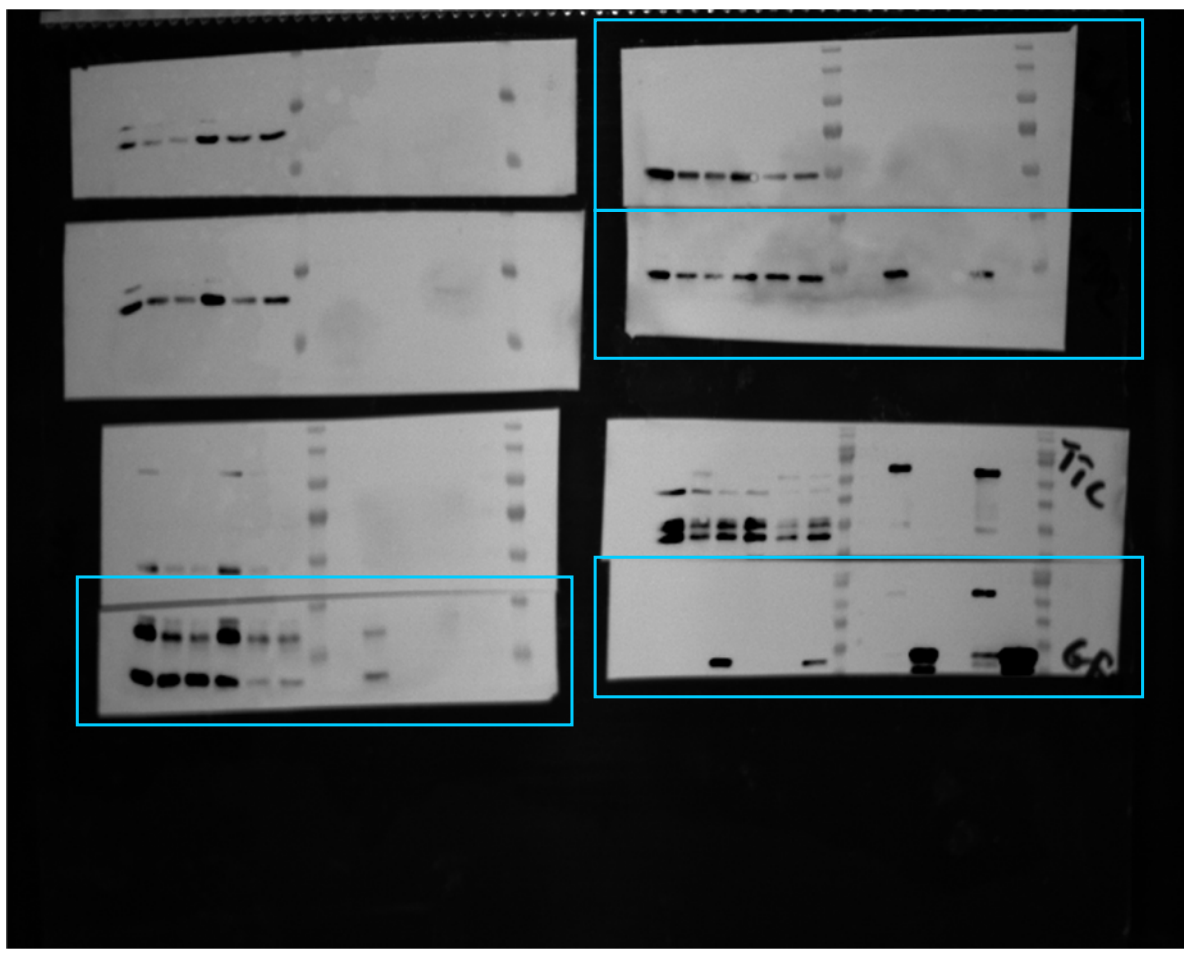

α-tubulin

WDR61

UNG

EGFP

PHF5A

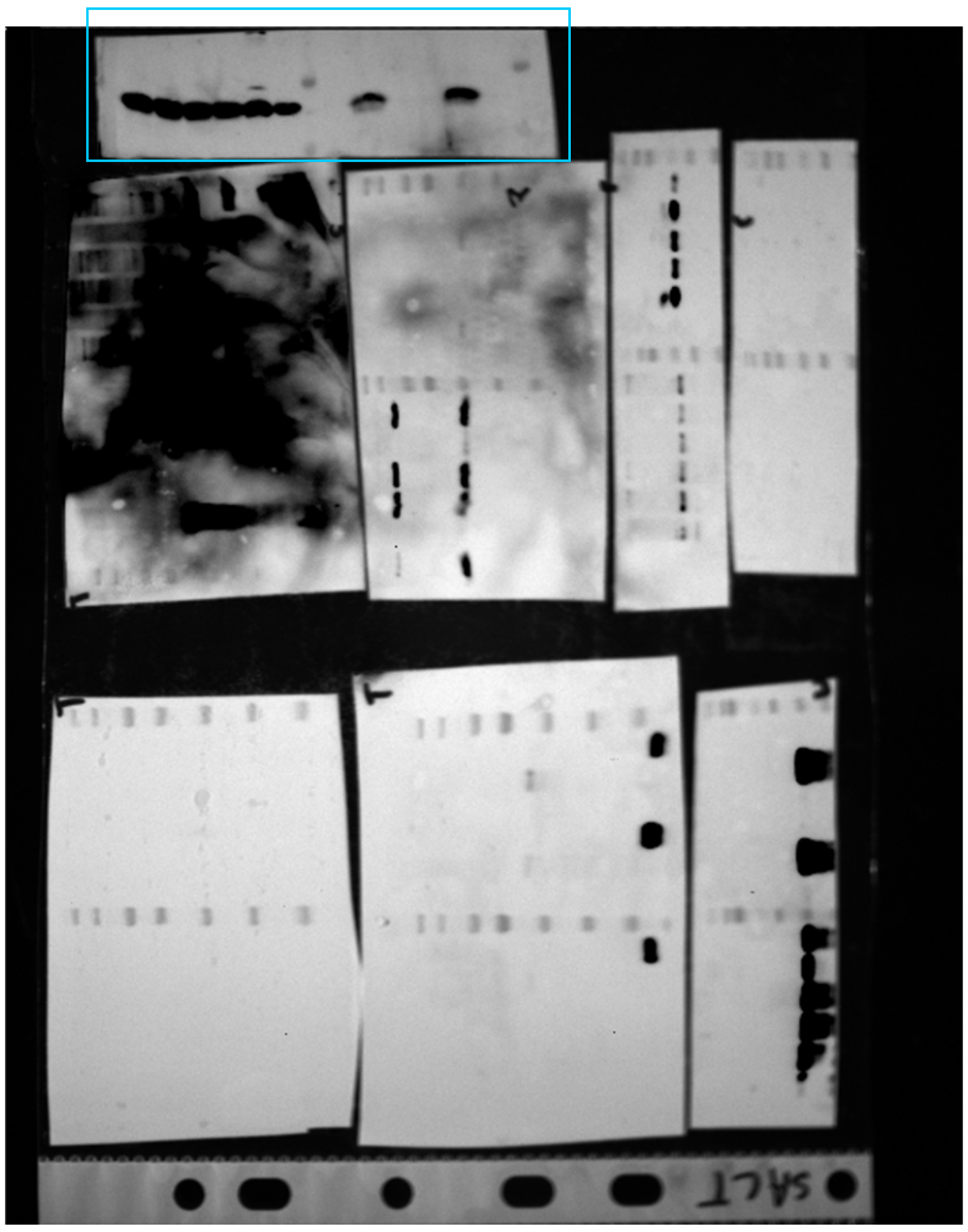

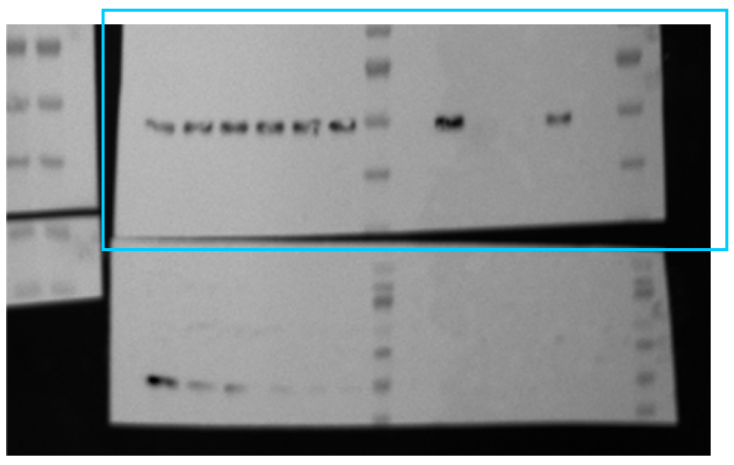

CCDC97

Supplementary Figure 2 h. coIP-WB MBP-TTC33 in 100 and 300 mM salt

western-blot results (uncropped membranes)

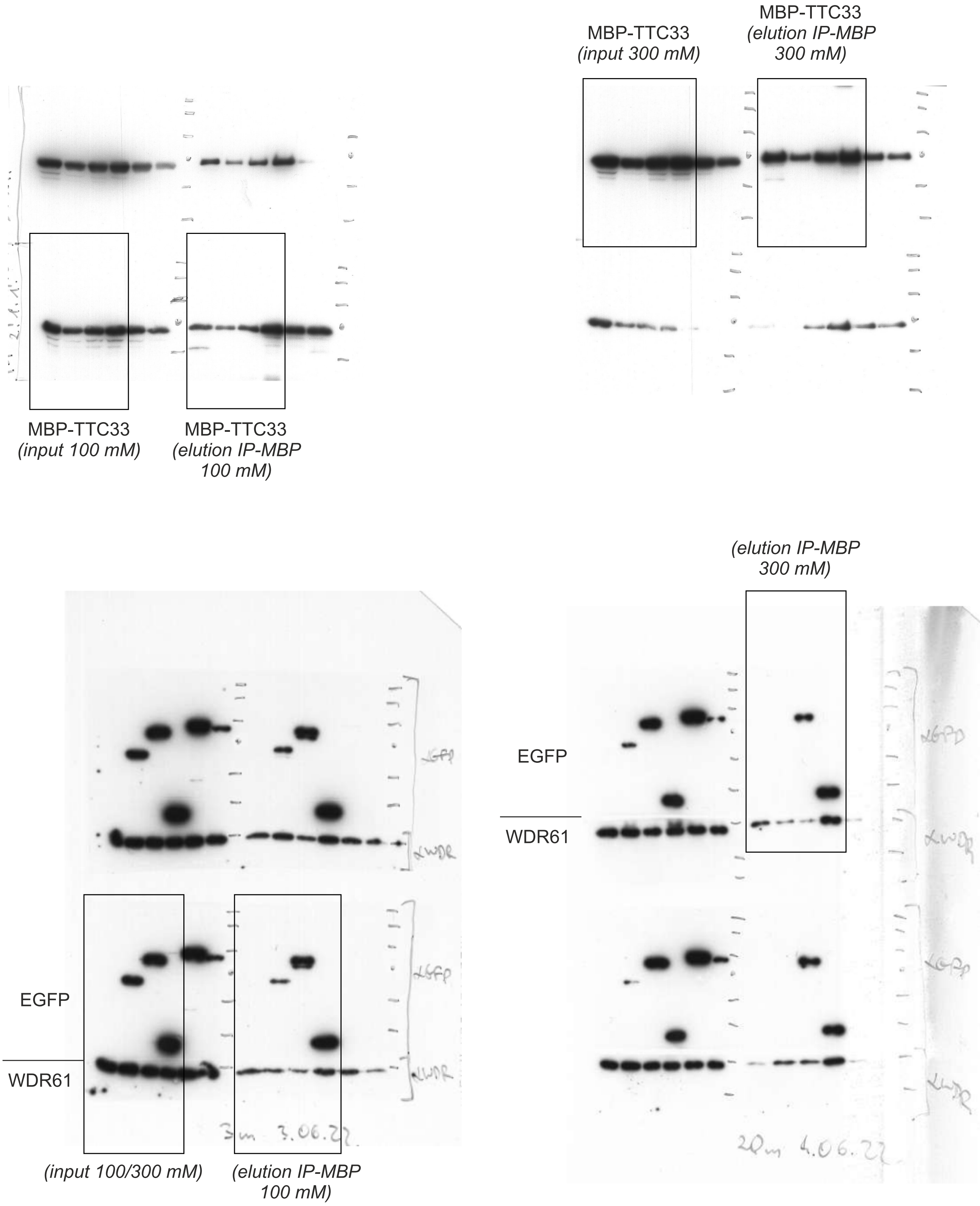

Supplementary Figure 3 a. Western-blot analyses of inputs used for testing the potential formation of a stable tetramer between TANC components and CCDC97 or UNG2

membranes after transfer stained with Ponceau S red

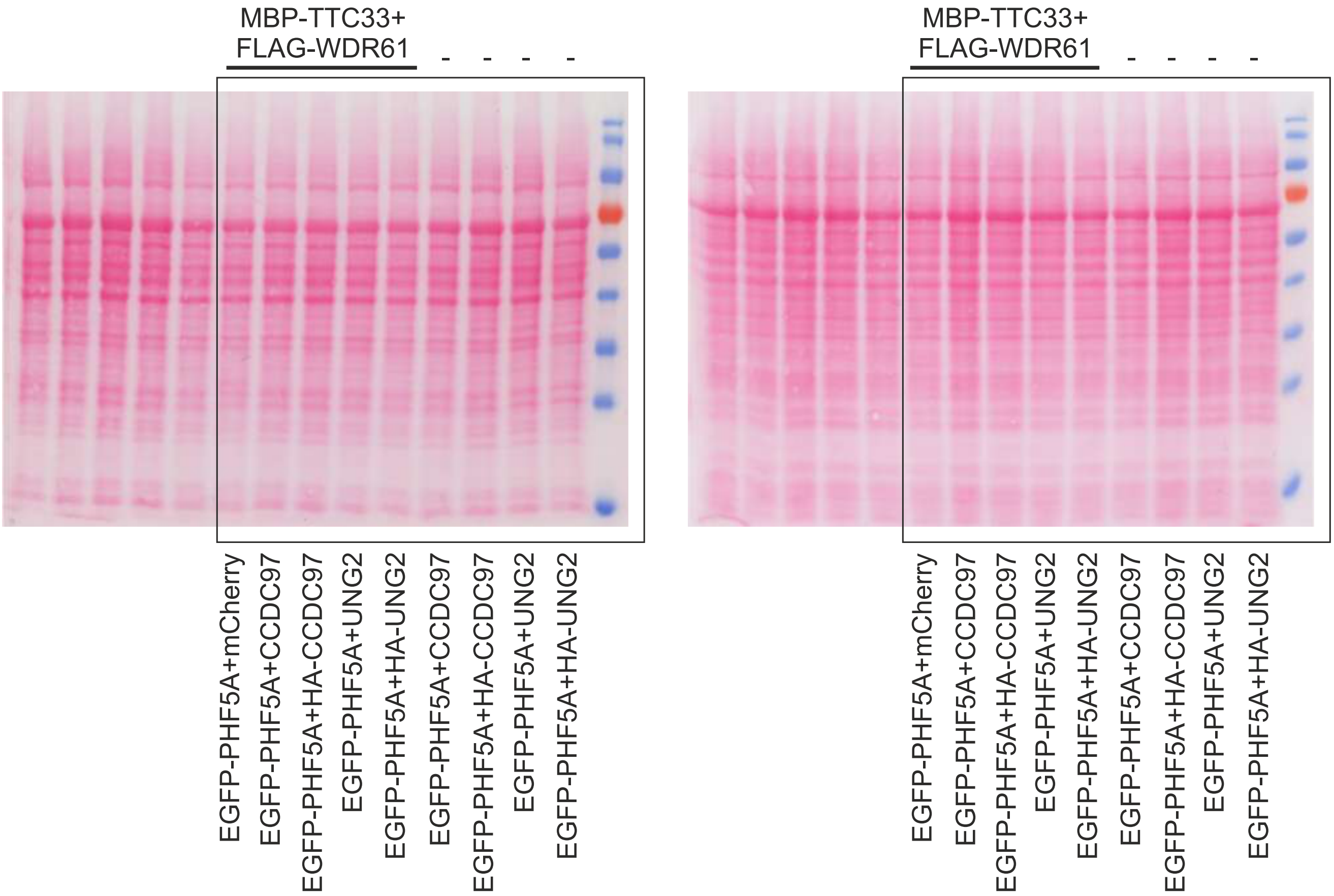

western-blot results (uncropped membranes)

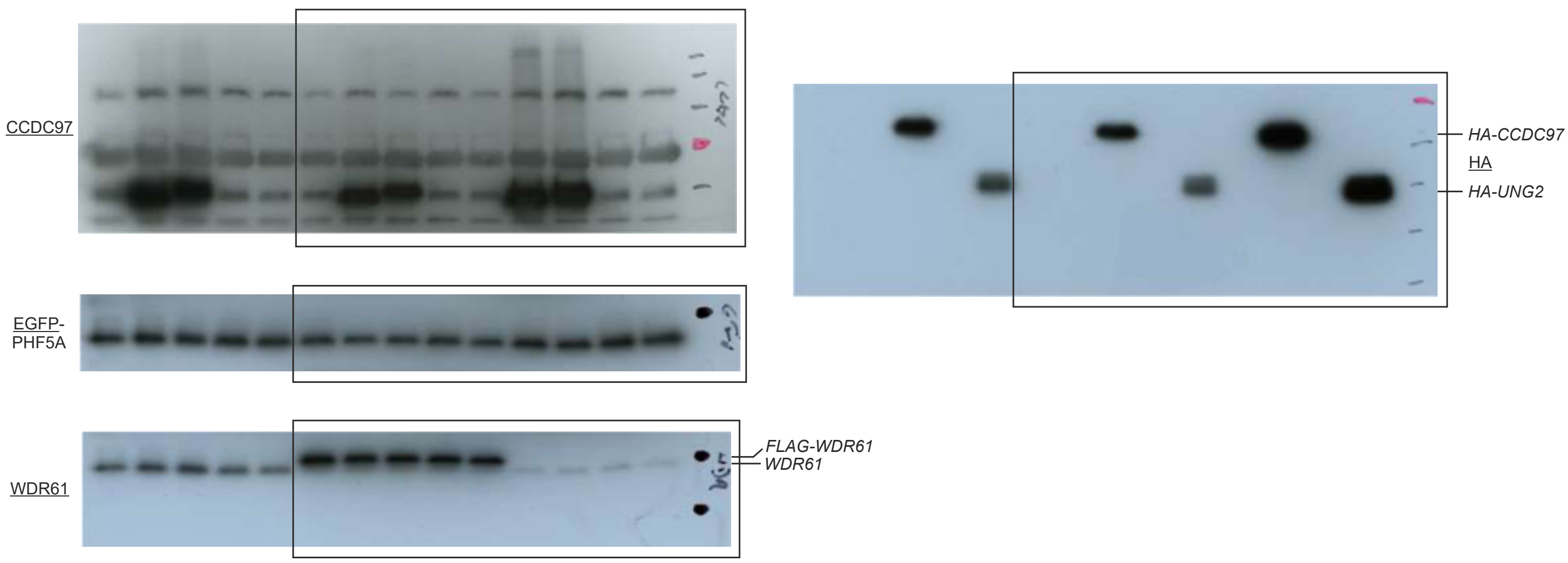

Supplementary Figure 3 a. Western-blot analyses of inputs used for testing the potential formation of a stable tetramer between TANC components and CCDC97 or UNG2 (continued)

membranes after transfer stained with Ponceau S red

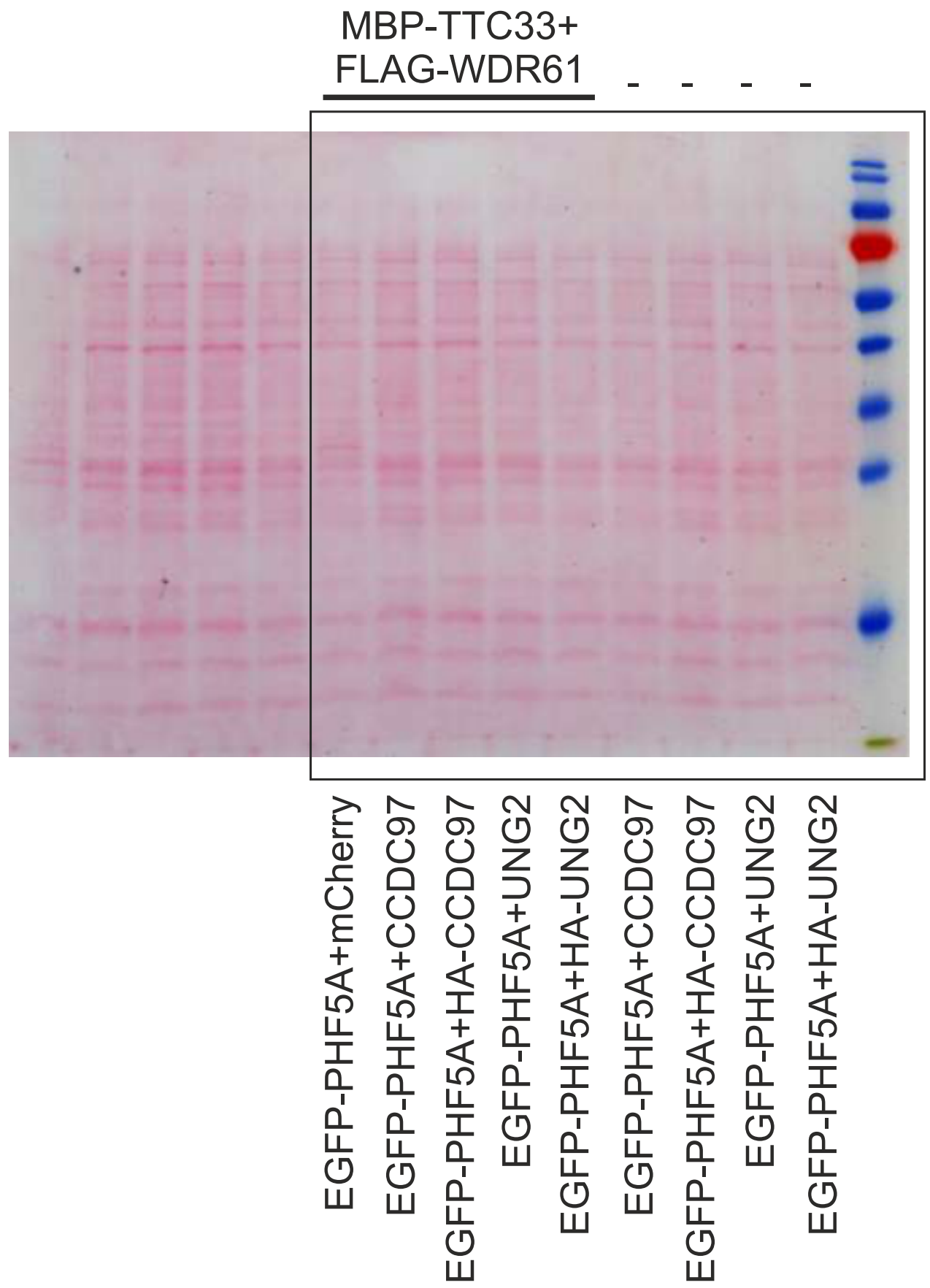

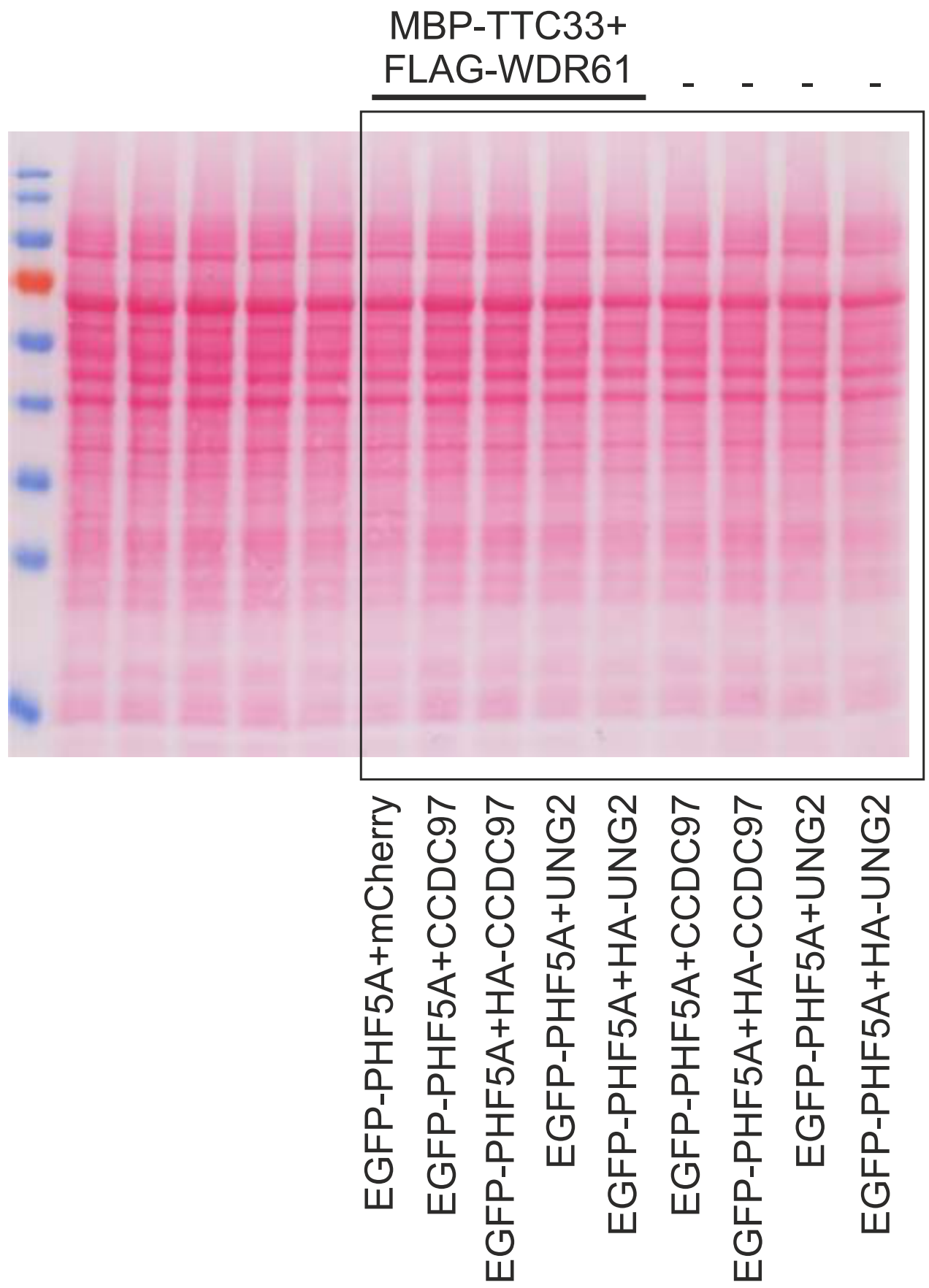

western-blot results (uncropped membranes)

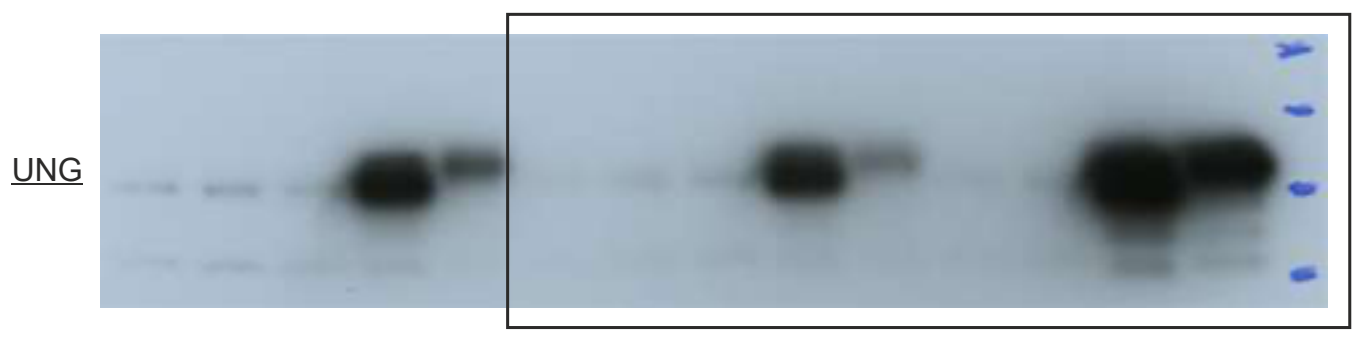

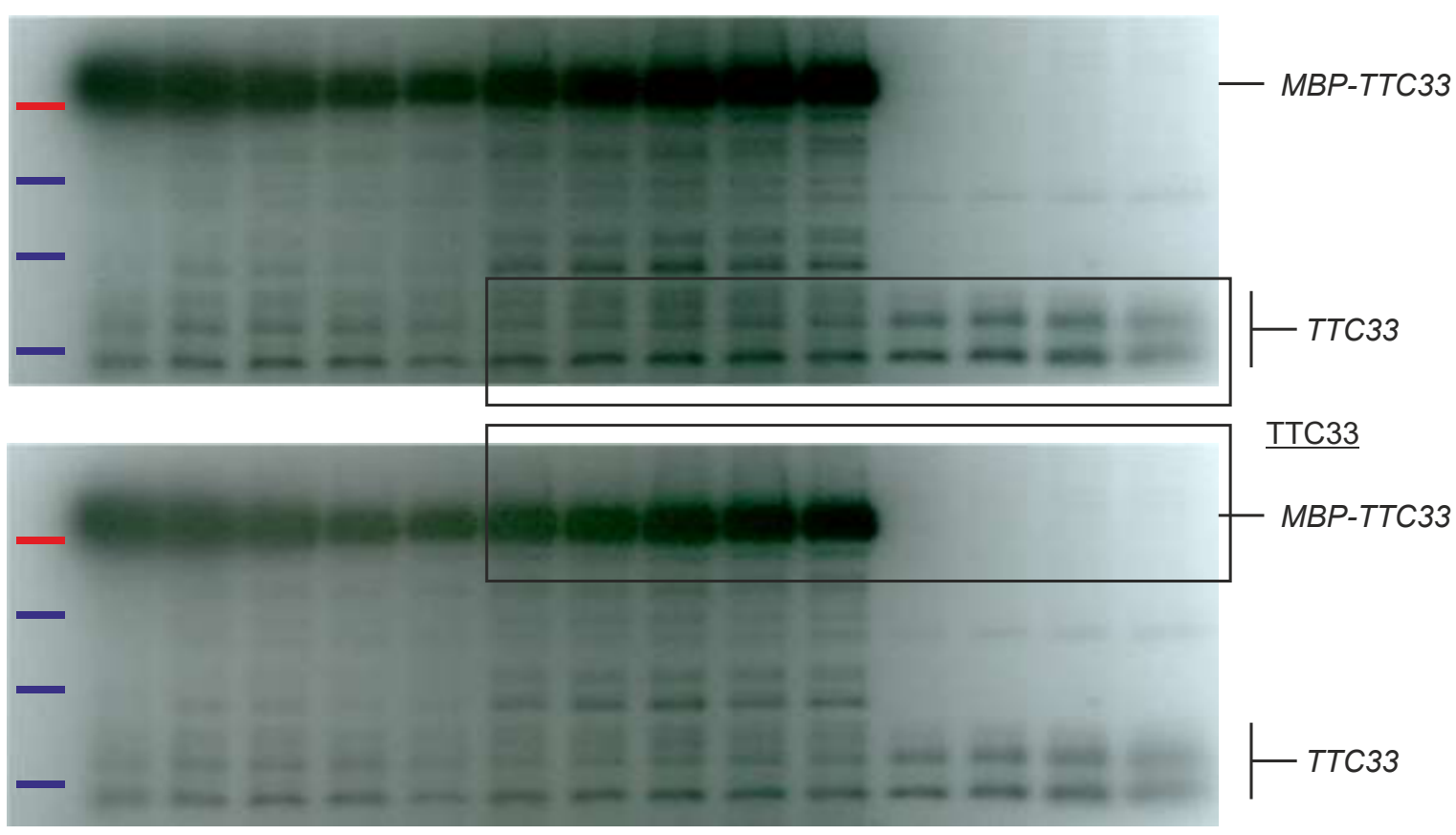

Supplementary Figure 3 a. Western-blot analyses of inputs used for testing the potential formation of a stable tetramer between TANC components and CCDC97 or UNG2 (continued)

membrane after transfer stained with Ponceau S red

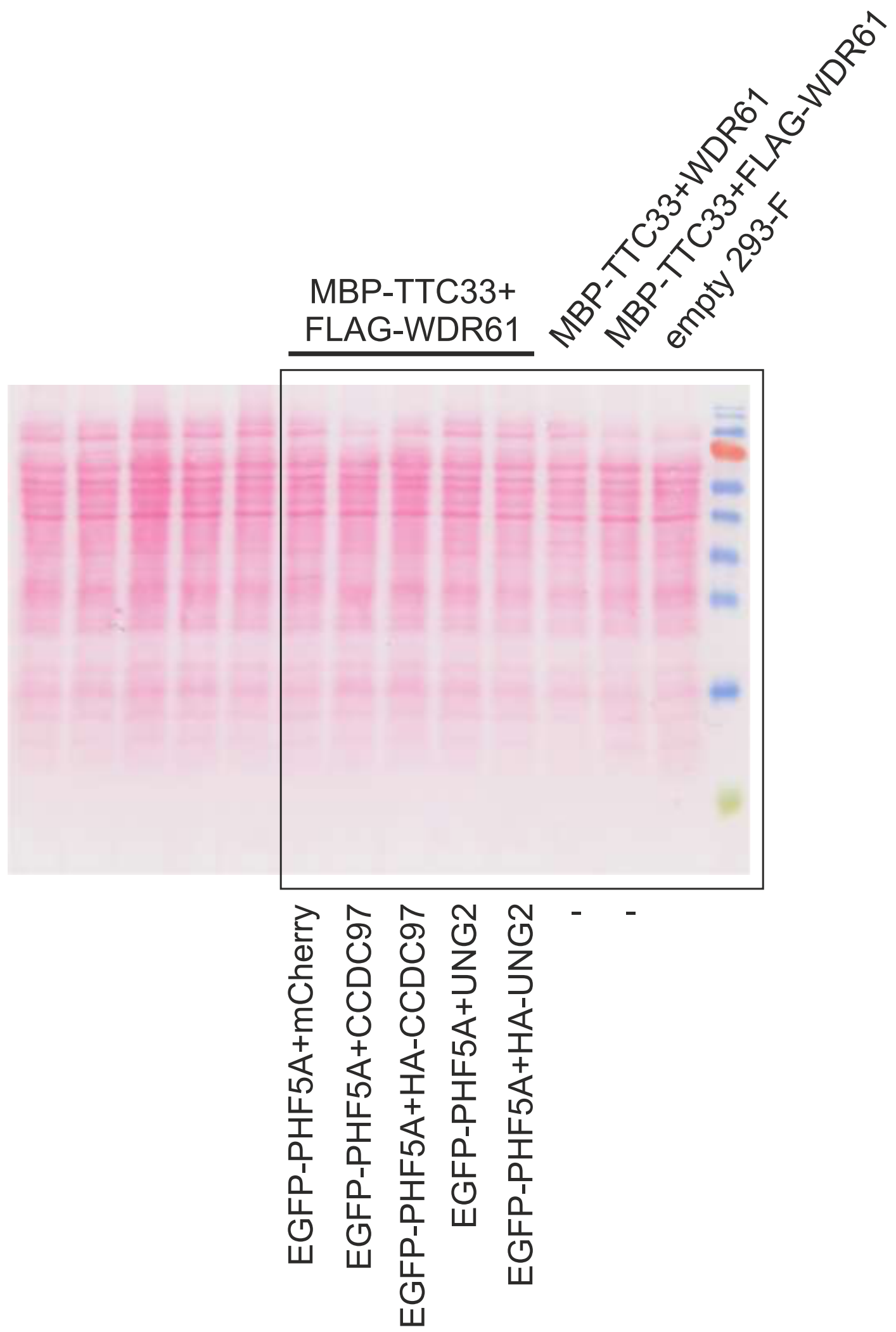

western-blot results (uncropped membrane)

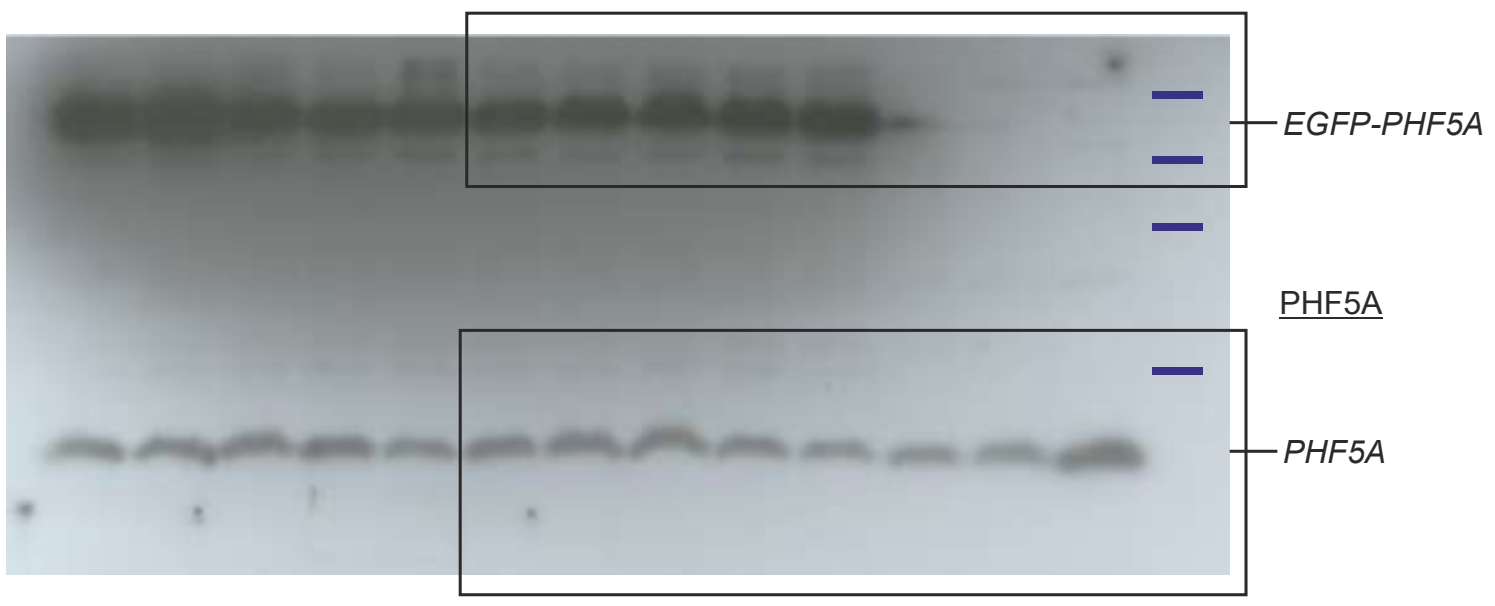

Supplementary Figure 3 b. Testing the potential formation of a stable tetramer between TANC components and CCDC97 using MBP- or EGFP-tagged CCDC97 as bait

Silver-stained SDS-PAGE gel with eluates after affinity chromatography on amylose or GFP-trap resin

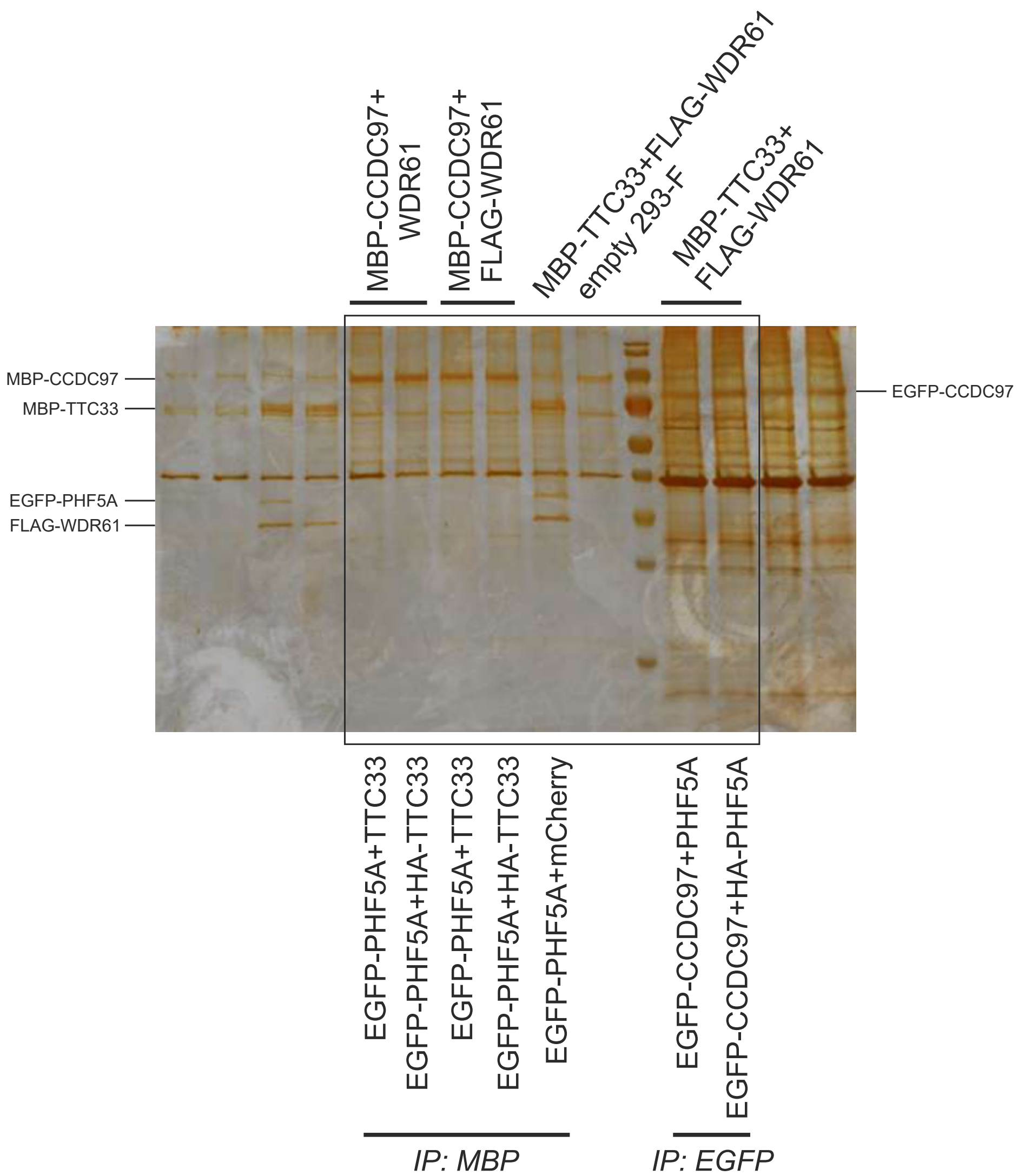

Supplementary Figure 3 c. Analyses of fractions from size exclusion chromatography (SEC) experiments

Silver-stained SDS-PAGE gels with selected SEC fractions for samples
MBP-TTC33+FLAG-WDR61 / EGFP-PHF5A,
MBP-TTC33+FLAG-WDR61 / EGFP-PHF5A+CCDC97,
MBP-TTC33+FLAG-WDR61 / EGFP-PHF5A+HA-CCDC97,
and MBP-TTC33+FLAG-WDR61 / EGFP-PHF5A+HA-UNG2

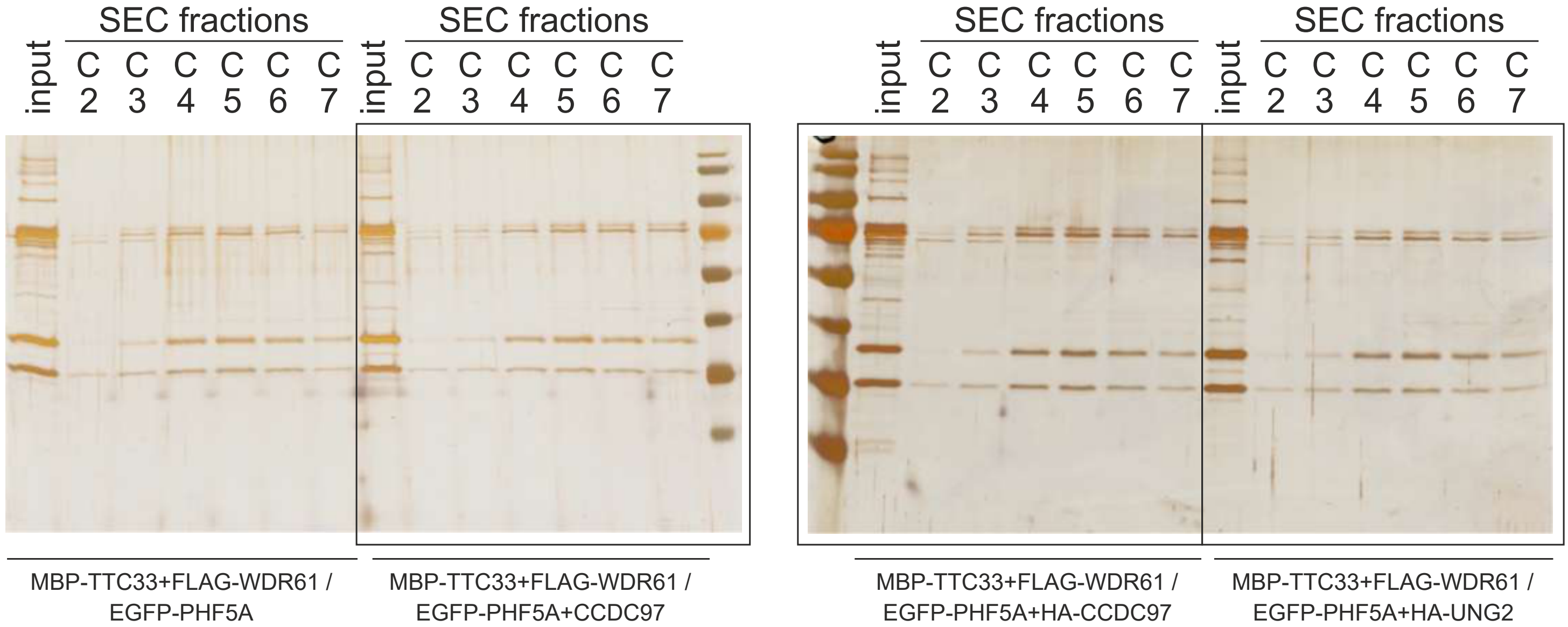

western-blot results (uncropped membranes)

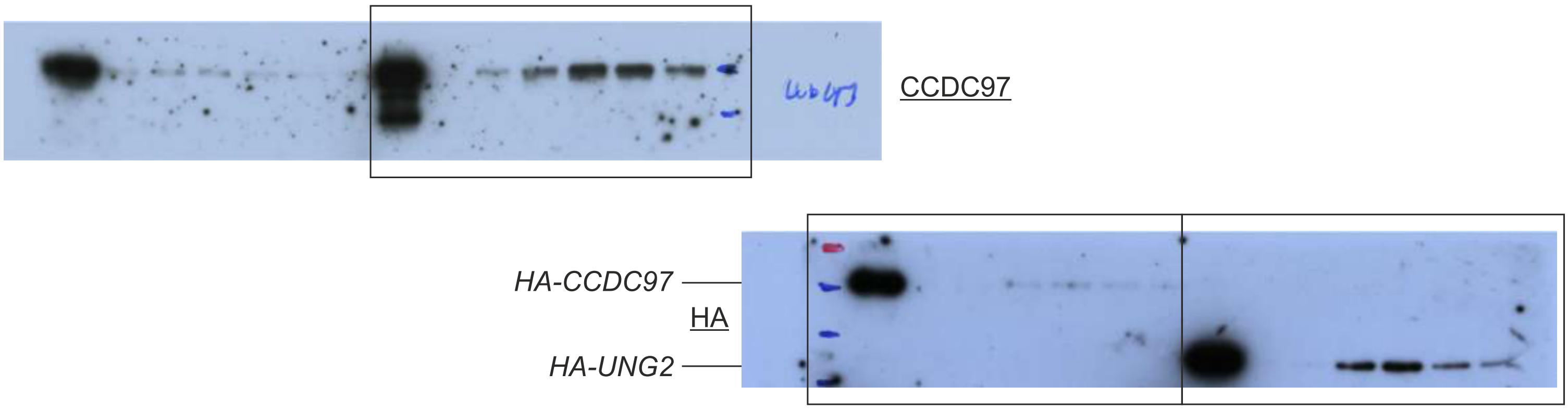

Supplementary Figure 3 d. Separation of whole cell extract from FreeStyle 293-F suspension cells in the glycerol gradient

membranes after transfer stained with Ponceau S red

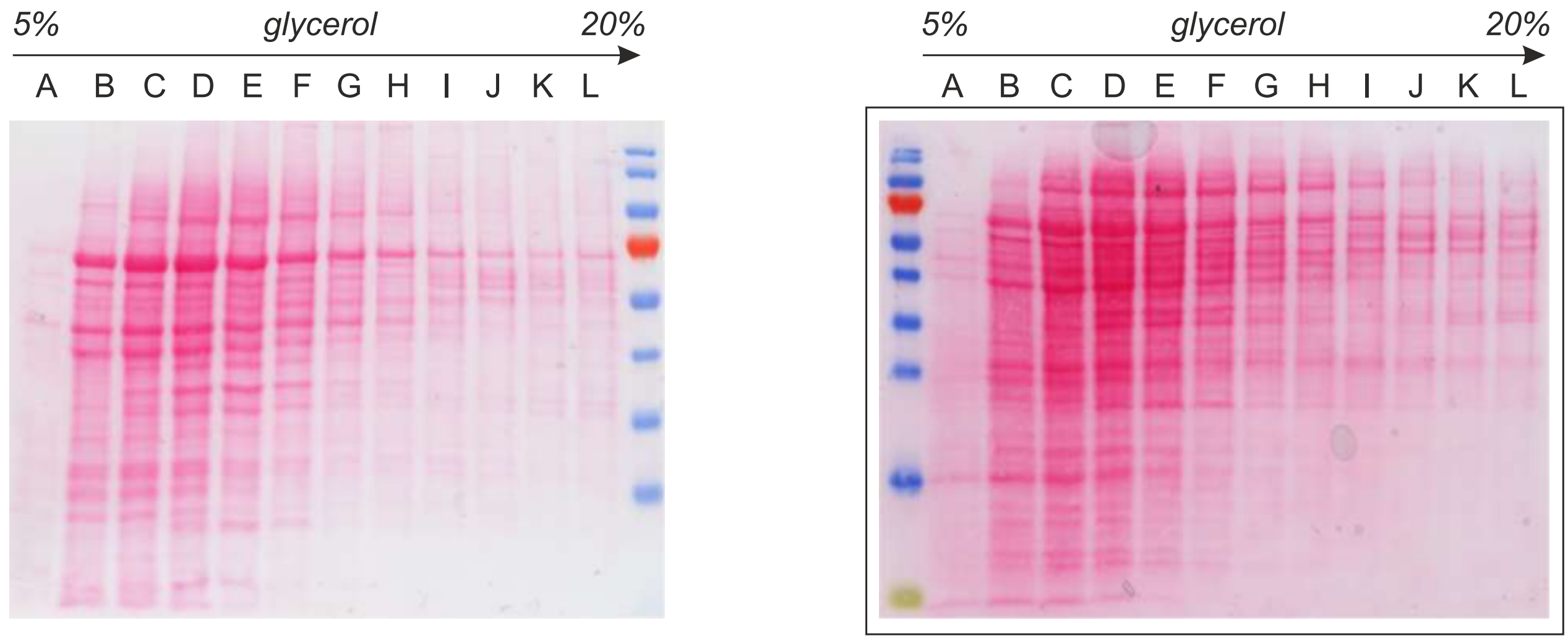

western-blot results (uncropped membranes)

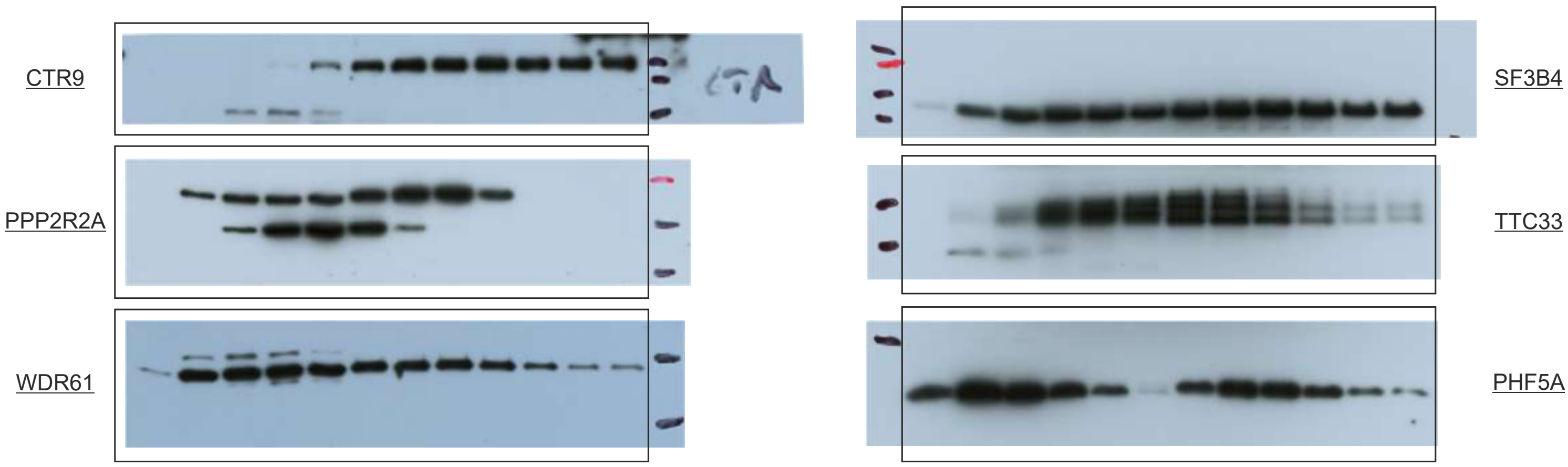

Supplementary Figure 3 d. Separation of whole cell extract from FreeStyle 293-F suspension cells in the glycerol gradient (continued)

membranes after transfer stained with Ponceau S red

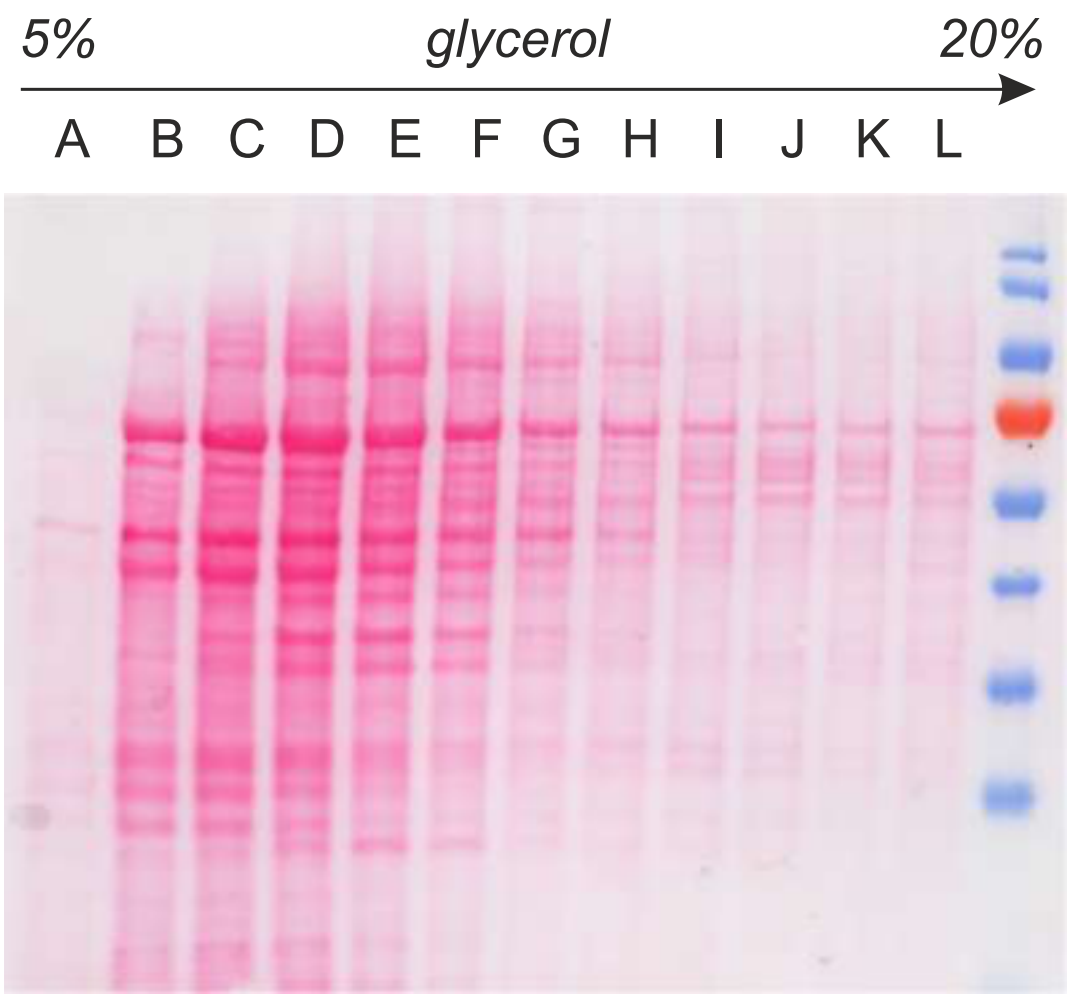

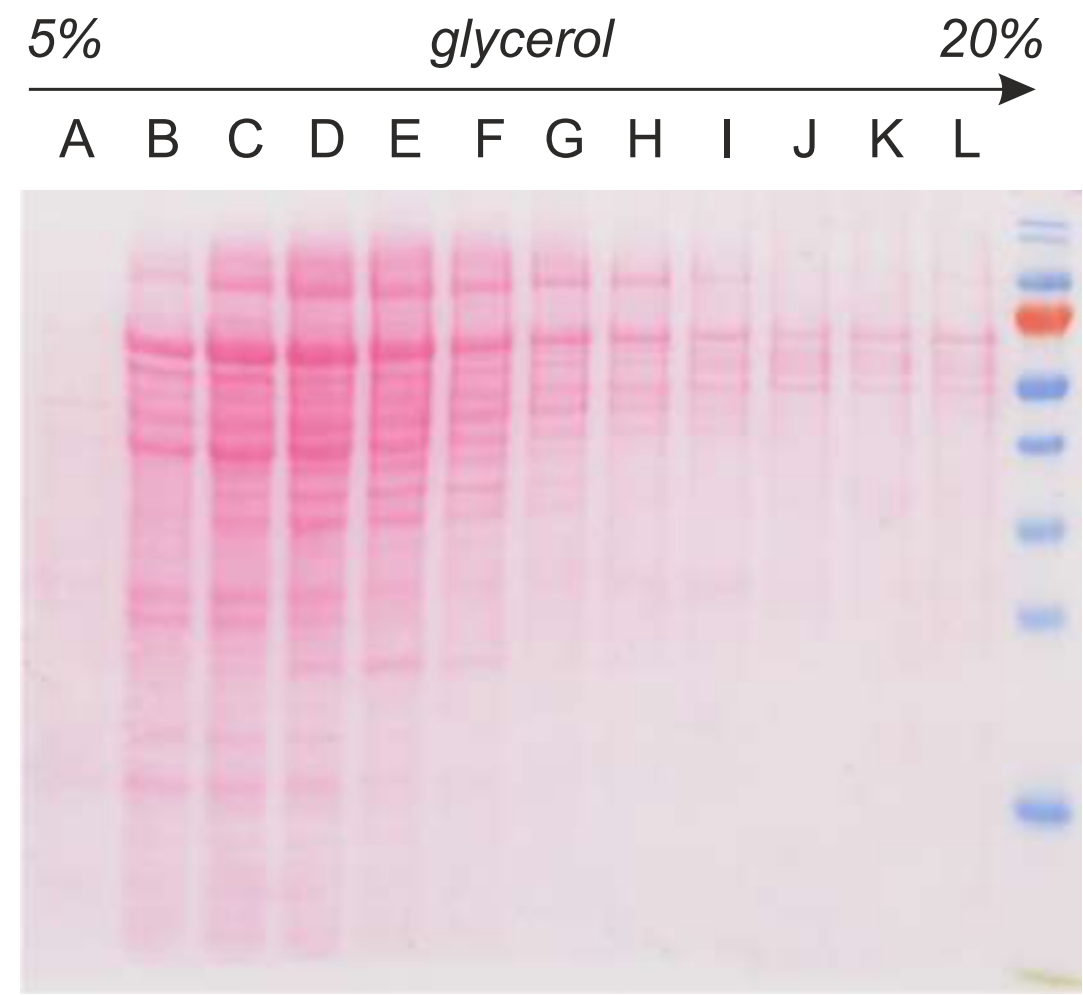

western-blot results (uncropped membranes)

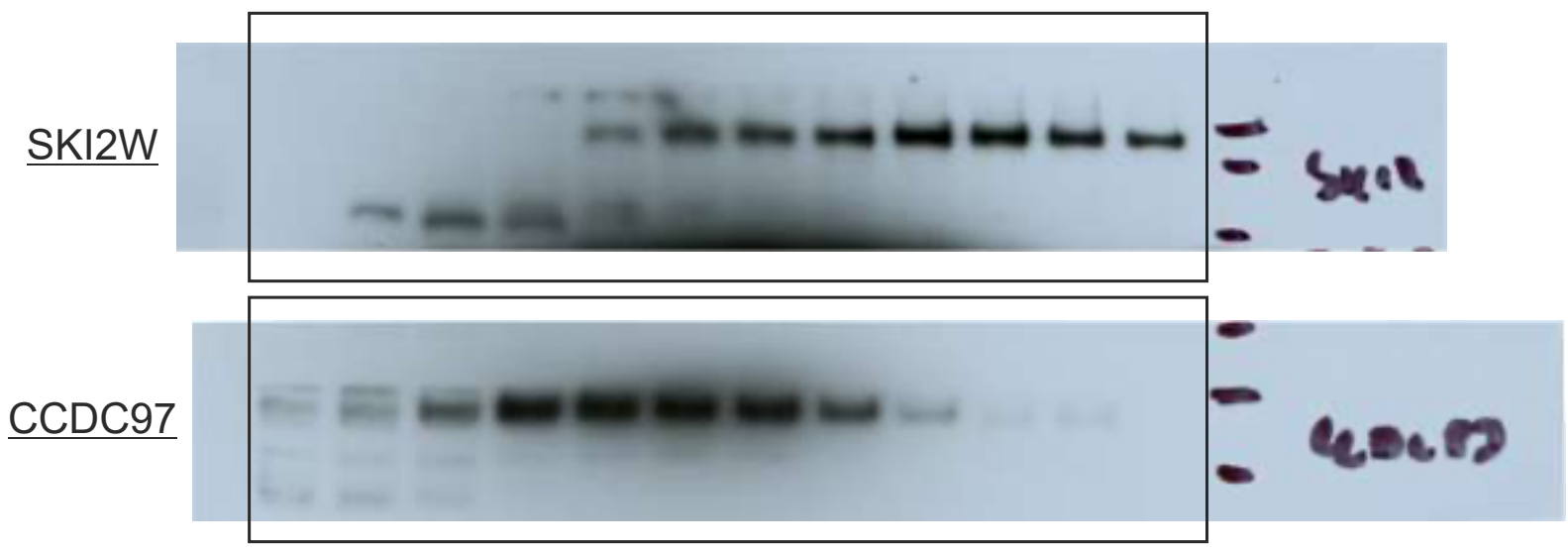

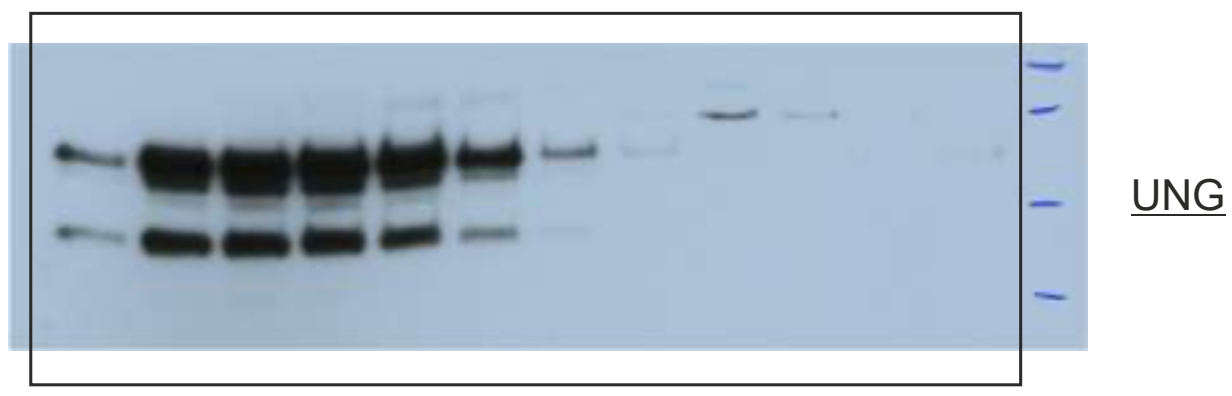

Supplementary Figure 5 c. Immunoprecipitation of MBP-tagged TTC33 WT and mutants with single amino acid substitions from HeLa Flp-In T-REx stable cell lines

left panel

membranes after transfer stained with Ponceau S red

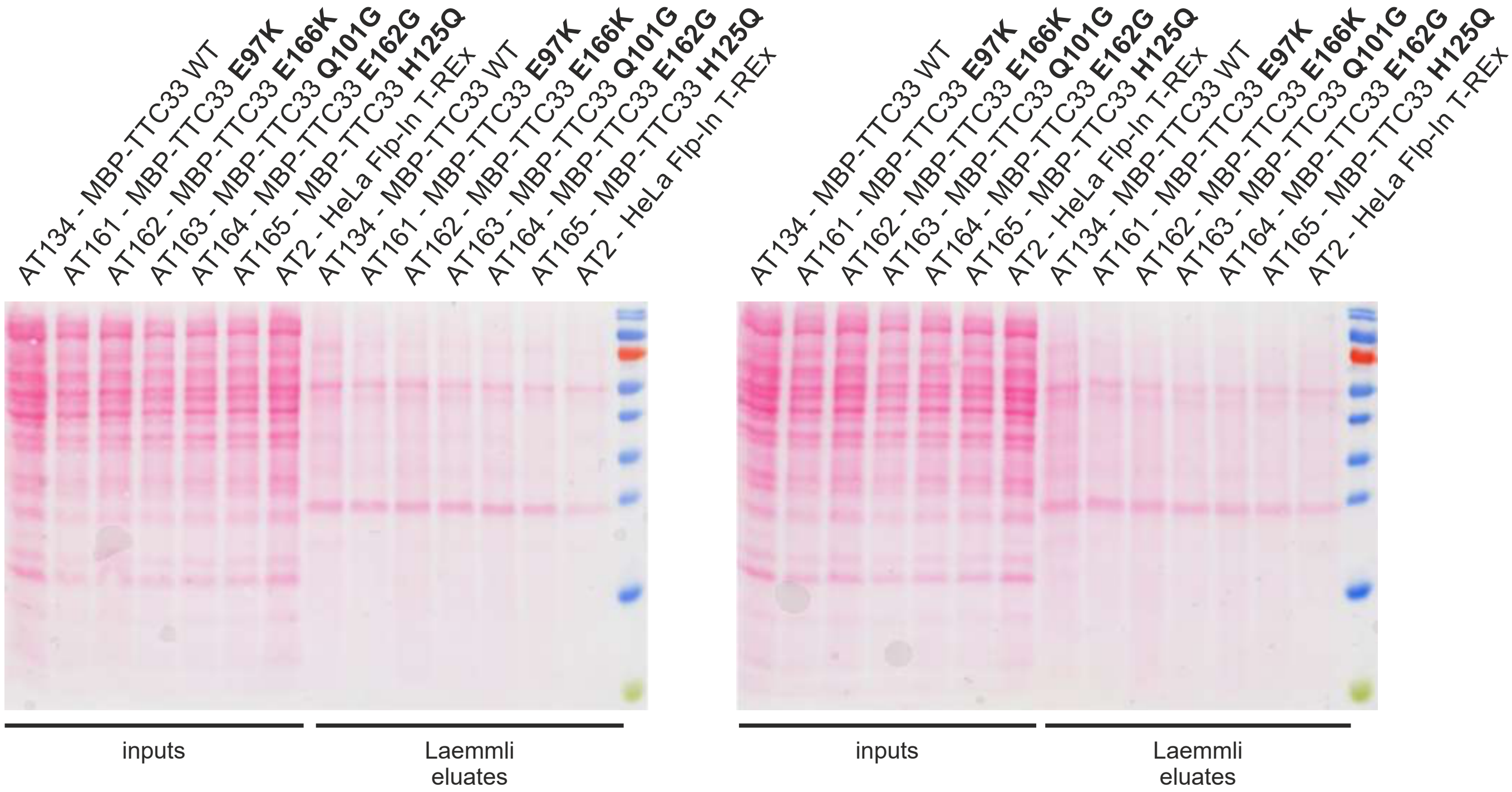

western-blot results (uncropped membranes)

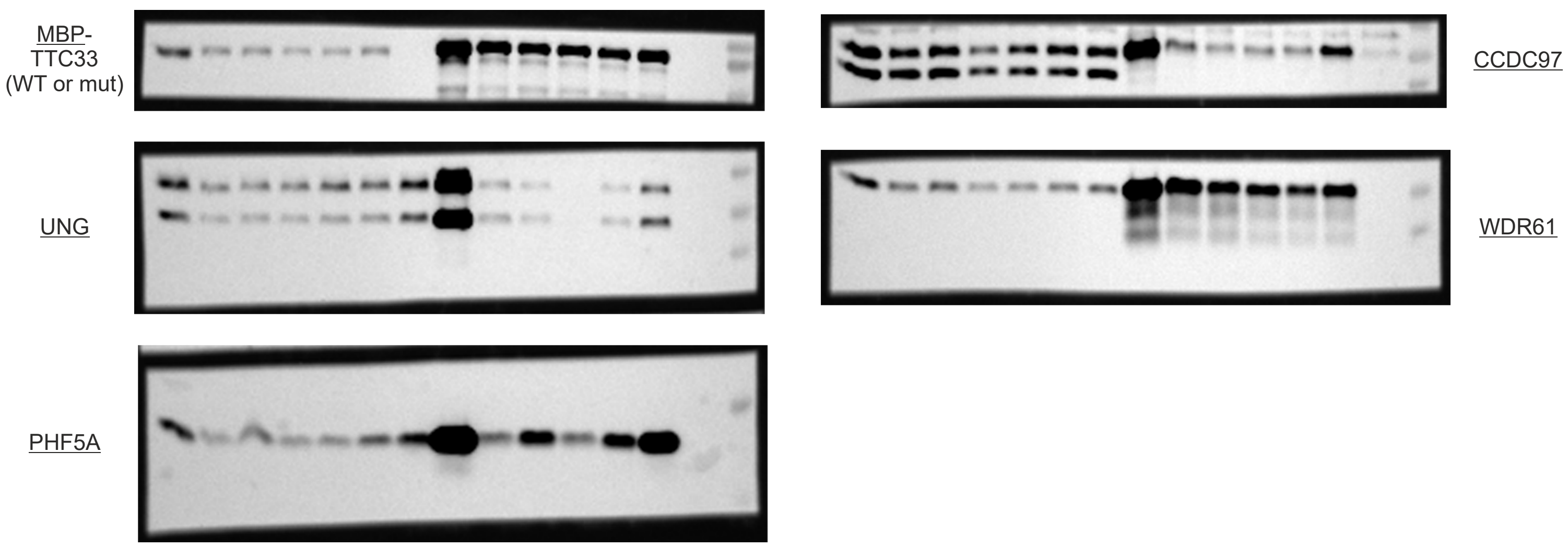

Supplementary Figure 5 c. Immunoprecipitation of MBP-tagged TTC33 WT and mutants with single amino acid substitions from HeLa Flp-In T-REx stable cell lines (continued)

right panel

membranes after transfer stained with Ponceau S red

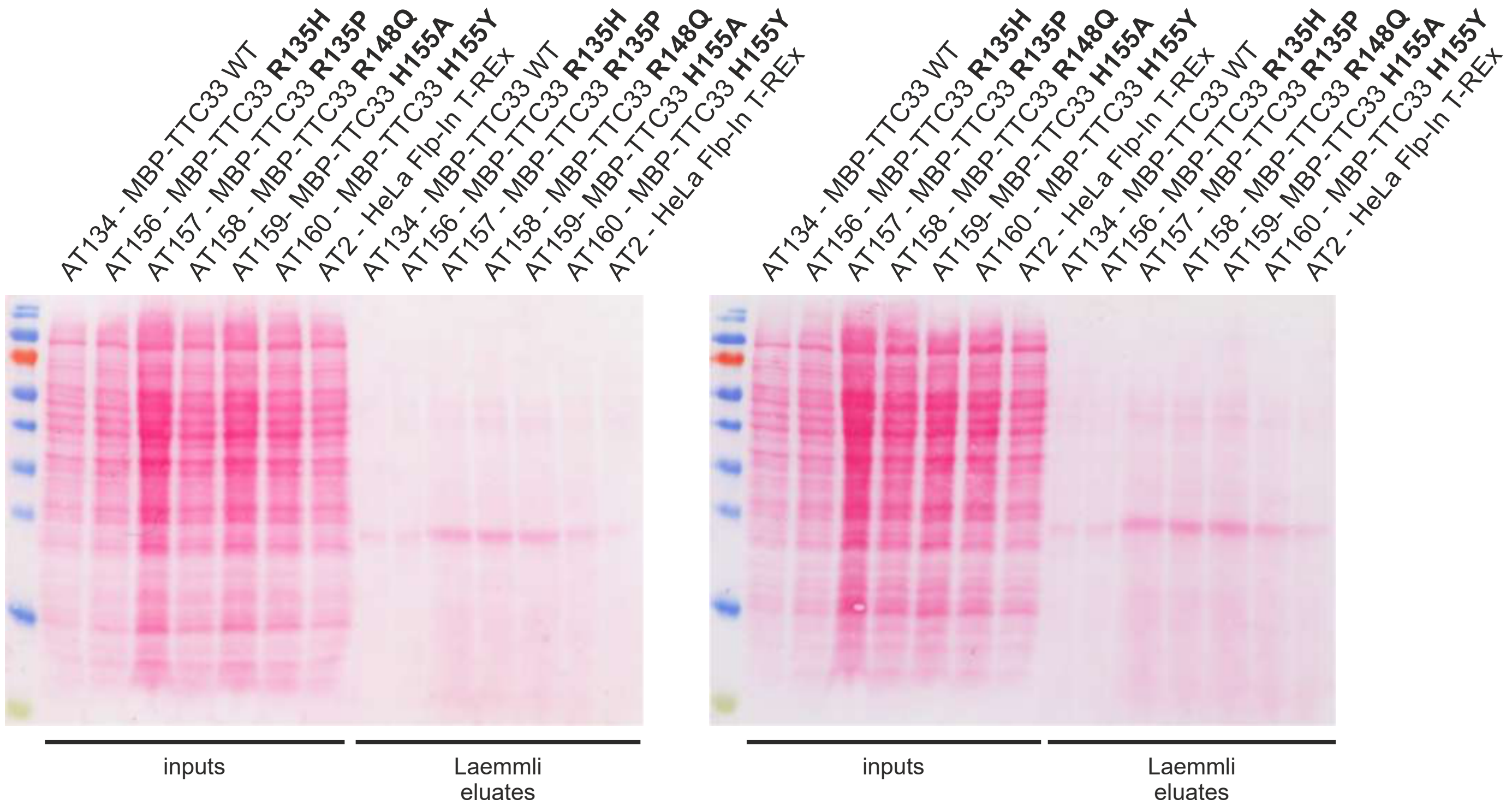

western-blot results (uncropped membranes)

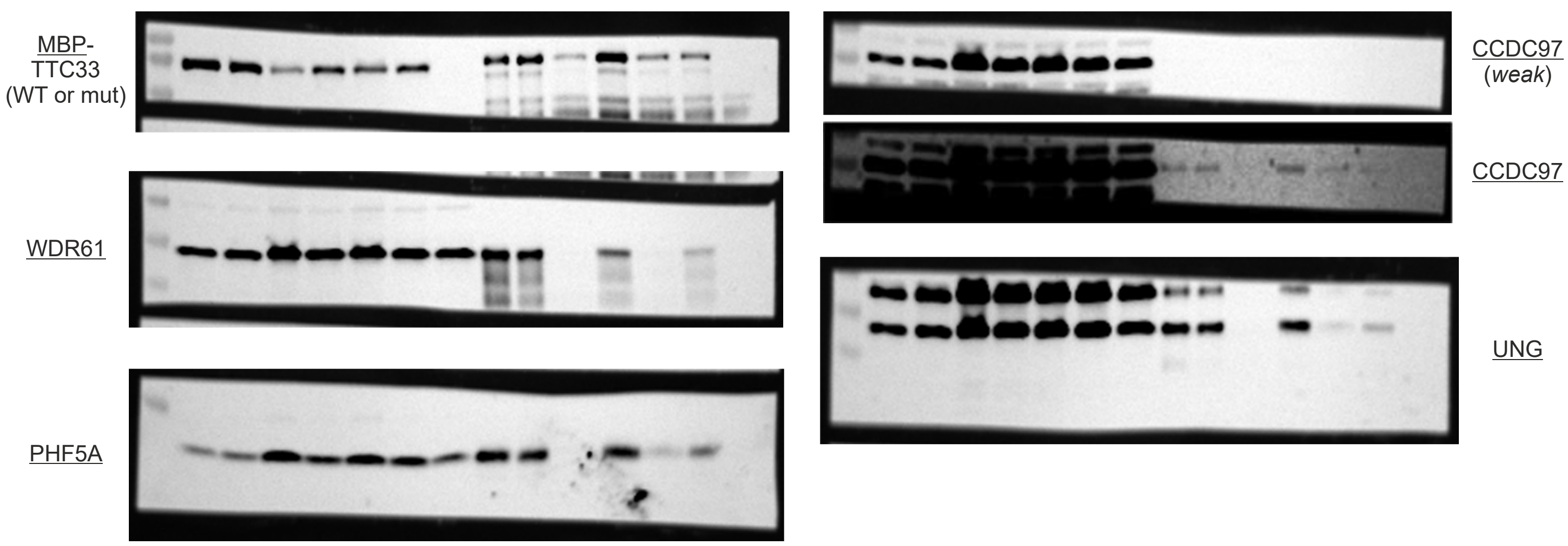

Supplementary Figure 6 b. EGFP-tagged TTC33 fragments with cotransfected MBP-tagged CCDC97 or PPP2R2A

western-blot results (uncropped membranes)

MBP-CCDC97

TTC33-EGFP
(FL or fragments)

WDR61

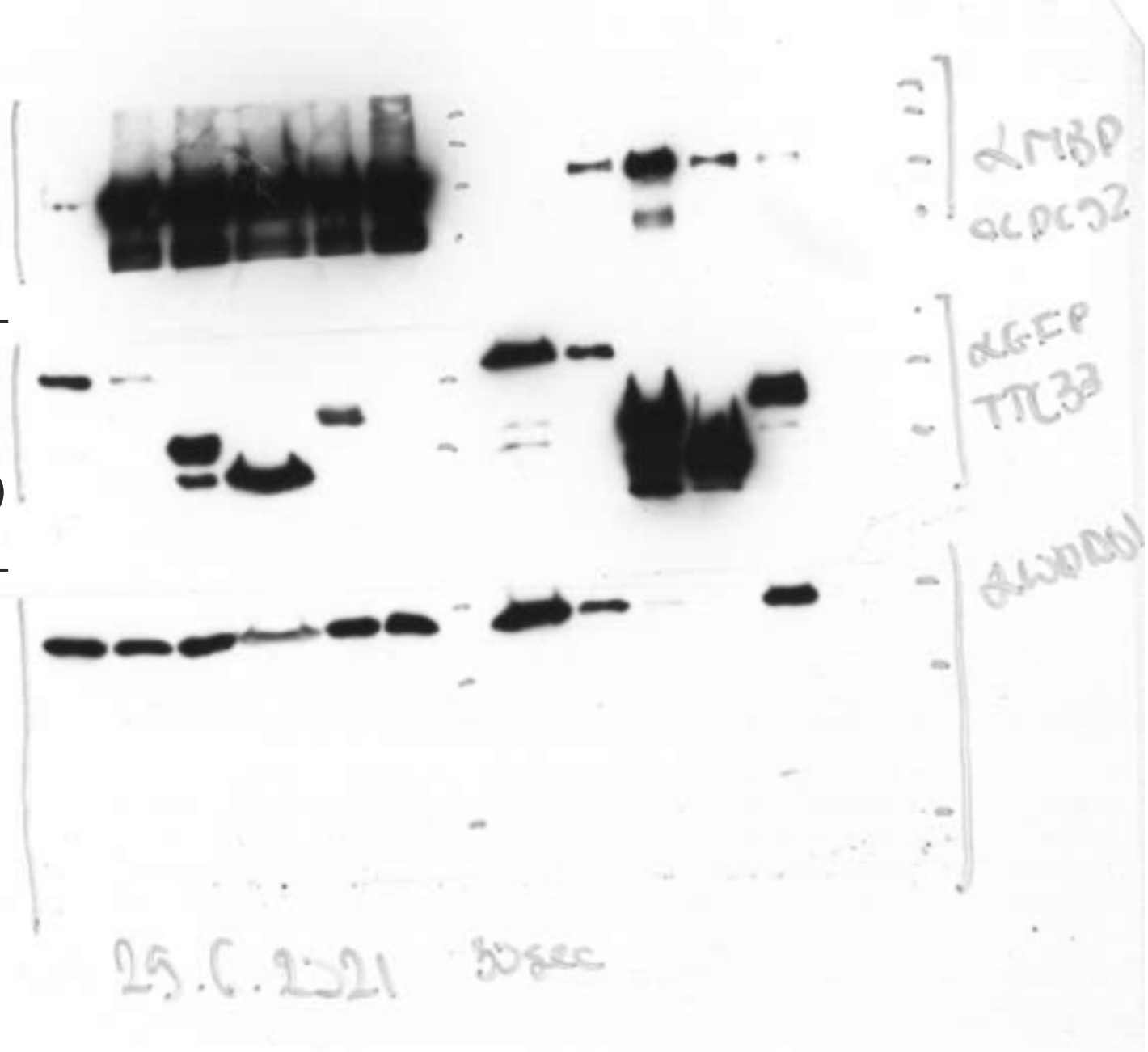

top panel

MBP-PPP2R2A

TTC33-EGFP
(FL or fragments)

WDR61

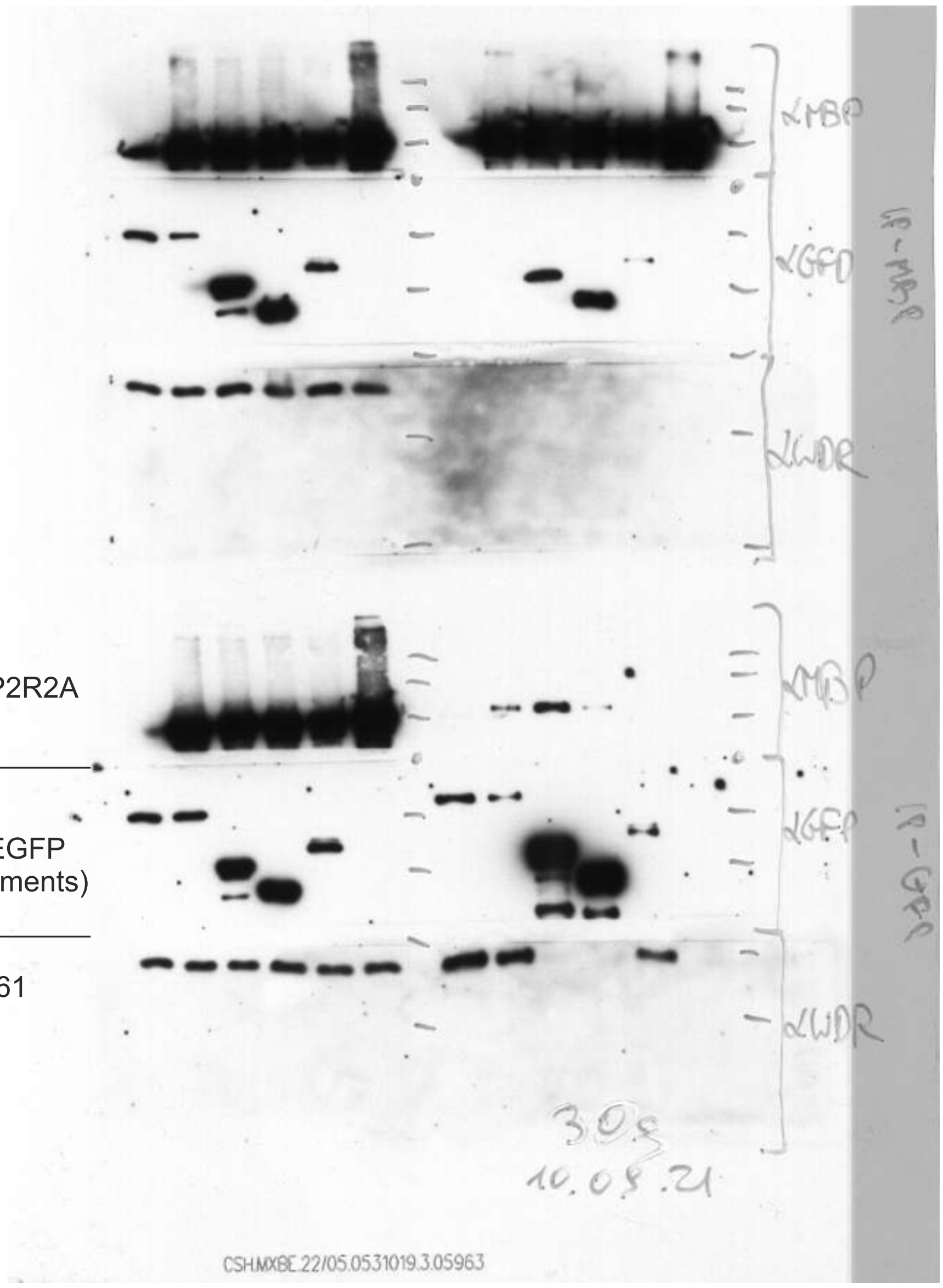

bottom panel

Supplementary Figure 6 c. EGFP-WDR61 WT or W20C as bait test for endogenous CTR9 and SKI2W

western-blot results (uncropped membranes)

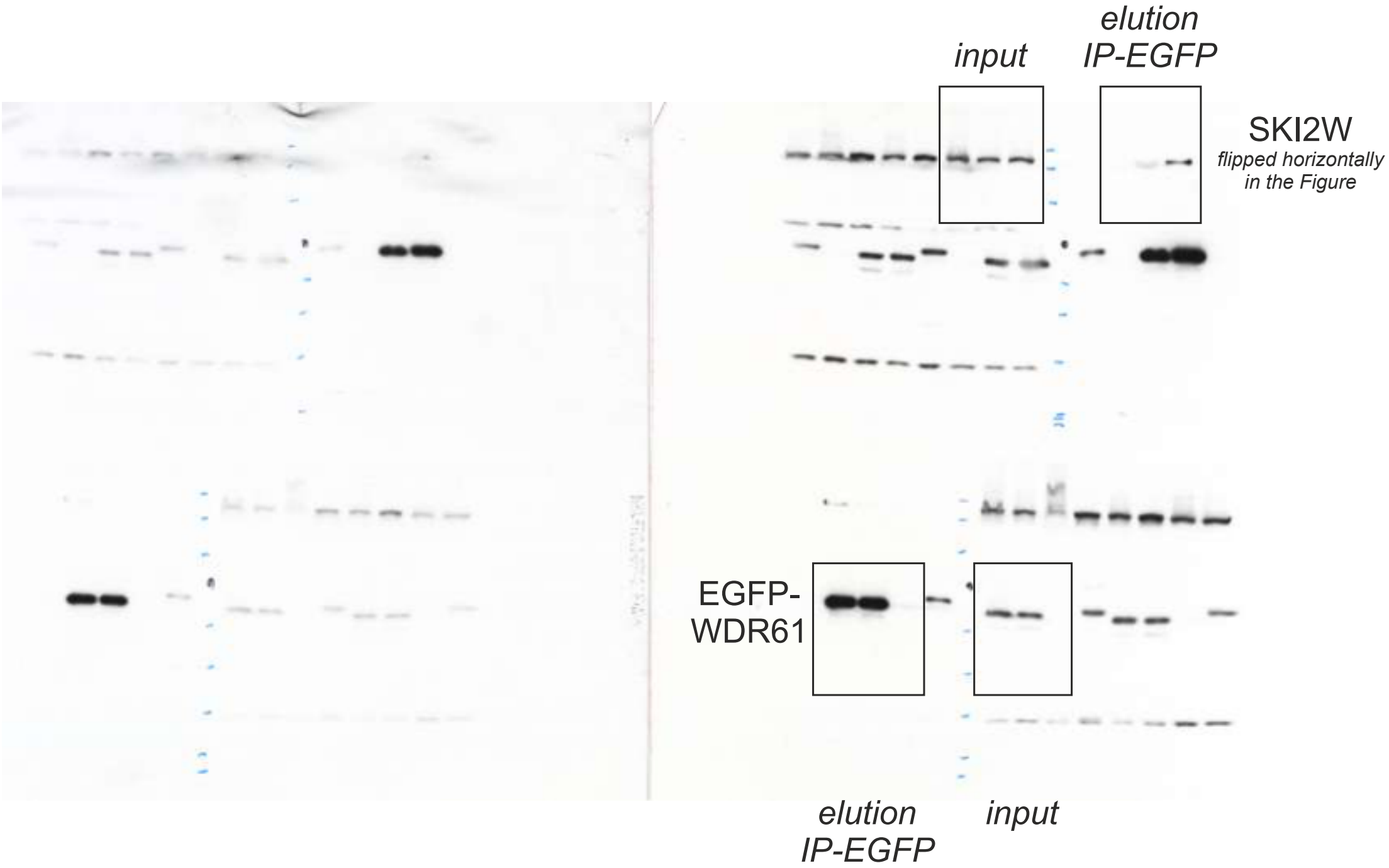

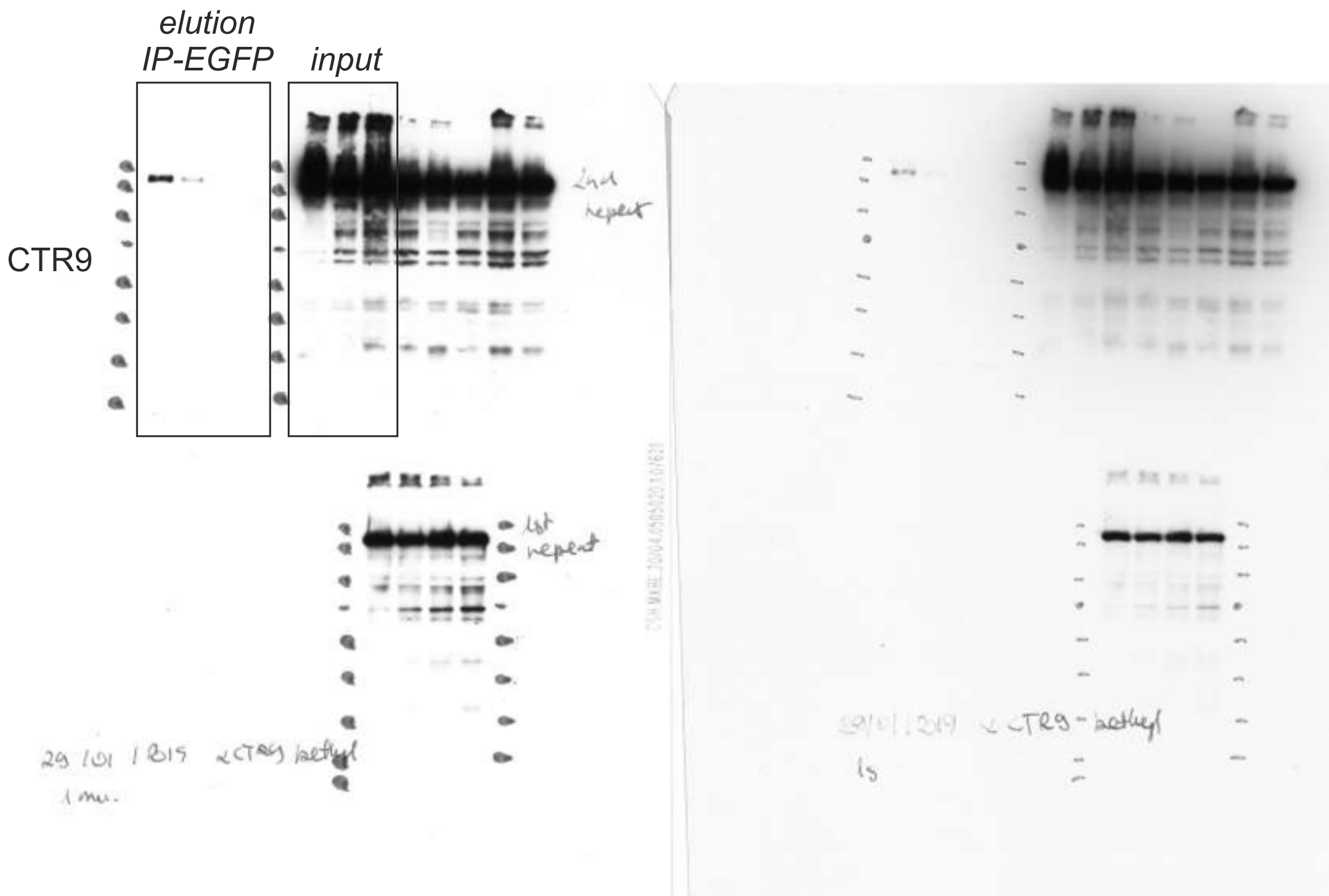

Supplementary Figure 6 d. MBP-TTC33 as bait cotransfected with EGFP-tagged WDR61 (WT or W20C)

western-blot results (uncropped membranes)

MBP-TTC33

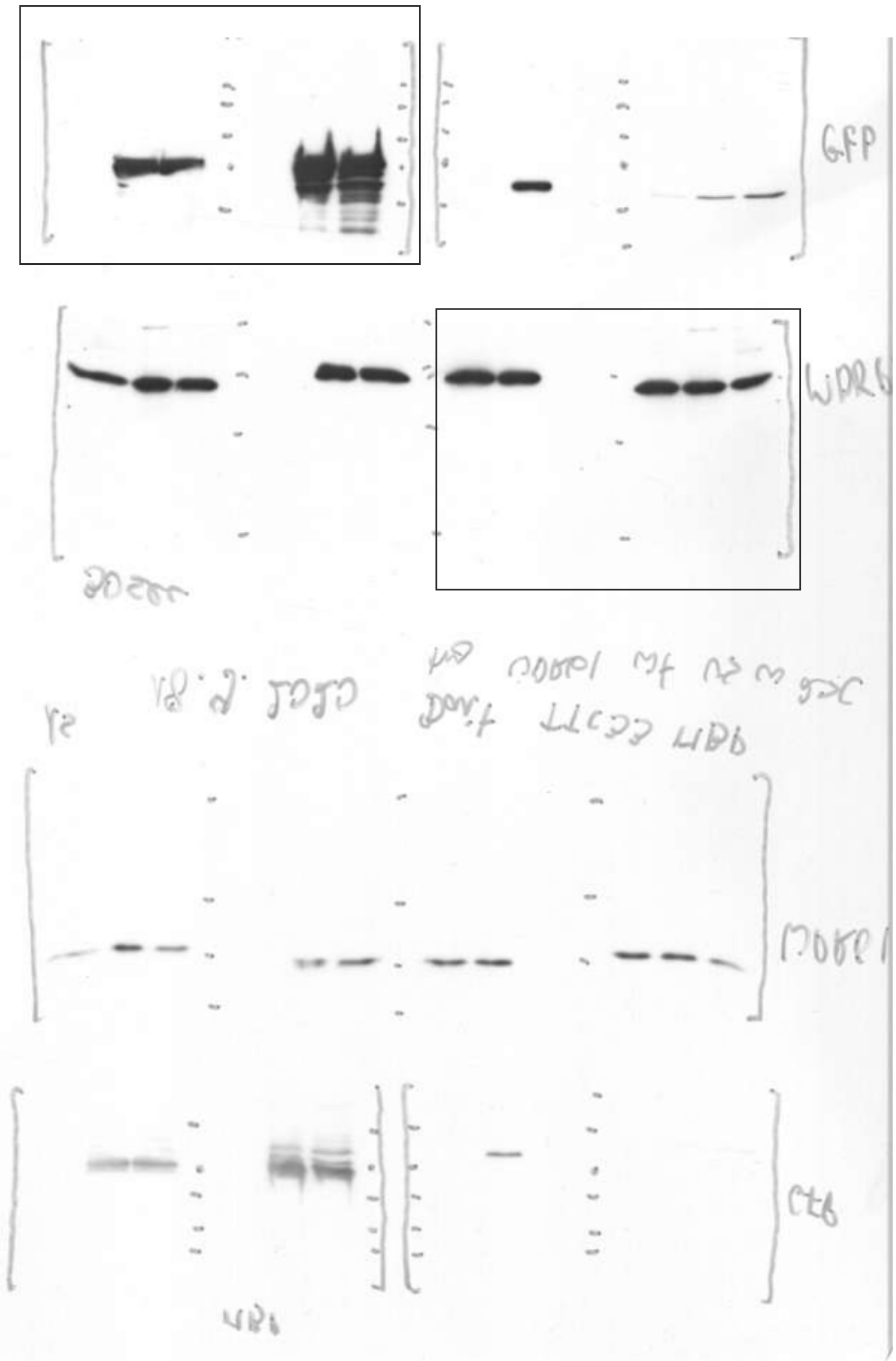

WDR61
flipped horizontally in the Figure

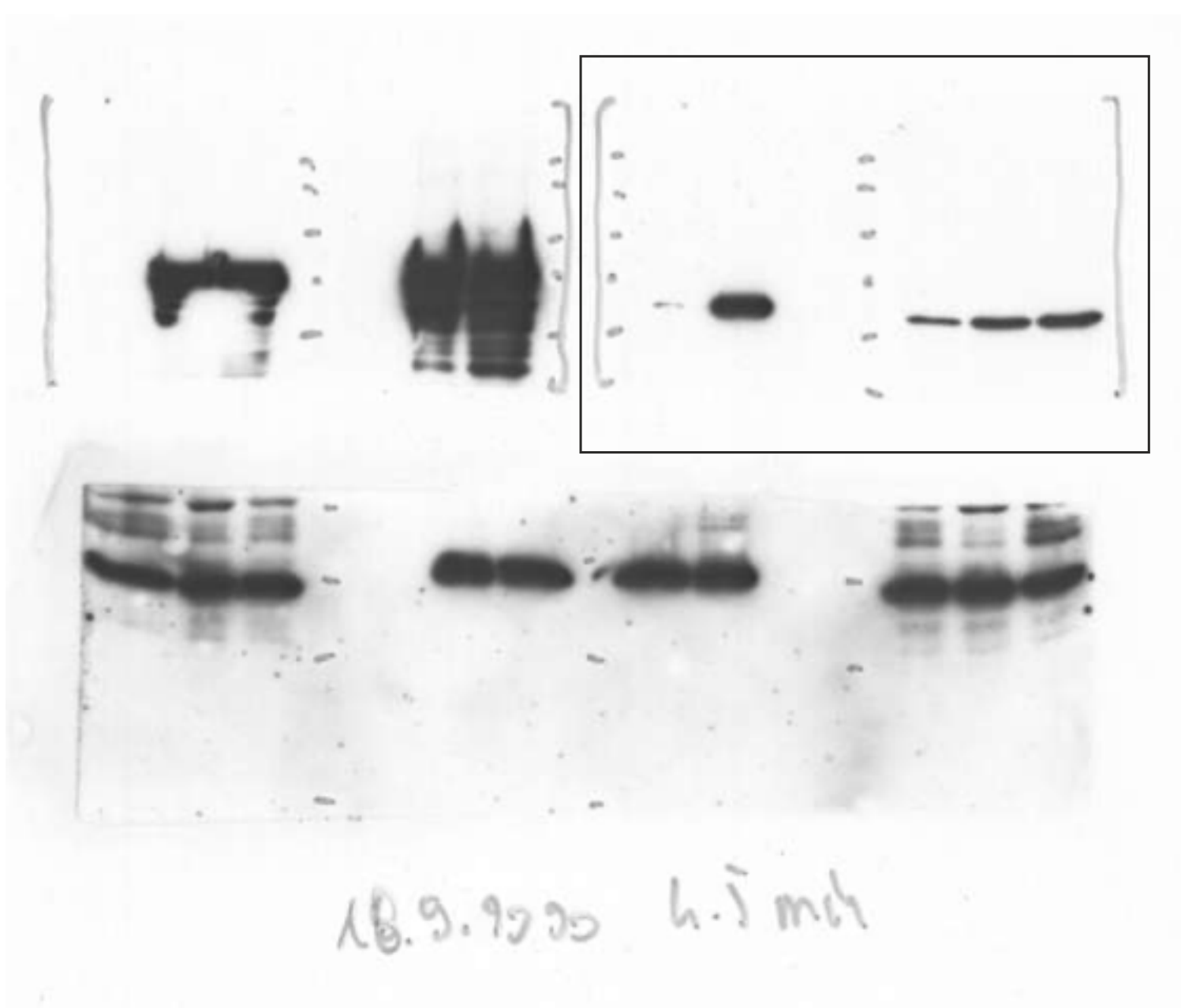

EGFP-WDR61
flipped horizontally in the Figure

Supplementary Figure 6 g. Western blot TTC33 overexpression with MG132

western-blot results (uncropped membranes)

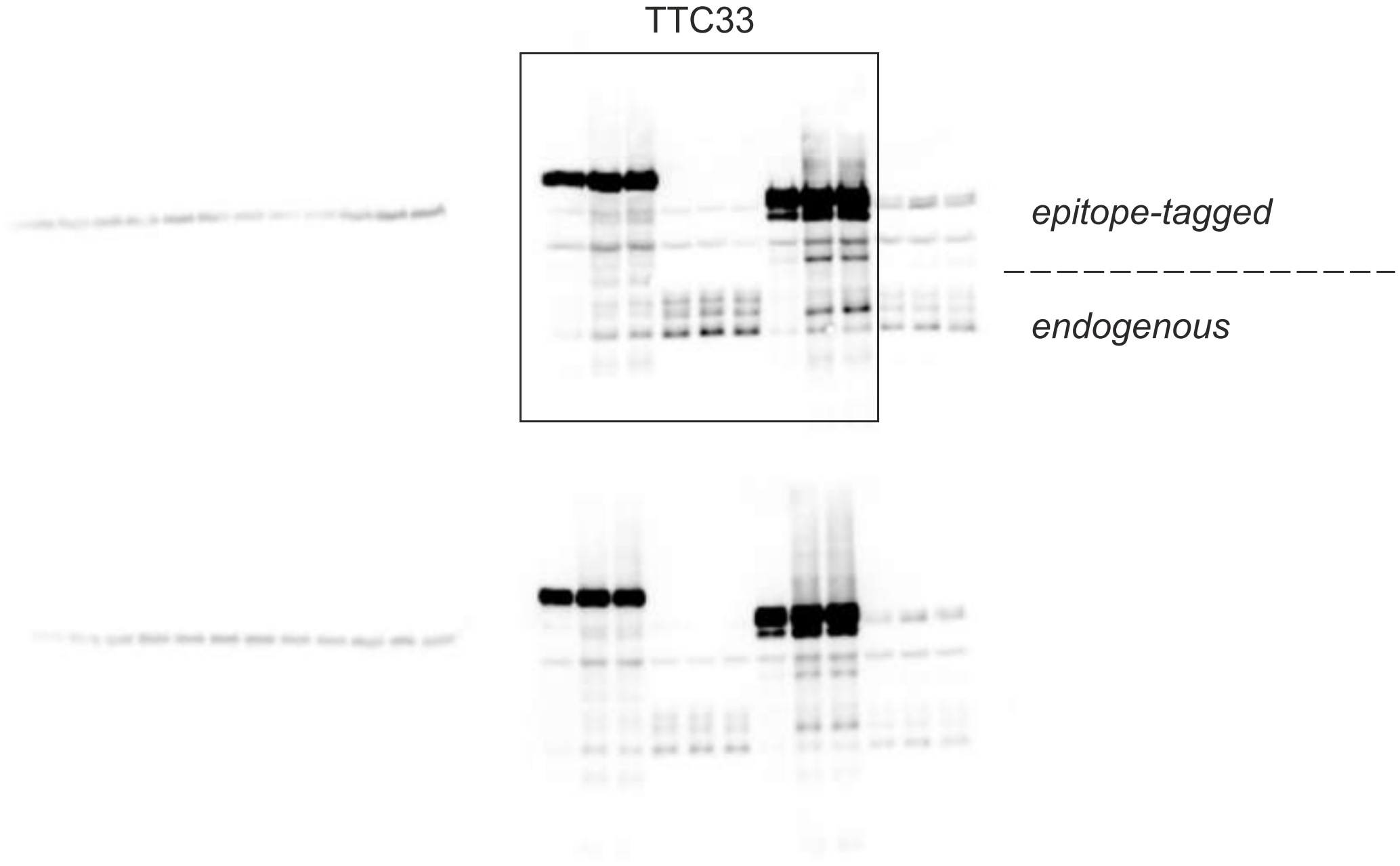

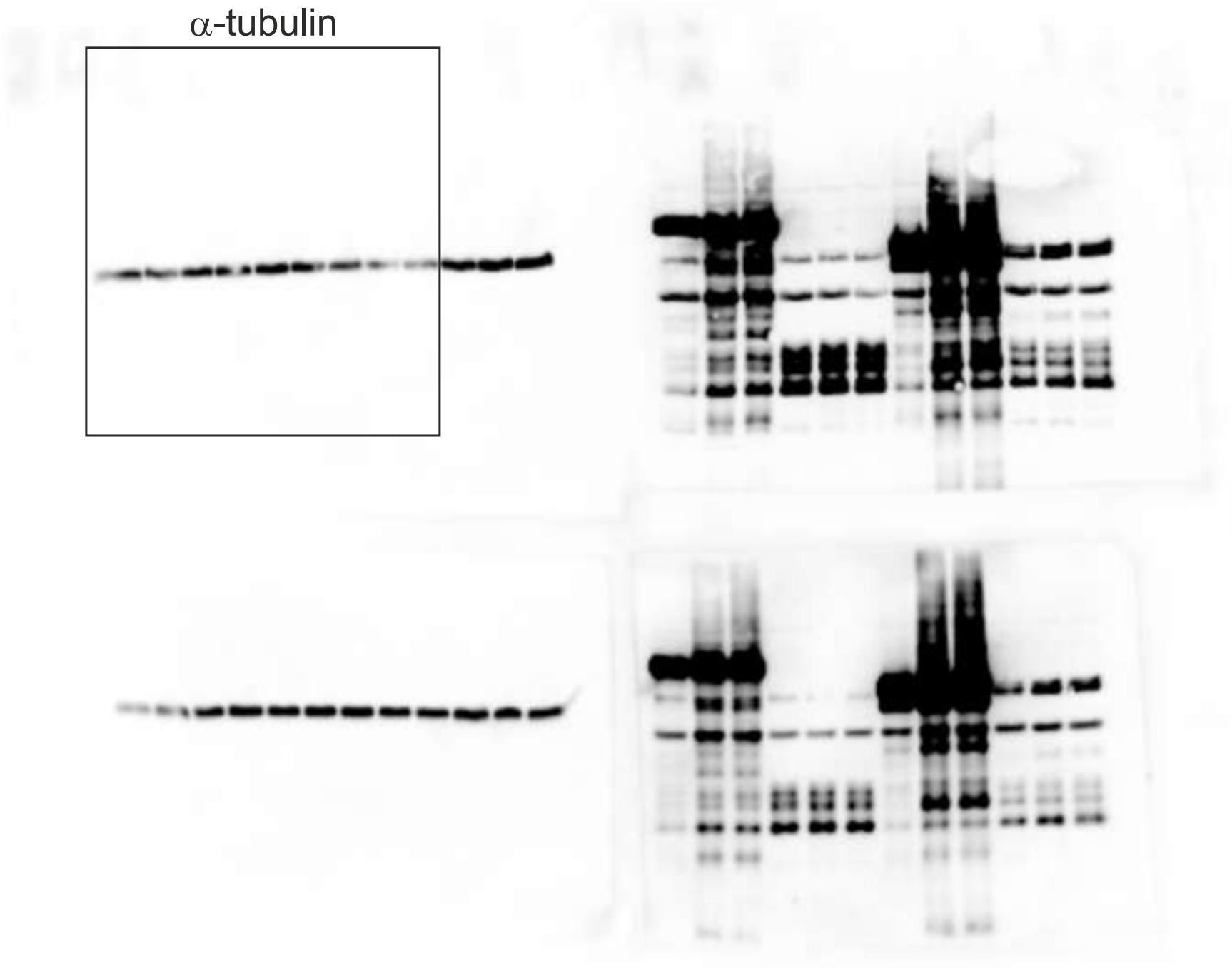

Supplementary Figure 6 h. coIP-WB TTC33-EGFP vs EGFP-TTC33_codon_optimized

western-blot results (uncropped membranes)

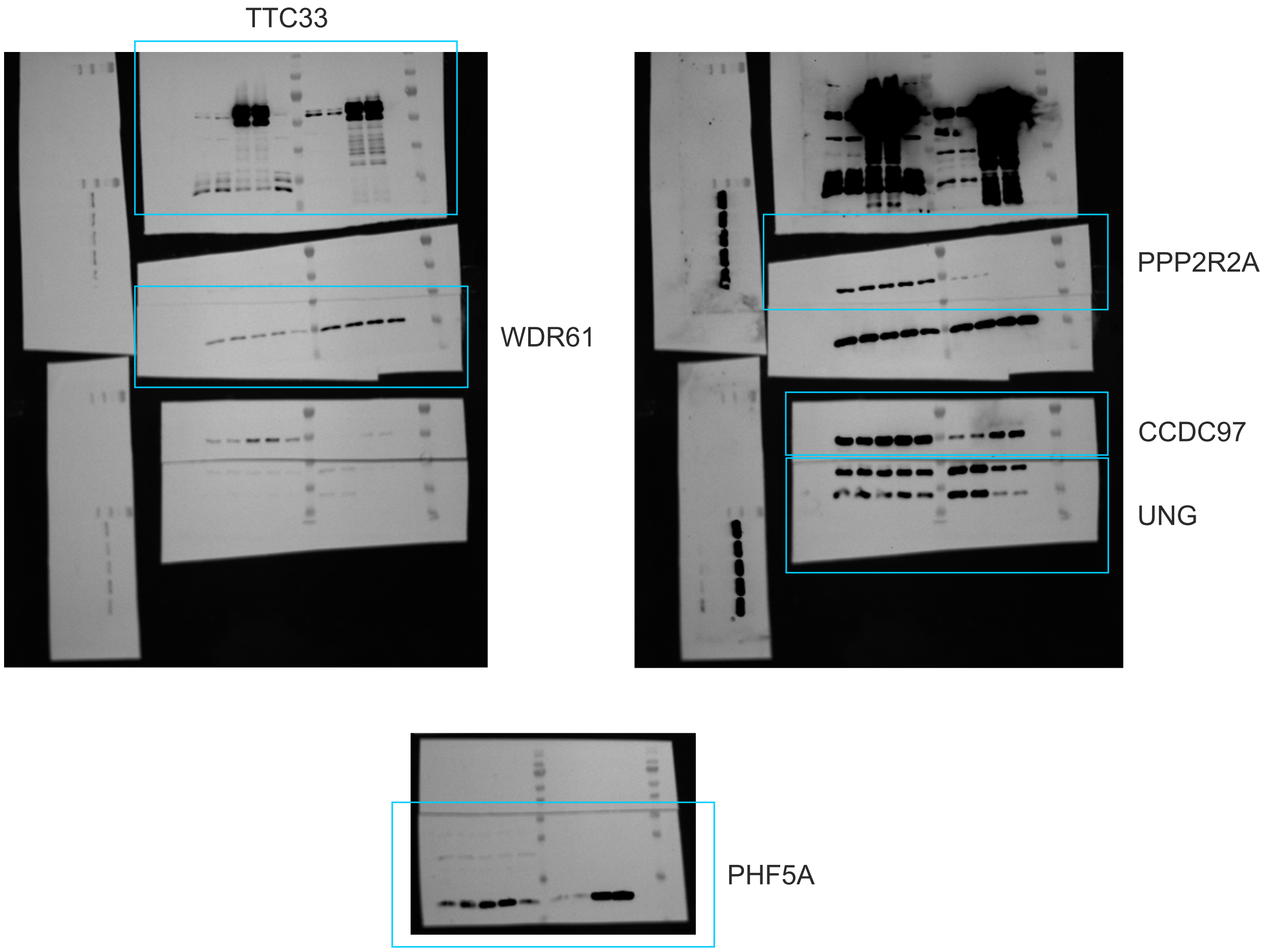

Supplementary Figure 7 a. Validation of PPP2R2A siRNA-mediated silencing

western-blot results (uncropped membranes)

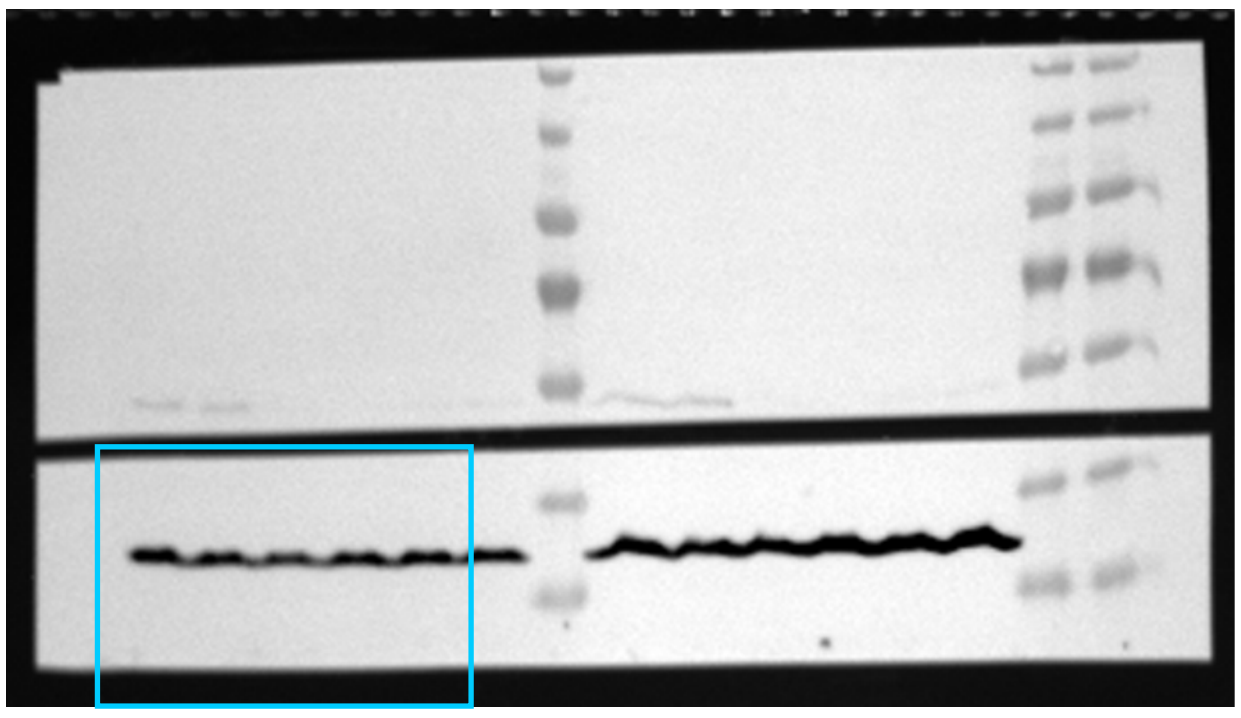

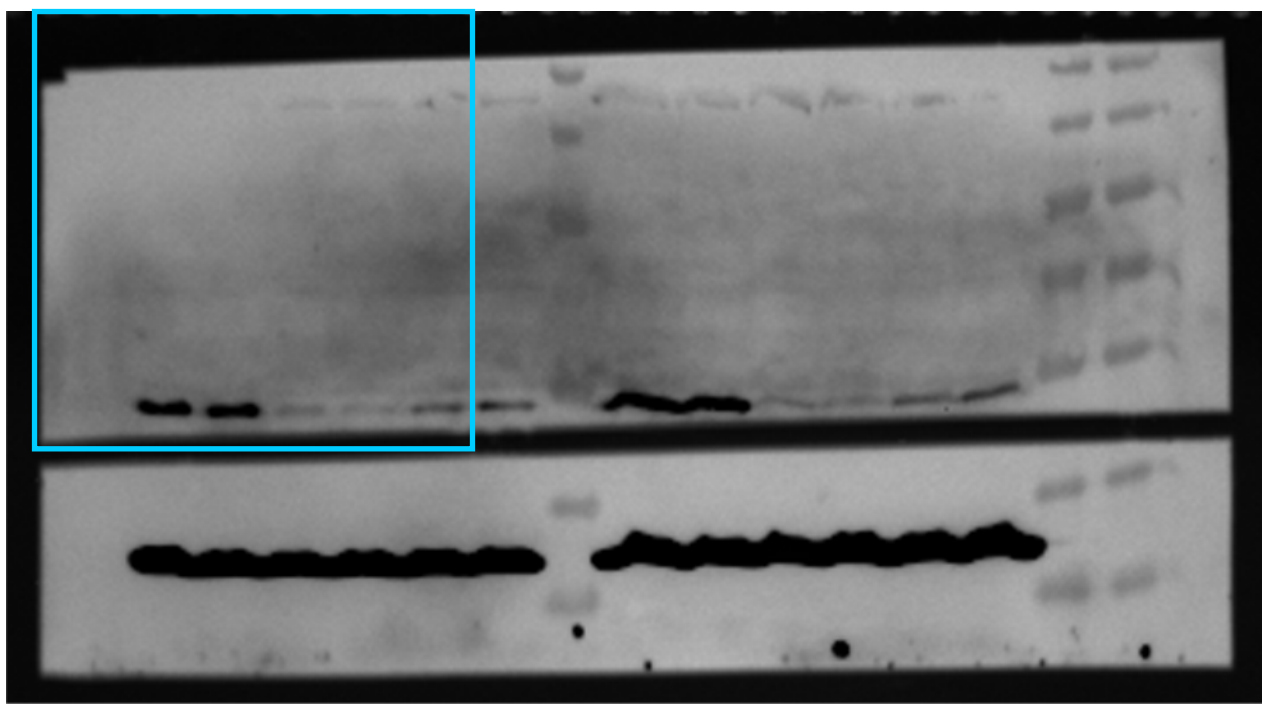

Supplementary Figure 7 c. Validation of TTC33 siRNA-mediated silencing

western-blot results (uncropped membranes)

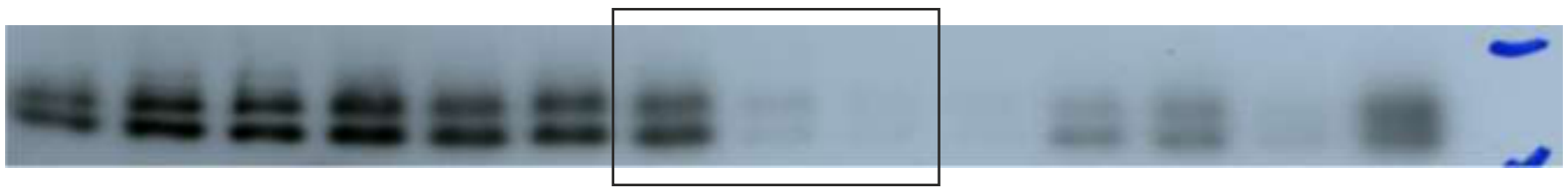

TTC33

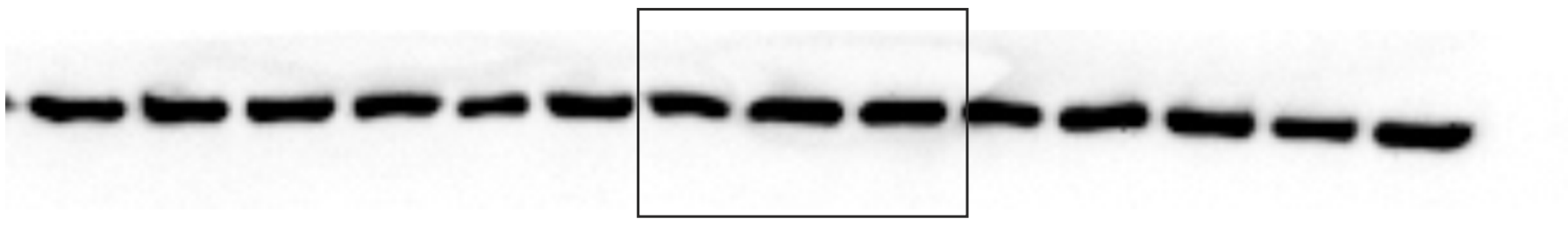

α-tubulin

Supplementary Figure 7 d. Results of *in vitro* UNG activity assays using extracts from 293 Flp-In T-REx stable cell lines with depletion or overexpression of various TAN components

<ins>*upper panel*</ins>

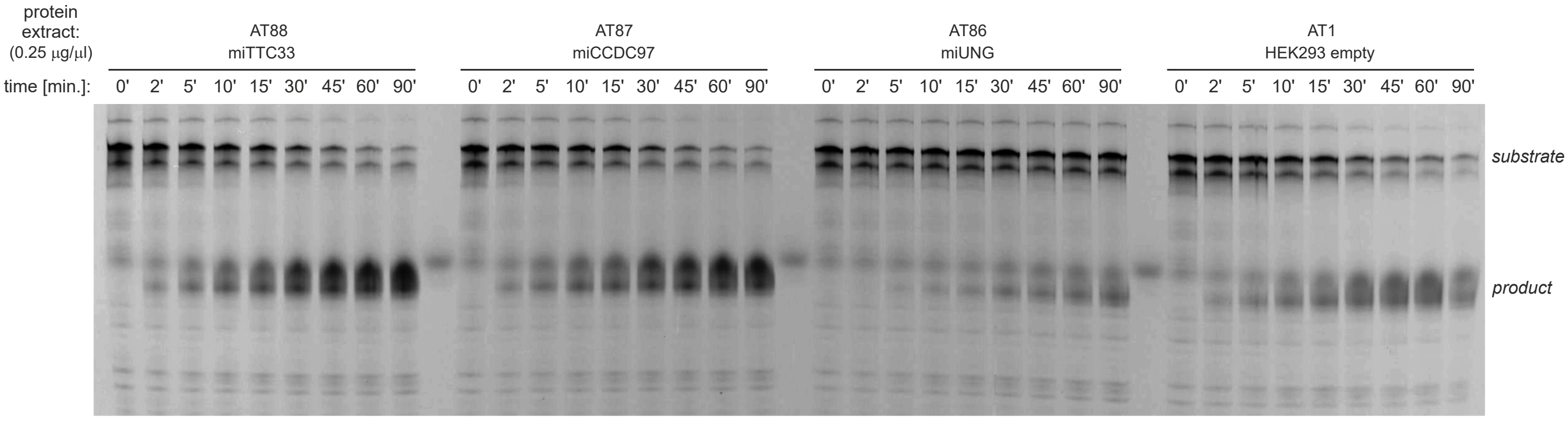

<ins>*lower panel*</ins>

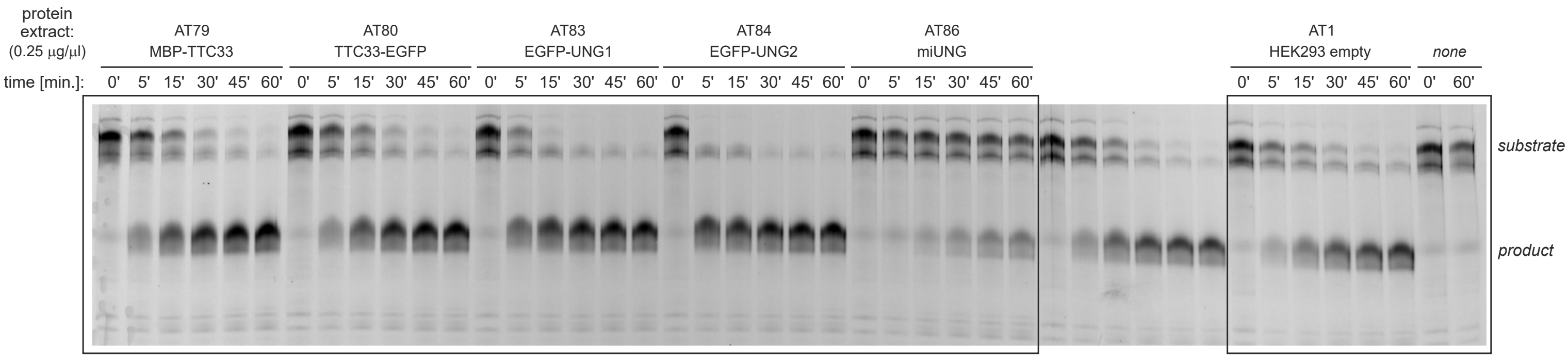

Supplementary References:

1 Karpenahalli, M. R., Lupas, A. N. & Söding, J. TPRpred: A tool for prediction of TPR-, PPR- and SEL1-like repeats from protein sequences. BMC Bioinformatics 8, 2 (2007). https://doi.org/10.1186/1471-2105-8-2

2 Gabler, F. et al. Protein Sequence Analysis Using the MPI Bioinformatics Toolkit. Curr. Protoc. Bioinformatics 72, e108 (2020). https://doi.org/10.1002/cpbi.108

3 Zimmermann, L. et al. A Completely Reimplemented MPI Bioinformatics Toolkit with a New HHpred Server at its Core. J. Mol. Biol. 430, 2237–2243 (2018). https://doi.org/10.1016/j.jmb.2017.12.007

4 Ovcharenko, I., Nobrega, M. A., Loots, G. G. & Stubbs, L. ECR Browser: a tool for visualizing and accessing data from comparisons of multiple vertebrate genomes. Nucleic Acids Res. 32, W280–W286 (2004). https://doi.org/10.1093/nar/gkh355

5 Fagerberg, L. et al. Analysis of the human tissue-specific expression by genome-wide integration of transcriptomics and antibody-based proteomics. Mol. Cell. Proteomics 13, 397–406 (2014). https://doi.org/10.1074/mcp.M113.035600

6 Abramson, J. et al. Accurate structure prediction of biomolecular interactions with AlphaFold 3. Nature 630, 493–500 (2024). https://doi.org/10.1038/s41586-024-07487-w

7 Cretu, C. et al. Molecular Architecture of SF3b and Structural Consequences of Its Cancer-Related Mutations. Mol. Cell 64, 307–319 (2016). https://doi.org/10.1016/j.molcel.2016.08.036

8 Vos, S. M. et al. Structure of activated transcription complex Pol II-DSIF-PAF-SPT6. Nature 560, 607–612 (2018). https://doi.org/10.1038/s41586-018-0440-4

9 Halbach, F., Reichelt, P., Rode, M. & Conti, E. The yeast ski complex: crystal structure and RNA channeling to the exosome complex. Cell 154, 814–826 (2013). https://doi.org/10.1016/j.cell.2013.07.017

10 Kögel, A., Keidel, A., Bonneau, F., Schäfer, I. B. & Conti, E. The human SKI complex regulates channeling of ribosome-bound RNA to the exosome via an intrinsic gatekeeping mechanism. Mol. Cell 82, 756–769.e8 (2022). https://doi.org/10.1016/j.molcel.2022.01.009

11 Liutkus, M., Sasselli, I. R., Rojas, A. L. & Cortajarena, A. L. Diverse crystalline protein scaffolds through metal-dependent polymorphism. *Protein Sci.* **33**, e4971 (2024). https://doi.org/10.1002/pro.4971

12 Liutkus M., Rojas A. L. & Cortajarena A. L. Rational Crystal Contact Engineering for Programmable Self-Assembled Protein Architectures. *Angew. Chem. Int. Ed. Engl.* **64**, e202516635 (2025). https://doi.org/10.1002/anie.202516635

13 Rafie K., Gorelik A., Trapannone R., Borodkin V. S. & van Aalten D. M. F. Thio-Linked UDP-Peptide Conjugates as O-GlcNAc Transferase Inhibitors. *Bioconjug. Chem.* **29**, 1834–1840. (2018). https://doi.org/10.1021/acs.bioconjchem.8b00194

14 Li Z., Hartl F. U. & Bracher A. Structure and function of Hip, an attenuator of the Hsp70 chaperone cycle. *Nat. Struct. Mol. Biol.* **20**, 929–935 (2013). https://doi.org/10.1038/nsmb.2608

15 Wu X. et al. Structural insights into assembly and regulation of the plasma membrane phosphatidylinositol 4-kinase complex. *Dev. Cell* **28**, 19–29 (2014). https://doi.org/10.1016/j.devcel.2013.11.012
