# Supplementary material for: TTC33 forms a complex with WDR61 and PHF5A to recruit factors relevant to genomic integrity
Source: Nat Commun. 2026 Aug 21;17:9984. doi: 10.1038/s41467-026-76928-z (PMC13590505; doi:10.1038/s41467-026-76928-z)
Supplement: Supplementary file 2 [file 41467_2026_76928_MOESM2_ESM.pdf]

Description of Additional Supplementary Files

File Name: Supplementary Data 1
Description: Evolutionary conservation analysis of human TTC33 and CCDC97 proteins, including sequence alignments and a summary of TTC33 population polymorphisms and somatic cancer mutations.

File Name: Supplementary Data 2
Description: Label-free mass spectrometry analysis of TTC33, CCDC97, PPP2R2A, and WDR61 purified proteins.

File Name: Supplementary Data 3
Description: Logistic growth rate parameters and raw cell growth measurements (time and confluence data).

File Name: Supplementary Data 4
Description: Survival analysis of TTC33, CCDC97, WDR61, PHF5A, UNG1/2, and PPP2R2A expression using open-access Human Protein Atlas data.

File Name: Supplementary Data 5
Description: List of oligonucleotides, plasmids, cell lines, and antibodies used in this study.
