# Supplementary material for: TTC33 forms a complex with WDR61 and PHF5A to recruit factors relevant to genomic integrity
Source: Nat Commun. 2026 Aug 21;17:9984. doi: 10.1038/s41467-026-76928-z (PMC13590505; doi:10.1038/s41467-026-76928-z)
Supplement: Supplementary file 3 [file 41467_2026_76928_MOESM8_ESM.pdf]

nature portfolio

Corresponding author(s): Agnieszka Tudek

Last updated by author(s): Jul 10, 2026

Reporting Summary

Nature Portfolio wishes to improve the reproducibility of the work that we publish. This form provides structure for consistency and transparency in reporting. For further information on Nature Portfolio policies, see our Editorial Policies and the Editorial Policy Checklist.

Statistics

For all statistical analyses, confirm that the following items are present in the figure legend, table legend, main text, or Methods section.

n/a	Confirmed	
☐	☒	The exact sample size (*n*) for each experimental group/condition, given as a discrete number and unit of measurement
☐	☒	A statement on whether measurements were taken from distinct samples or whether the same sample was measured repeatedly
☐	☒	The statistical test(s) used AND whether they are one- or two-sided<br>*Only common tests should be described solely by name; describe more complex techniques in the Methods section.*
☐	☒	A description of all covariates tested
☒	☐	A description of any assumptions or corrections, such as tests of normality and adjustment for multiple comparisons
☐	☒	A full description of the statistical parameters including central tendency (e.g. means) or other basic estimates (e.g. regression coefficient) AND variation (e.g. standard deviation) or associated estimates of uncertainty (e.g. confidence intervals)
☐	☒	For null hypothesis testing, the test statistic (e.g. F, t, r) with confidence intervals, effect sizes, degrees of freedom and P value noted<br>*Give P values as exact values whenever suitable.*
☒	☐	For Bayesian analysis, information on the choice of priors and Markov chain Monte Carlo settings
☒	☐	For hierarchical and complex designs, identification of the appropriate level for tests and full reporting of outcomes
☐	☒	Estimates of effect sizes (e.g. Cohen's d, Pearson's r), indicating how they were calculated

Our web collection on statistics for biologists contains articles on many of the points above.

Software and code

Policy information about availability of computer code

Data collection	Fiji/ImageJ (version 2.14.0/1.54f, build c89e8500e4) was used to generate maximum-intensity projections of confocal Z-stack images for fixed-cell and live-cell fluorescence microscopy and to quantify fluorescence signals in microscopy and comet assay images. IncuCyte software (version 2019B Rev2) was used to convert bright-field images into confluence masks and extract raw confluence measurements for cell growth assays. Dedicated ScanR software (Olympus) was used for automated image acquisition and image analysis in BrdU incorporation experiments, including nuclear segmentation and measurement of Hoechst and BrdU fluorescence intensities.
Data analysis	Fiji/ImageJ (version 2.14.0/1.54f, build c89e8500e4) was used for image processing, including nuclear segmentation, background level estimation, fluorescence intensity quantification, and maximum-intensity projections. Statistical analyses and data processing were performed using R and RStudio (versions 1.2.5033 or 2024.04.2+764), including nonlinear least-squares fitting, logistic and Gaussian curve fitting, Spearman correlation, hierarchical clustering, Welch's t-tests, and graph generation with the ggplot2, corrplot, and rstatix packages. Clinical significance analyses were performed using SPSS (version 29 for Windows). Bioinformatics analyses employed the ECR Browser, NLS Mapper, Prosite, TPRpred, Pfam, MultAlin, and NCBI BLAST. Structural modeling and validation were performed using the Swiss-Model Repository, the AlphaFold 3 server, and MolProbity. Molecular dynamics simulations were performed using GROMACS (version 2025.1) via the VisualDynamics interface, and protein structures and simulation trajectories were visualized using PyMOL (version 1.7 Enhanced for Mac OS X or version 3.1.6.1).

For manuscripts utilizing custom algorithms or software that are central to the research but not yet described in published literature, software must be made available to editors and reviewers. We strongly encourage code deposition in a community repository (e.g. GitHub). See the Nature Portfolio guidelines for submitting code & software for further information.

Data

Policy information about availability of data

All manuscripts must include a data availability statement. This statement should provide the following information, where applicable:

- Accession codes, unique identifiers, or web links for publicly available datasets
- A description of any restrictions on data availability
- For clinical datasets or third party data, please ensure that the statement adheres to our policy

Mass spectrometry data are deposited at PRIDE under the accession numbers: PXD059336, PXD059337, and PXD066861. Quantitatively analyzed mass spectrometry data is presented in Supplementary Dataset 2.
Raw data are deposited at Mendeley (doi: 10.17632/p822vhnbdj.1).

Research involving human participants, their data, or biological material

Policy information about studies with human participants or human data. See also policy information about sex, gender (identity/presentation), and sexual orientation and race, ethnicity and racism.

Reporting on sex and gender	Not applicable
Reporting on race, ethnicity, or other socially relevant groupings	Not applicable
Population characteristics	Not applicable
Recruitment	Not applicable
Ethics oversight	Not applicable

Note that full information on the approval of the study protocol must also be provided in the manuscript.

Field-specific reporting

Please select the one below that is the best fit for your research. If you are not sure, read the appropriate sections before making your selection.

[x] Life sciences [ ] Behavioural & social sciences [ ] Ecological, evolutionary & environmental sciences

For a reference copy of the document with all sections, see nature.com/documents/nr-reporting-summary-flat.pdf

Life sciences study design

All studies must disclose on these points even when the disclosure is negative.

Sample size	In our study sample size relates to either the number of biological replicates, or to the number of cells analyzed. This is specified in the figure legend, on the figure panel and in Methods section. For example, the sample size for immunofluerescence assays indicates the number of cells analyzed in each biological replicate. Here a minimum of two and maximum five biological replicates were performed for each relevant condition. In case of proliferative assays, the sample size indicate the number of biological replicates. In each biological replicate the growth rate was determined from confluence data established from several wells each seeded with around 5000 cells.
Data exclusions	Data point were excluded only if it did they did not met the quality criteria. The only excluded data points relate to the proliferation assays, and the criteria were pre-defined consisting of incorrect cell seeding or not sufficient depletion of protein of interest. Only 3 points were excluded out of around 200 points.
Replication	Experiments, whether biochemical, or functional, were performed in minimum two, and maximum five biological replicates.
Randomization	Randomization was not used in our study.
Blinding	Blinding was not used in our study.

Reporting for specific materials, systems and methods

We require information from authors about some types of materials, experimental systems and methods used in many studies. Here, indicate whether each material, system or method listed is relevant to your study. If you are not sure if a list item applies to your research, read the appropriate section before selecting a response.

Materials & experimental systems

n/a	Involved in the study
☐	☒ Antibodies
☐	☒ Eukaryotic cell lines
☒	☐ Palaeontology and archaeology
☒	☐ Animals and other organisms
☒	☐ Clinical data
☒	☐ Dual use research of concern
☒	☐ Plants

Methods

n/a	Involved in the study
☒	☐ ChIP-seq
☒	☐ Flow cytometry
☒	☐ MRI-based neuroimaging

Antibodies

Antibodies used

The list of antibodies used in our study is given in Supplementary Dataset 5:

Antibody name: Host and clonality: Dilution: Assay: Source: Catalog number:
Anti-GFP (B-2) mouse monoclonal 1:10 000 western blot Santa Cruz Biotechnology sc-9996
Anti-GFP rabbit polyclonal 1:3 000 western blot Chromotek pabg1
Anti-MBP mouse monoclonal 1:10 000 western blot New England Biolabs E8032S
Anti-alpha-tubulin (DM1A) mouse monoclonal 1:2 000 western blot Calbiochem CP06
anti-γH2AX rabbit polyclonal 1:350 immunofluorescence Cell Signalling Technology 9718T
Anti-histone H3 rabbit polyclonal 1:10 000 western blot Abcam ab1791
Anti-Phospho-p53 (Ser15) rabbit polyclonal 1:350 immunofluorescence Cell Signaling Technology 9284S
Anti-CCDC97 rabbit polyclonal 1:3 000 western blot Bethyl Laboratories A304-703A
Anti-CTR9 rabbit polyclonal 1:3 000 western blot Bethyl Laboratories A301-395A
Anti-PHF5A rabbit polyclonal 1:3 000 western blot Proteintech 15554-1-AP
Anti-PPP2R2A rabbit polyclonal 1:350 immunofluorescence Biorbyt orb136126
Anti-PPP2R2A rabbit polyclonal 1:2 000 western blot Biorbyt orb136126
Anti-SF3B4 (3A1) mouse monoclonal 1:3 000 western blot Novus Biologicals NBP1-92692SS
Anti-TTC33 rabbit polyclonal 1:3 000 western blot Bethyl Laboratories A304-701A
Anti-SKI2W rabbit polyclonal 1:2 000 western blot Proteintech 11462-1-AP
Anti-WDR61 rabbit polyclonal 1:3 000 western blot Bethyl Laboratories A305-191A
Anti-UNG1/2 rabbit polyclonal 1:3 000 western blot Proteintech 12394-1-AP
Anti-HA mouse monoclonal 1:5 000 western blot Sigma-Aldrich H3663
Anti-rabbit Alexa Fluor® 555 IgG (H+L) goat polyclonal 1:1 000 immunofluorescence Invitrogen A-21429
Anti-rabbit Alexa Fluor® 633 IgG (H+L) goat polyclonal 1:1 000 immunofluorescence Invitrogen A-21070
Anti-rabbit Alexa Fluor® 647 IgG (H+L) goat polyclonal 1:1 000 immunofluorescence Invitrogen A-21240
Anti-Rabbit IgG, H & L Chain Specific Peroxidase Conjugate goat polyclonal 1:10 000 western blot Merck 401393
Anti-Mouse IgG, H&L Chain Specific Peroxidase Conjugate goat polyclonal 1:10 000 western blot Calbiochem 401215
Anti-lamin A/C mouse monoclonal 1:1 000 western blot Santa Cruz Biotechnology sc-7292
anti-BrdU mouse monoclonal 1:400 immunofluorescence Santa Cruz Biotechnology sc-32323

Validation

Protein or species application was verified on the manufacturers website. Additionally, all antibodies directed towards TAN factors (PPP2R2A, WDR61, TTC33, CCDC97, and UNG1/2) were validated using western blot analysis on whole cell extract derived from control and knocked-down cells. Those data are directly shown in the Fig. 8g,h (WDR61), Fig. 9 (TTC33, CCDC97, and UNG1/2) and Fig. S7a (PPP2R2A). For antibodies directed at DNA damage markers, we controled for a change in intensity and subcellular distribution by immunofluorecence in control cells and those treated with doxorubicine, which induces DNA double strand breaks by poisoning the topoisomeras. This is shown in IF microscopy images in Fig. 9, S7, S8.

Eukaryotic cell lines

Policy information about cell lines and Sex and Gender in Research

Cell line source(s)

HeLa Flp-In T-REx (R71407; Thermo Fisher Scientific), HEK293 Flp-In T-REx cells (R78007; Thermo Fisher Scientific), A549 (ATCC CCL-185), and HCT116 (ATCC CCL-247), and FreeStyle™ 293-F suspension cells (R79007; Thermo Fisher Scientific).

Authentication

Commercial or cell bank supplier.

Mycoplasma contamination

Mycoplasma contamination was examined using the MycoSPY® Master Mix, 50 Assays PCR kit for mycoplasma detection (cat. no. M020-05; BIONTEX). Also microscopy DAPI stained pictures were examined for other contaminations.

Commonly misidentified lines
(See ICLAC register)

Not applicable to our study.

Plants

Seed stocks	Not applicable to our study.
Novel plant genotypes	Not applicable to our study.
Authentication	Not applicable to our study.
