# Supplementary material for: TTC33 forms a complex with WDR61 and PHF5A to recruit factors relevant to genomic integrity
Source: Nat Commun. 2026 Aug 21;17:9984. doi: 10.1038/s41467-026-76928-z (PMC13590505; doi:10.1038/s41467-026-76928-z)
Supplement: Supplementary file 4 [file 41467_2026_76928_MOESM9_ESM.pdf]

nature portfolio

Peer Review File

TTC33 forms a complex with WDR61 and PHF5A to recruit factors relevant to genomic integrity

Corresponding Author: Dr Agnieszka Tudek

This file contains all reviewer reports in order by version, followed by all author rebuttals in order by version.

Version 0:

Reviewer comments:

Reviewer #1

(Remarks to the Author)
The manuscript by Drabko et al. unveiled the TTC33-associated network (TAN) of protein interactions contained WDR61, PHF5A, and the SF3B subunits, PP2A-B55α phosphatase (PPP2R2A), UNG DNA glycosylase, and CCDC97 protein by a comparative label-free mass spectrometry. They identified the center of TAN as a trimeric core complex, including TTC33:WDR61:PHF5A through subunit co-purification in stoichiometric levels and co-migration in size exclusion chromatography. Furthermore, they computationally modeled the TANC structure and experimentally validated that WDR61 and PHF5A bind independently to opposite sides of TTC33 TPR4, and the TPR1-3 region is sufficient to recruit other TAN factors. Functionally, the imbalanced expression of TTC33 adversely affects cellular proliferation rates and resistance to genotoxic agents. However, the article lacks detailed evidence explaining the function of each subunit and whether TAN is involved in cell proliferation and resistance to genotoxic agents. Additionally, the absence of in vivo experiments further limits the study's overall impact and significance. Below are several concerns that need to be addressed.

1．The study suggests that TTC33 influences cell proliferation and the DNA damage response. However, whether TTC33 exerts this effect through its interaction with PHF5A and WDR61 or if it regulates the DNA damage response independently is unclear. The current evidence is insufficient, and further investigation is needed to clarify the specific mechanism by which TTC33 functions in cellular processes.
2．The authors mention that TTC33 protein levels are regulated in vivo, but the underlying regulatory mechanisms remain unclear. It is not determined whether TTC33 is controlled at the transcriptional or translational level or if its degradation is mediated by the ubiquitin-proteasome system. Investigating the mechanisms governing TTC33 homeostasis would enhance the study's depth.
3．The characterization of TTC33-related cellular phenotypes lacks specificity. While the findings suggest that TTC33 abundance affects cell proliferation and oxidative stress resistance, no functional rescue experiments have been conducted to validate these effects. Additionally, it remains uncertain whether PHF5A and WDR61 mediate these phenotypic changes. Addressing these points would help strengthen the study's conclusions.
4．Phosphorylation of p53 at serine 15 (Ser15) is used as a marker of DNA damage. However, whether TTC33 also influences other DNA damage markers, such as γ-H2AX is unclear. Additional experimental evidence is needed to support TTC33's role in regulating the DNA damage response further.

5．The study states that TTC33 is evolutionarily conserved in bony vertebrates but does not assess whether its function is preserved across species. Has the structure or function of TTC33 changed over evolution? Have the authors examined its conservation from a genomic or evolutionary perspective? A more detailed discussion on the functional conservation of TTC33 across species would be beneficial.

6．Only one cell line was used in the study, making it unclear whether the complex is commonly found in mammalian cells, which limits the significance of these findings.

7．The biological function of TTC33 in cell proliferation was also assessed in a single cell line, which is insufficient to support its general role in proliferation.

8．The experiments examining TTC33's role in cell proliferation are insufficient. Does it specifically affect certain stages of the cell cycle? Does it influence apoptosis?

9．The biological function of TTC33 has not been well characterized. How it affects DNA damage remains unclear. Additionally, whether TTC33 commonly influences DNA damage in mammalian or cancer cells is unknown.

10．In Figure 1h, TTC33, CCDC97, and PPP2R2A are shown to be independently localized in the nucleus. To demonstrate their co-localization within the nucleus or the subunits of TANC (TTC33, WDR61 and PHF5A), it would be beneficial to show their co-expression within the same cell using immunofluorescence (IF).

11．In this manuscript, the authors utilized co-IP experiments to analyze the association of target proteins under exogenous overexpression conditions. However, it would be important to investigate the endogenous interactions among these components.

12．In Figure 2e, the authors demonstrated that high salt concentrations tend to reduce protein-protein interactions. However, since the proteins appear to have been developed on different films, it is difficult to determine whether the observed differences between low and high salt conditions are due to protein interaction strength or differences in exposure time.

13．In Figure 3i, the authors propose a face-to-face conformation between WDR61 tryptophan 20 and TTC33 histidine 155. However, the discussion later focuses solely on the effect of the WDR61 W20C point mutation without considering TTC33. The impact of the TTC33 mutation should also be addressed.

14．In Figure S3h, it is difficult to accurately assess the binding efficiency due to unequal expression levels in the Input.

15．This manuscript does not demonstrate whether this complex is related to human disease and lacks in vivo experiments to verify its function.

Reviewer #2

(Remarks to the Author)
I co-reviewed this manuscript with one of the reviewers who provided the listed reports. This is part of the Nature Communications initiative to facilitate training in peer review and to provide appropriate recognition for Early Career Researchers who co-review manuscripts.

Reviewer #3

(Remarks to the Author)
In “Comparative mass spectrometry reveals a novel TTC33-associated network core (TANC) complex with functions in DNA Damage response”, Drabko and colleagues investigated the cellular role of the unknown protein TTC33. They first performed a mass-spectrometry based screening for interactors, then validated such interactions with Co-IP, discovering the formation of a complex with WDR61 and PHF5, which was then further explored with in silico modelling followed by Co-IP with point mutations. Other interactors, including with the Uracil-DNA glycosylase UNG1/2 were also further validated as less strong/frequent by co-IP. Based on this latest interaction, the authors focused on the possibility that TTC33 could be involved in DNA damage response and suggest that TTC33 levels could be relevant for genome stability

The cellular function of TTC33 is, as far as this reviewer knows, unknown, so the screening by interaction is interesting as it could potentially elucidate it. However, the strongest interactions found and studied, both in cells and in silico, are with WDR61 and PHF5, both involved in RNA metabolism, but the authors did not comment on this at all. On the contrary, they decided to further characterize the role of TTC33 in DNA damage response due to the finding of a weaker interaction with UNG1/2. However, this interaction is not well characterized (which regions of TTC33 are required? Is always occurring? Does it require WDR61 and PHF5?), and the characterization of the possible role of TTC33 in DNA damage is too preliminary to be conclusive (Does TTC33 depletion increase sensitivity to DNA damaging agents? Does it affect repair of UNG/APX induced single-stranded breaks? Or of DSBs? Or of replication?), significantly reducing the possible impact of this study.

Other points:
1. The validation of the interactions found by LF-MS in Fig.2 and Fig.S1-2-3 is extensive but very confusing. It would be much clearer to show first the validation for the interactions with TTC33 and then to expand the analysis to cross-interactions between the different component of the proposed TAN network and/or the weaker interaction. This is particularly relevant considering that the authors do focus on the TANC.
2. Why does the TANC complex run so much smaller than expected (Fig.2h)? Furthermore, the glycerol gradient (Fig.S2d) and, less, the SEC (Fig.2h) show that CCDC97 co-localize with the TANC (even more than PHF5A), but this is not really addressed in the text.
3. The dissection of the interactions between TTC33 and WDR61/PHF5A in Fig.3 is interesting, but I do not agree with the conclusions. For example, CCDC97 does not interact with C-term of TTC33, but it does with any fragment containing TPR2, so the interaction could be more limited. My suggestion is to add a table for all the tested domain/interaction. Also, have you tested TPR-2 or TPR4 alone?
4. The effect of H_2O_2 on wt or miTTC33 in Fig.3f seems very similar, considering the phenotype of untreated miTTC33-cells, making the point of a role for TTC33 in H_2O_2 response very weak.

Minor points:
1. Show the expression of GFP-TTC33 in cells by immunoblot. The bands shown in S1a-b are not enough. Same for WDR61.
2. Add a marker of nuclear fractionation (LaminA/C?) in Fig.S1g
3. What is the “control” in Fig.1h and i? No ab? No secondary ab? A better control would be after deletion/silencing (as shown in Fig.4). It is very strange that the IF for TTC33 shows such a clear nuclear localization, but for fractionation is more in the cytoplasm. The justification in the text is not consistent with the finding that CCDC97 is more in the nucleus but still it is seen in the cytoplasm.
4. Why are RNase/benzonase increasing the interaction between TTC33 and SF3B4? This doesn’t show that the interaction is weaker as the author say, just that is inhibited (?) by DNA/RNA.
5. The characterization of the TANC complex would benefit from showing more points after C7 in Fig.i (and Fig.S2C)
6. While the prediction of TTC33 structure is probably correct, I don’t think it can be “validated” by predicting other structures of other proteins, as the author claim, especially when for these proteins the structure was already available in 2018, the latest data sets on which AlphaFold was trained. On the contrary, the authors should show the Expected position error for all AlphaFold predictions (TTC3 and the TANC complex)
7. What is the EGFP-TTC33_rec in Fig.S3h? And how is it different from the TTC33-EGFP? In the text is just called “variant with altered TAN factor binding efficiency”, but it is just more expressed. Also, I don’t see how it immunoprecipitated less with WDR61
8. Is a TTC33 mutant in H155 still able to bind WDR61 and/or PHF5A? And which is the exact region of TTC33 interacting with PHF5A? Can it be mutated and recapitulate the lack of interaction with WDR61?
9. Is the PHF5A mutant in Fig.3m affecting the interaction between TTC33 and WDR61? Can it bind still SF3B? Also, please indicate the exact residues that are mutated as well as the exact residues of TTC33 involved in the binding.
10. Provide a quantification for the assay shown in Fig.S4a

Comments to text and figures:
1. The manuscript is overall very difficult to read, going from one finding to the next one without explanation.
2. The introduction is very confusing. I understand that the authors want to introduce all the TTC33-interactors, however, the introduction is not organic, and I think that such introduction could be done when the interactors are described/characterized after isolation. On the contrary, the introduction lacks a general overview of the DNA damage response and its key factors, including p53. A broader introduction on TPR proteins would also benefit the understanding of the work.
3. When something is mentioned first time (i.e. benzonase) it should be introduced.
4. The conclusions are often said before the data (as for the Fig.3l)
5. The alignment of the TTC33 proteins is informative, but the legend is confusing. I also recommend moving the schematic of human TTC33 with all the different TPR regions and the NLS in the first figure.
6. The western blot in Fig.S3a is overexposed and it is difficult to see the levels of the TPR mutants and of CCD97.
7. Where is the full length EGFP-PHF5A in the input of Fig.3m?
8. In Fig.4c the effect of doxorubicin in the IF is not clear (there is less p53-S15P signal than in the untreated cells). Also, I find contradictory that the authors mention the p53-S15P signal being “adjacent to the nucleus” (which means outside the nucleus) and co-localizing “with the centromere” (which are inside the nucleus as part of the chromosomes)

Reviewer #4

(Remarks to the Author)
This is a very interesting work combining computational, mass spectrometry and immunoprecipitation techniques for identifying the interacting partners of a previously uncharacterized protein: TTC33. The study is technically sound and the authors have an excellent experimental design validating their findings using a combination of techniques. There are several information however that are missing from the description of the methods, while the authors could further expand the computational analysis they conducted to infer more information about the functionality of the protein and its potential association with pathophysiological mechanisms.

-"Strikingly, only 11 proteins were common for both lists and only those likely represented bona fide TTC33 partners": The very low overlap of the interacting partners of TTC33 raises concerns for the accuracy of the MS workflow. To further increase confidence of the findings, the authors could try to calculate co-expression of TTC33 and its potential interacting partners in different tissues and conditions (using even publicly available data).
-Are the interacting partners of TTC33 enriched in any pathway or GO term? This will lead to a better understanding of the functionality.
-Aligning the structural models of alphafold and swissmodel would further allow to understand their similarities and differences. Moreover the models should be further evaluated using Ramachandran plots and Molprobity analysis.
-The authors do not provide RMSD values for the alignments.
-The process for selecting templates for homology modeling using swissmodel was not explained anywhere in the manuscript.
-The revealed protein complex should be also verified and analyzed with docking methods (such as using the alphafold-multimer tool).
-Molecular dynamics simulations are missing for both the TTC33 as well as the studied protein complex.
-"The MaxQuant protein intensity data was further analyzed using Rstudio (Version 1.2.5033). ": A github link for the analysis would further increase its transparency.

-"To balance samples in each experiment, the data was normalized. ": What type of normalization was applied? Total-ion-intensity based or something else?
-"The missing values were replaced by randomized data,which was at least one order of magnitude lower than the weakest hit in each sample.": This is ok but it should be coupled with a filtering approach to filter out proteins with too many missing values.
-Despite the importance of the worok the authors do not emphasize on the impact of identifying the interacting partners of TTC33? Are there dbSNP or GWAS hits associating it with any disease?

Version 1:

Reviewer comments:

Reviewer #1

(Remarks to the Author)
The authors have sufficiently addressed the questions raised by the reviewers.

Reviewer #2

(Remarks to the Author)
I co-reviewed this manuscript with one of the reviewers who provided the listed reports. This is part of the Nature Communications initiative to facilitate training in peer review and to provide appropriate recognition for Early Career Researchers who co-review manuscripts.

Reviewer #3

(Remarks to the Author)
In the revised manuscript, Tudek and colleagues addressed most of my comments adding numerous new/repeated experiments, defining in details the interaction between TTC33 and WDR61/PHF5, as well as their role in preserving genome stability. They also reorganize the data improving clarity.

I still have a concern on the identification of TTC33/WDR61/PHF5 as involved in genome integrity “surveillance” (line 40) or “DNA repair” (line 374, line 443). The extensive data provided by the authors hints to a role for TTC33 in genome integrity, but more in maintenance-preventing damage than repair after damage. For example, the comet assay detects increased breaks after TTC33 depletion, but the depletion of many repair factors does not really increase the comet, unless cells are treated with DNA damaging agents before. Data on cancer do not really correlate with DNA damage neither. Furthermore, hydrogen peroxide cause general cell damage, not only at the DNA, and the delay in recovery could be due to many different issues. A DSBs induced drug as Neocarzinostatin or IR radiation would be more appropriate to test DNA damage response/repair. Importantly, reduction in growth is not observed in UNG1/2 knock down, although there is an increased in p53 phosphorylation, while reduced growth is observed only when UNG1/2 is overexpressed, weakening the suggested similarities between TTC33 and UNG1/2 in repair (lines 437-439 and lines 449-453). Finally, a reduction in growth is also observed after TTC33 overexpression not only after TTC33 depletion. Does also overexpression increase H2AX and/or p53 foci? And how the authors interpret these phenotypes? While I agree with the authors that the work is mainly biochemical, and it is relevant for the identification of this novel complex, while further work should be addressing how TTC33 could participate in genome integrity maintenance, I strongly recommend toning down some conclusions and maintain a general “genome instability” prospective instead than DNA repair, keeping the discussion more open, also on the indirect role and the discrepancies.

Minor comments:
1. There are many proteins involved and it is not easy to follow all of their known functions and interactions, what was known before and what is now new. I recommend to add protein-protein interacting network (at least in the supplementary) to define the different complexes-before and after the study.
Did the coIP-LF-MS analysis of PP2RA identify also TTC33?
Did the coIP-LF-MS analysis of WDR61 identify also PP2RA?

2. I appreciate the introduction on genome stability, but there should also be a short introduction on what is known on TTC33 functon(not only structure).
3. I still do not agree on the conclusion that since “TTC33 and its interactors reside in common sub-cellular compartment, i.e. the nucleus”, “TAN might be biologically meaningful in various cell types” (Lines 157-160). The localization of both TTC33 and CCD97 is both cytoplasmic and nuclear (according to fractionation, TTC33 is almost more cytoplasmic, and according to imaging mostly nuclear, while CCD97 is still mostly nuclear but clearly also cytoplasmic with both assay), with no clear foci formation, so the conclusion would be valid for any pan-cellular protein. The conclusion should better reflect the significance of these data.

Comments to text and figures:
line 59: natural replication does not induce DSBs (as meiosis and CS recombination)
line 63: there is also alternative End Joining (alt-EJ), even more prone to errors than NHEJ.
line 67: “MDC1:53BP1:BRCA1:MRN foci” is an over simplification and is not further addressed in the manuscript, I suggest a simpler “repair foci”.

Lines 75-78: the explanation of cancer tissue-specificity is over simplistic. And in this case, also not completely correct, For example, familiar mutations in BER genes do not strongly correlate with liver or kidney cancers
Lines 135-136: I may be wrong, but the interaction between TTC33 and SF3B4 in HeLa cells is not validated by the Co-Ip shown in Figure 2d.
Line 138: the definition of SKI and PAF should be added.
Line 141: the definition of SF3A/B should be added.
Lines 154-155: it is not clear how Supplementary Figure 2a shows the nuclear functions of WDR61, SF3A/B, PP2A-B55alpha and UNG.
Line 247: the reduction in interaction with SF3B4 seem higher than “slightly”.
Line 257: I think it should be “TPR4 alone” and not “TPR alone”.
Lines 320-321: add how varied.
Figure 1: is the human there? And would it be possible to have the name order in each panel?
Figure 6 d-e: use the same color code.
Figure 7d and Supplementary Figure 5a: use another color to indicate the C-ter of PHF5 that is important for the interaction with TTC33. It is difficult to see where it is. The legend is not indicating what is shown in gray and orange
Figure 7e. add the name of the first and last aa, for both peptides
Figure 7f: is it possible to highlight in different colors the residues tested in the Co-Ip and their impact on the interaction?
Figure 7g. add a schematic of the mutant fragments
Figure 7h: add if the mutations is a rare variant or a cancer variant
Figure 8: red over orange is very difficult to see
Figure 9c: use the same order for the images and the graph-and also for Figure 9d
Figure 9e: do the author have an immunoblot for the complementation?

Reviewer #4

(Remarks to the Author)
This manuscript attempted to characterize and identify the interacting partners of a previously uncharacterized protein: TTC33. The manuscript had a very strong experimental design initially but lacked some method details, validation and elaborate computational analysis. During the revision, the authors substantially improved the documentation and presentation of the methods and results respectively. Moreover, the bioinformatics work now is significantly improved allowing for a better and more in depth study and characterization of the interaction sites of TTC33, more justified hypotheses and conclusions about its functionality and better documented characterization its association with diseases. The authors also improved the overall impact of their study by adding the experiments, data and analysis required for a better validation of the TTC33-associated interaction network and by performing further functional analysis of TTC33 and its partners.
I believe that there are now only minor missing parts in the manuscript summarized in the following two points:
-Regarding performing coexpression analysis between TTC33 and its partners in various tissues, the authors identified relevant dataset but instead of performing a correlation analysis (eg calculating Spearman’s Rho correlation between the potentially interacting pairs), they just confirmed the expression levels of TTC33 and the potentially interaction partners. Co-expression analysis might reveal potentially common regulatory patterns which are usually present in physically interacting proteins and performing this analysis would further improve the manuscript.
-The process for selecting templates for homology modelling through the Swissmodel automatic option is now selected. But to reassure that there des not exist any more relevant template the authors should provide a table of the potential templates that swissmodel reports including metrics such as coverage, identify, experimental method of template structure and oligomeric state.

Version 2:

Reviewer comments:

Reviewer #3

(Remarks to the Author)
The authors have addressed all my concerns, and I support the publication in the current form.
I feel there are only few minor points, that they may consider in the final version of the manuscript:
1- Line 64: DNA lesions “as Double Strand Breaks (DSBs) or single strand DNA” activate… as not all DNA lesions lead to PIKK kinase activation
2- Figure 2c-d: could the authors use the same order for both figures? It is difficult to follow where is what otherwise.
3- I still think that not all interactions listed in the TAN list (Figure 2c) are “positively validated” in Figure 2d, for example the ones with PP2A-B55alpha and SF3A/B. The authors should tone this down as “mostly validated”
4- Line 164: “likewise detected in the nucleus” is misleading, as TTC33 and CCDC97 are also in the cytoplasm. The correct sentence would be “likewise detected in the nucleus and cytoplasm”.
5- Line 389: I agree with the possible adaptation in cells O/E TTC33, as suggested by the authors in the rebuttal. I would add this hypothesis also in the text to better explain the shown phenotype.

Open Access This Peer Review File is licensed under a Creative Commons Attribution 4.0 International License, which permits use, sharing, adaptation, distribution and reproduction in any medium or format, as long as you give appropriate credit to the original author(s) and the source, provide a link to the Creative Commons license, and indicate if changes were made.
In cases where reviewers are anonymous, credit should be given to 'Anonymous Referee' and the source.
The images or other third party material in this Peer Review File are included in the article's Creative Commons license, unless indicated otherwise in a credit line to the material. If material is not included in the article's Creative Commons license and your intended use is not permitted by statutory regulation or exceeds the permitted use, you will need to obtain permission directly from the copyright holder.
To view a copy of this license, visit https://creativecommons.org/licenses/by/4.0/

POINT-BY-POINT RESPONSE

***

TTC33 forms a complex with WDR61 and PHF5A to recruit factors relevant to genomic integrity

***

TABLE OF CONTENTS

PREAMBLE..page 2

Table 1 (data: original vs. revised).........page 3

RESPONSE TO REVIEWER 1.................................page 13

RESPONSE TO REVIEWER 2.................................page 24

RESPONSE TO REVIEWER 3.................................page 25

RESPONSE TO REVIEWER 4.................................page 37

PREAMBLE

Following the Reviewers' comments, the manuscript underwent a major revision. **We are grateful for the input we received, as it has significantly increased the quality of our work.** As a consequence of numerous remarks, the text of the Introduction, Results, and Discussion has been extensively re-written, and novel data have been added. **The stylistic changes to the paper are marked in blue**, **whereas descriptions of new bioinformatics analyses and experimental data are highlighted in green**. Below we include the ***Table 1***, which sums up all the data presented in the paper, marking whether it was provided in the original (first) or revised version. **Since multiple changes were requested by various Reviewers, below we list the improvements, grouped thematically:**

Evolutionary conservation of the TTC33 and CCDC97 proteins:

1. Analysis of TTC33 and CCDC97 genomic loci conservation across vertebrate species (conclusion: loci conserved);
2. Analysis of TTC33 and CCDC97 protein sequence conservation using 300 vertebrate homologs;
3. Analysis of tetratricopeptide (TPR) repeat conservation in 12 vertebrate TTC33 proteins using TPRpred, along with identification of nuclear localization signals in those species (3 central TPR repeats consistently identified, NLS in N-terminus is common);
4. Analysis of TTC33 TPR domain structural conservation in 12 vertebrate species using AlphaFold 3 (TPR domain fold is conserved).

Validation of the TTC33-associated interaction network and assessment of its conservation across human cell lines:

1. High-throughput proteomic analysis of TTC33-EGFP interactors in HeLa cells, and analysis of MBP-TTC33 interactors purified from HEK293. Those two novel datasets expand previously obtained TTC33-EGFP data from HEK293 and FreeStyle-293-F (the stable TTC33 interactors list is unchanged regardless of cell line types and tags used for purifications);
2. Unified and clearly presented cross-comparison of 4 high-throughput mass spectrometry datasets of TTC33 interactors (from point 1; improved mass spectrometry datasets confirm stable TTC33 interactors, but also provide a scored list of putative weaker partners);
3. Improved coIP-WB validation of TTC33 interactors in HEK293 and HeLa cell lines using MBP tag;
4. Validation of MBP-CCDC97 interactor list obtained from HEK293 in HeLa cells by coIP-WB (which suggest that CCDC97 associates with TTC33, WDR61, PHF5A-SF3B, but not with UNG1/2);
5. Analysis of UNG1 and UNG2 isoform interactions using coIP-WB, as a cross-confirmation of analyses in point 4 (confirmed UNG1/2 association with TTC33, WDR61, PHF5A, but showed lack of association with CCDC97 and SF3B4. Considered with point 4, this shows that TTC33, WDR61, and PHF5A engage in mutually exclusive interactions with UNG1/2 or CCDC97 and SF3B);
6. Validation of the AlphaFold 3 structural model in both HEK293 and HeLa cell lines by either deletions or point mutations of TTC33 or WDR61, including somatic cancer mutations or population variants (shows detrimental effects of some variants);
7. Analysis of the available RNA-seq data showing ubiquitous expression of TTC33 and its protein partners in various human tissues.

Structural modeling of TTC33:WDR61:PHF5A complex (TANC) and model validation:

1. Structural alignment of AlphaFold 3 and SwissModel proposed for TTC33 core TPR domain (RMSD ~1.7);
2. Structural alignments of the human TTC33 TPR core domain to models of 12 vertebrate homologues (RMSD ~0.3);
3. Inclusion of position error plot, pTM (0.7), and ipTM (0.79) values for the AlphaFold 3 TANC model. Alignments of 5 AlphaFold 3 models proposed for TANC;

4. *In silico* validation of the AlphaFold 3 TANC model using the MolProbity suite, including Ramachandran analysis;
5. Molecular dynamics of TANC structural model, with detailed mapping of the evolution of the subunit interfaces;
6. Molecular docking of the PHF5A C-terminus into the TTC33 TPR domain;
7. Mutational analysis of TTC33. Mutations were designed based on the AlphaFold 3 model, its evolution during molecular dynamics, and evolutionary sequence conservation (as based on 300 vertebrate sequences). Whenever possible, population variants or somatic cancer mutations were used.

Functional analysis of TTC33 and its partners role in genomic integrity surveillance:

1. Role of WDR61 as a factor protecting TTC33 from proteolytic decay;
2. Assessment of γH2AX (DNA double-strand break marker) nuclear accumulation in:

a. cells knocked-down for TTC33 or CCDC97 using microRNAs, and CRISPR-Cas9 knock-out cell lines, and cells knocked-down for TTC33 using siRNAs;
b. PPP2R2A knock-down cell lines;
c. Functional rescue of γH2AX levels in cells depleted of WDR61 using microRNA, obtained by expression of a wild-type WDR61 insensitive to microRNA, but not with a W20C mutant incapable of binding to TTC33.

3. Assessment of proliferation rates of cancer cell lines A549, HeLa, and HCT116 under siRNA-mediated TTC33 knock-down, as a comparison to similar assays performed in HEK293 using microRNAs;
4. p53-S15P nuclear accumulation in A549 cells and validation of the phenotype in HEK293 cells using siRNAs;
5. Calculation of the number of cells in G1/S/G2 cell cycle phases for HEK293 control and cells knocked-down for TTC33 or CCDC97 (accumulation of G2 cells, and decrease of G1 in TTC33/CCDC97 knock-downs);
6. Analysis of BrdU incorporation in TTC33 or CCDC97 knocked-down cells;
7. Assessment of proliferation rates of UNG1/2 knock-down and UNG2 over-expressing cells and their sensitivity to hydrogen peroxide (ROS). Re-calculation of old datasets for TTC33 and CCDC97 knock-downs to put forward the ROS-sensitivity phenotype;
8. Analysis of TCGA clinical data showing TTC33 mRNA decrease in clear cell kidney adenocarcinoma (KIRC). Survival assessment shows roughly a 2-fold increase in survival chances in patients with higher expression of TTC33 and its interactors.

Regarding the functional analysis, the Reviewer 3 asked for the exploration of a putative role of TTC33 in RNA metabolism. We performed RNA-seq experiments to address this question, but consider the results as largely negative. We briefly discuss the obtained RNA-seq data in the present document, but refrain from including them in the revision.

The Table 1 below lists all Figure panels and presents the scientific aim, conclusion, and whether data were presented in the original manuscript or included during revision.

Table 1. List of experiments and bioinformatic analyses presented in the manuscript, with references to appropriate Figure panels, including specific aim, conclusion, and whether analyses were presented in the original submission or included during revision.

**Type of experiment**	**Figure panel**	**Aim**	**Conclusion**	**Submission First/Revised**
**BIOCHEMICAL CHARACTERIZATION OF TTC33 AND TAN**				
Evolutionary conservation of	Figure 1a	Provide evidence of TTC33 expression in various bony	The TTC33 genomic localization and gene	Revised

the TTC33 locus		vertebrates, and support the notion that TTC33 is a functional gene.	organization is conserved.	
Evolutionary conservation of the TTC33 protein and identification of structural motifs	Figure 1b, Supplementary Figure 1a, Supplementary Dataset 1	Provide evidence of TTC33 protein expression in various bony vertebrates. Identify the most conserved parts, which could point to functional relevance.<br><br>Identify the portion of the protein that might produce a conserved TPR fold (formed by 3 adjacent TPR repeats)	The N-terminal and central portions of TTC33 protein, which contain TPR motifs are most conserved. There are 3 adjacent TPRs, which is the minimum required to form a higher order TPR domain.	First: alignment of 12 vertebrate TTC33 proteins.<br><br>Revised: graphical sum up of alignment of 300 vertebrate species, analysis of TPR motif identification by TPPred
Identification of TPR repeats in vertebrate TTC33 proteins	Figure 1c	Identify and position TPR repeat motifs in a collection of 12 vertebrate TTC33 proteins (same as those used in Fig. 1b) using TPRpred.	Most vertebrate TTC33 proteins contain 3 adjacently localized TPR repeats.	Revised
Evolutionary conservation of the CCDC97 locus and protein	Figure 2e, Supplementary Figure 1g, Supplementary Dataset 1	Provide evidence of CCDC97 expression in various bony vertebrates, and support the notion that CCDC97 is a functional gene.<br><br>Provide evidence of CCDC97 protein expression in various bony vertebrates.	CCDC97 is a gene and protein conserved and expressed in bony vertebrates.	Revised
Label Free-Mass Spectrometry analysis of TTC33 interactors purified from various cell lines	Figure 2b, Supplementary Figure 1b-f, Supplementary Dataset 2	Identify TTC33 interactors.	Assay led to identification of TAN factors.	First: TTC33-EGFP purifications from HEK293 and FreeStyle 293-F cells<br><br>Revised: purification of TTC33-EGFP from HeLa cells and MBP-TTC33 from HEK293 cells
Validation of TTC33 interactors by coIP-WB in HEK293 and HeLa cells using MBP-tagged TTC33	Figure 2d	Confirm results of LF-MS analyses from Fig. 2b.	Confirmed the list of TTC33 interactors.	Revised
Expression of TTC33 and TAN factor mRNAs in various tissues from published RNA-seq data	Figure 3a	Assess if TTC33 or any other TAN factors are tissue-specific.	No tissue specificity found.	Revised

Imaging of sub-cellular localization of TTC33 and CCDC97 by microscopy in HEK293	Figure 3b	Assessing TTC33 and CCDC97 sub-cellular localization by GFP-tagged protein imaging in fixed and live cells.	TTC33 and CCDC97 are present in the nucleus and in the cytoplasm. Both have important nuclear fractions. The cytoplasmic CCDC97 fraction is more pronounced.	First: fixed cells<br><br>Revised: live cells
Cell fractionation to assess TAN subunit sub-cellular localization	Figure 3c	Sub-cellular fractionation coupled to western blot analysis of TTC33 and TAN factors.	TTC33 and CCDC97 are present in the nucleus and in the cytoplasm.	First.<br><br>(Revised: lamin marker added at the Reviewer's request)
Imaging of sub-cellular localization of EGFP-tagged PPP2R1A and PPP2CA, and of PPP2R2A by specific antibody by microscopy in HEK293	Supplementary Figure 2a	Define sub-cellular localization of the phosphatase.	The phosphatase catalytic and scaffolding subunits, which partake in many complexes are present in both the nucleus and the cytoplasm, whereas the regulatory subunit, PPP2R2A, is more distinctively nuclear.	First
LF-MS analysis of PPP2R2A interactors in HEK293	Figure 4a	Confirm PPP2R2A association with TAN factors.	Confirmed formation of complex with PPP2R1A and PPP2CA. Some association with CCDC97. FAM122A inhibitor found.	First
CoIP-WB analysis of PPP2R2A interactors in HEK293	Figure 4b	Confirm PPP2R2A association with WDR61 and compare it to TTC33.	Confirmed PPP2R2A association with endogenous WDR61, showed that it is less prominent compared to TTC33.	First
CoIP-WB analysis of PPP2R1A interactors in FreeStyle 293-F	Supplementary Figure 2b	Confirm PPP2R1A association with WDR61 and TTC33.	Confirmed PPP2R1A association with endogenous WDR61 and co-transfected TTC33, used CCT2 as a negative control to show specificity of the method.	First
CoIP-WB analysis of UNG2 interactors in FreeStyle 293-F	Supplementary Figure 2c	Confirm binding of UNG2 to selected TAN factors.	Confirmed association with endogenous WDR61 and co-transfected PPP2R2A and TTC33.	First
LF-MS analysis of WDR61 interactors in HEK293	Figure 4c, Supplementary Figure 2d	Confirm WDR61 association with TTC33 and TAN factors.	Confirmed.	First
LF-MS analysis of CCDC97 interactors in HEK293	Figure 4d, Supplementary Figure 2e	Confirm CCDC97 association with TTC33 and TAN factors.	Confirmed association with TTC33, WDR61, PHF5A and SF3A/B, but did not find UNG1/2.	First

CoIP-WB validation of CCDC97 interactors in HeLa	Figure 4e	Confirm results of LF-MS analysis of CCDC97 interactors in HEK293	Confirmed.	Revised
CoIP-WB validation of UNG1 and UNG2 interactors in HEK293	Figure 4f	Perform reciprocal analysis of UNG1 and UNG2 interactors to validate the LF-MS analysis of CCDC97 interactors.	UNG1 and UNG2 associated with TTC33, WDR61, PHF5A but did not bind to CCDC97 or SF3B4.	Revised
CoIP-WB assessment of TAN factor association with TTC33 in low and high salt (100 vs 300 mM) by immunoprecipitation	Supplementary Figure 2f,g	Perform TTC33 purification using either MBP-TTC33 or TTC33-EGFP to test for robustness of TAN factor binding at two salt concentrations. High salt generally leads to dissociation of weak/transient interactions, while facilitating identification of stable ones.<br><br>Perform purification of EGFP tag alone to test for its unspecific binding to TAN factors.	TTC33 stably associates with WDR61, PHF5A, and CCDC97, but UNG1/2 dissociates from it in IP buffer with 300 mM NaCl. This happens regardless of tag (MBP/EGFP) and whether the interacting partner is endogenous or co-transfected.<br><br>The EGFP tag alone does not precipitate TAN factors (Supplementary Fig. 2f).	First: Supplementary fig. 2g (revised figure mount)<br><br>Revised: Supplementary Fig. 2f
Large scale TTC33 purifications with WDR61, PHF5A, and CCDC97 or UNG2 from FreeStyle 293-F	Figure 5b, Supplementary Figure 3a,b	Attempt to purify the complex.	Consistently purified TTC33, WDR61, PHF5A in stoichiometric levels. In contrast, CCDC97 and UNG2 were always substoichiometric.	First
Size exclusion chromatography of TTC33:WDR61:PHF5A trimer	Figure 5c, Supplementary Figure 3c	Verify formation of the complex.	Confirmed co-migration of TTC33:WDR61:PHF5A trimer in stoichiometric levels.	First<br><br>(Revised: added a larger evaluation of samples by silver-stained SDS-PAGE)
Glycerol gradient from FreeStyle 293-F cells over-expressing of TTC33, WDR61, and PHF5A and from control cells	Figure 5d, Supplementary Figure 3d	Verify putative trimer formation *in vivo*.	Good co-migration of the trimer subunits observed for cells concomitantly co-expressing TTC33, WDR61, and PHF5A	First
Model TTC33 structure: AlphaFold 3 and SwissModel	Figure 6a	Gain insight into TTC33 structure.	TTC33 central domain adopts a TPR fold.	First
Compare TTC33 structural models produced by AlphaFold 3 and SwissModel	Figure 6b	Compare both models.	Models are similar: RMSD 1.775.	Revised

Assess TTC33 TPR domain folds in 11 other vertebrate species	Figure 6c	Gain insight into the TTC33 fold conservation.	The TPR domain fold is conserved.	Revised
Model TTC33:WDR61: PHF5A trimer structure	Figure 6d, Supplementary Figure 4a,b	Gain insight into the TANC structure.	WDR61 and PHF5A are recruited to TTC33 TPR domain independently.	First<br><br>(Revised: added pTM and ipTM scores)
Expected position error plot for TANC model 0	Figure 6e	Assess TANC AlphaFold 3 model quality.	Expected position error low within conserved structural motifs in WDR61, PHF5A, and TTC33 TPR domain.	Revised
Align 5 AlphaFold 3 TANC models	Supplementary Figure 4c,d	Assess reproducibility of TANC modeling with Alpha Fold 3.	Modeling is reproducible.	Revised
Align the PHF5A and WDR61 subunits modeled with AlphaFold 3 with experimental data	Figure 6f	Assess quality of WDR61 and PHF5A modeling by AlphaFold 3.	Models are nearly identical to experimental data (RMSD: 0.452, 0.821).	First<br><br>(RMSD added in revision)
Validate TANC model quality with MolProbity	Supplementary Figure 4e,f	Assess TANC model quality.<br><br>Provide MolProbity report for the model.	TANC AlphaFold 3 model aligns perfectly (RMSD ~0) with the MolProbity corrected one.	Revised
Ramachandran analysis of TANC model produced by AlphaFold 3	Figure 6g	Assess TANC AlphaFold 3 model quality (MolProbity corrected one)	Reasonable quality.	Revised
Validate independent WDR61 and PHF5A recruitment to TTC33	Figure 6h	Perform preliminary validation of TANC model by purifying TTC33:WDR61 and TTC33:PHF5A dimers.	Both TTC33:WDR61 and TTC33:PHF5A dimers could be purified in stoichiometric levels validating the model.	First
Perform molecular dynamics of the AlphaFold 3 TANC model	Figure 6i, Supplementary Figure 4g-l	Assess trimer stability *in silico*.	Trimer stable.	Revised
Close-up on the TTC33:PHF5A interface in the AlphaFold 3 model	Figure 7a,b	Provide information about the TTC33:PHF5A interface in the AlphaFold 3 model.	The PHF5A positively charged C-terminus inserts into the negatively charged TTC33 TPR domain concave.	Revised
Confront the TANC assembly with known SF3B structure	Supplementary Figure 5a	Assess if the proposed TTC33:PHF5A interaction would allow concomitant SF3B recruitment.	Yes.	First
CoIP-WB validation of the PHF5A C-terminus role in	Figure 7c	Validate the TTC33:PHF5A interface experimentally by removing the PHF5A C-terminus.	A PHF5A mutant devoid of the C-terminus lacks interaction with most TAN factors, and has	First<br><br>(Revised version contains improved

TAN formation			decreased binding to UNG1/2 and SF3B4.	coIP-WB analysis)
Molecular docking of the PHF5A C-terminal 9 amino acids	Figure 7d	Verify the docking of the PHF5A C-terminal peptide into the TTC33 concave.	Docking site comparable to the AlphaFold 3 model.	Revised
Molecular dynamics analysis of the TTC33:PHF5A interface	Figure 7e	Follow the evolution of polar bonds between TTC33 and PHF5A throughout a molecular dynamics simulation to pinpoint relevant bonds.	Mapped polar contacts relevant for subsequent TTC33 mutational analysis.	Revised
TTC33 fragmentation analysis	Figure 7g, Supplementary Figure 6b	Fragment the TTC33 protein to assess approximate binding sites for TAN factors.	The TPR domain is key for recruiting PHF5A, WDR61, CCDC97, and PPP2R2A.	First
Map of TTC33 population variants and somatic cancer mutations	Figure 7h, Supplementary Figure 5b, Supplementary Dataset 1	Analyze the repertoire of TTC33 variants for mutational analysis.	Selected 7 population variants or somatic mutations, produced 3 more substitutions with alanine or glycine.	Revised
TTC33 mutational analysis	Figure 7i, Supplementary Figure 5c, Supplementary Figure 6f	Validate the TTC33:PHF5A interaction surface by mutational analysis of TTC33 at sites that build the binding surface.	TTC33 point substitutions at residues Q101, E97, E162, E166, and R135 reduce association with PHF5A.	Revised
		Validate the TTC33:WDR61 interaction surface by mutational analysis of TTC33 at sites that form direct contacts with WDR61.	Two contacts are relevant TTC33_R148: WDR61_D17 and TTC33_H155: WDR61_W20, whereas the TTC33_H125:WDR61_W234 contact was showed not to be relevant	
Close-up on the TTC33:WDR61 interaction surface proposed by AlphaFold 3 with evolutionary conservation and population variants on both sides	Figure 8a	Present the WDR61:TTC33 interaction surface, and assess residue conservation.	Out of the 3 putative amino acid contacts, 2 are evolutionarily conserved.	Revised
Close-up on WDR61 interactions in SKI and PAF complexes.	Figure 8b, Supplementary Figure 6a	Present the WDR61 interaction with TPR proteins in known SKI and PAF complexes.	WDR61 always uses the top surface to bind to its TPR-motif-containing partners.	First
Molecular dynamics analysis of the TTC33:WDR61 interface	Figure 8c	Follow the evolution of polar bonds between TTC33 and WDR61 throughout a molecular dynamics simulation to pinpoint relevant bonds.	Limited the relevant contacts between both proteins to two out of three pairs.	Revised

LF-MS analysis of EGFP-WDR61 WT vs W20C in HEK293 cells	Figure 8d, Supplementary Figure 6c,d	Assess relevance of the WDR61 W20 residue for WDR61 recruitment to its partners: TAN, SKI, and PAF complexes.	The WDR61 W20 residue is key for interaction with its partners.	First
CoIP-WB analysis of EGFP-WDR61 WT vs W20C in HeLa cells	Figure 8e	Verify the findings from Fig. 8d (LF-MS of EGFP-WDR61 WT to W20C) in HeLa cells.	Results consistent.	Revised
Evaluation of endogenous TTC33 levels upon MBP-/EGFP-tagged TTC33 over-expression	Figure 8f	Assess effects of tagged TTC33 over-expression on endogenous TTC33.	Tagged TTC33 over-expression leads to down-regulation of endogenous TTC33.	First
Analysis of endogenous TTC33 mRNA levels by RT-qPCR in control cells and those expressing tagged exogenous TTC33	Supplementary Figure 6f	Inquire if endogenous TTC33 was down-regulated after tagged TTC33 expression because of alterations in mRNA levels.	No.	Revised
Analysis of TTC33 levels in HEK293 cells over-expressing tagged TTC33 and exposed to MG132 proteasomal inhibitor	Supplementary Figure 6g	Assess if down-regulation of endogenous TTC33 protein levels upon over-expression of tagged TTC33could be rescued by addition of MG132.	Yes, it can be rescued by MG132.	Revised
Analysis of WDR61 and TTC33 levels by western blot in cells expressing microRNAs against WDR61, and treated with MG132	Figure 8g	Assess the function of WDR61 in respect to TTC33, and the mechanism of TTC33 regulation.	WDR61 depletion using microRNAs leads to concomitant down-regulation of TTC33, which can be rescued by addition of proteasomal inhibitor MG132.	Revised
Analysis of WDR61 and TTC33 levels by western blot in cells expressing microRNAs against WDR61 and EGFP_WDR61_ WT or W20C insensitive to microRNAs	Figure 8h	Verify if TTC33 down-regulation in WDR61-knocked-down cells could be rescued by expression of a WT WDR61.	Yes.<br><br>WDR61 W20C mutant unable to bind TTC33 also unable to rescue the phenotype of TTC33 down-regulation.	Revised
Assess functionality of TTC33-EGFP and	Supplementary Figure 6h	Elucidate why TTC33-EGFP is expressed at much lower levels than other constructs (MBP-	Each protein has a different efficiency of precipitation of TAN	First

EGFP-TTC33_ *codon_optimized* by coIP-WB		TTC33 and EGFP-TTC33_*codon_optimized*).	factors.	
**PHENOTYPIC/ FUNCTIONAL CHRACTERIZATION OF TTC33 AND TAN**				
Comet assay	Figure 9b	To assess existence of DNA double-strand breaks in TTC33 and UNG knock-down.<br><br>DNA DSBs were shown to occur in UNG1/2 knocked-down cells by comet test by Pulukuri et al., 2009 (*doi: 10.1158/1541-7786.MCR-08-0508.*).	Chromatin in nuclei isolated from TTC33 and UNG knocked-down cells is more fragmented compared to control.	First
IF γH2AX	Figure 9c, Figure 9d, Figure 9e,<br><br>Supplementary Figure 7a,b	Evaluate *in vivo* marker of DNA double-strand breaks in TTC33 and CCDC97 knock-down by microRNA (Fig. 9c), and CRISPR-Cas9 knock-out (Fig. 9d). Test γH2AX accumulation in siRNA depleted TTC33 (Supplementary Fig. 7b).<br><br>Evaluate *in vivo* marker of DNA double-strand breaks in WDR61 knock-down with functional rescue using the EGFP-WDR61 WT and W20C (the latter is impaired in binding to TTC33) (Fig. 9e).<br><br>Elevated γH2AX was shown in PPP2R2A-depleted cells by Kalev et al., 2012 (*doi: 10.1158/0008-5472.CAN-12-1667*) and confirmed herein (Supplementary Fig. 7a).	Nuclear γH2AX levels are increased in TTC33, CCDC97 and WDR61 depleted cells. A phenotype that has been previously assigned to knock-down of PPP2R2A, which is an interactor of TTC33.<br><br>Importantly, in case of TTC33 increased nuclear γH2AX levels were observed in micro-, and siRNA knocked-down HEK293 cells, and in CRISPR-Cas9 deleted HEK293 cell line.<br><br>In case of HEK293 cells microRNA depleted of WDR61 the nuclear accumulation could be rescued by expression of a wild-type WDR61 insensitive to microRNAs, but not by the W20C mutant, which is incapable of binding to TTC33.	Revised
Uracil glycosylase assay (whole cell extract dsDNA oligo containing U:A)	Supplementary Figure 7c,d	Verify if alteration in TTC33 or CCDC97 levels can impact catalysis by UNG.<br><br>To test if altered BER activity could be the source of DNA DSBs in TAN-depleted cell lines.	No clear impact on UNG catalysis.	First<br><br>Revised: quantification added
Proliferation rate	Figure 10a	Assess if micro-RNA knock-down of TTC33, CCDC97, and UNG1/2 resulted in a decreased growth rate. Assess if over-expression of TTC33 or UNG2 decreased proliferation.	Knock-down of TTC33, CCDC97 results in decreased proliferation.<br><br>Over-expression of TTC33 and UNG2 also delays growth.	First for TTC33 and CCDC97,<br><br>but Revised for miUNG1/2 and UNG2 overexpression

		Rationale: considerable amounts of DNA damage in the cell will induce cell cycle checkpoints or apoptosis, which will translate to decreased overall growth.		
Proliferation rate	Figure 10b	Assess if TTC33 loss in cancer derived cells will also result in a delay in proliferation.<br><br>Validate the proliferative defect of HEK293 cells depleted of TTC33 using siRNA mediated knock-down.	Knock-down of TTC33 has a greater impact on growth of cancer-derived A549, HeLa, and HCT116 cells than embryonic kidney HEK293.<br><br>HEK293 cells treated with siRNAs against TTC33 had a tendency for slower growth, validating findings obtained with microRNAs.	Revised
IF p53-S15P	Figure 10c, Supplementary Figure 8a,b,c,d	p53-S15P is a marker of DNA damage cell cycle arrest. Assess if this marker of genotoxic stress was present in cells with altered TAN factor levels in HEK293 and A549 cells.<br><br>Validate if effects of miRNA mediated depletion of TTC33 could be validated with siRNAs.	p53-S15P is re-distributed in all tested conditions and cell lines.<br><br>Results obtained with microRNAs were validated with siRNAs.	First for miTTC33 (Fig. 10c, Supplementary Fig. 8a), miUNG1/2, as well as UNG1/2 and MBP-TTC33 overexpression (Supplementary Fig. 8e)<br><br>Revised for siRNA mediated knock-down of TTC33 in HEK293 and A549 (Supplementary Fig. 8c,d)
Assess percentage of cells in each cell cycle phase	Figure 10d	Identify the most likely reason for a delayed proliferation phenotype in TTC33- and CCDC97-depleted cells, which was shown in Fig. 10a and 10b.	TTC33- and CCDC97-depleted cells have a decrease in the number of cells in G1 phase and an increase in the fraction in G2. This indicates activation of a pre-mitotic cell cycle check point.	Revised
Assess BrdU incorporation as a marker of DNA synthesis	Figure 10e	Assess the level of DNA synthesis in TTC33- and CCDC97-depleted cells.	BrdU incorporation is increased in TTC33- and CCDC97-depleted cells. Assessed together with a decreased proliferation, this indicates ongoing DNA repair.	Revised
Test for sensitivity to reactive oxygen species (ROS)	Figure 10f	Assess if cells with altered TTC33 or CCDC97 levels were particularly sensitive to ROS.	Compared to control, all tested cell lines (depleted of TTC33, CCDC97, or UNG1/2; or over-	First<br><br>(though data recalculated to

		Re-evaluate published data (Bao et al., 2020: *doi: 10.1016/j.freeradbiomed.2020.06.010*; Liu et al., 2016: *doi: 10.1016/j.freeradbiomed.2016.07.030*) indicating that UNG depletion sensitized cells to ROS.	expressing TTC33 or UNG2) were more sensitive to ROS.<br><br>This was manifested by doubling of the proliferative phase after hydrogen peroxide treatment. Data was presented differently following Reviewer 3 comment.	present elongation in the proliferative phase)<br><br>Revised: included data on UNG1/2 depletion or UNG2 over-expression
Evaluation of the role of TTC33 and TAN factor levels in clinical data	Figure 10g,h, Supplementary Figure 9b	Assess if expression of TAN factors is linked to disease states.	TTC33 mRNA levels decrease with cancer stage in KIRC. Increased expression of TAN factors is favorable to survival.	Revised

RESPONSE TO REVIEWER 1

Reviewer #1 (Remarks to the Author):

The manuscript by Drabko et al. unveiled the TTC33-associated network (TAN) of protein interactions contained WDR61, PHF5A, and the SF3B subunits, PP2A-B55α phosphatase (PPP2R2A), UNG DNA glycosylase, and CCDC97 protein by a comparative label-free mass spectrometry. They identified the center of TAN as a trimeric core complex, including TTC33:WDR61:PHF5A through subunit co-purification in stoichiometric levels and co-migration in size exclusion chromatography. Furthermore, they computationally modeled the TANC structure and experimentally validated that WDR61 and PHF5A bind independently to opposite sides of TTC33 TPR4, and the TPR1-3 region is sufficient to recruit other TAN factors. Functionally, the imbalanced expression of TTC33 adversely affects cellular proliferation rates and resistance to genotoxic agents. However, the article lacks detailed evidence explaining the function of each subunit and whether TAN is involved in cell proliferation and resistance to genotoxic agents. Additionally, the absence of in vivo experiments further limits the study's overall impact and significance. Below are several concerns that need to be addressed.

This is the first study on TTC33 and CCDC97. In this work we intended to primarily characterize the biochemical relationships of TTC33 by identifying its interactors and binding patterns. We were fortunate to identify a novel complex, and in the revised version we show that it mediates mutually exclusive interactions with either UNG1/2 or SF3A/B and CCDC97. **In the revised manuscript, we improve on the mechanism of TTC33:WDR61:PHF5A core complex assembly, by performing molecular dynamics and extensive TTC33 mutational analysis. In addition, we show the crucial role of WDR61 in maintaining the stability of TTC33.**

The second goal of our work was to associate a phenotype with TTC33 deficiency that could eventually point towards the protein's function. Thanks to the insightful comments we received from all Reviewers, we were able to define that **loss of TTC33, and most of its interactors, leads to an increase in DNA double-strand breaks**, which is manifested by nuclear accumulation of γH2AX. Importantly, we now **demonstrated the specificity of this phenotype by performing a functional rescue**, and showed that the previously observed associated **growth defect is related to cell cycle arrest in G2**. We are aware that a detailed mechanism of TTC33's role in controlling genomic stability is yet undefined, but we strongly feel that deciphering this issue is out of the scope of this first ever work on TTC33.

As the phenotypic and functional section raised most of the Reviewer's concerns, and since we have made extensive changes, we briefly sum up our main findings in the preamble and below (B, D, F, G, I, and J points are novel data):

A. WDR61 ensures the TTC33 protein stability through direct interaction. In WDR61 absence, TTC33 is susceptible to degradation by the proteasome **(Fig. 8g,h)**.

B. The main phenotype associated with impairment of TAN subunits (TTC33, CCDC97, WDR61, UNG1/2, and PPP2R2A) is accumulation of DNA double-strand breaks (DNA DSBs). This was shown using a comet test for TTC33 and UNG1/2 knock-downs **(Fig. 9a,b)**, and by immunofluorescence of γH2AX (*in vivo* marker of DNA DSBs) upon TTC33, CCDC97, WDR61, and PPP2R2A downregulation **(Fig. 9c-e, Supplementary Fig. 7a,b)**. We also show a functional rescue of this phenotype in cells simultaneously expressing microRNAs against endogenous WDR61 and an ectopic EGFP-WDR61 WT variant not sensitive to miRNAs. Importantly, a WDR61 variant incapable of binding to TTC33 does not rescue γH2AX nuclear accumulation upon endogenous WDR61 knock-down **(Fig. 9e)**.

C. Decreased proliferation associated with TTC33 and CCDC97 depletion, observed using microRNA knock-down in HEK293 (**Fig. 10a**).

D. The TTC33 proliferation defect was also replicated using siRNAs in HEK293, A549, HCT116, and HeLa cell lines, and shown for PPP2R2A knock-down (**Fig. 10b**).

E. Accumulation of p53-S15P, observed by immunofluorescence in HEK293 cells knocked-down for TTC33

using si- and microRNAs. This phenotype is also observed in cells with altered UNG1/2 levels. p53-S15P has functions in cell cycle arrest linked to DNA damage (Loughery et al., 2014 *doi: 10.1093/nar/gku501*) (**Fig. 10c, Supplementary Figure 8**).

F. We show that cells knocked-down for TTC33 or CCDC97 have increased percentage of cells in G2 and decreased number of cells in G1 phase, indicative of cell cycle arrest (**Fig. 10d**).

G. Increased BrdU incorporation in cells depleted of TTC33 or CCDC97, combined with slower proliferation, indicates ongoing nucleic acid synthesis related to DNA repair (**Fig. 10e**).

H. We show that cells depleted of TTC33 or CCDC97 have equally increased sensitivity to hydrogen peroxide (reactive oxygen species source) as cells depleted of UNG1/2 or over-expressing UNG2 (**Fig. 10f**).

I. Using publicly available clinical data, we show that in kidney clear cell adenocarcinoma (KIRC) mRNA coding for TTC33 is down-regulated with cancer stage, and that higher TAN factor expression is associated with increased chances of survival (**Fig. 10g,h**).

J. We performed a mutational analysis of TTC33 using population variants and somatic cancer mutations. Affecting residues responsible for recruitment of WDR61 or PHF5A also impacts UNG1/2 recruitment (**Fig. 7i and Supplementary Fig. 5c**).

1 The study suggests that TTC33 influences cell proliferation and the DNA damage response. However, whether TTC33 exerts this effect through its interaction with PHF5A and WDR61 or if it regulates the DNA damage response independently is unclear. The current evidence is insufficient, and further investigation is needed to clarify the specific mechanism by which TTC33 functions in cellular processes.

In the revised version, we show that WDR61 is required to stabilize TTC33 *in vivo*. In WDR61 absence, TTC33 protein levels drop, and can be rescued by addition of MG132 proteasome inhibitor. TTC33 protein can be also rescued by expression of a wild-type WDR61 but not the W20C mutant that cannot bind to TTC33. This shows that a direct interaction between TTC33 and WDR61 is required to protect TTC33 from decay. This is shown in revised Fig. 8g,h, and associated Supplementary Fig. 6f,g.

As a direct response to this question, we believe that TTC33 partners are required to express the protein's function. That is why during our search for TTC33 function we compared the phenotypes related to its loss with those of its partners. In the original version we showed that nuclei of TTC33 or UNG1/2 depleted cells are more fragmented (comet test; Fig. 9a,b). Increased levels of DNA double-strand breaks (DSBs) in UNG1/2-depleted cells were previously reported (Pulukuri et al., 2009: *doi: 10.1158/1541-7786.MCR-08-0508*; Weeks et al., 2014: 57. *doi: 10.1038/cddis.2013.477*). **γH2AX is a direct *in vivo* indicator of DNA double-strand breaks. In the revised version, we showed that HEK293 cells microRNA-depleted of TTC33, or CCDC97, or WDR61 accumulate γH2AX (Fig. 9c,d). We observed increased nuclear γH2AX levels in HEK293 cells CRISPR-Cas9-deleted of TTC33 or CCDC97. We also replicated the phenotype in HEK293 cells siRNA-knocked down of TTC33. Finally, we showed that in HEK293 cells microRNA-depleted of WDR61 increased γH2AX levels can be brought down to control levels by expression of a wild-type WDR61 insensitive to microRNAs, but not with the W20C mutant that is incapable of binding to TTC33 (Fig. 9e).**

Increased levels of γH2AX were also previously observed by Kalev et al., (2012) in conditions of PP2A-B55α-depletion, which suggests that this histone variant might be a target of the phosphatase (see *doi: 10.1158/0008-5472.CAN-12-1667*, Figure 3 therein). During revision we knocked-down PPP2R2A and observed nuclear accumulation of γH2AX, corroborating those results (Supplementary Fig. 7a). In relation to PHF5A, Begum et al. (2021) showed by ChIP that loss of PHF5A impairs γH2AX deposition at the immunoglobulin site, but on whole cell extracts loss of PHF5A was associated with increased γH2AX (see *doi: 10.15252/embj.2020106393*, Figure 6B and 6C therein). **Given the new experimental evidence we put forward in our revised manuscript, and the published data, we conclude that loss of most, if not all, TAN subunits leads to increased DNA DSBs.** This is shown in revised Fig. 9, and associated Supplementary Fig. 7a,b.

At this stage of our research, we are unable to put forward a clear mechanism for DNA DSB

formation in TAN-deficient cells, but we strongly feel that this is a work for a separate dedicated publication. Simply, the possibilities are too many for a short revision time-frame. Given our glycosylase assays, we doubt that TTC33 strongly influences UNG1/2 catalysis (Supplementary Fig. 7c,d). However, DNA DSBs were suggested to also arise from non-catalytic UNG1/2 functions (Begum et al., 2009: *doi: 10.1073/pnas.081325210*; Yousif et al., 2014: *doi: 10.1073/pnas.140239111*), and impaired PPP2R2A activity (Kalev et al., 2012: *doi: 10.1158/0008-5472.CAN-12-1667*). In addition, transcription-related processes, such as R-loop formation, are also known to induce DNA DSBs (Bader and Bushell, 2020: *doi: 10.1038/s41419-020-2464-6*).

2. The authors mention that TTC33 protein levels are regulated in vivo, but the underlying regulatory mechanisms remain unclear. It is not determined whether TTC33 is controlled at the transcriptional or translational level or if its degradation is mediated by the ubiquitin-proteasome system. Investigating the mechanisms governing TTC33 homeostasis would enhance the study's depth.

We have in fact partly answered this question above. Following the Reviewer's comment, we further investigated the mechanism related to TTC33 level regulation. We show that in conditions of MBP-/EGFP-tagged TTC33 over-expression, addition of MG132 proteasome inhibitor can rescue the levels of the endogenous TTC33 protein (Fig. 8f and Supplementary Fig. 6g). We further confirm that the endogenous TTC33 mRNA levels are not altered in conditions of MBP-/EGFP-tagged TTC33 overexpression (Supplementary Fig. 6f). This allowed us to conclude that TTC33 is regulated through proteasomal decay.

Next, we showed that depletion of WDR61 also leads to TTC33 down-regulation, and that this protein decrease can also be rescued by MG132 (Fig. 8g). Finally, we demonstrated that TTC33 down-regulation in WDR61-depleted cells can be rescued by expression of a wild-type EGFP-WDR61, but not by a EGFP-WDR61 W20C mutant, which cannot interact with TTC33 (Fig. 8h). This indicates that a direct interaction between TTC33 and WDR61 is key for maintaining TTC33 stability.

3 The characterization of TTC33-related cellular phenotypes lacks specificity. While the findings suggest that TTC33 abundance affects cell proliferation and oxidative stress resistance, no functional rescue experiments have been conducted to validate these effects. Additionally, it remains uncertain whether PHF5A and WDR61 mediate these phenotypic changes. Addressing these points would help strengthen the study's conclusions.

We believe that both PHF5A and WDR61 partake in phenotypic changes observed in TTC33-deficient cells. The main advance in the research we produced during revision was to demonstrate that TTC33, CCDC97, WDR61, or PPP2R2A depleted cells accumulate nuclear γH2AX, suggesting increased DNA double strand breaks. This phenotype was previously shown for PPP2R2A- and PHF5A-depleted cells (Kalev et al., 2012: *doi: 10.1158/0008-5472.CAN-12-1667 → Fig. 3*; Begum et al., 2021: *doi: 10.15252/embj.2020106393 → Fig. 6c*).

<u>The question of phenotype specificity is an important one, which we have seriously addressed during revision.</u> The main reason why we did not perform a direct functional rescue of TTC33 depletion was related to the observation that the protein over-expression caused down-regulation of the endogenous one (Fig. 8f). A proliferative effect and nuclear accumulation of p53-S15P, associated with over-expression, led us to suspect that the protein large excess could be detrimental (Fig. 10a, Supplementary Fig. 8e). However, one of our coIP-WB experiments showed that each tagged TTC33 variant precipitated TAN factors with different efficiency (Supplementary Fig. 6h). Therefore, we could not be sure how much the over-expressed TTC33 can compensate for the endogenous one, and if it did not simply replicate the effects of TTC33 depletion (meaning that over-expression was enough to down-regulate endogenous TTC33, but insufficient to fully compensate). **In the revised version, to further validate the TTC33-related phenotypes, instead of performing a direct functional rescue, we decided to diversify the methods for removing TTC33, believing that if those resulted in the same phenotype, the conclusion would be as sound as if we performed a direct functional rescue. Thus, we have observed γH2AX accumulation in micro- and siRNA TTC33-depleted cells, and in knock-out cell lines. Additionally, we could achieve a functional rescue of nuclear γH2AX accumulation using WDR61 (point 'c' below, and response to question 2 above), which solved the main issue (Fig. 9e).** In sum, in the revised version we:

a. validate the nuclear γH2AX accumulation using TTC33 and CCDC97 microRNA-mediated knock-down and CRISPR-Cas9 knock-outs of respective coding genes (Fig. 9c,d);
b. validate the nuclear γH2AX accumulation using TTC33 siRNA-mediated knock-down (Supplementary Fig. 7b);
c. perform a functional rescue of γH2AX nuclear accumulation in microRNA WDR61-depleted cells expressing wild-type or W20C mutant (Fig. 9e);
d. validate the re-distribution of p53-S15P in microRNA TTC33-depleted HEK293 cells using siRNA-mediated depletion (two siRNAs were tested; Fig. 10c, Supplementary Fig. 8b, and Supplementary Fig. 8b,c);
e. validate the growth phenotype related to TTC33 loss, induced by expressing microRNAs in HEK293 cells, by using siRNA-mediated knock-down. We further show that TTC33 knock-down in three different cancer-derived cell lines (HeLa, A549, and HCT116) results in an even more pronounced proliferation delay than in HEK293 (Fig. 10b), and that marked re-distribution of p53-S515P could be observed in A549 (Supplementary Fig. 8d).

4 Phosphorylation of p53 at serine 15 (Ser15) is used as a marker of DNA damage. However, whether TTC33 also influences other DNA damage markers, such as γ-H2AX is unclear. Additional experimental evidence is needed to support TTC33's role in regulating the DNA damage response further.

We are grateful for this remark, which helped us to greatly improve our phenotypic analysis. Indeed, γH2AX is a more direct marker of DNA double-strand breaks. The revised Figure 9 and associated Supplementary Figure 7a,b show that depletion of TTC33, CCDC97, WDR61, or PPP2R2A leads to a comparable increase in nuclear γH2AX levels. This phenotype was observed in microRNA TTC33-, CCDC97- or WDR61-depleted cells, and in case of WDR61 could be functionally rescued by expression of a wild-type WDR61, but not with the mutant impaired in TTC33 binding. Nuclear γH2AX accumulation was also observed in siRNA TTC33-depleted, and in TTC33 or CCDC97 CRISPR-Cas9 knock-out cells.

5 The study states that TTC33 is evolutionarily conserved in bony vertebrates but does not assess whether its function is preserved across species. Has the structure or function of TTC33 changed over evolution? Have the authors examined its conservation from a genomic or evolutionary perspective? A more detailed discussion on the functional conservation of TTC33 across species would be beneficial.

It is true that in the primary submission we only stated that TTC33 is conserved in bony vertebrates, and showed a simple multiple protein sequence alignment from 12 exemplary vertebrate species. In the revised version we improve on this analysis:

Figure 1a: we show a genomic alignment of the *TTC33* locus from vertebrate species, which demonstrates that the gene is consistently neighbored by *PRKAA1* and *PTGER4* genes, and that *TTC33* mRNA is spliced from four coding and one 5'UTR exon.

Figure 1b (top graph): we re-evaluate TTC33 protein sequence conservation, using an alignment from 300 vertebrate species. This showed that TTC33 sequence was strongly conserved within the N-terminus and TPR2-3-4 region, supporting the notion that the latter is the protein's main structural feature. We still show the detailed alignment from the 12 species as a visual reference (bottom part of the figure).

Figure 1c and **Supplementary Figure 1a:** in the Supplement, we show the sequences of the four human TPR repeats detected by TPRpred algorithm along with the p-value (Karpenahalli et al., 2007: *doi: 10.1186/1471-2105-8-2*; https://toolkit.tuebingen.mpg.de/tools/tprpred). We also used TPRpred to detect TPR repeats in the 12 protein sequences from representative vertebrate species that were previously shown in Fig. 1b. In the Fig. 1c, we display each protein's length, location of putative TPR motifs, and the p-value range. This showed that in all vertebrate species there are 3 centrally located TPRs, which are adjacent to one another. Since the minimum number of TPR repeats that can form a TPR fold is exactly three (Zeytuni and Zarivach, 2012: *doi: 65. 10.1016/j.jmb.2017.12.007*), we presume that evolutionary pressure operated to preserve this central structure. Depending on the species, other less well distinguished (lower p-value) TPRs were sometimes predicted at the N- or C-termini of TTC33. Additionally, we identified NLS sequences in

those 12 selected TTC33 proteins. Data summing NLS and TPR coordinates in those selected species, and exact NLS_scores/TPR_p-values are listed in Supplementary Dataset 1.

Figure 6c: we performed an AlphaFold 3 structural modeling of the core TPR2-3-4 domain from various vertebrate species (the 12 protein sequences shown in Fig. 1b), and aligned the obtained models. This showed that the TPR2-3-4 domain (residues 42-168 of the human protein) is consistently modeled similarly, regardless of species. Most models' pTM score, including the human one, is above 0.8, which indicates that the structures are highly reliable. The average RMSD of each vertebrate structure with the human reference is 0.33, confirming high similarity. As an additional verification, we aligned the human TPR2-3-4 model with the full-length TTC33 extracted from the TANC model and this comparison again showed a RMSD of 0.332.

Altogether, we can now state that the genomic structure of *TTC33* locus is conserved, and so is the length of the protein itself. Within the presumed structure of TTC33 protein, the most conserved is the TPR2-3-4 domain, which we show is responsible for recruitment of PHF5A and WDR61, and formation of the TANC complex (Fig. 6, Fig. 7g → AlphaFold 3 modeling and TTC33 fragmentation assay). Given those observations, we can surmise that TTC33 (and likely the TANC complex) is present and exerts its functions in many vertebrate species.

Since CCDC97 is also largely uncharacterized, we similarly analyzed its genomic locus (Supplementary Fig. 1g) and protein sequence conservation across 300 species (Fig. 2e). This showed that while the genomic location of CCDC97 is conserved, conservation of the amino acid sequence at the protein level displayed less stringency, compared to TTC33.

6 Only one cell line was used in the study, making it unclear whether the complex is commonly found in mammalian cells, which limits the significance of these findings.

Originally, two cell lines were used: HEK293 adherent cells and FreeStyle 293-F suspension cells. Both differ not only in adherence to solid surface, but also in growth media and milieu (5% vs 8% CO_2). Nevertheless, since FreeStyle 293-F derive from HEK293, we can see that both systems might be too similar.

To counter this issue, in the revised version we:

a. perform label-free mass spectrometry analysis on TTC33-EGFP interactors purified from HeLa cells. This showed that TTC33 co-purified with the entire set of TAN factors previously identified in HEK293 and FreeStyle 293-F cells (Fig. 2d; Supplementary Fig. 1f; Supplementary Dataset 2).

b. perform additional co-immunoprecipitations coupled to western blot in HeLa cells for key experiments:

1. Figure 2d: coIP-WB to confirm TAN factor co-purification with TTC33 in HEK293 and HeLa cells side-by-side. Here, a MBP-tagged TTC33 version was used. A change of tag further confirmed the specificity of the interactions;

2. Figure 4e: coIP-WB of EGFP-CCDC97 in HeLa cells to confirm results of LF-MS obtained in HEK293 cells for MBP-CCDC97 (Fig. 4d);

3. Figure 7i and Supplementary Fig. 5c: the mutational analysis of TTC33 was performed in HEK293 and HeLa cells;

4. Figure 8e: coIP-WB from HeLa validating the TAN factor co-purification with WDR61 wild-type protein, but not with the W20C mutant. The LF-MS data was originally obtained in HEK293 cells (Fig. 8d), and we previously also validated this result in HEK293 cells (Supplementary Fig. 6c,d).

c. in revised Fig. 3a we evaluate TTC33 expression across tissues using the datasets published by Fagerberg

et al. (2014: *doi: 10.1074/mcp.M113.035600*). In this study, the authors produced transcriptomic profiles of a collection of human tissues. This shows that TTC33 and CCDC97 are widely expressed in most human cell types, and their respective mRNAs are only roughly two-fold lower in abundance compared to their better described TAN partners: UNG, PPP2R2A, PHF5A, *etc.*

Considering all the data that we gathered so far, we think we can safely conclude that TTC33 forms the TANC complex and interacts with other TAN factors in cells of various tissues.

7 The biological function of TTC33 in cell proliferation was also assessed in a single cell line, which is insufficient to support its general role in proliferation.

Yes, the proliferation phenotype was originally only established in a stable HEK293 cell line expressing microRNAs directed against TTC33 mRNA under the control of a tetracycline-inducible promoter. This showed a small but reproducible effect over the course of 3, 6 or 9 days depletion (Fig. 10a). We see how this phenotype should have been further validated.

In the revised version, we used two siRNAs to deplete TTC33 from a wider panel of cell lines of different origin, including HEK293, A549, HCT116, and HeLa cells. We observed a significant tendency towards slower proliferation in embryonic kidney-derived HEK293 cells (Fig. 10b). Given that siRNAs are scarcely transfected with a nearly 100% efficiency, we believe that this result supports our previous findings obtained using endogenously and stably expressed microRNAs. **Importantly, cell lines derived from cancer patients, A549 (lung), HCT116 (colon), and HeLa (cervix), displayed a much more pronounced proliferation delay** after transfection with TTC33-directed siRNAs (Fig. 10b). **In case of A549 and HEK293, we confirmed the cellular re-distribution of p53-S15P (a marker of DNA damage-related cell cycle arrest) using a siRNA-mediated TTC33 knock-down** (Supplementary Fig. 8c,d), which we previously observed in HEK293 cells expressing microRNAs (Fig. 10c; Supplementary Fig. 8b).

We now also show that the proliferative defect of TTC33-depleted cells is shared not only by CCDC97-depletion, but also UNG2 over-expression and PPP2R2A knock-down (Fig. 10a,b).

Additionally, we calculated the number of cells in G1/S/G2 phases in HEK293 control and cells depleted of TTC33 or CCDC97, and defined that loss of either protein led to an increase in number of cells in G2, and concomitant decrease in G1, which points to a cell cycle arrest (Fig. 10d).

8 The experiments examining TTC33's role in cell proliferation are insufficient. Does it specifically affect certain stages of the cell cycle? Does it influence apoptosis?

The Reviewer is right that we did not sufficiently examine this question. For the purpose of this point-by-point response, we examined SYTOX incorporation in A549, HCT116, and HeLa cells depleted of TTC33 using siRNAs. Cells knocked-down for TTC33 did not accumulate SYTOX, in spite of displaying a lower proliferation rate compared to cultures treated with control siRNAs (proliferation rate: Fig. 10b; SYTOX accumulation panel A in Figure below). Since SYTOX preferentially enters dying cells, with this experiment we excluded the possibility of increased apoptosis/necrosis.

Next, we hypothesized that cells depleted of TTC33 likely have issues related to cell cycle progression (duration of particular phases of cell division). Pursuing this matter further, in the revised version we show that TTC33, CCDC97, or WDR61 depletion leads to an increase in the fraction of cells that are in G2, with a concomitant decrease in those that are in G1 and S phases (revised Fig. 10d and panel B in Figure below). We conclude that cells knocked-down for TTC33 have an extended duration of the G2 phase.

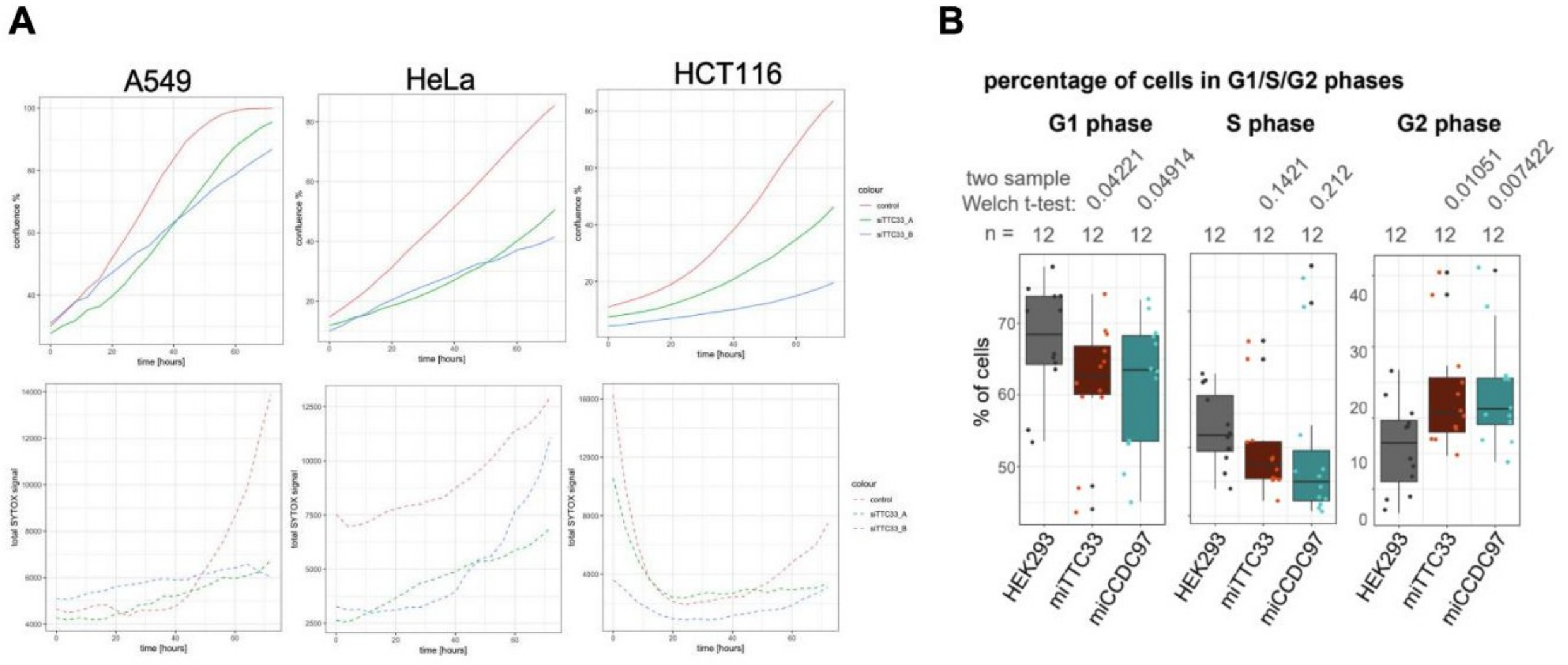

Response Figure. A. *IncuCyte S3 analysis of cell confluence and SYTOX incorporation. Top row shows A549, HCT116, and HeLa cell culture confluence following transfection with control siRNA or two stealth siRNAs directed against TTC33. The bottom plots show the level of SYTOX incorporation in respective cells. Please note that SYTOX is heavily incorporated in the A549 control culture, when it attains maximum confluence, indicating that the assay is functional.* ***B.*** *Graph shows novel Figure 10d. The fraction of cells in G1, S, and G2 was estimated for HEK293 control, and cells depleted of TTC33 or CCDC97. Two biological replicates are shown, each composed of six technical replicates shown as dots (and given as 'n'). Each dot represents a mean value obtained from analysis of few hundred cells (typically 300).*

9 The biological function of TTC33 has not been well characterized. How it affects DNA damage remains unclear. Additionally, whether TTC33 commonly influences DNA damage in mammalian or cancer cells is unknown.

The main aim of our work at this stage was to identify TTC33 interactors and characterize their biochemical relationships. We intended to demonstrate a phenotype tied to TAN, which could point towards a biological function executed by its interaction network. We believe that with the novel data put forward in the revised version, we strengthen a notion of TTC33 role in DNA damage control, but we strongly believe that a detailed analysis merits an independent study. In relation to the phenotypic study, we:

a. performed a comet test, which showed that nuclei of HEK293 cells depleted of TTC33 and UNG are more fragmented, indicating that they have increased DNA double-strand breaks (Fig. 9a,b);
b. showed by IF that depletion of most of TAN factors results in nuclear accumulation of DNA damage markers (γH2AX and p53-S15P) in HEK293 cells. The p53-S15P nuclear re-distribution was also confirmed in A549 (Fig. 9 and Supplementary Fig. 7a,b for γH2AX, and Fig. 10c, Supplementary Fig.8b,c,d,e for p53-S15P);
c. showed that HEK293 cells knocked-down for TTC33 have a delayed cell cycle (Fig. 10d), and demonstrated that the growth of cancer-derived A549, HeLa, and HCT116 cells is more affected after TTC33 knock-down than HEK293 (Fig. 10b);
d. showed that HEK293 cells depleted of TTC33 and CCDC97 are more sensitive to reactive oxygen species, which is in line with the observations pointing to impaired DNA repair (Fig. 10f);
e. showed that expression of TTC33 and TAN factors is relevant to survival of patients with KIRC (Fig. 10g,h, Supplementary Fig. 9b).

Altogether, this data strongly points towards the relevance of TTC33 and TAN for genomic integrity. We agree with the Reviewer that the underlying mechanism is obscure. Since we could not detect any major impact of TTC33 or CCDC97 on UNG1/2 catalytic function (Supplementary Fig. 7c,d), TAN must influence some repair mechanisms other than the initial BER glycosylase step. When there are multiple lesions in the

DNA, repair pathways often act concomitantly. For example, excision of bases on opposite strands by the BER mechanism can lead to formation of double strand breaks, which are either repaired by homologous or non-homologous end joining. UNG2 induces such DSBs at the immunoglobulin locus during antibody class-switch recombination (CSR), to the repair of which PHF5A was also suggested to contribute (Begum et al., 2021: *doi: 10.15252/embj.2020106393*). Interestingly, outside the catalytic activity, UNG was also proposed to play alternative scaffolding roles in CSR (Begum et al. 2007: *doi: 10.1074/jbc.M607439200*; Begum et al., 2009: *doi: 10.1073/pnas.0813252106*; Yousif et al., 2014a: *doi 10.1073/pnas.1402391111*; Yousif et al., 2014b: *doi:10.1093/intimm/dxu071*). However, seemingly controversial, this putative scaffolding role is also shared by the mitochondrial UNG isoform, which protects the DNA from reactive oxygen species (Liu et al., 2016: *doi: 10.1016/j.freeradbiomed.2016.07.030*). UNG also partakes in repair at replication forks, countering base misincorporation in opposite uracil (Weeks et al., 2014: *doi:10.1038/cddis.2013.477*). In addition, PP2A-B55α phosphatase was also ascribed a role in resolution of γH2AX upon DNA damage repair after homologous recombination (Kalev et al., 2012: *doi: 10.1158/0008-5472.CAN-12-1667*). **Altogether, various TAN factors play roles related to homologous or non-homologous end joining, and we predict a likely role of TTC33 in either in those processes. We now more clearly discuss this in the final section of the publication. However, we strongly believe that careful dissection of this putative role is more suitable for an independent story.**

10 In Figure 1h, TTC33, CCDC97, and PPP2R2A are shown to be independently localized in the nucleus. To demonstrate their co-localization within the nucleus or the subunits of TANC (TTC33, WDR61 and PHF5A), it would be beneficial to show their co-expression within the same cell using immunofluorescence (IF).

This assay was one of the three experiments performed to determine the sub-cellular localization of TTC33 and CCDC97. The other two were cell fractionation coupled to western blot analysis (now Fig. 3c), and microscopic localization of EGFP-tagged TTC33 or CCDC97 in fixed cells (now Fig. 3b). Both assays revealed a mixed nucleo-cytoplasmic localization. We now confirmed this by performing microscopic localization of EGFP-tagged TTC33 or CCDC97 in live cells (now Fig. 3b). In this context, the IF performed using protein-specific antibodies gave a nearly exclusively nuclear localization for both proteins. Given the divergence from the two other results, and the fact that neither of the dedicated antibodies was recommended for IF by the manufacturer, we removed this result.

We understand the Reviewer's concern in this point. Confirming co-expression of factors in the same cell is important to ensure the specificity of the investigated interaction. However, we do not believe there is a reason to doubt the ubiquitous expression of TTC33 or CCDC97 in homogeneous HEK293 cell line. In general, mRNA levels are a good indicator of protein expression. In the novel Fig. 3a we show that mRNAs encoding TTC33 or CCDC97 are expressed in various human tissues at a just two-fold lower level compared to other TAN factors, WDR61, PP2A-B55α, SF3A/B, which also partake in other prominent complexes, and are expressed in every cell.

11 In this manuscript, the authors utilized co-IP experiments to analyze the association of target proteins under exogenous overexpression conditions. However, it would be important to investigate the endogenous interactions among these components.

Yes, this is rather common strategy that we, and many others before us, consequently chose to follow. In our study we performed coIPs coupled to label-free mass spectrometry and western blot using either MBP-TTC33 or TTC33-EGFP (Fig. 2b,e). In stable cell lines the expression of TTC33-EGFP bait was not much different than that of the endogenous protein (Fig. 8f). Thus, in fact, two (HEK293, HeLa) out of our four datasets assessing TTC33 interactors come from experiments performed on near-endogenous TTC33 levels. The results are not different from those obtained with much higher TTC33-EGFP expression in FreeStyle 293-F and using MBP-TTC33 in HEK293. This is justified by the fact that cross-comparison of either dataset leads to the same list of strong TAN interactors (see Supplementary Fig. 1f → filtering example; Supplementary Dataset 2 → full filtering procedure, and Methods).

In case of EGFP, the experiments were performed using a magnetic resin (either home-made or commercially available from Chromotek). The MBP tag was precipitated using either α-MBP antibody-coated magnetic beads or amylose resin. Either experimental setting produced roughly equal results. The

associations were then re-tested reciprocally using tagged CCDC97, WDR61, and UNG as baits. Experiments performed with CCDC97 and UNG as baits revealed interesting new observations that point towards a mutually exclusive interaction of TTC33:WDR61:PHF5A with either CCDC97-SF3B and UNG (new data shown in the Fig. 4e,f). Especially considering that we revealed those mutually exclusive interaction, this shows that none of the tags alone mediates interactions with either TAN factor, excluding the possibility that the employed technical setting produced false positive results. In addition, in the novel Supplementary Fig. 2f we performed a solitary EGFP immunoprecipitation and showed that in contrast to TTC33-EGFP the tag alone did not co-purify the TAN factors.

Taken altogether, we think that our approach was rather standard, and since we diversified the tags, their placement on the baits' termini, and resins, the likelihood of producing unspecific interactions is very low.

12 In Figure 2e, the authors demonstrated that high salt concentrations tend to reduce protein-protein interactions. However, since the proteins appear to have been developed on different films, it is difficult to determine whether the observed differences between low and high salt conditions are due to protein interaction strength or differences in exposure time.

This assay was originally performed to determine whether any TAN factors could form a stable complex with TTC33, and was shown as a conceptual introduction to the later purification and SEC analysis of the TTC33:WDR61:PHF5A trimer. We did take a shortcut in putting the result together, for which we apologize. Originally each salt concentration had the inputs loaded on the same gel, and we now show the full image below, and the relevant lanes in the revised Supplementary Fig. 2g. Additionally, we replicated the result by assessing TTC33-EGFP interaction with endogenous WDR61, PHF5A, CCDC97, and UNG1/2, and the result was identical. This new data is shown Supplementary Fig. 2f.

In the revised version we provide more solid evidence for trimer formation. We extensively validated our original LF-MS data obtained from MBP-CCDC97 protein purifications from HEK293 cells. This dataset had confirmed co-purification of TTC33, WDR61, PHF5A, and the SF3B complex (Fig. 4d). However, the UNG1/2 glycosylase was not identified, even though it was abundantly detected in TTC33 and WDR61 purifications. During revision we confirmed this result by coIP-WB using a different cell line and tag (HeLa; EGFP) (Fig. 4e). Furthermore, we performed reciprocal purification of EGFP-UNG1/2, which showed that the glycosylase co-purified with TTC33, WDR61, PHF5A, but not with CCDC97 or SF3B4 (Fig. 4f). This suggested that PHF5A can be taken out of the context of the SF3B complex to interact together with TTC33 and WDR61 in a mutually exclusive manner with either UNG1/2 or CCDC97 and SF3B.

We also would like to remind that for the sake of transparency we deposited all the raw data at Mendeley (*doi: 10.17632/p822vhnbdj.1*; link: https://data.mendeley.com/preview/p822vhnbdj?a=d2b464dd-21dc-4b68-abb1-0f9798a1e353). The dataset can be viewed with a link listed in the data availability section, after logging in to a personal Mendeley account.

Supplementary Fig. 2g

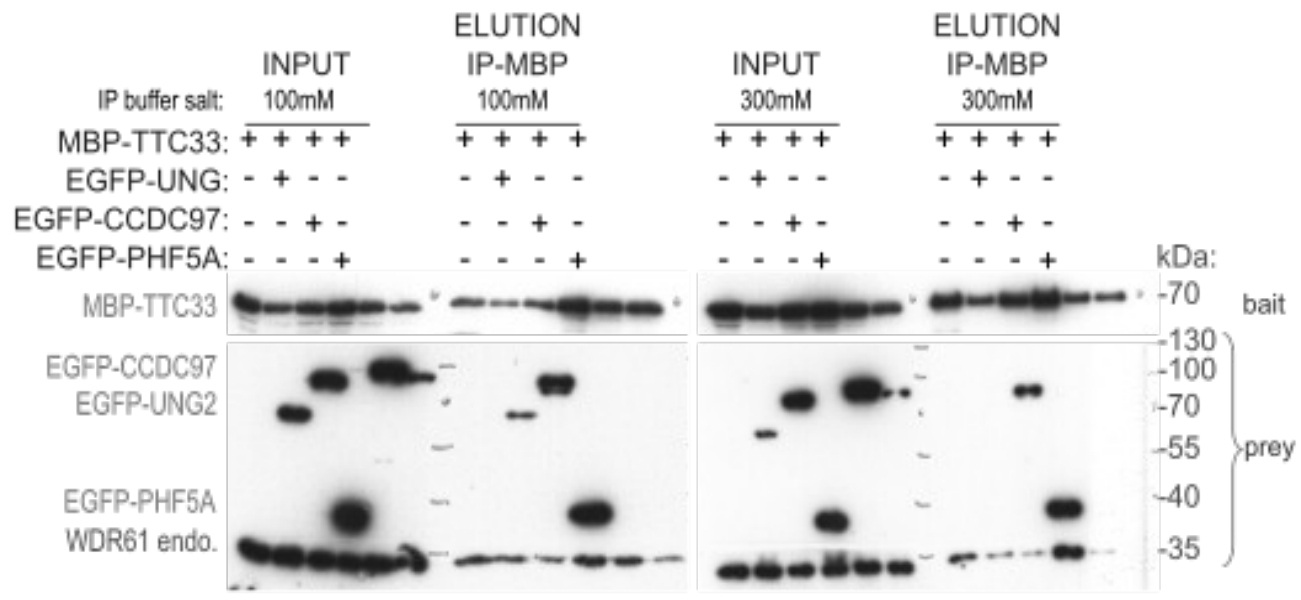

Supplementary Fig. 2f

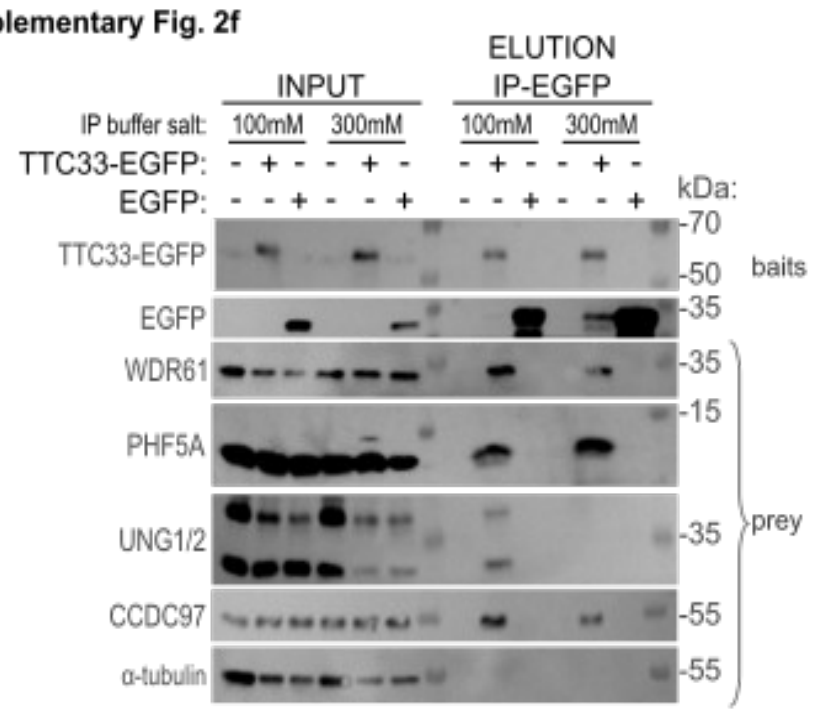

Two panels from Supplementary Fig. 2. ***Fig. S2g.*** *CoIP-WB analysis of proteins co-purifying with MBP-TTC33 from FreeStyle 293-F cells under 100 vs 300 mM NaCl concentration. EGFP-tagged CCDC97, UNG2, or PHF5A were co-transfected with the MBP-TTC33 bait. Endogenous WDR61 was detected using protein specific antibody. Those are results of a coIP-WB experiment presented in the original submission, but we show the full blots with each elution being loaded on the same gel with its inputs. These blots are now displayed in the supplement, but cropped to remove unnecessary lanes.* ***Fig. S2f****. CoIP-WB analysis of TAN factors co-purifying with TTC33-EGFP from FreeStyle 293-F cells under 100 vs 300 mM NaCl concentrations. TAN factors were detected using protein-specific antibodies. EGFP was also expressed as an additional negative control to show that the tag alone does not interact with endogenous TAN factors. This is a novel experiment, performed during revision, in which only endogenous proteins interacting with transfected TTC33-EGFP were detected. The tag on the TTC33 bait was altered to cross-verify the result presented in Supplementary Fig. 2g.*

13． In Figure 3i, the authors propose a face-to-face conformation between WDR61 tryptophan 20 and TTC33 histidine155. However, the discussion later focuses solely on the effect of the WDR61 W20C point mutation without considering TTC33. The impact of the TTC33 mutation should also be addressed.

The Reviewer is right that the mutual site of interaction within TTC33 should have been confirmed. Therefore, in the revised version we now provide a mutational analysis of TTC33 (Fig. 7i, Supplementary Fig. 5c). The H155 residue, which faces WDR61 W20, has been substituted with alanine or tyrosine. The latter mutation is reported as a somatic cancer variant (H155Y: COSV100531955). Both substitutions, but particularly H155A, resulted in a marked decrease of TTC33:WDR61 association, thus validating the interaction.

We also more closely examined the TTC33:WDR61 interaction surface and selected two other possible contacts: TTC33_R148:WDR61_D17 and TTC33_H125:WDR61_W234 (Fig. 8a). We examined the evolutionary conservation of each TTC33 residue (Fig. 8a), their movement during a molecular dynamic simulation (Fig. 8c), and performed additional mutational analysis (Fig. 7i, Supplementary Fig. 5c). This showed that the conserved TTC33_R148 was more relevant to WDR61 binding than the TTC33_H125, which is only preserved in 54% of vertebrates.

Importantly, in our revised work we have also carried out such mutational analysis for the TTC33:PHF5A interface. Where possible, population or somatic variants of TTC33 were tested. This

analysis is now presented in revised Fig. 7i and Supplementary Fig. 5c.

14 In Figure S3h, it is difficult to accurately assess the binding efficiency due to unequal expression levels in the Input.

This co-immunoprecipitation was repeated. Now the inputs are balanced, and we probed for more TAN factors. The new result is shown in Figure 7c. Briefly, removal of the terminal 9 amino acids in PHF5A results in loss of interaction with TTC33, CCDC97, WDR61, and a severely reduced association with UNG1/2, and SF3B4. The latter result indicated that even though the C-terminus does not enter in contact with the SF3B complex (Supplementary Fig. 5a → image of SF3B structure from Cretu et al., 2016: *doi: 10.1016/j.molcel.2016.08.036*), it is still important for overall protein integrity. Therefore, to cross-validate the TTC33:PHF5A interaction surface, we performed molecular docking experiments (novel Fig. 7d), and TTC33 mutational analyses, including somatic cancer mutations and population variants, where possible (novel Fig. 7h, 7i, novel Supplementary Fig. 5c)

15 This manuscript does not demonstrate whether this complex is related to human disease and lacks in vivo experiments to verify its function.

Following the Reviewer's comment about the lack of link of TTC33 to human diseases, we undertook two actions. As stated above, **we assessed the biochemical effects of several population and somatic variants of TTC33 (Fig. 7h, 7i, Supplementary Fig. 5b,c), and found that the amino acid substitutions caused by mutations not only reduce associations with WDR61 or PHF5A, but also affect recruitment of UNG1/2 DNA glycosylase.** Thus, the cellular proteome is likely disturbed by these rare variants. Though none of them is as-of-yet directly linked to disease state, they are clearly detrimental to the protein function. Given that the increased cancer risk is most often related to polygenic factors, we think that this shows that rare TTC33 variants can contribute to the risk. **Second, to support this notion, we re-analyzed existing cancer transcriptomic data provided by The Cancer Genome Atlas (Fig. 10g,h, Supplementary Fig. 9b). This showed that TTC33 levels decreased with cancer stage in patients with kidney renal clear cell carcinoma (KIRC). Furthermore, increased expression of all TAN factors was associated with better overall survival.** This is apparently in line with our results demonstrating that TAN protects genome integrity. Importantly, the body of our functional results were obtained in kidney-derived HEK293 cells.

Additionally, we improved the phenotypic characterization of TTC33 and its partners, to more reliably show that loss of most TAN factors was associated with increased genotoxic stress. The main phenotypes arising from TAN factor knock-down are related to increased DNA double-strand breaks, which were originally identified by a comet test in TTC33- and UNG1/2-depleted cells (Fig. 9a,b). In the revised version, we support this discovery by immunofluorescence of the γH2AX marker in TTC33, CCDC97, WDR61, and PPP2R2A knock-down cells (Fig. 9c-e; Supplementary Fig. 7a,b). Additionally, cells with altered TTC33, CCDC97 and UNG1/2 levels also display a re-distribution of p53-S15P marker of DNA damage related cell cycle arrest (Fig. 10c, Supplementary Fig. 8a-e). We showed that TTC33 depletion caused a slight decrease in proliferation of HEK293 cells, and strongly affected cancer-derived A549, HCT116, and HeLa cells (Fig. 10b). This decreased proliferation rate was caused by a delay in G2 cell cycle phase (Fig. 10d). An increased incorporation of BrdU (Fig. 10e), associated with slow proliferation, and a sensitivity to ROS (Fig. 10f), further support the notion of increased ongoing DNA repair.

DNA DSBs can result *i.a.* from increased BER activity (which is unlikely considering our glycosylase assays), abnormal resolution of R-loops, impaired NHEJ or homologous repair, and issues with replication fork stability. Given the number of possible sources, it is beyond our current capabilities to provide a molecular mechanism in this submission. This is a question more suitable for future studies, once a dedicated funding is secured.

RESPONSE TO REVIEWER 2

Reviewer #2 (Remarks to the Author):

I co-reviewed this manuscript with one of the reviewers who provided the listed reports. This is part of the NatureCommunications initiative to facilitate training in peer review and to provide appropriate recognition for Early Career Researchers who co-review manuscripts.

We thank the Reviewer for the effort put into evaluating and improving our work.

RESPONSE TO REVIEWER 3

Reviewer #3 (Remarks to the Author):

In "Comparative mass spectrometry reveals a novel TTC33-associated network core (TANC) complex with functions in DNA Damage response", Drabko and colleagues investigated the cellular role of the unknown protein TTC33. They first performed a mass-spectrometry based screening for interactors, then validated such interactions with Co-IP,discovering the formation of a complex with WDR61 and PHF5, which was then further explored with in silico modelling followed by Co-IP with point mutations. Other interactors, including with the Uracil-DNA glycosylase UNG1/2 were also further validated as less strong/frequent by co-IP. Based on this latest interaction, the authors focused on the possibility that TTC33 could be involved in DNA damage response and suggest that TTC33 levels could be relevant for genome stability
The cellular function of TTC33 is, as far as this reviewer knows, unknown, so the screening by interaction is interesting as it could potentially elucidate it. However, the strongest interactions found and studied, both in cells and in silico, are with WDR61 and PHF5, both involved in RNA metabolism, but the authors did not comment on this at all **(see RESPONSE A)**. On the contrary, they decided to further characterize the role of TTC33 in DNA damage response due to the finding of a weaker interaction with UNG1/2. However, this interaction is not well characterized (which regions of TTC33 are required? Is always occurring? Does it require WDR61 and PHF5?) **(see RESPONSE B)**, and the characterization of the possible role of TTC33 in DNA damage is too preliminary to be conclusive (Does TTC33 depletion increase sensitivity to DNA damaging agents? Does it affect repair of UNG/APX induced single-stranded breaks? Or of DSBs? Or of replication?) **(see RESPONSE C)**, significantly reducing the possible impact of this study.

Our work was mainly biochemical – we primarily focused on identifying the TTC33 interaction network and the relationships between its components. We were fortunate to characterize a novel complex, and during revision we greatly improved on the mechanism of its assembly, by performing molecular dynamics and a mutational analysis of TTC33, using population variants and somatic cancer mutations. We also demonstrated that the main role of WDR61 in the complex was related to conferring TTC33 stability by protection from proteasomal decay. This wouldn't be possible without the valuable insight from all Reviewers, for which we are grateful. In the frame of this initial study, we wished to pin a phenotype to TTC33 and its partners, and here again, we thank the Reviewers for useful suggestions. As a result of this major revision, we can now state that loss of TTC33 and its interactors leads to formation of DNA double-strand breaks, manifested by nuclear accumulation of γH2AX. Importantly, this phenotype can be functionally rescued, which demonstrates its specificity. As to the exact mechanism of DNA DSB formation, and its possible relationship to transcription or splicing, we strongly feel that it is a subject for a prospective grant application and separate study. Above, 3 main points were raised by the Reviewer. We rephrased them, and provided answers below.

RESPONSE A (to question: Is a role of TTC33 in RNA metabolism likely, given its interaction with WDR61 and PHF5A?):

As the Reviewer pointed out, both WDR61 and PHF5A play roles in RNA metabolism. Obviously, we considered whether TTC33 could also partake in the regulation of RNA expression, either at the level of transcription, RNA stability control, or splicing. In the early stages of this project, we performed RNA sequencing experiments, in which we evaluated the transcriptomic profiles of HEK293 cells depleted of TTC33 or CCDC97, with a depth of over 50 mln reads, to evaluate not only differential expression but also alternative splicing. Depletion of CCDC97 altered the expression of only about 100 mRNAs/ncRNAs, whereas knock-down of TTC33 had only a slightly better effect with roughly 400 differentially expressed transcripts. The total number of human coding RNAs alone estimated at 21000. Thus the low number of affected transcripts, and the small overlap between knock-downs discouraged us from thinking about a major role in RNA metabolism. Using the rMATS algorithm, we identified around 1000 alternatively spliced mRNAs, but evaluation of the data on IGV, and subsequent unsuccessful validations, led us to think that the high number of targets were identified largely due to the considerable depth of the sequencing. Altogether, we have considered the possibility of a TTC33 role in RNA metabolism, but we are currently not convinced. Perhaps the TTC33 and TAN role is limited to a small number of transcripts, and with a larger transcriptomic

cross-evaluation of TAN factors, other than TTC33 or CCDC97, we will identify it. However, this is at present outside of our funding targets, and more suitable for an independent project. Given, the consistent results showing a γH2AX nuclear accumulation, which is most likely linked to DNA DSBs, in many TAN mutants, we lean more naturally towards the DNA damage control hypothesis.

<u>RESPONSE B (to question: What is the biochemical relationship of UNG1/2 with TTC33 and CCDC97?):</u>

B1. Which regions of TTC33 are required for interaction with UNG1/2? Does it require WDR61 and PHF5A?

In our coIP-WB TTC33 fragmentation assays we did not show UNG1/2, which is the reason for the Reviewer's important question. TTC33 fragmentation led to full loss of UNG1/2 binding (see panel A in the Figure below). The result itself was not very informative, and rather suggested that the binding site is complex and spread out throughout the entire protein, thus we did not include it in the original version. We could not model this interaction using AlphaFold 3, as UNG2 was modeled in a different conformation each time, even though the TANC complex was modeled similarly (see panel B in the Figure below). We know from our previous high-throughput analyses that a WDR61 point mutant W20C loses interaction with TTC33 and all other TAN factors, including UNG1/2 (Fig. 8d,e). Thus, it is rather unlikely that WDR61 mediates interaction with UNG1/2 directly. During revision we also improved on the analysis of PHF5A mutant lacking the C-terminus, and we observed that though binding to UNG1/2 is decreased in the mutant, it is not fully abolished (Fig. 7c), thus PHF5A might contribute to the binding surface, but is not solely responsible for binding to UNG1/2. Lastly, we performed an extensive TTC33 mutational analysis (Fig. 7i; Supplementary Fig. 5c). Though mutants were designed mainly to validate the TTC33:PHF5A or TTC33:WDR61 interaction surfaces, we observed that mutating key residues engaged in PHF5A or WDR61 binding leads to a decrease in UNG1/2 levels. All but one of the mutations had a low ΔΔG factor, indicating that there was a low likelihood of a strong influence on TTC33 structure (Fig. 7h). **Altogether, we think that the UNG1/2 interaction surface is primarily formed of multiple contacts along TTC33 protein, with a slight support from PHF5A. Even though the data presented in the publication point toward this conclusion, we chose not to elaborate on it. This question (contrary to the one about the architecture of the TANC trimeric complex, which we successfully described based using *in silico* modeling tools) can only be definitely answered with genuine structural studies (see panel B in the Figure below), which are out of the scope of the present study, and our funding capacity. Here, we are content with identifying TTC33 and UNG1/2 as interactors, which is perfectly enough to introduce the reader to the ensuing phenotypic analysis.**

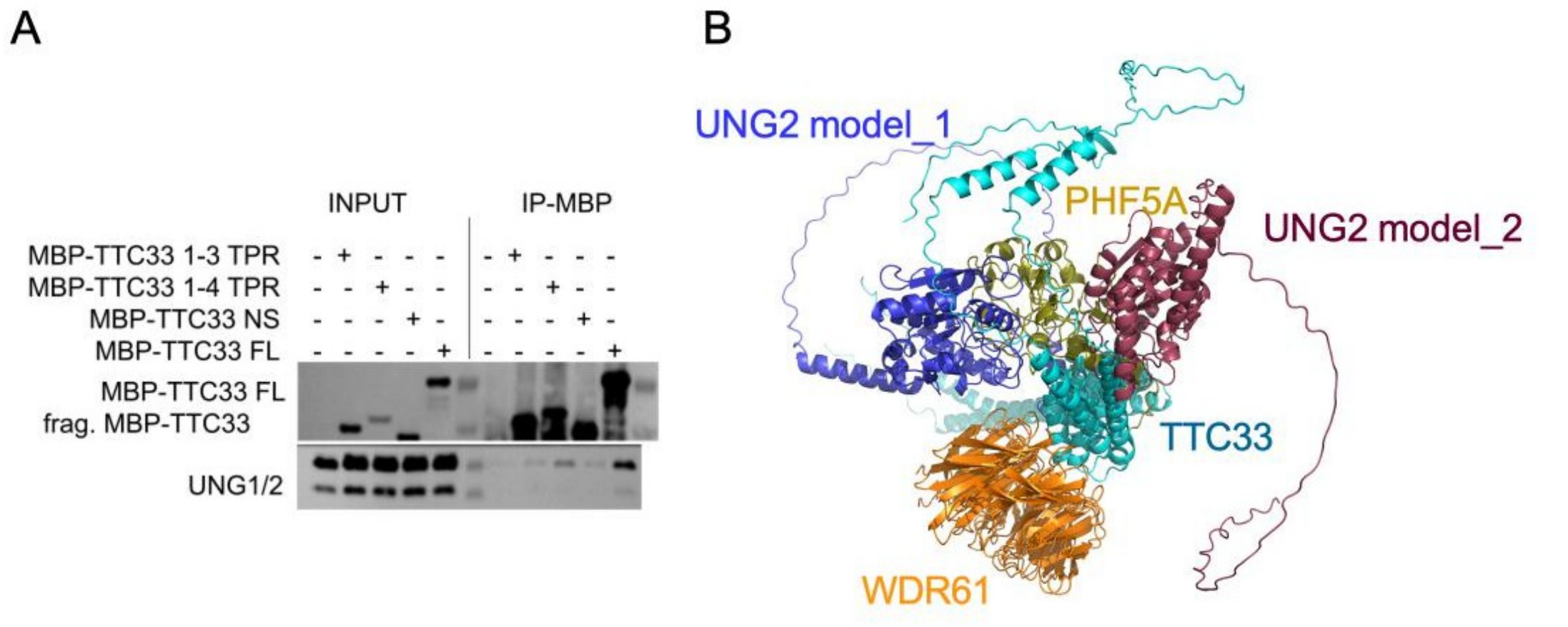

Figure. A. *coIP-WB analysis of UNG1/2 co-purification with MBP-TTC33 full-length or fragments.* ***B.*** *Attempt at modeling of the TANC complex with UNG2. Two models are structurally aligned, and in each the UNG2 protein is placed at a different location in relation to the TANC trimer. This shows that AlphaFold 3 modeling cannot be used to predict TANC interaction with UNG1/2.*

B2. Is the TTC33:UNG interaction always occurring?

No. In fact, in the revised version of the manuscript we provide new data that shows that the TTC33 interaction network is actually composed of two sub-networks. A trimer of TTC33:WDR61:PHF5A (TANC) forms mutually exclusive interactions with either UNG1/2 or CCDC97-SF3B. This revised data is now presented in the Figure 4.

Specific data pointing to this conclusion are rooted in our label-free mass spectrometry of CCDC97 interactors (HEK293 cells; MBP-tag), presented in the original version of the publication (now Fig. 4d). There, we confirmed TTC33, WDR61, PHF5A, and SF3B as CCDC97 binding partners. However, we did not identify UNG1/2 in this dataset. Initially, we dismissed this result, but with more validations, performed during revision (Fig. 4e), we understood that the result from high-throughput proteomics was specific. Our novel coIP-WB performed in HeLa cells using an EGFP-tagged CCDC97 as bait also did not identify UNG1/2 as interactors, though other TAN factors were consistently detected. Consequently, we performed a purification of EGFP-tagged UNG1 and UNG2 isoforms from HEK293 cells (Fig. 4f), and the WB analysis showed that both UNG isoforms interacted with TTC33, WDR61, PHF5A, but neither interacted with CCDC97 nor with the SF3B4 subunit of SF3B. This suggested that TTC33:WDR61:PHF5A interact either with UNG1/2 or with CCDC97 and SF3B complex proteins.

Functionally, we show that loss of both TTC33 and CCDC97 results in increased DNA DSB manifested with nuclear accumulation of γH2AX. Thus, even though CCDC97 forms no direct, physical link to UNG1/2, it does play a role in TTC33-related genomic integrity surveillance (see response below). Here, we can only speculate on the mechanism, but we strongly feel that an in-depth analysis is more suited for the next study.

<u>RESPONSE C (to question: How supported in our study is a role of TTC33 in DNA repair, and is it only hypothesized from a TTC33:UNG interaction?):</u>

Among TTC33 interactors, UNG1/2 is the most obvious one linked to DNA repair. However, a role in genomic stability is also played by the PP2A-B55α and PHF5A. PPP2R2A regulatory subunit of PP2A phosphatase was proposed to be involved in resolution of DNA double-strand breaks after irradiation, and its depletion is marked *in vivo* by elevated γH2AX levels (Kalev et al., 2012: *doi: 10.1158/0008-5472.CAN-12-1667*). In the revised work we confirmed that PPP2R2A knock-down elevates nuclear γH2AX levels by IF (Supplementary Fig. 7a). In turn, PHF5A, in addition to its role in splicing as the subunit of SF3B, was proposed to partake together with p400 chaperone in deposition of γH2AX histone variant and resolution of double-strand breaks at the immunoglobulin locus (Begum et al, 2021: *doi: 10.15252/embj.2020106393*). Thus, DNA repair is a major function played by many TAN factors, justifying our experimental efforts in this area.

C1. Does TTC33 depletion increase sensitivity to DNA damaging agents? Does it affect repair of UNG/APX induced single-stranded breaks? Or of DSBs? Or of replication?

We would like to emphasize that we performed a comet test on control cells and their counterparts depleted of TTC33 or UNG1/2 already in the original version of our manuscript (now Fig. 9b). This technique shows the level of DNA fragmentation in the nuclei, and loss of both TTC33 and UNG1/2 clearly increased DNA fragmentation. This was previously reported by other authors in case of UNG1/2 knock-down (Pulukuri et al., 2009: *doi: 10.1158/1541-7786.MCR-08-0508*). A most direct marker of DNA double-strand breaks was the nuclear accumulation of γH2AX. During the revision, we systematically assessed γH2AX nuclear levels, and found them to be increased in knock-downs of TTC33, CCDC97, WDR61, and PPP2R2A (Fig. 9c,e; Supplementary Fig. 7a,b). This phenotype was replicated in TTC33 and CCDC97 knock-out cells (Fig. 9d). **<u>Thus, the main phenotype associated with loss of TAN factors is DNA DSB accumulation.</u>** TTC33 has only a minor impact on uracil excision in our *in vitro* tests (Supplementary Fig. 7c,d), thus the mechanism of DNA DSB occurrence is unclear to us.

DNA damage induces either apoptosis or cell cycle arrest. We observed that HEK293 cells knocked-down for TTC33 or CCDC97 **have slower proliferation (Fig. 10a,b), and accumulate nuclear levels of**

p53-S15P (Fig. 10c; Supplementary Fig. 8b,c), which is a marker of cell cycle arrest in anticipation of DNA repair. In the revised version, we show that these two phenotypes can be replicated in other cancer-derived cell lines (A549, HeLa, and HCT116; Supplementary Fig. 8d). We also assess the percentage of cells in G1, S, and G2 cell cycle phases, and discover that **down-regulation of TTC33 or CCDC97 proteins increases the number of cells in G2, and decreased in G1** (Fig. 10d). This supports the notion that loss of either protein results in cell cycle arrest.

The occurrence of increased genotoxic stress in cells depleted of TTC33 or CCDC97 is also supported by other minor observations. We show that despite decreased proliferation, TTC33- or CCDC97-knocked-down cells have increased intake of BrdU, which indicates higher DNA synthesis (Fig. 10e). Together with lower proliferation, this supports the notion of ongoing DNA repair. Also, **depletion of CCDC97, TTC33, or UNG1/2 sensitizes cells to hydrogen peroxide (source of reactive oxygen species)** (Fig. 10f**)**. Sensitivity to ROS is a common phenotype associated with impaired DNA repair.

Finally, given the increased levels of genotoxic stress in HEK293 cell lines, we reasoned that this phenotype on an organismal level could be mutagenic and accelerate carcinogenesis. Thus, we re-analyzed publicly available clinical data and observed that TTC33 mRNA levels decreased with cancer stage in kidney clear cell adenocarcinoma (KIRC), and that high expression of all TAN factors was associated with higher survival rates (Fig. 10g,h; Supplementary Fig. 9b). This indicates that levels of TTC33 and its partners might be relevant to disease progression.

In sum, we provide ample evidence that TAN is relevant to genomic integrity by protecting cells from DNA DSBs, though the mechanism is likely not directly linked to the efficiency of uracil excision by UNG1/2. However, this is not startling, given the clear roles of PP2A-B55α and PHF5A in DNA DSB repair (see above).

Other points:
1. The validation of the interactions found by LF-MS in Fig.2 and Fig.S1-2-3 is extensive but very confusing. It would be much clearer to show first the validation for the interactions with TTC33 and then to expand the analysis to cross-interactions between the different component of the proposed TAN network and/or the weaker interaction. This is particularly relevant considering that the authors do focus on the TANC.

We followed the Reviewer's suggestion and modified the text and Figures accordingly. Both batches of high-throughput datasets are now supplemented with novel validations performed by coIP coupled to western blot. Those validations are shown next to the high-throughput mass spectrometry results.

<u>**SET1:**</u>

Figure 2b → high-throughput mass spectrometry (TTC33-EGFP purified from HEK293 and FreeStyle 293-F presented previously, and new datasets: TTC33-EGFP purified from HeLa and MBP-TTC33 purified from HEK293);
Figure 2d → validation of high-throughput proteomics by coIP-WB. New experiment was performed, in which MBP-TTC33 was purified either from HEK293 or HeLa. We changed tags to show that regardless of tag (EGFP or MBP) TTC33 always purified TAN factors;

<u>**SET2:**</u>

Figure 4a → high-throughput results for MBP-PPP2R2A purified from HEK293;
Figure 4b → coIP-WB for MBP-PPP2R2A or MBP-TTC33 used as baits, showing comparison of WDR61 binding;

<u>**SET3:**</u>

Figure 4d → high-throughput results for MBP-CCDC97 purified from HEK293;
Figure 4e and 4f → coIP-WBs for EGFP-CCDC97 purified from HeLa or EGFP-UNG1/EGFP-UNG2 purified from HEK293.

2. Why does the TANC complex run so much smaller than expected (Fig.2h)? Furthermore, the glycerol gradient (Fig.S2d) and, less, the SEC (Fig.2h) show that CCDC97 co-localize with the TANC (even more than PHF5A), but this is not really addressed in the text.

Based strictly on the regression line produced by the column calibration (Panel A in the Figure below), the TANC trimer seems to migrate around the size of 100 kDa (Panel B in the Figure below), which is smaller than expected, but bigger than the individual components (70.9 kDa: MBP-TTC33; 34.6 kDa: FLAG-WDR61, 40.5 kDa: EGFP-PHF5A). We now included a broader analysis of SEC fractions by silver-stained SDS-PAGE gel (revised Figure 5c and Panel C in the Figure below), and it is clear that the entire peak contains all three subunits at stoichiometric levels, and none of the individual subunits is present in subsequent fractions, indicating that TTC33:WDR61:PHF5A assembled into the trimer very efficiently, and none of TANC subunits migrates as a monomer. That said, it is hard to determine the exact mass of the trimer in SEC, as based on the relationship of the series of mass standards and elution volumes, there is a large 95% confidence interval (Panel B in the Figure below). Nonetheless, the TANC complex migration does fall within this expected interval.

Migration of proteins in size exclusion chromatography is tied to their hydrodynamic radius, which depends on both the protein molecular weight and its compactness. WDR61 and PHF5A are both globular, and this is apparent from structural studies (Cretu et al., 2016: *doi: 10.1016/j.molcel.2016.08.036*; Vos et al., 2018: *doi: 10.1038/s41586-018-0440-4*). WDR61 and PHF5A monomers should thus migrate in accordance with the column calibration. In turn, our structural modeling indicates that TANC complex is not globular, but instead strongly elongated with a large disordered C-terminal region of TTC33 (accounting for 21% of the complex; Supplementary Fig. 4c,d). Such disordered regions can slow a protein/complex migration in SEC, as loose peptides will interact with the porous column. We believe that this might be the main reason for a slightly delayed migration of the TANC complex in SEC. Notably, SEC alone provides only approximation of a protein/complex molecular weight. To determine it accurately, it should be coupled with MALS (Multi-Angle Light Scattering; SEC-MALS), but this technique requires typically the amount of material within the range of tens-to-hundreds of micrograms, which is far more than we are currently able to obtain following purification from human cells. Perhaps our planned follow-up studies on the structure of TANC with UNG or CCDC97, will allow us to address this issue further. In this case, we will need to switch to a more efficient recombinant expression system with the aim of getting TANC trimer and associated TAN factors in higher quantities and of greater purity. However, we strongly believe that this extensive additional biochemical work is out of the scope of the presented study.

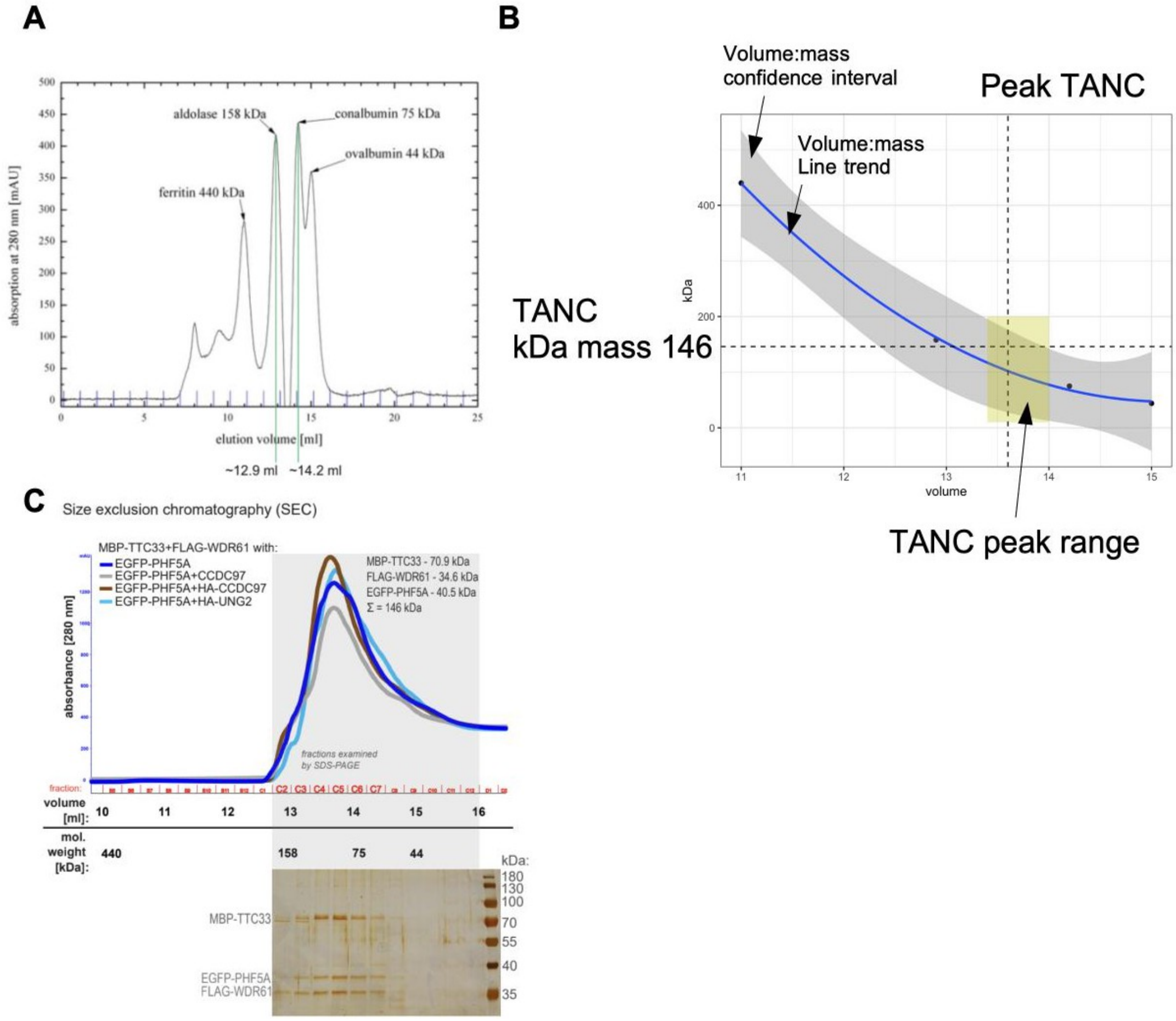

***Figure. A.** Graph shows SEC column calibration manual. **B.** Graph plots the SEC column mass and elution volume (black dots), and draws a trend line (blue) with a 95% confidence interval (grey area). Dashed lines indicate the TANC peak elution volume and the expected complex size, while the yellow area indicate the peak elution volume range. **C.** Panel shows the revised Figure 5c with the novel silver-stained SDS-PAGE gel containing more fractions (as requested in one of the points below).*

3. The dissection of the interactions between TTC33 and WDR61/PHF5A in Fig.3 is interesting, but I do not agree with the conclusions. For example, CCDC97 does not interact with C-term of TTC33, but it does with any fragment containing TPR2, so the interaction could be more limited. My suggestion is to add a table for all the tested domain/interaction. Also, have you tested TPR-2 or TPR4 alone?

We agree that CCDC97 likely binds to the N-terminal part of the TPR domain, and that TPR4 is not required. This is based on the fact that most TPR-containing fragments, including those devoid of TPR4, bind to CCDC97 (now Supplementary Fig. 6b). However, we do not believe that the CCDC97 binding site is composed of a short linear motifs (SLiMs), thus we did not proceed with further fragmentation. This conclusion was strengthened during revision, when we performed a mutational analysis of TTC33 (Fig. 7i; Supplementary Fig. 5c). The TTC33 mutants we designed were targeting residues proposed to participate in WDR61 or PHF5A binding (Fig. 7f,h; Fig. 8a, Supplementary Fig. 5b). We observed that when TTC33 association with WDR61 or PHF5A was affected, binding to CCDC97 and UNG1/2 was also decreased (Fig, 7i, Supplementary Fig. 5c). Mutations were localized in the TPR4 or immediately adjacent to it. Except for one, all mutants we designed had a low ΔΔG (Fig. 7h), indicating there is little chance of a major change to the proteins structure. We thus conclude that slight conformational changes to the TTC33 side-chains forced by the analyzed variants likely disrupt binding interfaces all over the TTC33 protein. This is supported by the notion that the only variant with a high ΔΔG, namely R135P, located in TPR4 middle part was the most efficient at disrupting PHF5A, WDR61, CCDC97, and UNG1/2 binding (substitution to proline often

disrupts the helix fold).

We produced a table that sums up both our fragmentations assays and mutational TTC33 analysis (Supplementary Fig. 6e). We also included a table that lists the ΔΔG values for the point mutants, that indicate the likelihood of a major impact on protein stability (Figure 7h).

4. The effect of H2O2 on wt or miTTC33 in Fig.3f seems very similar, considering the phenotype of untreated miTTC33-cells, making the point of a role for TTC33 in H2O2 response very weak.

We re-evaluated these data, which are now shown in revised Fig. 10f (scheme in Supplementary Fig. 9a). Given the reports by Bao et al., 2020 (*doi: 10.1016/j.freeradbiomed.2020.06.010*) and Liu et al., 2016 (*doi: 10.1016/j.freeradbiomed.2016.07.030*), which indicate that UNG1/2-depletion sensitized cells to ROS, we decided to replicate the data using our HEK293 cell line expressing micro-RNAs directed against UNG1/2. We also used a cell line over-expressing UNG2, as – at least in yeast – it was shown to cause cell cycle arrest (Elder et al., 2003: *doi: 10.1016/s0006-291x(03)01036-2*). We reasoned that this should inform us about the maximum phenotype that could be manifested in our experimental system.

In control conditions, the cell line depleted of UNG1/2 grew comparably to control HEK293 cells, while UNG2 over-expression decreased cell growth to a rate comparable to TTC33 or CCDC97 depletions (revised Fig. 10a). To evaluate the effect of exposure to hydrogen peroxide, we calculated the duration of the proliferative phase in hours in control conditions and in the recovery period after exposure to hydrogen peroxide. Here, the proliferative phase is understood as the time required for the cells to attain 50% confluence starting from 5%. In control cells exposure to hydrogen peroxide extended the duration of the proliferative phase by 10 hours on average. Either depletion of over-expression of TTC33 or UNG doubled this effect to roughly 20 hours. We believe that there is a sensitivity to hydrogen peroxide in cells depleted of TTC33 that is comparable to conditions in which UNG levels are altered.

In the revised manuscript we refined our interpretation of this result. Though UNG1 and UNG2 were suggested to somehow play a direct role in response to ROS, we clearly overstated the putative link to TTC33 in the process. ROS sensitivity is a feature of many cellular states when DNA repair is less efficient. We thus think that this result simply speaks as an extra argument that DNA repair is less efficient when TTC33 or CCDC97 are depleted, but it is not proof of a functional link with UNG1/2 in this process. We thank the Reviewer for toning down our conclusions.

Minor points:

1. Show the expression of GFP-TTC33 in cells by immunoblot. The bands shown in S1a-b are not enough. Same for WDR61.

Western blot analyses were added, as requested. Data can now be found in Supplementary Fig.1b-e, and Supplementary Fig. 2d,e.

2. Add a marker of nuclear fractionation (LaminA/C?) in Fig.S1g

A lamin A/C marker was added, as requested. The signal is clearly nuclear, as expected. The result can now be found in Fig. 3c.

3. What is the “control” in Fig.1h and i? No ab? No secondary ab? A better control would be after deletion/silencing (as shown in Fig.4). It is very strange that the IF for TTC33 shows such a clear nuclear localization, but for fractionation is more in the cytoplasm. The justification in the text is not consistent with the finding that CCDC97 is more in the nucleus but still it is seen in the cytoplasm.

The Reviewer raises an important question about the proportion of TTC33 and CCDC97 in each cellular compartment, namely the nucleus and cytoplasm. This comment led us to reconsider our data. In our view, the most quantitative result in the manuscript is the one assessing each protein localization by subcellular fractionation coupled to western blot analysis (now Fig. 3c). Here, the identity of the detected bands was tested as the Reviewer suggested by western blot analysis of cells knocked-down for TTC33 or CCDC97 (now Fig. 9a). The result indicated a mixed nucleo-cytoplasmic localization. The second best result is the protein localization by microscopy using the EGFP tag (now Fig. 3b). In case of CCDC97 a clear nucleo-

cytoplasmic signal was seen in fixed cells, which we also confirmed in live cells during revision. This supported the fractionation result. EGFP-tagged TTC33 seemed to display a more nuclear signal, but when the pictures were displayed in black-white setting instead of the green-black originally put forward, the cytoplasmic fraction became more apparent, also in live cells (now Fig. 3b). Given that the IF performed previously using protein-specific antibodies did not show any cytoplasmic fraction, we doubted the result specificity. Neither of the antibodies was recommended by the manufacturer for IF, and it is possible that off-targets are produced on heavily cross-linked material, such as formaldehyde fixed cells. Thus, in the revised version we limit this chapter to showing cell fractionation coupled to western blot and EGFP-tagged TTC33 and CCDC97 localization, and more firmly state the intracellular localization of both proteins, though still the nucleus is the most common compartment for all TAN factors.

4. Why are RNase/benzonase increasing the interaction between TTC33 and SF3B4? This doesn't show that the interaction is weaker as the author say, just that is inhibited (?) by DNA/RNA.

The intention of this experiment was to demonstrate whether the link between TTC33 and PHF5A-SF3B was mediated by RNA, because SF3B was shown to bind to RNA (Cretu et al., 2016: *doi: 10.1016/j.molcel.2016.08.036*). Since after treatment with nuclease there was no decline in interaction, but rather a minor increase, the response is negative and the link between TTC33 and PHF5A-SF3B is likely direct (protein-to-protein). However, given published results, we see how this minor increase in SF3B4 levels could have led to additional speculations. To increase the quality of the analysis, the experiment was since replicated in a different setting, using a MBP-TTC33 bait expressed in both HEK293 and HeLa cell line. We added RNase A treatment (and skipped benzonase since there was little difference between the two in our prior experiments), which did not influence the interaction between TTC33 and PHF5A or SF3B4 (see revised Fig. 2d). Similarly, this new data confirms that TTC33 association with other TAN factors appears not to be mediated by RNA.

If we understand the Reviewer's comment correctly, an underlying question is also raised regarding the relative strength/consistency of interaction between TTC33 and PHF5A-SF3B. We uphold our original statement that PHF5A was more strongly enriched in the high-throughput mass spectrometry data than most of the remaining subunits of the SF3B and associated SF3A complexes. In the revised version of the manuscript we produced two more mass spectrometry datasets aimed at identifying TTC33 interactors (MBP-TTC33 from HEK293, and TTC33-EGFP from HeLa, in addition to originally shown TTC33-EGFP from HEK293 or FreeStyle 293-F), and we re-analyzed the data to better justify our statement. The revised Supplementary Fig. 1f shows an extract of the novel Supplementary Dataset 2, in which a global analysis is performed for all 4 high-throughput datasets produced to assess the TTC33 interactome. Two sets of criteria were used to process data. The stringent require a protein to be among the 20% enriched in a given dataset and represented by at least 2 peptides, while the relaxed criteria ask a minimum 40% enrichment and one peptide. The relaxed criteria produced a list of 1334 proteins identified in either of the four datasets. Of those, 311 were identified using the stringent criteria. Out of the SF3A and SF3B complexes only PHF4A, SF3B5, and SF3B6 passed the stringent criteria and were consistently identified in all datasets. In contrast, other SF3A/B subunits were less consistently identified, though still mostly classified as enriched. Also, in our improved coIP-WB analysis in Fig. 2d, performed in HEK293 and HeLa, PHF5A is more consistently co-purified with MBP-TTC33 compared to SF3B4. Thus, our biochemical data support the conclusion that TTC33 is more stably associated with PHF5A compared to the remainder of SF3B complex.

5. The characterization of the TANC complex would benefit from showing more points after C7 in Fig.i (and Fig.S2C)

This has been corrected, as requested, and is now shown in Fig. 5c. The fractions past the C7, which were outside the peak of 280 nm signal, are essentially empty, indicating that neither of TANC subunits migrates as a monomer in SEC.

6. While the prediction of TTC33 structure is probably correct, I don't think it can be "validated" by predicting other structures of other proteins, as the author claim, especially when for these proteins the structure was already available in 2018, the latest data sets on which AlphaFold was trained. On the contrary,

the authors should show the Expected position error for all AlphaFold predictions (TTC3 and the TANC complex)

TANC complex quality control: As quality control for the TANC complex model, we now include the expected position error plot for the TANC complex (Fig. 6e) and indicate the pTM and ipTM values for model_0 (0.7 and 0.79 respectively; Fig. 6d), and for the 4 additional models proposed by AF3 (pTM range: 0.7-0.69, and ipTM range: 0.79-0.78; Supplementary Fig. 4d). We aligned five AlphaFold 3 TANC models and showed that modeling of the WDR61, PHF5A, and TTC33 TPR core were almost identical in five attempts (Supplementary Fig. 4c; RMSD ~0.302-0.355). The biggest discrepancies were observed around the TTC33 N- and C-termini, which were modeled at random. Additionally, we tested the AF3 TANC the best model_0 using the MolProbity suite and provide associated statistics (Supplementary Fig. 4e,f), and a Ramachandran plot, which indicates that 93.7% of residues were in favored and 99% in allowed positions (Fig. 6g).

The alignments of WDR61 and PHF5A AlphaFold 3 and experimental structures are of course no direct proof of good quality for the TANC complex model, but we believe that for most readers they will be a nice visual support for the quality plots we now included and listed above. We thus kept them and included RMSD values (0.821 for WDR61 and 0.452 for PHF5A; Fig. 6f).

TTC33 protein model: Originally, the TTC33 protein model was extracted from the complex, so the expected position error is already shown in Fig. 6e. To cross validate the TTC33 modeling in the context of a complex to solitary protein assessment, we now provide a structural alignment of this TTC33 model to the SwissModel structure, which was obtained using homology modeling (RMSD of 1.775; Fig. 6b). As mentioned above, the N- and C-termini of TTC33 are modeled at random with low AlphaFold 3 reliability score, and are very mobile in our molecular dynamics simulations (Figure 6i; Supplementary Fig. 4g-l), which was also true for the TTC33 solitary models. Therefore, including the position error for the full-length TTC33 AF3 model alone would be a redundancy in our view. We thus now performed an independent modeling of the human TTC33_TPR2-3-4 domain and adjacent regions (corresponding to residues 42-169), and its RMSD to the full-length TTC33 extracted from the TANC model is 0.332 (Fig. 6b,c). This modeling of TTC33_TPR domain displayed a high pTM score of 0.86. To complement this analysis, and test the evolutionary conservation of the core TPR domain, we also modeled those regions in 11 other vertebrate species, whose amino acid sequences were aligned to the human TTC33 in the Figure 1b, and analyzed for TPR presence in Fig. 1c. Vast majority of those structural models had an equally good pTM score of ~0.85-0.86. The RMSD of each vertebrate TTC33_TPR domain to the human model was within 0.165 to 0.502 (Fig. 6c). Altogether, this supports the notion that the central TTC33 TPR2-3-4 adopts a classical convex-concave TPR fold in human and other vertebrate species, while the N- and C-termini are unstructured.

7. What is the EGFP-TTC33_rec in Fig.S3h? And how is it different from the TTC33-EGFP? In the text is just called"variant with altered TAN factor binding efficiency", but it is just more expressed. Also, I don't see how it immunoprecipitated less with WDR61

EGFP-TTC33_rec is a construct that was codon-optimized using the ThermoFisher GeneArt service. Inclusion of only the most optimal codons improves translation and ultimately protein expression. In contrast, the TTC33-EGFP construct is encoded by the wild-type ORF. Furthermore, both constructs have tags attached to different ends, as indicates by their names. To clarify those differences, where appropriate, we corrected the name EGFP-TTC33_rec to EGFP-TTC33*_codon_optimized*.

Asking about WDR61 immunoprecipitation, the Reviewer refers to a coIP-WB experiment, in which EGFP-tagged protein derived from each construct was used as bait (now shown in Supplementary Fig. 6h) to compare association with WDR61, PHF5A, CCDC97, UNG1/2, and PPP2R2A. Equal amounts of whole cell extracts were used, but EGFP-TTC33 was robustly expressed from the codon-optimized construct, as opposed to the TTC33-EGFP, which is expressed at near-endogenous levels (this was discussed above in our response to Reviewer's 1 question 11, page 20, and documented in the Fig. 8f). As expected, the protein encoded by the optimized construct was more abundant in the elution. Given this, it is not astonishing that it precipitated more of PHF5A and CCDC97. However, compared to TTC33-EGFP, the more abundantly produced EGFP-TTC33*_codon_optimized* version associated with lower amounts of UNG1/2 and PPP2R2A. WDR61 levels are equal in the eluates, but compared to (or otherwise – normalized to) the bait amount, the

EGFP-TTC33 precipitated less WDR61 than TTC33-EGFP. Thus, both versions have an altered efficiency of association with various TAN factors, as was stated in the original (and is elaborated in the revised figure legend) version of the manuscript. We presume that this disparity might be due to different tag location or altered protein co-translational folding due to modified translation rate resulting from codon optimization. This experiment is now shown in Supplementary Fig. 6h.

8. Is a TTC33 mutant in H155 still able to bind WDR61 and/or PHF5A? And which is the exact region of TTC33 interacting with PHF5A? Can it be mutated and recapitulate the lack of interaction with WDR61?

TTC33:WDR61 interaction surface. During revision the H155 residue was mutated to alanine or tyrosine. The H155Y substitution corresponded to a somatic cancer variant COSV100531955. Both mutations decreased TTC33 association with WDR61 (see Figure 7h,i). The TTC33_H155 residue is conserved in 99% of the 300 assessed vertebrate species (see revised Fig. 1b, Supplementary Fig. 5b; Fig. 8a), strongly pointing to its functional significance.

Additionally, we further re-analyzed the TTC33:WDR61 interaction surface in the AlphaFold 3 model and identified 2 additional putative contacts: TTC33_H125:WDR61_W234 and TTC33_R148:WDR61_D17. We analyzed the stability of the contacts between residue pairs during the molecular dynamics simulation and discovered that the TTC33_R148:WDR61_D17 link was very stable, similarly to TTC33_H155:WDR61_W20, whereas the conformation of the TTC33_H125:WDR61_W234 pair fluctuated extensively. The TTC33_R148 residue is conserved in 96% of vertebrates, whereas TTC33_H125 only in 54%. In concordance, a TTC33 R148Q (somatic cancer variant: COSV61802519/rs1182763551) decreased WDR61 binding, whereas TTC33_H125 substitution did not alter WDR61 recruitment.

TTC33:PHF5A interaction surface. In the revised version we present the presumed mechanism of TTC33:PHF5A interaction in the AlphaFold 3 model, which is based on the interaction of the negatively charged TTC33 TPR domain concave with the positively charged PHF5A C-terminus (Fig. 7a,b). We also evaluate the surface evolution in a molecular dynamic simulation (Fig. 7e,f). We thus identify major contact points between TTC33 and PHF5A (*e.g.* TTC33 Q101, E97) and several less consistent ones. The interaction surface is tested by removal of PHF5A C-terminus (Fig. 7c), TTC33 fragmentation assay (Fig. 7g), and TTC33 mutational analysis (Fig. 7i, Supplementary Fig. 5c). Most mutations of TTC33 residues predicted to enter in contact with PHF5A led to a decrease in interaction, though it was never fully abrogated. This is not surprising to us, since many potentially compensatory contacts may be formed, because the interaction is mostly based on an association of negatively and positively charged surfaces (Fig. 7a).

Our revision work clearly refined the mechanism of TTC33:WDR61 interaction, and we are grateful for the Reviewer's suggestions. This data is now shown in revised Fig. 7, Fig. 8, and Supplementary Fig. 5b,c.

9. Is the PHF5A mutant in Fig.3m affecting the interaction between TTC33 and WDR61? Can it bind still SF3B? Also, please indicate the exact residues that are mutated as well as the exact residues of TTC33 involved in the binding.

Due to the issue with unequal amounts of the baits in the inputs presented in the figure included in the original submission, we have replicated the coIP-WB analysis of PHF5A full-length and C-terminal mutant, (revised Fig. 7c). The PHF5A mutant lacking the most C-terminal 9 amino acids cannot bind to TTC33, WDR61, CCDC97, and has a profoundly decreased binding efficiency to UNG1/2 and SF3B4. The deleted residues are now indicated in the Fig. 7a (RKKYGFKKR), and in the revised coIP-WB experiment the full length protein is named WT: 1-110 while the mutant is referred to as mut: 1-101. We believe that these changes should clarify the matter.

As requested, we now provide a close-up picture of the interaction surface between TTC33 and PHF5A with polar bonds (Fig. 7b), and map how these bonds evolve during a molecular dynamics simulation (Fig. 7e). This is recapitulated in a schematic shown in the Fig. 7f, and subjected to further analysis by TTC33 protein fragmentation (Fig. 7g) and TTC33 mutational analysis, which was replicated in two distinct cell lines, HEK293 and HeLa (Fig. 7h,i and Supplementary Fig. 5c).

10. Provide a quantification for the assay shown in Fig.S4a

As requested, we quantified the glycosylase assay results (now displayed in the Supplementary Figure 7c,d). As expected, uracil excision is inhibited after UNG1/2 knock-down, and accelerated after UNG1 or UNG2 over-expression. TTC33 or CCDC97 knock-down slightly delays uracil excision, whereas TTC33 over-expression accelerates the process. However, the changes are so subtle, that in our view are unlikely to be biologically relevant. This conclusion is rephrased in the revised version.

Comments to text and figures:
1. The manuscript is overall very difficult to read, going from one finding to the next one without explanation.
2. The introduction is very confusing. I understand that the authors want to introduce all the TTC33-interactors, however, the introduction is not organic, and I think that such introduction could be done when the interactors are described/characterized after isolation. On the contrary, the introduction lacks a general overview of the DNA damage response and its key factors, including p53. A broader introduction on TPR proteins would also benefit the understanding of the work.
3. When something is mentioned first time (i.e. benzonase) it should be introduced.
4. The conclusions are often said before the data (as for the Fig.3I)

Response to minor comments 1, 2, 3, and 4: As requested, we extensively re-wrote the Introduction, Results, and Discussion sections, as suggested by the Reviewer and Editor. Description of new data is marked in green, whereas reshuffled data or re-edited text elements are marked in blue. We hope that we considerably improved the clarity of the manuscript and the stylistics.

5. The alignment of the TTC33 proteins is informative, but the legend is confusing. I also recommend moving the schematic of human TTC33 with all the different TPR regions and the NLS in the first figure.

We followed the Reviewer's suggestions. Fig. 1a now shows a genomic alignment of *TTC33* locus from various representative vertebrate species. It is followed by a protein alignment from 12 species, and a graph showing amino acid conservation calculated from a protein sequence alignment issued from 300 vertebrate species (Fig. 1b). Finally, new Fig. 1c shows schematic mapping of TPR repeats and NLS in TTC33 proteins from 12 vertebrate species shown in Fig. 1b. The TPR repeats are color-coded according to likelihood (p-value). This novel graph shows that in all species there are likely 3 consecutive TPR repeats located in the middle part of TTC33, able to form a minimal TPR fold. Finally, where identified, NLS sequences are also mapped to each TTC33 protein in Fig. 1c.

6. The western blot in Fig.S3a is overexposed and it is difficult to see the levels of the TPR mutants and of CCD97.

The Reviewer refers to the TTC33 fragmentation assay, which was done using transiently-transfected FreeStyle 293-F cells. Here, each variant was expressed at different levels. In our procedure, we transfect the cells with equal amounts of plasmids, and protein expression from each construct is controlled by the same promoter. There is not much we can do about this imbalance in expression, because this is an offset of how efficiently the cells express each individual variant. We understand that this limits the quantitative interpretation of the result in case of CCDC97 or PPP2R2A, but does not preclude the rough identification of the WDR61- and PHF5A-binding region. We strongly believe that those limitations were mitigated by our novel TTC33 mutational analysis (see Fig. 7i, Supplementary Fig. 5c). Here, the expression of TTC33 mutants was comparable to the levels of the wild-type protein. Thus, we put more effort into this more detailed analysis, and assessed interactions with PHF5A, WDR61, UNG1/2, and CCDC97.

7. Where is the full length EGFP-PHF5A in the input of Fig.3m?

Indeed, in the experiment shown in the original version, the expression of the full-length EGFP-PHF5A was low, compared to the C-terminally truncated mutant. A new coIP-WB experiment was performed to fix this

issue. It is shown in revised Fig. 7c. Essentially, truncation of the PHF5A C-terminus reduces the protein's ability to associate with TTC33 and other TAN factors.

8. In Fig.4c the effect of doxorubicin in the IF is not clear (there is less p53-S15P signal than in the untreated cells). Also, I find contradictory that the authors mention the p53-S15P signal being “adjacent to the nucleus” (which means outside the nucleus) and co-localizing “with the centromere” (which are inside the nucleus as part of the chromosomes)

Since doxorubicin induces DNA double-strand breaks (Yang et al., 2015: *doi: 10.1016/j.mrfmmm.2015.01.007*), we used the drug to provide a positive control for our IF experiments. In the particular IF referred to by the Reviewer, doxorubicin was applied to the cells for only 30 min. Thus, the full extent of the effect on p53-S15P was not yet observed. During the revision we performed additional experiments (p53-S15P localization in HEK293 and A549 cells treated with siRNAs against TTC33; Supplementary Fig. 8c,d), where doxorubicin was applied for 2 hours. In those additional experiments the effect is more pronounced. However, even in the particular experiment inquired about by the Reviewer nuclear p53-S15P levels are increased, as indicated by the quantification (see Fig. 10c for the quantification of IF data shown in Supplementary Fig. 8b).

In HEK293 control cells, the p53-S15P antibody detected an aggregate neighboring the DAPI/Hoechst signal. Similar, but a more discreet signal, is also present in A549. Upon addition of doxorubicin, this aggregate disintegrates to give way to an evenly distributed nuclear signal. We do not know what is the identity of this aggregate in control cells, and we simply speculated that it might be a remnant of the centromere, because of the work by Contadini et al., 2019 (*doi: 10.1038/s41419-019-2076-1*). We see that this was too far-fetched, and thus removed this speculation from the revised manuscript.

RESPONSE TO REVIEWER 4

Reviewer #4 (Remarks to the Author):

This is a very interesting work combining computational, mass spectrometry and immunoprecipitation techniques for identifying the interacting partners of a previously uncharacterized protein: TTC33. The study is technically sound and the authors have an excellent experimental design validating their findings using a combination of techniques. There are several information however that are missing from the description of the methods, while the authors could further expand the computational analysis they conducted to infer more information about the functionality of the protein and its potential association with pathophysiological mechanisms.

We are thankful for the overall positive assessment of our work. During revision, and thanks to the various comments we received from all Reviewers, we believe that the quality of our work has improved significantly. For a better overview of this major revision in the preamble and Table 1 we sum up the various experiments and bioinformatic analyses performed.

We are particularly grateful to this Reviewer for the suggestion to perform a molecular dynamics simulation, which has greatly increased our understanding of the architecture of the TTC33:PHF5A and TTC33:WDR61 binding interfaces. Secondly, the sharp question about dbSNPs, and GWAS prompted us to re-evaluate publicly available clinical data. This, in conjunction with the molecular dynamics results, led us to perform a TTC33 mutational analysis, using population variants and somatic cancer variants. Additionally, we have improved on the quality of our TTC33 interactor analysis by producing additional datasets. Though this did not change the strict list of stable TTC33 interactors, it provides a resource to explore weaker potentially functionally relevant partners in the future.

-"Strikingly, only 11 proteins were common for both lists and only those likely represented bona fide TTC33 partners": The very low overlap of the interacting partners of TTC33 raises concerns for the accuracy of the MS workflow. To further increase confidence of the findings, the authors could try to calculate co-expression of TTC33 and its potential interacting partners in different tissues and conditions (using even publicly available data).

Mass spectrometry data: We think we phrased this section quite poorly. The overlap between raw datasets was much larger than those 11 proteins, but we applied stringent filtering conditions to ensure that only proteins strongly enriched and represented in most replicates were utilized for cross comparison. Preparation of samples for mass spectrometry analysis consisted of purifying protein complexes in native conditions, with no cross-linking applied. As a result, the stable TTC33 partners will be those that are the most enriched and consistently co-purifying. We do not exclude the possibility that transient or weak interactors, which are meaningful to TTC33 function, also co-purified, but their identification should be more random, compared to the stable interactors. Thus, the low number of overlapping partners in our mass spectrometry data was a result of the purification method combined with stringent filtering we applied (see below), which was clearly not indicative of any quality issue with the dataset itself; more so that the interactors could be validated by coIP coupled to western blot analyses (Fig. 2d). However, in response to Reviewer 3 remarks, during revision we increased the strength and reliability of our analysis by producing two more datasets. In sum, we assess TTC33 interactors in:

1) HEK293 TTC33-EGFP,
2) FreeStyle 293-F TTC33-EGFP,
3) HEK293 MBP-TTC33,
4) HeLa TTC33-EGFP.

We now present our analysis in a more transparent way in Supplementary Fig. 1f and Supplementary Dataset 2. In the revised Supplementary Dataset 2 we present each dataset separately with a log2_FC column indicating protein enrichment (column G). For each dataset, we produced two lists of significantly enriched proteins: *stringent* and *relaxed*. The *stringent criteria* were identical as previously stated: interactor had to be among the 20% most enriched proteins, represented by at least 2 peptides. The *relaxed criteria* required the

hit to be among the 40% most enriched proteins and represented by at least 1 peptide. We merged the list of interactors identified using the relaxed criteria obtaining in sum 1334 proteins. Of those, 311 were identified in either dataset as enriched using the stringent criteria. For those, we produced a gene ontology analysis, which is shown in the supplementary dataset (see overview below).

Next, we assigned 3 scores to each of the 1334 proteins obtained with the relaxed criteria. *Score_q80* assigned 1 point if the protein was detected in a given datasets using the stringent criteria, thus maximum score values was 4. *Score_q60* assigned 1 point if the protein was detected in a given datasets using the relaxed criteria, thus maximum score values was also 4. *Score_q60q80* is a sum of score_q60 and score_q80, producing a maximum value of 8. Factors with *score_q60q80* equal to 8 are essentially the TAN factors originally put forward in the first submission (TTC33, WDR61, PHF5A, CCDC97, UNG1/2, most PP2A-B55α subunits, and selected SF3B subunits). Score 7 complements the TAN list with missing PPP2R1A subunit of PP2-B55α, and all remaining SF3A/B proteins. This analysis is now provided in Supplementary Dataset 2 with an extract in Supplementary Fig. 1f, providing a resource for future exploration of weaker interactions.

Co-expression of TTC33 and its partners in various tissues: We profited from data produced by Fagerberg et al. (2014: *doi: 10.1074/mcp.M113.035600*), who assessed transcript expression in various human tissues. TTC33 and CCDC97 mRNA are present in all tissues, and both expressed at the same level (Fig. 3a). Other TAN factors: UNG1/2, PPP2R2A, WDR61, PHF5A, and SF3B4 are expressed at a 2-4-fold higher level. Altogether, all TAN factors are ubiquitously expressed.

-Are the interacting partners of TTC33 enriched in any pathway or GO term? This will lead to a better understanding of the functionality.

The strict list of TAN factors (SF3B-PHF5A, PP2A-B55α, WDR61, CCDC97, and UNG1/2) is too short to produce a gene ontology analysis. As mentioned above, a merged list of all significantly enriched proteins from either of the four datasets produced numerous GO groups, now listed in Supplementary Dataset 2. Groups with the lowest FDR were related to mRNA processing and splicing, mostly due to the presence of a complete set of SF3A and SF3B subunits, and other U2 subunits. However, other GO groups indicated cell cycle regulation, DNA repair, including double-strand break, and nucleotide metabolism. The DNA repair and DNA DSB repair is particularly interesting in the context of our functional analysis. As mentioned above perhaps exploration of the weaker interaction, in the context of follow-up works, will yield more insight into the TTC33 function, and we appreciate the Reviewer for pushing us to improve our analysis.

-Aligning the structural models of alphafold and swissmodel would further allow to understand their similarities and differences. Moreover the models should be further evaluated using Ramachandran plots and Molprobity analysis.

TTC33 TPR domain fold. We present a structural alignment of the TTC33 AlphaFold 3 model_0 and SwissModel, as backbone or backbone with side-chains. The RMSD is 1.755 (Fig.6b). We also performed AF3 modeling of TPR domains of human and 11 other vertebrate TTC33 proteins (residues 42-168 of the human protein; Fig. 6c). All of those sequences contained 3 adjacent, similarly located, TPR repeats (Fig. 1c). The pTM scores of those models ranged from 0.71 to 0.86, and all aligned with a RMSD of 0.165 to 0.502 to the human model. The human TPR domain model aligned at RMSD 0.332 with the full-length TTC33 extracted from the TANC model. Overall, this strengthens the notion that the fold of the TTC33 core TPR domain is conserved.

TANC structural model quality control. Using AF3, we generated 5 TANC models, which were very similar in the region of the TTC33 TPR domain core, PHF5A, and WDR61. All models aligned with a RMSD ranging from 0.302 to 0.355, and gave a high pTM (0.69-0.7) and ipTM (0.78-0.79) scores (Supplementary Fig. 4c,d). The key differences pertained to the TTC33 C-terminus, which is likely unstructured. As a quality analysis, we now show the position error plot (Fig. 6e). Following the Reviewer's suggestion, we used MolProbity to evaluate the TANC AlphaFold 3 model. The algorithm proposed slight adjustment to the TPR4 helix 2 and to part of the TTC33 C-terminus (Supplementary Fig. 4e). Nonetheless, a structural alignment of the original AF3 model to the MolProbity-corrected one gave a RMSD of ~0.000

(Supplementary Fig. 4e). A full report on the MolProbity analysis is given in Supplementary Fig. 4f. We display the Ramachandran plot of the TANC MolProbity corrected model in Fig. 6g (93.7% residues in favored, and 99% in allowed positions). The raw json files are deposited at Mendeley (*doi: 10.17632/p822vhnbdj.1*).

As for any *in silico* model, there is uncertainty about its accuracy, but we tried to mitigate it by extending the experimental validation of the model, which is presented in Fig. 6h, Fig. 7, and Fig. 8. This consisted in TTC33 fragmentation (previously shown), removal of PHF5A C-terminus (improved coIP-WB assessing more interactors), TTC33 mutational analysis (including using somatic cancer mutations and population variants), and mutation of WDR61 (previously shown).

-The authors do not provide RMSD values for the alignments.

This was corrected, we now indicate the RMSD values in the Figure panels, where structural alignments are presented.

-The process for selecting templates for homology modeling using swissmodel was not explained anywhere in the manuscript.

SwissModel is an automated database allowing for homology modeling. It uses experimental structures deposited at PDB as templates. In case of the human TTC33, a protein composed of a consensus of 8 TPR motifs (pdb code: 8chy) was used as template for SwissModeling. To our taste, this is the most unbiased approach. We clarify this in the Figure caption.

-The revealed protein complex should be also verified and analyzed with docking methods (such as using the alphafold-multimer tool).

Again, this was a useful suggestion indeed. We used the GalaxyWeb server (Ko et al., 2012: *doi: 10.1093/nar/gks493*) to perform molecular docking of a peptide corresponding to the PHF5A C-terminal 9 amino acids into the TTC33 structural model produced by AF3. We examined the 10 models proposed by the algorithm and in each of them the peptide was docked into the TTC33 concave at the same region chosen by AlphaFold 3 as the docking site. This strengthens the AF3 modeling and is now presented in the Fig.7d.

In addition, we have improved on the experimental validation of the TTC33:PHF5A interface. Compared to the wild-type protein, the EGFP-tagged PHF5A mutant lacking the terminal 9 amino acids does not interact with TTC33, WDR61, and CCDC97, and has decreased interaction with UNG1/2 (Fig. 7c).

We also performed a mutational analysis of TTC33. The selection of residues to be mutated was performed based on the original AF3 model and its evolution during a molecular dynamics simulation (Fig. 7b,e, see response below for detail). We mapped the interactions (Fig. 7f), and introduced mutations to glycine, alanine, or – where possible – population variants and somatic cancer mutations. The latter are listed in Fig. 7h, along with a ΔΔG score. All but one mutations used had a low ΔΔG score, which suggested little influence on TTC33 structure. The key residue was Q101, which according to the AF3 model binds to PHF5A K108. In addition, we validated several other residues contributing to the interaction (E97, R135, E162, and E166); (Fig. 7i and Supplementary Figure 5c).

-Molecular dynamics simulations are missing for both the TTC33 as well as the studied protein complex.

As suggested by the Reviewer, we performed a Molecular Dynamics simulation using the TANC complex. We are particularly grateful for this suggestion as it was extremely helpful for identifying the residues relevant to formation of the TTC33:WDR61 and TTC33:PHF5A interfaces.

The trimer remained stably associated throughout the simulation (Fig. 6i and Supplementary Fig. 4g-l), which was consistent with the results of our size exclusion chromatography analysis (Fig. 5c). However, the subunit interfaces underwent considerable remodeling. We mapped the interactions of TTC33 with PHF5A and TTC33 with WDR61 in the original AF3 model and during the molecular dynamics simulation (Fig. 7e,f, and Fig. 8a,c). We also tied the structural and molecular dynamics simulations to an assessment of evolutionary conservation of particular residues (Fig. 1b, Fig. 7f, and Fig. 8a). Finally, we selected population variants, or somatic cancer mutations in TTC33 to assess their impact on binding to PHF5A,

WDR61, UNG1/2, and CCDC97 (Fig. 7h). If no such variants were reported, we introduced alanine or glycine. With one exception, mutations with low ΔΔG score were used to ensure as little structural change to TTC33 as possible. Overall, mutations (including population/cancer SNPs) that strongly affected either WDR61 or PHF5A binding also decreased UNG1/2 association. This not only validated the model, but also demonstrated the nefarious impact of the SNPs on the cellular proteome. This novel data is aggregated in Figures 7 and 8.

-"The MaxQuant protein intensity data was further analyzed using Rstudio (Version 1.2.5033). ": A github link for the analysis would further increase its transparency.

In Mendeley Data (*doi: 10.17632/p822vhnbdj.1*) and PRIDE repository (accession numbers: PXD059336, PXD059337 and PXD06686), we provide the raw data tables, issued from the MaxQuant analysis of our mass spectrometry results. In Mendeley data we deposited text files with the code written for the analysis. Two versions are given: with filtering out hits not detected in bait samples (that was suggested by the Reviewer and is described in one of the responses to the question below) and without filtering.

-"To balance samples in each experiment, the data was normalized. ": What type of normalization was applied? Total-ion-intensity based or something else?

After preliminary data filtration (see response to next question), raw intensities of particular hits/protein in each sample were normalized to the sum of intensities of all proteins identified. This is specified in Methods section.

-"The missing values were replaced by randomized data,which was at least one order of magnitude lower than the weakest hit in each sample.": This is ok but it should be coupled with a filtering approach to filter out proteins with too many missing values.

We followed the Reviewer's suggestion, which has greatly improved the quality of the analysis. In the revised version, prior to further analysis, we removed most hits that were identified predominantly in the negative controls and not detected in the bait IPs, as those were considered contaminants not interacting with proteins of interest. The imputations were performed as previously, but much lower number of values needed replacing. The median imputation in negative control was 16%, as compared to 4% for samples containing baits (see imputation report in Supplementary Dataset 2). This filtering did not alter the final conclusion, but did improve the visual aspect of the graphs. Both the filtered and unfiltered analysis is deposited in Mendeley Data.

-Despite the importance of the work the authors do not emphasize on the impact of identifying the interacting partners of TTC33 (**RESPONSE A**)? Are there dbSNP or GWAS hits associating it with any disease (**RESPONSE B**)?

RESPONSE A. What is the relevance of TTC33 partners to the protein function?

We strongly believe that TTC33 function consists in modulating the function of one of its better characterized partners. Throughout our work we tested this concept of function by proximity. TTC33 interactors can be classified as having roles in either RNA metabolism, as in case of WDR61, PHF5A-SF3B, or DNA damage repair, which is mediated by UNG1/2 and PPP2R2A.

In response to Reviewer's 3 question, **we assessed TTC33 function in RNA metabolism by performing transcriptomic analyses of cells depleted of TTC33 or CCDC97. This did not reveal any strong, nor consistent phenotypes, discouraging us from further investigations** (*see response to Reviewer 3 → page 25-26*).

During revision we defined a specific role of WDR61 in the TANC complex. We observed that knock-down of WDR61 led to a decrease in TTC33 protein levels (Fig. 8g), which could be rescued by addition of a proteasomal inhibitor MG132. We further showed that a decrease in TTC33 protein levels in WDR61 knocked-down cells could be rescued by ectopic expression of a wild-type EGFP-WDR61 encoded by a mRNA insensitive to microRNA, but not by the W20C mutant incapable of binding to TTC33 (Fig. 8h).

Since our proteomic study on WDR61 WT and W20C mutant led us to conclude that WDR61 likely does not mediate any interaction with other TAN factors (Fig. 8d,e), **we conclude that WDR61 role is limited to specifically stabilize the TANC complex, by protecting TTC33 protein from proteasomal decay**.

Given the so far negative outcome of testing TTC33 role in RNA metabolism, we proceeded with evaluating the role in DNA damage. In the original submission we asked if there were any phenotypical similarities between TTC33 and UNG1/2. Depletion of both proteins (Fig. 9a) leads to increased genomic DNA fragmentation, which was tested using a comet test (Fig. 9b in the revised manuscript). TTC33- or CCDC97-knocked-down cells are also sensitive to reactive oxygen species (assessed by a proliferative assay; Fig. 10f). This sensitivity is qualitatively similar to effects obtained using UNG1/2 depletion or UNG2 over-expression. Despite those phenotypical links, our *in vitro* glycosylase assays show that uracil excision is not strongly affected in cells depleted for TTC33 or CCDC97 (Supplementary Fig. 7c,d). Thus, DNA double-strand breaks in TTC33-depleted cells arise from a different mechanism. The PP2A-B55α phosphatase was shown to play a role in DNA DSB resolution upon irradiation (Kalev et al., 2012: *doi: 10.1158/0008-5472.CAN-12-1667*). That is why in the revised version we inquired more deeply about this phenotype (see Fig. 9c,d,e and Supplementary Fig. 7a,b). The *in vivo* marker of DNA DSB is nuclear accumulation of γH2AX, and using immunofluorescence we confirmed that depletion of PPP2R2A leads to increased nuclear levels of γH2AX (Supplementary Fig. 7a). We also showed increased levels of this histone variant phosphorylation in HEK293 cells knocked-down for TTC33 or CCDC97 using microRNAs (Fig. 9c), and also in HEK293 cells lines in which genes encoding both proteins were separately stably deleted using CRISPR-Cas9 (Fig. 9d). Furthermore, cells knocked-down for WDR61 shared this phenotype, which could be rescued by expression of a wild-type WDR61, but not by the W20C mutant, that cannot bind to TTC33 (Fig. 9e). **Thus, the main phenotype associated with impairment of most TAN factors is related to accumulation of DNA DSBs and manifested by increased nuclear γH2AX levels.** This points towards increased genomic stress, which likely explains the decreased proliferation of HEK293 cells knocked down for TTC33 or CCDC97 using microRNAs (Fig. 10a). **In the revised manuscript, we show that depletion of either TTC33 or CCDC97 leads to an increase in the fraction of cells in G2 and concomitant decrease in G1, indicating cell cycle arrest** (Fig. 10d)**. This resonates perfectly with the accumulation of p53-S15P, reported by us in the original submission** (now presented in the Figure 10c and Supplementary Figure 8b,c,d)**.** Despite lower proliferation, TTC33- or CCDC97-depleted cells have increased BrdU incorporation (Fig. 10e). This is a marker of ongoing DNA synthesis, which – put together with decreased growth – can only be explained by DNA synthesis related to DNA repair. Importantly, we now show that the proliferative defect of TTC33-depleted cells can be replicated (and is more apparent) in cancer-derived cell lines, namely HeLa, A549, and HCT116 (Fig. 10b).

In sum, we believe that TTC33 partners are vital to the protein's function, and that this function is related to genomic integrity maintenance. Given our own transcriptomic data, a link to RNA metabolism is unclear, and as such the presence of PHF5A and associated SF3B among TTC33 partners might seem puzzling. However, PHF5A also plays roles related to DNA repair, as together with the p400 chaperone, it mediates deposition of γH2AX during antibody class switch recombination, and promotes non-homologous end joining (Begum et al., 2021: *doi: 10.15252/embj.2020106393*). Thus, PHF5A functions clearly are not limited to splicing.

RESPONSE B. Are there dbSNP or GWAS hits associating it with any disease?

In the revised work we address this question by:

- analyzing publicly available clinical data from The Cancer Genome Atlas. We found that in kidney clear cell adenocarcinoma (KIRC), the abundance of TTC33 mRNA is decreasing with cancer stage. Higher expression of all TAN factors (TTC33, CCDC97, WDR61, PHF5A, PPP2R2A, and UNG1/2) is associated with roughly 2-fold increased chances of overall survival. High expression of TTC33 or PHF5A is also associated with increased chances of 5-year survival. (Fig. 10g,h; Supplementary Fig. 9b; Supplementary Dataset 4).

- producing a mutational analysis of TTC33. Mutations that we tested targeted sites relevant to WDR61 or PHF5A binding to provide a much needed validation of the model. However, instead of replacing all selected amino acids with standard alanine or glycine, we also introduced population variants and somatic cancer

mutations (Fig. 7h; Supplementary Fig. 5b). We found that mutations strongly affecting WDR61 or PHF5A binding also lead to a decrease in UNG1/2 and CCDC97 association. All but one mutation had a low $\Delta\Delta G$ score, indicating that major changes to protein structure are unlikely. Yet, the impact on the TTC33-associated interactome was strong. Given that for most patients cancer is a polygenic disease, we think our results mark the tested variants as potentially harmful. These data are now shown in Fig. 7f,h,i and Supplementary Fig. 5b,c.

POINT-BY-POINT RESPONSE

Revision round 2

***

TTC33 forms a complex with WDR61 and PHF5A to recruit factors relevant to genomic integrity

Rafał Tomecki[1,2,#], Małgorzata Drabko[1,#], Małgorzata Siek[1], Maria Matykiewicz[1], Miłosz Ludwinek[1,a], Łukasz S. Borowski[3,4], Aneta Jurkiewicz[3], Kamil Kobyłecki[1], Agnieszka Jabłońska[1], Dominik Cysewski[5,b], Agata Malinowska[5], Magdalena Bakun[5], Rafał Płoski[6], Roman J. Szczęsny[3], and Agnieszka Tudek[1,7,*]

[1]Laboratory of RNA Processing and Decay, Institute of Biochemistry and Biophysics, Polish Academy of Sciences, A. Pawińskiego 5A, 02-106 Warsaw, Poland

[2]Laboratory of Epitranscriptomics, Institute of Bioengineering, Faculty of Biology, Biological and Chemical Research Centre, University of Warsaw, S. Banacha 2c, 02-097 Warsaw, Poland

[3]Laboratory of RNA Biology, Institute of Biochemistry and Biophysics, Polish Academy of Sciences, A. Pawińskiego 5A, 02-106 Warsaw, Poland

[4]Institute of Genetics and Biotechnology, Faculty of Biology, University of Warsaw, A. Pawińskiego 5A, 02-106 Warsaw, Poland

[5]Mass Spectrometry Laboratory, Institute of Biochemistry and Biophysics, Polish Academy of Sciences, A. Pawińskiego 5A, 02-106 Warsaw, Poland

[6]Department of Medical Genetics, Medical University of Warsaw, A. Pawińskiego 3c, 02-106 Warsaw, Poland

[a]present address: Chair and Department of Biochemistry, Medical University of Warsaw, S. Banacha 1, 02-097 Warsaw, Poland

[b]present address: Medical University of Białystok, Clinical Research Centre, J. Kilińskiego 1, 15-089 Białystok, Poland

#These authors contributed equally

[7]Lead contact

*Correspondence should be addressed to: atudek@ibb.waw.pl

***

TABLE OF CONTENTS

PREAMBLE..page 02

LIST OF EXPERIMENTS AND ANALYSES
ADDED IN THE SECOND REVISION ROUND.....page 03

RESPONSE TO REVIEWER 1.................................page 04

RESPONSE TO REVIEWER 2.................................page 05

RESPONSE TO REVIEWER 3.................................page 06

RESPONSE TO REVIEWER 4.................................page 23

EDITORIAL CHANGES TO FIT
FORMATTING REQUIREMENTS...........................page 28

PREAMBLE

We would like to thank the Reviewers for the effort put into revising our work. The expertise provided has helped us to substantially improve our work.

As a result of this second round of revision, we performed additional analyses and experiments. The major additions are listed in the next section. The portions of the main text related to those novel data are marked in the main text in **red**.

Due to Nature Communications formatting requirements, we were forced to shorten the text by roughly 400 words. We had carefully edited several portions of the text to ensure the message remains intact. The list of such changes is given in the last section of this point-by-point response. In the main text, those sections are marked in **blue**.

LIST OF EXPERIMENTS AND ANALYSES ADDED IN THE SECOND REVISION ROUND

1. **Correlation matrixes of tissue expression of TTC33 and its associated proteins identified by LF-MS with distinction between strict TAN factors and other putative interactors**

    Fig. 3b,c; Supplementary Fig. 2a

2. **γH2AX immunofluorescence in control and cells over-expressing TTC33**

    Supplementary Fig. 7b

3. **Assessment of survival capacity under doxorubicin for cells knocked-down of and over-expressing TTC33**

    Fig. 10f,g; Supplementary Fig. 9c,d; raw data added to Supplementary Dataset 3

4. **Quality report on template structures for Swiss-Model analysis of TTC33 with structural alignment to AlphaFold3 model**

    Supplementary Table 1

RESPONSE TO REVIEWER 1

The authors have sufficiently addressed the questions raised by the reviewers.

We thank the Reviewer for the insightful suggestions that greatly improved our work and for recognizing the effort we put into the revision.

RESPONSE TO REVIEWER 2

Reviewer #2 (Remarks to the Author):

I co-reviewed this manuscript with one of the reviewers who provided the listed reports. This is part of the Nature Communications initiative to facilitate training in peer review and to provide appropriate recognition for Early Career Researchers who co-review manuscripts.

We thank the Reviewer for the time and effort put into the revision, and for the valuable remarks that helped improve our work.

RESPONSE TO REVIEWER 3

In the revised manuscript, Tudek and colleagues addressed most of my comments adding numerous new/repeated experiments, defining in details the interaction between TTC33 and WDR61/PHF5, as well as their role in preserving genome stability. They also reorganize the data improving clarity.

We thank the Reviewer for recognizing the effort invested in the revision. We would also like to emphasize that the Reviewer's comments prompted us to substantially improve the quality and clarity of our work.

I still have a concern on the identification of TTC33/WDR61/PHF5 as involved in genome integrity "surveillance" (line 40) or "DNA repair" (line 374, line 443). The extensive data provided by the authors hints to a role for TTC33 in genome integrity, but more in maintenance-preventing damage than repair after damage. For example, the comet assay detects increased breaks after TTC33 depletion, but the depletion of many repair factors does not really increase the comet, unless cells are treated with DNA damaging agents before (RESPONSE 1). Data on cancer do not really correlate with DNA damage neither (RESPONSE 2). Furthermore, hydrogen peroxide cause general cell damage, not only at the DNA, and the delay in recovery could be due to many different issues. A DSBs induced drug as Neocarzinostatin or IR radiation would be more appropriate to test DNA damage response/repair (RESPONSE 3). Importantly, reduction in growth is not observed in UNG1/2 knock down, although there is an increased in p53 phosphorylation, while reduced growth is observed only when UNG1/2 is overexpressed, weakening the suggested similarities between TTC33 and UNG1/2 in repair (lines 437-439 and lines 449-453) (RESPONSE 4). Finally, a reduction in growth is also observed after TTC33 overexpression not only after TTC33 depletion. Does also overexpression increase H2AX and/or p53 foci? (RESPONSE 5) And how the authors interpret these phenotypes? While I agree with the authors that the work is mainly biochemical, and it is relevant for the identification of this novel complex, while further work should be addressing how TTC33 could participate in genome integrity maintenance, I strongly recommend toning down some conclusions and maintain a general "genome instability" prospective instead than DNA repair, keeping the discussion more open, also on the indirect role and the discrepancies.

The Reviewer asks many interesting functional questions. Below we separated the answers to each question marked in the original remark above.

RESPONSE 1: ('*I still have a concern on the identification of TTC33/WDR61/PHF5 as involved in genome integrity "surveillance" (line 40) or "DNA repair" (line 374, line 443). The extensive data provided by the authors hints to a role for TTC33 in genome integrity, but more in maintenance-preventing damage than repair after damage. For example, the comet assay detects increased breaks after TTC33 depletion, but the depletion of many repair factors does not really increase the comet, unless cells are treated with DNA damaging agents before***')**

Yes, we agree with the Reviewer about the result interpretation. At this stage we cannot be sure of the exact TAN function, and whether the DNA damage observed in TAN-deficient cells is a direct consequence or a side effect. We have discussed this in the final paragraph of the discussion, but some unnecessarily stronger remarks were scattered throughout the draft. In the revised version we improved the wording, and we list the introduced changes in the table below. Text marked in red was added during 2nd revision round.

Line:	Action	Original quote	Revised quote
Line 40 (part of the ABSTRACT)	the conclusion was rephrased in line with the Reviewer's suggestion	*Loss of TAN subunits leads to increased DNA fragmentation and in vivo accumulation of DNA damage response markers: γH2AXP and p53-S15P, linking TAN to genome integrity surveillance.*	*Loss of TAN subunits results in genomic instability manifested by increased DNA fragmentation and in vivo accumulation of DNA damage response markers: γH2AXP and p53-S15P.*
Line 374 (part of the RESULTS)	the text was revised to increase the precision of the	*While the proliferative phase of control cells was extended by 11 hours due to ROS, it was twice as*	*While the proliferative phase of control cells was extended by 11 hours due to ROS, it was twice as*

	conclusions	*long for cells knocked-down or over-expressing either TTC33 or UNG1/2 (Fig. 10f), confirming that altered levels of either protein impair DNA repair, and sensitize cells to genotoxic agents.*	*long as for cells knocked-down or over-expressing either TTC33 or UNG1/2 (Supplementary Fig. 9a,b). Thus, either condition reduced tolerance to ROS, likely because it exacerbated the genomic instability already induced by alterations in TTC33 or UNG1/2 abundance.*
Line 443 (part of DISCUSSION)	claims were toned down to avoid claiming a direct role in DNA repair	*Thus, in general, TAN-depleted cells are impaired at DNA DSB repair.*	*Thus, in general, TAN-depleted cells display genomic instability, but the source of the damage is unclear.*

RESPONSE 2: ('*Data on cancer do not really correlate with DNA damage neither.*')

The TCGA analysis was included to explore potential clinical contexts in which altered TAN factor expression may be relevant, and this was done at the explicit request of Reviewer 4. The analysis pinpointed KIRC as the sole cancer in which a clear association was found for most TAN factors. This was rather interesting, as most of our biochemical and functional work was done on non-cancerous, albeit kidney-derived, cells.

Since we could not determine whether TTC33 impacts DNA repair directly, we have carefully edited the manuscript to underline its relevance to genomic integrity, instead of directly suggesting a role in DNA repair (as listed in response to the first question above).

That said, we have two remarks:

1. Mutations in *bona-fide* DNA repair enzymes do not necessarily direct all cell types equally towards carcinogenesis, as various tissues predominantly deal with different damaging agents (Sun et al., (2019: doi: 10.20892/j.issn.2095-3941.2019.0097), e.g. NER mutations worsen skin cancer risk, while BRCA1 sensitizes to breast and ovarian cancer partially due to high estrogen metabolism in those cells.
2. We observed that, in HEK293 cells, TAN factor deficiencies mildly increase DNA damage, as indicated by elevated γH2AX and p53-S15P levels comparable to those induced by an early, yet nonlethal, exposure to doxorubicin. Because prolonged genomic instability is a known driver of mutagenesis and carcinogenesis, we believe that the clinical association observed in TCGA data may prove consequential and therefore prefer to retain the TCGA analysis in the manuscript.

RESPONSE 3: ('*Furthermore, hydrogen peroxide cause general cell damage, not only at the DNA, and the delay in recovery could be due to many different issues. A DSBs induced drug as Neocarzinostatin or IR radiation would be more appropriate to test DNA damage response/repair.*')

We appreciate the Reviewer's suggestion as it gave us a clearer insight into the phenotypes associated with alterations in TTC33 levels.

Unfortunately, IR radiation is technically unavailable to us. On the other hand, doxorubicin is an established chemotherapeutic known to specifically induce DNA DSBs by inhibiting topoisomerase function (Yang et al., 2015: doi: 10.1016/j.mrfmmm.2015.01.007). We systematically and reproducibly used it as a positive control inducing DNA damage in our immunofluorescence assays, thus to answer this question, it was a suitable agent, likely equivalent to Neocarzinostatin.

To assess the impact of doxorubicin on proliferation and survival, cells were exposed to a range of concentrations. At 0.5 µM, cells failed to proliferate and gradually lost confluence, indicating cell death. At 0.1 and 0.05 µM, cells proliferated to a maximum confluence before gradually declining. In control HEK293 cells, 0.025 and 0.01 µM allowed proliferation similar to untreated cultures. **Overall, the response to**

doxorubicin strongly depended on its concentration. Representative data are shown in Fig. 10f, Supplementary Fig. 9c, and Supplementary Dataset 3.

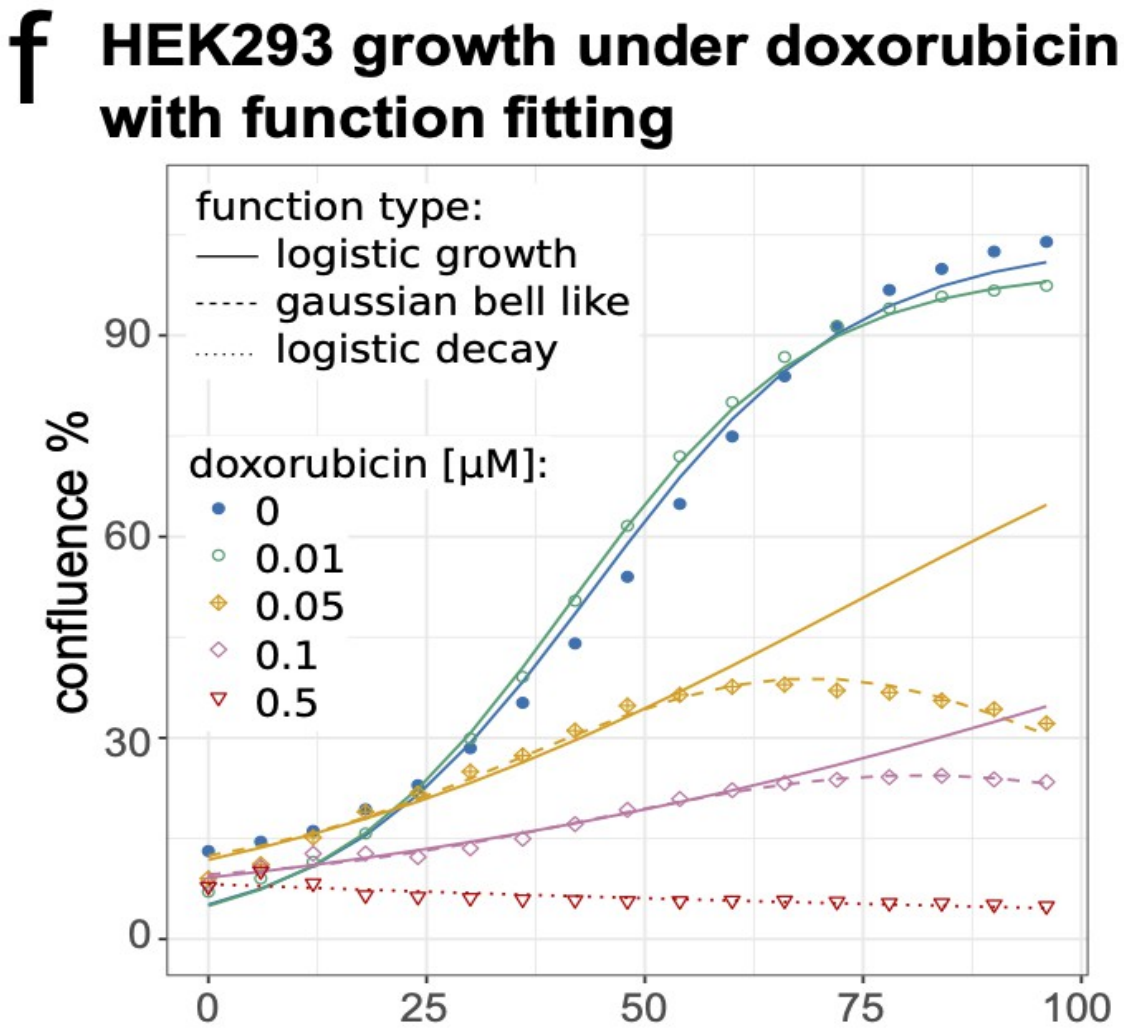

Figure 10. f *Points depict experimental % of confluence data for HEK293 cells grown in control conditions or in the presence of 0.01, 0.05, 0.1, or 0.5 µM doxorubicin. Lines represent the logistic growth/decay or Gaussian bell-like functions fitted into the raw data. Briefly, control cells and those exposed to 0.01 or 0.025 µM doxorubicin were modeled using logistic growth. Those at 0.5 µM more accurately followed logistic decay. The intermediate concentrations of 0.1 and 0.05 µM produced curves characteristic of initial growth and then population death. The initial growth phase (until the 65th hour) was modeled with logistic growth, while the full experimental growth was described by a Gaussian bell-like distribution (properties of this distribution are explained in Supplementary Fig. 9c). Exemplary raw data for other conditions is shown in Supplementary Fig. 9c.*

To quantitatively compare data between cell lines and replicates, we modeled growth patterns. Proliferation in **control cells and in those treated with 0.01 and 0.025 µM** doxorubicin was modeled using **logistic growth curve** fitting, as was done previously in our work. **Cell death** induced by 0.5 µM concentration was modeled using **logistic decay**. **The intermediate concentrations of 0.1 and 0.05 µM** were modeled using the **logistic growth in the first proliferative phase** (the initial 65 hours of growth), **and Gaussian bell-like distribution**, which was more suited for the complete growth data (proliferation-peak-death). All thus obtained function parameters are listed in revised Supplementary Dataset 3. Exemplary raw data with fitted functions are presented in Fig. 10f, Supplementary Fig. 9c. **This approach allowed us to compare the effects of TTC33 knock-down or over-expression to similar manipulations of UNG2 levels over 6-10 biological replicates.**

Due to differences in growth rates between cell lines (shown in Fig. 10a) **we normalized the changes in logistic growth rate under doxorubicin to untreated conditions** (doxorubicin exposure vs. control proliferation rate for each biological replicate separately).

At low doxorubicin levels (0.01µM), which did not strongly affect control HEK293 growth, the proliferation of all tested cell lines (TTC33, UNG1/2 knock-down, and over-expression of EGFP-UNG2 and EGFP-TTC33_*codon_optimized*) was markedly decreased. This trend was maintained in higher, cytotoxic, concentrations for TTC33 and UNG1/2 knock-down and EGFP-UNG2 over-expression (Supplementary Fig. 9e).

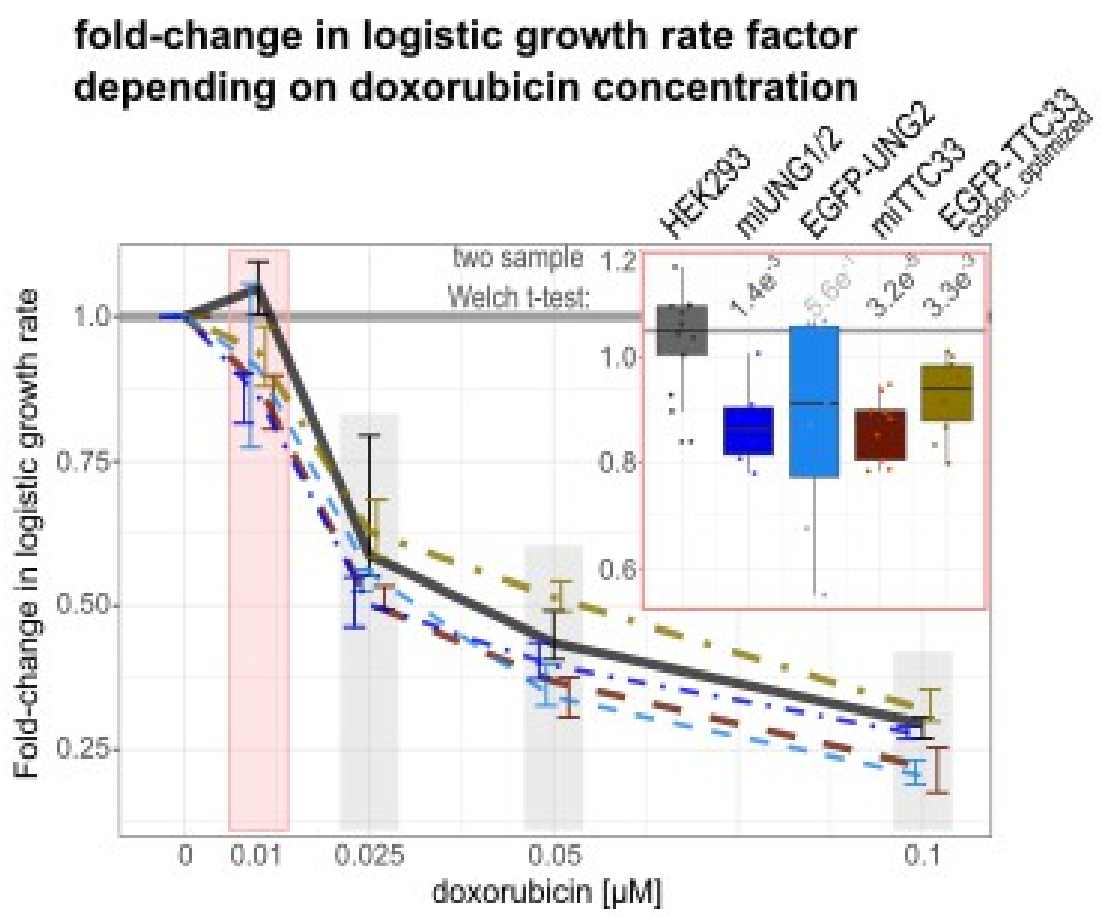

Supplementary Figure 9. e *As lines the graph shows changes to the median fold-change in the logistic growth rates depending on the doxorubicin concentration for HEK293 control, and cells depleted of or over-expressing TTC33 or UNG2. Error bars represent the position of the 25th and 75th distribution quantile. The raw values are listed in Supplementary Dataset 3. The bar plot in the top right corner zooms into the fold-change distribution at 0.01 µM doxorubicin concentration. The single points designate biological replicates. The fold-change was calculated separately for each biological replicate.*

The use of the Gaussian bell-like distribution allowed for assessment of cytostatic effects of doxorubicin (0.05 and 0.1 µM). The function has three main parameters, which are now compared between cell lines on Fig. 10g.:

- A – designates maximum confluence,
- sigma – controls the spread of the curve,
- mu (µ) that indicates the time required to reach maximum confluence.

We observed that **knock-down of UNG1/2 or TTC33 and over-expression of UNG2 decreased the overall proliferation time (mu) and proliferation rate** (also shown using the logistic fit → Fig. S9c), **resulting in decreased maximum population size (max. confluence). This can be interpreted as an increased sensitivity to doxorubicin.**

In contrast, **cells over-expressing EGFP-TTC33_*codon_optimized* behaved differently under cytostatic doxorubicin**. Even though the **maximum confluence attained was nearly identical to that of control cells**, this cell line **attained it much later (higher sigma and mu)**. Because the maximum confluence attained by EGFP-TTC33_*co* cells never greatly surpassed that of HEK293 control, we cannot propose doxorubicin resistance. This phenotype is rather symptomatic of **cell adaptation**.

This analysis is now displayed in Fig. 10 g. Also, for the convenience of the Reviewer, we provide an insight into replicate 13 raw data. In this final experiment we let the cells proliferate for 6 days to fully appreciate the control and EGFP-TTC33_*codon_optimized* growth curves.

Figure 10g

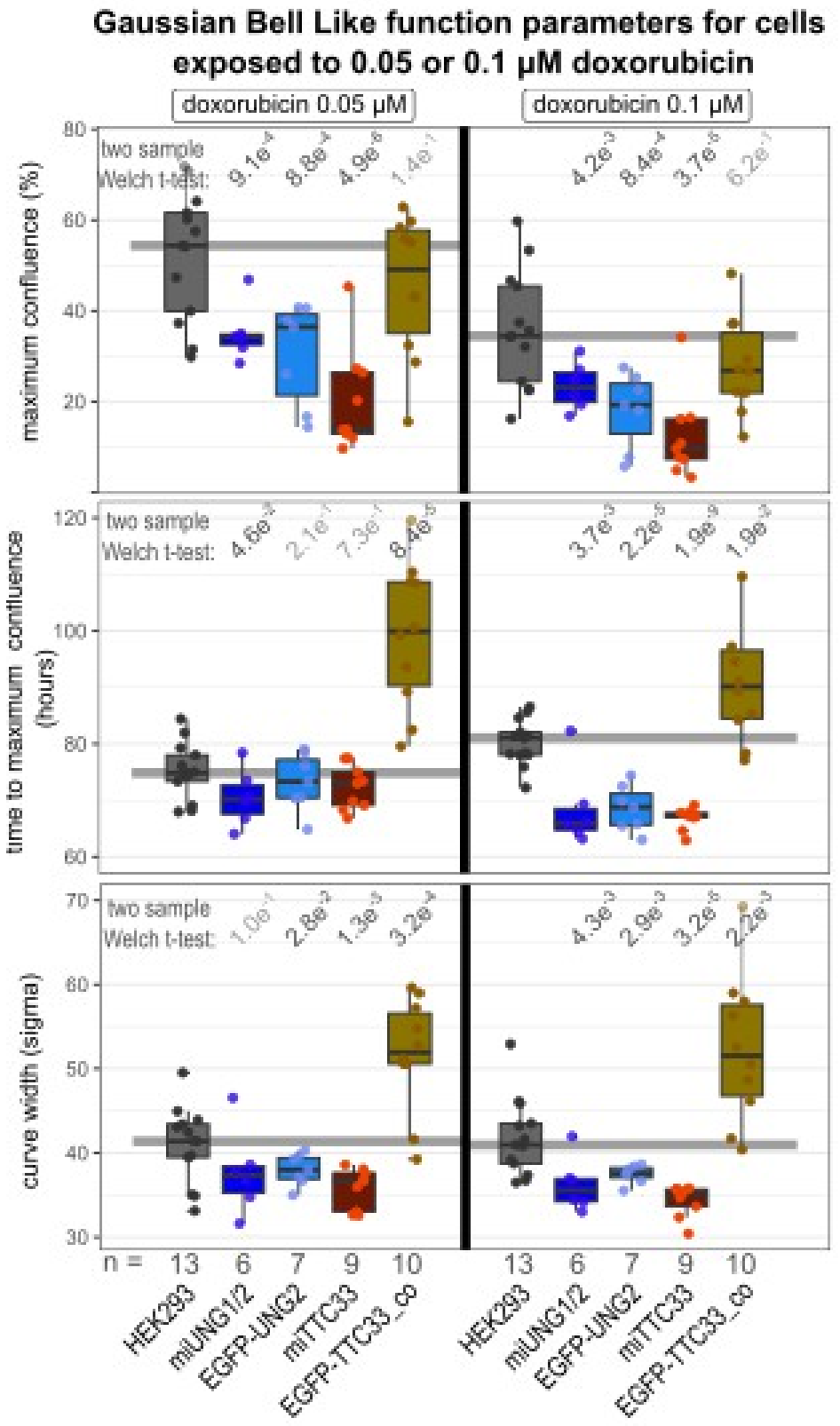

Figure 10. ***g*** *Box plot shows the distribution of the Gaussian bell-like parameters describing growth of cells under 0.1 and 0.05 µM doxorubicin. The centre of the box displays the median, while the edges the 25th and 75th distribution quantile. Raw data values are presented as dots. A two-sample Welch t-test result is listed above the graph.*

Raw data panel doxorubicin:
Plots show raw data from biological replicate 13. Error bars arise from technical replicates (4 per conditions). Cells were grown for 6 days to examine more closely the growth profile of EGFP-TTC33_*codon_optimized* in comparison to HEK293 control and UNG1/2 knock-down or EGFP-UNG2 over-expression.

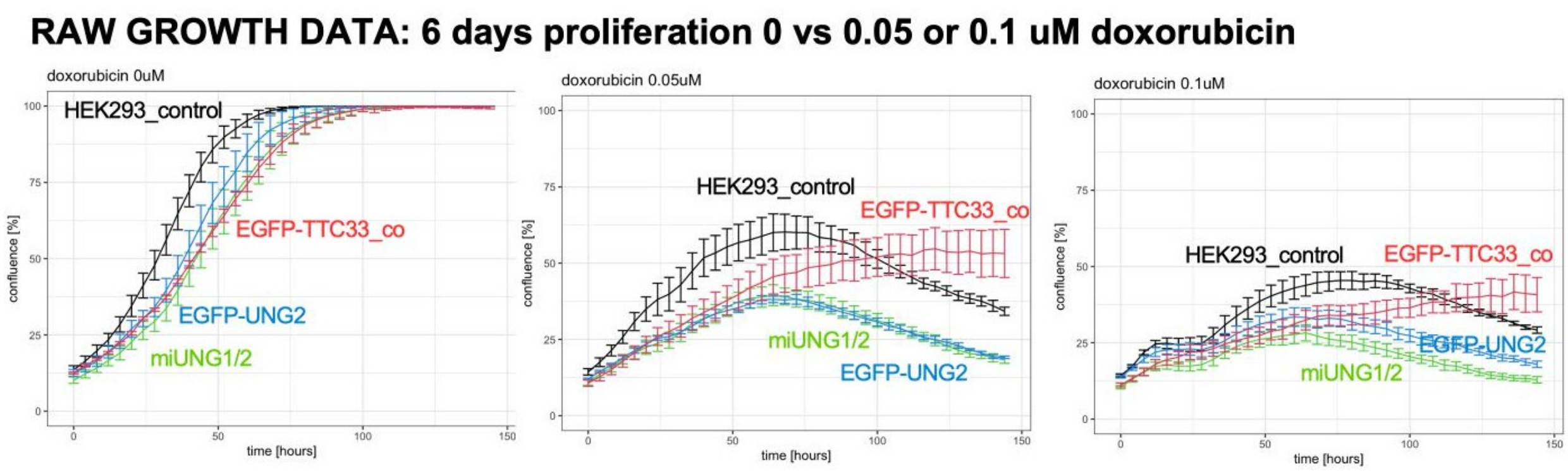

In view of this data we revised the main text and discussion:

MAIN TEXT:

'*To test for tolerance of DNA DSBs, we evaluated sensitivity to doxorubicin. The drug impaired control cell line growth in a dose-dependent manner (Fig. 10f; Supplementary Fig. 9c,d). At low concentration, which did not affect control cell growth, proliferation of cells depleted of or over-expressing TTC33 or UNG2 was slightly inhibited (Supplementary Fig. 9e). At higher concentrations of doxorubicin, cell confluence exhibited a growth–plateau–decline profile (Fig. 10f; Supplementary Fig. 9c,d). TTC33-depleted cells displayed reduced growth capacity, reaching a lower maximal confluence and doing so earlier and over a shorter growth period compared with control cells. This phenotype was shared by both UNG1/2-depleted and UNG2 over-expressing cells. TTC33 over-expressing cells reached a similar maximal confluence as control cells, but did so later and over a more prolonged growth period (Fig. 10g). Collectively, these findings suggest that appropriate TTC33 levels are required for an efficient cellular response to genotoxic stress.*'

DISCUSSION:

'*In contrast, doxorubicin specifically induces DNA DSBs, and the altered response to this drug more directly implicates TTC33 in cellular adaptation to DNA damage. At cytotoxic levels, commonly used in cancer therapies, TTC33 loss increased sensitivity, while its over-expression enabled longer cell survival and proliferation, albeit at a lower rate, suggesting a degree of cellular adaptation.* '

Off note:

1. the additional replicates assessing growth of HEK293 cells and UNG1/2, TTC33 depletions and over-expressions were added to the general growth overview previously presented in Fig. 10a. The number of biological replicates for control HEK293 cells exposed to tetracyclin increased from 17 to 30 biological replicates, UNG1/2 knock-down from 8 to 14, EGFP-UNG2 from 4 to 11, miTTC33 from 22 to 31, and TTC33 over-expression from 34 to 44. This did not affect the previously reported growth defects observed in TTC33 knock-down or over-expression, CCDC97 knock-down and EGFP-UNG2 over-expression. It did detect a minor growth defect in UNG1/2 knock-down.
2. the ROS sensitivity panel was transferred to the Supplement (at present Fig. S9b).

RESPONSE 4: ('*Importantly, reduction in growth is not observed in UNG1/2 knock down, although there is an increased in p53 phosphorylation, while reduced growth is observed only when UNG1/2 is overexpressed, weakening the suggested similarities between TTC33 and UNG1/2 in repair (lines 437-439 and lines 449-453)*')

We agree that TTC33 or CCDC97 loss does not fully replicate all the phenotypes linked to UNG1/2 loss. More so that we showed that neither has a strong effect on uracil excision in whole-cell extracts (Supplementary Fig. 7d,e). The proliferative phenotype is more markedly shared with PPP2R2A, which has also been linked to DNA repair (Kalev et al., 2012). In our work we simply showed that TTC33 and CCDC97 loss induces genomic instability, which is a phenotype previously reported for *bona-fide* DNA repair enzymes, including UNG1/2 and PPP2R2A. We agree with the Reviewer that this does not directly tie TTC33 or CCDC97 to DNA repair, as both factors could supervise a chromatin-related process that increases the risk of DNA damage. To mitigate those issues, we improved the wording of both sections. We also stress that we had discussed the question of the possible source of DNA damage in TTC33-deficiencies in the final paragraph of the discussion.

Line:	Action	Original quote	Revised quote
437-439 (part of DISCUSSION):	the text was revised to increase the precision of the conclusions	*Since both UNG1/2 and PP2A-B55α participate in DNA repair, with the former initiating BER, and the latter dephosphorylating γH2AX following DNA damage repair , we concluded that a function in DNA repair is prominent among characterized TAN factors. Indeed, the chromatin in nuclei isolated from TTC33-depleted cells was clearly more fragmented, which suggests a decreased ability to repair DNA DSBs. Accordingly, nuclear γH2AX levels, which is the most direct marker of DNA DSBs, were increased in TTC33, CCDC97, PPP2R2A, and WDR61 knocked-down cells. Thus, in general, TAN-depleted cells are impaired at DNA DSB repair.*	*UNG1/2 and PP2A-B55α participate in DNA repair, with the former initiating BER, and the latter dephosphorylating γH2AX following DNA damage repair. Considering that many TAN subunits function in DNA repair, we investigated genomic stability in TTC33. Chromatin in nuclei isolated from TTC33-depleted cells was clearly more fragmented, and nuclear γH2AX levels, which represent the most direct marker of DNA DSBs, were increased in TTC33, CCDC97, PPP2R2A, and WDR61 knocked-down cells. Thus, in general, TAN-depleted cells display genomic instability, but the source of the damage is unclear.*
449-453 (part of DISCUSSION):	the text was revised to increase the precision of the conclusions<br><br>(fragment also modified following experimental request)	*Finally, cells depleted of TTC33 and CCDC97 are more sensitive to ROS, similarly to those lacking UNG1/2. ROS produce multiple chemical modifications to DNA, lipids, and proteins, which are poorly tolerated by any cell already impaired in DNA repair. More so if BER, which is responsible for the removal of oxidized bases, is compromised.*	*Finally, TTC33 levels affected the cellular response to ROS and doxorubicin. Increased sensitivity to ROS was a phenotype shared with UNG1/2 depletions. ROS oxidize DNA, lipids, and proteins, which is poorly tolerated by cells already experiencing genomic instability, regardless of its source. In contrast, doxorubicin specifically induces DNA DSBs, and the altered response to this drug more directly implicates TTC33 in cellular adaptation to DNA damage. At cytotoxic levels, commonly used in cancer therapies, TTC33 loss increased sensitivity, while its over-expression enabled longer cell survival and proliferation, albeit at a lower rate, suggesting a degree of cellular adaptation.*

Off note, with increased number of replicates following the doxorubicin resistance assay, we are able to more precisely evaluate the growth defect of cells depleted of UNG1/2 and we did find a very modest decrease in proliferation rate. This is summed up in the revised Fig. 10a.

Figure 10

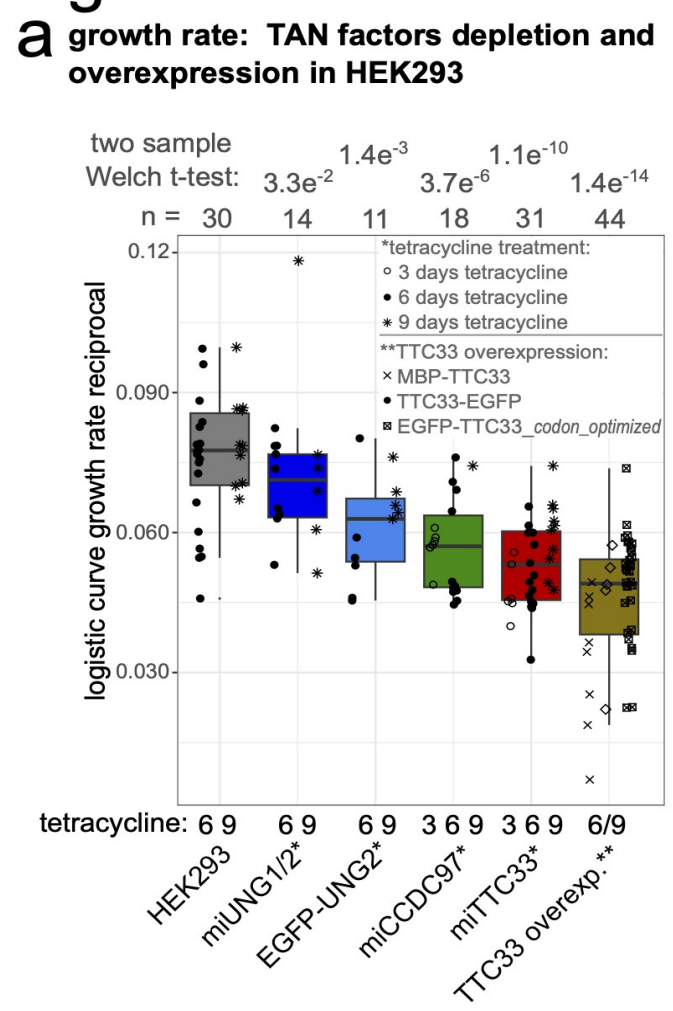

<u>RESPONSE 5:</u> ('*Finally, a reduction in growth is also observed after TTC33 overexpression not only after TTC33 depletion. Does also overexpression increase H2AX and/or p53 foci?*')

Yes, we showed that TTC33 over-expression does result in nuclear accumulation of p53-S15P. This was shown in Supplementary Fig. 8e. Thanks to the Reviewer's comment, we realized that an IF test of nuclear γH2AX accumulation upon TTC33 over-expression was missing from our analysis. This has been addressed during the revision step. In revised Supplementary Fig. 7b we show modest accumulation of nuclear γH2AX in cells over-expressing TTC33, which is consistent across 3 biological replicates. To this end, three cell lines were evaluated:

1. MBP-TTC33,
2. MBP-TTC33_*codon_optimized*, and
3. EGFP-TTC33_*codon_optimized*.

The first construct produces TTC33 from an open reading frame equivalent to the wild-type copy, while the constructs utilized to establish the remaining two were codon-optimized to increase TTC33 expression. This was performed using the ThermoFisher GeneArt service. Levels of TTC33 over-expression, including total protein western blot analysis, are discussed in Fig. 8f and related Supplementary Fig. 6g,h.

In the previous revision we showed that TTC33 levels are subject to regulation by proteolysis, and that WDR61 is key to maintain normal TTC33 levels. Accumulation of damage upon both TTC33 knock-down and over-expression suggests that the protein dosage matters. However, the exact mechanism of damage is probably different, since the growth outcomes under cytostatic doxorubicin levels are clearly different. We thank the Reviewer for spotting this omitted data that supports this conclusions.

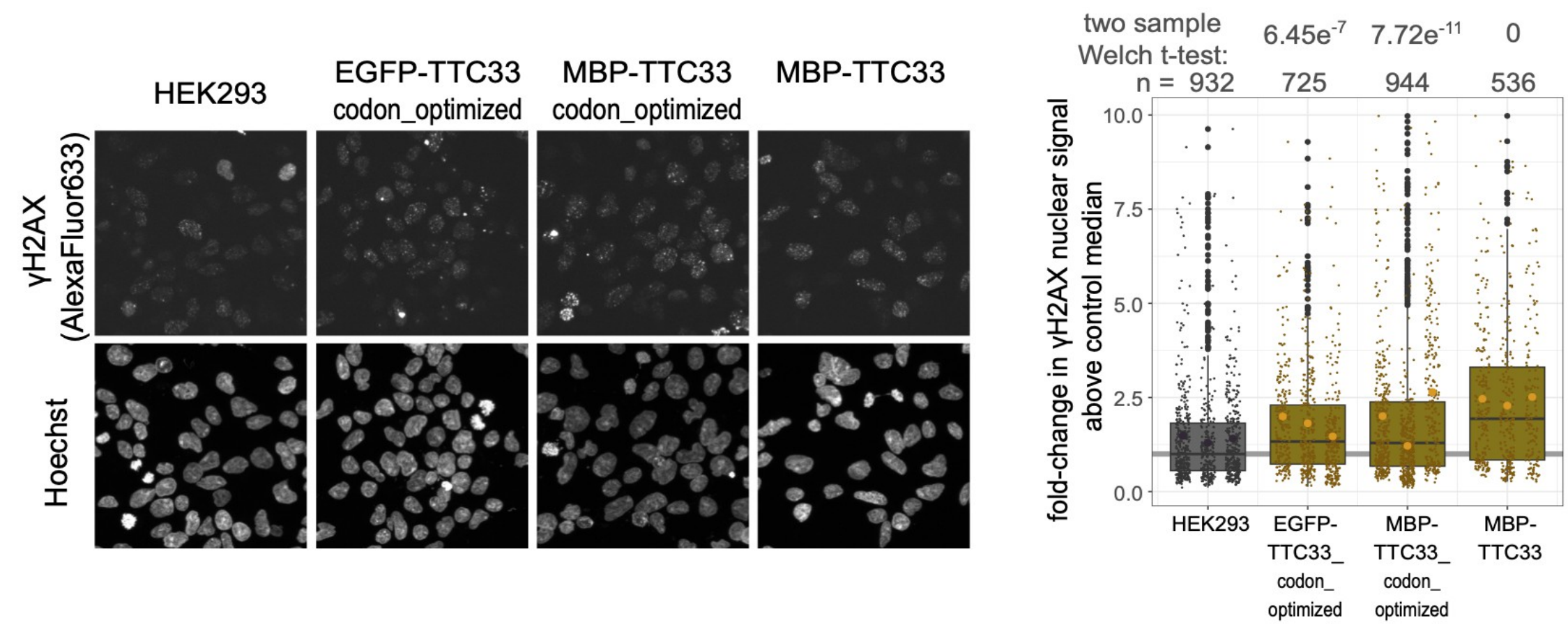

Supplementary Fig. 7b. *The left panel shows IF images of γH2AX (top row) in nuclei (bottom row) of control HEK293 and cells over-expressing TTC33. N-terminal EGFP or MBP tags were used as those provided higher expression. The protein was expressed from an original open reading frame or a codon-optimized one. The graph on the right shows the quantitative analysis of the IF data. Box plots combine data from all replicates and display the median along with the 25th and 75th percentiles. Small dots show the level of signal normalized to the control median in individual nuclei. Those are grouped by replicate and the mean of each replicate is indicated as a large dot. The number of observations and the result of a one-tailed Welch two-sample test are indicated above the graph.*

Minor comments:
1. There are many proteins involved and it is not easy to follow all of their known functions and interactions, what was known before and what is now new. I recommend to add protein-protein interacting network (at least in the supplementary) to define the different complexes-before and after the study.

In Fig. 2c we simply listed the proteins associated with TTC33 in our LF-MS analyses in Fig. 2b. This list also aggregates those factors into complexes and indicates their function. We would like to keep this for simplicity. Fig. 4g shows a scheme of the two TTC33 interaction sub-networks. This scheme synopsizes the conclusions derived from the LF-MS analyses and coIP-WB experiments performed to validate the existence of the TAN network. The collected data showed that TTC33, PHF5A, and WDR61 interact in a mutually exclusive manner with either UNG1/2 or SF3B and CCDC97. The scheme was updated to include the designation of the assemblies as TAN-U and TAN-SC, respectively. In Fig. 5e, the initial scheme demonstrated that TTC33-PHF5A-WDR61 forms a trimeric complex, as evidenced by the data presented earlier in Figure 5. This trimer interacts with PP2A-B55α, SF3A/B, CCDC97, and UNG1/2. We hereby propose an improved version of the scheme, which also integrates the two sub-networks (TAN-U and TAN-SC). Additionally, the figure legends for both schemes have been refined. It was also noted that the legend to Figure 5e was missing. We appreciate the opportunity to address this matter.

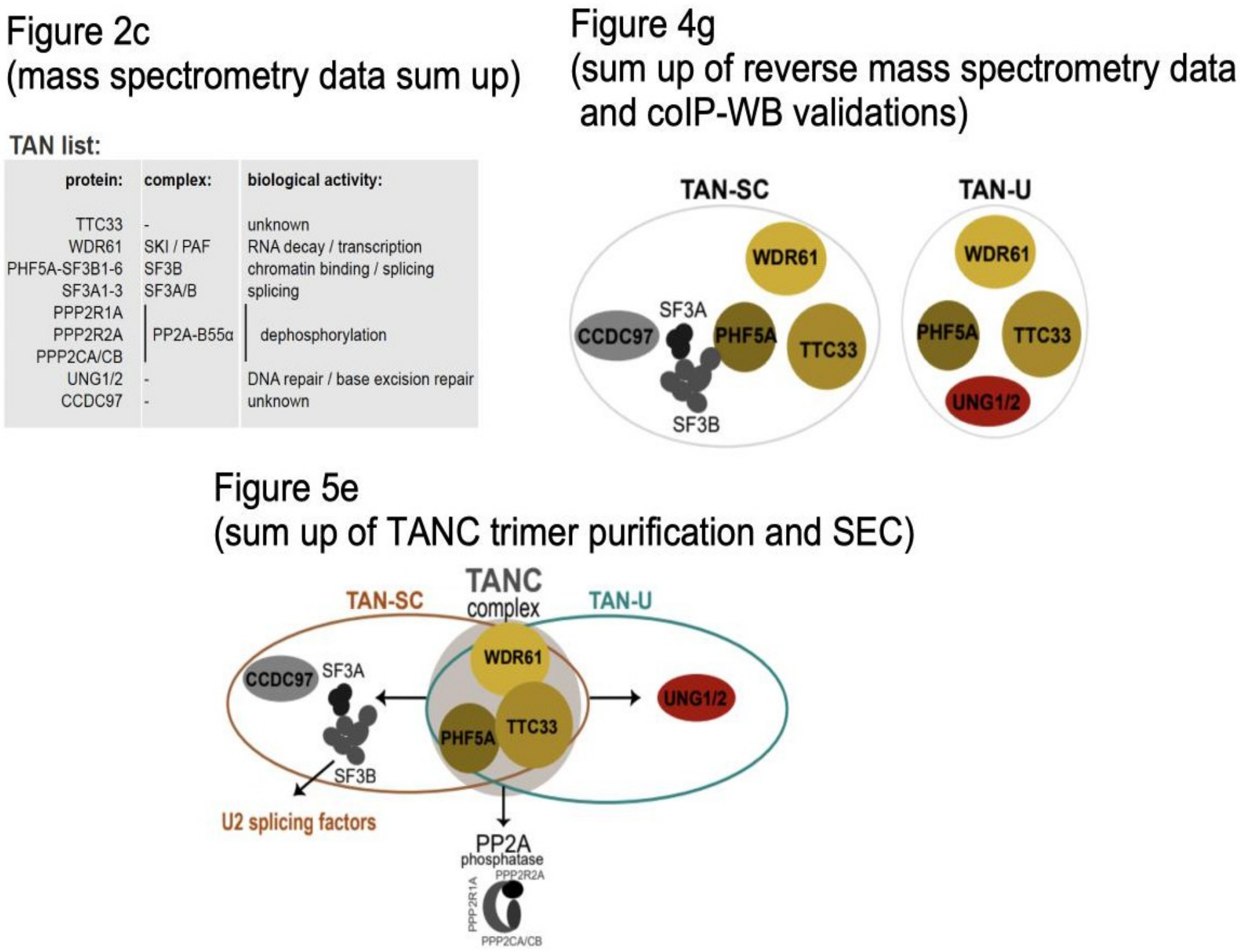

Q1: Did the coIP-LF-MS analysis of PP2RA identify also TTC33?
Q2: Did the coIP-LF-MS analysis of WDR61 identify also PP2RA?

We respond collectively to the two questions listed above (marked Q1 and Q2). In short, **both TTC33 and WDR61 co-purify with PP2A-B55α** subunits: scaffolding PPP2R1A, catalytic PPP2CA or CB, and regulatory PPP2R2A. In case of WDR61, the association with PP2A-B55α subunits was greatly reduced in the WDR61_W20C mutant that is incapable of binding to TTC33.

When purified and analyzed by LF-MS, the PPP2R2A protein only co-purify with the scaffolding and regulatory subunits, along with its known inhibitor, FAM122A*. However, FAM122A was never identified as TTC33 or WDR61 interactor in LF-MS analyses! This suggests that TTC33 and WDR61 purify an active PP2A-B55α complex. We have not stressed this to in the manuscript to avoid over-interpreting our data. Importantly, when strongly over-expressed, PPP2R2A associates with other TAN factors, which can be observed by coIP-WB.

The table below sums up this data, which is scattered throughout the manuscript**:**

**Experiment type:**	**BAIT: TTC33**	**BAIT: WDR61**	**BAIT: PP2A**
**LF-MS**	Fig. 2b:<br><br>Identified PP2A subunits catalytic PPP2CA, scaffolding PPP2R1A, and regulatory PPP2R2A	Fig. 4c:<br><br>Identified PP2A subunits catalytic PPP2CA, scaffolding PPP2R1A, and regulatory PPP2R2A<br><br>Fig. 8d:<br><br>showed that WDR61 co-purification with PP2A subunits was decreased in the WDR61 W20C point mutant that was impaired in interaction with TTC33.	BAIT PPP2R2A:<br><br>Fig 4a:<br><br>showed that regulatory subunit PPP2R2A co-purified with PPP2R1A and PPP2CA/CB.<br><br>However, TAN factors were not reliably identified. Instead PPP2R2A co-purified with its inhibitor, FAM122A.<br><br>IMPORTANTLY: **FAM122A did not co-purify with TTC33 or WDR61.**
**coIP-WB**			Fig. 4b:<br><br>showed PPP2R2A co-purification with low levels of endogenous WDR61<br><br>Fig. S2c:<br><br>showed PPP2R1A co-purification with endogenous WDR61, and co-expressed TTC33.<br><br>Fig. S2d:<br><br>showed co-purification of co-expressed UNG2 and PPP2R2A (PPP2R2A as prey!)

**To facilitate understanding of our data we improved the citation regarding FAM122A. This protein was proposed to be a PP2-B55α inhibitor by Li et al., 2020, and we have cited this research in the first manuscript version. However, a more comprehensive study was produced by Wasserman et al. (2025); (https://www.nature.com/articles/s41467-024-50015-7), who demonstrated that FAM122A directly interacts with and inhibits PP2A-B55α, thereby suppressing its phosphatase activity in the DNA damage response and influencing cell-cycle progression.*

2. I appreciate the introduction on genome stability, but there should also be a short introduction on what is known on TTC33 functon(not only structure).

We agree that adding information about TTC33 function to the introduction section would be beneficial, however, very little information on TTC33 is available in peer-reviewed publications. There are only four

scientific papers, which we listed below, that mention TTC33. All do so in the context of transcriptomic profiling of cancer cells or tumors and reveal a putative role of PRKAA1 kinase in carcinogenesis. *TTC33* is encoded on the same strand and downstream of *PRKAA1* kinase, which we show in the Fig. 1a. This locus undergoes rearrangements frequent enough to be at times reported in unrelated cancers (lymphoma, ovarian, prostate, breast cancer). Those rearrangements lead to truncation of the PRKAA1 C-terminus and production of a chimeric transcript with downstream TTC33 or its fragment. However, we verified that those chimeric transcripts did not necessarily encode TTC33 in frame with *PRKAA1*. Thus, most publications listed below rather point towards *PRKAA1* truncation being the factor contributing to carcinogenesis, with no clear possible function for the TTC33-coding part.

To address the Reviewer's comment, we re-wrote the beginning of the last paragraph of introduction. We now mention and cite two representative works by Matsumoto et al. (2021) and Plebani et al. (2012) in the introduction, and emphasize the lack of functional characterization of TTC33.

'*Here, we investigated Tetratricopeptide Repeat (TPR) Domain 33 (TTC33), a predicted TPR-containing protein. TTC33 is encoded adjacent to PRKAA1 within a genomic locus reported to undergo rearrangements in several cancers (Matsumoto et al., 2021; Plebani et al., 2012). Apart from its annotation in large-scale interaction and cancer transcriptomic datasets, no direct functional characterization of TTC33 has been reported.*'

Publications reporting PRKAA1-TTC33 locus truncation in cancers (the two bolded ones have been added to the list of References in the revised version):

Matsumoto Y, Tsukamoto T, Chinen Y, Shimura Y, Sasaki N, Nagoshi H, Sato R, Adachi H, Nakano M, Horiike S, Kuroda J, Taki T, Tashiro K, Taniwaki M. Detection of novel and recurrent conjoined genes in non-Hodgkin B-cell lymphoma. J Clin Exp Hematop. 2021 Jun 5;61(2):71-77. doi: 10.3960/jslrt.20033. Epub 2021 Apr 20. PMID: 33883344; PMCID: PMC8265495.

Meng J, Fan X, Zhang M, Hao Z, Liang C. Do polymorphisms in protein kinase catalytic subunit alpha-1 gene associated with cancer susceptibility? a meta-analysis and systematic review. BMC Med Genet. 2018 Oct 19;19(1):189. doi: 10.1186/s12881-018-0704-8. PMID: 30340465; PMCID: PMC6194619.

Plebani R, Oliver GR, Trerotola M, Guerra E, Cantanelli P, Apicella L, Emerson A, Albiero A, Harkin PD, Kennedy RD, Alberti S. Long-range transcriptome sequencing reveals cancer cell growth regulatory chimeric mRNA. Neoplasia. 2012 Nov;14(11):1087-96. doi: 10.1593/neo.121342. PMID: 23226102; PMCID: PMC3514740.

Luo JH, Liu S, Zuo ZH, Chen R, Tseng GC, Yu YP. Discovery and Classification of Fusion Transcripts in Prostate Cancer and Normal Prostate Tissue. Am J Pathol. 2015 Jul;185(7):1834-45. doi: 10.1016/j.ajpath.2015.03.008. Epub 2015 May 9. PMID: 25963990; PMCID: PMC4483459.

3. I still do not agree on the conclusion that since “TTC33 and its interactors reside in common sub-cellular compartment, i.e. the nucleus”, “TAN might be biologically meaningful in various cell types” (Lines 157-160). The localization of both TTC33 and CCD97 is both cytoplasmic and nuclear (according to fractionation, TTC33 is almost more cytoplasmic, and according to imaging mostly nuclear, while CCD97 is still mostly nuclear but clearly also cytoplasmic with both assay), with no clear foci formation, so the conclusion would be valid for any pan-cellular protein. The conclusion should better reflect the significance of these data.

As previously stated, modifications were made to the section addressing TTC33 and CCDC97 localization. It is evident that there is a general consensus on the nucleo-cytoplasmic localization of proteins. However, the results are not unequivocal with respect to the proportion of each protein in the nucleus or cytoplasm. The disparities may be attributed to technical nuances; it is conceivable that the incorporation of the EGFP tag could modify the protein localization equilibrium. Conversely, the amounts of protein resulting from fractionation may not accurately reflect the actual subcellular proportions. In light of the these procedural issues, it was deemed preferable to incorporate both assays and thereby refrain from quantitative deliberations entirely.

'*TTC33 contains a putative NLS (Fig. 1b,c), prompting us to examine its localization. Microscopy revealed that large fractions of EGFP-tagged TTC33 or CCDC97 were localized to the nucleus in living and fixed cells alike, though a*

cytoplasmic presence was also evident, especially for CCDC97 (Fig. 3d). These intracellular localization patterns were evidenced by cell fractionation coupled to western blot (Fig. 3e). Thus, TTC33 and CCDC97 localize to both nucleus and cytoplasm. Importantly, their interactors (WDR61, SF3A/B, PP2A-B55α, and UNG) are likewise detected in the nucleus (Fig. 3e; Supplementary Fig. 2b); (Kalev et al., 2012; Reynhout et al., 2019; Seshacharyulu et al., 2013; Vos et al., 2018; Cretu et al., 2016; Sousa et al., 2007).'

Off note, the section addressing tissue expression of TTC33 and TAN factors has undergone modification to incorporate a correlation matrix (new Fig. 3b,c and Supplementary Fig. 2a) in response to the comments provided by Reviewer 4. Please refer to the Responses to Reviewer 4 for further details.

Comments to text and figures:
line 59: natural replication does not induce DSBs (as meiosis and CS recombination)

We removed 'replication' from this line.

line 63: there is also alternative End Joining (alt-EJ), even more prone to errors than NHEJ.

We included this information in the indicated line in the revised manuscript along with a new reference to the a-EJ review by Sallmyr and Tomkinson, (2018).

Sallmyr A, Tomkinson AE. Repair of DNA double-strand breaks by mammalian alternative end-joining pathways. J Biol Chem. 2018 Jul 6;293(27):10536-10546. doi: 10.1074/jbc.TM117.000375. Epub 2018 Mar 12. PMID: 29530982; PMCID: PMC6036210.

line 67: "MDC1:53BP1:BRCA1:MRN foci" is an over simplification and is not further addressed in the manuscript, I suggest a simpler "repair foci".

This was fixed as suggested.

Lines 75-78: the explanation of cancer tissue-specificity is over simplistic. And in this case, also not completely correct, For example, familiar mutations in BER genes do not strongly correlate with liver or kidney cancers

We agree with the Reviewer that we oversimplified the link between DNA repair pathways, tissue-specific metabolism, and cancer. While strong links clearly exist, such as UV-crosslink repair and skin cancer, the association between BER mutations and liver or kidney cancers is less solid. We replaced the lines 75-78 with a more generic statement in line with the cited review by Semmler et al. (2019):

'*DNA repair enzymes are expressed in all cell types (Sun et al., 2019). However, due to tissue-specific gene expression patterns or metabolic particularities, some cells may rely more heavily on certain repair pathways to maintain genomic integrity (Semmler et al., 2019).*'

Lines 135-136: I may be wrong, but the interaction between TTC33 and SF3B4 in HeLa cells is not validated by the Co-Ip shown in Figure 2d.

The coIP-WB shown in Fig. 2d was intended as a validation of the LF-MS data in Fig. 2b. **It clearly confirms MBP-TTC33 association with WDR61, CCDC97, UNG1/2, and PHF5A in both HEK293 and HeLa cells. Thus, we did largely confirm TTC33 association with the majority of TAN factors**. The specificity of this experiment was further conformed by using α-tubulin as negative control.

Aside from PHF5A, the majority of other **SF3B complex subunits** were identified in our LF-MS analysis less consistently (Fig. 2b; Supplementary Dataset 2), and therefore **their validation was expected to be more problematic**. However, **the coIP-WB in Fig. 2d clearly confirmed TTC33 association with SF3B4 in HEK293** cells but did not reveal a major enrichment in HeLa, most likely due to the technical problem resulting from the unspecific cross-recognition of some unrelated protein with molecular weight similar to that of SF3B4. While we understand the Reviewer's concern that the interaction in HeLa is less

certain, we would like to stress that the LF-MS data from HeLa we produced using a TTC33-EGFP bait showed clear SF3B subunit enrichment.

We feel we had discussed this issue thoroughly enough in the main text (in the paragraph following reference to Fig. 2d), where we say that in our LF-MS most SF3B subunits, aside from PHF5A, were identified only using relaxed criteria. We also presented the LF-MS data in Supplementary Fig. 1f and its caption, and presented the components of the entire analysis in Supplementary Dataset 2.

Line 138: the definition of SKI and PAF should be added.

This has been corrected. Thank you for spotting this issue.

Line 141: the definition of SF3A/B should be added.

As above, this has also been corrected.

Lines 154-155: it is not clear how Supplementary Figure 2a shows the nuclear functions of WDR61, SF3A/B, PP2A-B55alpha and UNG.

Former Supplementary Fig. 2a (presently 2b) showed microscopy images of PP2A-B55α localization. The nuclear functions of WDR61, SF3A/B, and UNG1/2 are well established by published manuscripts. However, since the nuclear DNA repair function of PP2A-B55α is somewhat less characterized and inspired mainly by the work of Kalev et al., (2012: DOI: 10.1158/0008-5472.CAN-12-1667), we simply added a microscopic localization assay to complement the fractionation result shown in present Fig. 3e. It seemed relevant to us at the time, because PP2A-B55α was also suggested to play cytoplasmic functions (*e.g.* Pan et al., 2024: DOI: 10.1016/j.celrep.2024.114379).

We further simplified this section, and hope that this increased clarity:

'*TTC33 contains a putative NLS (Fig. 1b,c), prompting us to examine its localization. Microscopy revealed that large fractions of EGFP-tagged TTC33 or CCDC97 were localized to the nucleus in living and fixed cells alike, though a cytoplasmic presence was also evident, especially for CCDC97 (Fig. 3d). These intracellular localization patterns were evidenced by cell fractionation coupled to western blot (Fig. 3e). Thus, TTC33 and CCDC97 localize to both nucleus and cytoplasm. Importantly, their interactors (WDR61, SF3A/B, PP2A-B55α, and UNG) are likewise detected in the nucleus (Fig. 3e; Supplementary Fig. 2b); (Kalev et al., 2012; Reynhout et al., 2019; Seshacharyulu et al., 2013; Vos et al., 2018; Cretu et al., 2016; Sousa et al., 2007).*'

Line 247: the reduction in interaction with SF3B4 seem higher than “slightly”.

We agree, and apologize for the wording. We now state:
'*It also reduced the interaction with SF3B4 (Fig. 7c)*'

Line 257: I think it should be “TPR4 alone” and not “TPR alone”.

We clarified this fragment:

'*This narrowed the WDR61/PHF5A-binding region to the TPR domain and identified TPR4 as an essential one.*'

Lines 320-321: add how varied.

This segment of the text was corrected:

'*We compared the interaction profiles of TTC33-EGFP and EGFP-TTC33_codon_optimized by coIP-WB (Supplementary Fig. 6h), and observed that TTC33-EGFP precipitated more efficiently WDR61, UNG1/2,*

and PPP2R2A when compared to bait level. We conclude that the N-terminal tagging impairs TTC33 abundance regulation, possibly due to altered interactions with TAN factors.'

Figure 1: is the human there? And would it be possible to have the name order in each panel?

Fig. 1a shows the genomic alignments of TTC33 locus in several vertebrates. There, the human genome assembly is used as reference and therefore is not explicitly displayed. Figures 1b and 1c display TTC33 protein sequence analysis, and each follow the same order.

Regretfully, the ECR Browser displays a limited choice of species, with preference for mammalian genomes. Thus, ordering the panel 1a exactly as in the protein analysis presented in panels 1b,c is not possible using this tool.

Figure 6 d-e: use the same color code.

This has been corrected, thank you for the suggestion.

Figure 7d and Supplementary Figure 5a: use another color to indicate the C-ter of PHF5 that is important for the interaction with TTC33. It is difficult to see where it is. The legend is not indicating what is shown in gray and orange

The panel has been improved in color usage and appropriately described.

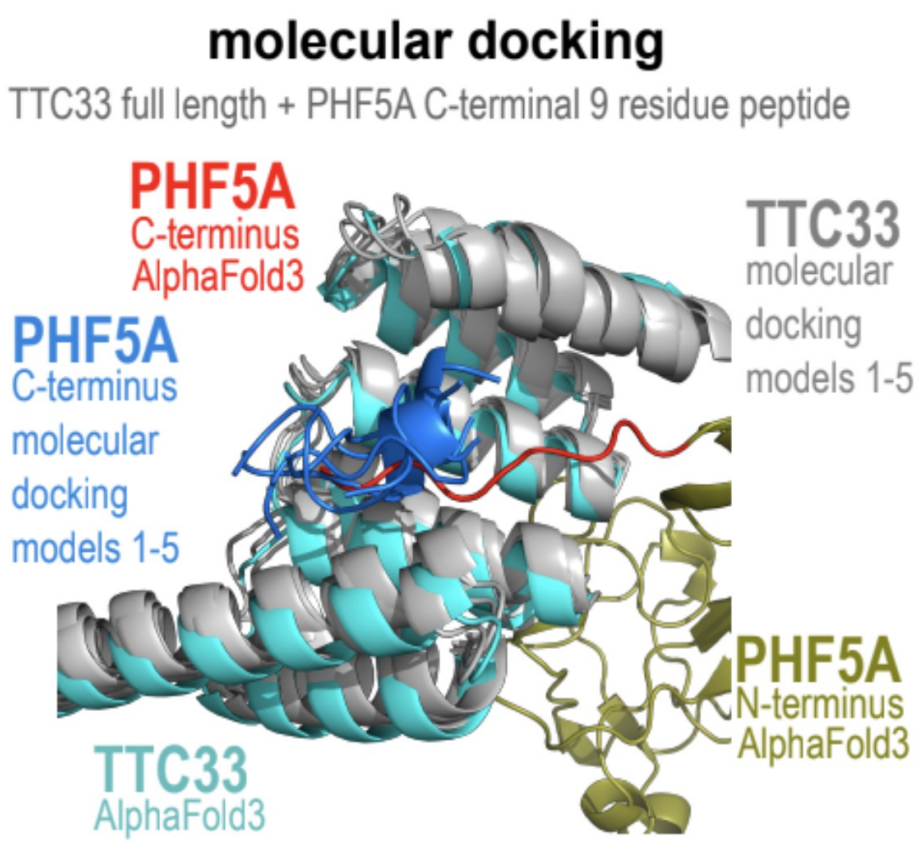

Figure 7e. add the name of the first and last aa, for both peptides

Selected amino acid names were added to the first state in the series to provide orientation in regards to the more thoroughly described panel 7b.

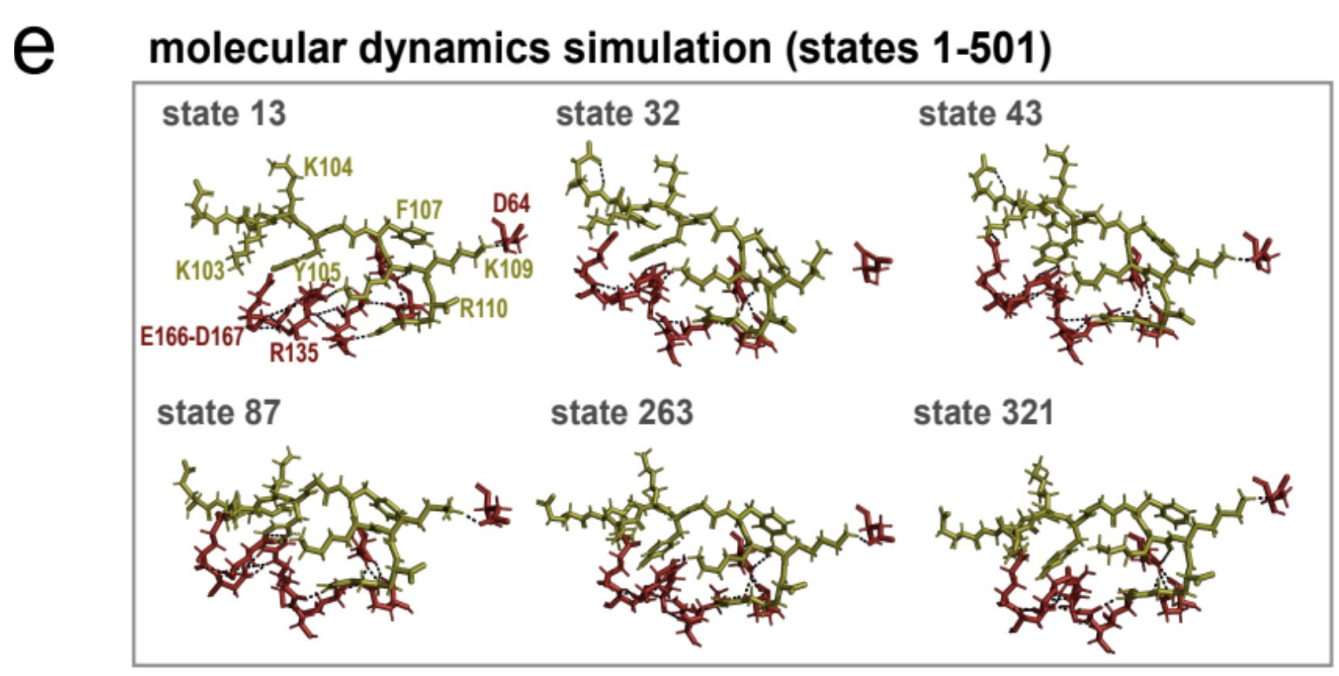

Figure 7f: is it possible to highlight in different colors the residues tested in the Co-Ip and their impact on the interaction?

The scheme itself was modified as requested. The legend below the scheme was improved.

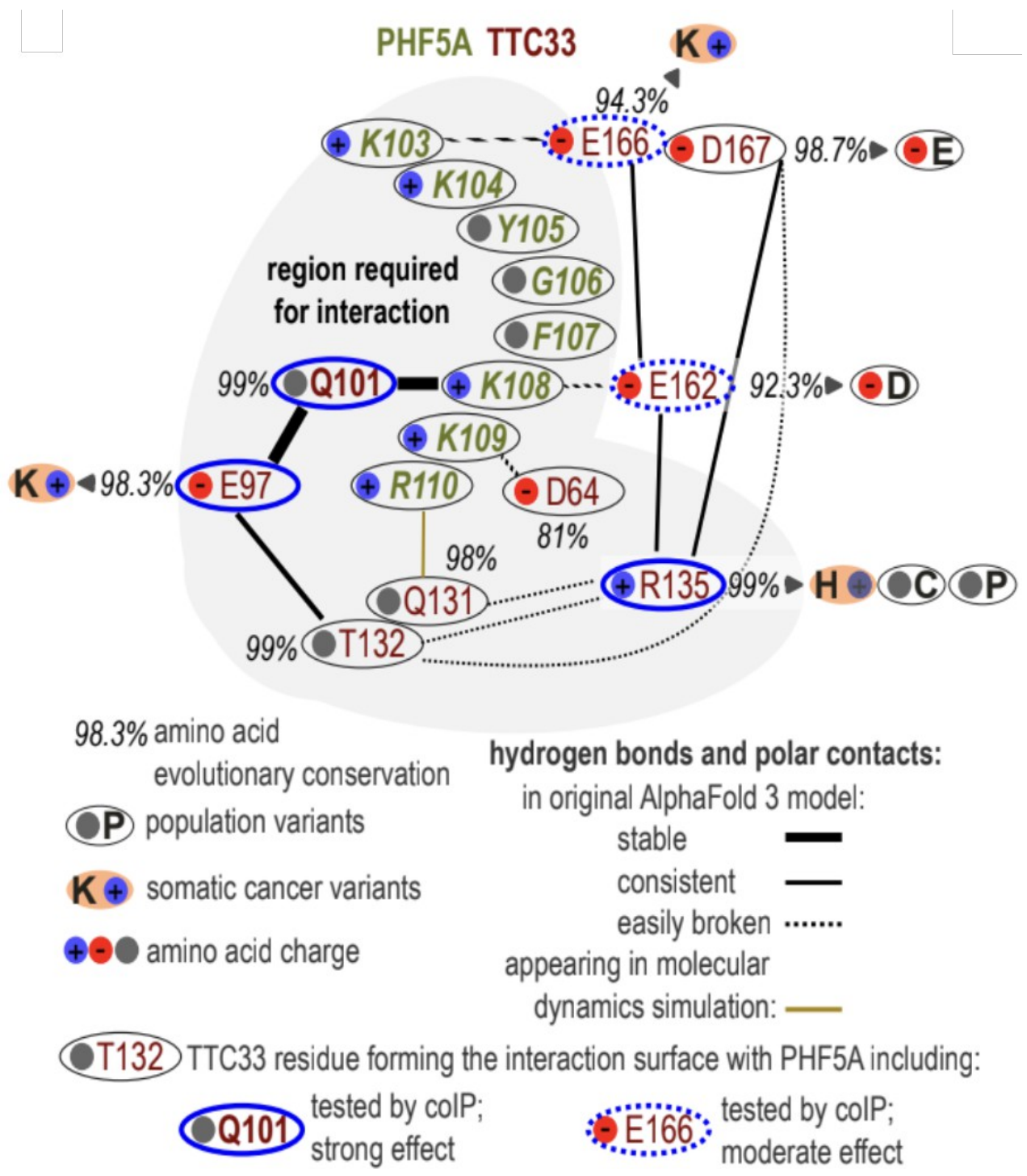

Figure 7g. add a schematic of the mutant fragments

A schematic was added to Fig. 7g and Supplementary Fig. 6b. As an example, we show Fig. 7g scheme:

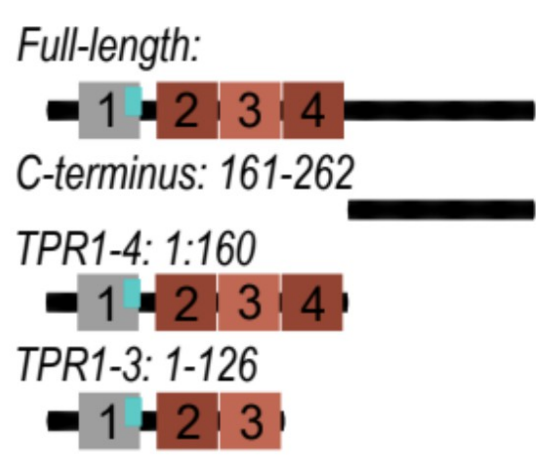

Figure 7h: add if the mutations is a rare variant or a cancer variant

We added a column named 'SOMATIC (COSMIC)' which will inform if the variant was assessed as somatic mutation by the COSMIC database. All the point mutations used in this study were assessed as possibly moderate impact by the database. The COSMIC database routinely assesses somatic variants arising in cancers.

In addition, we also added a 'Population variant' column that indicates if the variant was reported in 1000Genomes, ExAC, TOPMex, dsSNP, or gnomAD databases. The gnomAD database and its predecessor ExAC best represent true population variant frequencies, as they are based on transcriptomic and genomic data that do not promote inclusion of disease cohorts. 1000 Genomes presents mainly common variants, but its population size is limited (resulting in possible exclusion of rare variants). In contrast, TOPMed includes many disease cohorts, whereas dbSNP is primarily a variant registry and does not discriminate between population or disease variants nor does it tackle disease association. We thus aggregated the gnomAD, ExAC, and 1000Genomes into category 'healthy' and the TOPMed and dbSNP to 'healthy with disease cohorts'. Additional explanation was included in the figure legend.

h

MUTATION	VARIANT IDENTIFIER	ΔΔG	CADD	SOMATIC (COSMIC)	POPUL. VARIANT
E97K	COSV61802990/rs144481517	-1.08	29.6	YES: M	gnomAD, ExAC, 1000Genomes, TOPMed, dbSNP
H125Q	rs1482328927	-0.10	14.4	NO	TOPMed
R135H	COSV61802702/rs759828624	+0.40	32.0	YES: M	gnomAD, ExAC, TOPMed, dbSNP
R135P	rs759828624	+5.42	32.0	NO	gnomAD, ExAC, TOPMed
R148Q	COSV61802519/rs1182763551	-0.16	29.4	YES: M	gnomAD, TOPMed, dbSNP
H155Y	COSV100531955	-0.36	27.3	YES: M	
E166K	COSV61802309	-0.12	27.1	YES: M	

ΔΔG: unlikely destabilizing; likely destabilizing
CADD: likely benign; probably deleterious; highly likely deleterious
Population variants: gnomAD, ExAC, 1000Genomes (healthy); TOPMed, dbSNP (healthy with disease cohorts)

Figure 7h *Table showing a collection of TTC33 population variants and somatic cancer mutations along with their identifiers, which were used for coIP-WB analyses shown in panel i. Each mutation targets a residue relevant to WDR61 or PHF5A binding. Two scores are indicated in the table. The ΔΔG score predicts how a single mutation will affect protein stability. Values close to 0 indicate low impact, while larger positive and negative values point towards destabilizing or stabilizing effects, respectively. The CADD score integrates several metrics (sequence conservation, genomic and protein features, etc.), ultimately indicating if a substitution is likely to be detrimental to protein function. The 'SOMATIC (COSMIC)' column informs if the variant is listed in the COSMIC cancer variant database. The 'YES: M' indicates that the variant was assessed as somatic one and had predicted moderate impact, according to the repository. The final column reports if the variant is listed in gnomAD, ExAC, 1000Genomes, TOPMed, or dbSNP databases. The first three (gnomAD, ExAC, 1000Genomes) repositories list variants detected in healthy population, whereas the TOPMed and dbSNP databases include healthy individuals and information about disease cohorts. A full overview of TTC33 mutations is given in Supplementary Fig. 5b, where a combined SIFT-Polyphen score measures the putative mutation relevance to protein function.*

Figure 8: red over orange is very difficult to see

In Fig. 8a,b,c we replaced the red color designating the WDR61 amino acids entering in contact with TTC33 or TTC37 from red to light blue. We used the color blindness simulator to verify that this combination will give the best contrast (https://www.color-blindness.com/coblis-color-blindness-simulator/).

Figure 9c: use the same order for the images and the graph-and also for Figure 9d

The order was corrected as requested.

Figure 9e: do the author have an immunoblot for the complementation?

Yes, the western blot analysis showing that EGFP-WDR61 WT can complement for knock-down of endogenous WDR61 was shown and discussed in Fig. 8h. This experiment also shows that the W20C mutant does not complement WDR61 loss, supporting the LF-MS analysis presented in Fig. 8d and validation coIP-WB in Fig. 8e, which show that the mutant has impaired interaction with PAFc, SKIc, and TAN. In fact, those data were appropriately referenced in the Fig. 9e legend but in a convoluted manner as '(see also Fig. 8d,e,h)'. We expanded this to: '*(for WDR61 WT and W20C mutant analysis by LF-MS and western blot see also Fig. 8d,e,h)*'.

RESPONSE TO REVIERWER 4

Reviewer #4 (Remarks to the Author):

This manuscript attempted to characterize and identify the interacting partners of a previously uncharacterized protein: TTC33. The manuscript had a very strong experimental design initially but lacked some method details, validation and elaborate computational analysis. During the revision, the authors substantially improved the documentation and presentation of the methods and results respectively. Moreover, the bioinformatics work now is significantly improved allowing for a better and more in depth study and characterization of the interaction sites of TTC33, more justified hypotheses and conclusions about its functionality and better documented characterization its association with diseases. The authors also improved the overall impact of their study by adding the experiments, data and analysis required for a better validation of the TTC33-associated interaction network and by performing further functional analysis of TTC33 and its partners.

We appreciate the Reviewer's positive assessment of the original work and the effort put into the revision. We'd like to express gratitude for the Reviewer's sharp remarks that helped us to improve out work, and were also a learning opportunity for the group transitioning towards novel research areas.

I believe that there are now only minor missing parts in the manuscript summarized in the following two points:

-Regarding performing coexpression analysis between TTC33 and its partners in various tissues, the authors identified relevant dataset but instead of performing a correlation analysis (eg calculating Spearman's Rho correlation between the potentially interacting pairs), they just confirmed the expression levels of TTC33 and the potentially interaction partners. Co-expression analysis might reveal potentially common regulatory patterns which are usually present in physically interacting proteins and performing this analysis would further improve the manuscript.

Indeed, we did not fully understand the original request. **In the revised version we produced two correlation matrixes using the human tissue mRNA expression data from Fagerberg et al. (2014).**

The graph shown in Supplementary Fig. 3a shows tissue abundance correlation of TAN factors (TTC33, WDR61, PHF5A-SF3B1-6, SF3A1-3, UNG1/2, PP2A-B55α). This shows that expression of SF3A/B-CCDC97 is highly correlated. Though expression of the remaining TAN factors is more loosely clustered, strong correlations were identified, notably WDR61-TTC33 (rho 0.69), WDR61-PPP2R2A (rho 0.61), and WDR61-UNG1/2 (rho 0.73).

The novel main Fig. 3b contains an extended analysis of tissue abundance correlation of TAN and other less consistently identified interactors. Those 42 proteins, used in this analysis, co-purifying with TTC33 were listed in Supplementary Fig. 1f. The correlation matrix is clustered into 4 groups. The TAN factors clearly group in cluster 3 and 4, while clusters 1 and 2 contain factors largely not associated in tissue abundance with TAN. In cluster 3 and 4 we further identify, and briefly characterize in panel 3c, several proteins associated in expression with the group 3 CCDC97:SF3A/B cluster, or being close to with TTC33 or WDR61 in cluster 4.

In our experience, interaction of bait proteins with factors less consistently identified in LF-MS might be difficult to validate, as even with relatively high enrichment some might still be false positives. However, we are convinced that the Reviewer's suggestion for this analysis might help us in the future to identify further functional TTC33 interactors, as shared expression pattern might support direct functional interaction. We are thus grateful for this suggestion.

Supplementary Figure 2

a

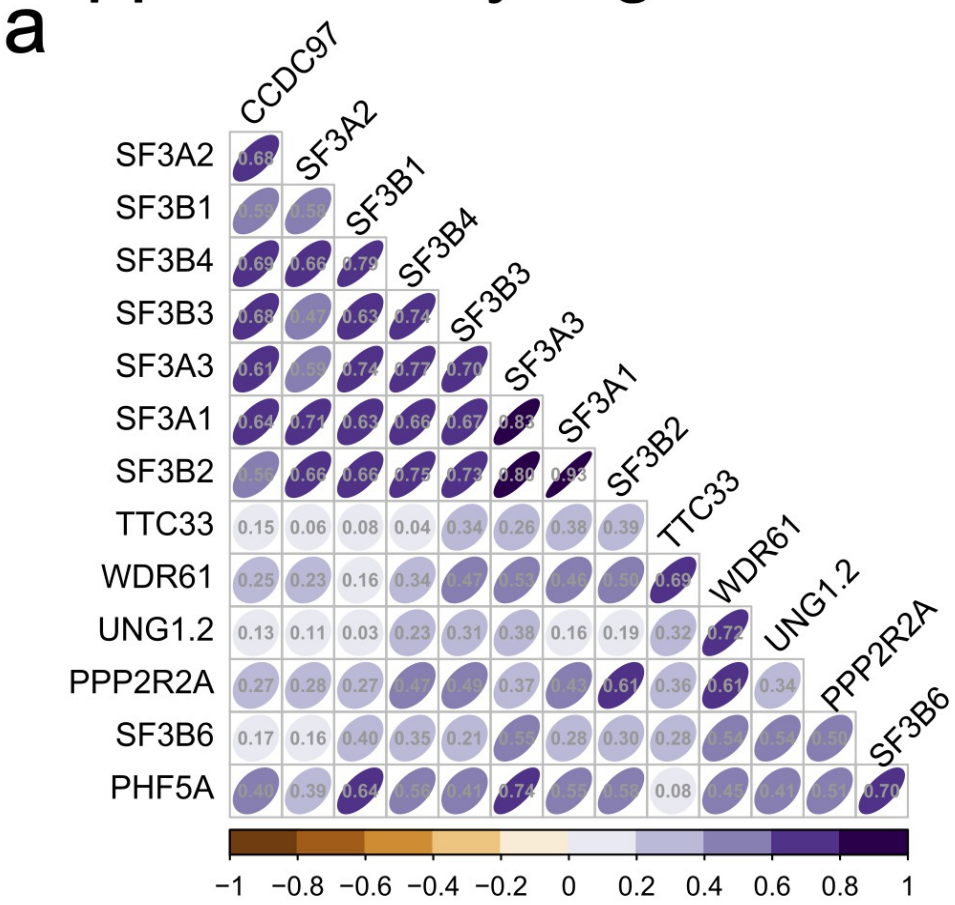

Figure 3

b

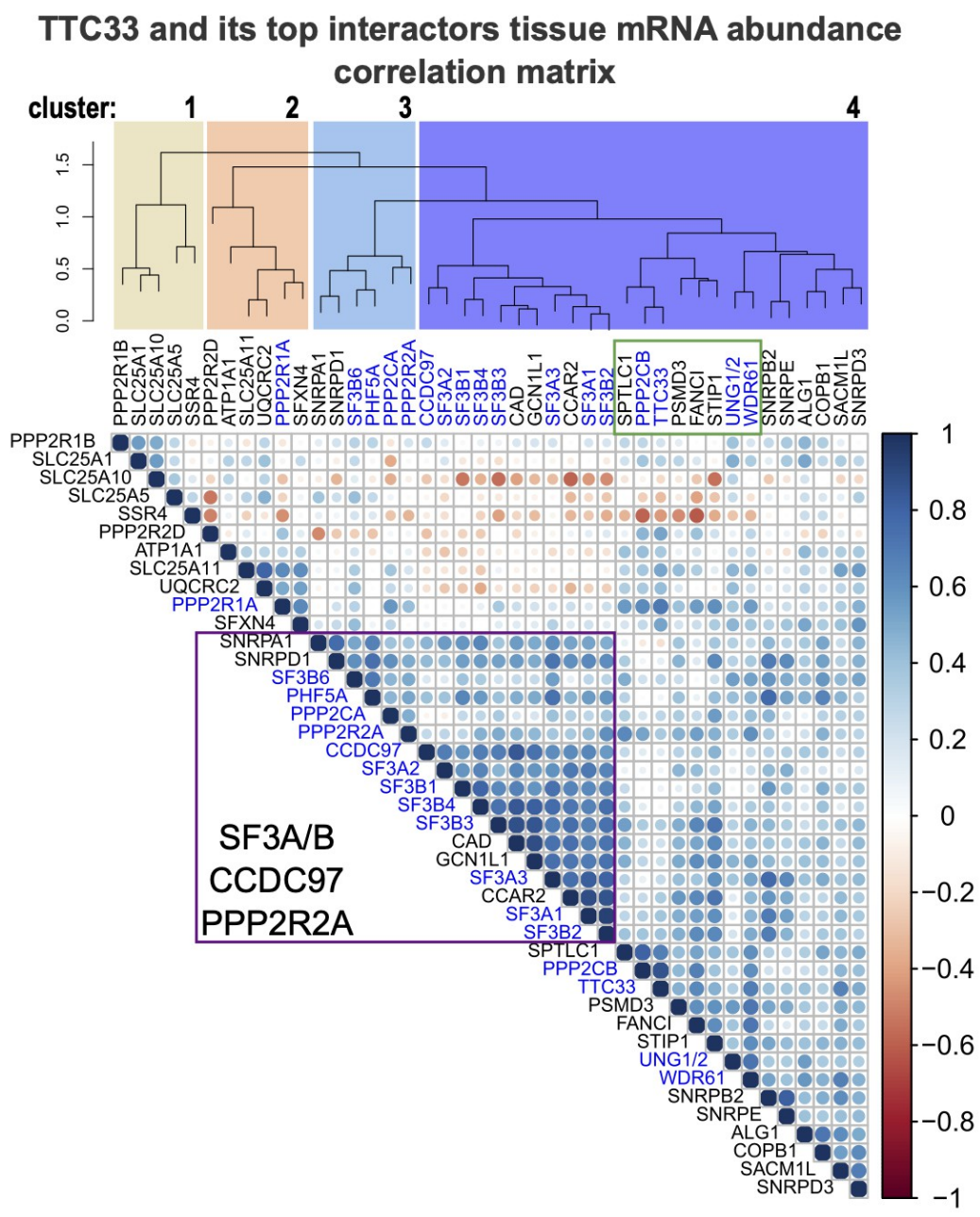

c

SPLICING FACTORS:
CCAR2 (DBC1) - DBIRD complex subunit acting in RNAPII elongation and alternative splicing
SNRP-A1, -B2, -D1, -D3, -E - U2 splicing factor subunits

MISCELLANEOUS:
SPTLC1 - Serine palmitoyltransferase 1
GCN1L1 - stalled ribosome sensor

PROTEIN FOLDING AND DECAY:
PSMD3 - proteasome subunit
STIP1 - stress induced phosphoprotein; HSP90AA1 co-chaperone

DNA DAMAGE REPONSE or REPAIR:
FANCI - Fanconi anemia group I protein
CAD - multifunctional enzyme acting in pyrimidine biosynthesis pathway

Supplementary Figure 2. a *Graph presents a clustered correlation plot evaluating human tissue expression of TAN subunits. Data was derived from Fagerberg et al., (2014). Raw abundance of TAN factors is shown in Fig. 3a, while a broader correlation matrix including a larger TTC33 interactor list is shown in Fig. 3b.*

Figure 3. b *Correlation matrix evaluating human tissue expression (Fagerber et al., 2014) of TAN factors and an extended list of potential TTC33 interactors derived from LF-MS datasets (listed in Supplementary Fig. 1f and Supplementary Dataset 2). The matrix is clustered by expression similarity into four groups. Expression analysis limited to TAN factors is shown in Supplementary Fig. 2a.* ***c*** *Panel summarizes the roles of putative TTC33 interactors other than TAN, which closely clustered with either TAN subunit.*

Main text quote:

'TAN proteins are expressed in humans with no pronounced tissue specificity (Fig. 3a). However, when compared with the extended list of TTC33 interactors, expression of TAN factors markedly correlated (Fig. 3b; Supplementary Fig. 1f,2a). The TAN cluster was split into highly correlated SF3B-CCDC97 and more loosely associated TTC33-WDR61-UNG1/2. Within the extended interactor list, only a few additional factors showed correlated expression, primarily involved in splicing, protein folding and degradation, and DNA damage response or repair (Fig. 3c).'

-The process for selecting templates for homology modelling through the Swissmodel automatic option is now selected. But to reassure that there des not exist any more relevant template the authors should provide a table of the potential templates that swissmodel reports including metrics such as coverage, identify, experimental method of template structure and oligomeric state.

Yes, the Reviewer is right that we omitted this crucial methodology description and quality report. **To amend this shortcoming, we produced the Supplementary Table 1 (also shown below). It lists the top 5 templates and models proposed by Swiss-Model for TTC33 structural modeling.**

All TTC33 structural models are deposited at Mendeley data together with their respective reports. The full transcript of potential templates is also deposited herein. This full list includes alternative pdb models of most 5 top-ranking templates listed in Supplementary Table 1. For simplicity, and to omit redundancies, we only listed the best-ranking template in the Supplementary Table 1 report.

The Supplementary Table 1 is referenced in the main text, and in the Figure 6a caption, wherein we now mention that four other additional models were also examined, out of which three produced structural similarity to the main 8chy model, shown in the main Figure 6a.

During this analysis the 8chy_TTC33 model retained its highest rank, giving the best QMEAN score. A structural model 9qhc has been published in 2025 (when our work was in revision) that produces a better RMSD with the TTC33 AlphaFold 3 model (1.695 compared to 1.775), however its QMEAN score is slightly lower (0.7 compared to 0.73). Overall, models based on templates 1-4 display strong similarity to the AlphaFold 3 TTC33 model. For those 4 proteins their sequence identity to TTC33 scores above 20, whereas the fifth model was produced around a protein displaying only 11.5 identity, which might explain a high RMSD to TTC33 AlphaFold 3.

Supplementary Table 1. List of the highest-ranking Swiss-Model templates for structural modeling of TTC33. The 'Template PDB code' column indicates the structure used as template, while the codes in parentheses indicate alternative models of the same protein. Those most often differ in ligand content. Only the highest-ranking model of the collection was analyzed. Alternative models are listed in order proposed by Swiss-Model.

RANK	Template PDB code<br><br>(variants)	Coverage (graphical: TTC33 region covered by model; numerical: identity coverage)	I (S)[1]	GMQE [template search (model)]	QMEAN	Method (Resol. Å)	Oligo-meric state<br><br>(ligand)	RMSD to TTC33 Alpha Fold3 model	Reference<br><br>DOI<br><br>(description)
1	8chy.1.A<br><br>(8ot7.1.A)	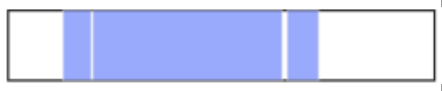<br>0.59	23.33 (0.32)	0.39 (0.41)	0.73±0.07	X-ray (2)	monomer<br><br>(13xZn)	1.78	Liutkus et al., 2024[S1]<br><br>https://doi.org/10.1002/pro.4971<br><br>(synthetic model 8 repeat TPR protein with zinc)
2	9qhc.1.A<br><br>(9qhc.2.A 9qhb.1.A)	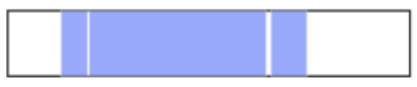<br>0.58	22.88 (0.32)	0.39 (0.40)	0.70±0.07	X-ray (2.88)	monomer<br><br>(1xCa)	1.7	Liutkus et al., 2025[S2]<br><br>https://doi.org/10.1002/anie.202516635<br><br>(natural: 8-repeat CTPR protein; *Escherichia coli*)
3	5npr.1.A	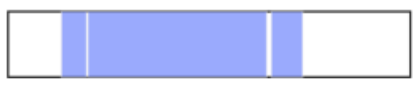<br>0.57	22 (0.31)	0.37 (0.39)	0.68±0.07	X-ray (1.8)	monomer<br><br>1xLig.[2]	1.91	Rafie et al., 2018[S3]<br><br>https://doi.org/10.1021/acs.bioconjchem.8b00194<br><br>(natural: O-GlcNAc transferase; *Homo sapiens*)
4	4j8d.1.A	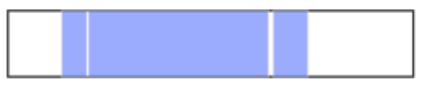<br>0.58	21.19 (0.31)	0.36 (0.39)	0.67±0.07	X-ray (2.8)	monomer<br><br>(none)	2.01	Li et al., 2013[S4]<br><br>https://doi.org/10.1038/nsmb.2608<br><br>(natural: Hsc70-interacting protein; *Rattus norvegicus*)
5	4n5c.4.A<br><br>(4n5c.2.A 4n5c.3.A 4n5c.1.A 4n5c.7.A 4n5c.5.A 4n5c.6.A 4n5c.8.A)	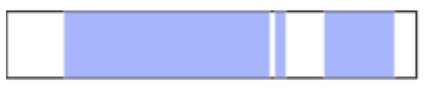<br>0.76	11.50 (0.25)	0.38 (0.35)	0.48±0.06	X-ray, (3.3)	monomer<br><br>(none)	18.35	Wu et al., 2014[S5]<br><br>https://doi.org/10.1016/j.devcel.2013.11.012<br><br>(natural: Ypp1; *Saccharomyces cerevisiae* S288C)

[1] I – Identity, S- Similarity

[2] Lig. - [[(2~{R},3~{S},4~{R},5~{R})-5-[2,4-bis(oxidanylidene)pyrimidin-1-yl]-3,4-bis(oxidanyl)oxolan-2-yl]methoxy-oxidanyl-phosphoryl] propyl hydrogen phosphate

Supplementary References:

S1 Liutkus, M., Sasselli, I. R., Rojas, A. L. & Cortajarena, A. L. Diverse crystalline protein scaffolds through metal-dependent polymorphism. *Protein Sci.* **33**, e4971 (2024).

S2 Liutkus M., Rojas A. L. & Cortajarena A. L. Rational Crystal Contact Engineering for Programmable Self-Assembled Protein Architectures. *Angew. Chem. Int. Ed. Engl.* **64**, e202516635 (2025).

S3 Rafie K., Gorelik A., Trapannone R., Borodkin V. S. & van Aalten D. M. F. Thio-Linked UDP-Peptide Conjugates as O-GlcNAc Transferase Inhibitors. *Bioconjug. Chem.* **29**, 1834–1840. (2018).

S4 Li Z., Hartl F. U. & Bracher A. Structure and function of Hip, an attenuator of the Hsp70 chaperone cycle. *Nat. Struct. Mol. Biol.* **20**, 929–935 (2013).

S5 Wu X. et al. Structural insights into assembly and regulation of the plasma membrane phosphatidylinositol 4-kinase complex. *Dev. Cell* **28**, 19–29 (2014).

EDITORIAL CHANGES TO FIT THE FORMATTING REQUIREMENTS

1. Due to Reviewer 3 request, to simplify the introduction we removed two citations.

Maynard, S., Schurman, S. H., Harboe, C., de Souza-Pinto, N. C. & Bohr, V. A. Base excision repair of oxidative DNA damage and association with cancer and aging. Carcinogenesis 30, 2–10 (2009).

Poetsch, A. R. The genomics of oxidative DNA damage, repair, and resulting mutagenesis. Comput. Struct. Biotechnol. J. 18, 207–219 (2020).

2. To reduce word count, we simplified the following sentences:

The changes listed in the table below aimed to reduce word count without altering the meaning of the sentence

SECTION	ORIGINAL LINE	SHORTENED VERSION
INTRODUCTION	Following base excision, an apurinic/apyrimidinic (AP) site is formed that is a substrate for APE1-mediated endonucleolytic cleavage.	Base excision creates an apurinic/apyrimidinic (AP) site, which is endonucleolytically cleaved by APE1.
INTRODUCTION	DNA lesions activate the phosphoinositide-3-kinase-related protein kinases (PIKK kinases)	DNA lesions activate the phosphoinositide-3-kinase-related protein (PIKK) kinases
INTRODUCTION	PIKK kinases also modify p53 protein at various sites. p53 is a short-lived protein due to MDM2-mediated ubiquitylation. p53 phosphorylation stabilizes the protein, leading to its activation as a transcription factor. Phosphorylated p53 promotes transcription of p21 cyclin inhibitor, leading to cell cycle arrest. If DNA damage is unresolved, it activates transcription of pro-apoptotic genes (Aubrey et al., 2018). The combination of p53 modifications dictates the set of genes activated, and ultimately cell survival or apoptosis. In particular, p53-serine15 phosphorylation (p53-S15P) induces cell cycle arrest related to detection of DNA damage (Loughery et al., 2014).	PIKK kinases also modify p53 at various sites. p53 is short-lived due to MDM2-mediated ubiquitylation. p53 phosphorylation stabilizes the protein, activating it as a transcription factor. Phosphorylated p53 promotes transcription of p21 cyclin inhibitor, causing cell cycle arrest, and if DNA damage persists, it stimulates expression of pro-apoptotic genes (Aubrey et al., 2018). The combination of p53 modifications determines which genes are transcribed, ultimately influencing cell survival or apoptosis. In particular, p53-serine15 phosphorylation (p53-S15P) induces DNA damage-related cell cycle arrest (Loughery et al., 2014).
RESULTS (first section title)	Specific to bony vertebrates TTC33 is composed of conserved TPR repeats	TTC33 is composed of conserved TPR repeats
RESULTS	We sought to define the list of TTC33 protein interactors using mass spectrometry. We cloned the human TTC33 fused to either a C-terminal EGFP or N-terminal MBP into expression vectors, which were used to establish stable adherent HEK293 or HeLa Flp-In T-REx cell lines (henceforth simply called HEK293	To identify TTC33 protein interactors by mass spectrometry, we generated stable HEK293 and HeLa Flp-In T-REx cell lines (hereafter HEK293 and HeLa, respectively) expressing human TTC33 fused to C-terminal EGFP or N-terminal MBP under a tetracycline-inducible promoter. In addition, TTC33-EGFP was transiently expressed in FreeStyle 293-F suspension

	and HeLa, respectively) producing fusion proteins under the control of a tetracycline-inducible promotercytacjaR19 (Szczęsny et al., 2018).To identify TTC33 protein interactors by mass spectrometry, we generated stable HEK293 and HeLa Flp-In T-REx cell lines (hereafter HEK293 and HeLa, respectively) expressing human TTC33 fused to C-terminal EGFP or N-terminal MBP under a tetracycline-inducible promoter (Szczęsny et al., 2018). In addition, TTC33-EGFP was transiently expressed in FreeStyle 293-F suspension cells, frequently used for protein purifications.	cells.
RESULTS	The final interactor, CCDC97, is encoded by yet another uncharacterized gene, found in bony vertebrates.	The final interactor, CCDC97, is encoded by yet another bony vertebrate uncharacterized gene.
RESULTS	To corroborate TANC formation *in vivo*, we first separated a whole cell protein extract from non-transfected FreeStyle 293-F cells in a 5-20% glycerol gradient. This showed TTC33 and CCDC97 co-migration	To confirm TANC formation in vivo, whole-cell extracts from non-transfected FreeStyle 293-F cells were separated on a 5–20% glycerol gradient, showing TTC33 and CCDC97 co-migration
RESULTS	We therefore performed the same analysis on extracts from FreeStyle 293-F cells over-expressing all TANC subunits simultaneously and observed much better co-migration (Fig. 5d).	However, the same analysis on extracts from FreeStyle 293-F cells over-expressing all TANC subunits simultaneously and observed much better co-migration (Fig. 5d).
RESULTS	In such a manner, subunits of PP2A phosphatase were also identified. This heterotrimeric enzyme exists in multiple isoforms composed of either one of the sixteen regulatory subunits, associated with a scaffolding and a catalytic protein. The latter two are each represented by two variants	PP2A subunits were also identified. This heterotrimeric enzyme exists in multiple isoforms with one of sixteen regulatory subunits plus scaffolding and catalytic proteins, each present in two variants
RESULTS	The TTC33 C-terminus was the most prone to conformational changes	The TTC33 C-terminus was most conformationally flexible
RESULTS	Altogether, we obtained a structural model of TTC33:WDR61:PHF5A trimer of reasonable quality for further validations. Notably, this model supported the results of our biochemical analyses.	Altogether, we obtained a reasonable structural model of the TTC33:WDR61:PHF5A trimer for further validation, which supported our biochemical results.

RESULTS	We also designed a series of TTC33 point mutants by selecting from a catalogue of rare population variants and cancer somatic mutations (Fig. 7h; Supplementary Fig. 5b). Alternatively, or if naturally occurring variants were not reported for a given residue, we introduced a glycine or alanine instead.	We also designed TTC33 point mutants based on rare population variants and cancer-associated somatic mutations (Fig. 7h; Supplementary Fig. 5b). If no natural variants were reported at a given residue, we substituted glycine or alanine.
RESULTS	TAN depletion in HEK293 cells increased the levels of DNA damage. We hypothesized that on an organismal level this could increase mutagenesis and accelerate carcinogenesis. We thus turned to analysis of RNA-sequencing datasets from The Cancer Genome Atlas (Liu et al., 2018) and revealed that *TTC33* expression decreased with cancer stage in patients with Kidney Renal Clear Cell Carcinoma (KIRC; Fig.10g). Regression analysis of TCGA data also demonstrated that higher expression of all TAN factors was positively correlated with roughly 2-times longer overall survival and, for most TAN factors, a higher 5-year survival rate among patients with KIRC (Fig. 10h; Supplementary Fig. 9b; Supplementary Dataset 4). This suggests that TAN factor expression may collectively serve as a potential prognostic indicator in KIRC.	TAN depletion in HEK293 cells increased genomic instability, suggesting that its loss may enhance mutagenesis and accelerate carcinogenesis *in vivo*. Analysis of RNA-sequencing datasets from The Cancer Genome Atlas (Liu et al., 2018) revealed that *TTC33* expression decreased with cancer stage in patients with KIRC (Fig.10h). Regression analysis further revealed that higher expression of all TAN factors was positively correlated with roughly 2-times longer overall survival and, for most TAN factors, improved 5-year survival rate among patients with KIRC (Fig. 10i; Supplementary Fig. 9f; Supplementary Dataset 4). This suggests that TAN factor expression may collectively serve as a potential prognostic indicator in KIRC.
DISCUSSION	It might be so,	Likely so,
DISCUSSION	Many of the variants we tested were reported as somatic cancer SNPs of uncertain significance, and in this work we showed that they have the capacity to disrupt normal cellular protein networks.	Many of the variants we tested, previously reported as somatic cancer SNPs of uncertain significance, were shown here to disrupt normal cellular protein networks.
DISCUSSION	In addition, PHF5A together with p400 chaperone mediates deposition of γH2AX to promote NHEJ at the antibody locus during class switch recombination	PHF5A together with p400 chaperone mediates deposition of γH2AX to promote NHEJ at the antibody locus during class switch recombination
DISCUSSION	In light of future follow-up structural studies, removal of the TTC33 N-terminal helical region and the unstructured C-terminus is warranted **in order to** produce a more globular and easily purifiable protein.	In light of future follow-up structural studies, removal of the TTC33 N-terminal helical region and the unstructured C-terminus is warranted to produce a more globular and easily purifiable protein.
DISCUSSION	Mass spectrometry analysis is known for its high sensitivity, which is useful for the identification of weak protein	Mass spectrometry is highly sensitive, which is useful for the identification of weak interactions, but also generates many

	interactions, but generates many false-positive hits. This ultimately obscures the identification of stable, functionally relevant interactions.	false-positives that obscure the identification of stable, functionally relevant interactions.
DISCUSSION	UNG1 was shown to associate with PRDX3 to physically coat the mitochondrial DNA, protecting it from ROS	UNG1 associates with PRDX3 to physically coat the mitochondrial DNA, protecting it from ROS
DISCUSSION	In TAN-deficient cells, DNA DSBs accumulate spontaneously, but the mechanism is unclear.	DNA DSBs accumulate spontaneously in TAN-deficient cells, though the mechanism is unclear.
DISCUSSION	In sum, future efforts directed at deciphering the exact TAN function will focus on eliminating a plethora of such hypotheses.	In sum, future efforts to define the TAN function will aim to narrow down these hypotheses.

POINT-BY-POINT RESPONSE

Revision round 3

***

TTC33 forms a complex with WDR61 and PHF5A to recruit factors relevant to genomic integrity

Rafał Tomecki[1,2,#], Małgorzata Drabko[1,3#], Małgorzata Siek[1,3], Maria Matykiewicz[1], Miłosz Ludwinek[1,‡], Łukasz S. Borowski[4,5], Aneta Jurkiewicz[4], Kamil Kobyłecki[1], Agnieszka Jabłońska[1], Dominik Cysewski[6,§], Agata Malinowska[6], Magdalena Bakun[6], Rafał Płoski[7], Roman J. Szczęsny[4], and Agnieszka Tudek[1,*]

[1] Laboratory of RNA Processing and Decay, Institute of Biochemistry and Biophysics, Polish Academy of Sciences, A. Pawińskiego 5A, 02-106 Warsaw, Poland

[2] Laboratory of Epitranscriptomics, Institute of Bioengineering, Faculty of Biology, Biological and Chemical Research Centre, University of Warsaw, S. Banacha 2c, 02-097 Warsaw, Poland

[3] Doctoral School of Molecular Biology and Biological Chemistry at IBB PAS, Pawinskiego 5a, 02-106 Warsaw, Poland

[4] Laboratory of RNA Biology, Institute of Biochemistry and Biophysics, Polish Academy of Sciences, A. Pawińskiego 5A, 02-106 Warsaw, Poland

[5] Institute of Genetics and Biotechnology, Faculty of Biology, University of Warsaw, A. Pawińskiego 5A, 02-106 Warsaw, Poland

[6] Mass Spectrometry Laboratory, Institute of Biochemistry and Biophysics, Polish Academy of Sciences, A. Pawińskiego 5A, 02-106 Warsaw, Poland

[7] Department of Medical Genetics, Medical University of Warsaw, A. Pawińskiego 3c, 02-106 Warsaw, Poland

[‡] Present address: Chair and Department of Biochemistry, Medical University of Warsaw, S. Banacha 1, 02-097 Warsaw, Poland

[§] Present address: Medical University of Białystok, Clinical Research Centre, J. Kilińskiego 1, 15-089 Białystok, Poland

# These authors contributed equally to this work

* Correspondence and requests for materials should be addressed to atudek@ibb.waw.pl

***

TABLE OF CONTENTS

RESPONSE TO REVIEWER 3..................................page 02

RESPONSE TO REVIEWER 3

The authors have addressed all my concerns, and I support the publication in the current form.

We thank the Reviewer for the appreciation of our work and for the effort put into the revision process. The valuable comments have enabled us to substantially improve our work.

I feel there are only few minor points, that they may consider in the final version of the manuscript:

1- Line 64: DNA lesions "as Double Strand Breaks (DSBs) or single strand DNA" activate… as not all DNA lesionslead to PIKK kinase activation

Response to point 1: The sentence was revised, as requested: '*DNA lesions, such as DSBs and SSBs, activate the phosphoinositide-3-kinase-related protein (PIKK) kinases, which phosphorylate the H2AX variant of histone H2A at serine 139, leading to the formation of γH2AX.* '

2- Figure 2c-d: could the authors use the same order for both figures? It is difficult to follow where is what otherwise.

Response to point 2: This was corrected.

3- I still think that not all interactions listed in the TAN list (Figure 2c) are "positively validated" in Figure 2d, forexample the ones with PP2A-B55alpha and SF3A/B. The authors should tone this down as "mostly validated"

Response to point 3: The sentence was revised, as requested: '*We thus defined the TTC33-associated network (TAN) (Fig. 2c), and validated most interactions by co-immunoprecipitation coupled with western blot (coIP-WB) in HEK293 and HeLa (Fig. 2d).*'

4- Line 164: "likewise detected in the nucleus" is misleading, as TTC33 and CCDC97 are also in the cytoplasm. Thecorrect sentence would be "likewise detected in the nucleus and cytoplasm".

Response to point 4: The sentence was revised, to better reflect the data: '*Thus, TTC33 and CCDC97, similarly to WDR61, localize to both the nucleus and cytoplasm. Other TAN factors (SF3A/SF3B, UNG2, and PP2A-B55α) are predominantly nuclear (Fig. 3e; Supplementary Fig. 2b)*'

5- Line 389: I agree with the possible adaptation in cells O/E TTC33, as suggested by the authors in the rebuttal. Iwould add this hypothesis also in the text to better explain the shown phenotype.

Response to point 5: Sections of the results and discussion contained such conclusions or were improved, as requested. RESULTS: 'TTC33 over-expressing cells reached a similar maximal confluence as control cells, but did so later and over a more prolonged growth period (Fig. 10g), suggesting enhanced tolerance rather than resistance to doxorubicin.' DISCUSSION: 'At cytotoxic levels, commonly used in cancer therapies, TTC33 loss increased sensitivity, while its over-expression enabled longer cell survival and proliferation, albeit at a lower rate, suggesting a degree of cellular adaptation. '
