# Supplementary material for: TTC33 forms a complex with WDR61 and PHF5A to recruit factors relevant to genomic integrity
Source: Nat Commun. 2026 Aug 21;17:9984. doi: 10.1038/s41467-026-76928-z (PMC13590505; doi:10.1038/s41467-026-76928-z)

Figure 2 d. Immunoprecipitation of MBP-tagged TTC33 from 293 Flp-In T-REx and HeLa Flp-In T-REx stable cell lines in the absence or presence of RNase A

membranes after transfer stained with Ponceau S red

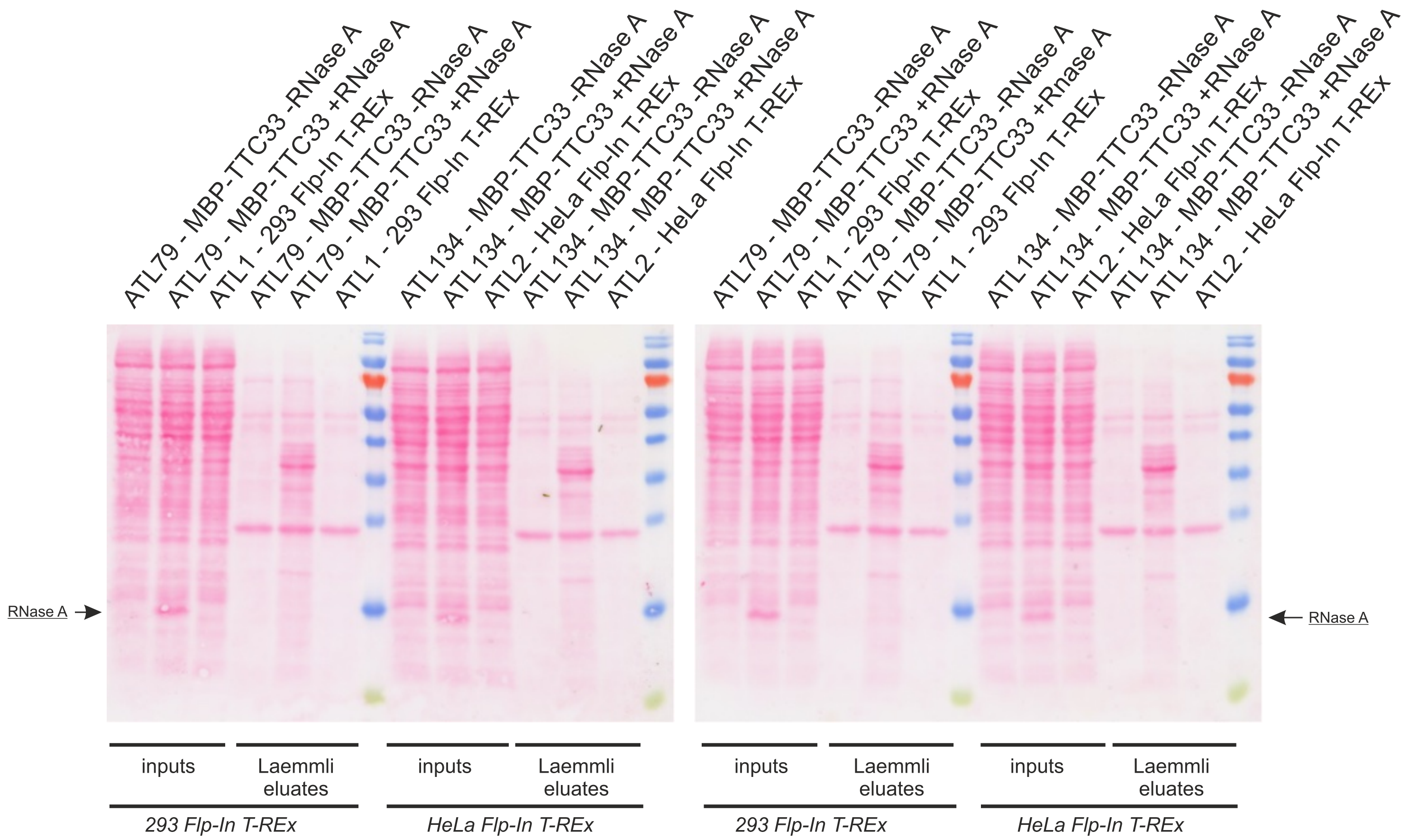

western-blot results (uncropped membranes)

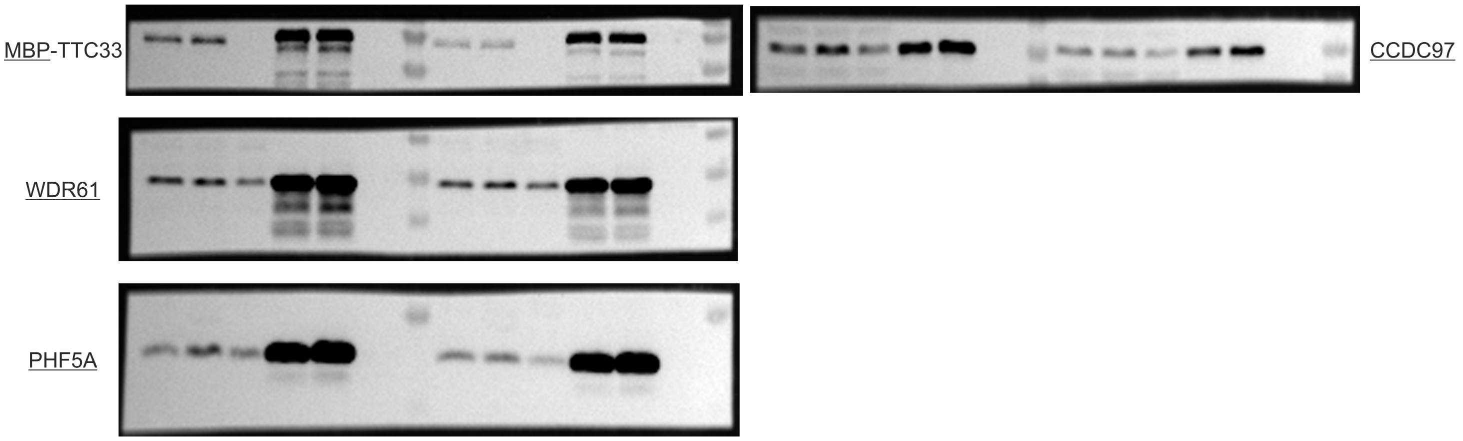

Figure 2 d. Immunoprecipitation of MBP-tagged TTC33 from 293 Flp-In T-REx and HeLa Flp-In T-REx stable cell lines in the absence or presence of RNase A (continued)

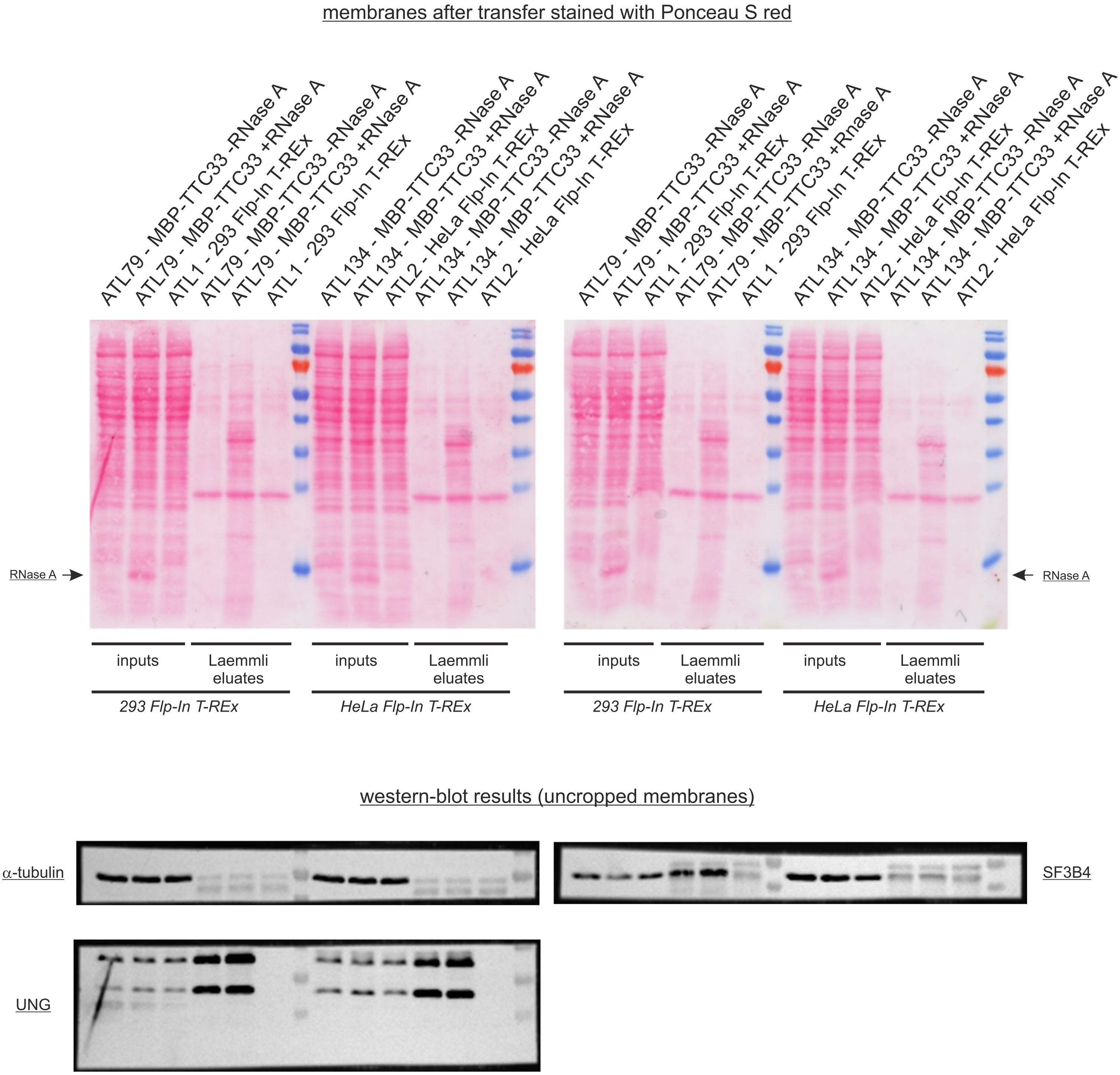

Figure 2 d. Immunoprecipitation of MBP-tagged TTC33 from 293 Flp-In T-REx and HeLa Flp-In T-REx stable cell lines in the absence or presence of RNase A (original iBright scans)

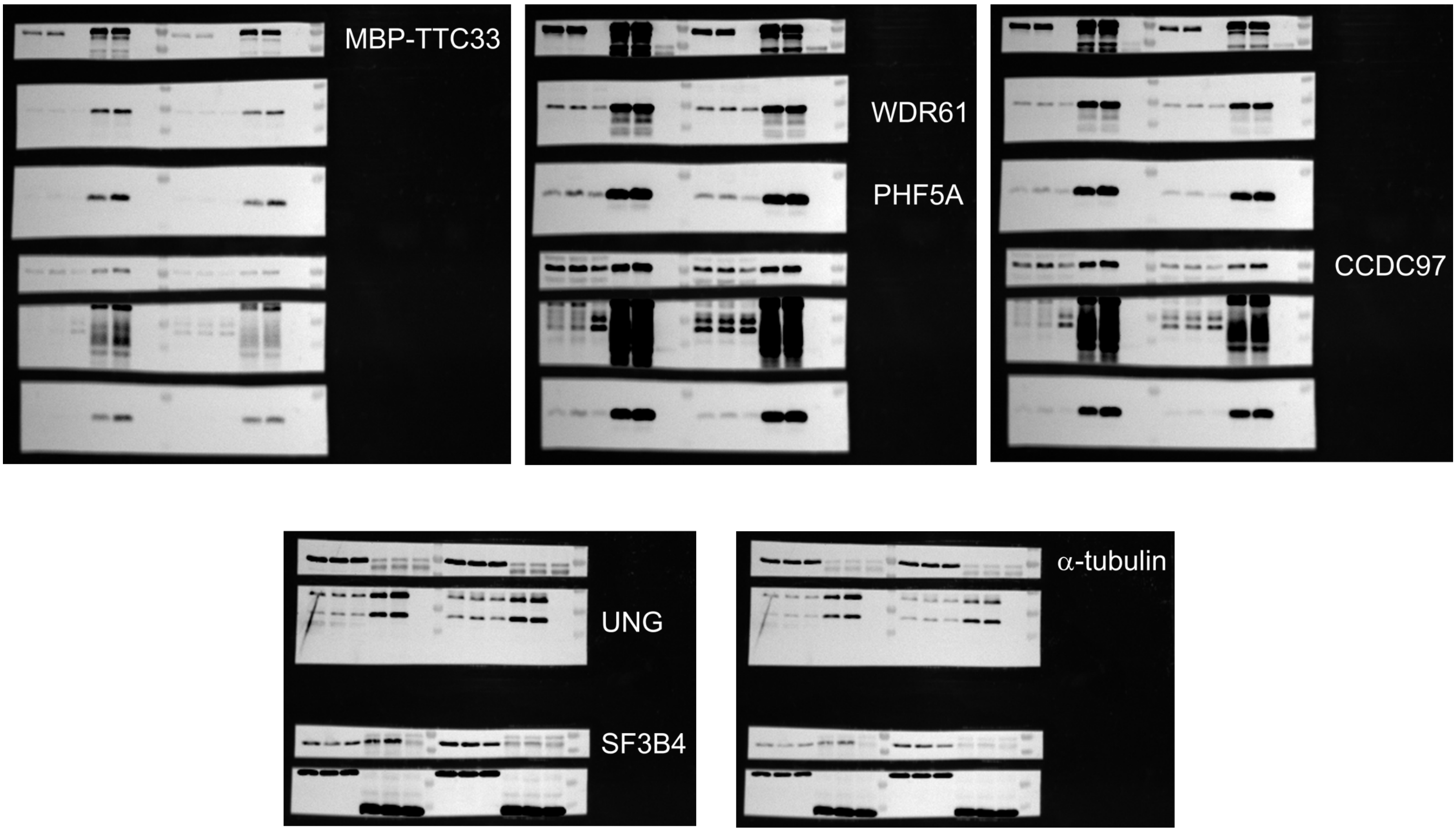

Figure 3 c. Cell fractionation experiment and western-blot analysis of subcellular fractions

membranes after transfer stained with Ponceau S red

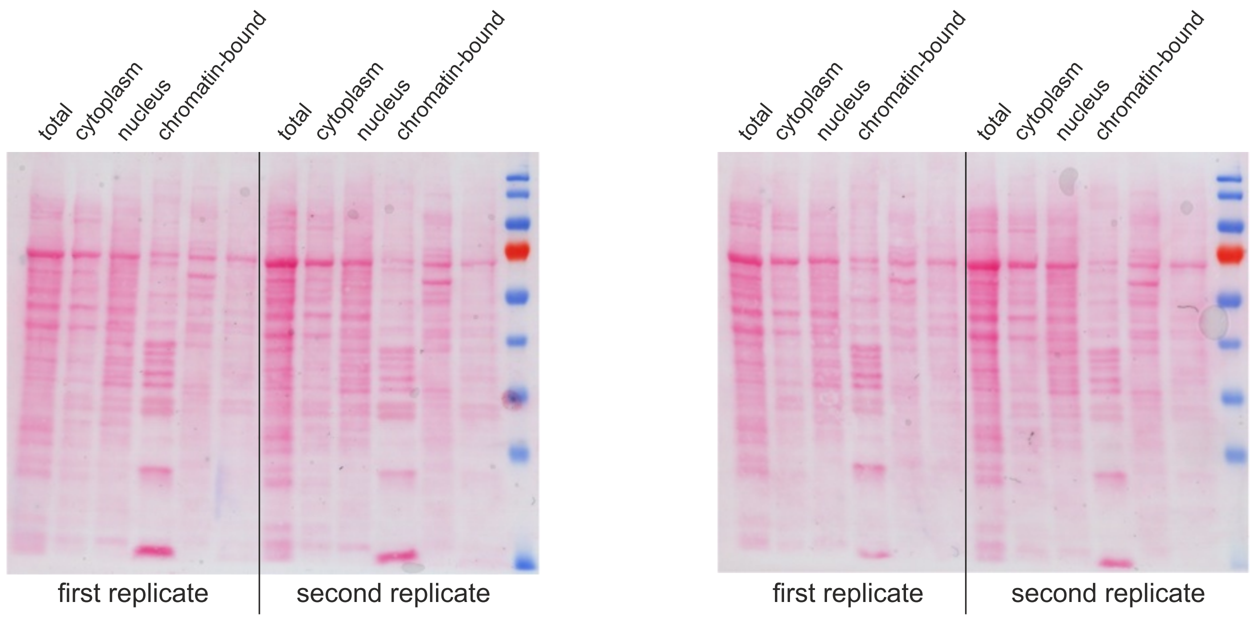

western-blot results (uncropped membranes)

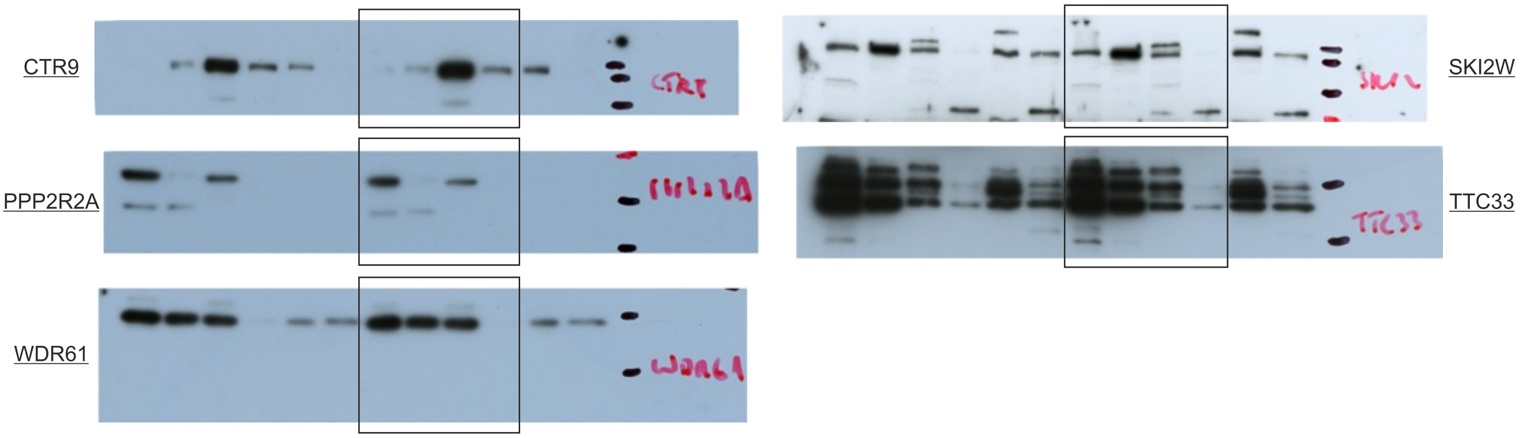

Figure 3 c. Cell fractionation experiment and western-blot analysis of subcellular fractions (continued)

membranes after transfer stained with Ponceau S red

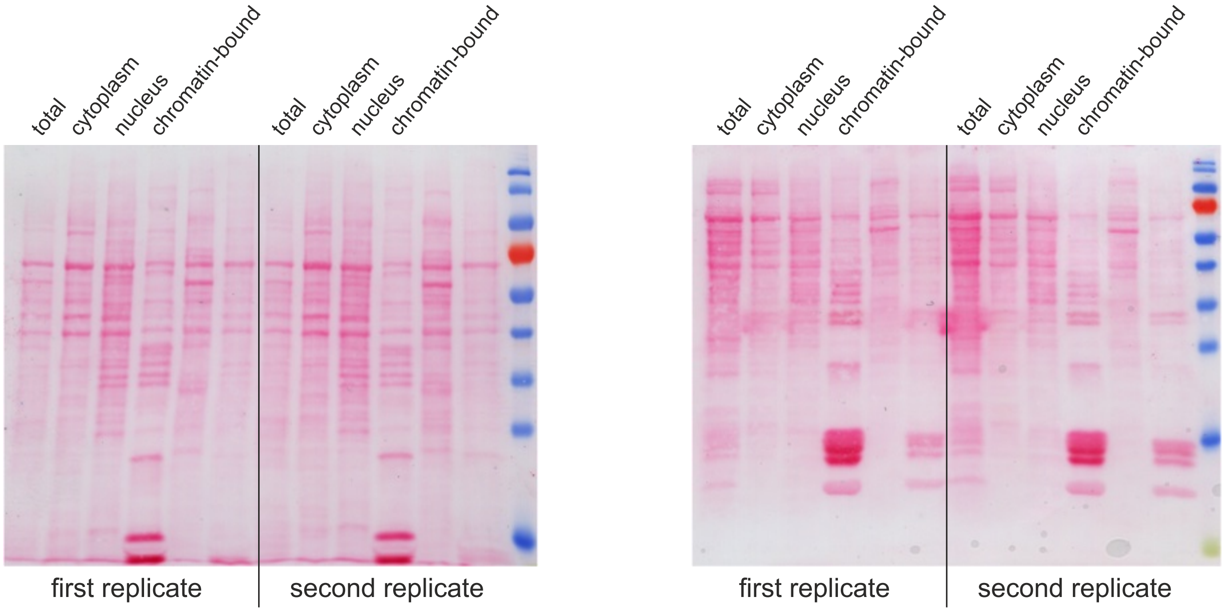

western-blot results (uncropped membranes)

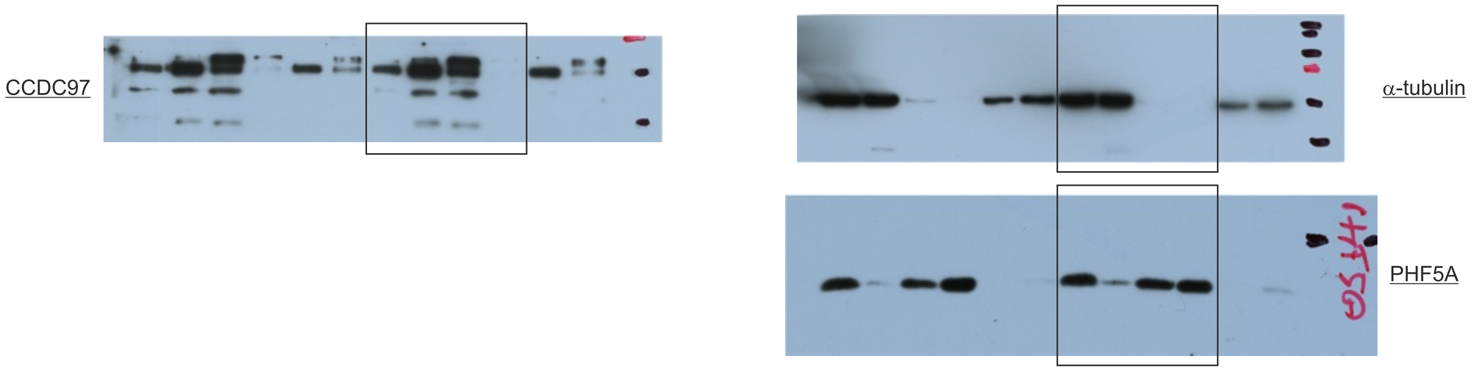

Figure 3 c. Cell fractionation experiment and western-blot analysis of subcellular fractions (continued)

membranes after transfer stained with Ponceau S red

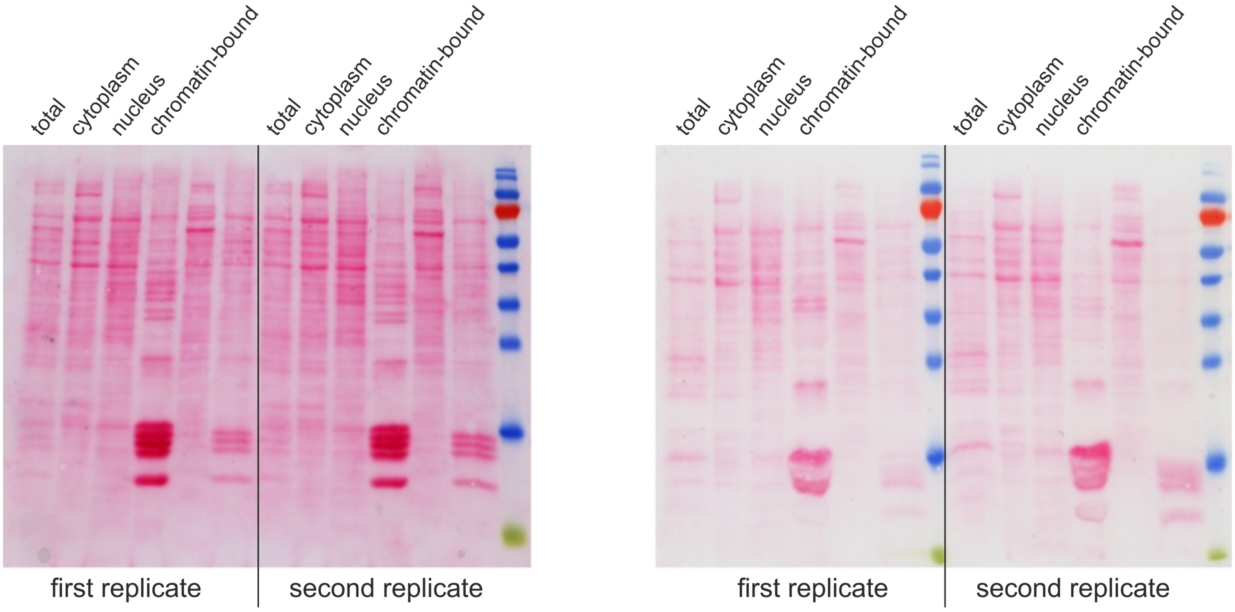

western-blot results (uncropped membranes)

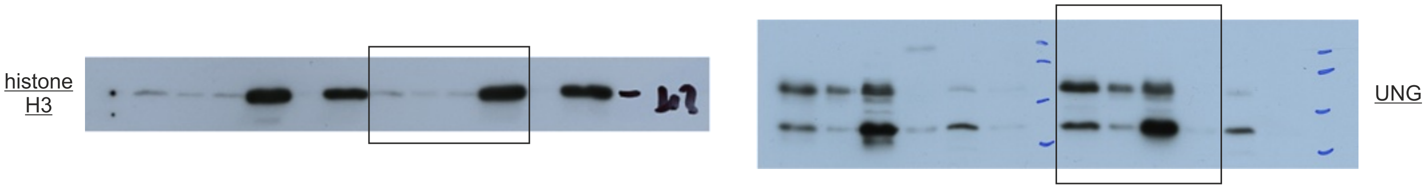

membrane after transfer stained with Ponceau S red

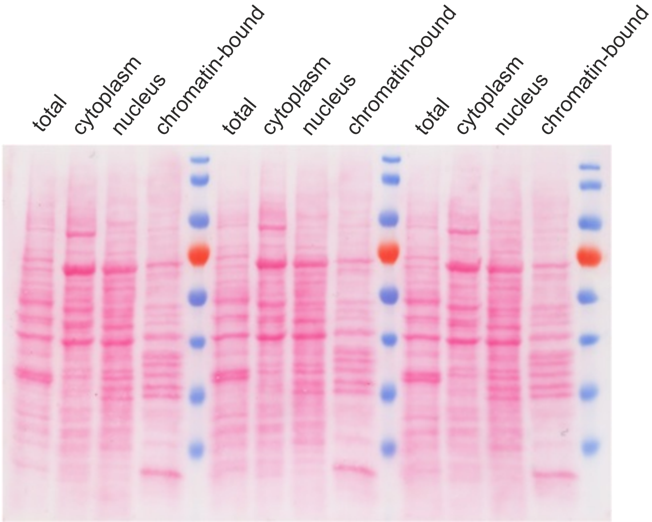

only second replicate run in technical triplicate

western-blot results (uncropped membrane)

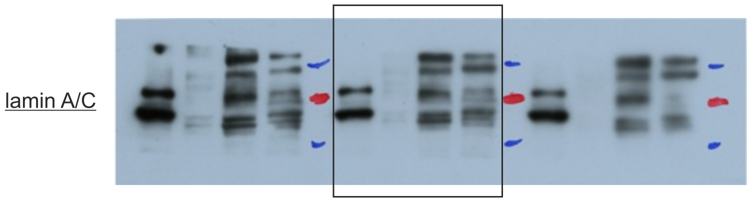

Figure 4 b. Immunoprecipitation of MBP-tagged PPP2R2A or TTC33 from 293 Flp-In T-REx and detection of endogenous WDR61

western-blot results (uncropped membranes)

MBP

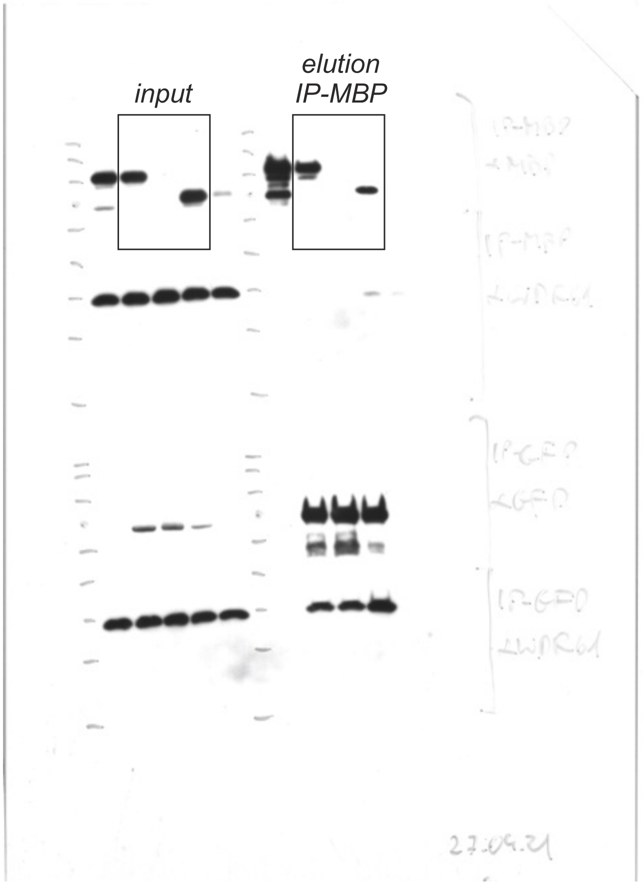

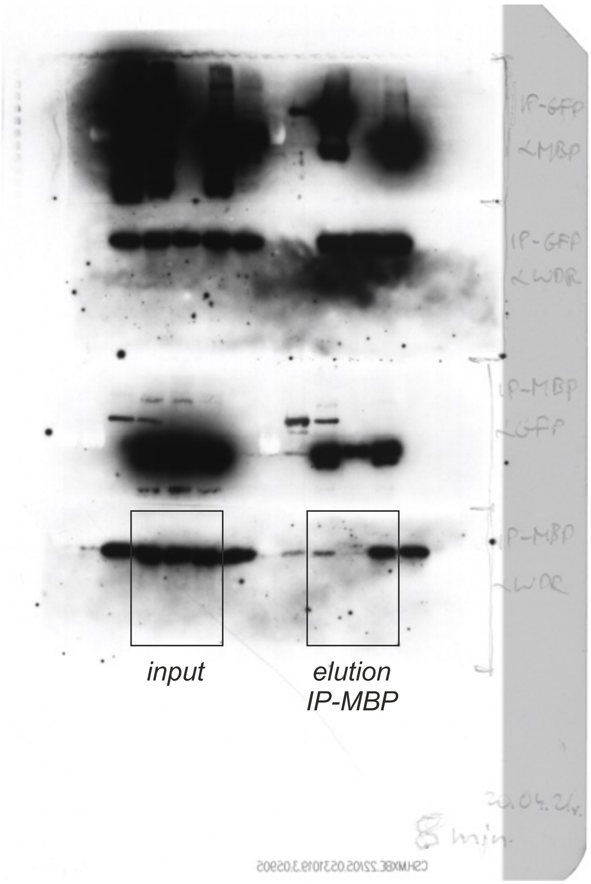

WDR61

Figure 4 e. Immunoprecipitation of EGFP-tagged CCDC97 from HeLa Flp-In T-REx stable cell line in the absence or presence of RNase A

membranes after transfer stained with Ponceau S red

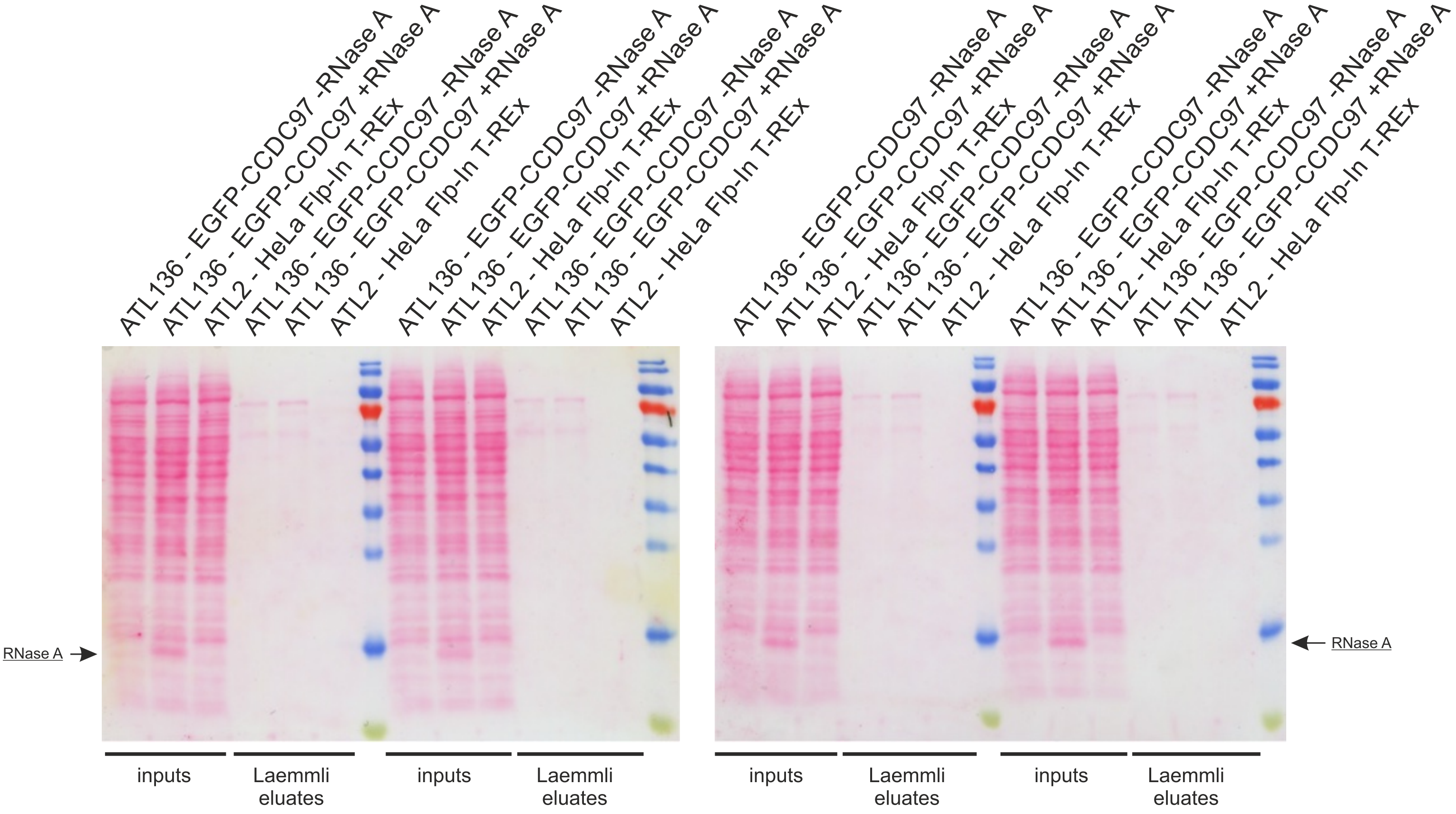

western-blot results (uncropped membranes)

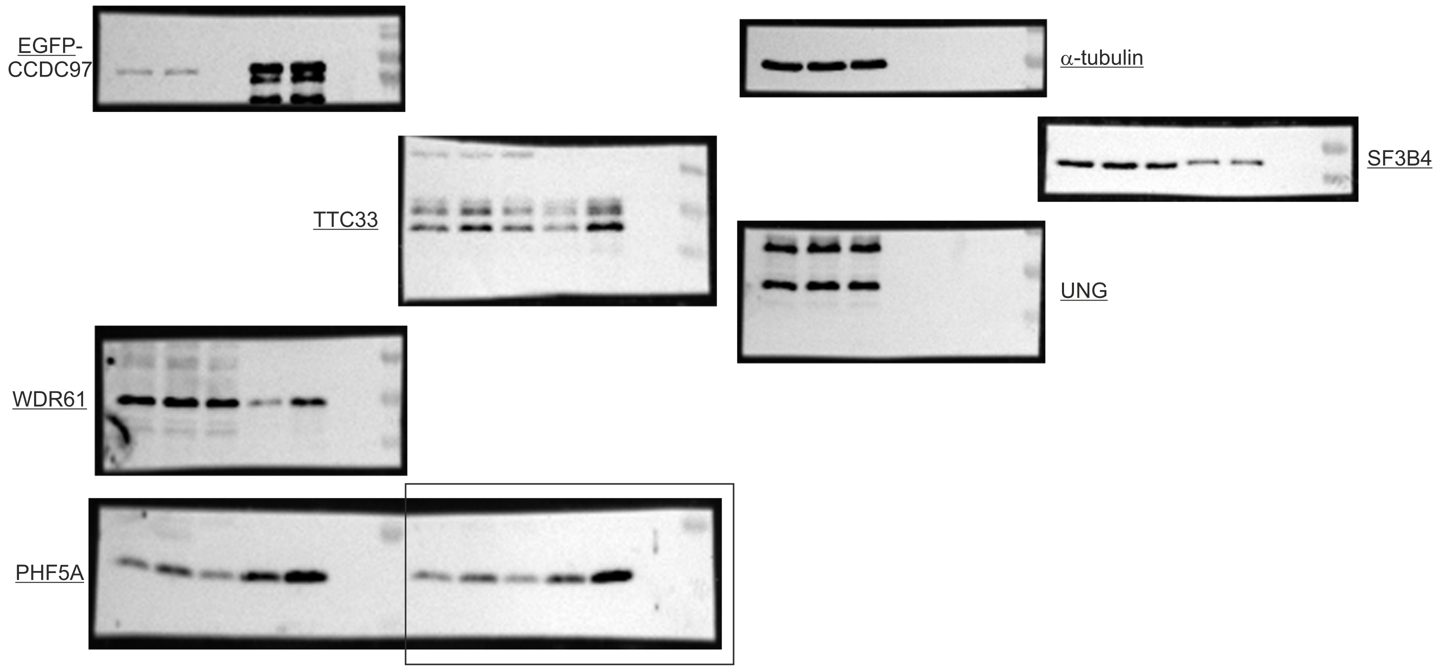

Figure 4 e. Immunoprecipitation of EGFP-tagged CCDC97 from HeLa Flp-In T-REx stable cell line in the absence or presence of RNase A (original iBright scans)

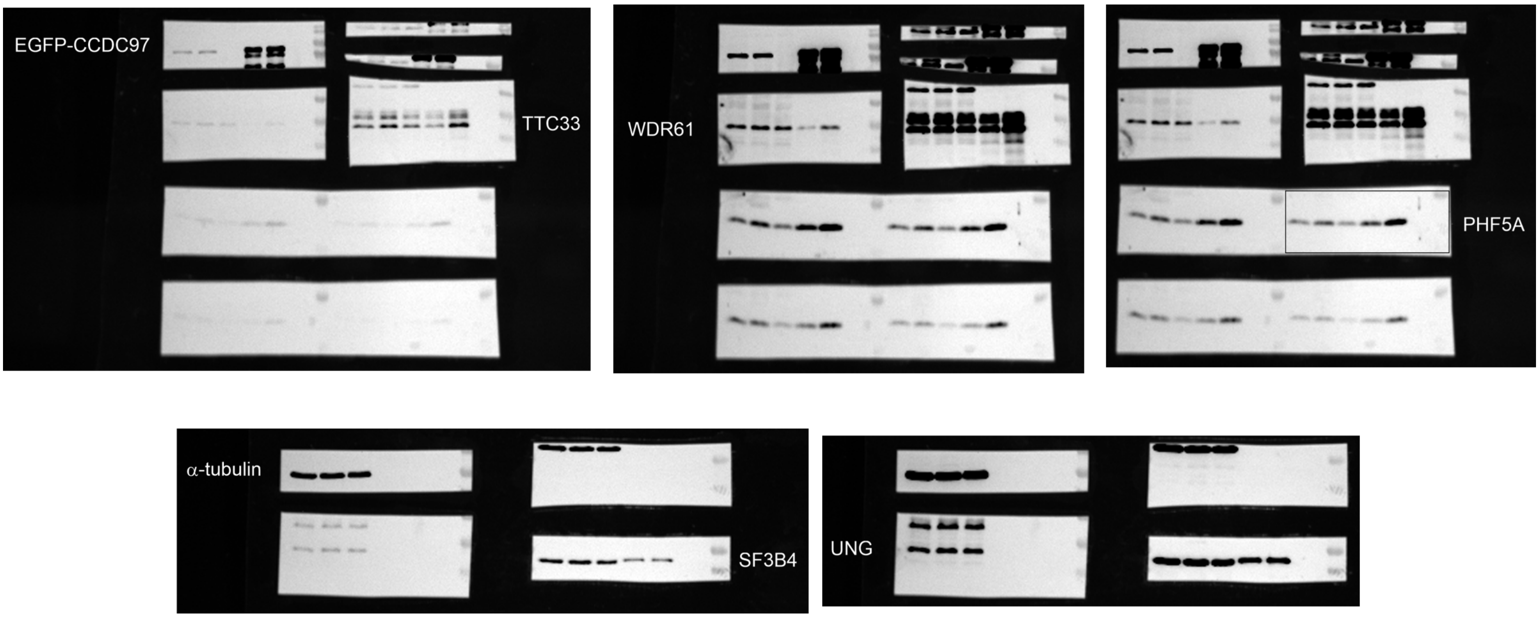

Figure 4 f. Immunoprecipitation of EGFP-tagged UNG1 or UNG2 from 293 Flp-In T-REx stable cell lines

membranes after transfer stained with Ponceau S red

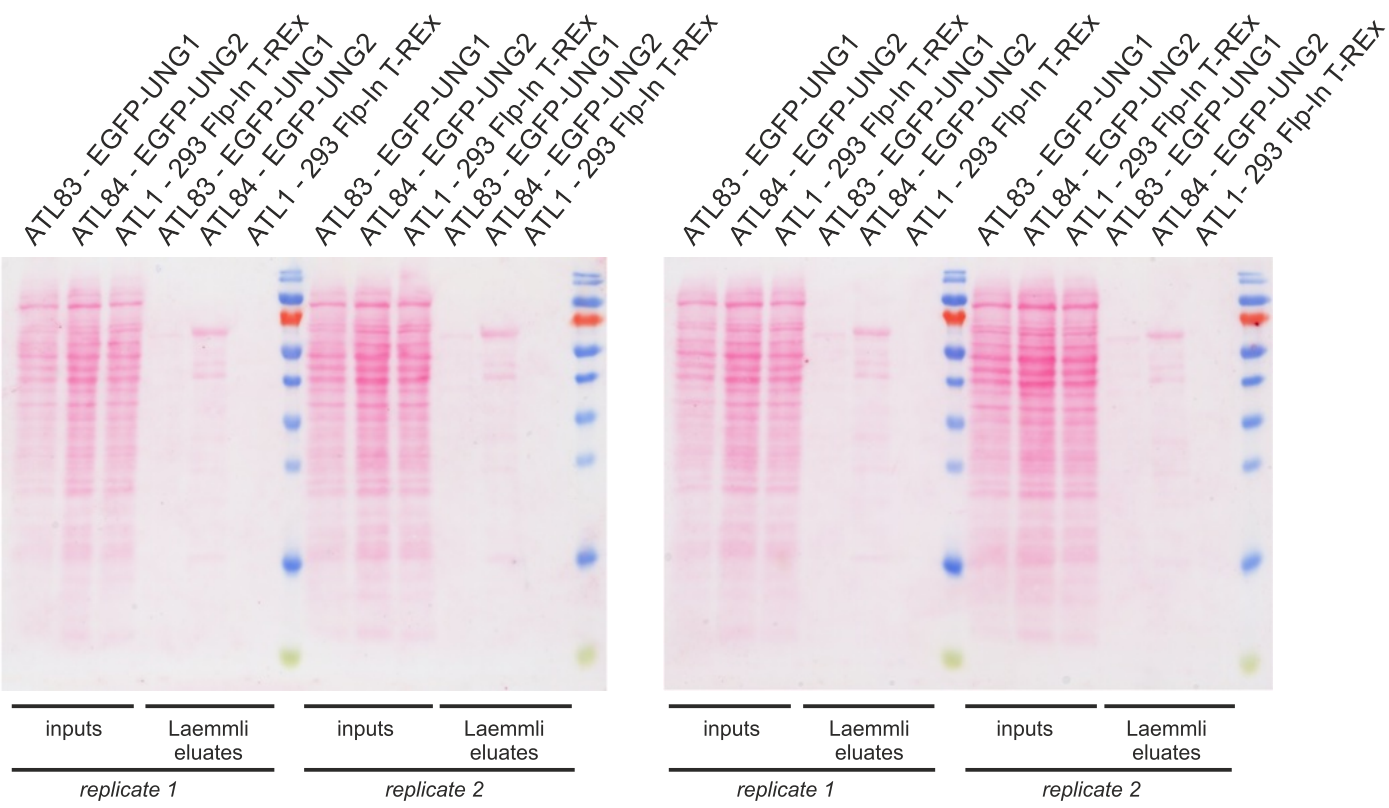

western-blot results (uncropped membranes)

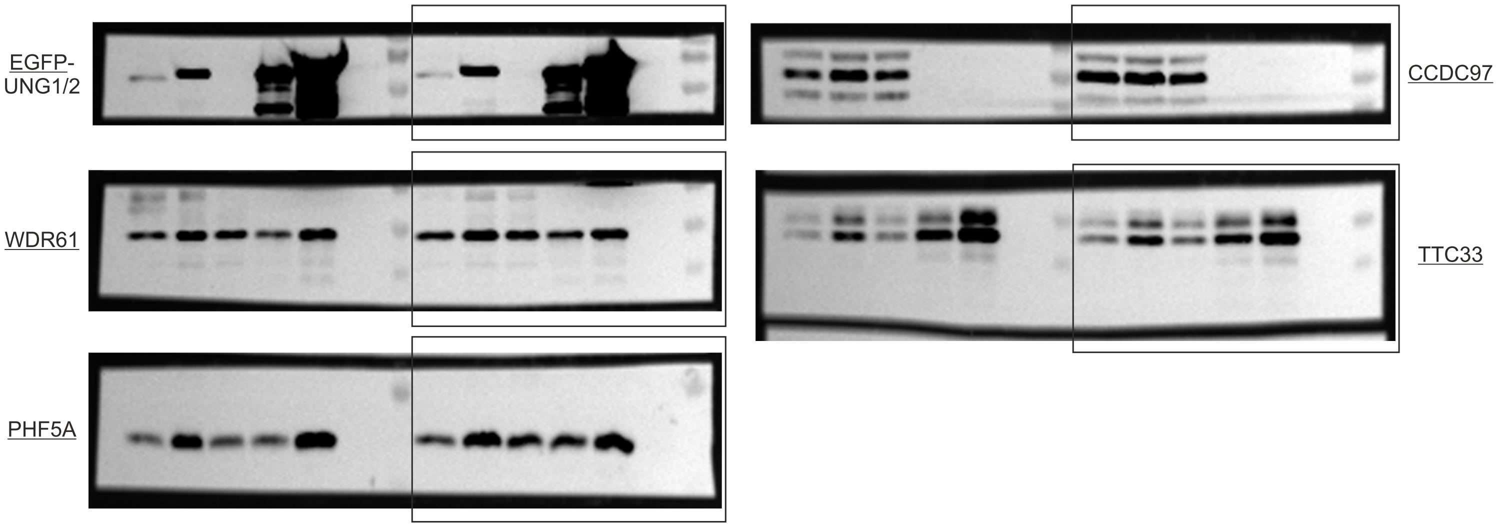

Figure 4 f. Immunoprecipitation of EGFP-tagged UNG1 or UNG2 from 293 Flp-In T-REx stable cell lines (continued)

membranes after transfer stained with Ponceau S red

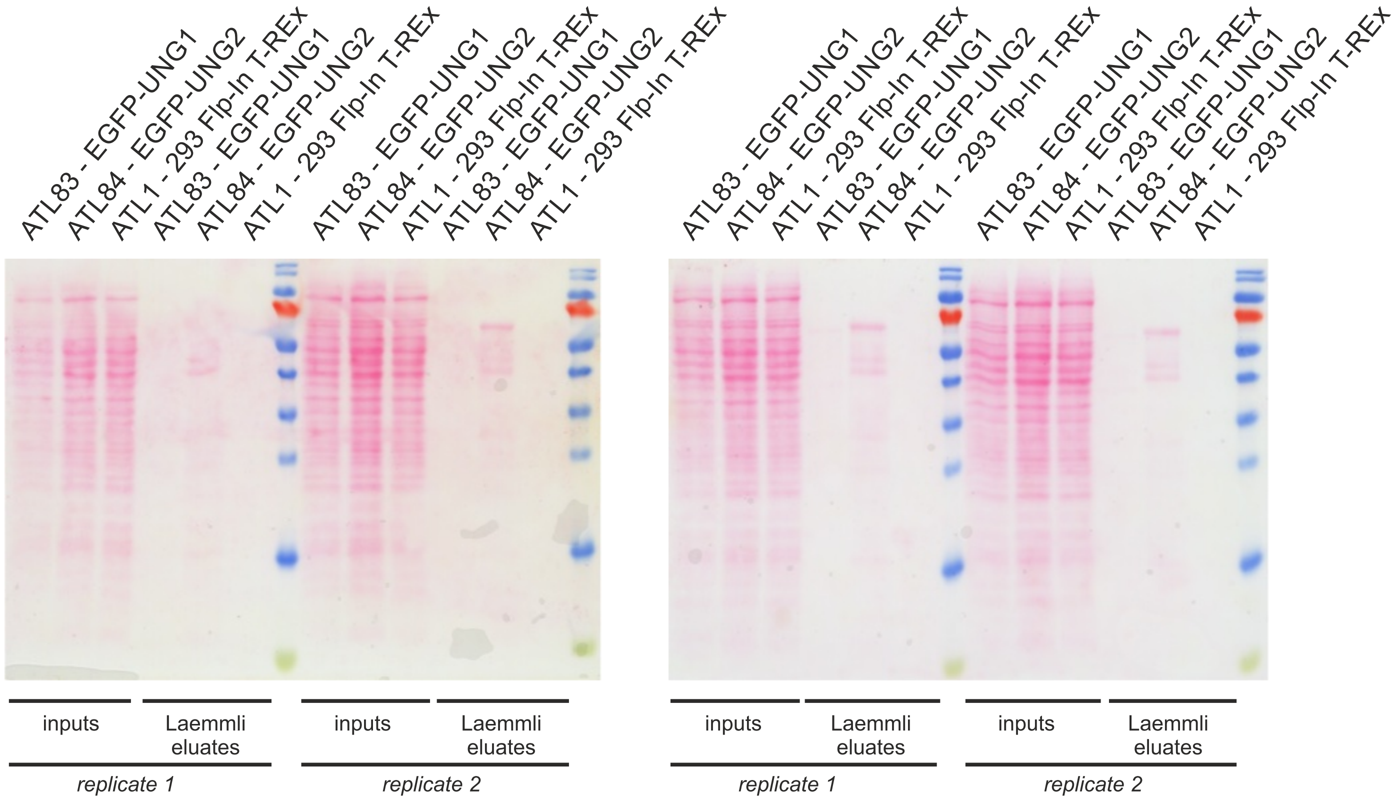

western-blot results (uncropped membranes)

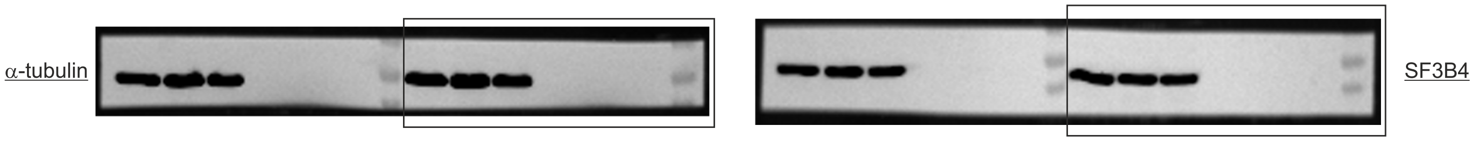

Figure 4 f. Immunoprecipitation of EGFP-tagged UNG1 or UNG2 from 293 Flp-In T-REx stable cell lines (original iBright scans)

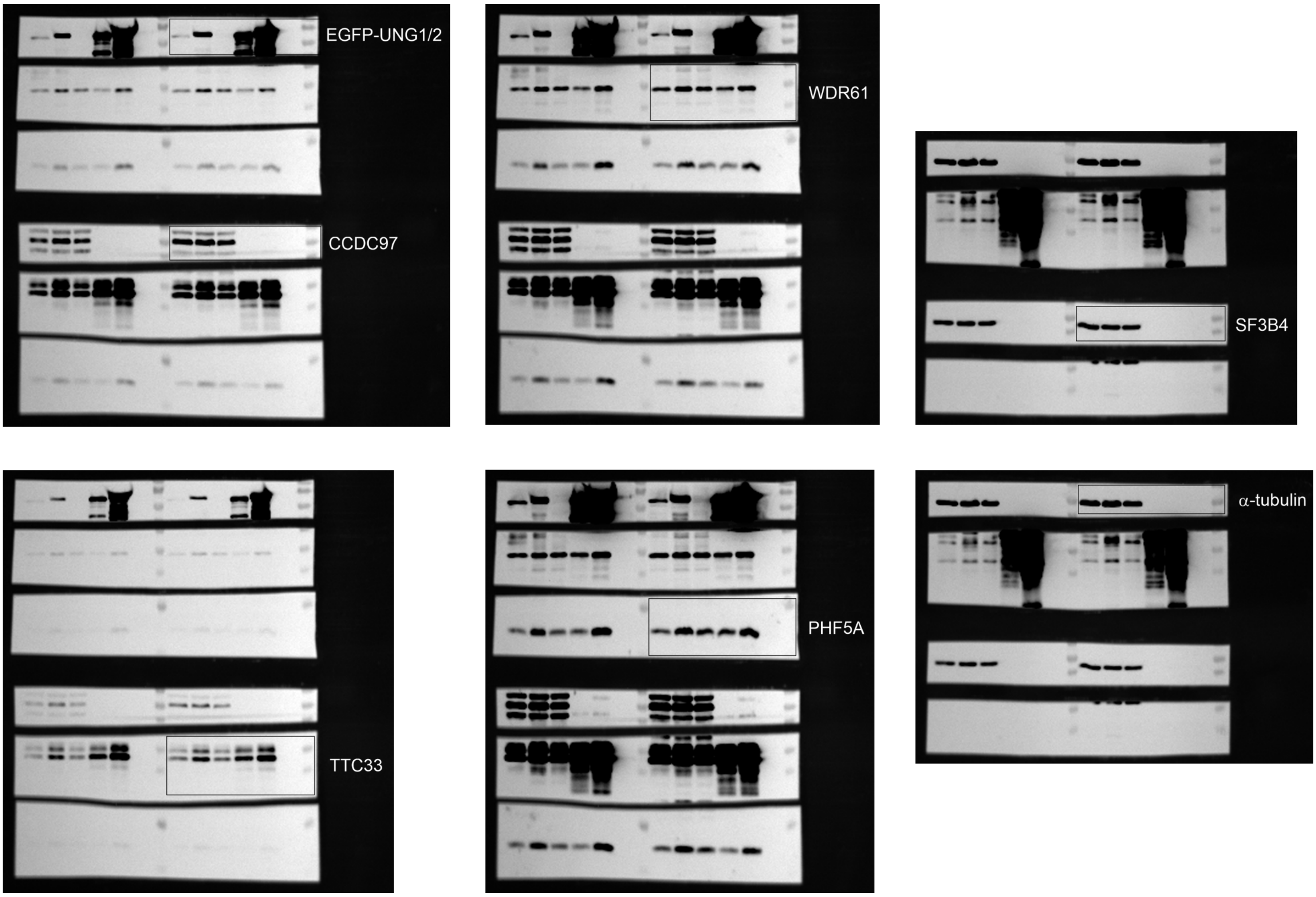

Figure 5 b. Testing the potential formation of a stable tetramer between TANC components and CCDC97 or UNG2

Coomassie-stained SDS-PAGE gel with eluates after affinity chromatography on amylose resin

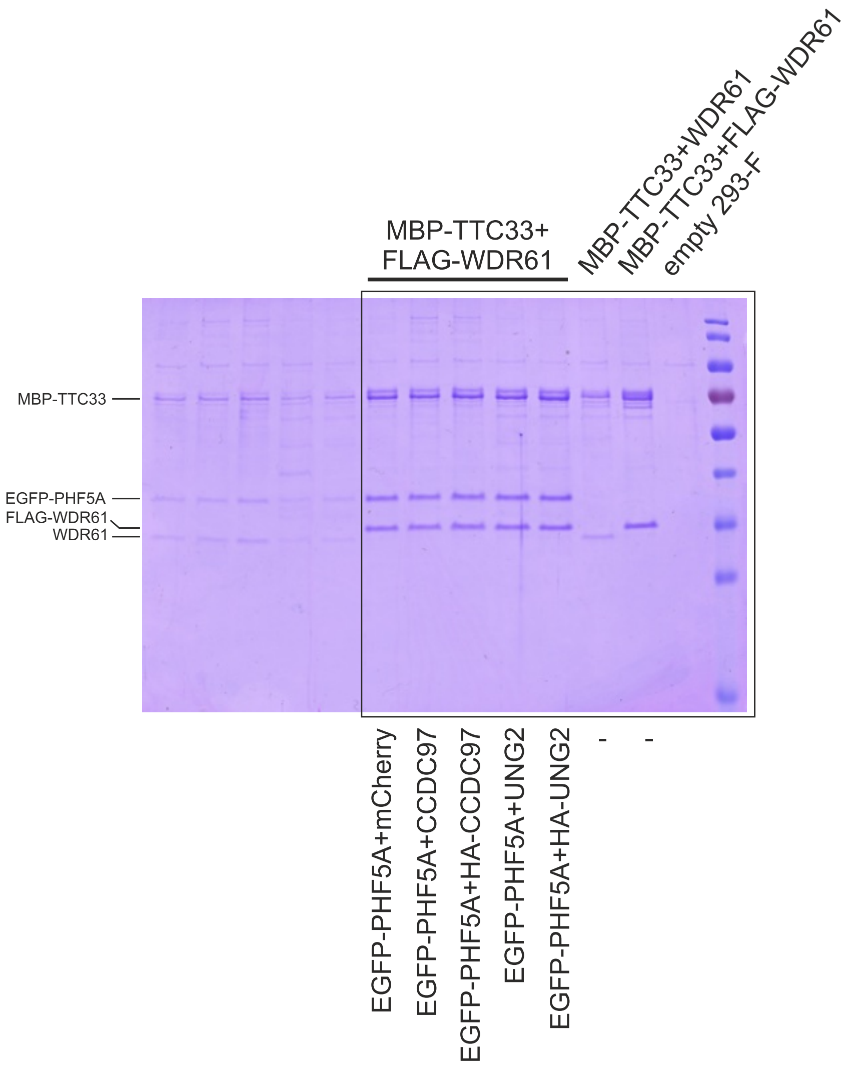

Figure 5 b. Testing the potential formation of a stable tetramer between TANC components and CCDC97 or UNG2 (continued)

membranes after transfer stained with Ponceau S red

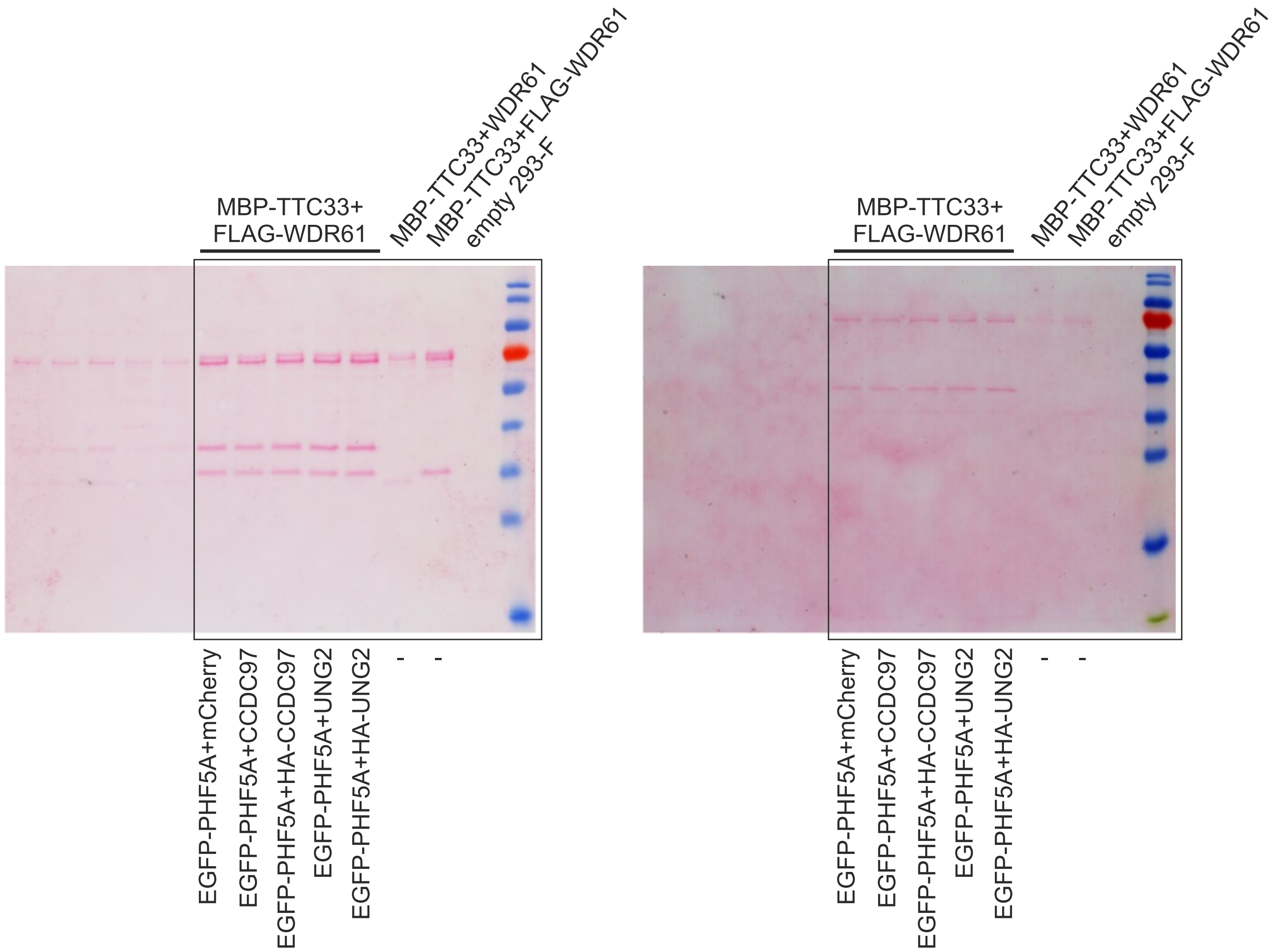

western-blot results (uncropped membranes)

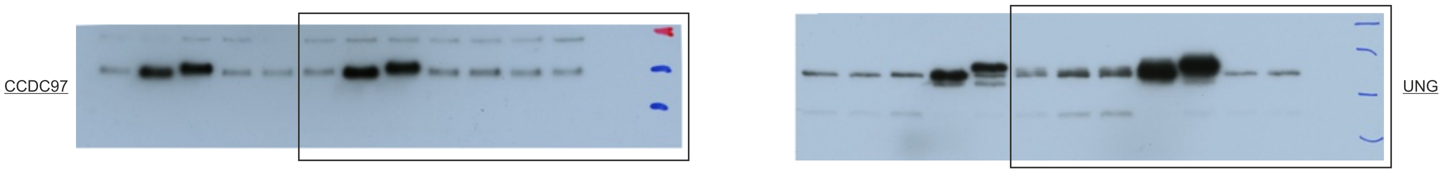

Figure 5 b. Testing the potential formation of a stable tetramer between TANC components and CCDC97 or UNG2 (continued)

membrane after transfer stained with Ponceau S red

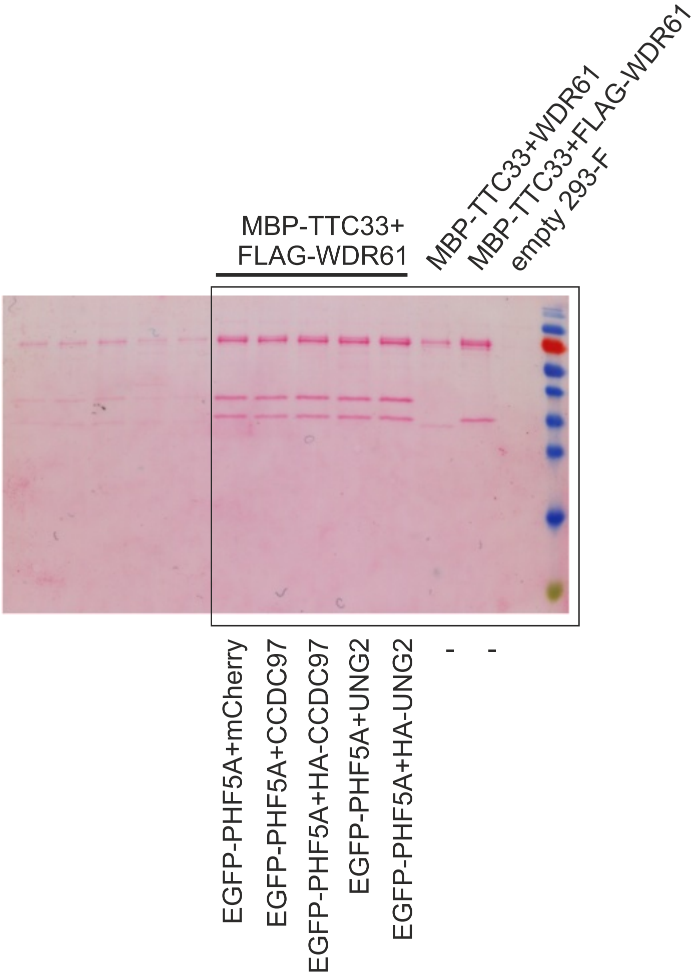

western-blot results (uncropped membrane)

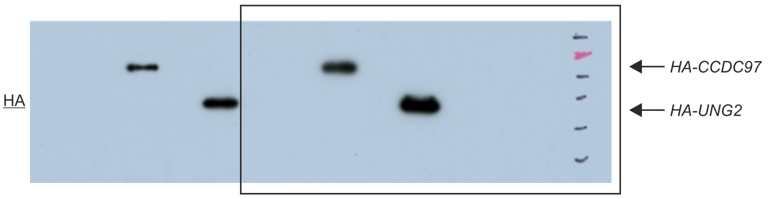

Figure 5 b. Testing the potential formation of a stable tetramer between TANC components and CCDC97 or UNG2 (original scans of gel and membranes)

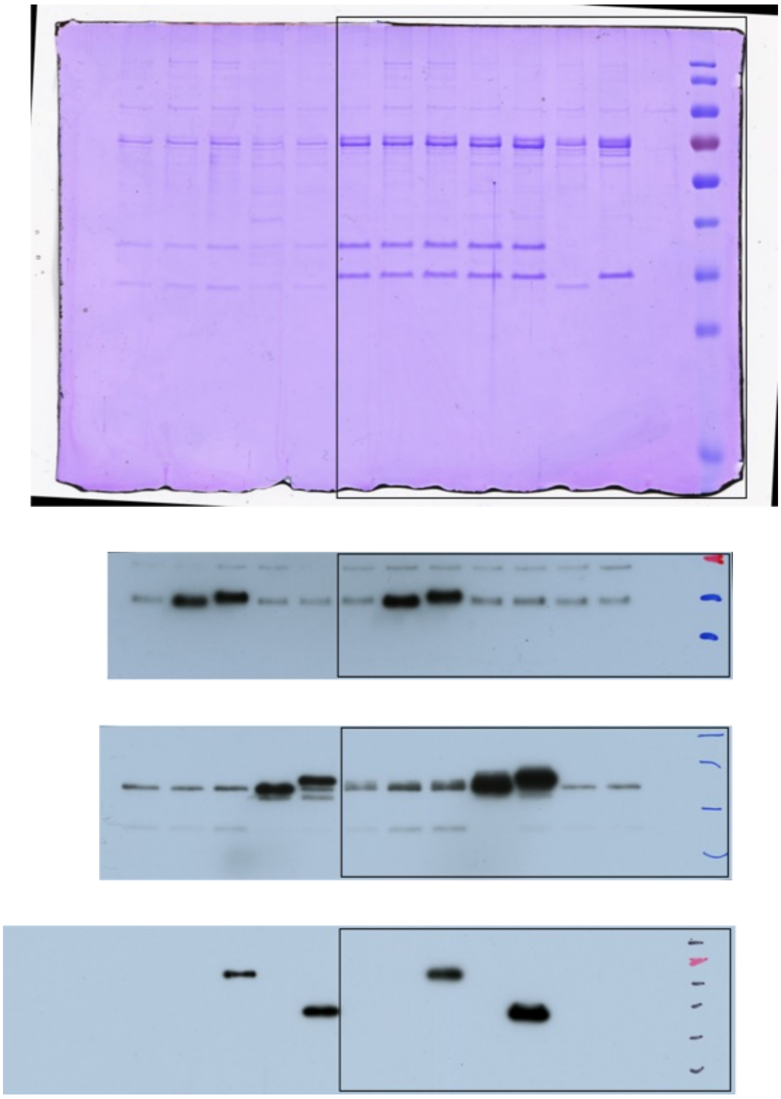

Figure 5 c. Size exclusion chromatography (SEC) analyses

Coomassie-stained SDS-PAGE gel with inputs (eluates from amylose resin) used for gel filtration on Superdex^{TM} 200 Increase 10/300 GL column

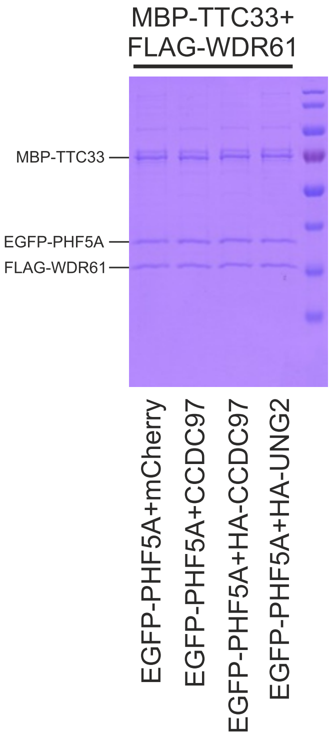

Figure 5 c. Size exclusion chromatography (SEC) analyses (continued)

SEC result ($A_{280\ nm}$) for the sample MBP-TTC33+FLAG-WDR61 / EGFP-PHF5A

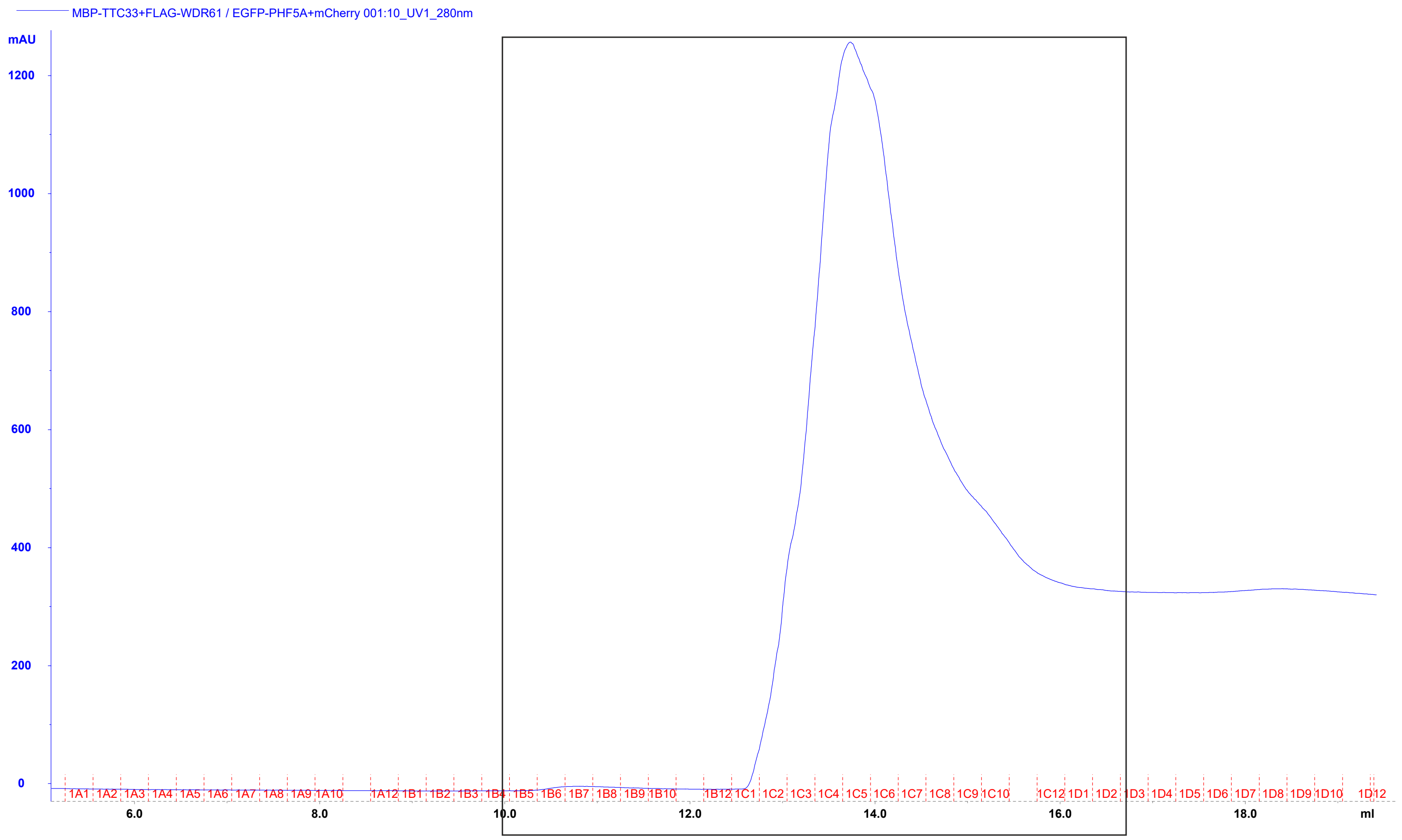

Figure 5 c. Size exclusion chromatography (SEC) analyses (continued)

SEC result ($A_{280\ nm}$) for the sample MBP-TTC33+FLAG-WDR61 / EGFP-PHF5A+CCDC97

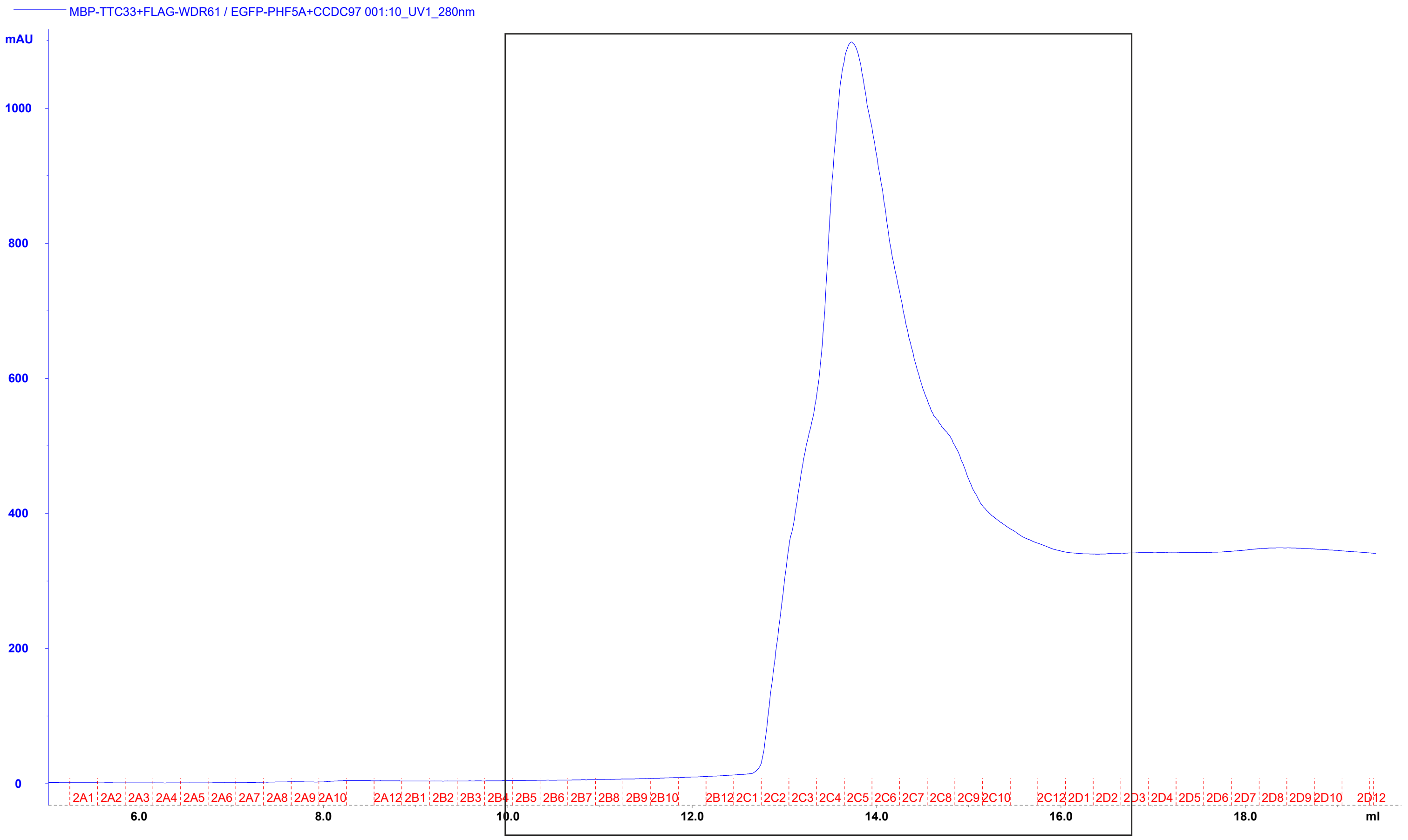

Figure 5 c. Size exclusion chromatography (SEC) analyses (continued)

SEC result ($A_{280\ nm}$) for the sample MBP-TTC33+FLAG-WDR61 / EGFP-PHF5A+HA-CCDC97

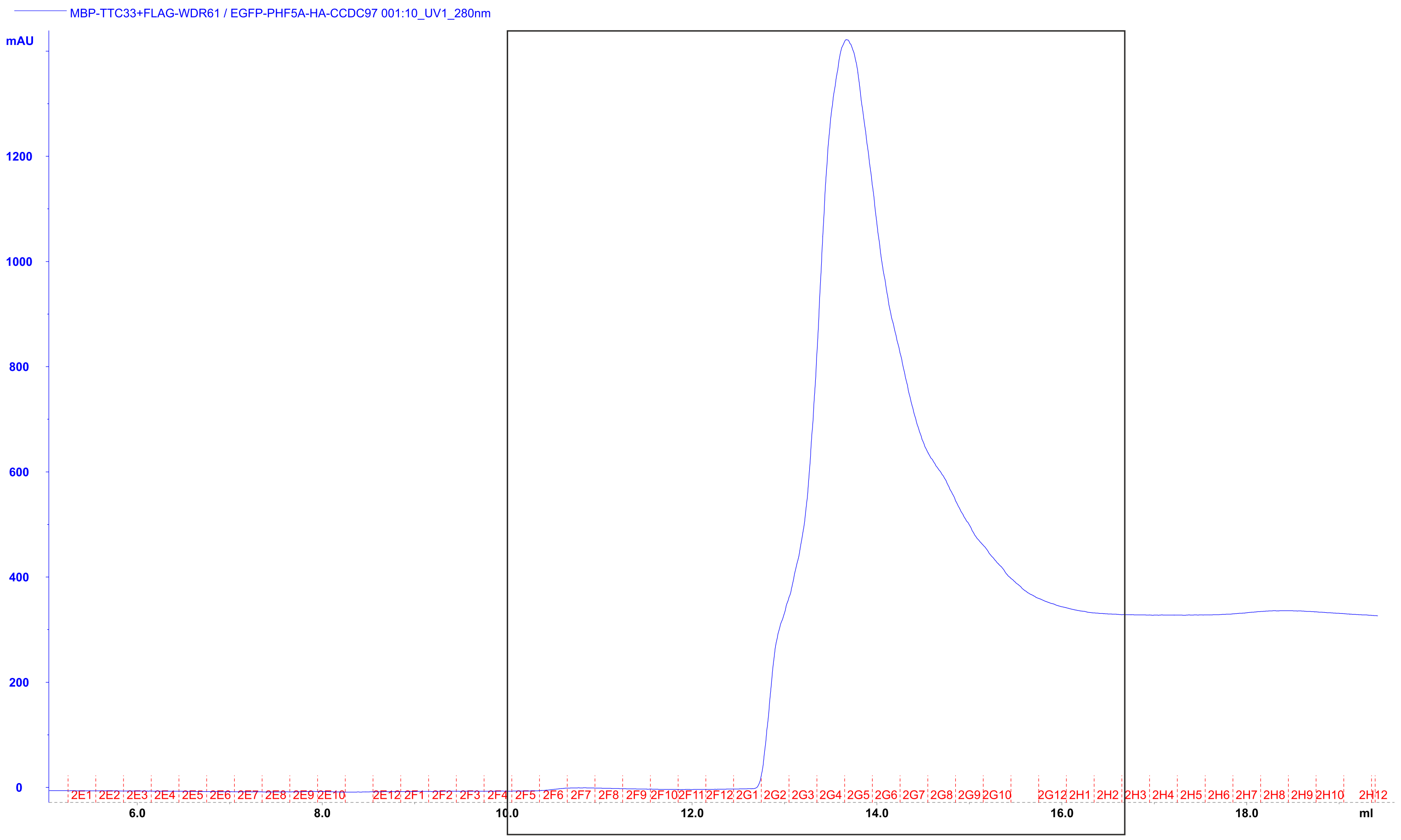

Figure 5 c. Size exclusion chromatography (SEC) analyses (continued)

SEC result ($A_{280\ nm}$) for the sample MBP-TTC33+FLAG-WDR61 / EGFP-PHF5A+HA-UNG2

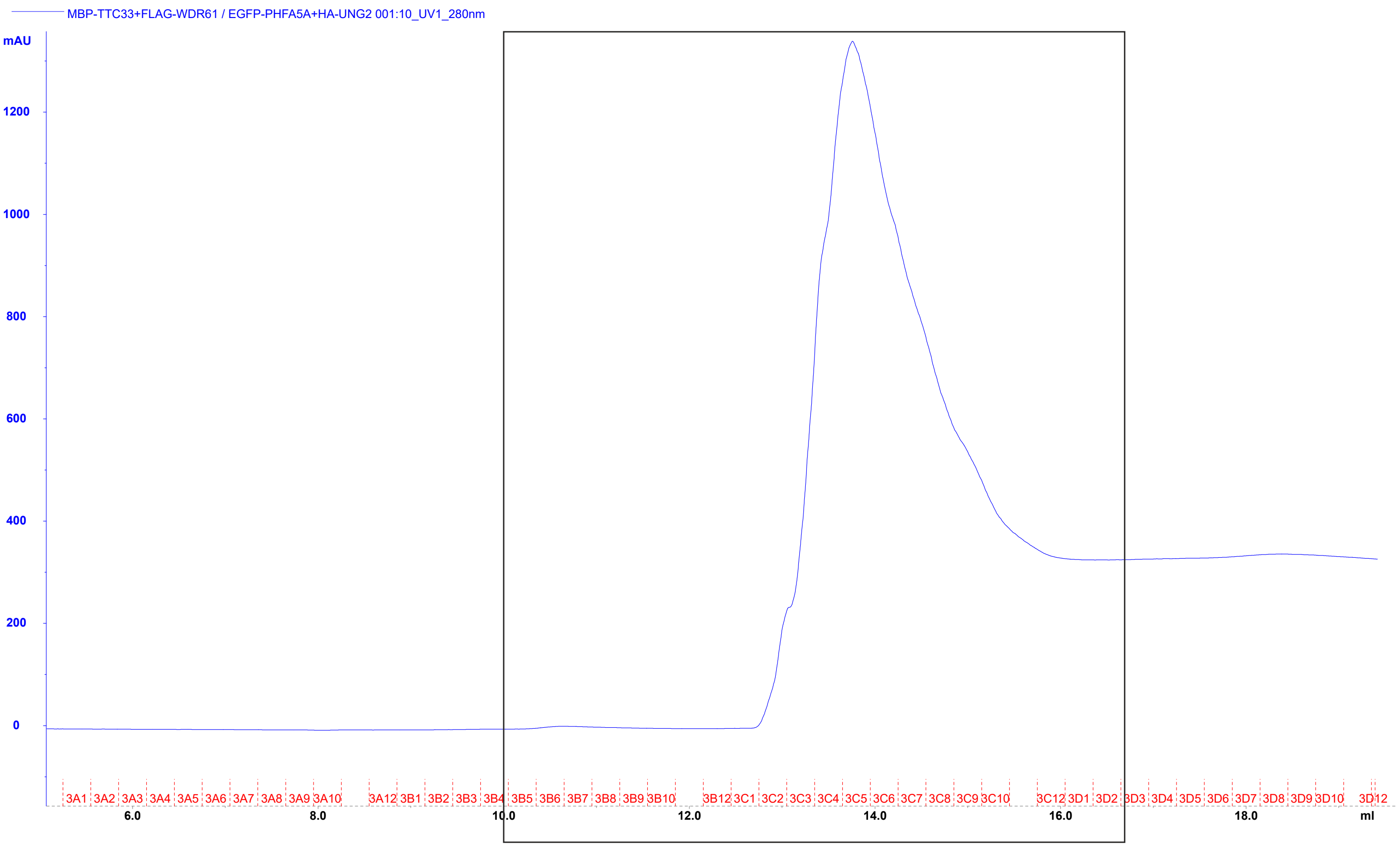

Figure 5 c. Size exclusion chromatography (SEC) analyses (continued)

Overlay of chromatograms for all 4 analyzed samples

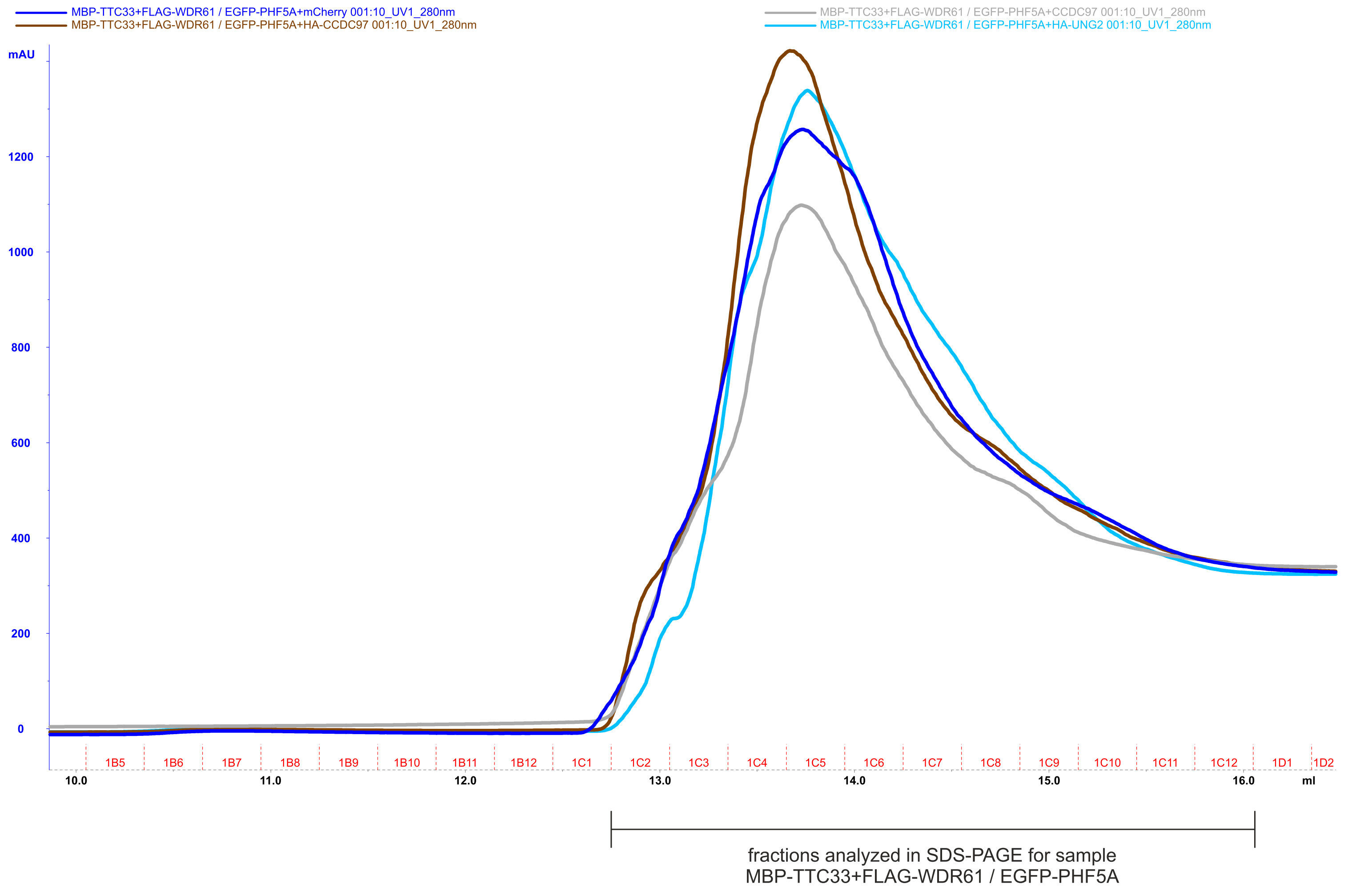

Silver-stained SDS-PAGE gel with selected SEC fractions for sample MBP-TTC33+FLAG-WDR61 / EGFP-PHF5A

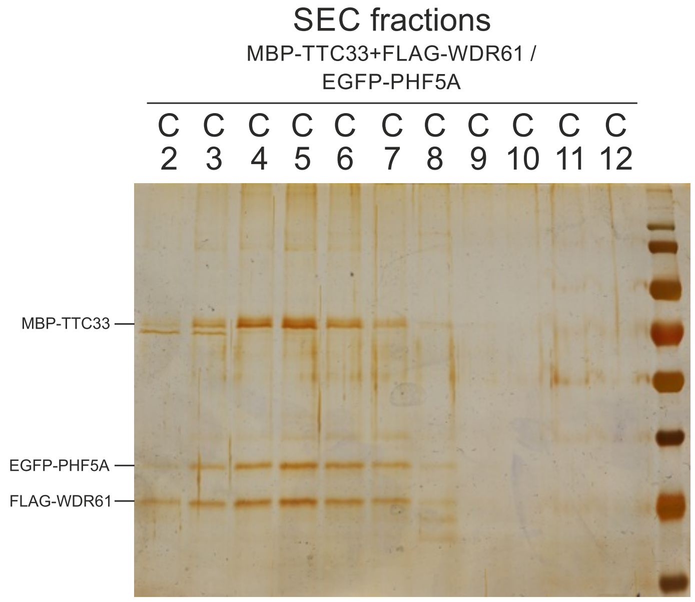

Figure 5 c. Size exclusion chromatography (SEC) analyses (original gel scan)

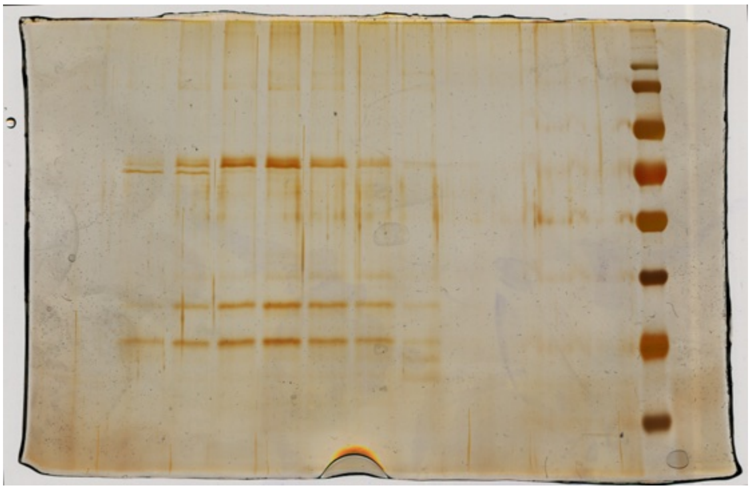

Figure 5 d. Separation of whole cell extract from FreeStyle 293-F suspension cells co-transfected with plasmids coding for MBP-TTC33 + EGFP-PHF5A + WDR61 in the glycerol gradient

membrane after transfer stained with Ponceau S red

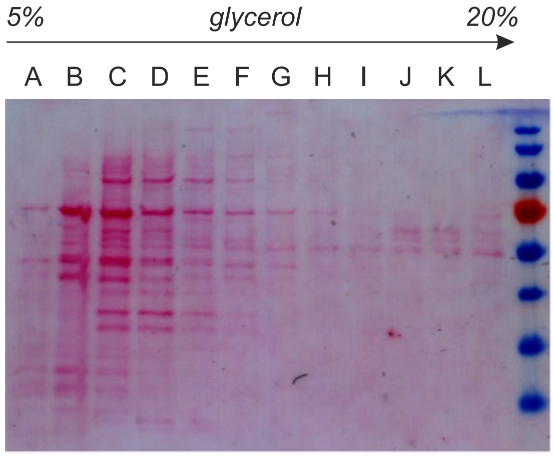

western-blot results (uncropped membranes)

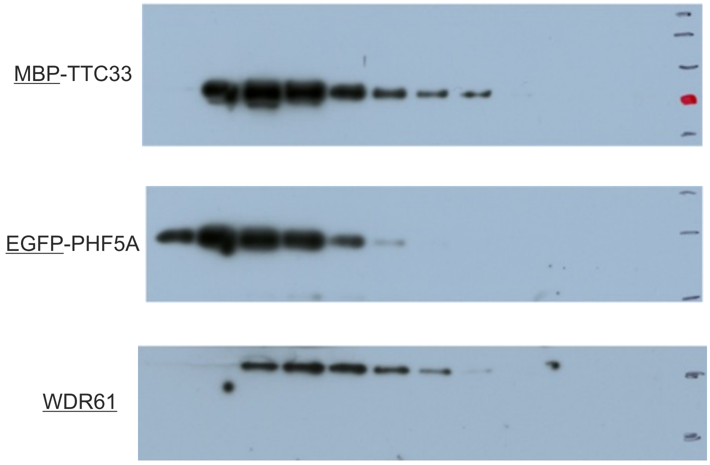

Figure 5 d. Separation of whole cell extract from FreeStyle 293-F suspension cells co-transfected with plasmids coding for MBP-TTC33 + EGFP-PHF5A + WDR61 in the glycerol gradient (original membrane scans)

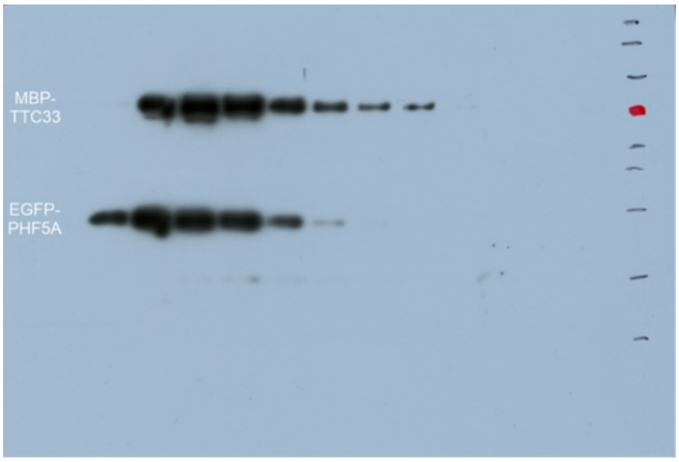

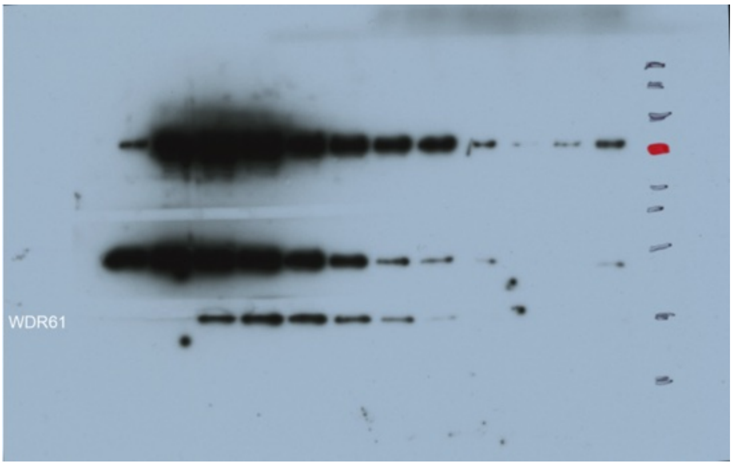

Figure 6 h. Confirmation of binary interactions within TANC

Coomassie-stained SDS-PAGE gels with eluates after affinity chromatography on amylose resin

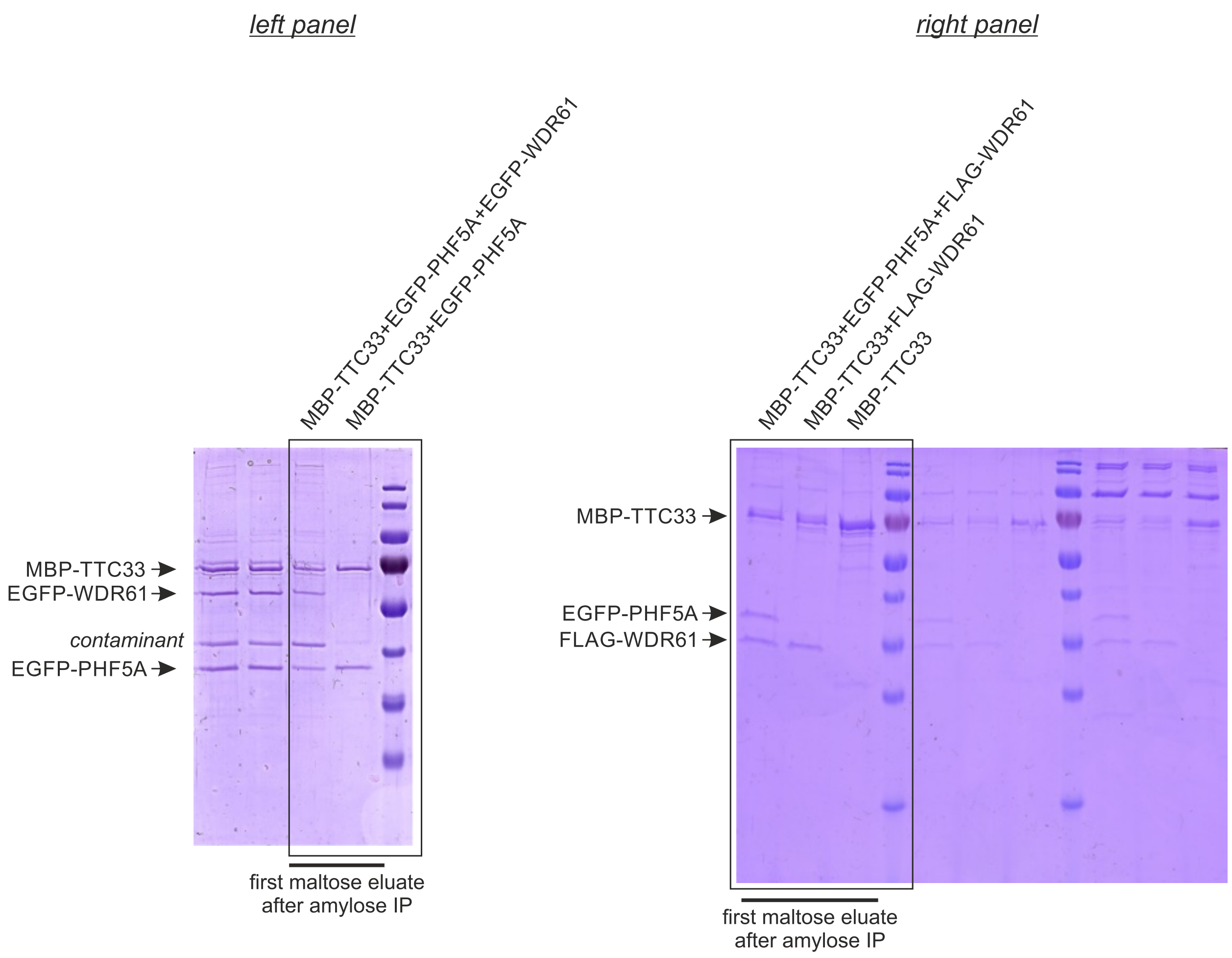

Figure 6 h. Confirmation of binary interactions within TANC (original gel scans)

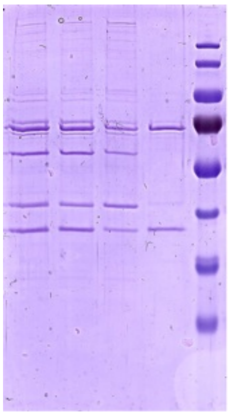

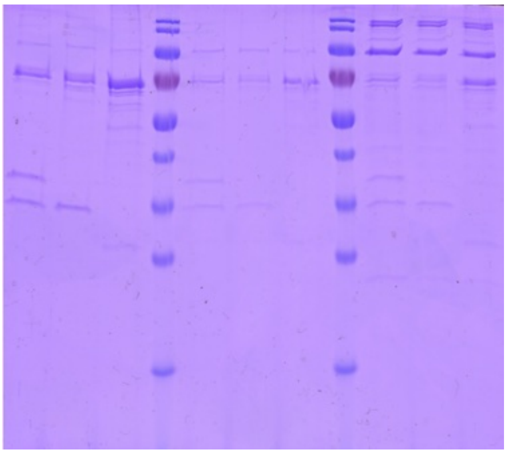

Figure 7 c. Immunoprecipitation of EGFP-tagged PHF5A WT (aa 1-110) and mut (C-terminally truncated; aa 1-101) from transiently transfected FreeStyle 293-F cells

membranes after transfer stained with Ponceau S red

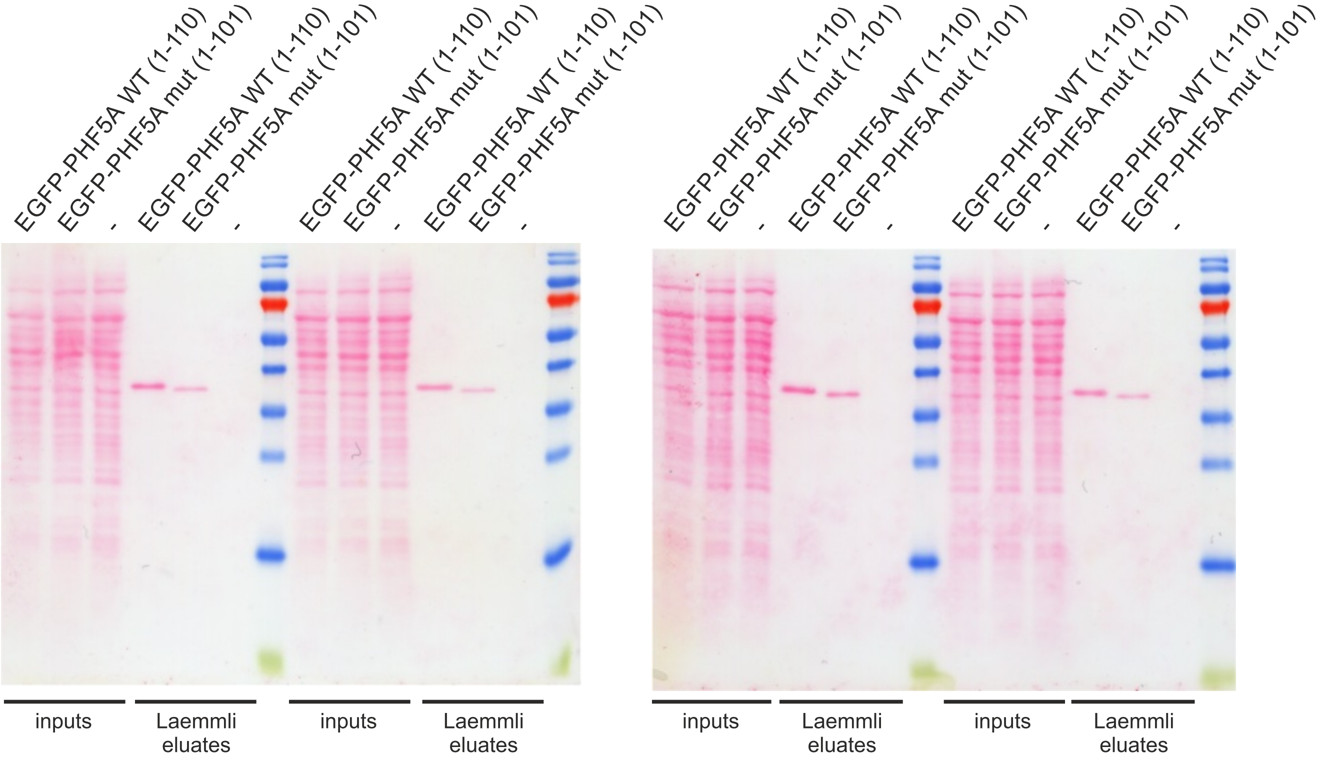

western-blot results (uncropped membranes)

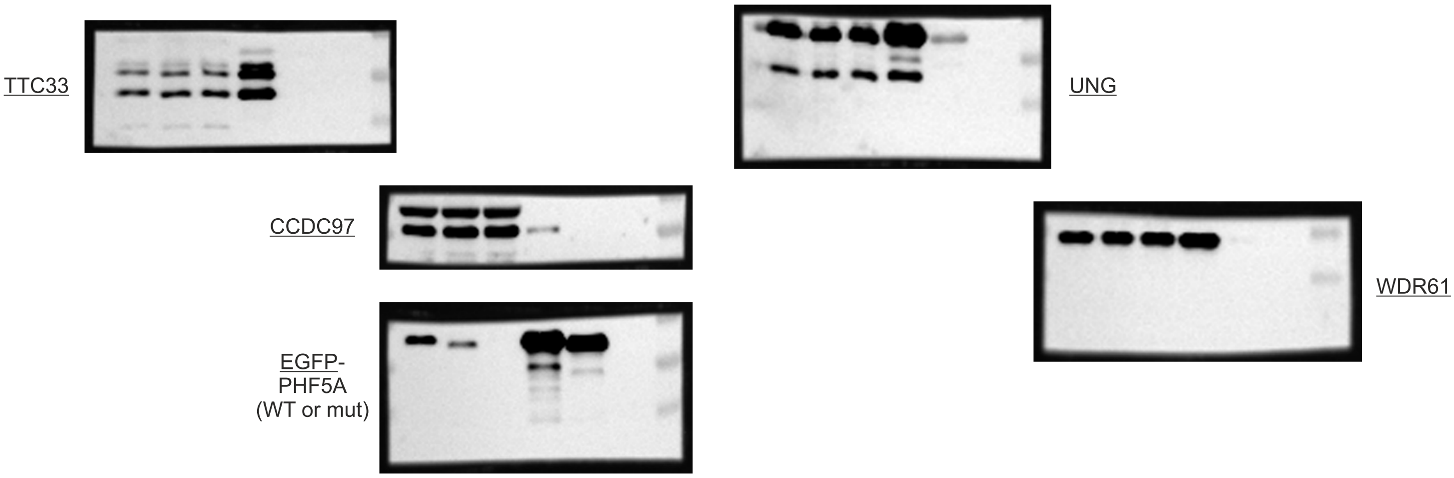

Figure 7 c. Immunoprecipitation of EGFP-tagged PHF5A WT (aa 1-110) and mut (C-terminally truncated; aa 1-101) from transiently transfected FreeStyle 293-F cells (continued)

membrane after transfer stained with Ponceau S red

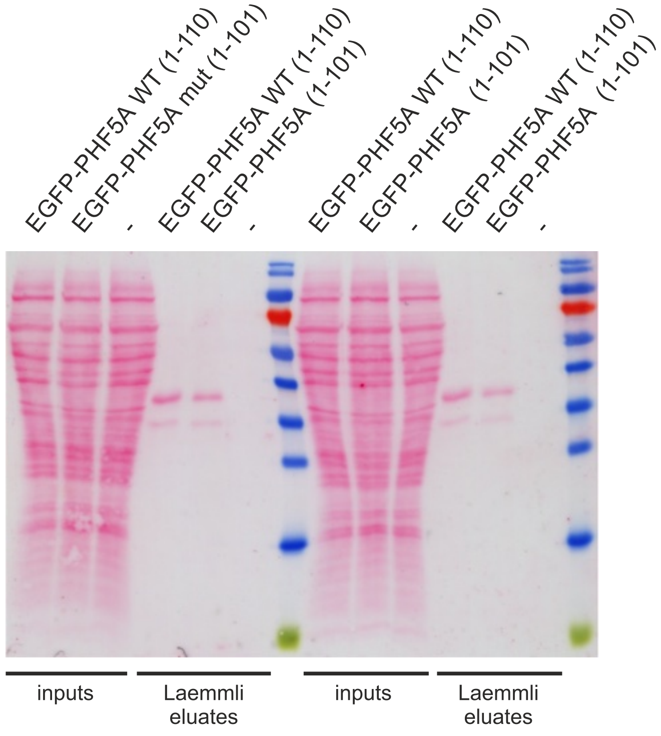

western-blot results (uncropped membrane)

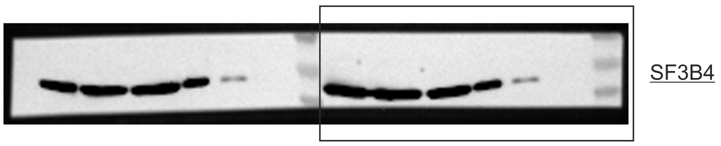

Figure 7 c. Immunoprecipitation of EGFP-tagged PHF5A WT (aa 1-110) and mut (C-terminally truncated; aa 1-101) from transiently transfected FreeStyle 293-F cells (original iBright scans)

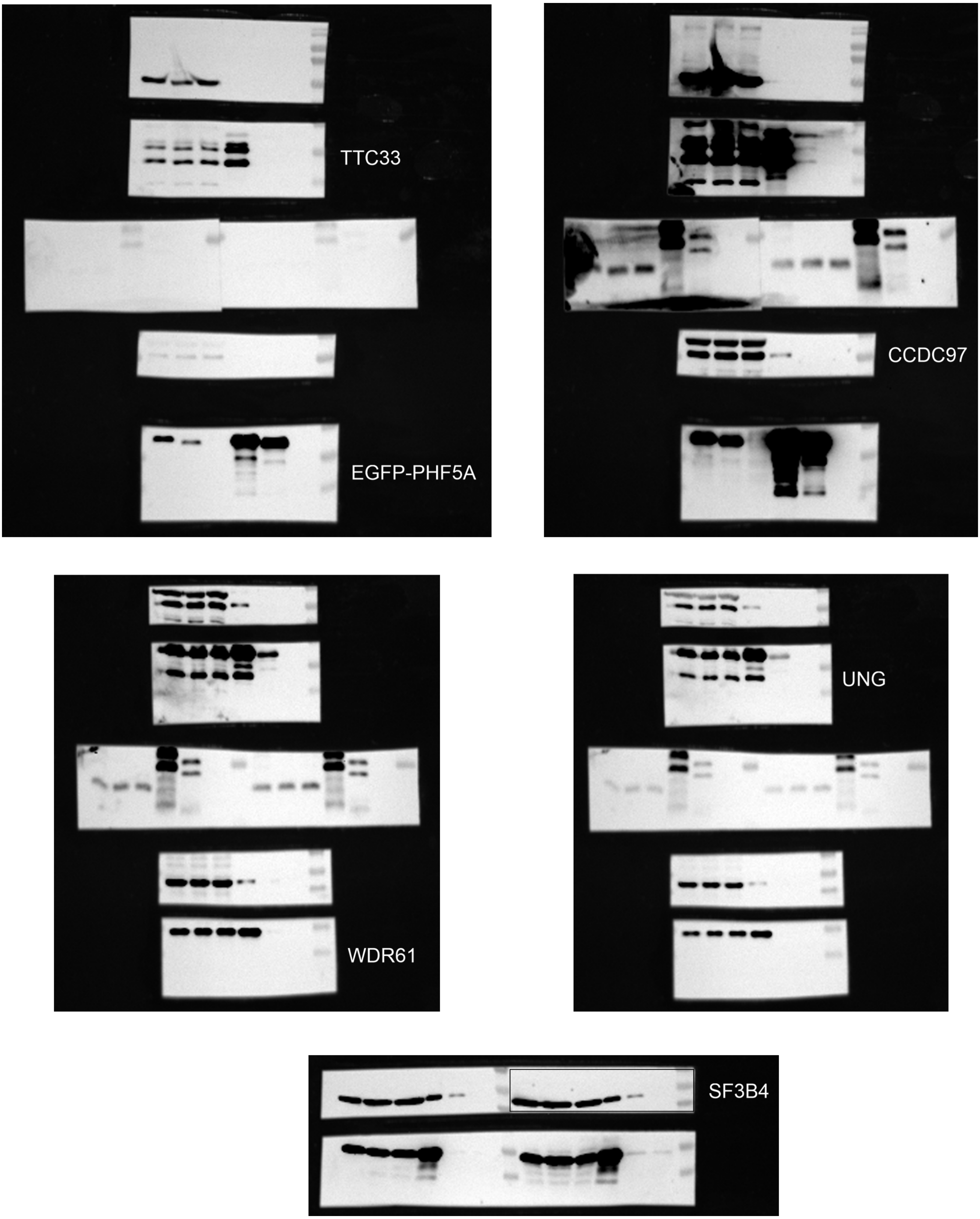

Figure 7 g. TTC33 fragments

western-blot results (uncropped membranes)

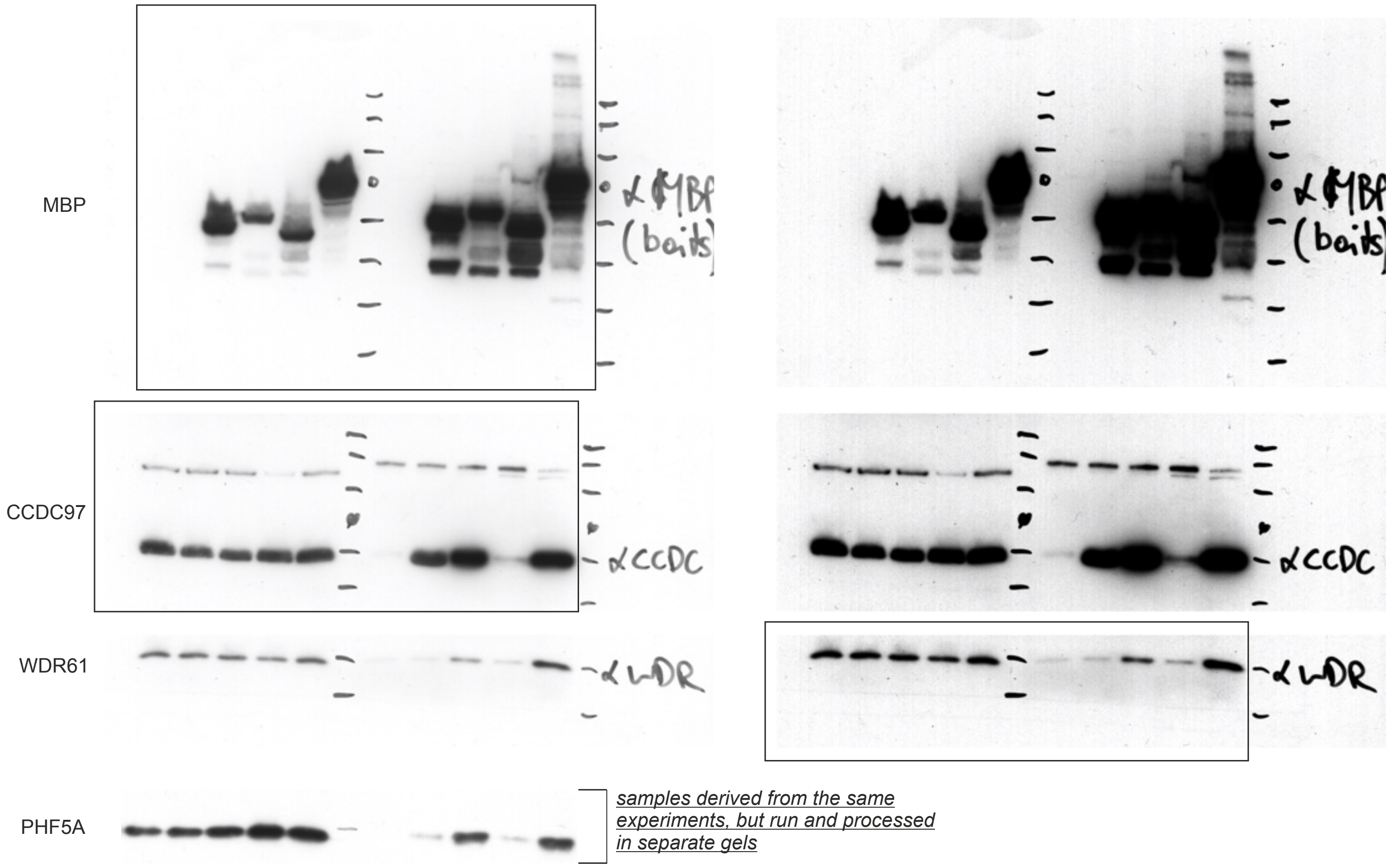

Figure 7 i. Immunoprecipitation of MBP-tagged TTC33 WT and mutants with single amino acid substitutions from 293 Flp-In T-REx stable cell lines

left panel

membranes after transfer stained with Ponceau S red

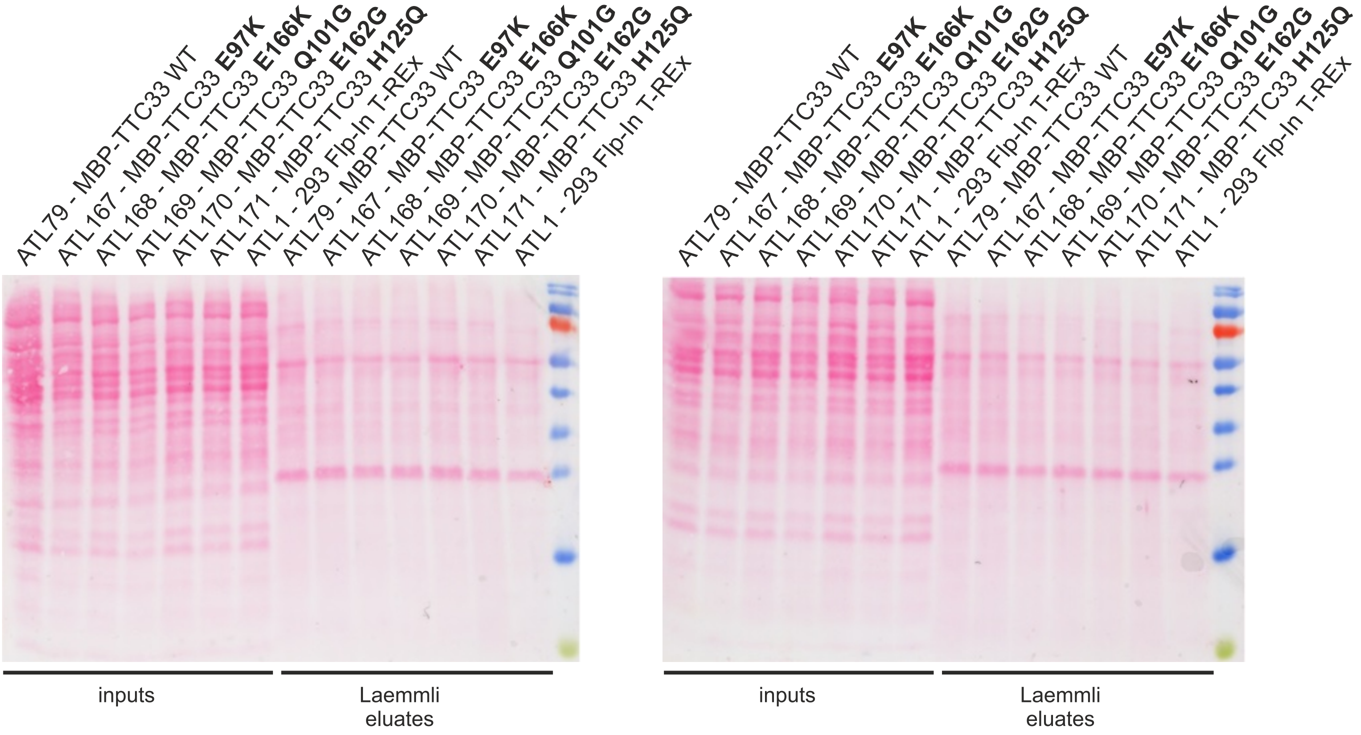

western-blot results (uncropped membranes)

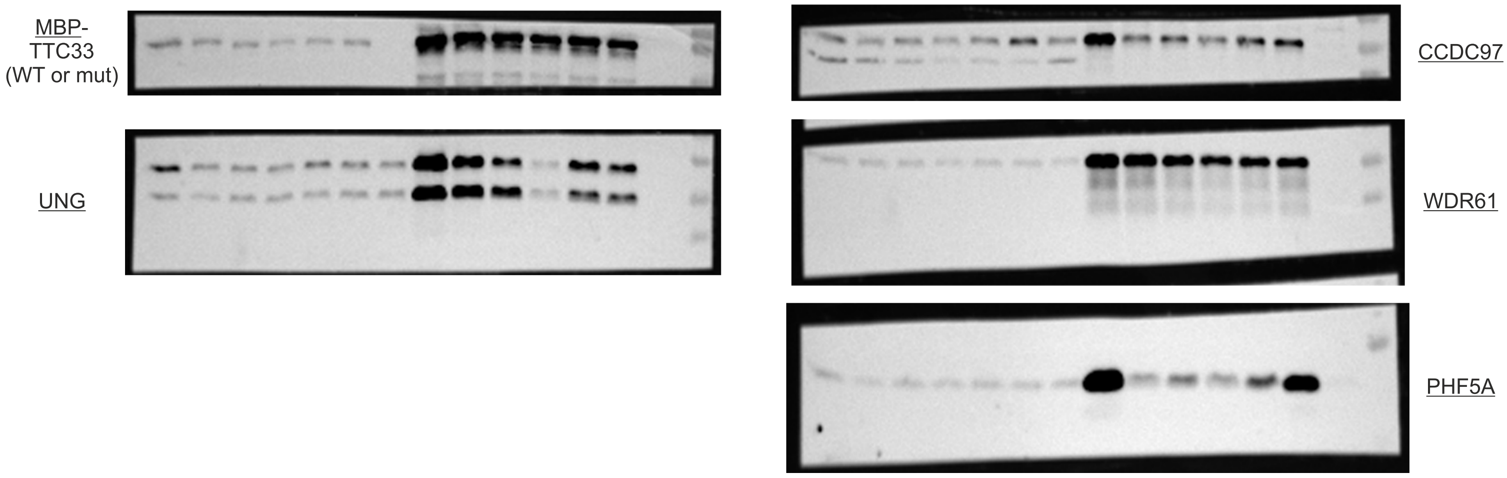

Figure 7 i. Immunoprecipitation of MBP-tagged TTC33 WT and mutants with single amino acid substitutions from 293 Flp-In T-REx stable cell lines (continued)

right panel

membranes after transfer stained with Ponceau S red

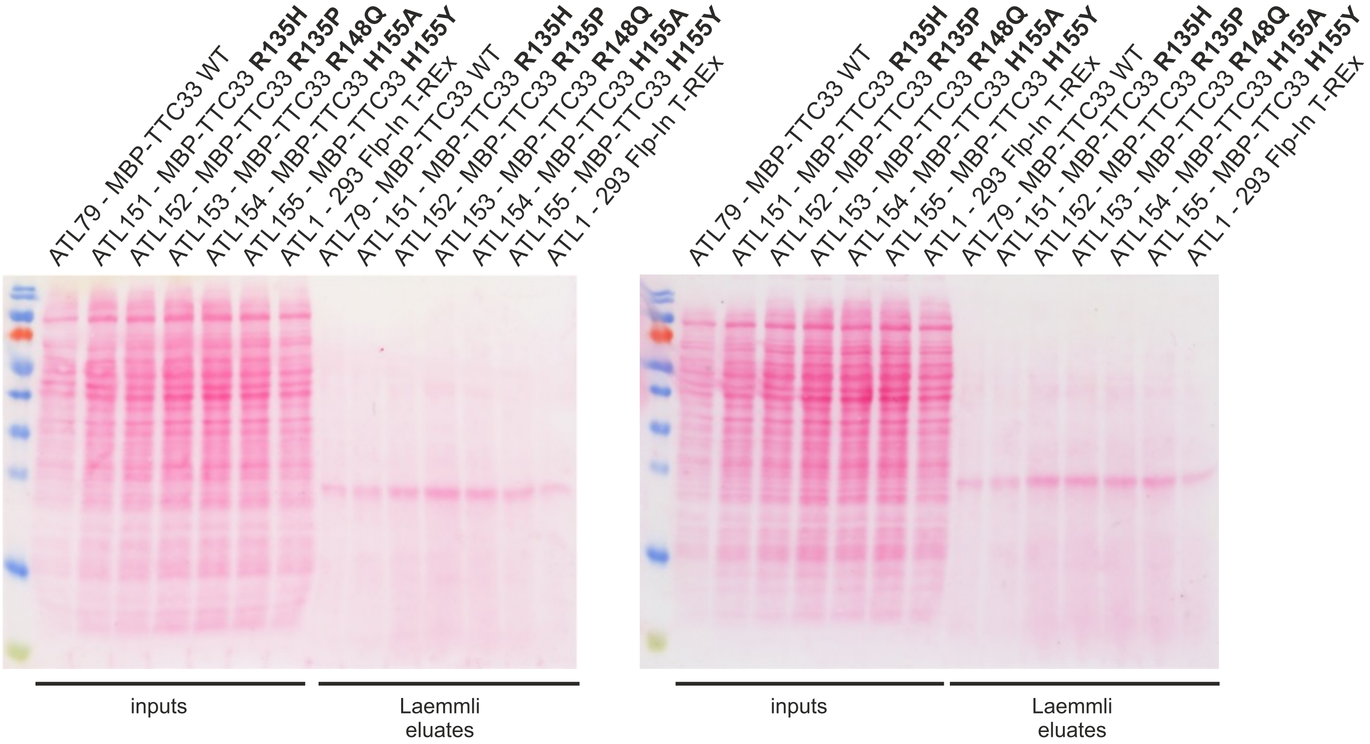

western-blot results (uncropped membranes)

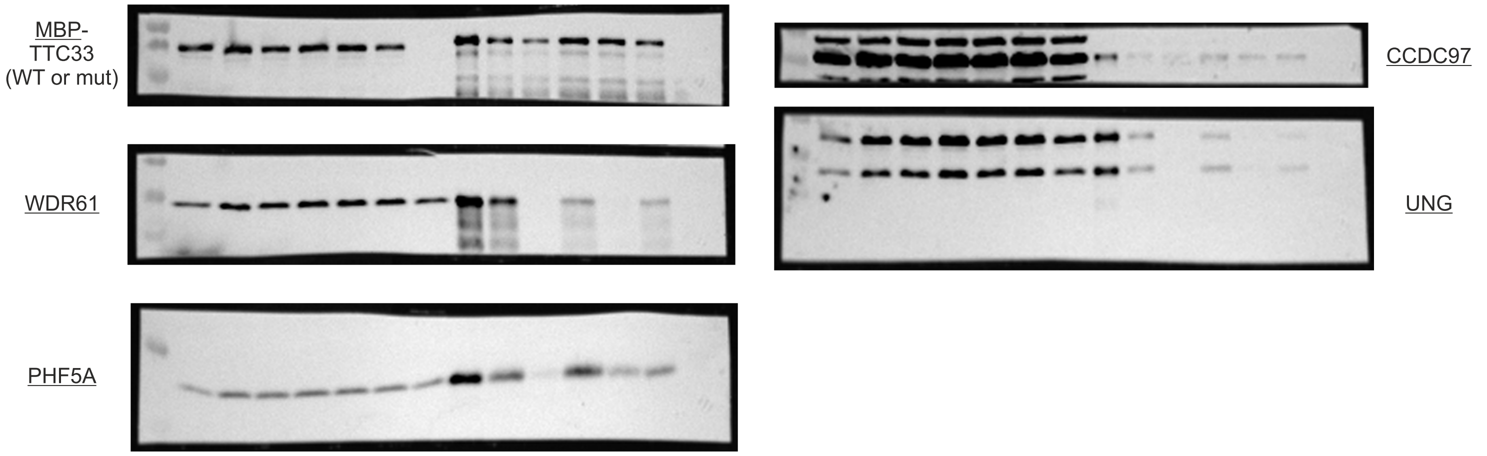

Figure 7 i. Immunoprecipitation of MBP-tagged TTC33 WT and mutants with single amino acid substitutions from 293 Flp-In T-REx stable cell lines (original iBright scans)

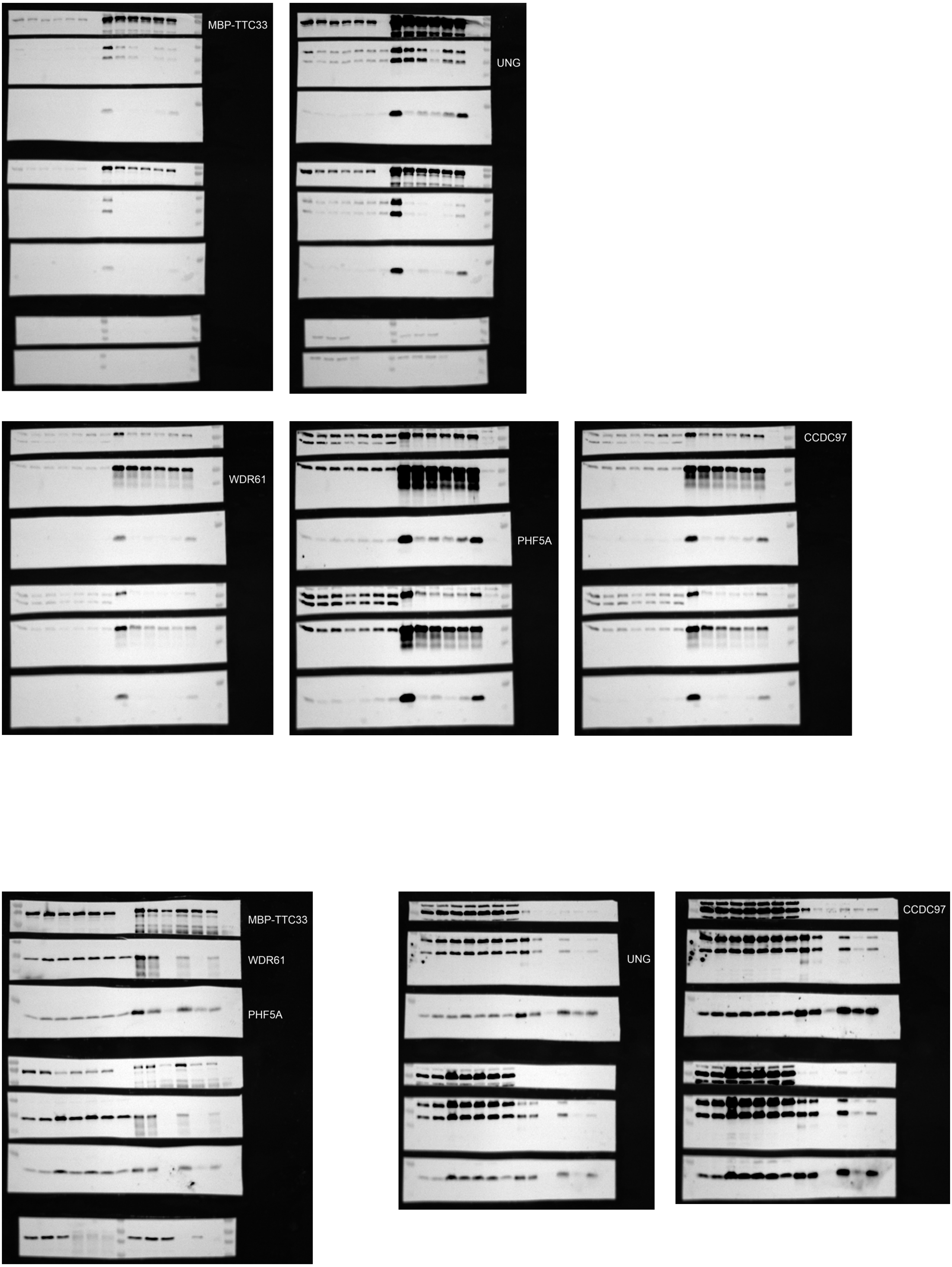

Figure 8 e. Immunoprecipitation of EGFP-tagged WDR61 WT and mut (W20C) from HeLa Flp-In T-REx stable cell lines

membranes after transfer stained with Ponceau S red

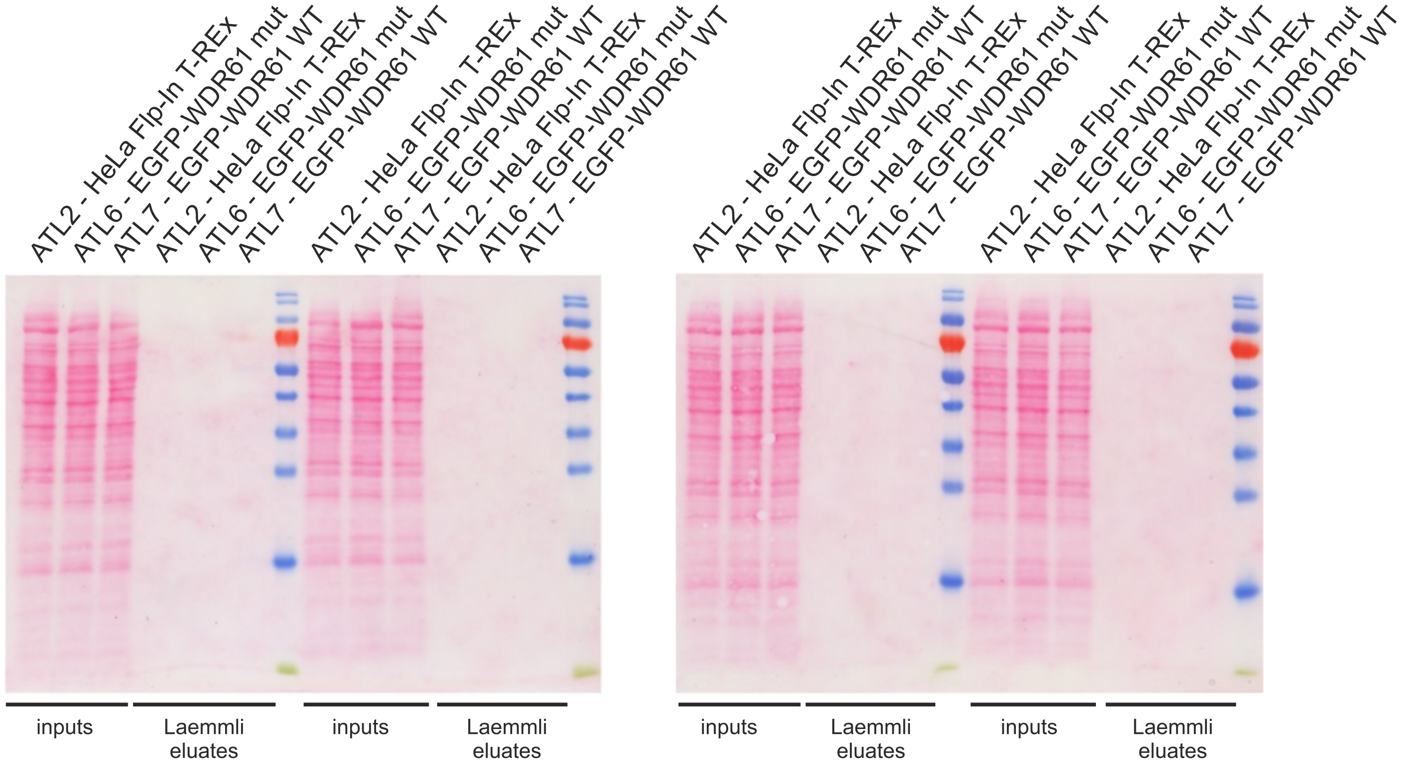

western-blot results (uncropped membranes)

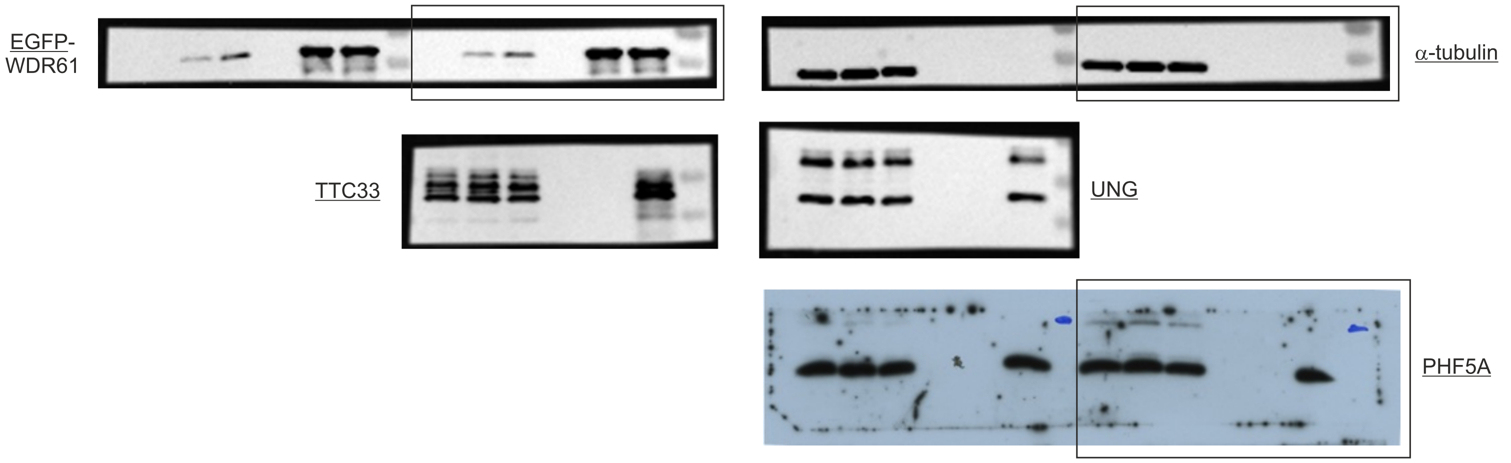

Figure 8 e. Immunoprecipitation of EGFP-tagged WDR61 WT and mut (W20C) from HeLa Flp-In T-REx stable cell lines (continued)

membranes after transfer stained with Ponceau S red

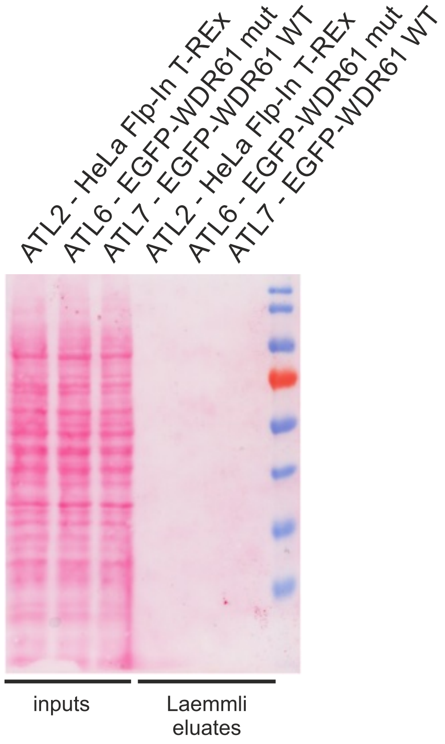

western-blot results (uncropped membrane)

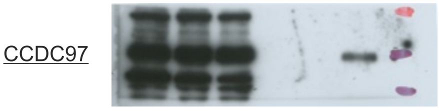

Figure 8 e. Immunoprecipitation of EGFP-tagged WDR61 WT and mut (W20C) from HeLa Flp-In T-REx stable cell lines (original scans)

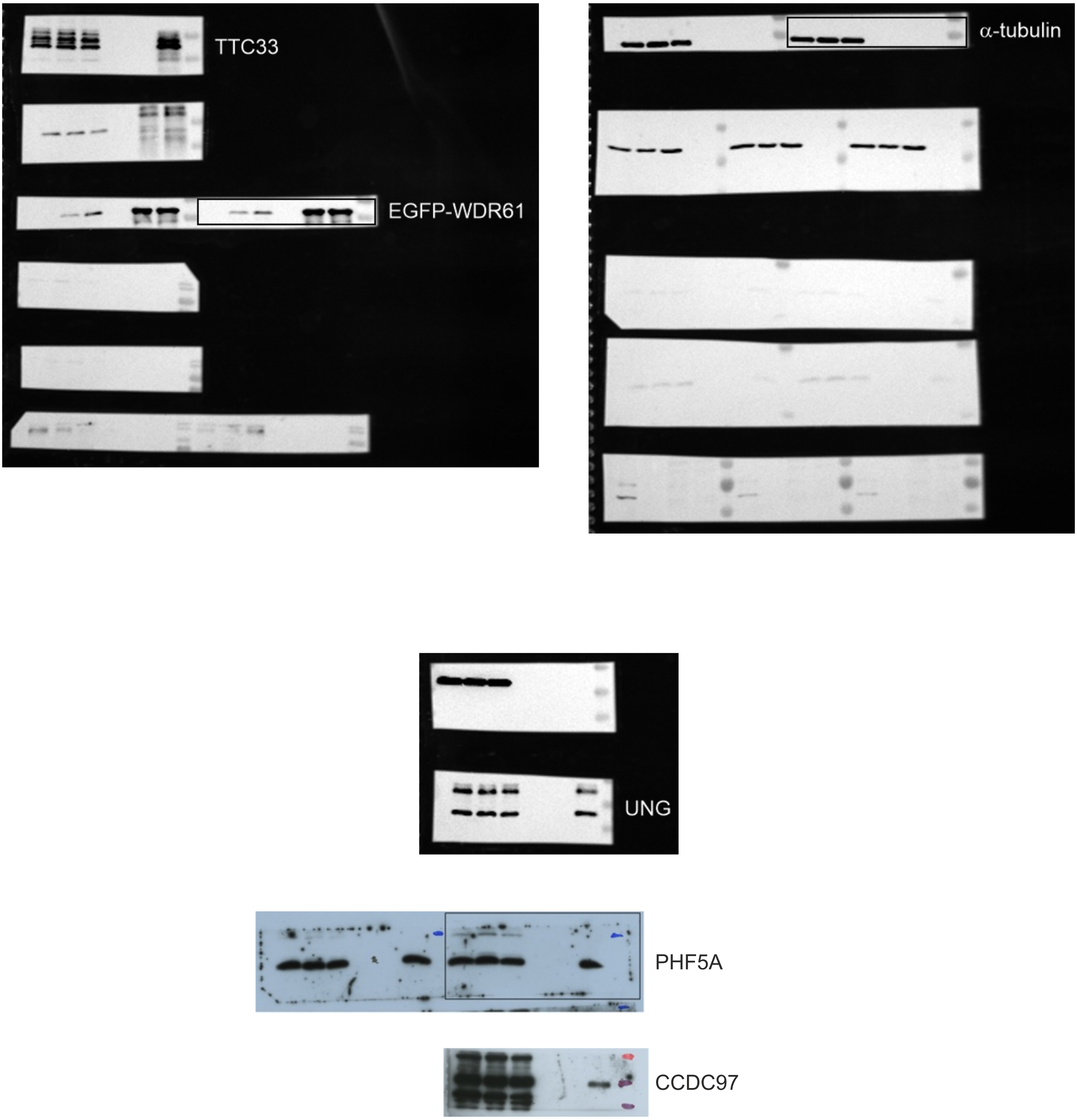

Figure 8 f. Endogenous TTC33 protein downregulation upon overexpression of tagged TTC33

western-blot results (uncropped membranes)

TTC33

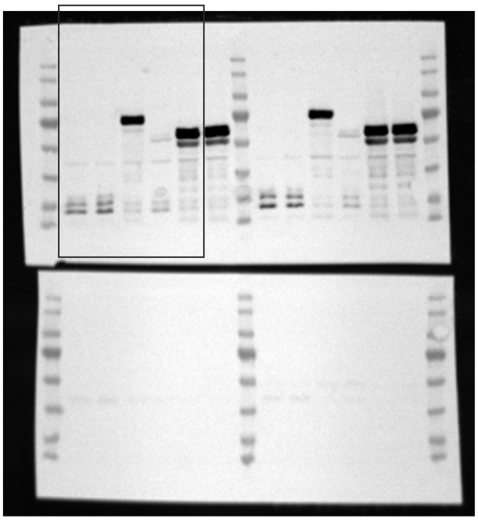

α-tubulin

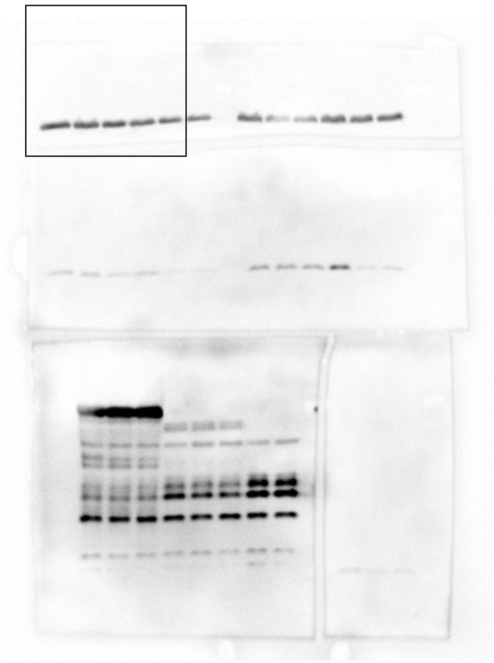

Figure 8 g. miWDR61 treated with MG132 WB to test TTC33 decay by the proteasome

western-blot results (uncropped membranes)

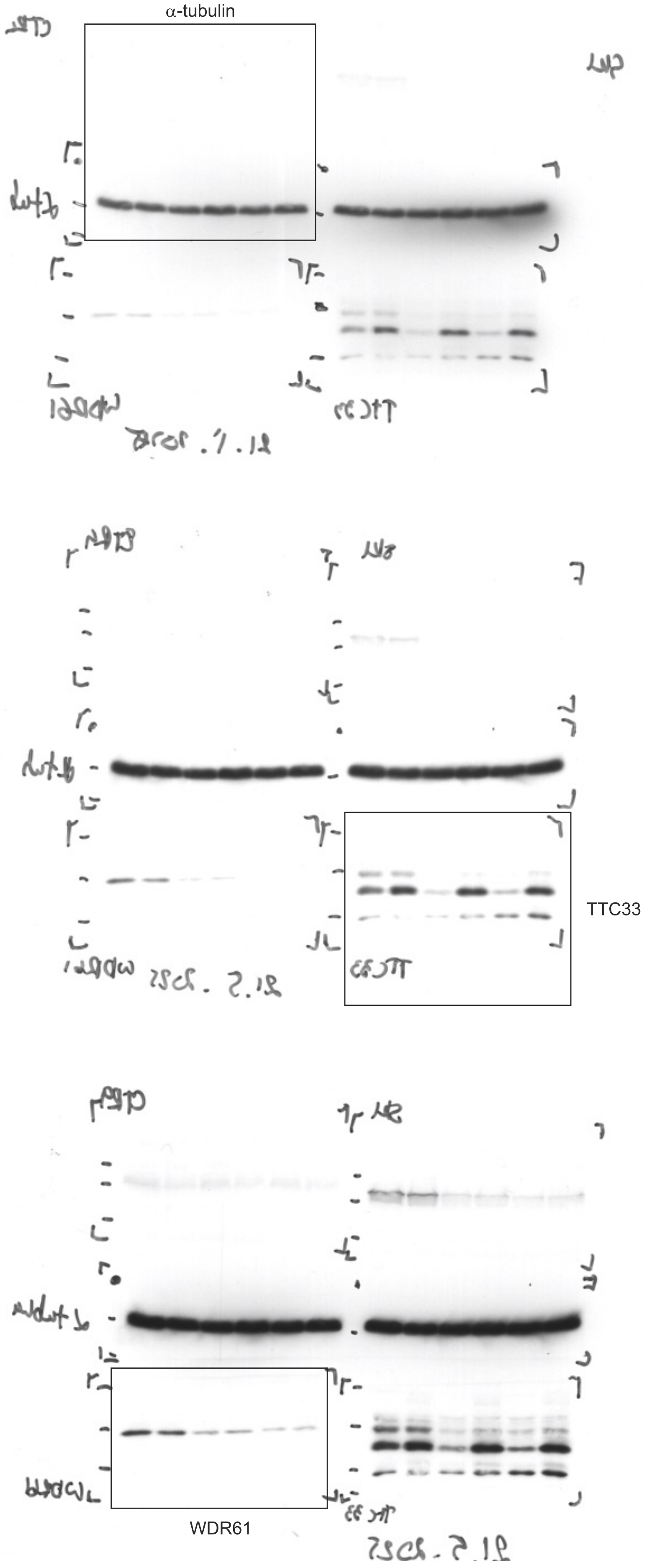

Figure 8 h. Decreased TTC33 levels upon WDR61 depletion could be complemented by ectopic expression of EGFP-tagged WDR61 WT, but not mut (W20C) protein variant

membranes after transfer stained with Ponceau S red

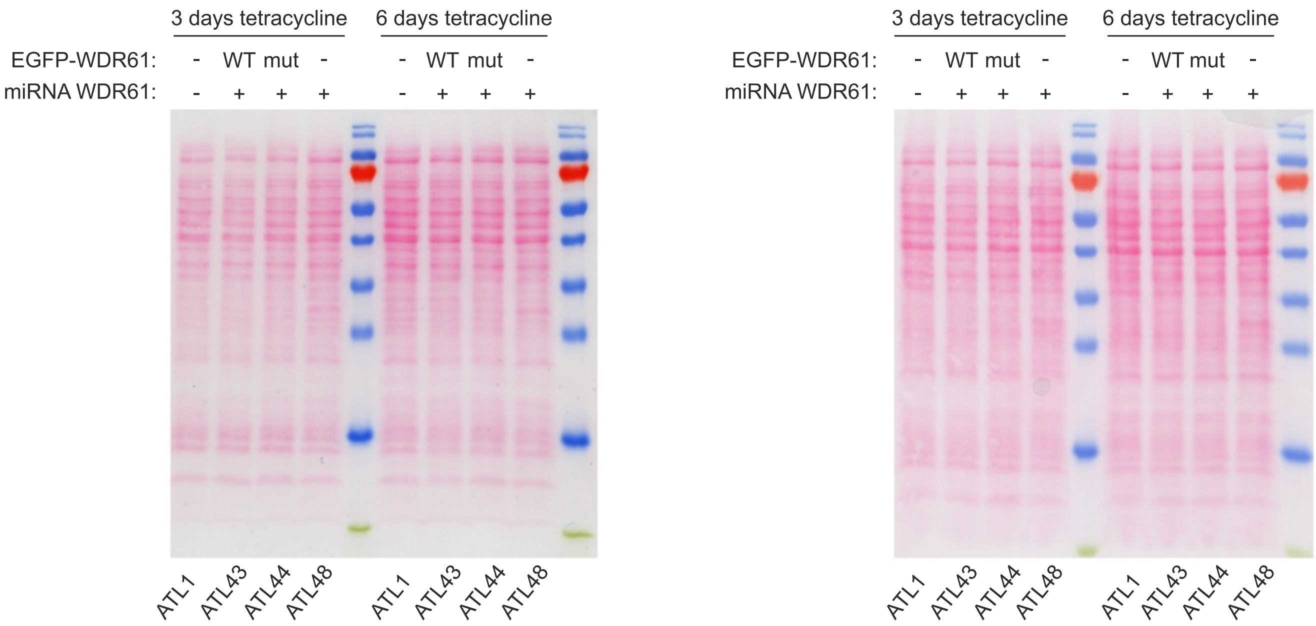

western-blot results (uncropped membranes)

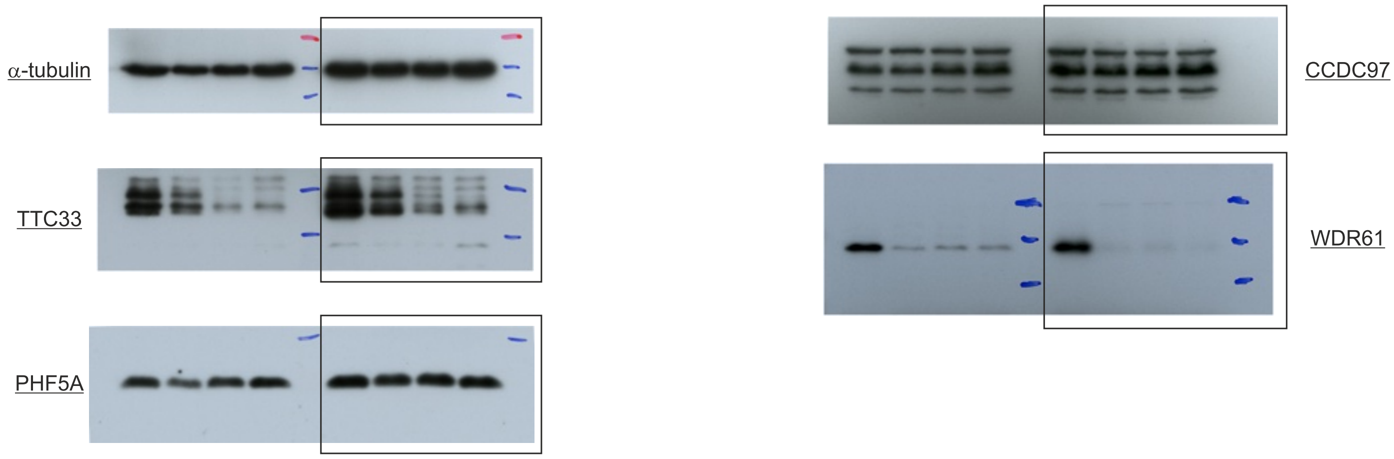

Figure 8 h. Decreased TTC33 levels upon WDR61 depletion could be complemented by ectopic expression of EGFP-tagged WDR61 WT, but not mut (W20C) protein variant (continued)

membranes after transfer stained with Ponceau S red

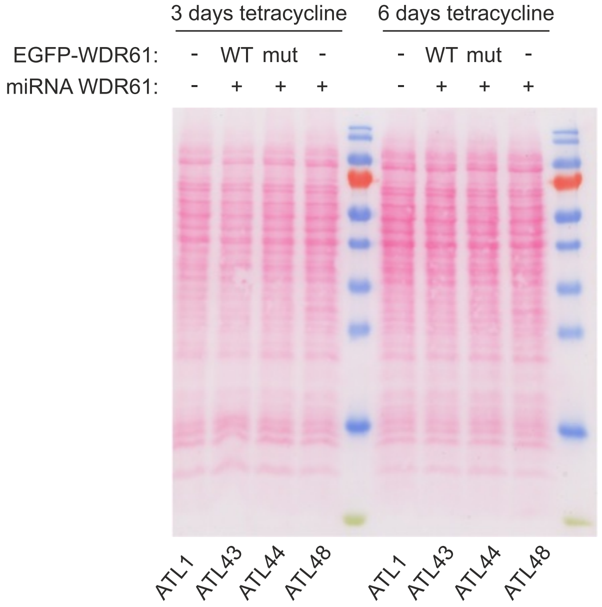

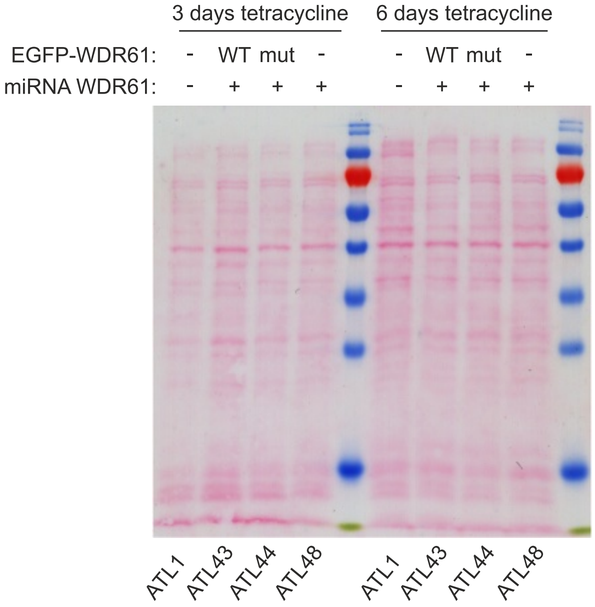

western-blot results (uncropped membranes)

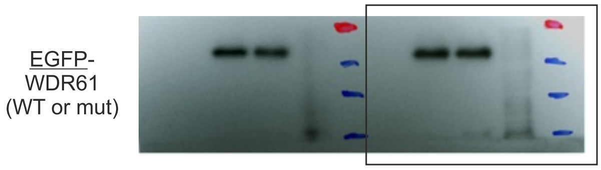

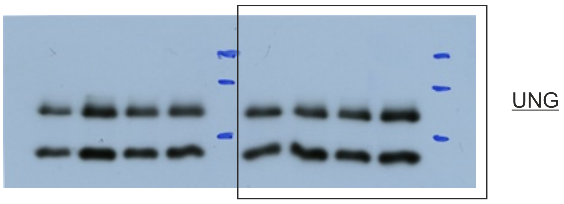

Figure 8 h. Decreased TTC33 levels upon WDR61 depletion could be complemented by ectopic expression of EGFP-tagged WDR61 WT, but not mut (W20C) protein variant (original scans)

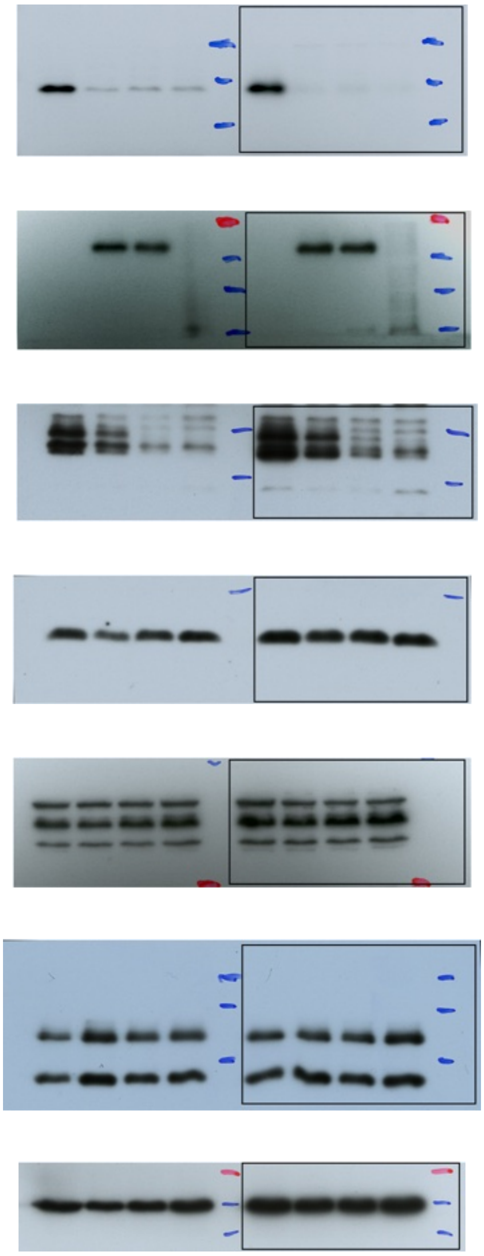

Figure 9 a. Validation of miRNA-mediated UNG, TTC33, or CCDC97 depletion

membranes after transfer stained with Ponceau S red

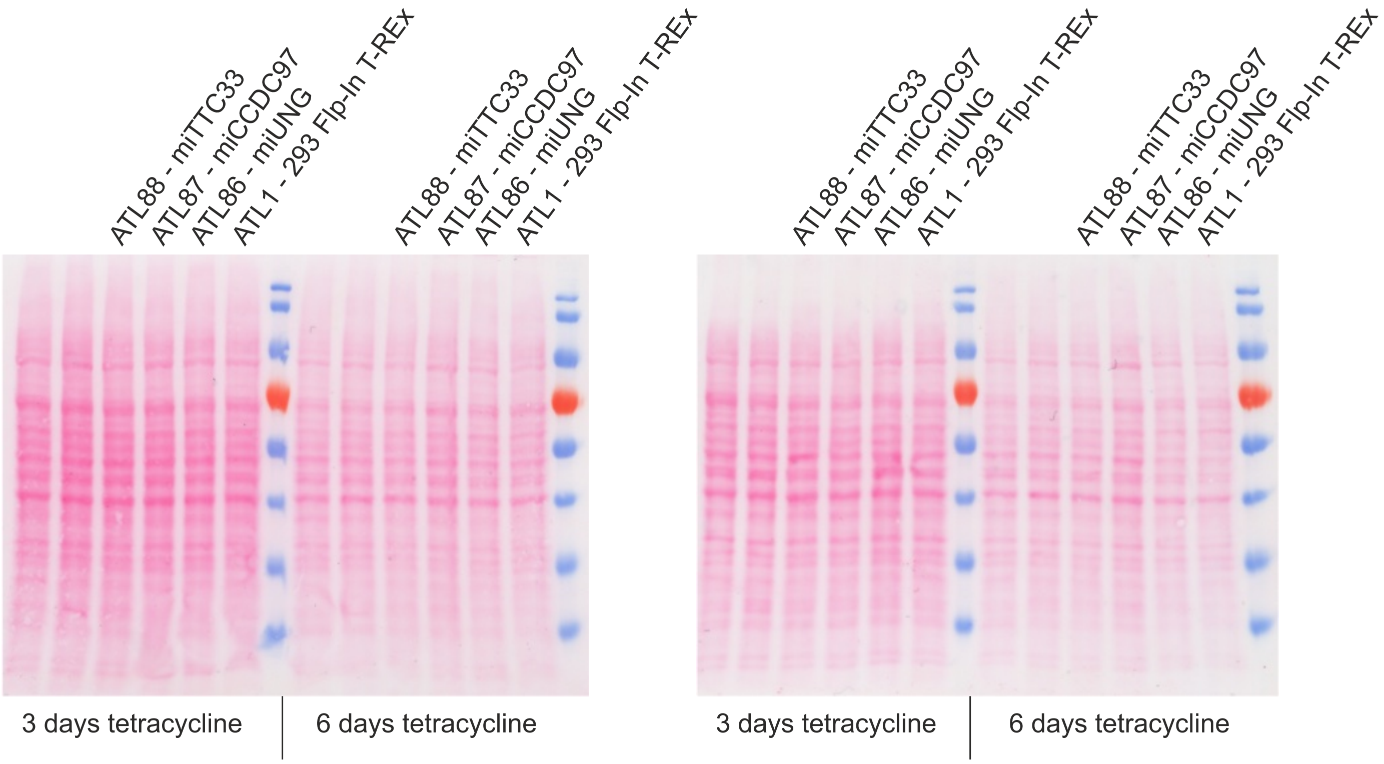

western-blot results (uncropped membranes)

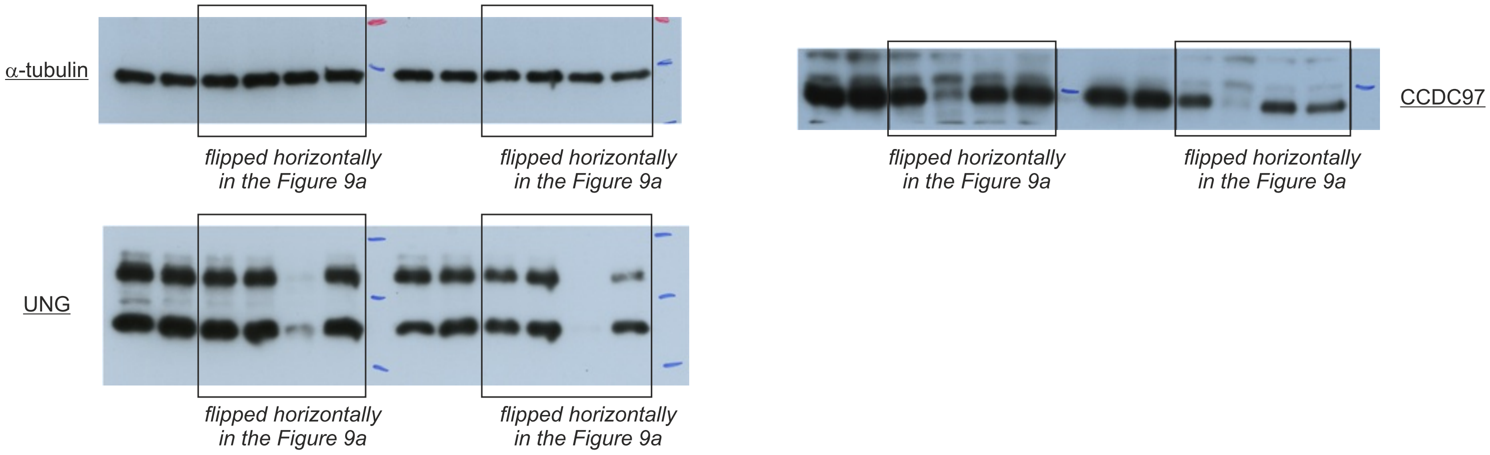

Figure 9 a. Validation of miRNA-mediated UNG, TTC33, or CCDC97 depletion (continued)

membrane after transfer stained with Ponceau S red

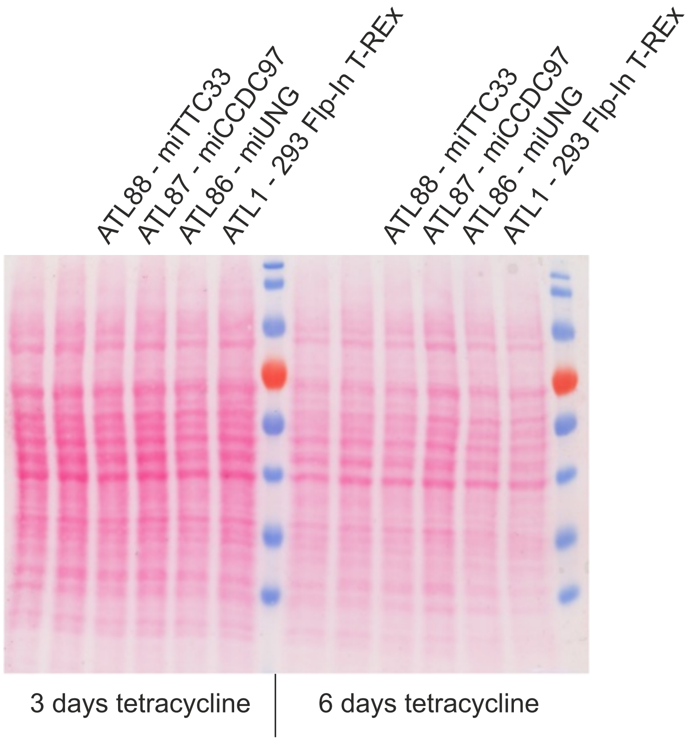

western-blot results (uncropped membrane)

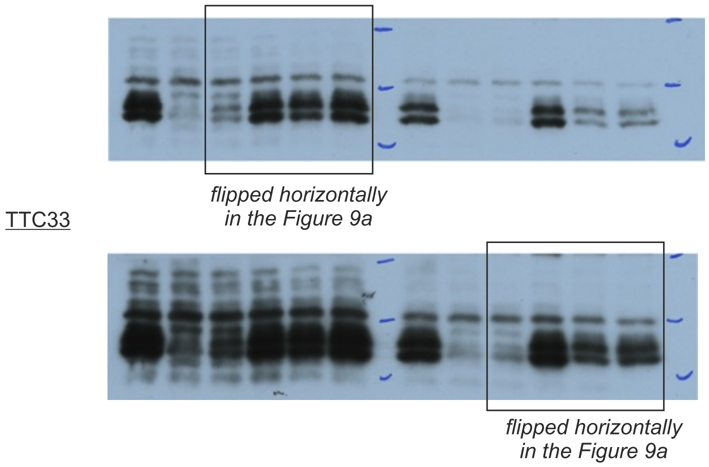

Figure 9 a. Validation of miRNA-mediated UNG, TTC33, or CCDC97 depletion (original scans)

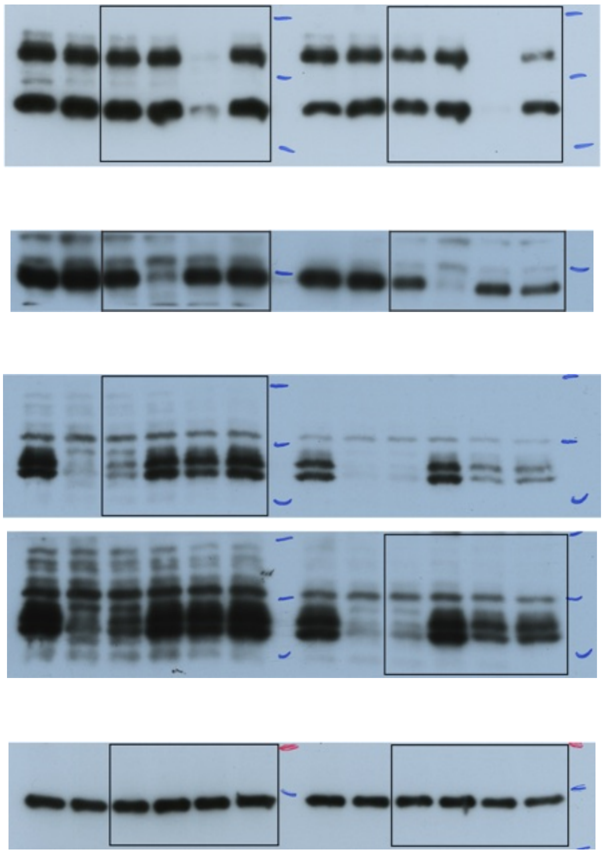

Figure 9 d (*upper part*). Verification of TTC33 or CCDC97 Crispr-Cas9 knock-outs in single clones at the protein level using western blot

membranes after transfer stained with Ponceau S red

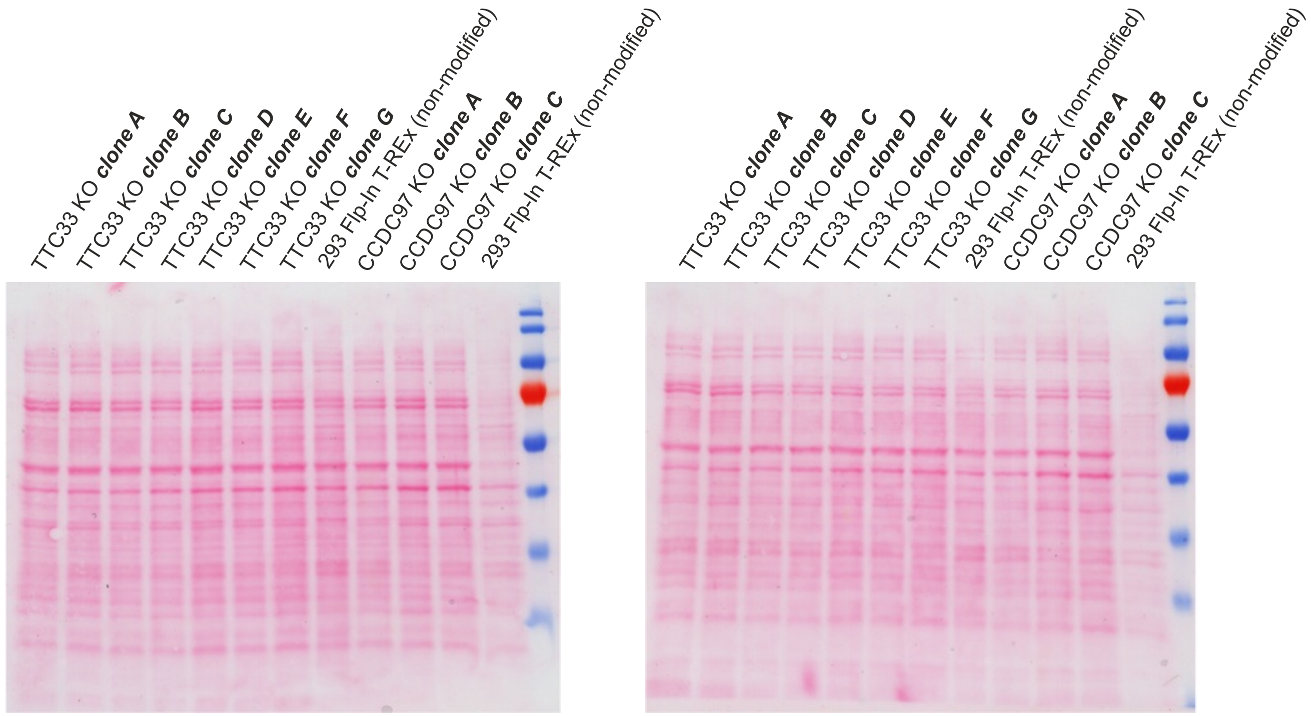

western-blot results (uncropped membranes)

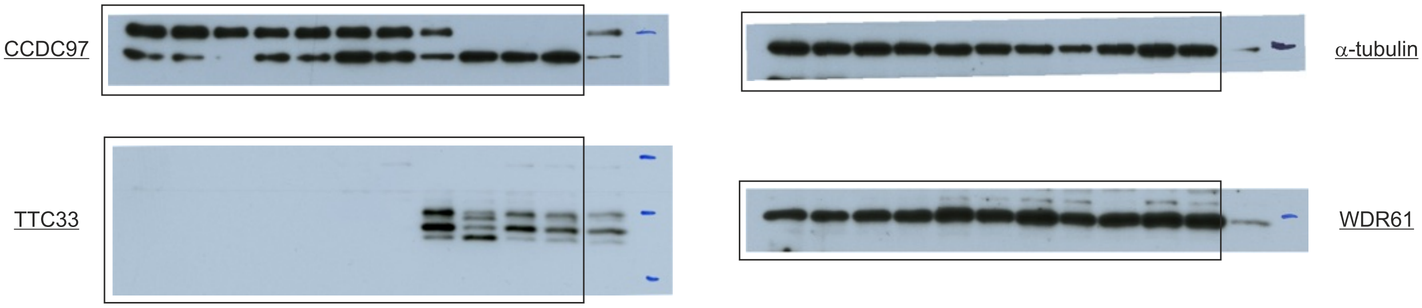

Supplement: Supplementary file 5 [file 41467_2026_76928_MOESM11_ESM.zip › Source_Data_2_main_merged.pdf]
